# Structure–metabolism relationships of 4-pentenyl synthetic cannabinoid receptor agonists using in vitro human hepatocyte incubations and high-resolution mass spectrometry

**DOI:** 10.1007/s00204-025-04080-6

**Published:** 2025-05-20

**Authors:** Steven R. Baginski, Karin Lindbom, Bryan Valencia Crespo, Ghidaa Bessa, Tobias Rautio, Xiongyu Wu, Johan Dahlén, Lorna A. Nisbet, Craig McKenzie, Henrik Gréen

**Affiliations:** 1 Leverhulme Research Centre for Forensic Science, School of Science and Engineering, University of Dundee, Dundee, UK; 2 Division of Clinical Chemistry and Pharmacology, Department of Biomedical and Clinical Sciences, Linköping University, Linköping, Sweden; 3 Department of Physics, Chemistry and Biology, Linköping University, Linköping, Sweden; 4 Chiron AS, Trondheim, Norway; 5 Department of Forensic Genetics and Forensic Toxicology, National Board of Forensic Medicine, Linköping, Sweden; 6 Present Address: Drug Discovery Unit, School of Life Sciences, University of Dundee, Dundee, UK

**Keywords:** Forensic toxicology, New psychoactive substances, Synthetic cannabinoid receptor agonists, Biotransformation, Human hepatocytes, Liquid chromatography-mass spectrometry

## Abstract

The rapid structural evolution and extensive metabolism of synthetic cannabinoid receptor agonists (SCRAs) following consumption makes their detection in biological samples challenging, especially in urine. In vitro metabolite identification studies are an essential tool for identifying analytical targets to confirm SCRA consumption in clinical and forensic toxicology casework. Systematic studies on structurally related SCRAs allow structure–metabolism relationships (SMRs) to be determined, helping to predict the metabolites of emerging and future compounds. In this study, a series of amino acid-derived 4-pentenyl SCRAs and the OXIZID 4-pentenyl SCRA BZO-4en-POXIZID were incubated at 5 µM with pooled human hepatocytes for up to 3 h. Metabolites were identified using liquid chromatography–quadrupole time-of-flight mass spectrometry (LC–QTOF-MS) and SMRs investigated using the proportion that each type of biotransformation contributed to the total abundance of metabolites for each parent compound. Metabolites were mainly produced via terminal amide/ester hydrolysis, dihydrodiol formation on the tail, hydroxylation and *N*-dealkylation, but also by ketone formation, dehydrogenation, glucuronidation and combinations thereof. Methyl valinate (MMB) and ethyl valinate (EMB) SCRAs underwent extensive ester hydrolysis (90.9–96.0%), which was less dominant for methyl *tert*-leucinate (MDMB) SCRAs (47.6–60.7%), with dihydrodiol formation (24.3–27.7%) and hydroxylation (6.2–17.3%) becoming more prominent. For AB-4en-PICA and *tert*-leucinamide (ADB) SCRAs, hydroxylation was the major metabolic pathway (47.9–58.4%), while tail dihydrodiol formation was most prominent for BZO-4en-POXIZID (61.5%). Clear SMRs were identified for 4-pentenyl SCRAs and can be used to recommend biomarkers of consumption and aid prediction of metabolites of emerging and future SCRAs.

**Supplementary Information:**

The online version contains supplementary material available at 10.1007/s00204-025-04080-6.

## Introduction

Synthetic cannabinoid receptor agonists (SCRAs) are new psychoactive substances (NPS) that bind to and activate the cannabinoid receptors, CB_1_ and CB_2_ (Advisory Council on the Misuse of Drugs 2020). Although designed to mimic Δ^9^-tetrahydrocannabinol (Δ^9^-THC), the major psychoactive component of cannabis and a partial agonist of cannabinoid receptors, SCRAs are full CB_1_ agonists and their consumption can result in altered consciousness, increased anxiety, seizures, tachycardia, heart attacks, cardiac arrest, respiratory depression and death (Darke et al. 2021; Giorgetti et al. 2020). SCRAs are currently the largest group of NPS monitored by the European Union Drugs Agency (EUDA), previously known as the European Monitoring Centre for Drugs and Drug Addiction (EMCDDA), with a total of 245 SCRAs reported by the end of 2022, including 24 new compounds that same year (EMCDDA 2023).

**Fig. 1:**
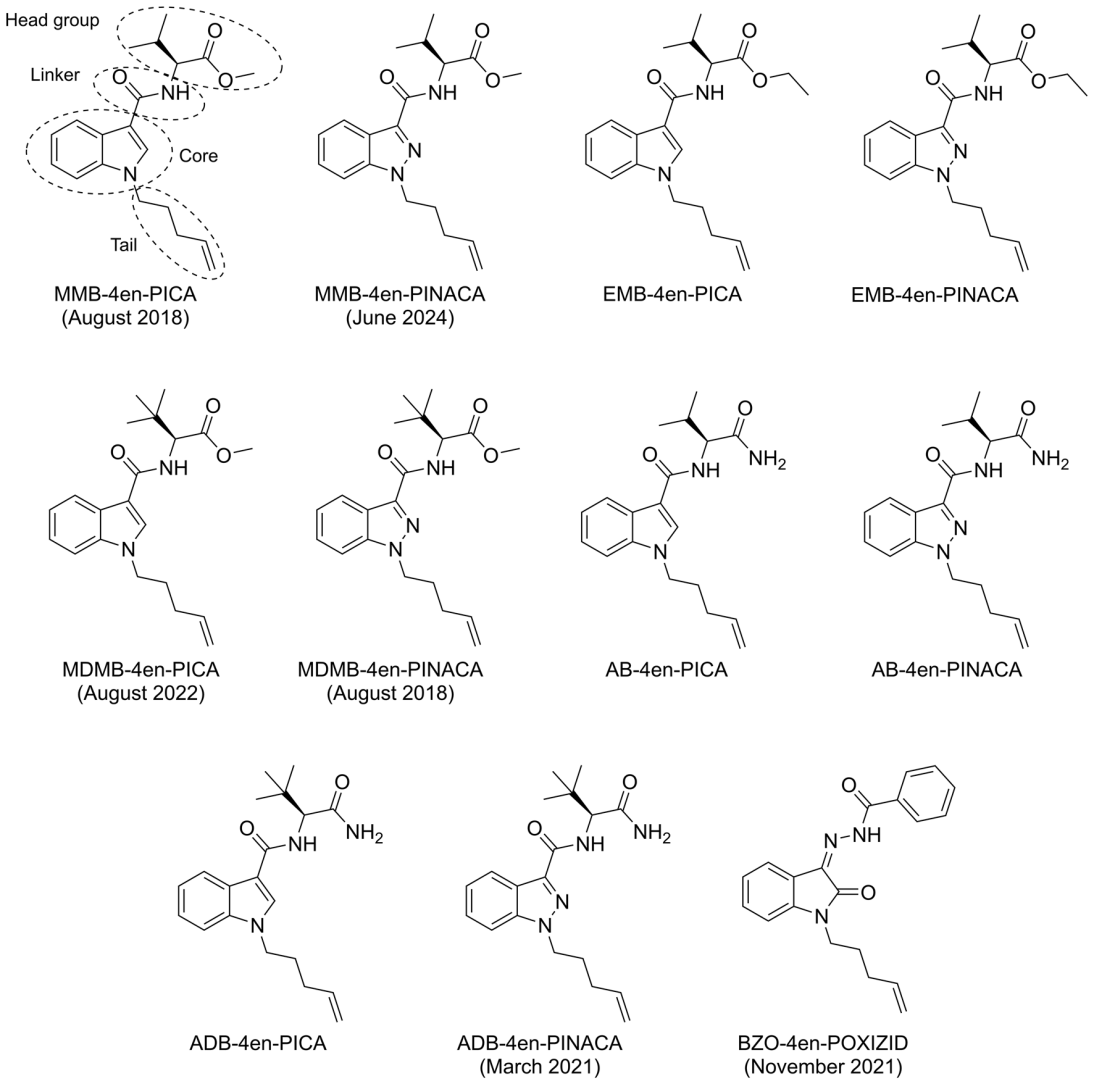
Structures of the amino acid-derived and OXIZID 4-pentenyl SCRAs investigated in this study (with the date that a detection on the illicit drug market was first reported, where applicable)

In 2018, amino acid-derived 4-pentenyl SCRAs, containing a pentyl tail with an alkene in the 4-position (Fig. 1), were detected on the illicit drug market in Europe for the first time. MDMB-4en-PINACA was notified by the EMCDDA in August 2018 after identification in a test purchase (EMCDDA 2021), while the first detections in the USA were reported in September 2019 from analysis of toxicological samples (NPS Discovery 2019). MDMB-4en-PINACA subsequently increased in prevalence in 2019, becoming one of the most detected SCRAs worldwide by 2020 (Norman et al. 2021). It has remained the most prevalent SCRA in the USA, with 1800 reports of MDMB-4en-PINACA recorded by the National Forensic Laboratory Information System (NFLIS) in 2022, accounting for 33% of all SCRA detections (NFLIS 2022). Despite the rise in prevalence of *tert*-leucinamide SCRAs ADB-BUTINACA and ADB-HEXINACA in Scottish prisons from 2021 to 2022 (Kronstrand et al. 2022), the number of MDMB-4en-PINACA detections have once again increased considerably in the UK since 2022 (UK Home Office 2022b). After the introduction of MDMB-4en-PINACA, its structural analogues, MMB-4en-PICA (AMB-4en-PICA, MMB022) and ADB-4en-PINACA (Fig. 1), were identified on the illicit market in 2018 and 2021, respectively (Kronstrand et al. 2022; Watanabe et al. 2020b), though these appear to have been considerably less prevalent. A number of detections of MDMB-4en-PICA were reported in August 2022 (UK Home Office 2022a) from detections in UK prisons between April 2020 and March 2021, while MMB-4en-PINACA was recently detected for the first time in February 2024 in the USA and reported in June 2024 (NPS Discovery 2024). The introduction of wide-ranging SCRA analogue controls by China in 2021, which included amino acid-derived SCRAs, has resulted in significant structural diversification. One new class to emerge was the oxindole hydrazide (OXIZID) SCRAs, including the 4-pentenyl analogue BZO-4en-POXIZID, which was formally notified by the EMCDDA as an NPS in November 2021 (EMCDDA 2022). Furthermore, MDMB-4en-PINACA has been co-detected with the SCRA precursor MDMB-INACA in seized samples in the USA since 2023 (NPS Discovery 2023), suggesting a shift towards in situ production of SCRAs such as the 4-pentenyl compounds, following the generic controls introduced by China.

SCRAs tend to be extensively and rapidly metabolised once consumed (Brandon et al. 2021), and their potency (Antonides et al. 2021; Cannaert et al. 2020; Grafinger et al. 2021) and lipophilicity (Brandon et al. 2021; Brandon et al. 2023; Kakehashi et al. 2020) can result in low concentrations of parent compounds in some biological matrices, particularly in urine samples, making metabolites essential biomarkers of SCRA consumption in clinical and forensic toxicology casework. In vitro incubations of SCRAs with human liver microsomes (HLMs) or human hepatocytes (HHeps) have proved an effective tool in identifying key metabolites for this purpose (Diao and Huestis 2019). The metabolic profiles of MDMB-4en-PINACA (Gu et al. 2022; Ozturk and Yeter 2020; Watanabe et al. 2020a), MMB-4en-PICA (Watanabe et al. 2020b) and ADB-4en-PINACA (Kronstrand et al. 2022) have previously been elucidated using this approach, with terminal group (ester/amide) hydrolysis, dihydrodiol formation on the tail and hydroxylation identified as the most prevalent metabolic pathways. Dihydrodiol formation and hydroxylation were also the key metabolic pathways for BZO-4en-POXIZID (Watanabe et al. 2023). For MDMB-4en-PINACA, the major metabolites found in vitro have been detected in authentic blood and/or urine samples (Gu et al. 2022; Ozturk and Yeter 2020; Watanabe et al. 2020a). The in vitro metabolism of ADB-4en-PINACA (Kronstrand et al. 2022) also appears to match in vivo metabolism (Fong and Moy 2024). Despite these studies, the metabolic profiles of MDMB-4en-PICA, MMB-4en-PINACA and several envisioned 4-pentenyl structural analogues (EMB-4en-PICA, EMB-4en-PINACA, AB-4en-PICA, AB-4en-PINACA and ADB-4en-PICA, Fig. 1) are yet to be elucidated.

Producers frequently adapt the structures of SCRAs to evade newly introduced legislation designed to control these compounds, particularly that of producer countries. This creates a time-lag from when a compound enters the market, is first detected, its harms established, and its metabolites identified to develop appropriate assays for detection in biological matrices. Systematic assessment of the pharmacodynamics and metabolism of a whole series of SCRAs can, therefore, yield useful information on relative risk and metabolite formation respectively, before compounds even appear on the illicit drug market. While structure–activity relationships (SARs) of 4-pentenyl SCRAs have been explored, revealing the greater potency of methyl *tert*-leucinate (MDMB), *tert*-leucinamide (ADB) and indazole carboxamide (INACA) SCRAs compared to methyl valinate (MMB), valinamide (AB) and indole carboxamide (ICA) compounds (Grafinger et al. 2021), structure–metabolism relationships (SMRs) have not yet been fully identified. SMRs of earlier emerging SCRAs, which included these head groups and cores with a range of tails, have been investigated using HLM incubations, highlighting how structure affects terminal ester or amide hydrolysis, secondary amide hydrolysis, *N*-dealkylation, dehydrogenation, dehalogenation on the tail, dihydrodiol formation on the core and hydroxylation (Franz et al. 2019). However, this work was carried out before the emergence of 4-pentenyl SCRAs and no ethyl ester (e.g., ethyl valinate, EMB) analogues were assessed in the study. Furthermore, HHep incubations tend to better represent in vivo metabolism than those of HLMs, as HHeps contain all of the phase I and phase II liver enzymes, co-factors, drug transporters and drug-binding proteins required for metabolism, and possess the cell membranes which the drug must first penetrate, as occurs in vivo (Diao and Huestis 2019). Comparisons of several studies on *tert*-leucinamide SCRA metabolism using HHeps have also previously revealed that greater levels of metabolism occur on longer (hexyl/pentyl) SCRA tails (Baginski et al. 2023; Carlier et al. 2017) compared to those with a shorter (butyl) chain (Kronstrand et al. 2022), for which biotransformation at the core is favoured. These findings were also replicated in vivo (Kronstrand et al. 2022; Giorgetti et al. 2024).

In the present study, HHep incubations and liquid chromatography–quadrupole time-of-fight mass spectrometry (LC–QTOF-MS) were used to assess the impact of the presence of an alkene group in the 4-position of the pentyl tail of a SCRA on metabolism. This was achieved by elucidating SMRs for a series of amino acid-derived compounds (MMB-4en-PICA, MMB-4en-PINACA, EMB-4en-PICA, EMB-4en-PINACA, MDMB-4en-PICA, MDMB-4en-PINACA, AB-4en-PICA, AB-4en-PINACA, ADB-4en-PICA, ADB-4en-PINACA) and the OXIZID SCRA BZO-4en-POXIZID (Fig. 1). The HHep incubation data for ADB-4en-PINACA and BZO-4en-POXIZID used here has been published previously (Kronstrand et al. 2022; Watanabe et al. 2023), but has not been reported and discussed in the context of SMRs.

## Materials and methods

### Materials

Cryopreserved primary HHeps (LiverPool, 20 donor pool, lot: BEK, characterised for phase I and II metabolic pathways) and InVitro Gro HT thawing medium were purchased from Bioreclamation IVT (Brussels, Belgium). Liquid chromatography materials (LC–MS grade acetonitrile and formic acid) and cell culture media (Williams E medium, L-glutamine and HEPES buffer) were obtained from Thermo Fisher Scientific (Gothenburg, Sweden). Ultra-pure water was obtained using a Milli-Q water purification system (Millipore, Billerica, MA, USA). LC–MS grade methanol was obtained from Merck (Darmstadt, Germany) and ethanol was from Kemetyl AB (Jordbro, Sweden). Dihydrodiol tail metabolite reference standards were synthesised for the amino acid 4-pentenyl SCRAs, to confirm that dihydrodiol formation in the incubations occurred on the SCRA tail rather than the indole or indazole core. All amino acid-derived SCRAs and dihydrodiol metabolite reference standards were synthesised in-house at Linköping University based on previously described methods (Watanabe et al. 2020b) and were the (*S*)-enantiomers; previous studies have shown that these parent SCRAs are much more prevalent and potent than the corresponding (*R*)-enantiomers (Antonides et al. 2021). No major impurities were identified in the ^1^H NMR spectra of these synthesised reference standards (see online supplementary information), indicating net purities of > 95%. BZO-4en-POXIZID (96.7% net purity), was purchased from Chiron AS (Trondheim, Norway). Stock solutions of SCRAs were prepared in methanol or ethanol and diluted on the day of the experiment in Williams E medium.

### Hepatocyte incubations

Incubations of the SCRAs with HHeps and subsequent metabolite identification were carried out as previously described in a published procedure, with slight modifications (Åstrand et al. 2019; Stalberga et al. 2022). To summarise, 5 µM of each SCRA was incubated with HHeps (100,000 cells) at 37 °C, 5% CO_2_ in a total volume of 100 μL of Williams E medium supplemented with L-glutamine and HEPES. Incubations were carried out in duplicate and quenched by the addition of 100 µL ice-cold acetonitrile at 0, 0.5, 1 and 3 h, after which the incubates were centrifuged at 1,100 × *g* for 15 min at 4 °C. The resulting supernatant was analysed by LC–QTOF-MS. On each day, negative controls (HHeps without drug) and degradation controls (drug without HHeps) were also incubated for 3 h.

### LC–QTOF-MS analysis

Chromatographic separation was achieved using an Agilent 1290 Infinity ultra-high performance liquid chromatography (UHPLC) system (Agilent Technologies, Kista, Sweden); 4 µL supernatant was injected onto an Acquity HSS T3 column (150 × 2.1 mm, 1.8 μm) fitted with an Acquity VanGuard precolumn (Waters, Sollentuna, Sweden). Gradient elution was employed using 0.1% formic acid in water (A) and 0.1% formic acid in acetonitrile (B) mobile phases: 1% B (0–0.6 min); 1–20% B (0.6–0.7 min); 20–85% B (0.7–13 min); 85–95% B (13–15 min); 95% B (15–18 min); 95–1% B (18–18.1 min); 1% B (18.1–19 min), with a flow rate of 0.5 mL/min and column temperature of 60 °C. The UHPLC system was coupled to an Agilent 6550 iFunnel QTOF mass spectrometer (Agilent Technologies, Kista, Sweden) with a Dual Agilent Jet Stream electrospray ionisation (ESI) source. Data were acquired in positive ESI mode using auto MS/MS acquisition: scan range, 100–950 m*/z* (MS) and 50–950 m*/z* (MS/MS); precursor intensity threshold, 5,000 counts; precursor number per cycle, 5; fragmentor voltage, 380 V; collision energy (CE), 3 eV at 0 m*/z* ramped up by 8 eV per 100 m*/z*; gas temperature, 150 °C; gas flow, 18 L/min; nebulizer gas pressure, 345 kPa; sheath gas temperature, 375 °C; and sheath gas flow, 11 L/min.

### Metabolite identification and assessment of structure–metabolism relationships

Agilent MassHunter Qualitative Analysis software (version B.07.00) was used for LC–QTOF-MS data analysis as described previously (Åstrand et al. 2019; Stalberga et al. 2022), with the following parameters used to determine peak areas: mass error, 10 ppm; absolute peak area threshold, 20,000 counts; maximum number of matches, 20; and chromatogram extraction window, 50 ppm. For a metabolite to be reported, the following criteria were set: mass error < 5 ppm for [M + H]^+^ (except for saturated peaks where the mass accuracy could deviate), consistent isotopic pattern, product ion spectrum consistent with the proposed structure, retention time (RT) between 2 and 13 min and plausible for the proposed structure, and absence of the peaks in the 0 h samples and negative and degradation controls. For compounds that were identified in at least one sample timepoint but present at low concentrations (an approximate peak area threshold of 20,000 counts) in other samples, a manual assessment was carried out (using automatic or manual integration with a peak area threshold of 5000 counts) to decide if the compound should be reported as a metabolite.

To identify structure–metabolism relationships, the total proportion (%) that each type of biotransformation contributed to the summed abundance of metabolites (as measured by peak area) across the 0.5, 1 and 3 h incubations was determined. For metabolites arising from a combination of metabolic pathways (e.g., terminal group hydrolysis + hydroxylation), the peak areas were divided by the number of metabolic pathways (e.g., 2) and assigned to each individual biotransformation type.

## Results and discussion

### Structure–metabolism relationships

**Fig. 2:**
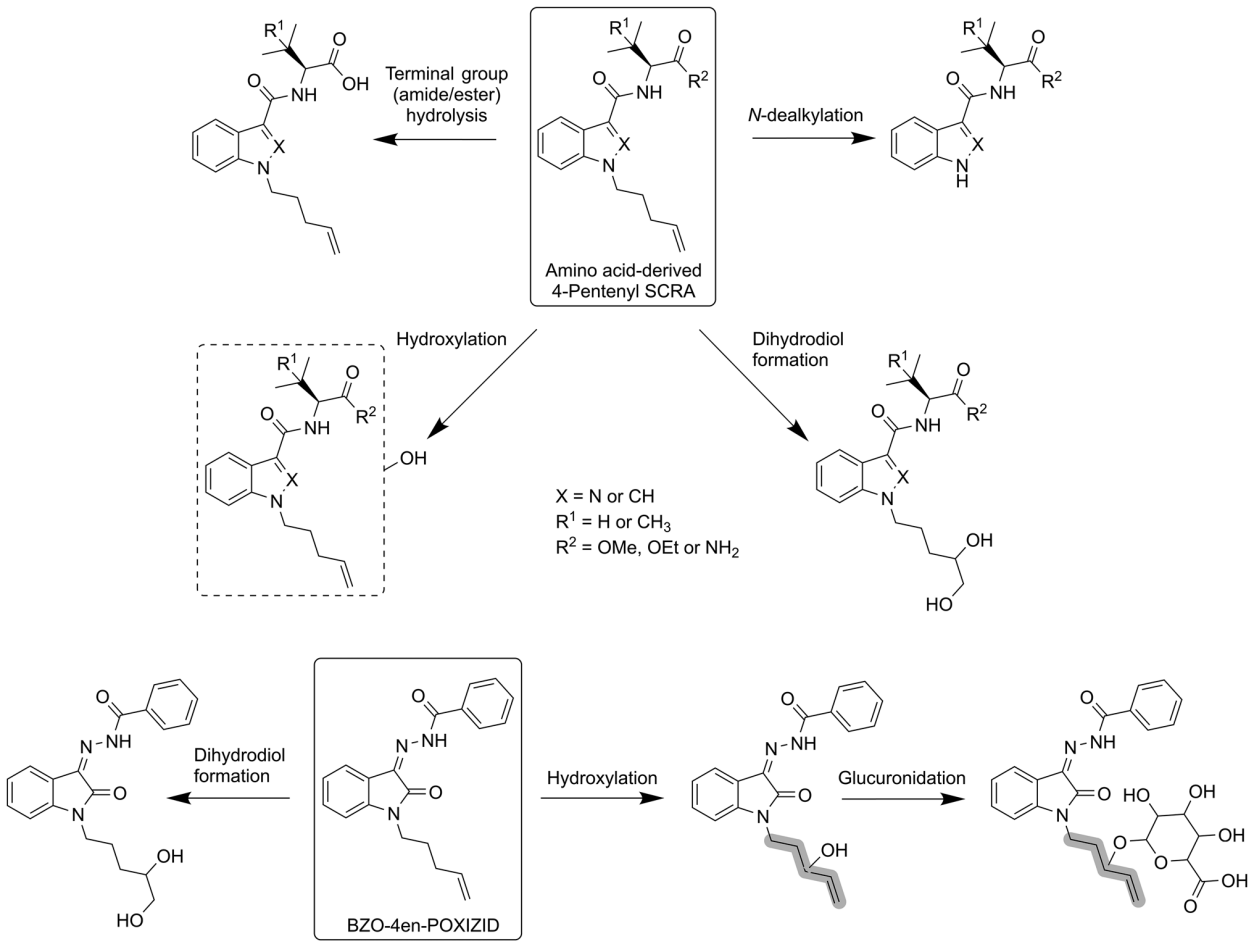
The major metabolic pathways identified for the 4-pentenyl SCRAs

**Fig. 3:**
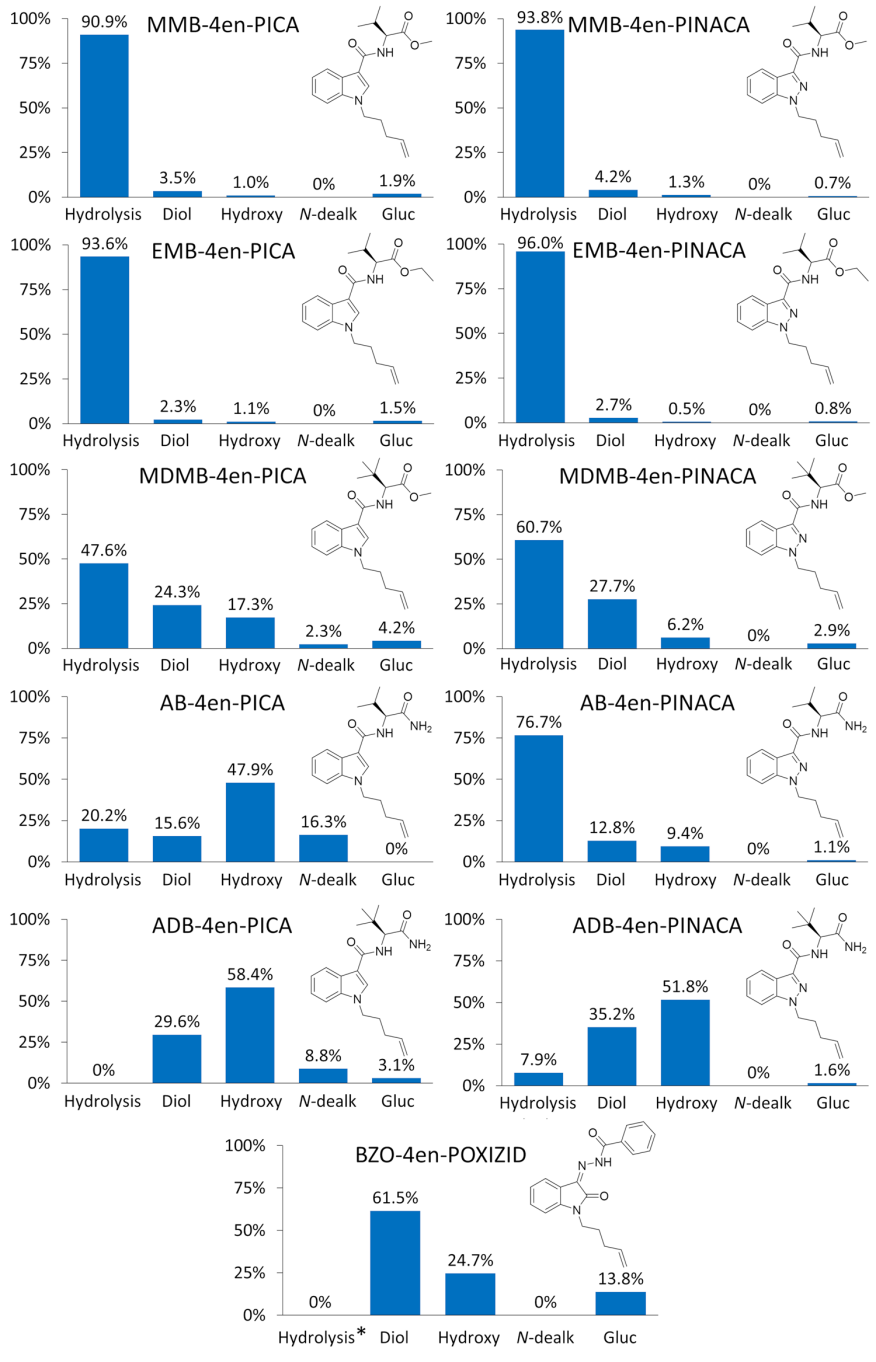
The proportion that terminal amide/ester hydrolysis (Hydrolysis), dihydrodiol formation (Diol), hydroxylation (Hydroxy), *N*-dealkylation (*N*-dealk) and glucuronidation (Gluc) contributed to the overall metabolism of the 4-pentenyl SCRAs. *No terminal ester or amide group present in the molecule

Four major phase I metabolic pathways were identified for the 4-pentenyl analogues included in this study: (i) terminal group (ester/amide) hydrolysis; (ii) dihydrodiol formation on the tail; (iii) hydroxylation anywhere on the molecule (but predominantly the tail); and (iv) *N*-dealkylation (Fig. 2). To a much lesser extent, ketone formation, dehydrogenation, secondary amide hydrolysis and head group–linker cleavage were also observed for some SCRAs. The only phase II metabolic pathway identified for the 4-pentenyl SCRAs was glucuronidation, but this was far less prominent than the major phase I routes. The extent to which each major metabolic pathway contributed to the overall metabolism of the SCRAs is summarised in Fig. 3. Analytical data (chromatograms and mass spectra), tables listing the specific metabolites formed and figures depicting proposed metabolic pathways for each SCRA can be found in the online supplementary information.

The results agreed well with a previous SMR study (Franz et al. 2019), which covered a similar range of compounds but did not include 4-pentenyl analogues and used HLMs rather than HHeps. Ester hydrolysis was the predominant metabolic pathway for methyl valinate (MMB) SCRAs (90.9–93.8% contribution to compound metabolism, Fig. 3). The addition of an extra methyl moiety in the ethyl ester groups of the ethyl valinate (EMB) SCRAs had little effect on the proportion of ester hydrolysis observed (93.6–96.0%) compared to the methyl ester analogues. This trend has previously been observed both in vitro and in vivo for the methyl *tert*-leucinate (MDMB) SCRA with a 5-fluoro-pentyl tail, 5F-MDMB-PICA (Truver et al. 2020), and its ethyl ester analogue, 5F-EDMB-PICA (Giorgetti et al. 2023). In the present study, ester hydrolysis remained the major type of biotransformation for the MDMB 4-pentenyl SCRAs (47.6–60.7%, Fig. 3), although this was a less dominant reaction than observed for the MMB and EMB SCRAs. This is likely due to the bulky *tert*-butyl side chain of the MDMB head group increasing steric hindrance and thus reducing interaction of the molecules with the metabolising carboxylesterases, compared to the less bulky *iso*-propyl side chain of the MMB and EMB SCRAs. Ester hydrolysis was more prominent for MDMB-4en-PINACA (60.7%) than for its indole analogue, MDMB-4en-PICA (47.6%), as previously observed in structurally related SCRAs without a 4-pentenyl tail (Franz et al. 2019). This may be due to the additional nitrogen in the indazole core enabling stronger interactions with metabolising enzymes and stabilising the preferred conformation for enzyme–substrate formation through intramolecular interactions with the carboxamide linker (Franz et al. 2019; Krishna Kumar et al. 2019). Terminal alkene tails have been demonstrated to be reactive moieties in SCRA molecules, with dihydrodiol formation occurring via cytochrome P450-catalysed epoxidation followed by epoxide hydrolase-catalysed or even spontaneous hydrolysis of the unstable epoxide intermediate (Watanabe et al. 2020a, 2020b). The lower contribution of ester hydrolysis to MDMB SCRA metabolism resulted in higher levels of dihydrodiol formation on the tail (24.3–27.7%), but also, more hydroxylation (6.2–17.3%) as summarised in Fig. 3. Dihydrodiol formation on the indazole or indole core was not observed for any SCRA included in the study, as confirmed with synthesised dihydrodiol tail reference standards and LC–QTOF-MS analysis (supplementary information). Dihydrodiol formation on the core of a SCRA, where observed, is typically only a very minor metabolic pathway (Franz et al. 2019). The substantial contribution of this biotransformation previously reported for ADB-BUTINACA metabolism (Kronstrand et al. 2022) likely arose due to the terminal amide moiety present in the head group and the short butyl tail decreasing metabolism at these regions of the molecule. In the present study, the lower reactivity of the aromatic ring of the indazole/indole cores to epoxidation, arising from electron delocalisation, explains why biotransformation of the alkene in the tail to a dihydrodiol was much more favourable.

The greater stability of amides to hydrolysis compared to esters (Franz et al. 2019) unsurprisingly resulted in a lower proportion of terminal group hydrolysis for valinamide (AB) 4-pentenyl SCRAs (20.2–76.7%) compared to the corresponding MMB SCRAs (90.9–93.8%). *tert*-Leucinamide (ADB) 4-pentenyl SCRAs, having the combination of both the bulky *tert*-butyl side chain *and* the terminal amide group, showed the greatest stability of all SCRAs in the study to terminal group hydrolysis (0–7.9%, Fig. 3). The increased activation of indazole compounds towards terminal group hydrolysis compared to indole analogues was more pronounced in the terminal amide-containing 4-pentenyl SCRAs than for the ester containing MDMB 4-pentenyl SCRAs, with terminal amide hydrolysis accounting for 20.2% of AB-4en-PICA metabolism compared to 76.7% of AB-4en-PINACA metabolism and 0% of ADB-4en-PICA metabolism versus 7.9% of ADB-4en-PINACA metabolism (Fig. 3). While terminal amide hydrolysis was the most prevalent biotransformation for AB-4en-PINACA, hydroxylation was instead the major metabolic pathway for AB-4en-PICA and the ADB 4-pentenyl SCRAs (47.9–58.4%). This greater hydrolytic stability resulted in more pronounced dihydrodiol formation on the tail of the ADB 4-pentenyl SCRAs (29.6–35.2%) compared to the AB SCRAs (12.8–15.6%). As shown in Fig. 3, *N*-dealkylation was also a key metabolic pathway for 4-pentenyl SCRAs with indole cores e.g., ADB-4en-PICA (8.8%) and AB-4en-PICA (16.3%), and a minor metabolic pathway for MDMB-4en-PICA (2.3%), but was not detected in any of the indazole 4-pentenyl SCRA analogue incubates. *N*-dealkylation would proceed via hydroxylation of the α-carbon atom of the pentenyl tail, followed by elimination of the tail as an aldehyde. The negative mesomeric effect (− M) between the indole nitrogen and the oxygen in the carboxamide linker, which encourages this elimination, would be disrupted by the additional nitrogen atom in the indazole analogues, hindering dealkylation (Franz et al. 2019).

BZO-4en-POXIZID has a very different structure to the amino acid-derived 4-pentenyl SCRAs included in this study, lacking a terminal amide or ester moiety. As a result, dihydrodiol formation on the 4-pentenyl tail (61.5%) was the most prevalent of all compounds studied (Fig. 3); this was also the predominant metabolic pathway of BZO-4en-POXIZID in HLM incubations (Watanabe et al. 2023). However, the abundances of metabolites were small relative to the parent compound, suggesting much slower metabolism than the earlier emerging SCRAs studied to date. The highest level of glucuronidation was also seen for this OXIZID 4-pentenyl SCRA (13.8%), which was a less prominent biotransformation for the amino acid-derived compounds (0–4.2%). This is an important finding as hydrolysis of glucuronides can significantly increase the abundance of phase I metabolites to improve their detection.

Knowledge of SMRs supports the prediction of the metabolites of emerging and future SCRAs. This is invaluable to clinical and forensic toxicology laboratories, facilitating the preliminary identification of SCRA consumption in screening procedures before metabolite reference standards are available. In turn, this supports the monitoring of their prevalence in toxicology casework at the earliest opportunity and retrospective data mining of previously collected non-targeted high-resolution mass spectrometry (HRMS) data. This work also supports reference standard producers in ensuring the timely availability of SCRA metabolite standards.

### Suggested biomarkers of 4-pentenyl SCRA consumption

Elucidation of SMRs aids the selection of particular metabolites as recommended biomarkers of parent SCRA consumption and the data derived from this study are summarised in Table 1. Extensive dihydrodiol formation on the tail for BZO-4en-POXIZID resulted in the dihydrodiol product (K1) as the most abundant metabolite, which is therefore recommended as a BZO-4en-POXIZID biomarker. For MMB-4en-PICA, MMB-4en-PINACA, EMB-4en-PICA, EMB-4en-PINACA and AB-4en-PINACA, terminal group hydrolysis was such a dominant biotransformation that the ester/amide hydrolysis products alone (A1, B1, C1, D1 and H1) are recommended as analytical targets. In a previous study of MMB-4en-PICA metabolism using HLMs, ester hydrolysis was also the major biotransformation observed, and the dihydrodiol and combined ester hydrolysis + dihydrodiol metabolites were ranked second and fourth in abundance, respectively (Watanabe et al. 2020b). In the present study using HHeps, the dihydrodiol (A7) was the least abundant metabolite identified after 3 h, while the combined ester hydrolysis + dihydrodiol product (A2) was ranked second (Supplementary Table S1), highlighting the differences that can be seen between the two in vitro methods.

**Table 1:** Recommended biomarkers of 4-pentenyl SCRA consumption

Parent 4-Pentenyl SCRA	Recommended Metabolite Biomarker(s)
MMB-4en-PICA, MMB-4en-PINACA, EMB-4en-PICA, EMB-4en-PINACA and AB-4en-PINACA	Terminal group (ester/amide) hydrolysis metabolite
MDMB-4en-PICA	Ester hydrolysis metabolite, dihydrodiol tail metabolite, ester hydrolysis + dihydrodiol tail metabolite and hydroxylated metabolites
MDMB-4en-PINACA	Ester hydrolysis metabolite, ester hydrolysis + dihydrodiol tail metabolite and hydroxylated metabolites
AB-4en-PICA, ADB-4en-PICA and ADB-4en-PINACA	Mono-hydroxylated tail metabolites and dihydrodiol tail metabolite
BZO-4en-POXIZID	Dihydrodiol tail metabolite

Reduced ester hydrolysis for methyl *tert*-leucinate (MDMB) compounds compared to methyl valinate (MMB) SCRAs means that although the ester hydrolysis metabolite is most abundant for both MDMB-4en-PICA and MDMB-4en-PINACA (E1 and F1, respectively), the dihydrodiol (E2) and ester hydrolysis + dihydrodiol (E3) products of MDMB-4en-PICA, and the ester hydrolysis + dihydrodiol metabolite (F3) of MDMB-4en-PINACA are also abundant enough to be recommended as suitable biomarkers of consumption of these SCRAs. Watanabe et al*.* found F3 to actually be a more abundant MDMB-4en-PINACA metabolite than F1 in both the 5 h HHep incubations and a single urine sample, though the ester hydrolysis product was the only metabolite detected in blood (Watanabe et al. 2020a). This difference between the HHep studies is due to the shorter incubation time of 3 h used in the present study. The ester hydrolysis and/or combined ester hydrolysis + dihydrodiol metabolites of MDMB-4en-PINACA have been detected in authentic blood matrices and urine samples in several other studies too, sometimes along with the parent compound (Goncalves et al. 2022; Gu et al. 2022; Kleis et al. 2021; Krotulski et al. 2021; Ozturk and Yeter 2020; Wu et al. 2023).

One problem clearly arising from these closely related SCRAs is that they can produce common metabolites, hindering identification of the particular SCRA consumed, which may have different risk profiles depending on their pharmacological activity and pharmacokinetics. The ester hydrolysis and combined ester hydrolysis + dihydrodiol products of MDMB-4en-PINACA, for example, are also produced by ADB-4en-PINACA (Fong and Moy 2024; Kronstrand et al. 2022). Mono-hydroxylated products have previously been suggested as more specific biomarkers of MDMB-4en-PINACA intake as the terminal ester group and 4-pentenyl tail are left intact, enabling unequivocal identification of MDMB-4en-PINACA consumption (Gu et al. 2022; Ozturk and Yeter 2020); the high levels of hydroxylation seen for MDMB-4en-PICA, AB, and ADB SCRAs in our study (9.4–58.4%) supports this suggested strategy for these compounds too. Indeed, as hydroxylation was the major metabolic pathway for AB-4en-PICA, ADB-4en-PICA and ADB-4en-PINACA, mono-hydroxylated tail metabolites (G1, I1 and J1) are some of the most abundant metabolites for these compounds. As shown in Table 1, they are therefore recommended as analytical targets to identify consumption of the parent SCRAs, along with the dihydrodiol products (G4, I2 and J2). The mono-hydroxylated tail metabolite (J1) and dihydrodiol tail metabolite (J2) of ADB-4en-PINACA were also some of its most abundant metabolites in vivo, following analysis of two authentic urine samples from routine forensic toxicology casework (Fong and Moy 2024). Knowledge of the formation of common metabolites can potentially be exploited by toxicologists, by using them as targets in initial screening procedures to detect a range of SCRAs, before specific identification of the SCRA in question for confirmatory analysis using a unique metabolite. In addition, MDMB-4en-PINACA was sometimes detected in both blood and urine samples in previous studies, so parent SCRAs may also be used as suitable additional analytical targets.

### Study limitations

Although glucuronidation may appear to be a much less dominant metabolic pathway compared to the phase I reactions, a number of glucuronides were detected for each SCRA. The poor ionisation of glucuronides and in-source fragmentation in ESI mass spectrometry can result in significant signal reduction, which may have made this type of biotransformation appear less prominent than is actually the case. A longer incubation time may have also resulted in enhanced glucuronidation, as this metabolic pathway typically occurs *after* phase I biotransformations and was more prominent in studies of in vivo MDMB-4en-PINACA metabolism (Wu et al. 2023). Enzymatic hydrolysis is therefore still likely to be an important step in the analysis of 4-pentenyl SCRA metabolites in urine. Indeed, many of the phase I metabolites recommended as biomarkers of SCRA intake in Table 1 were also detected as glucuronides, so would be even more abundant after deglucuronidation. Different ionisation efficiencies could have also led to signal reduction or enhancement for the other metabolites, so their abundances may not reflect true metabolite concentrations and hence the contribution of a particular metabolic pathway. However, LC–QTOF-MS is commonly used within both clinical and forensic toxicology settings, especially for screening of samples. The metabolites identified in this study are, therefore, still likely to be the most suitable analytical targets for detection in practice.

As this work aimed to find SMRs for potential future SCRAs that are analogues of established compounds, most of the metabolites in this study have not yet been confirmed in authentic clinical and forensic samples. Although the liver is the major site of drug metabolism, SCRA metabolism also takes place in other organs and tissues such as the lungs and blood, which may contribute to metabolism differently. The complexity of in vivo metabolism can therefore never be fully represented using in vitro techniques. Nonetheless, metabolism of MDMB-4en-PINACA is well investigated and in vivo ADB-4en-PINACA metabolism has also been reported. The in vitro data presented in this study correlates well with published in vivo data, giving additional confidence in the SMRs elucidated and the biomarkers recommended.

## Conclusions

Terminal group (ester/amide) hydrolysis, dihydrodiol formation on the tail, hydroxylation and *N*-dealkylation have all been identified as the major phase I metabolic pathways for 4-pentenyl SCRAs. Elucidation of SMRs has allowed clear patterns to be identified within the 4-pentenyl SCRAs included in this study: terminal group hydrolysis is favoured for SCRAs containing esters and/or *iso*-propyl groups (MMB, EMB and AB-4en-PINACA), while those with terminal amides and/or *tert*-butyl side chains are more resistant to hydrolysis and display greater levels of dihydrodiol formation on the tail and hydroxylation (MDMB-4en-PICA, MDMB-4en-PINACA, AB-4en-PICA, ADB-4en-PICA and ADB-4en-PINACA). As observed for the metabolism of BZO-4en-POXIZID, the absence of terminal ester/amide head groups and replacement with more metabolically stable moieties results in enhanced dihydrodiol formation on the tail moiety. SCRAs display a clear difference in metabolism based on their structure. SMRs can be used to suggest biomarkers of consumption of SCRAs and can be applied to future SCRAs to predict their metabolites before confirmatory studies are available.

## Supplementary Information

Below is the link to the electronic supplementary material.

Supplementary file1 (PDF 11673 KB)

Supplementary file2 (PDF 1333 KB)

Supplementary file3 (PDF 338 KB)

Supplementary file4 (PDF 4726 KB)

## Data Availability

All data generated and analysed for this study are included in this published article and the online supplementary information.
