# Supplementary material for: Structure–metabolism relationships of 4-pentenyl synthetic cannabinoid receptor agonists using in vitro human hepatocyte incubations and high-resolution mass spectrometry
Source: Arch Toxicol. 2025 May 20;99(8):3331–41. doi: 10.1007/s00204-025-04080-6 (PMC12367891; doi:10.1007/s00204-025-04080-6)
Supplement: Supplementary file 1 — Supplementary file1 (PDF 11673 KB) [file 204_2025_4080_MOESM1_ESM.pdf]

Structure–metabolism relationships of 4-pentenyl synthetic cannabinoid receptor agonists using *in vitro* human hepatocyte incubations and high-resolution mass spectrometry

Supplementary Information for Archives of Toxicology – Chromatograms and Mass Spectra

Steven R Baginski[1,*], Karin Lindbom[2], Bryan Valencia Crespo[3], Ghidaa Bessa[3], Tobias Rautio[3], Xiongyu Wu[3], Johan Dahlén[3], Lorna A Nisbet[1], Craig McKenzie[1,4], Henrik Gréen[2,5,*]

[1] Leverhulme Research Centre for Forensic Science, School of Science and Engineering, University of Dundee, Dundee, UK

[2] Division of Clinical Chemistry and Pharmacology, Department of Biomedical and Clinical Sciences, Linköping University, Linköping, Sweden

[3] Department of Physics, Chemistry and Biology, Linköping University, Linköping, Sweden

[4] Chiron AS, Trondheim, Norway

[5] Department of Forensic Genetics and Forensic Toxicology, National Board of Forensic Medicine, Linköping, Sweden

*Corresponding authors: Steven Baginski, sbaginski001@dundee.ac.uk and Henrik Gréen, henrik.green@liu.se

MMB-4en-PICA

Metabolism

MMB-4en-PICA, RT 11.07 min, *m/z* 343.2038

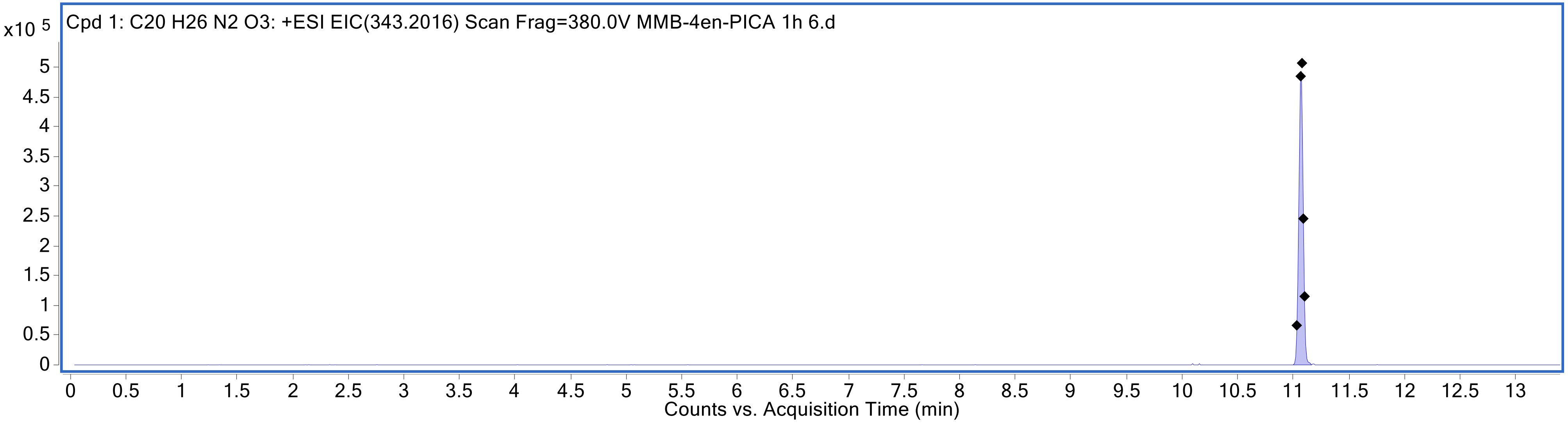

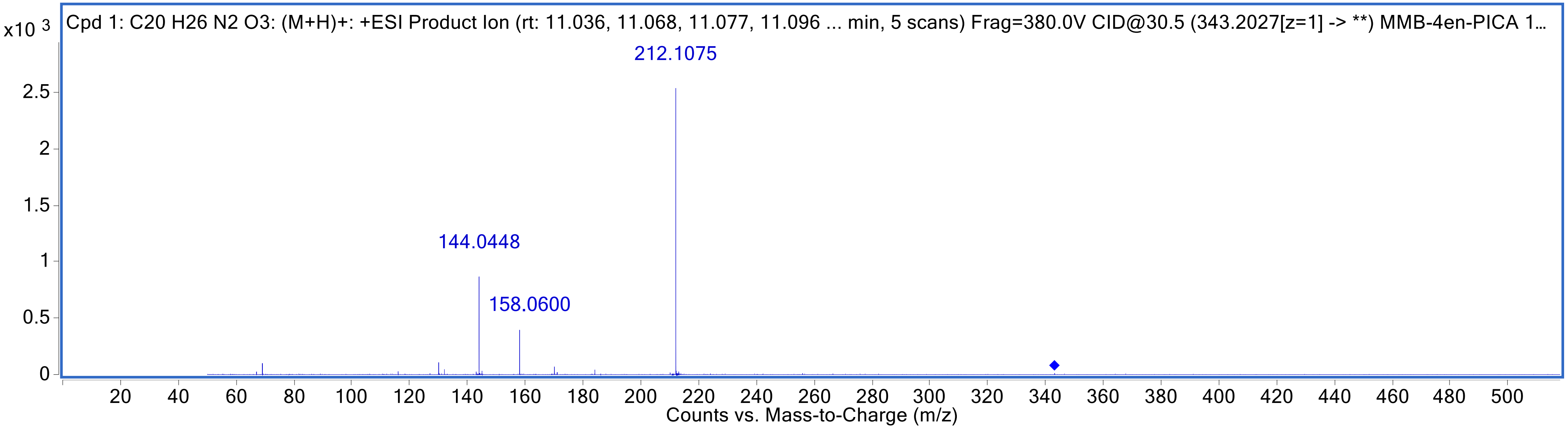

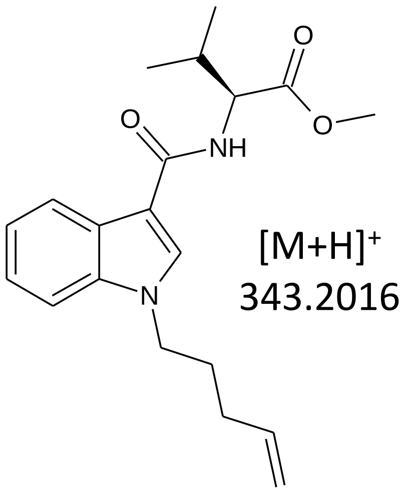

$[M+H]^+$
343.2016

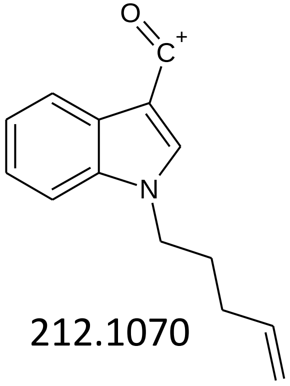

212.1070

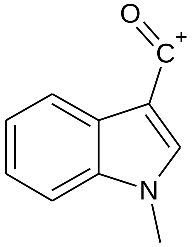

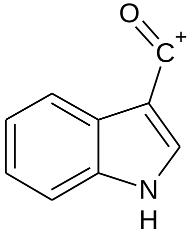

158.0600 144.0444

A1, Ester hydrolysis, RT 9.15 min, *m/z* 329.1879

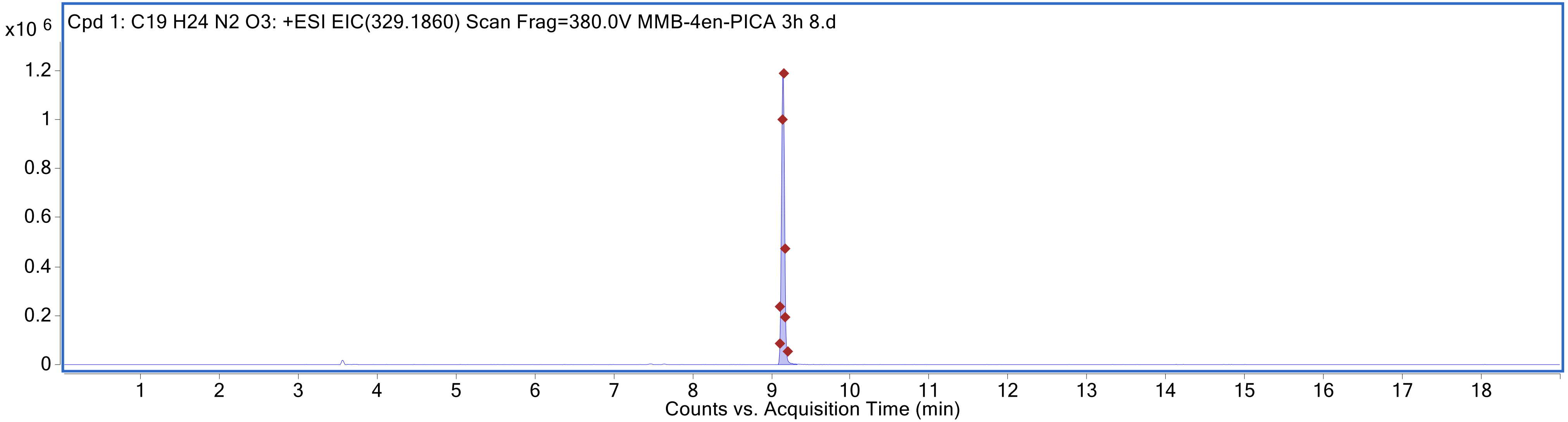

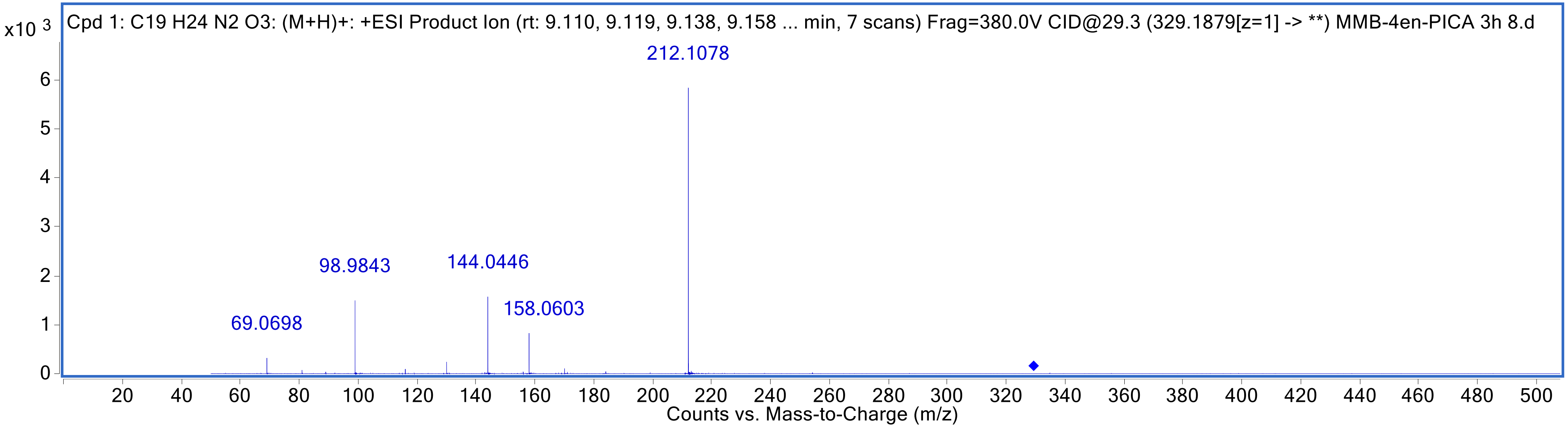

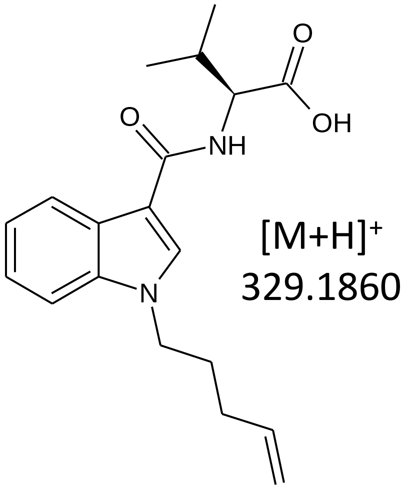

$[M+H]^+$
329.1860

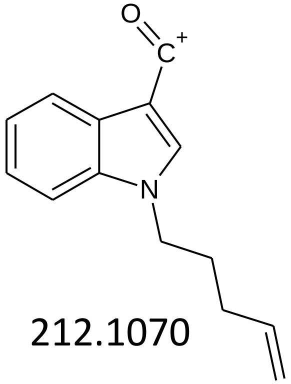

212.1070

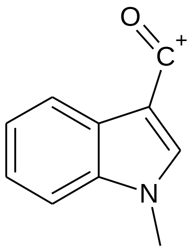

158.0600

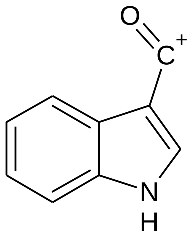

144.0444

A2, Ester hydrolysis + dihydrodiol formation, RT 4.66 min, *m/z* 363.1919

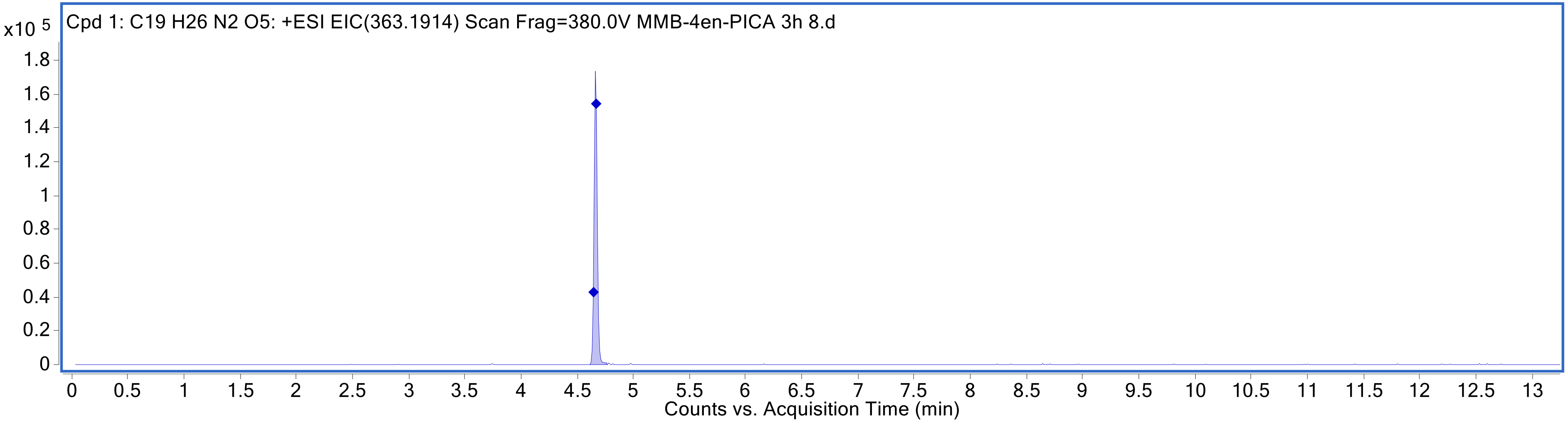

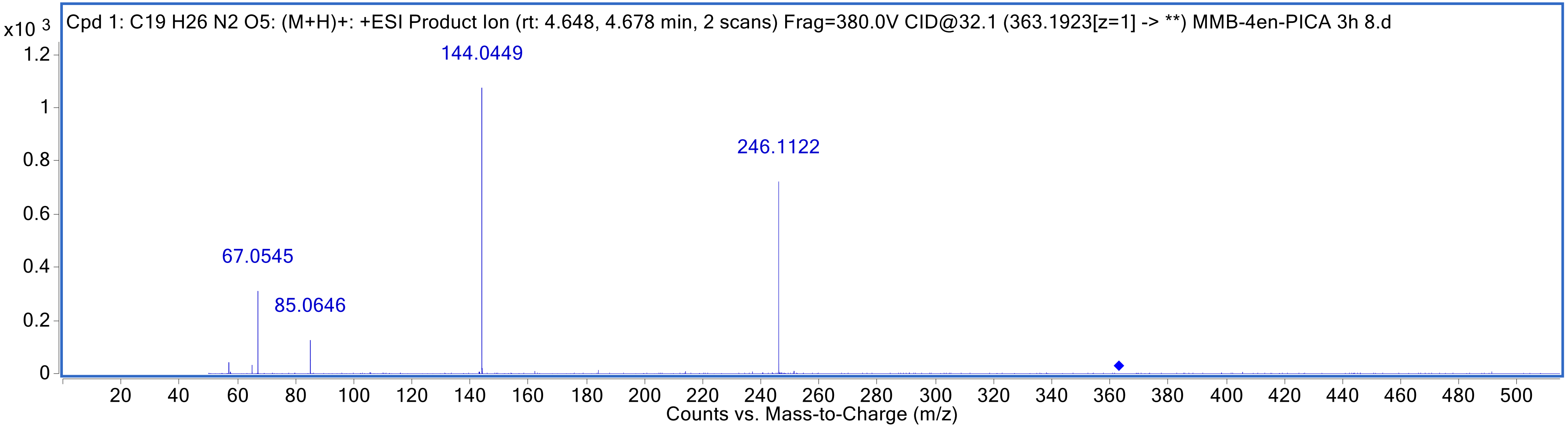

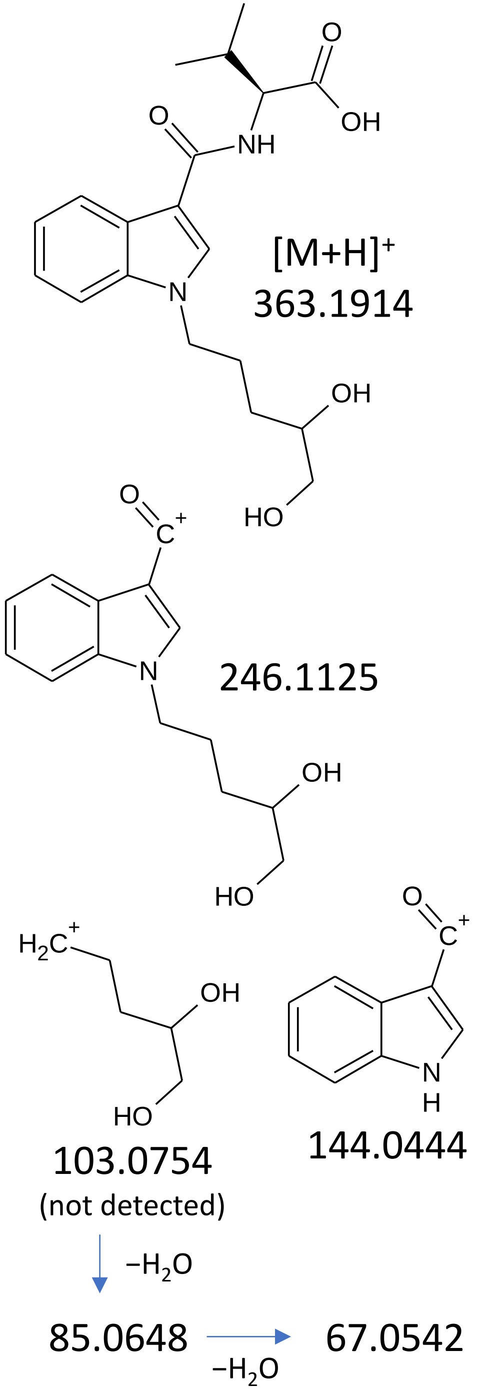

A3, Ester hydrolysis + glucuronidation, RT 7.49 min, *m/z* 505.2180

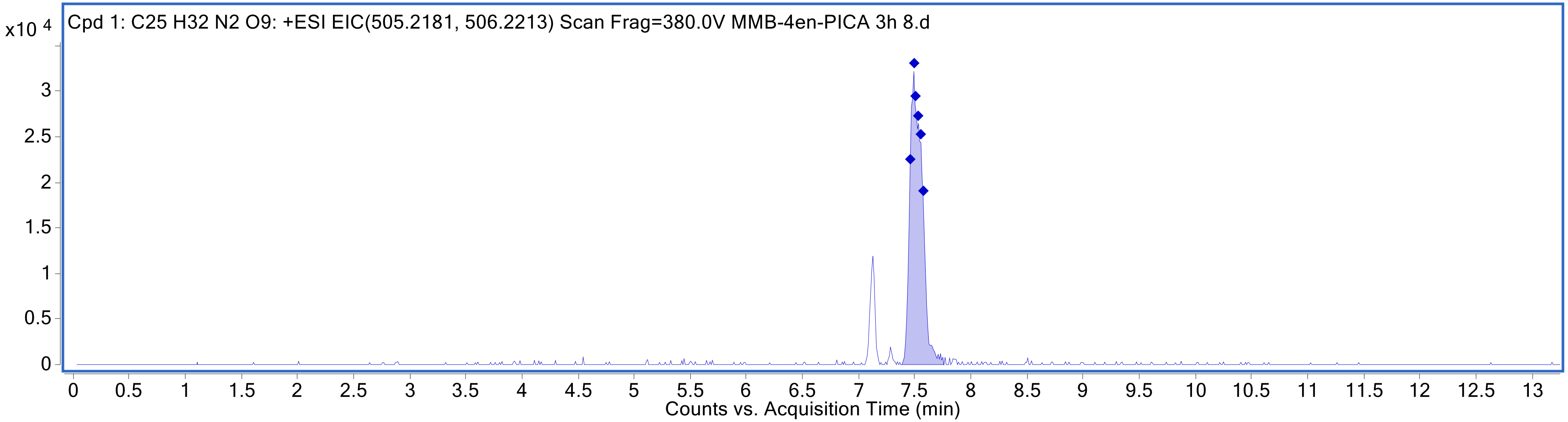

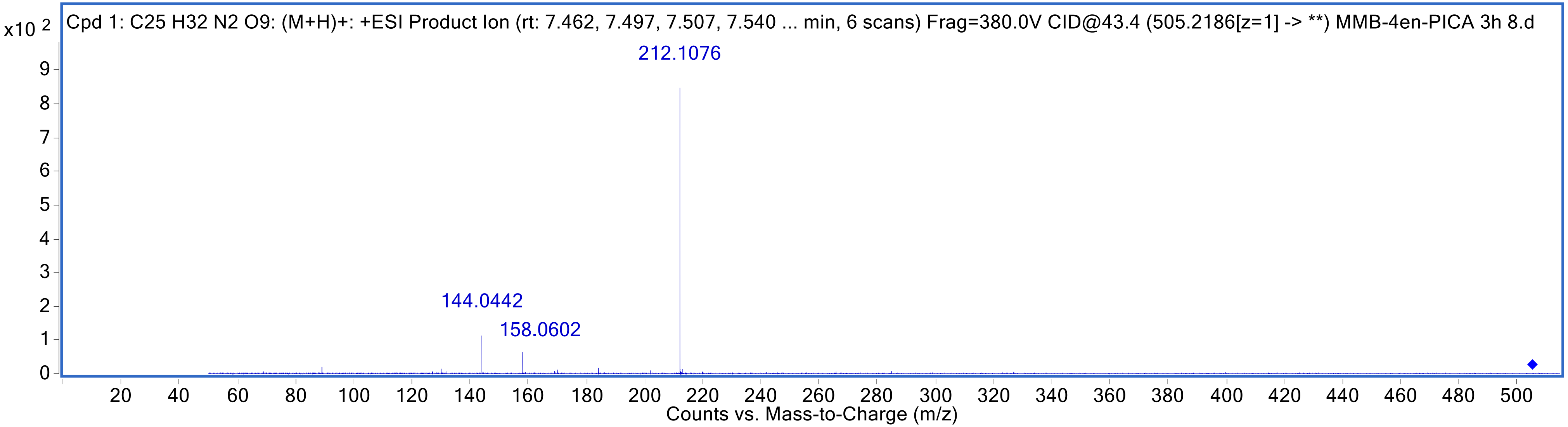

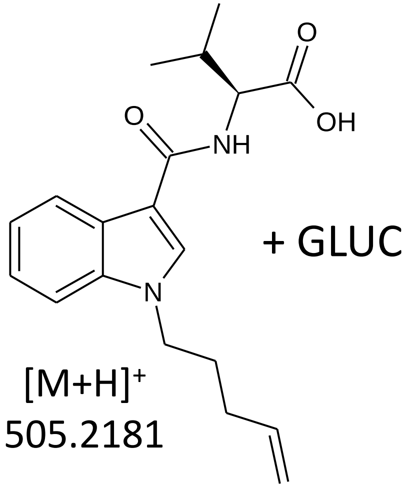

+ GLUC

$[M+H]^+$
505.2181

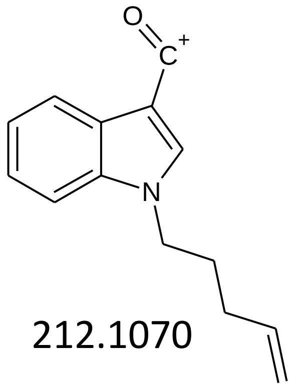

212.1070

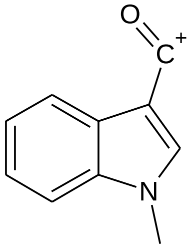

158.0600

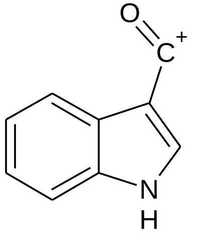

144.0444

A4, Head group–linker cleavage, RT 6.91 min, *m/z* 229.1340

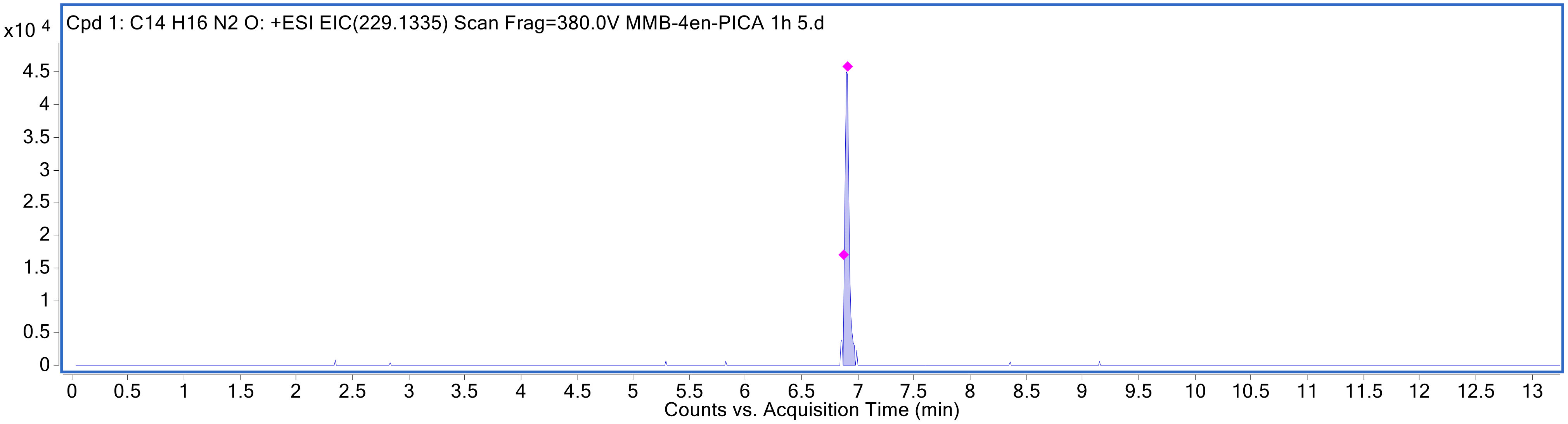

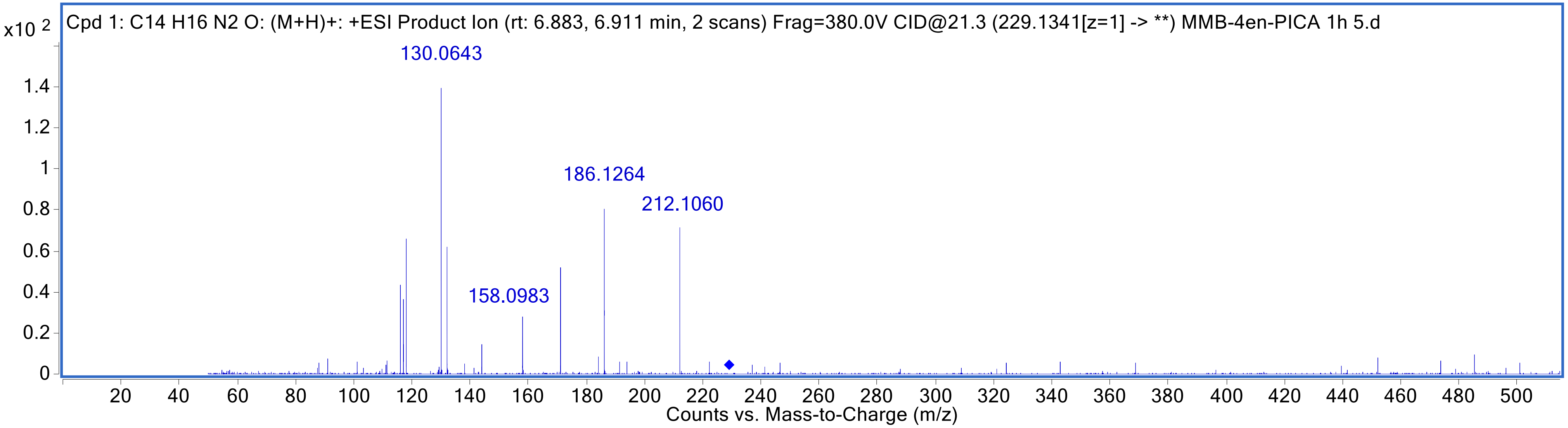

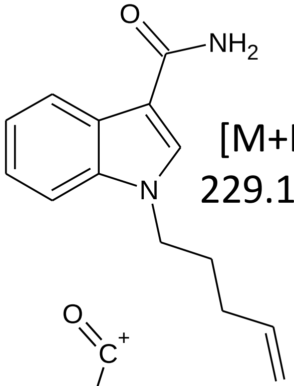

$[M+H]^+$ 229.1335

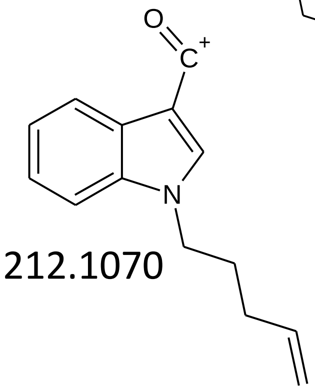

212.1070

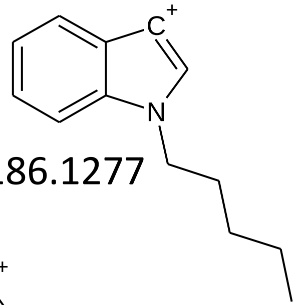

186.1277

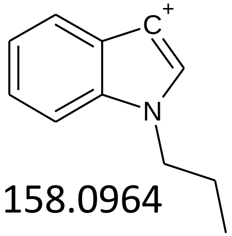

158.0964

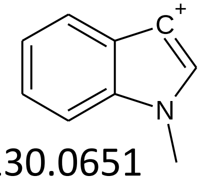

130.0651

A5, Ester hydrolysis + mono-hydroxylation (pentenyl tail), RT 6.31 min, *m/z* 345.1811

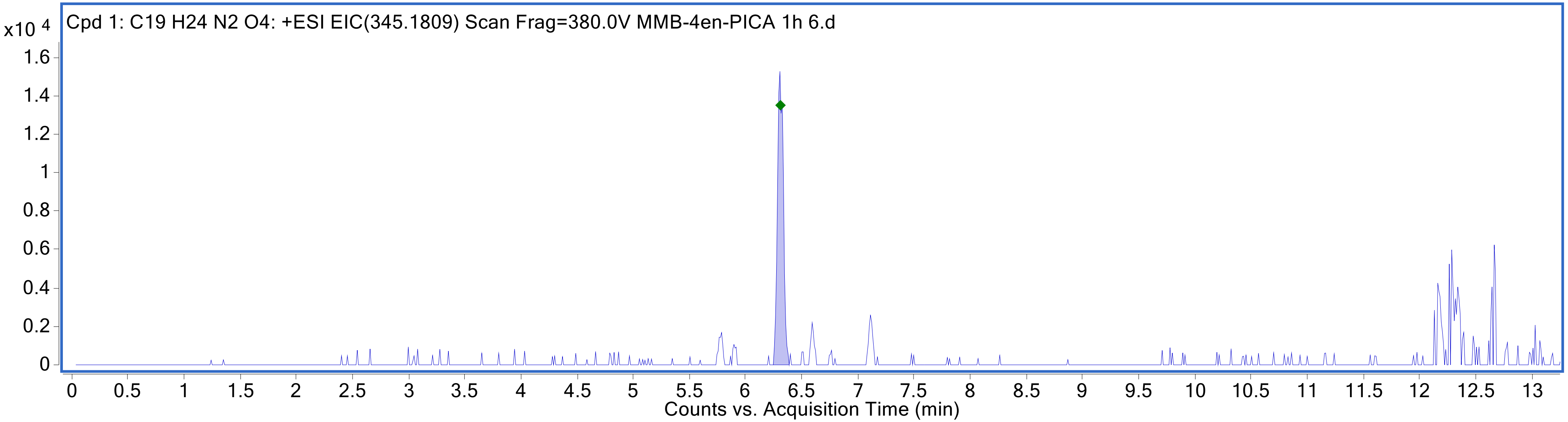

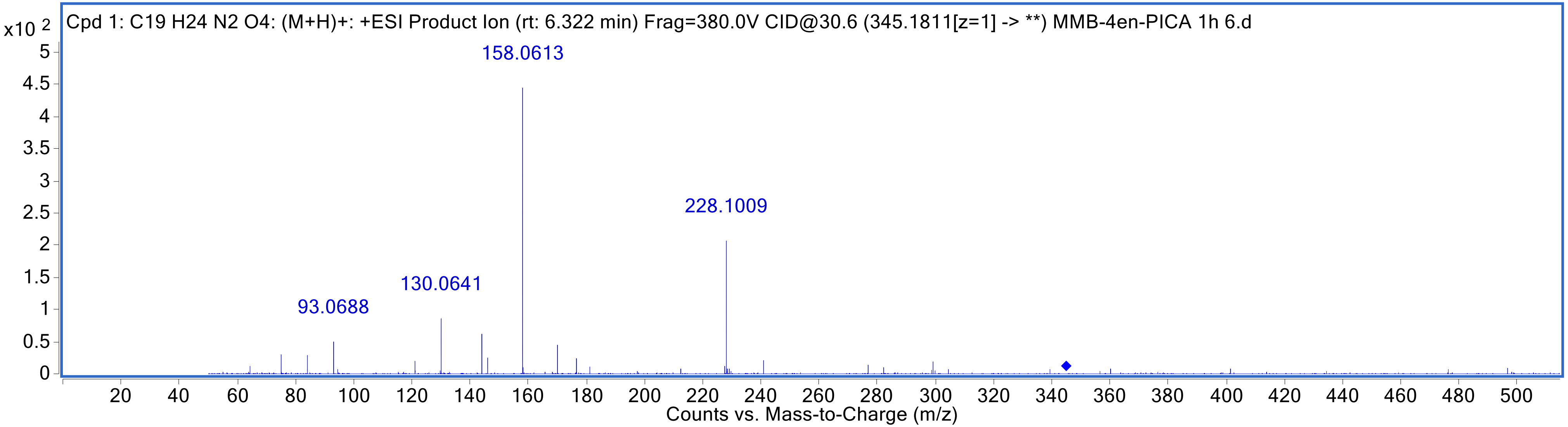

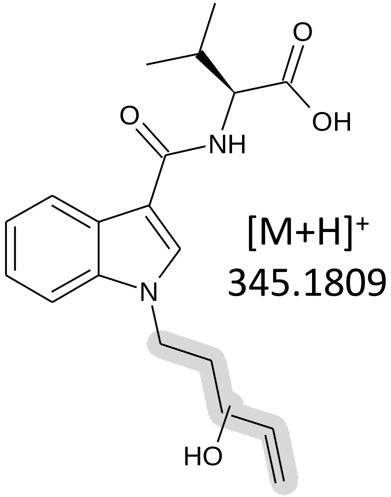

[M+H]+
345.1809

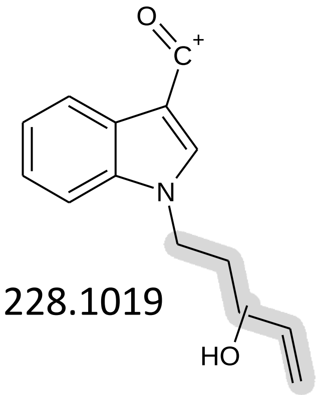

228.1019

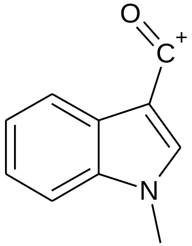

158.0600

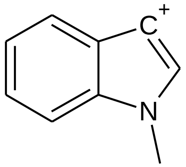

130.0651

A6, Secondary amide hydrolysis + mono-hydroxylation (pentenyl tail), RT 4.66 min, *m/z* 246.1129

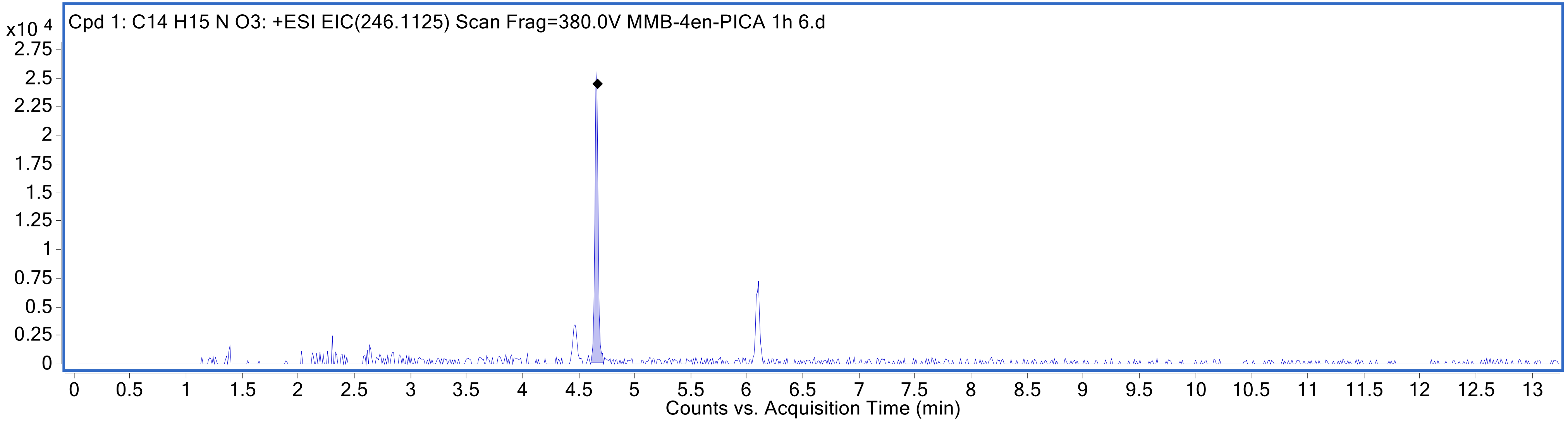

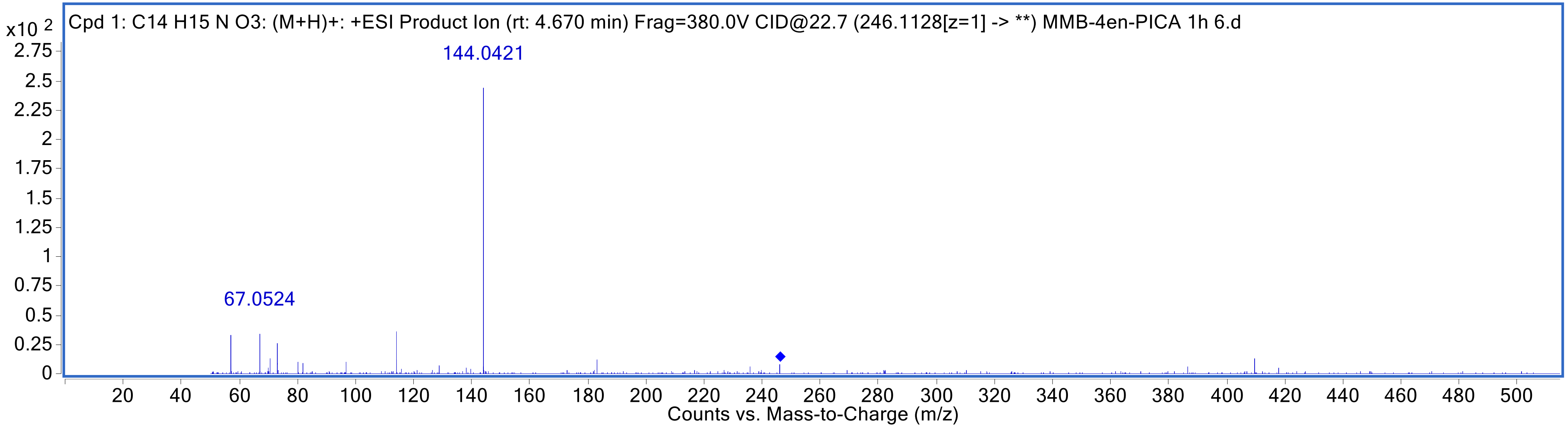

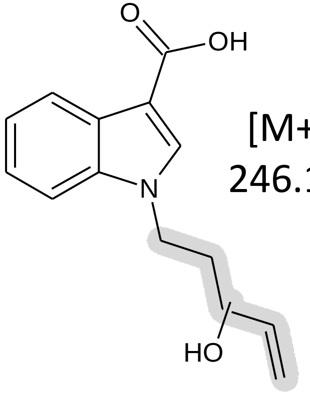

$[M+H]^+$
246.1125

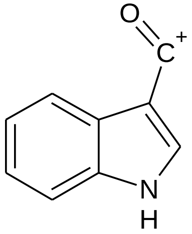

144.0444

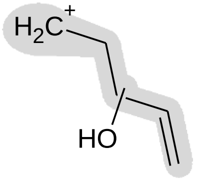

85.0648 (not detected) → $-H_2O$ 67.0542

A7, Dihydrodiol formation, RT 6.09 min, *m/z* 377.2069

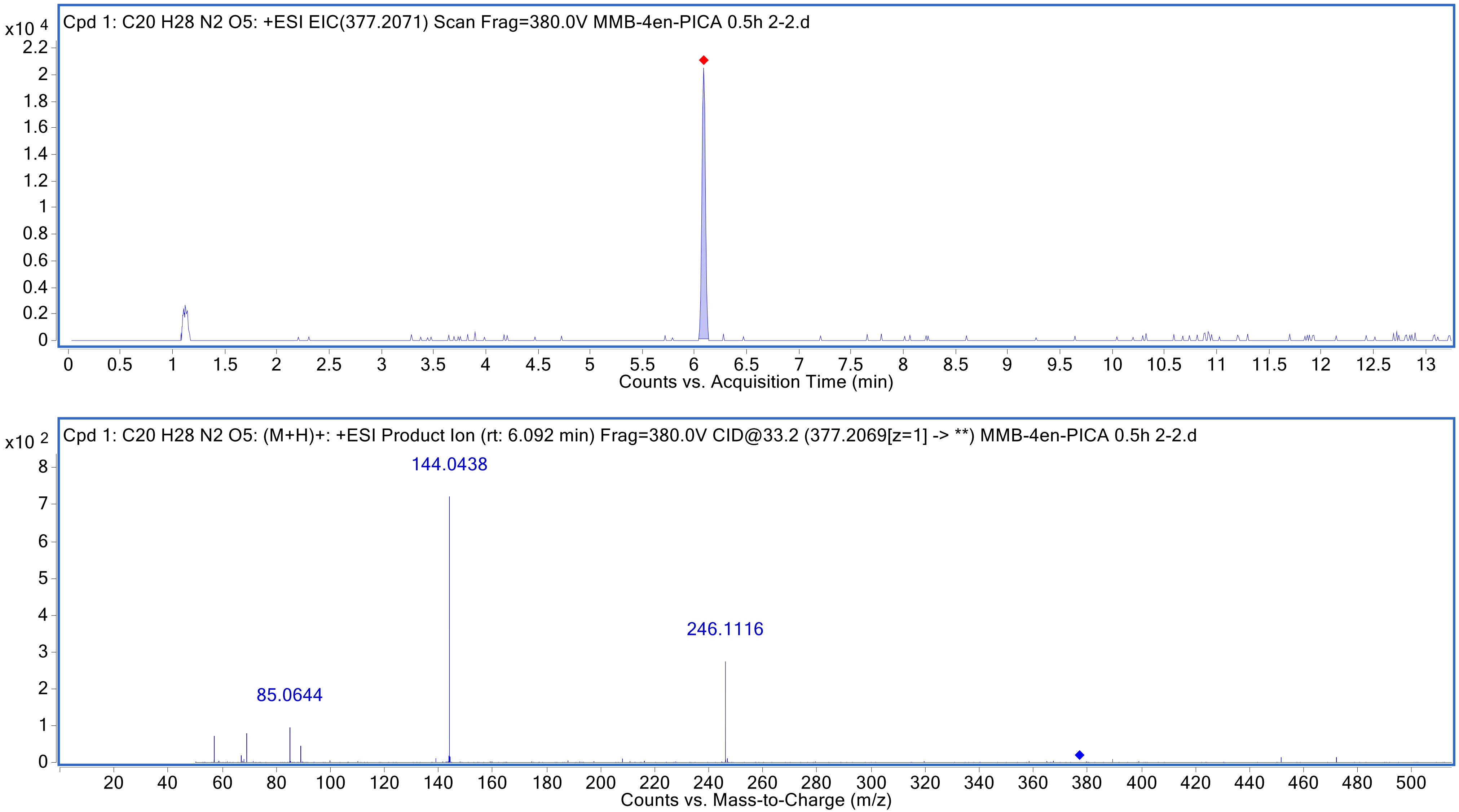

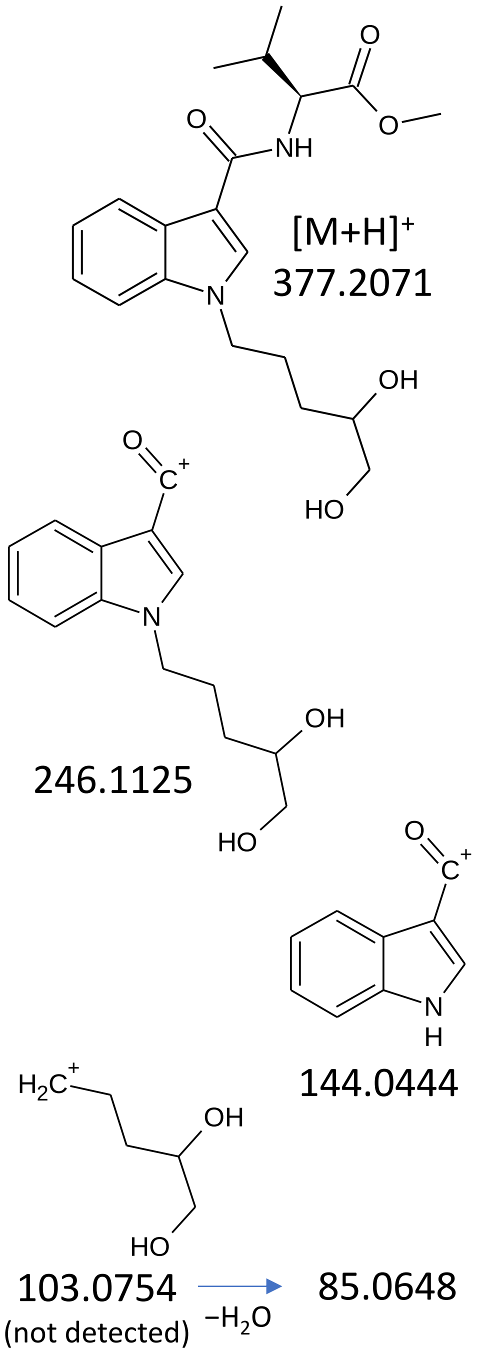

Dihydrodiol reference standard, RT 5.46 min, *m/z* 377.2067

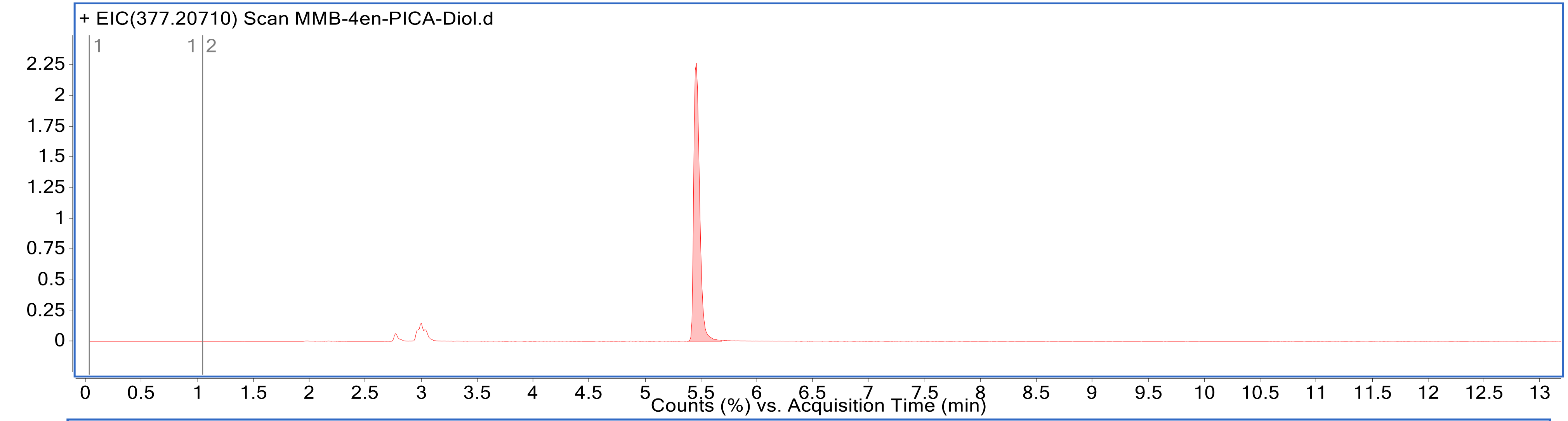

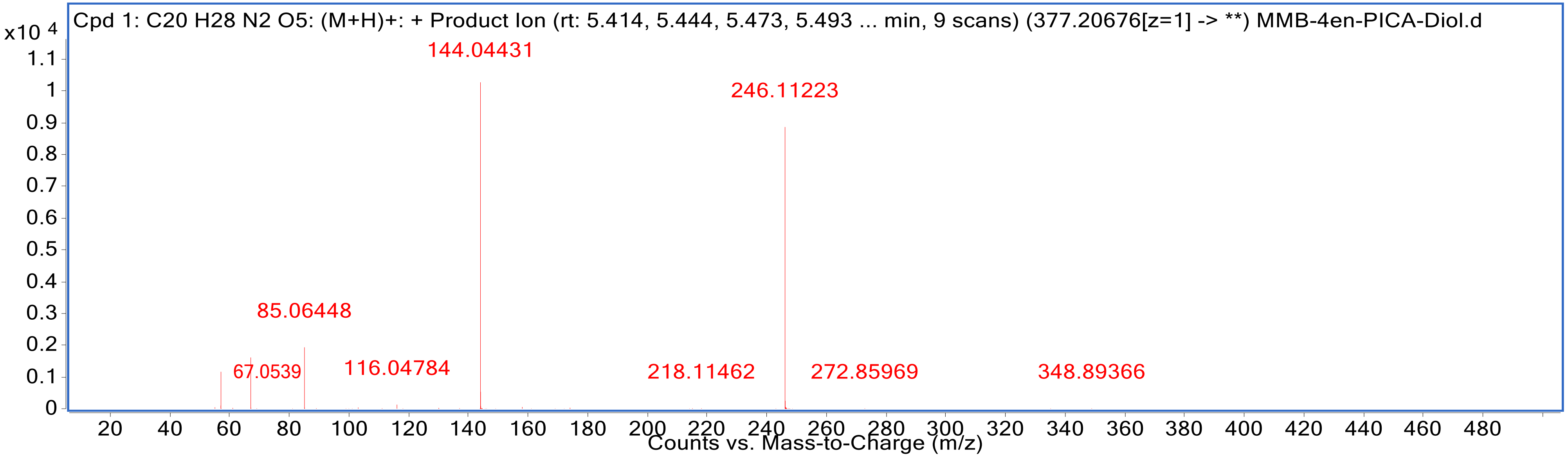

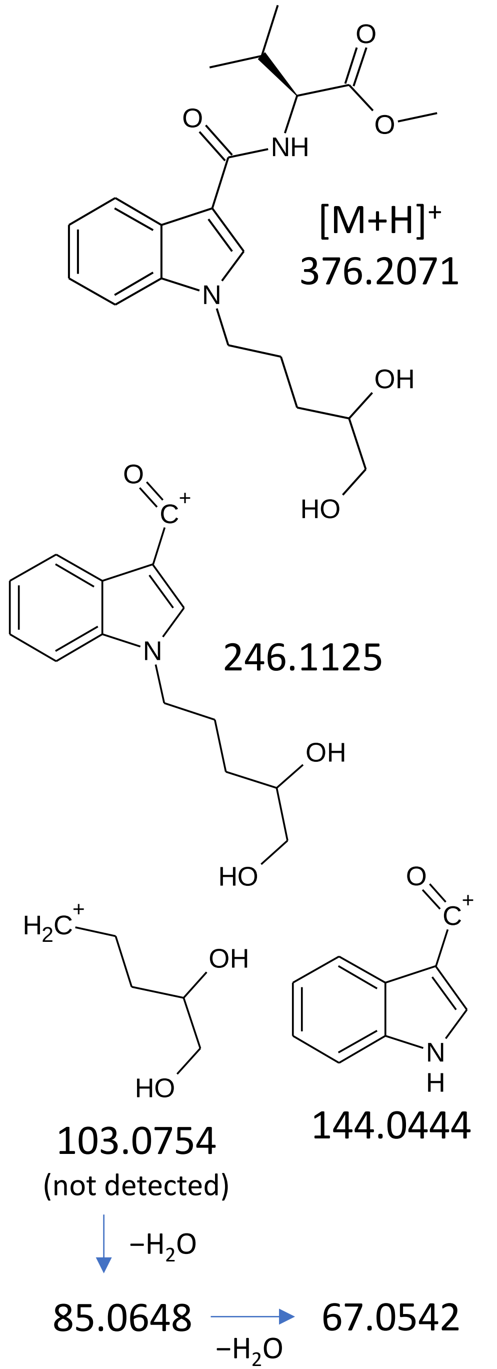

MMB-4en-PINACA

Metabolism

MMB-4en-PINACA, RT 12.05 min, *m/z* 344.1989

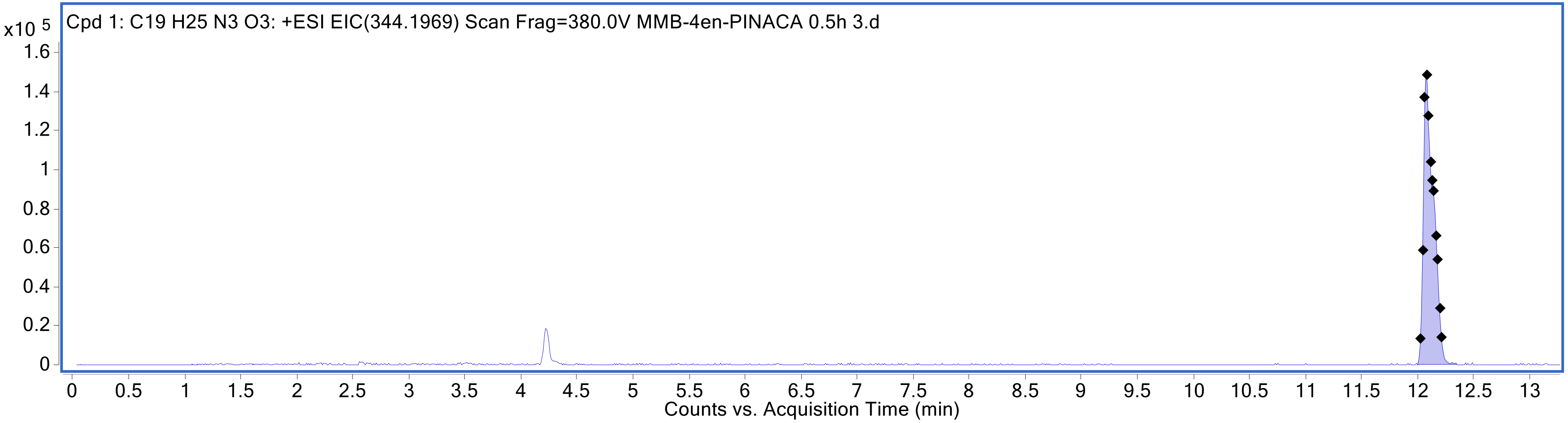

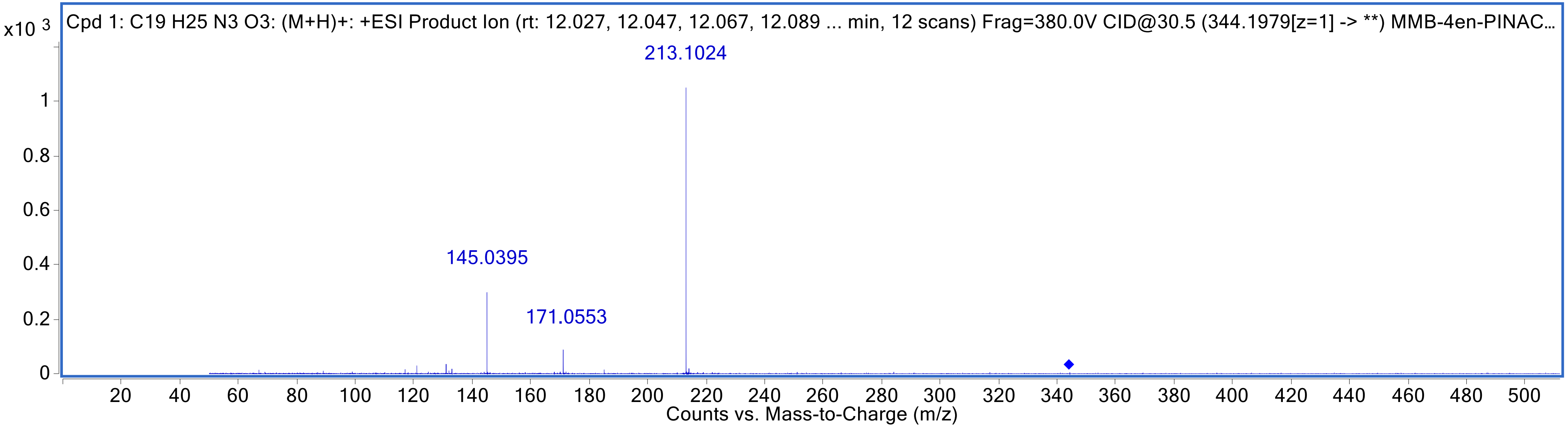

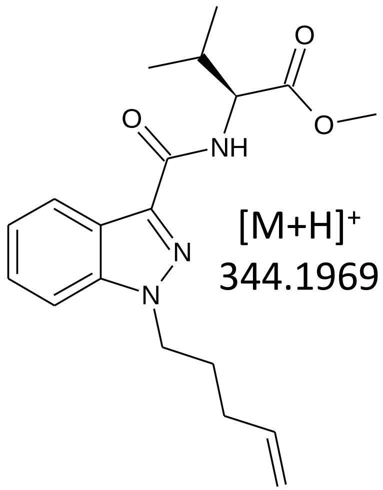

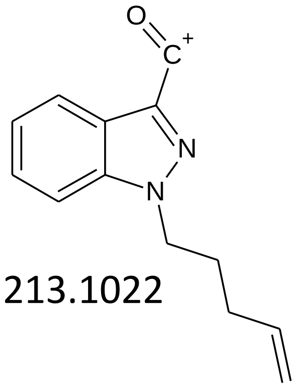

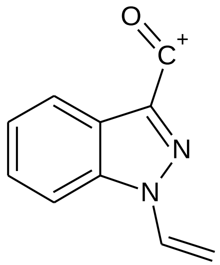

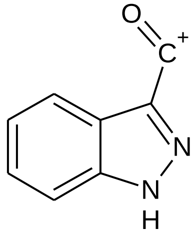

B1, Ester hydrolysis, RT 9.72 min, *m/z* 330.1827

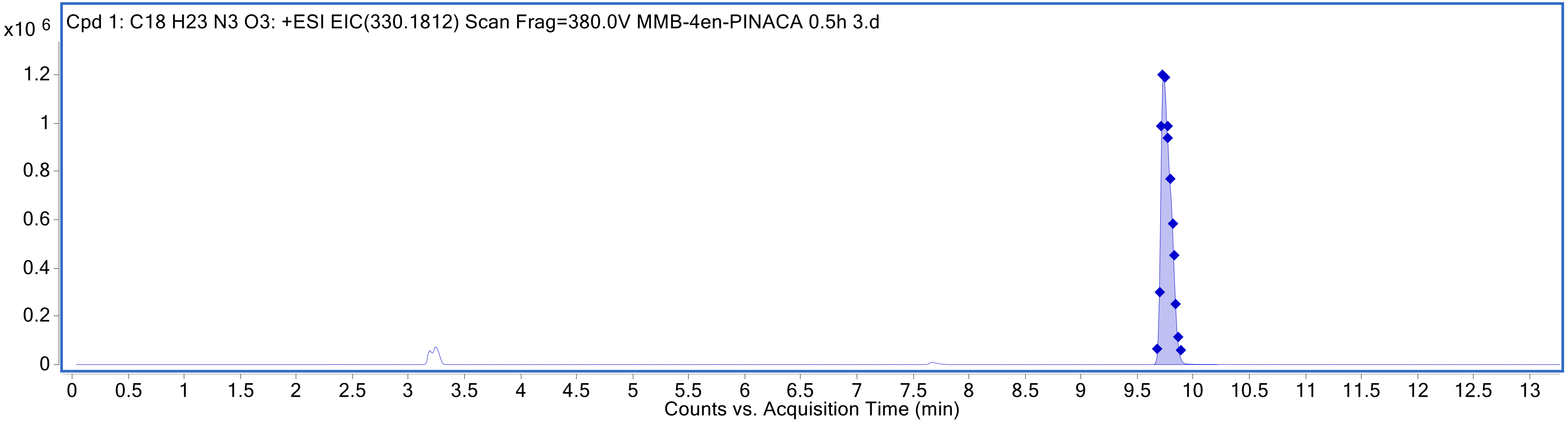

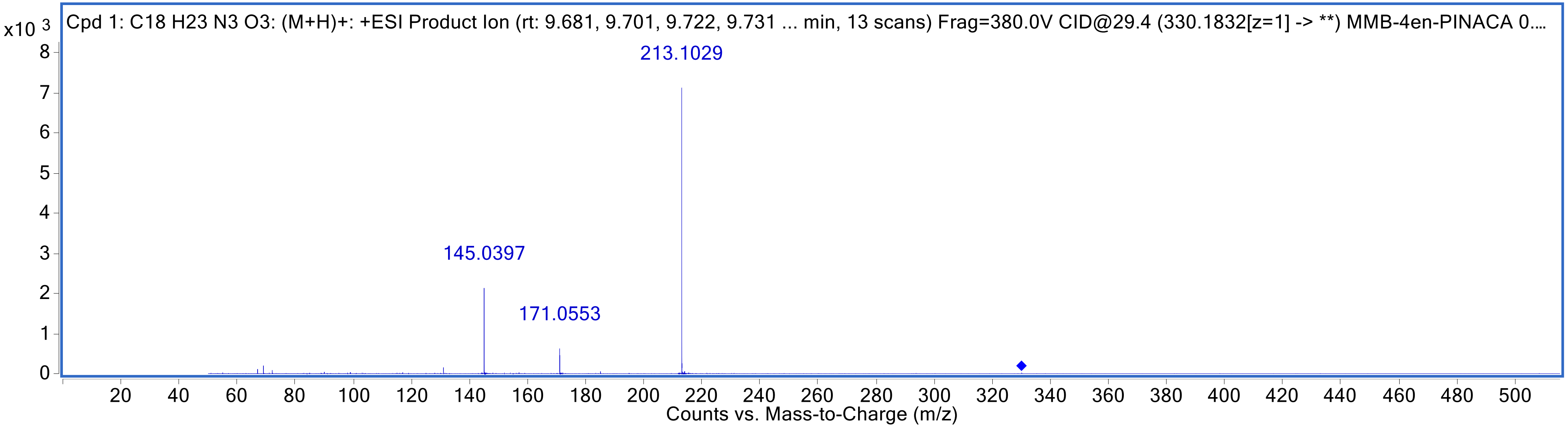

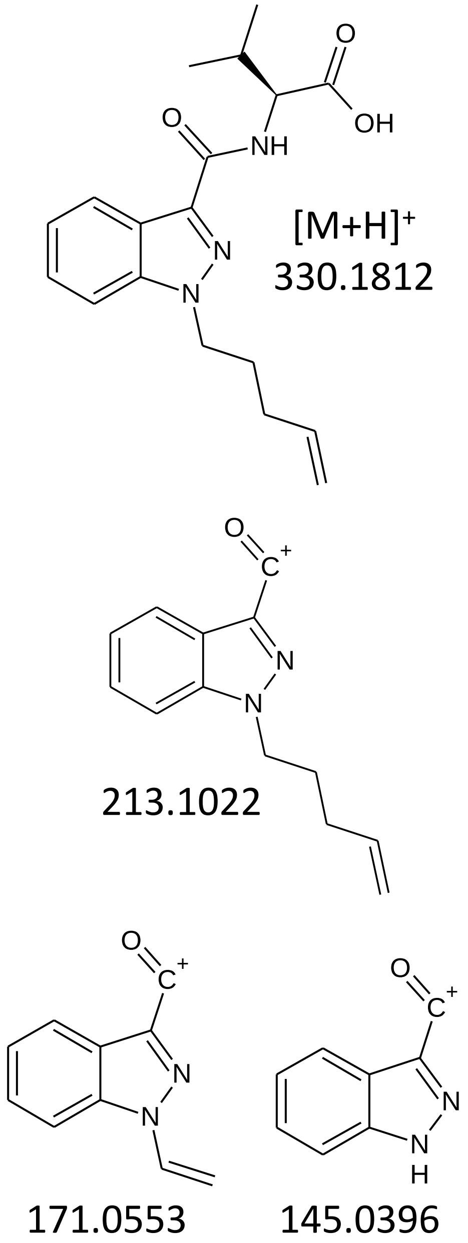

B2, Ester hydrolysis + dihydrodiol formation, RT 4.85 min, *m/z* 364.1873

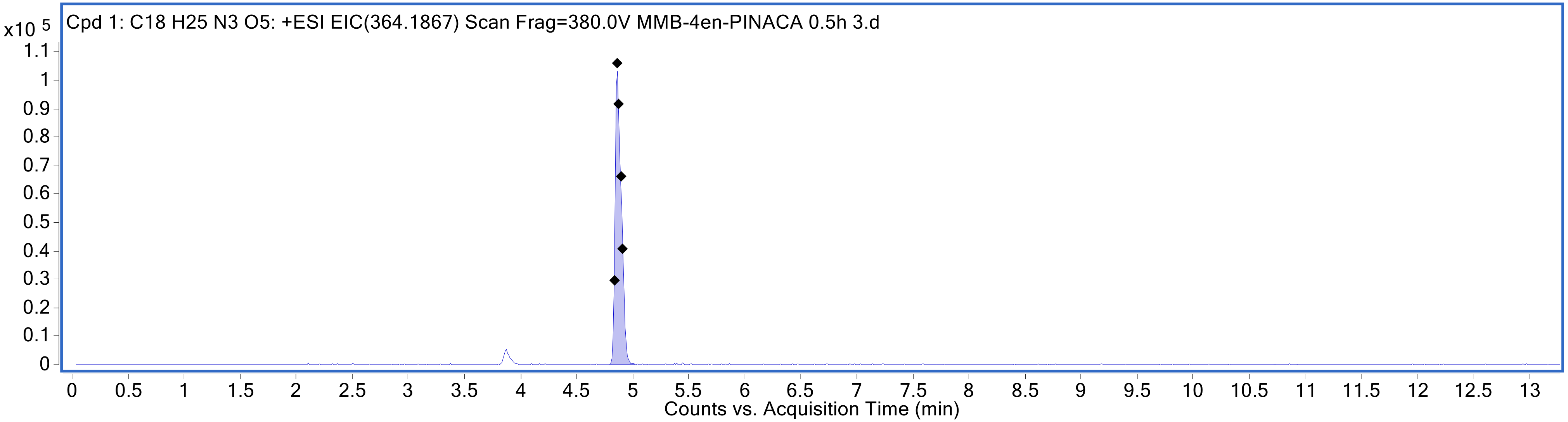

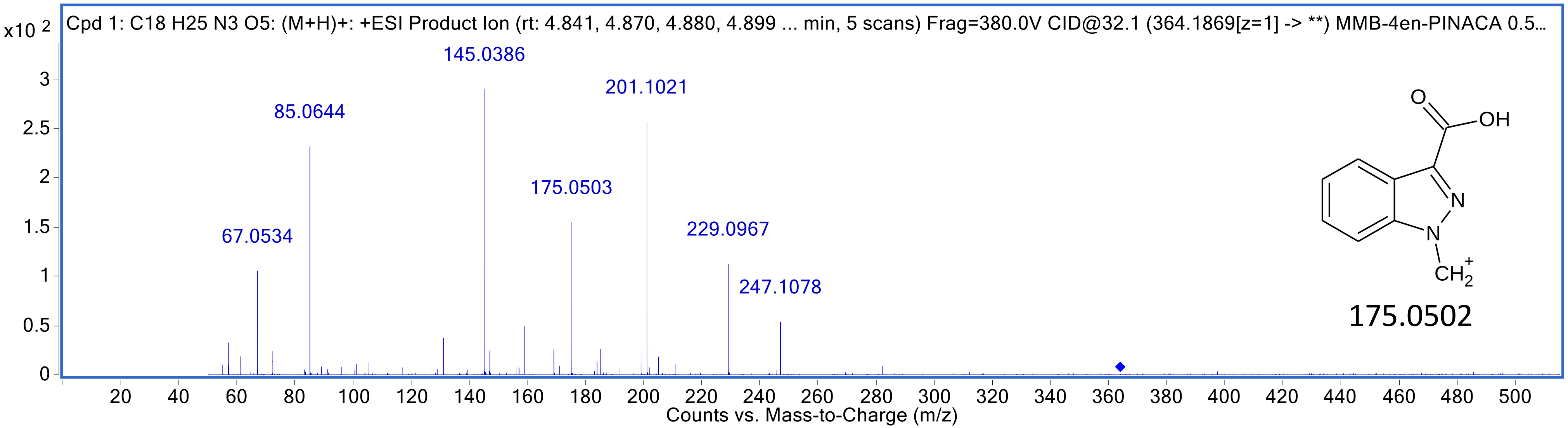

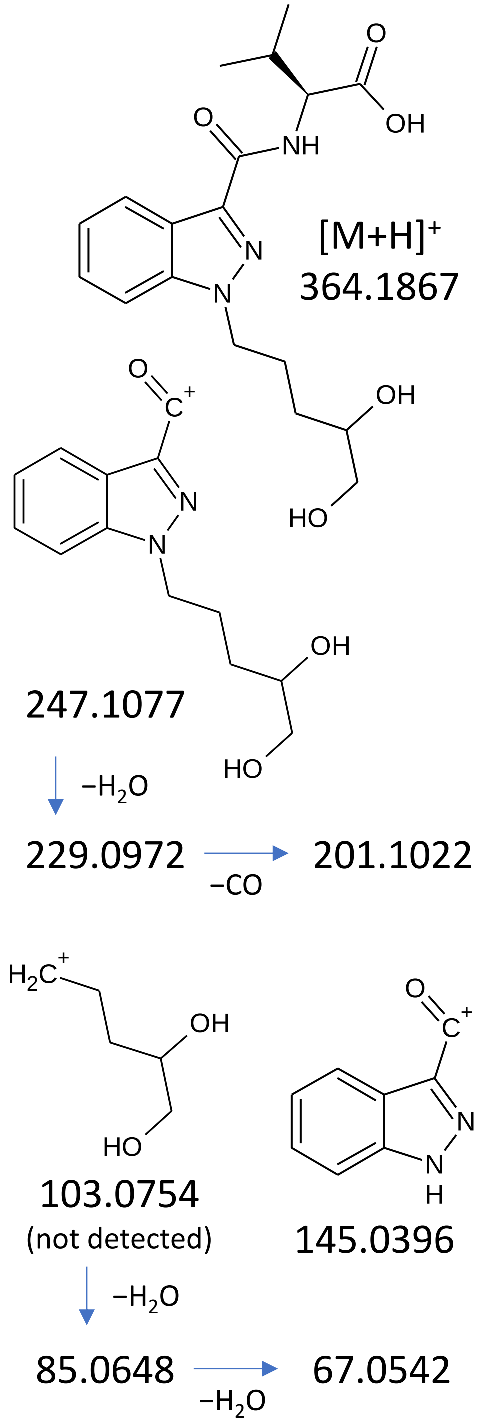

B3, Ester hydrolysis + glucuronidation, RT 7.68 min, *m/z* 506.2122

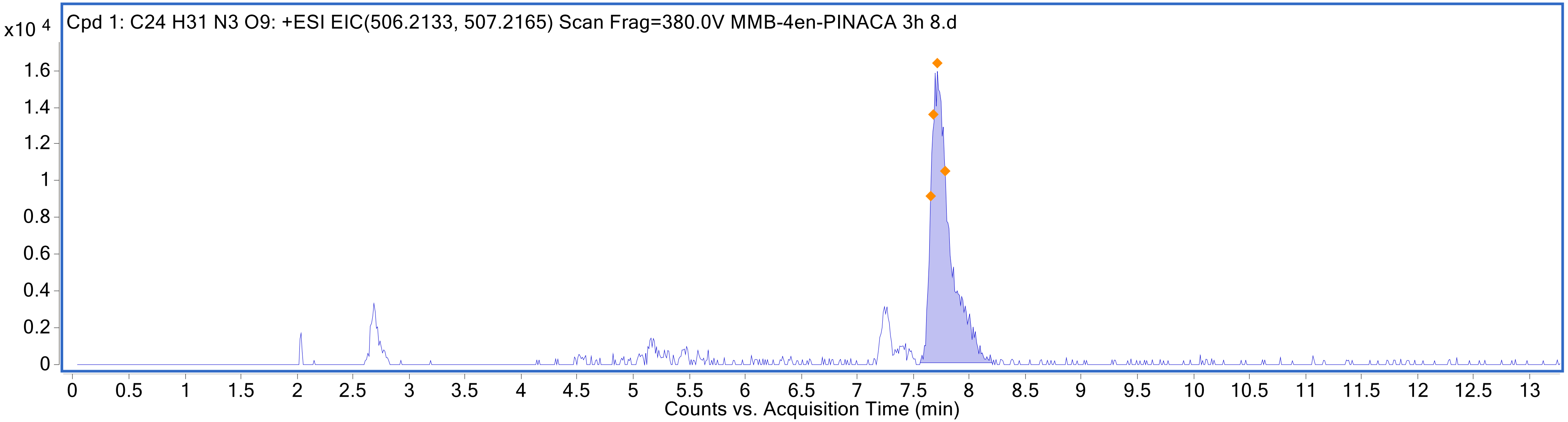

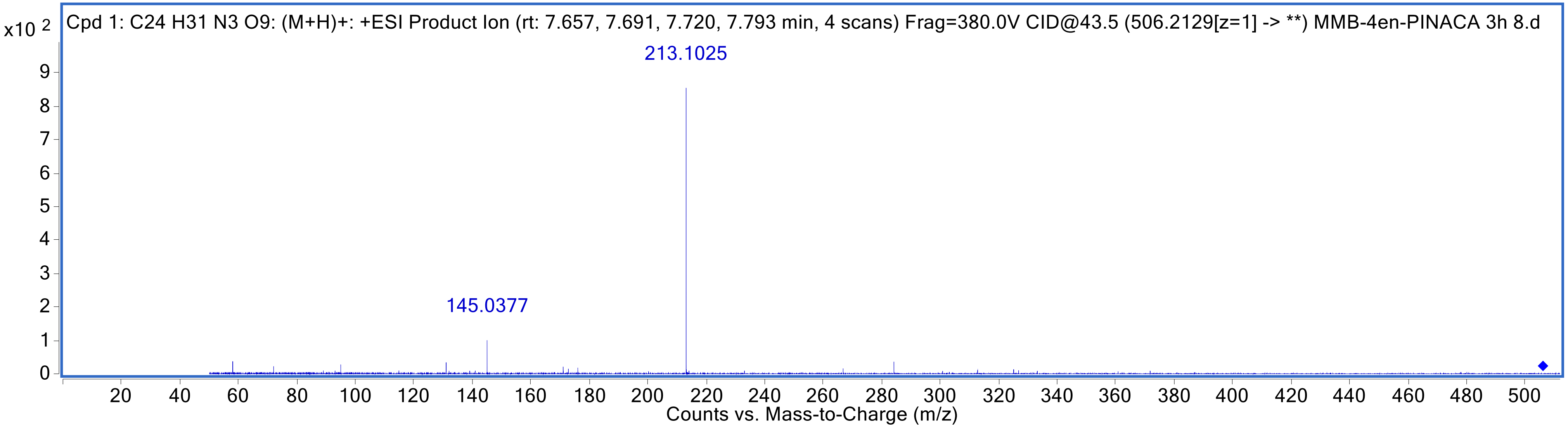

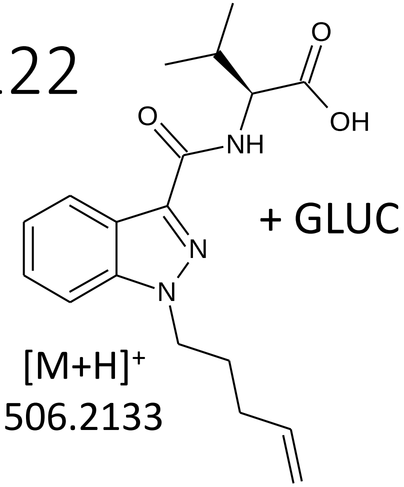

+ GLUC

[M+H]+
506.2133

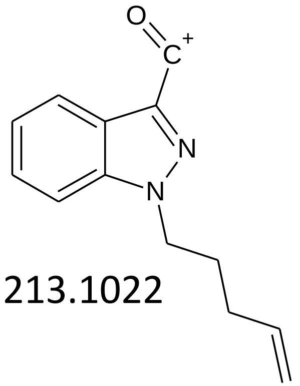

213.1022

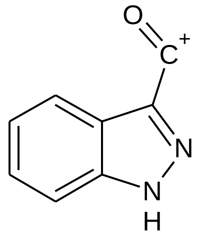

145.0396

B4, Ester hydrolysis + mono-hydroxylation (pentenyl tail), RT 6.55 min, *m/z* 346.1756

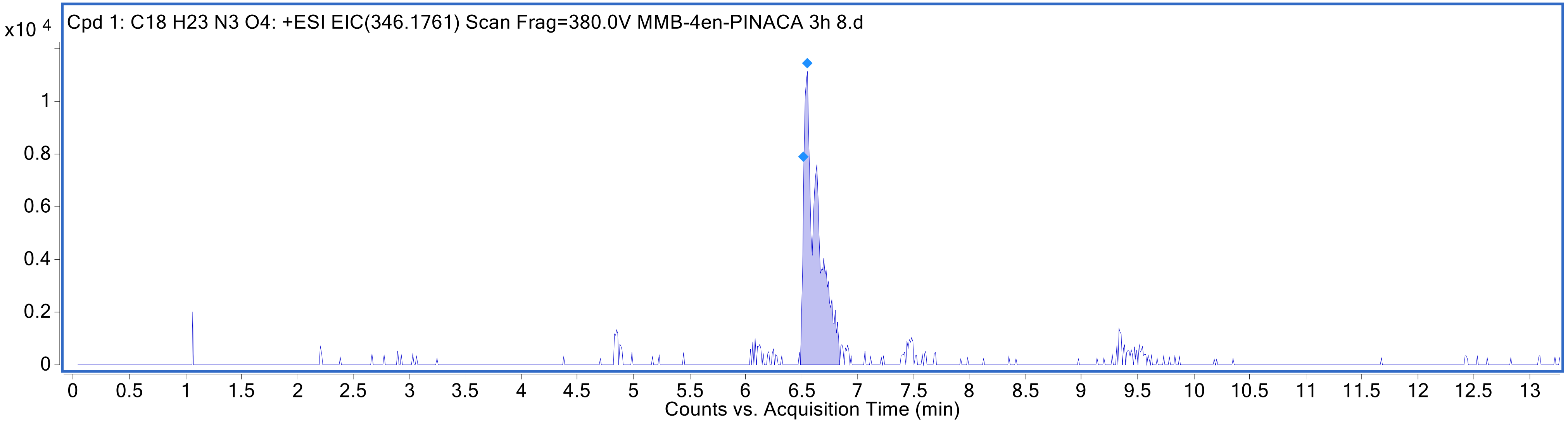

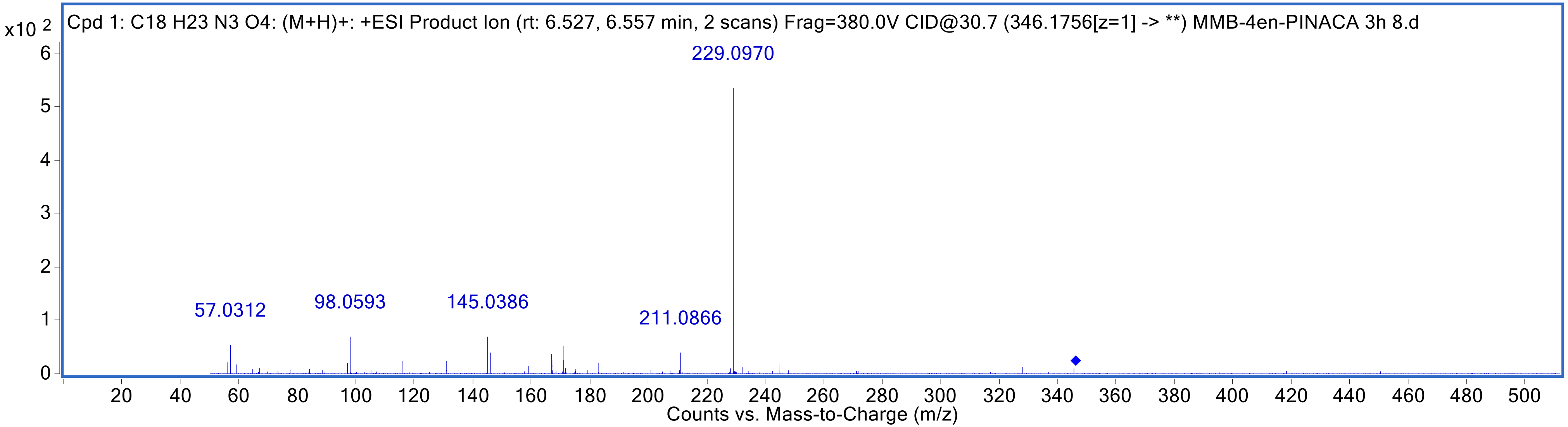

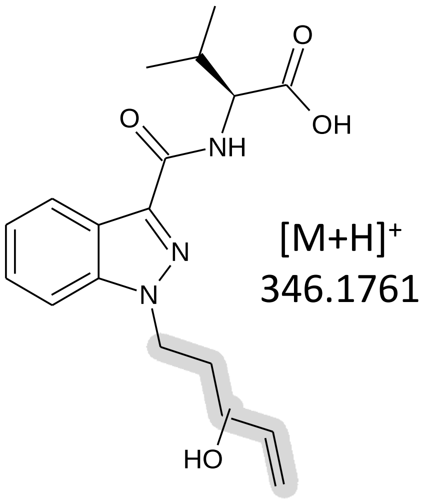

$[M+H]^+$
346.1761

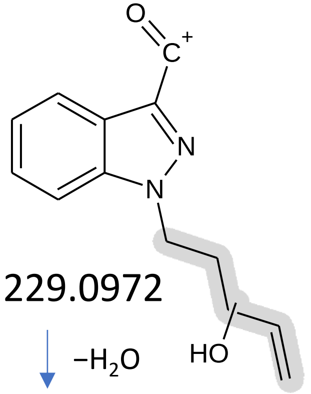

229.0972
$\downarrow -H_2O$
211.0866

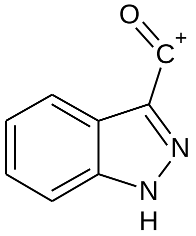

145.0396

B5, Ester hydrolysis + di-hydroxylation (pentenyl tail), RT 5.39 min, *m/z* 362.1709

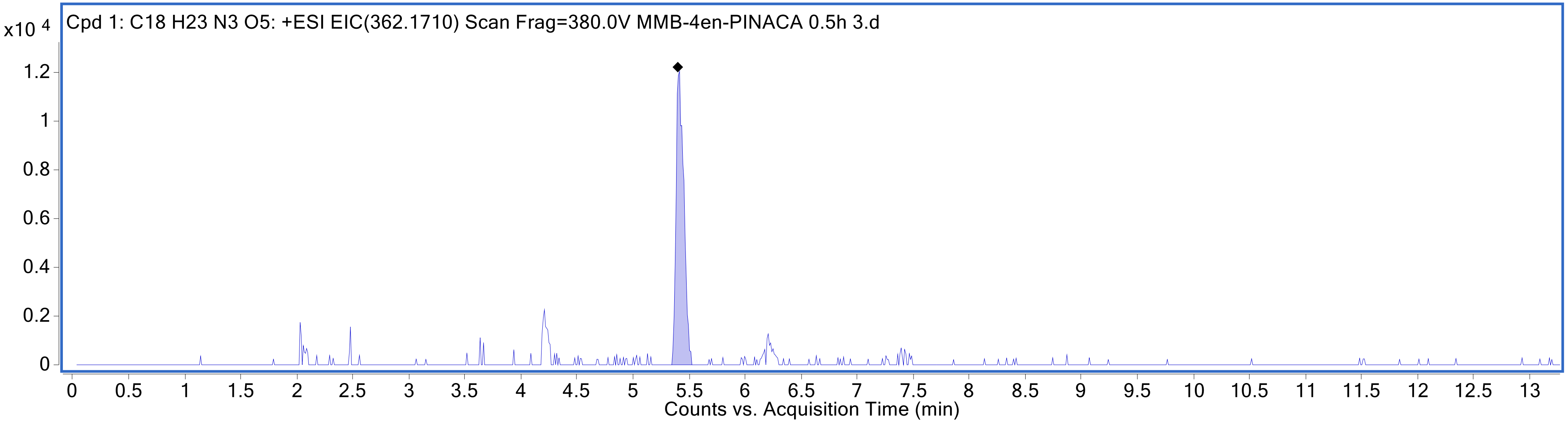

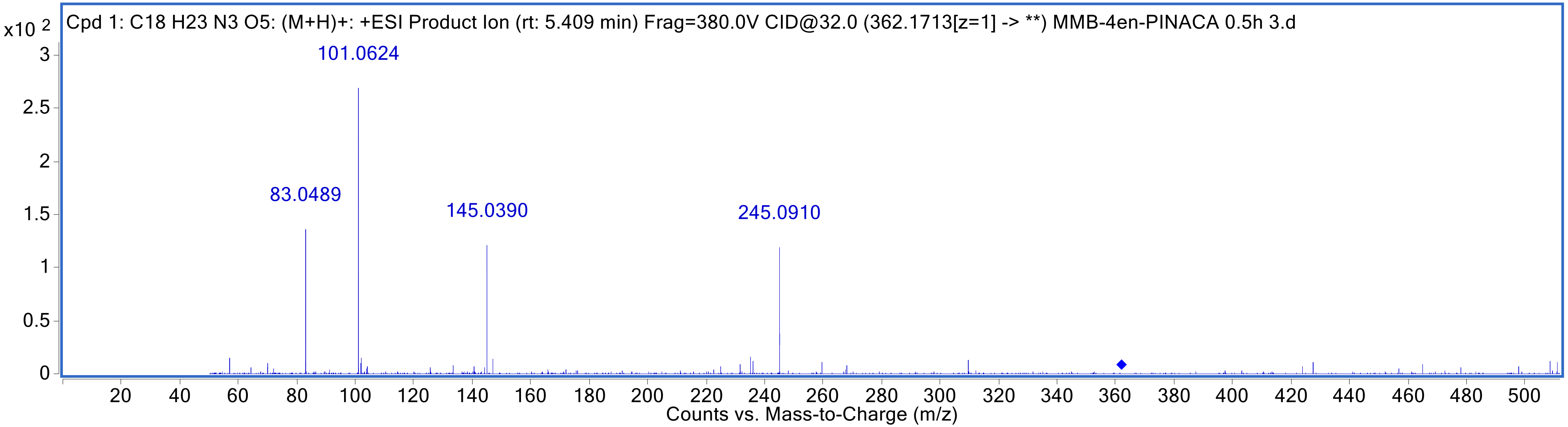

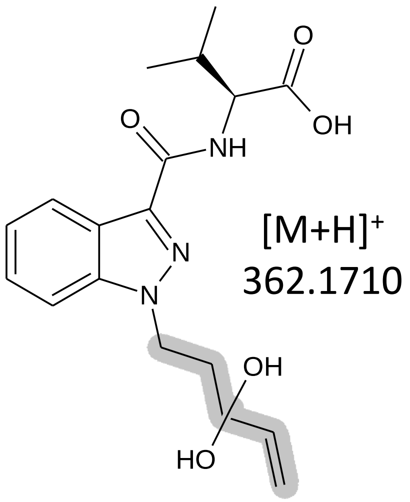

$[M+H]^+$

362.1710

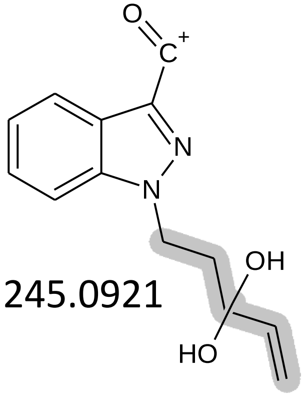

245.0921

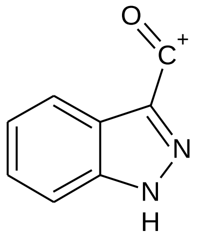

145.0396

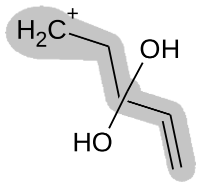

101.0597

↓ $-H_2O$

83.0491

B6, Dihydrodiol formation, RT 6.36 min, *m/z* 378.2026

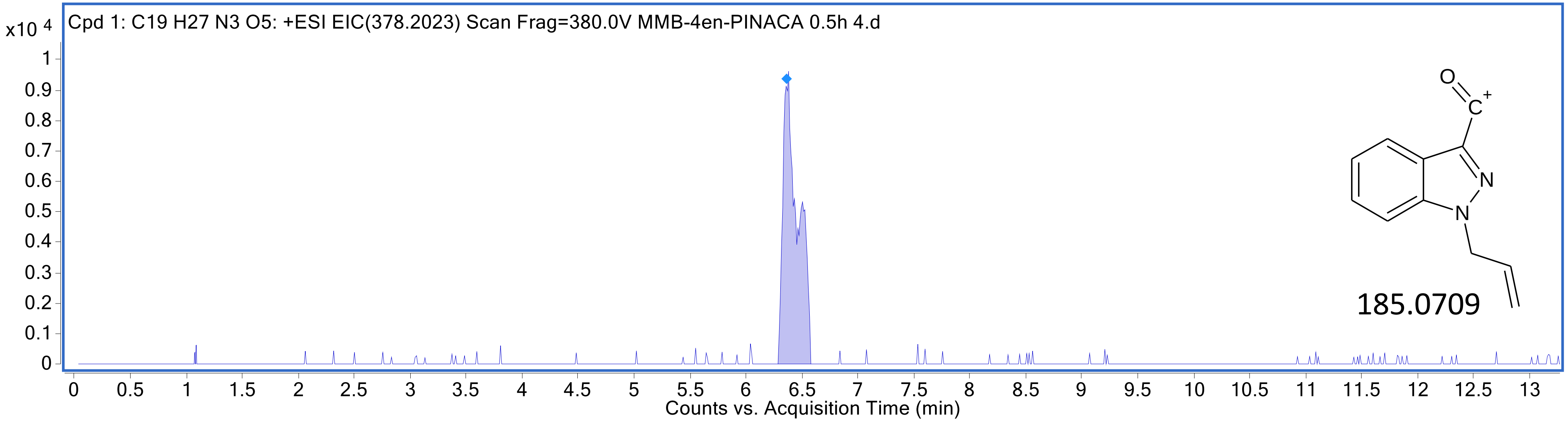

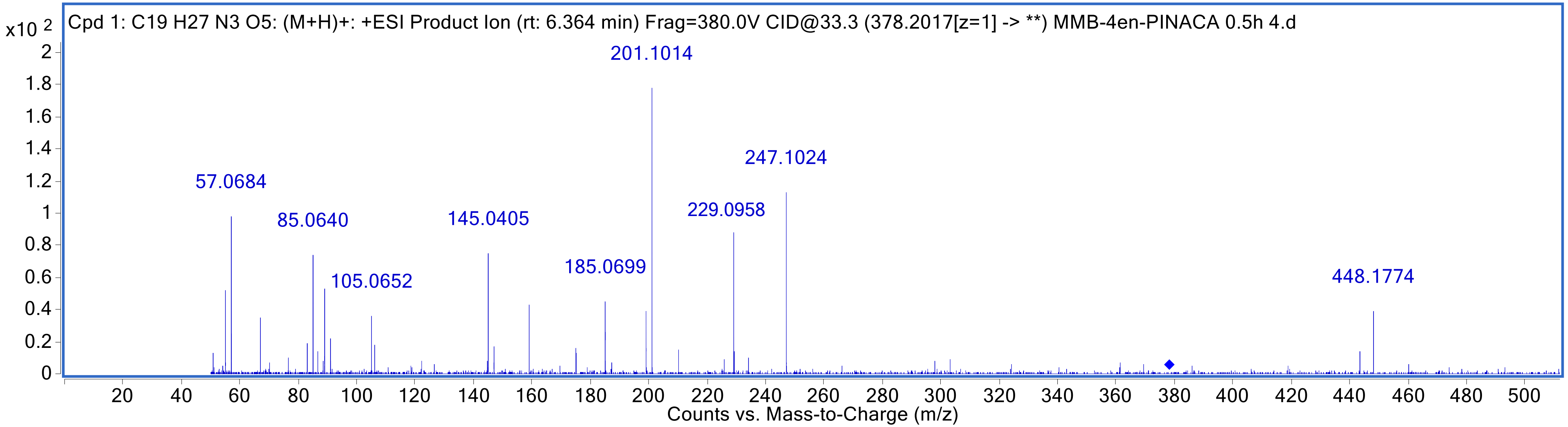

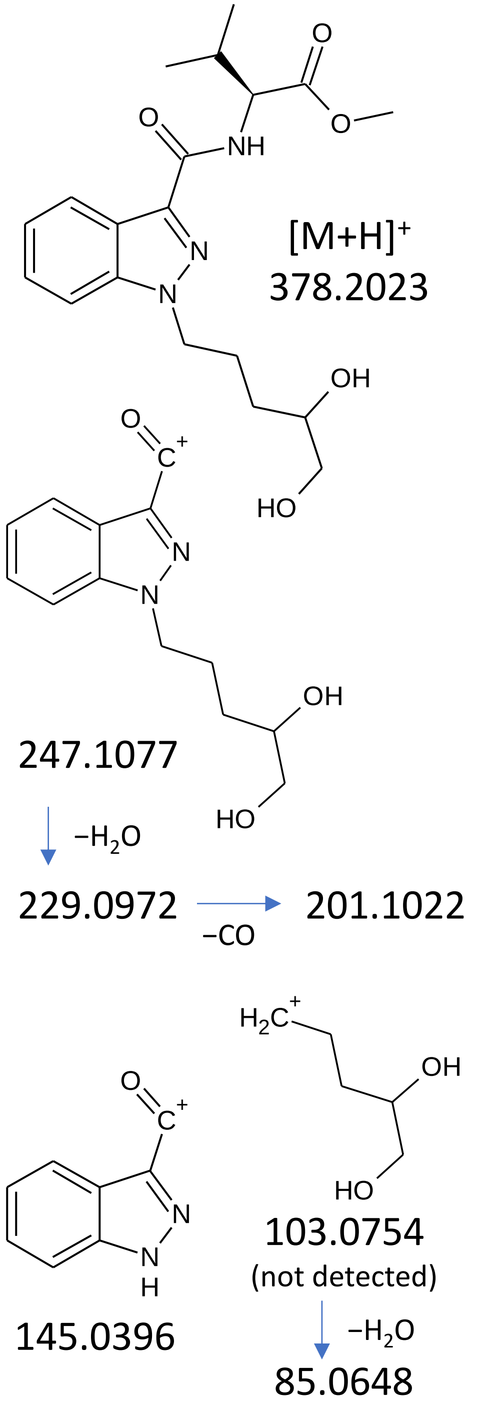

Dihydrodiol reference standard, RT 6.39 min, *m/z* 378.2031

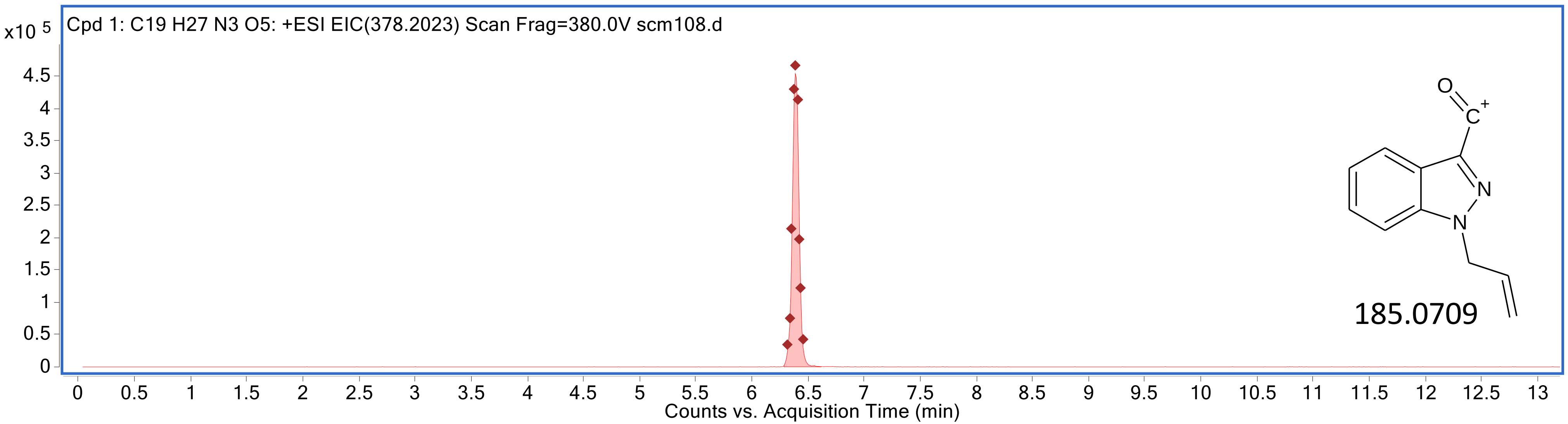

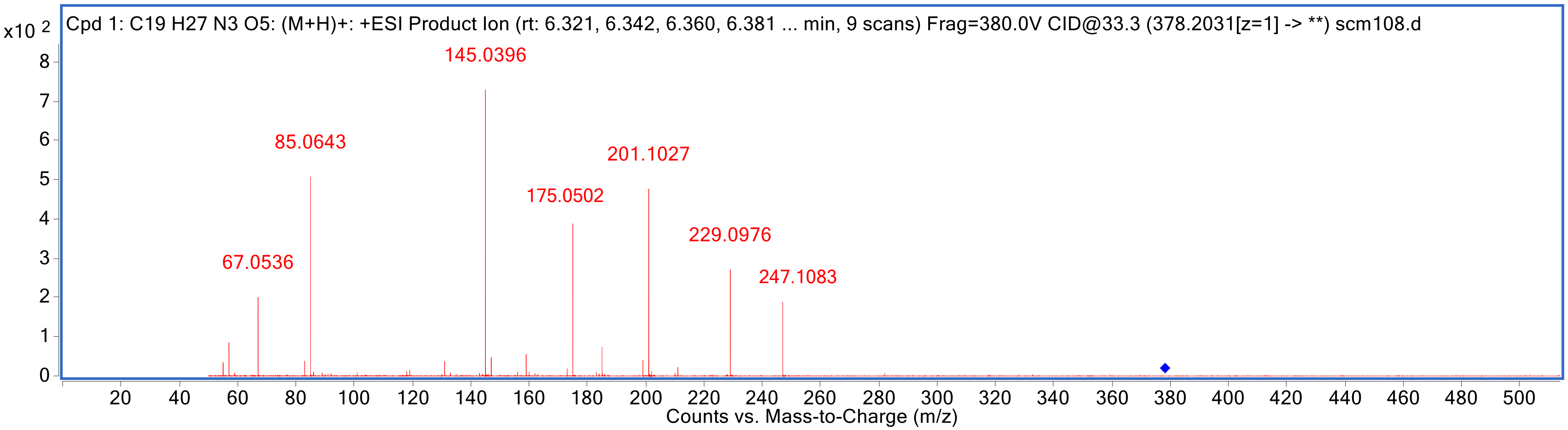

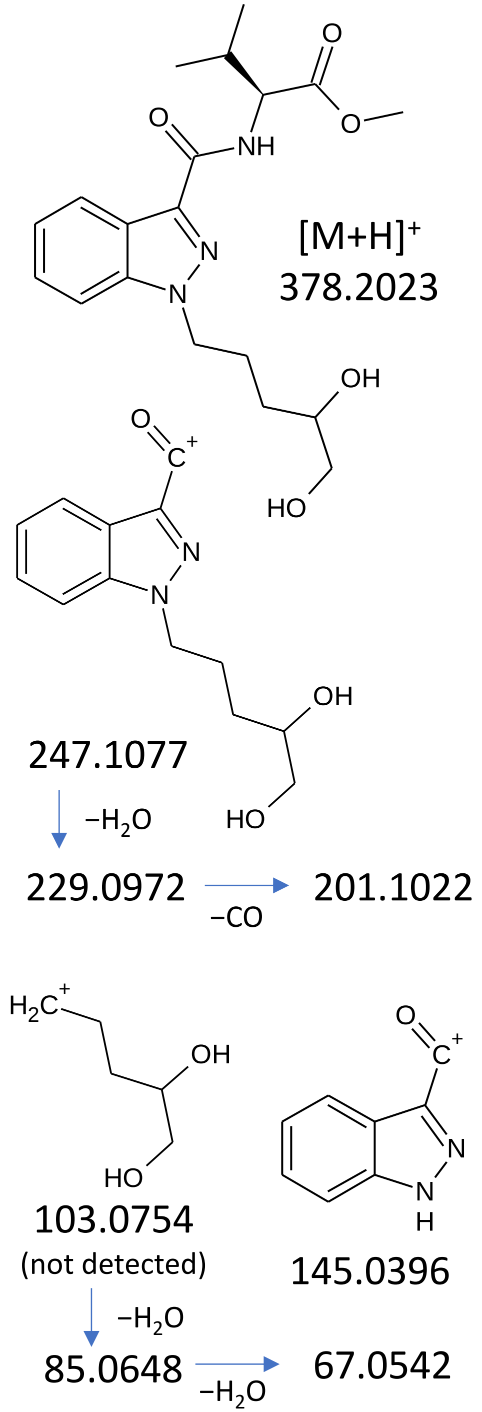

EMB-4en-PICA

Metabolism

EMB-4en-PICA, RT 10.62 min, *m/z* 357.2188

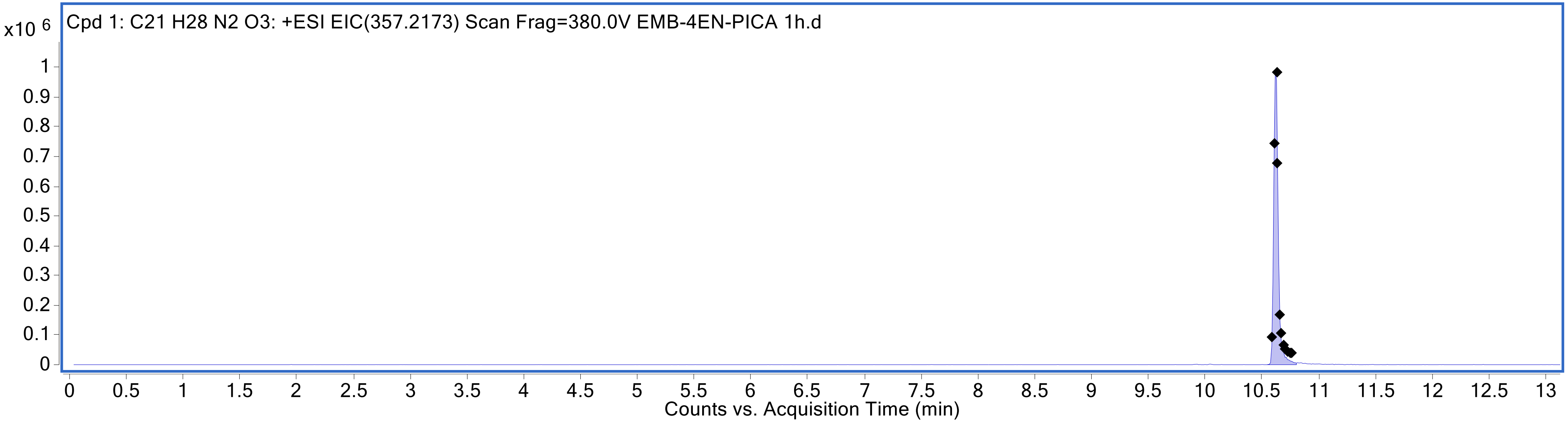

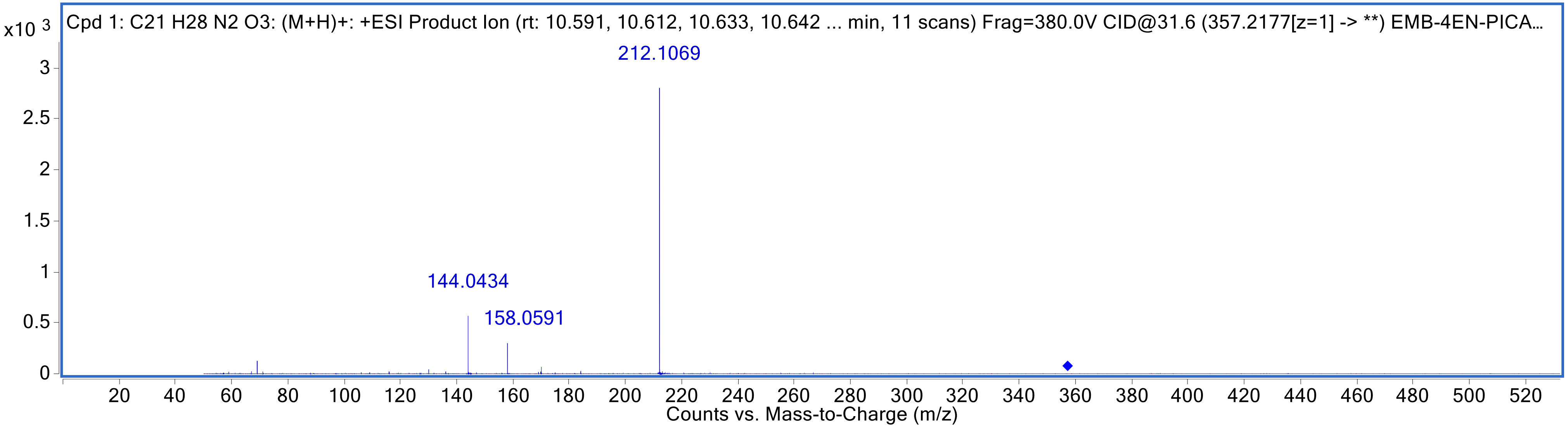

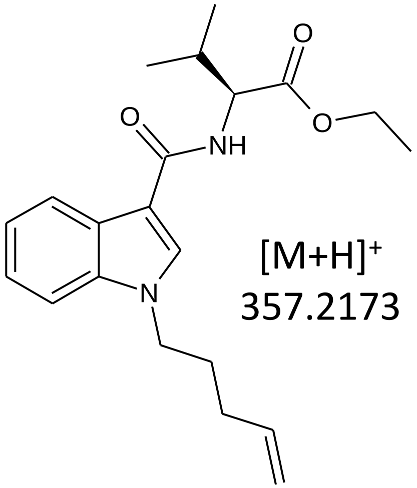

$[M+H]^+$
357.2173

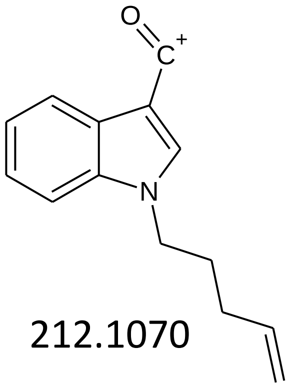

212.1070

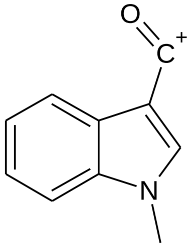

158.0600

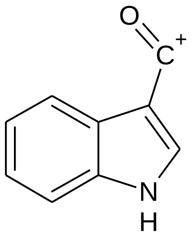

144.0444

C1, Ester hydrolysis, RT 8.28 min, *m/z* 329.1868

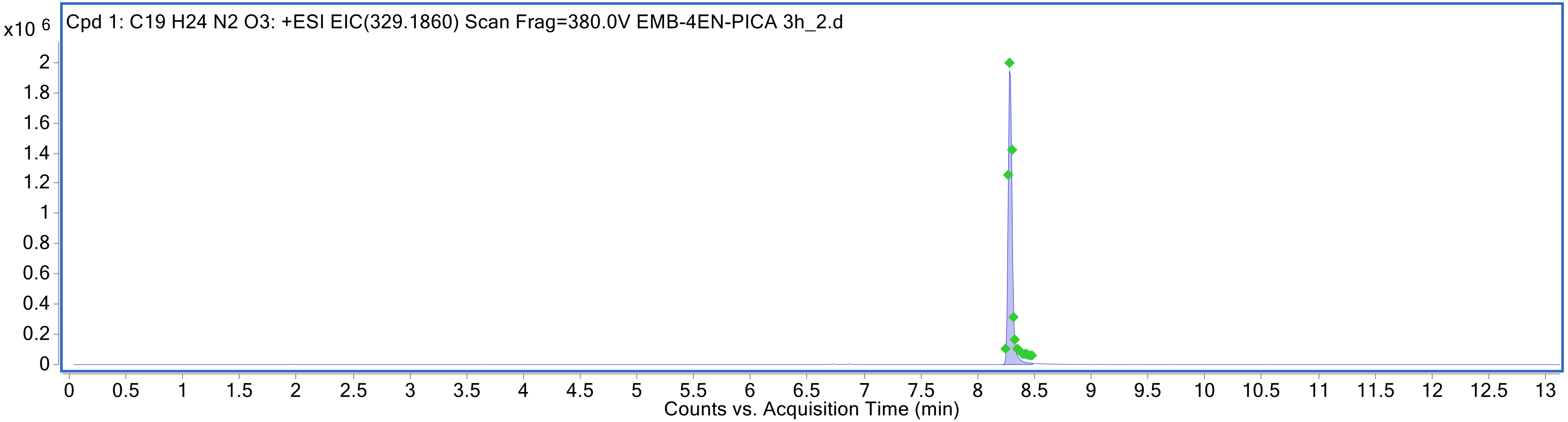

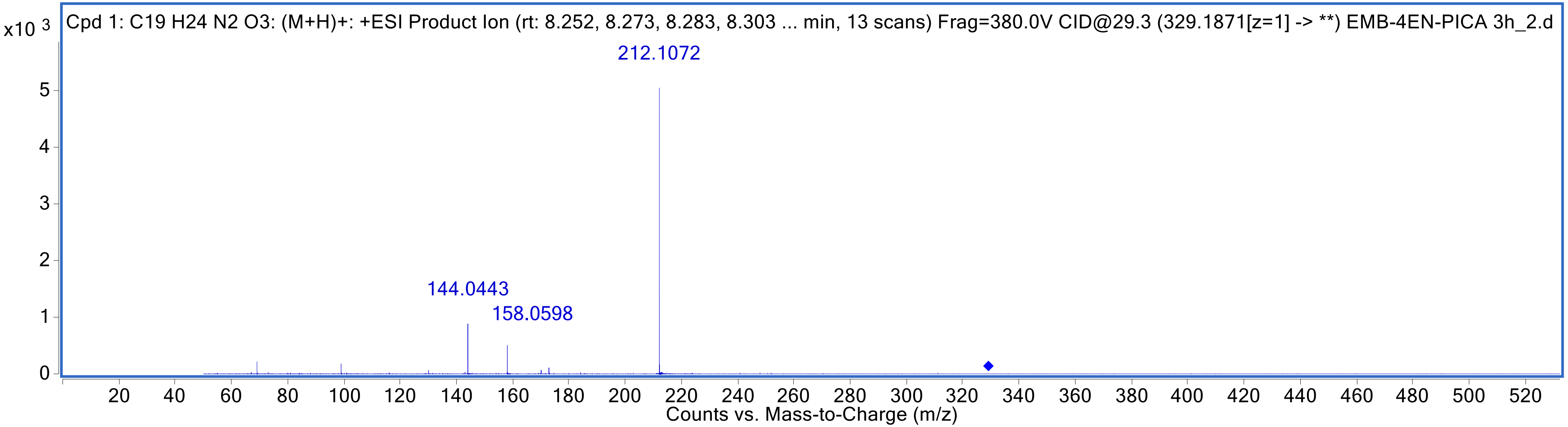

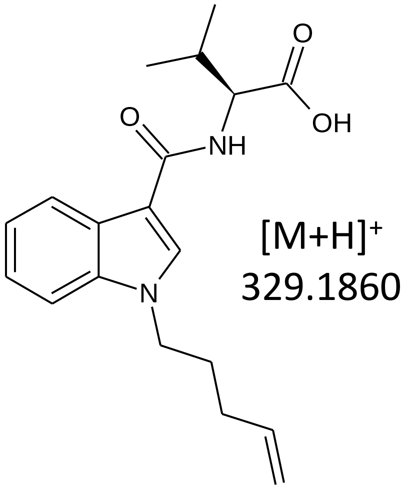

$[M+H]^+$
329.1860

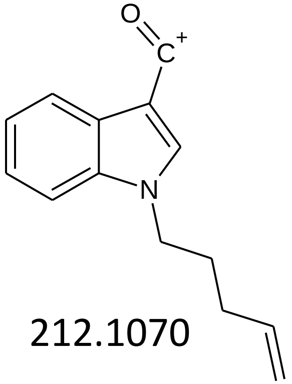

212.1070

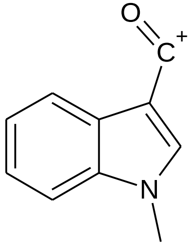

158.0600

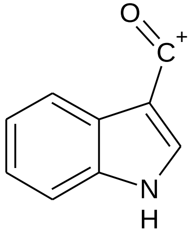

144.0444

C2, Ester hydrolysis + dihydrodiol formation, RT 4.42 min, *m/z* 363.1913

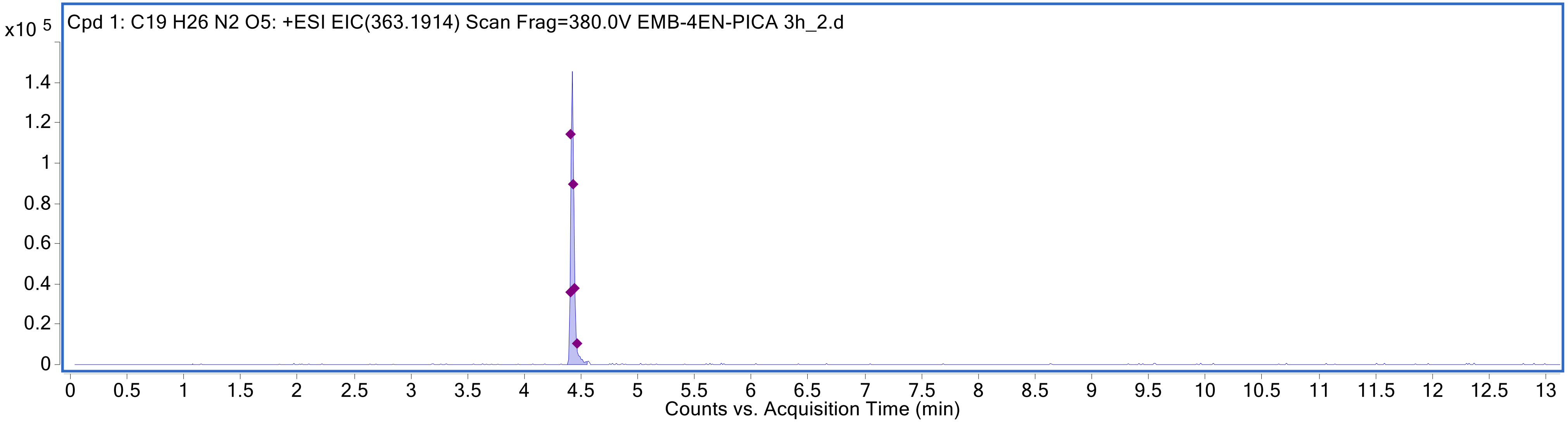

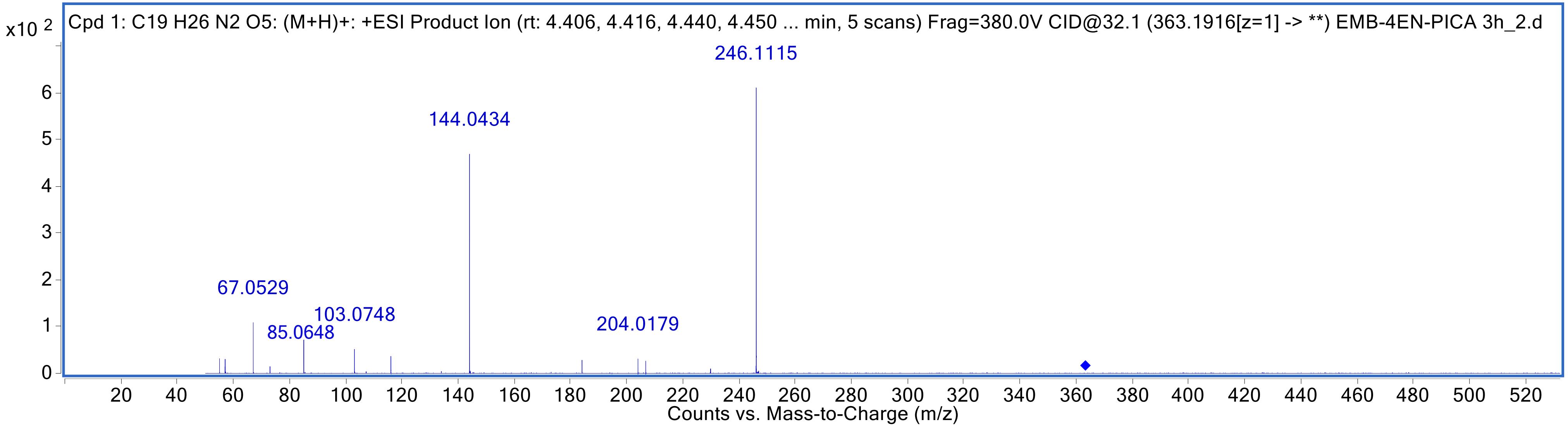

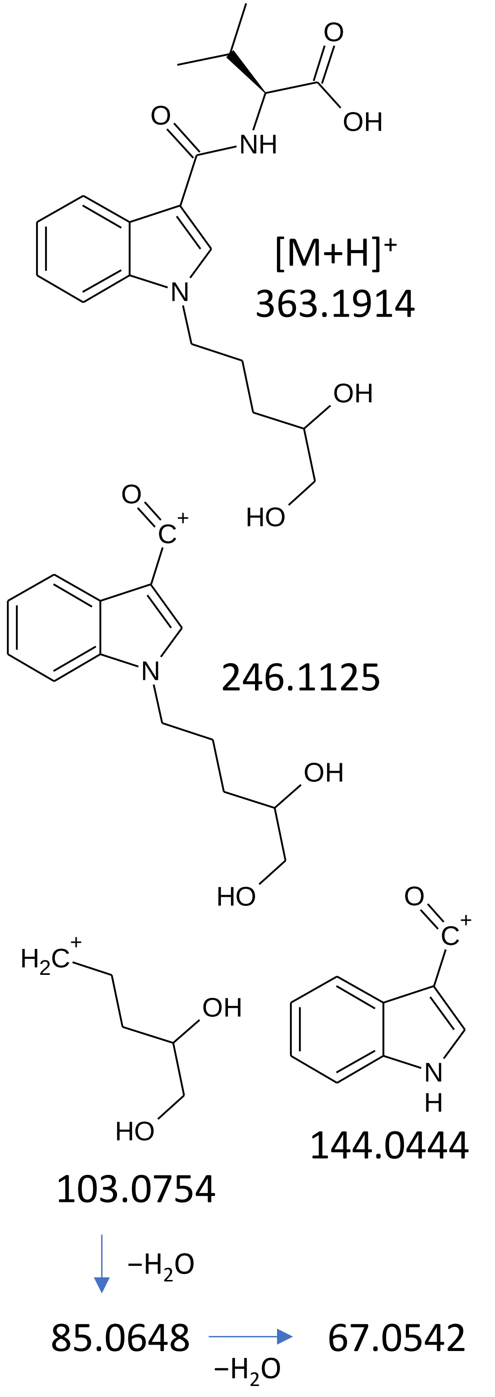

C3, Ester hydrolysis + glucuronidation, RT 6.86 min, *m/z* 505.2179

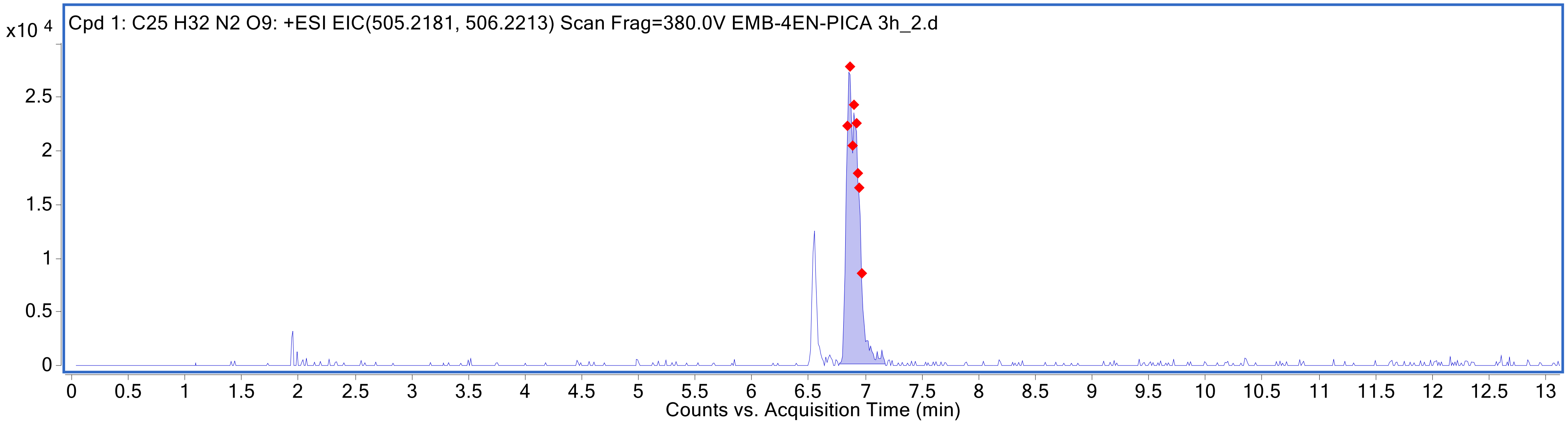

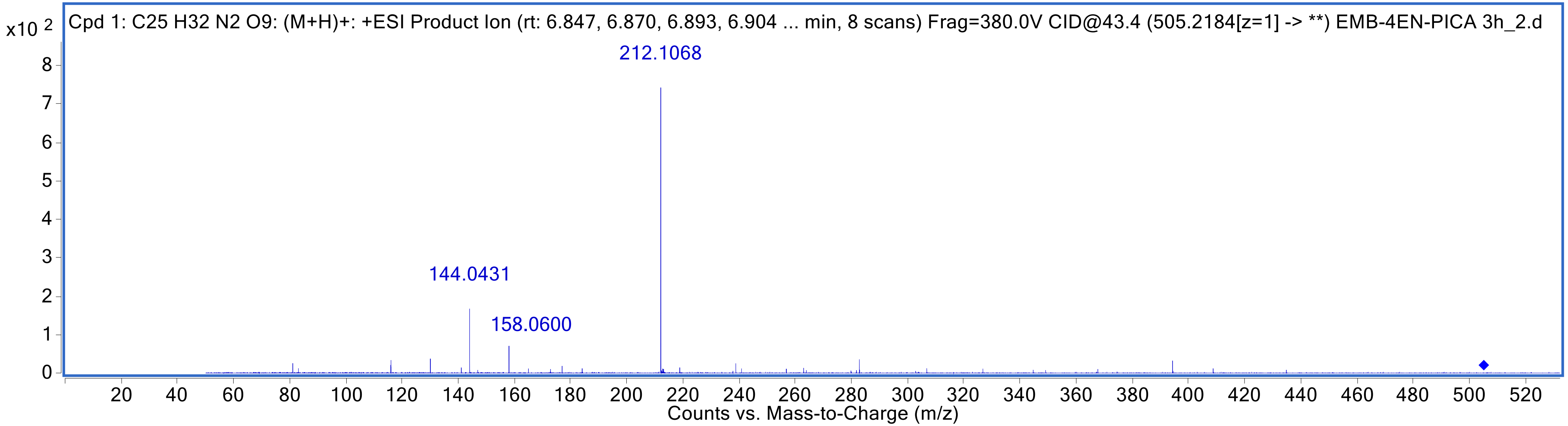

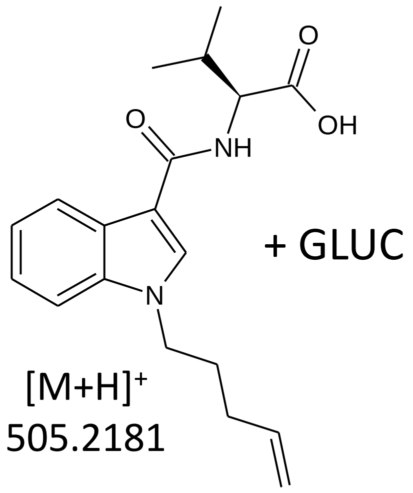

$[M+H]^+$
505.2181

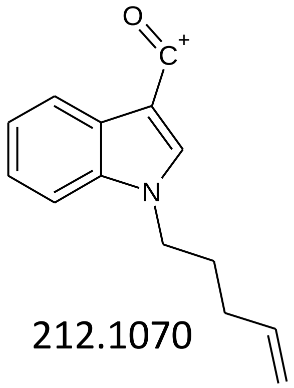

212.1070

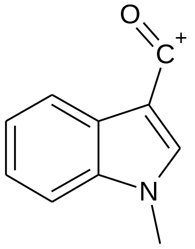

158.0600

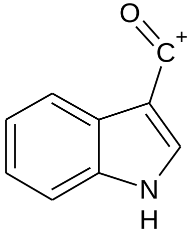

144.0444

C4, Secondary amide hydrolysis + mono-hydroxylation (pentenyl tail), RT 4.42 min, *m/z* 246.1121

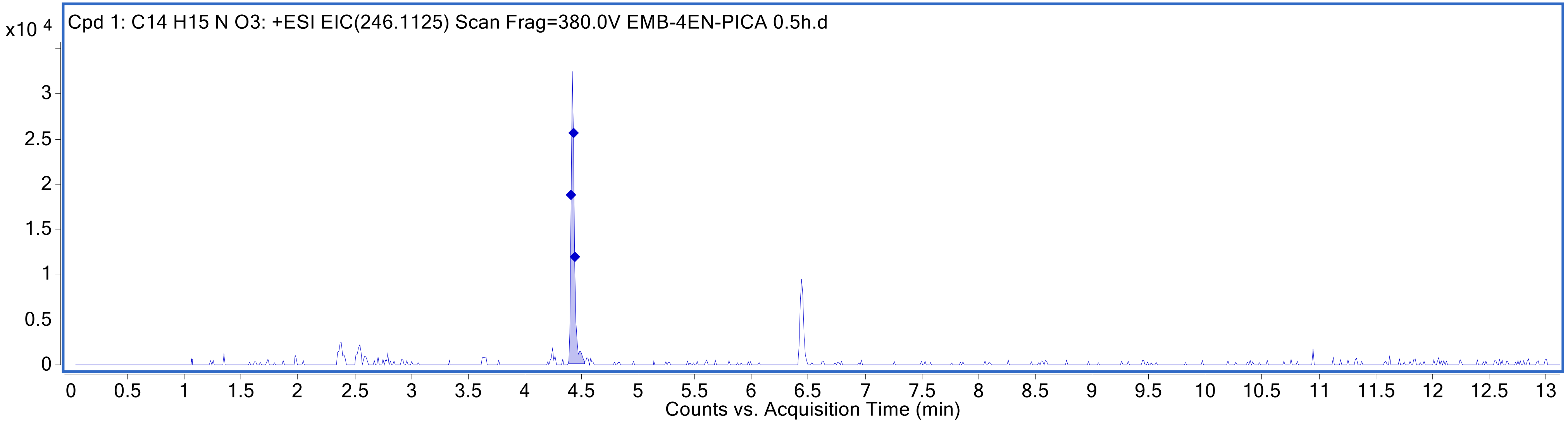

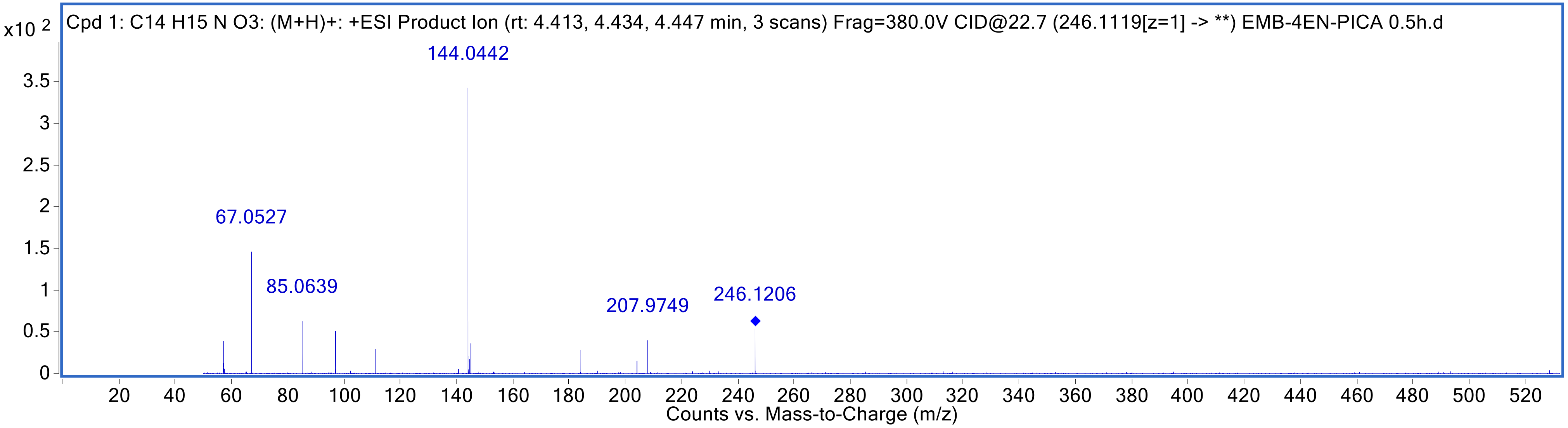

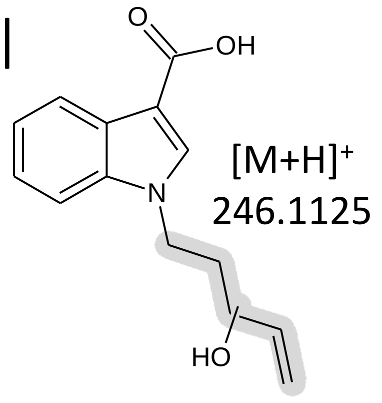

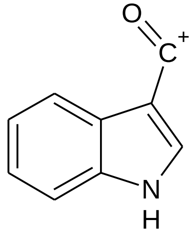

144.0444

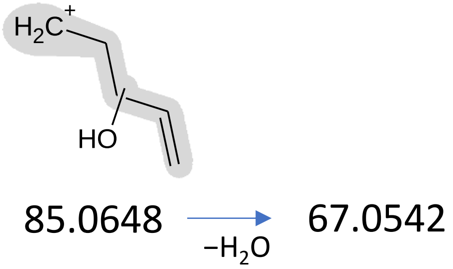

C5, Head group–linker cleavage, RT 6.41 min, *m/z* 229.1330

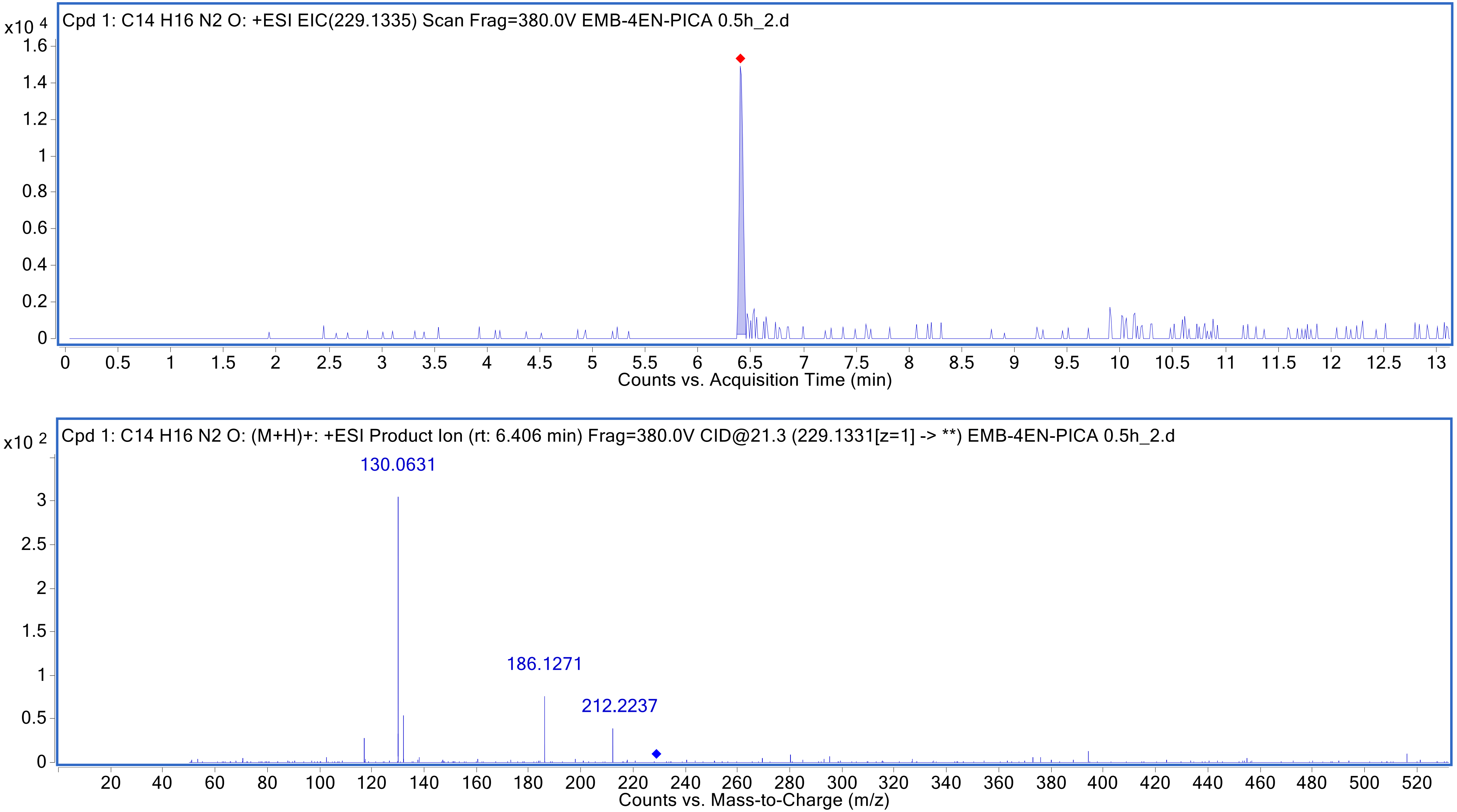

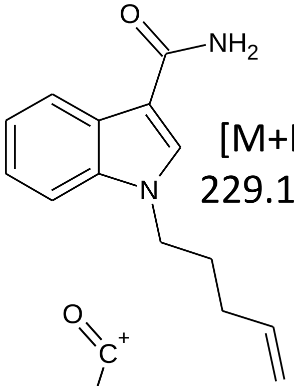

[M+H]+
229.1335

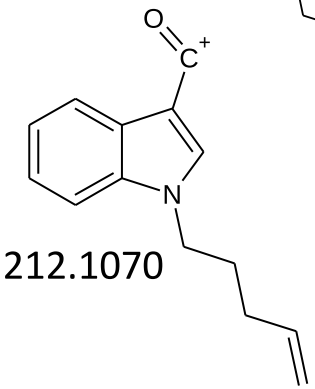

212.1070

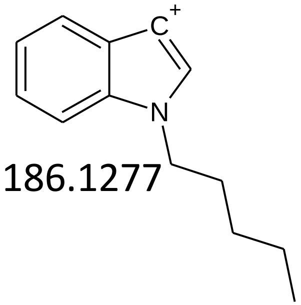

186.1277

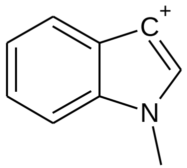

130.0651

C6, Ester hydrolysis + mono-hydroxylation (pentenyl tail), RT 5.87 min, *m/z* 345.1806

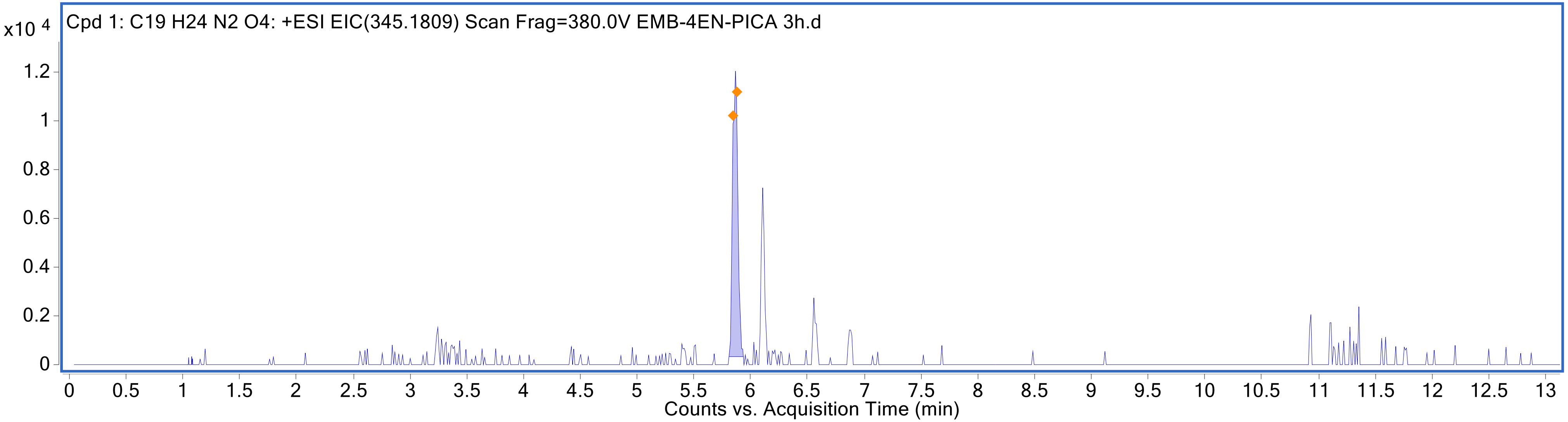

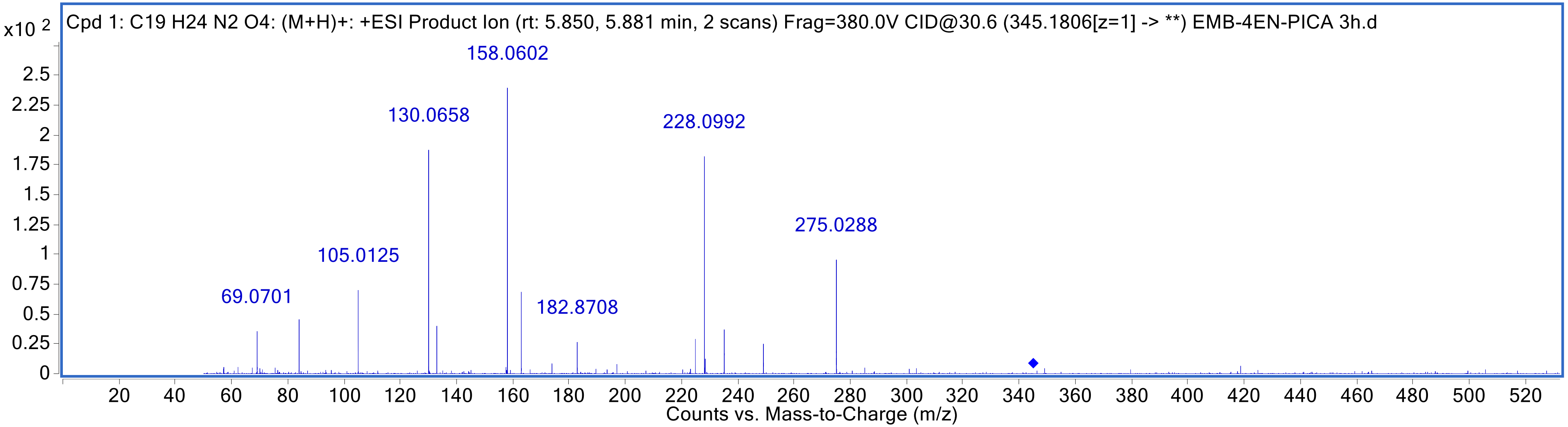

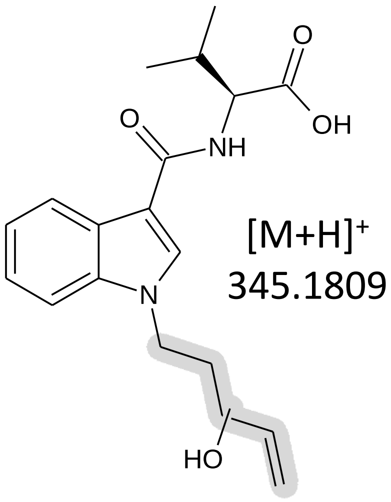

[M+H]+
345.1809

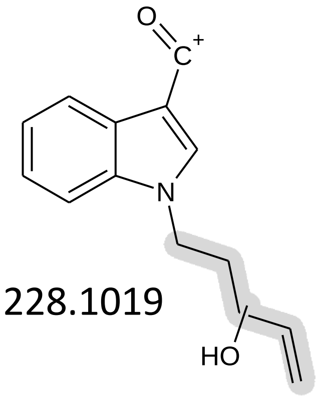

228.1019

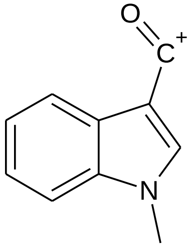

158.0600

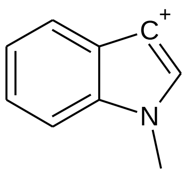

130.0651

C7, Dihydrodiol formation, RT 6.44 min, *m/z* 391.2225

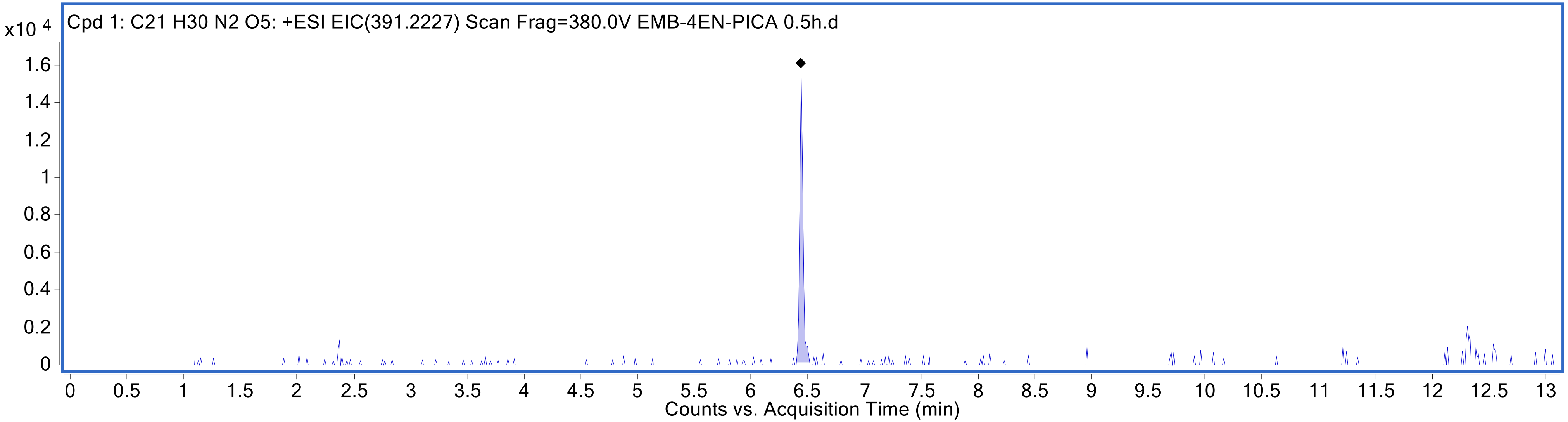

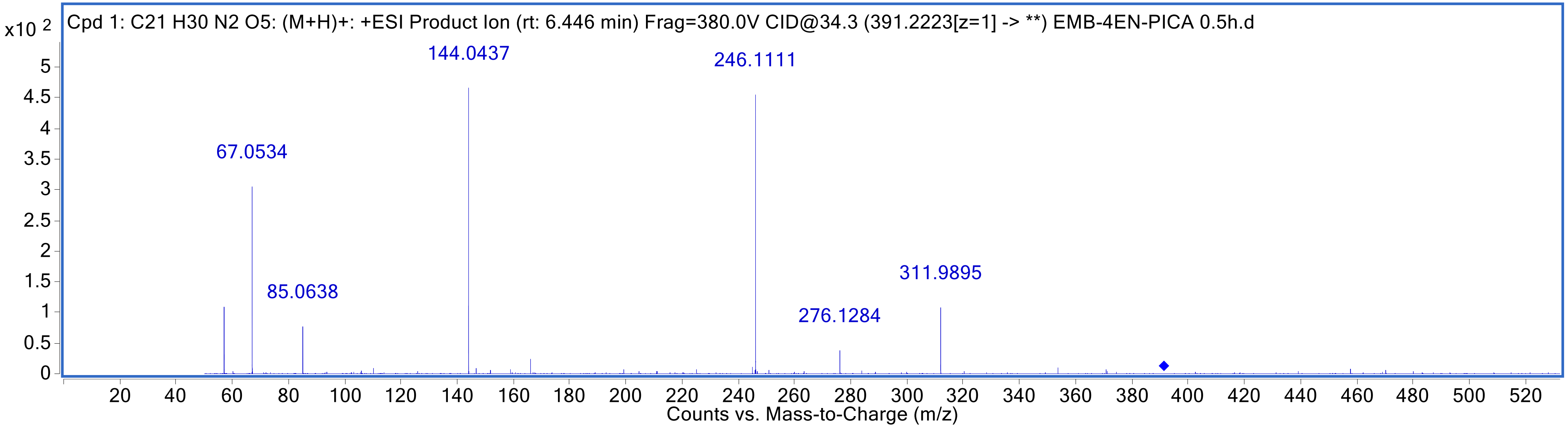

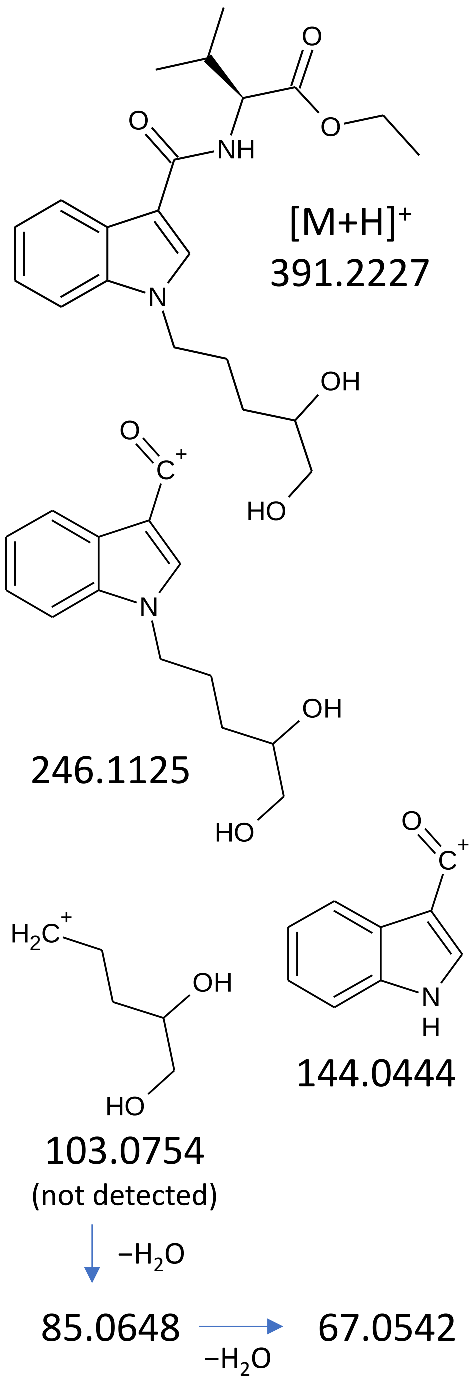

Dihydrodiol reference standard, RT 6.44 min, *m/z* 391.2258

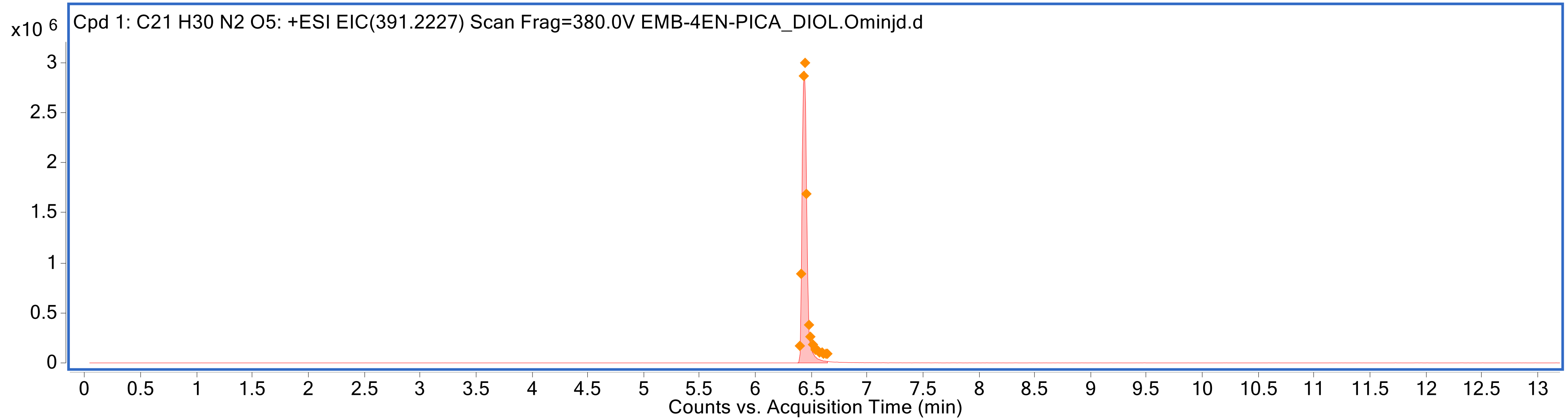

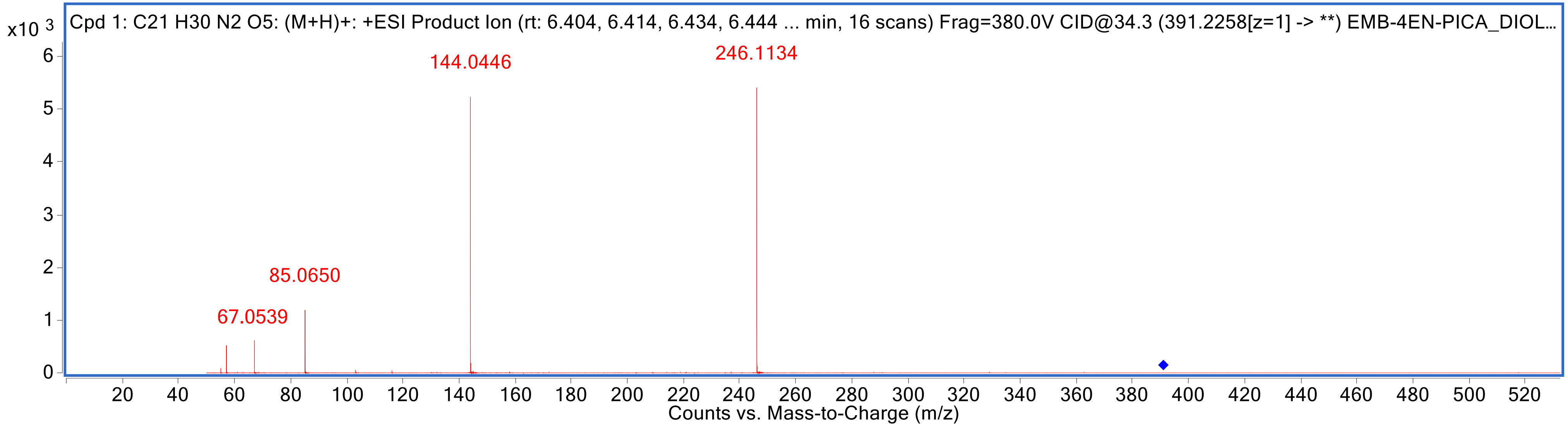

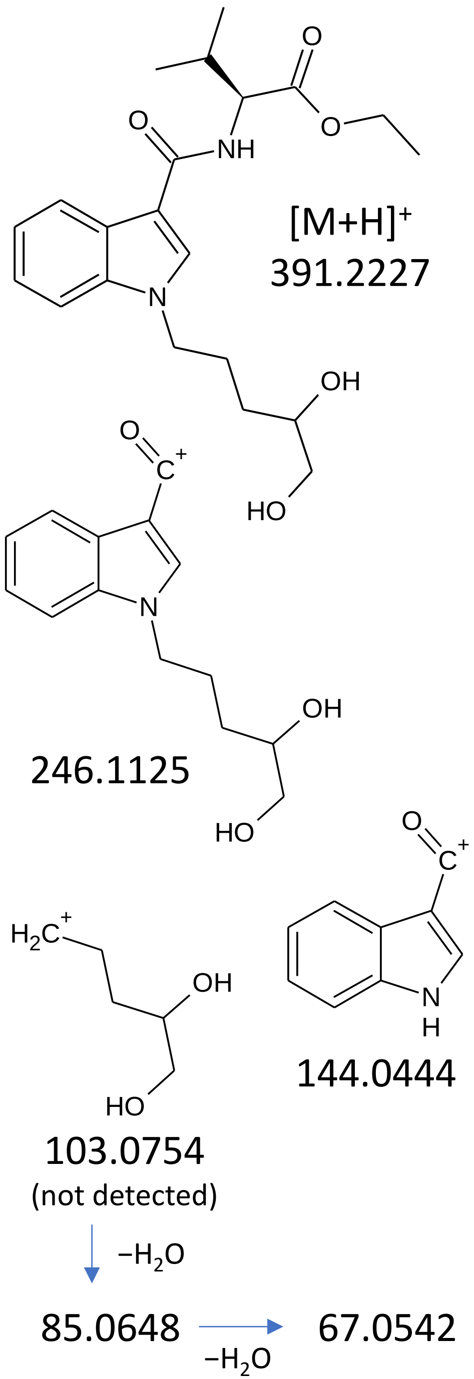

C8, Ester hydrolysis + glucuronidation, RT 6.55 min, *m/z* 505.2193

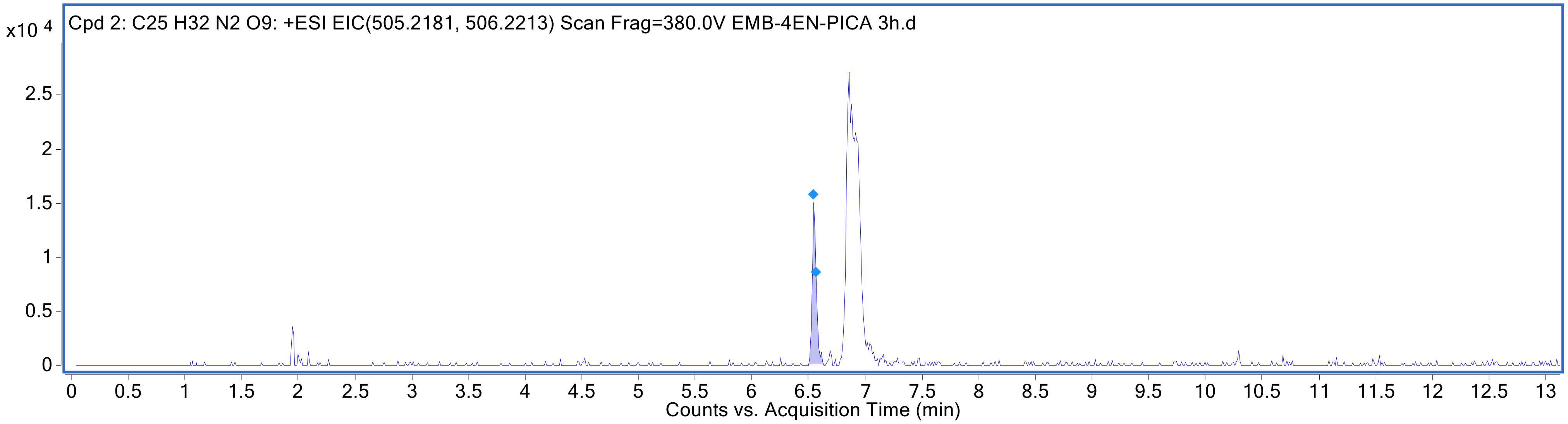

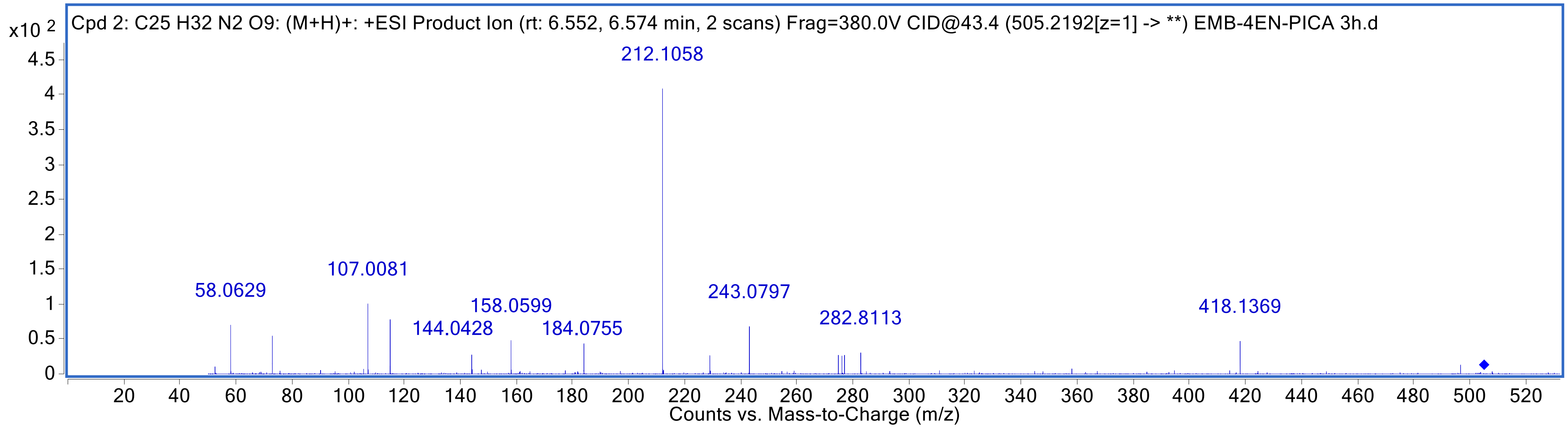

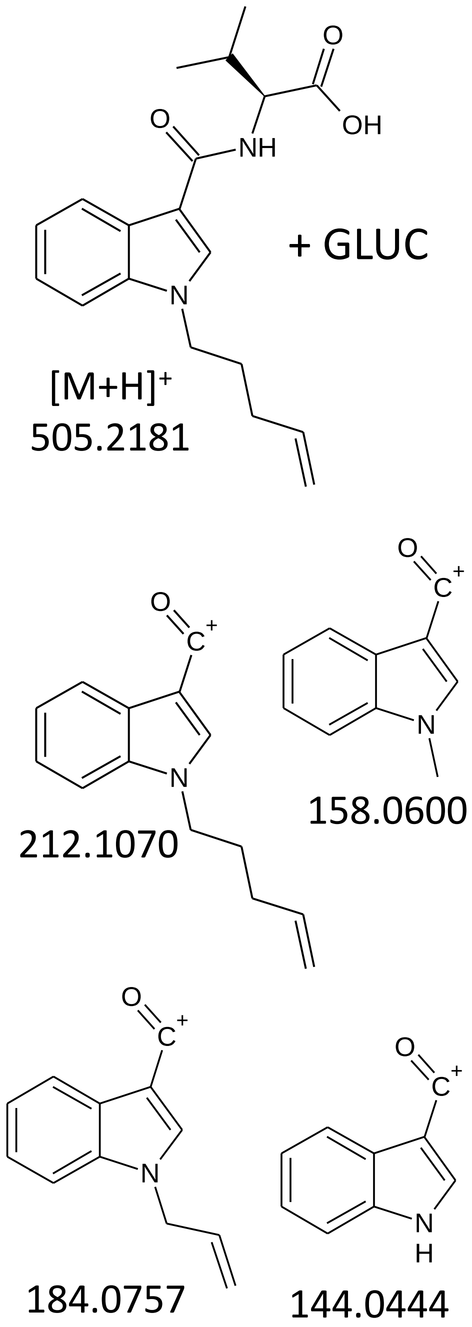

EMB-4en-PINACA

Metabolism

EMB-4en-PINACA, RT 11.61 min, *m/z* 358.2146

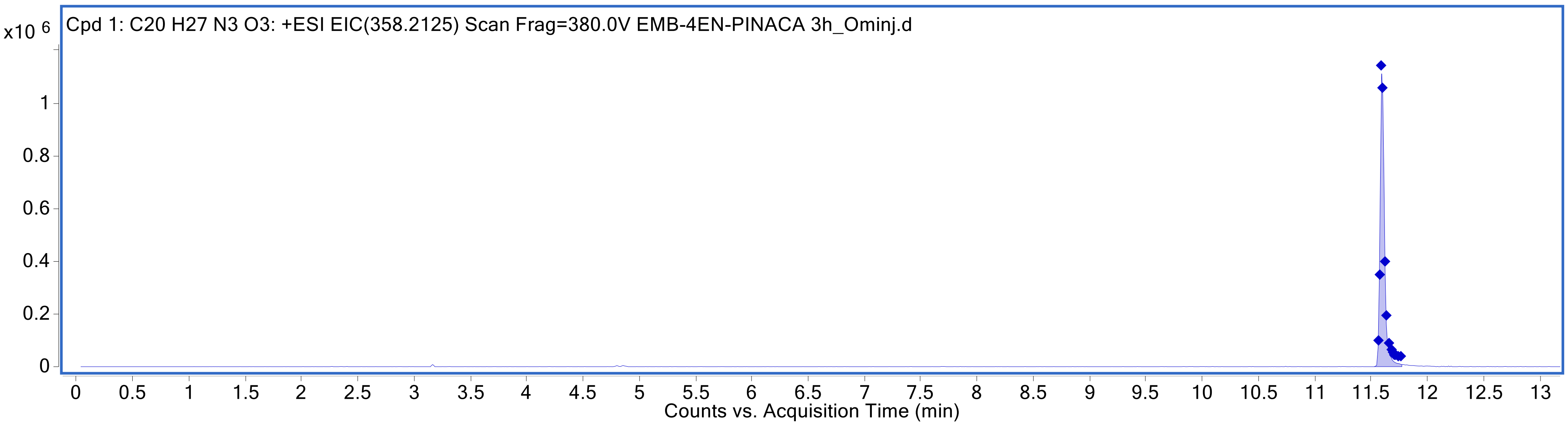

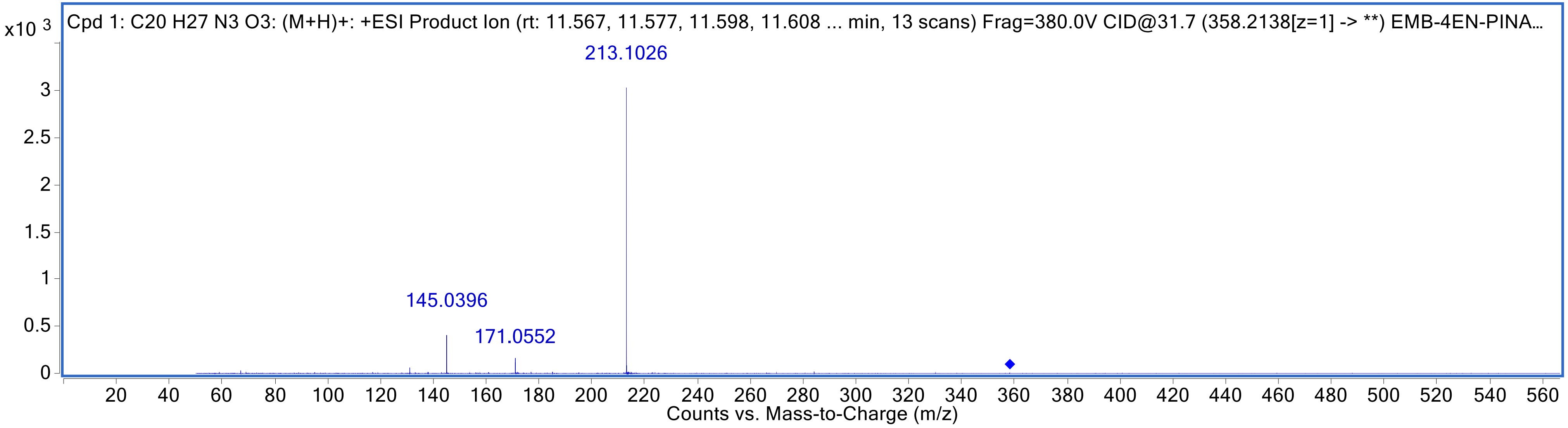

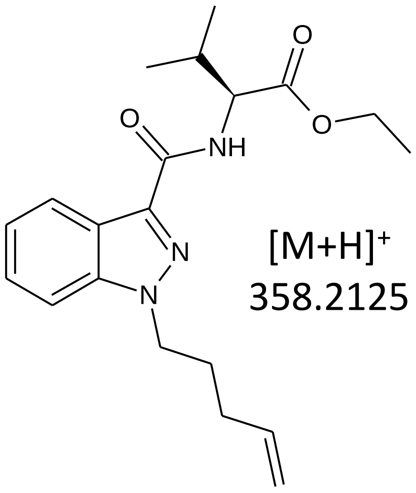

$[M+H]^+$
358.2125

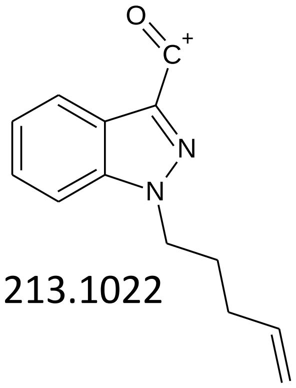

213.1022

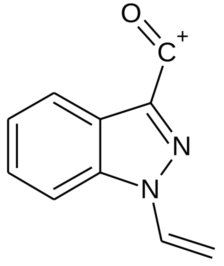

171.0553

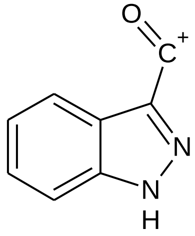

145.0396

D1, Ester hydrolysis, RT 8.84 min, *m/z* 330.1834

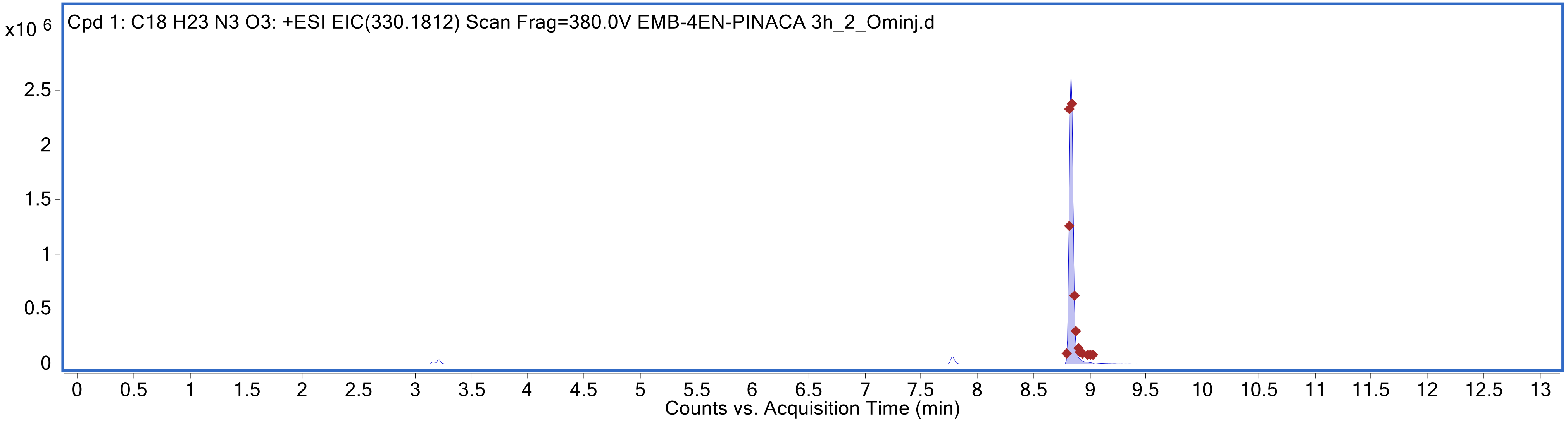

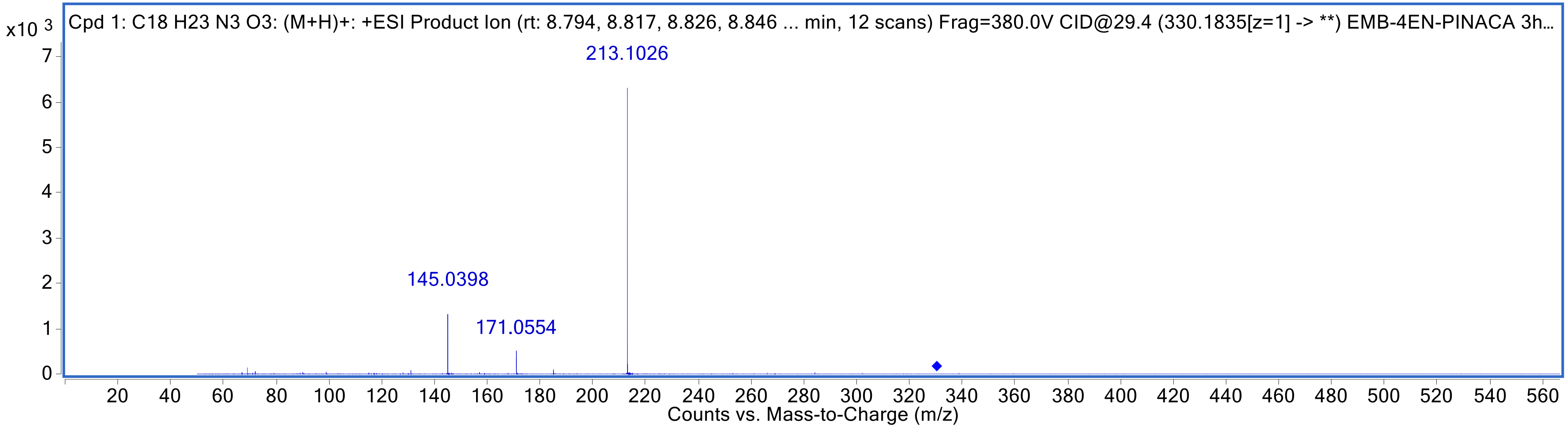

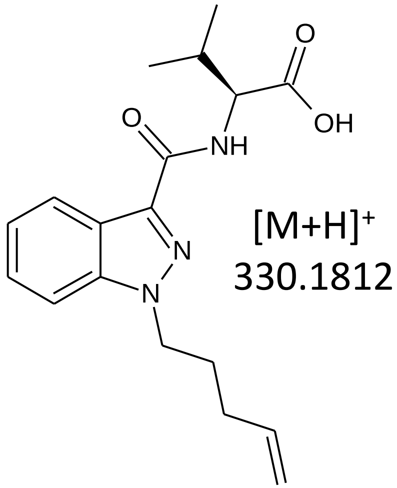

$[M+H]^+$
330.1812

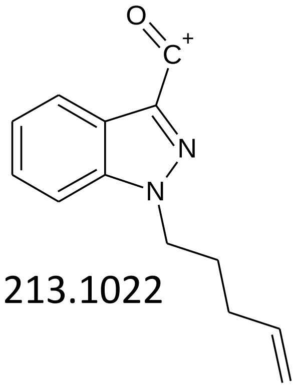

213.1022

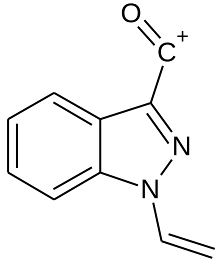

171.0553

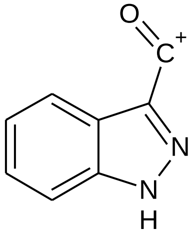

145.0396

D2, Ester hydrolysis + dihydrodiol formation, RT 4.63 min, *m/z* 364.1875

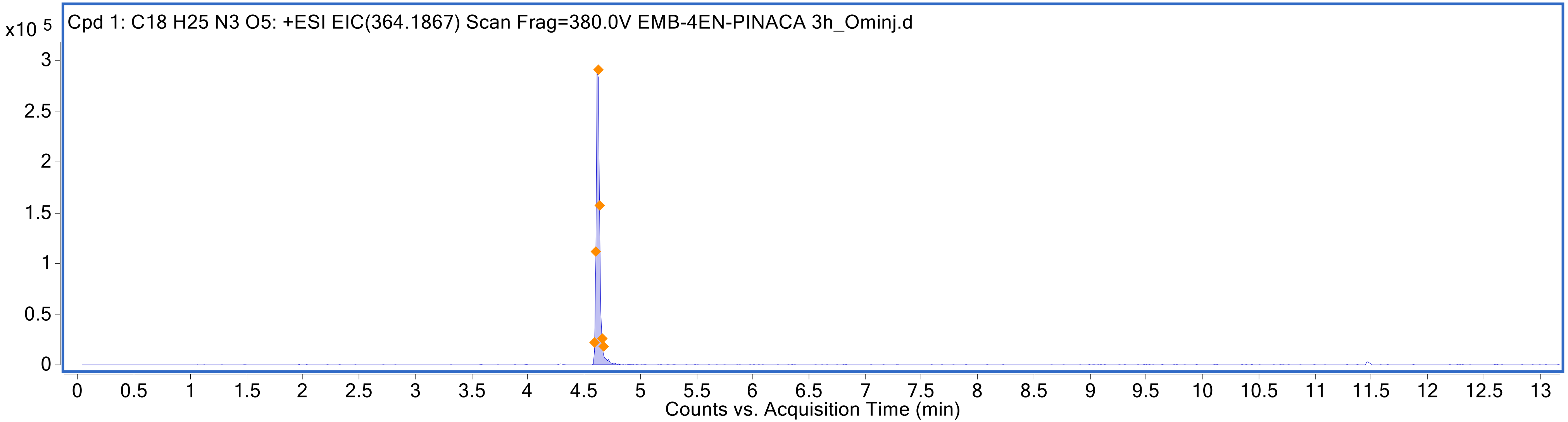

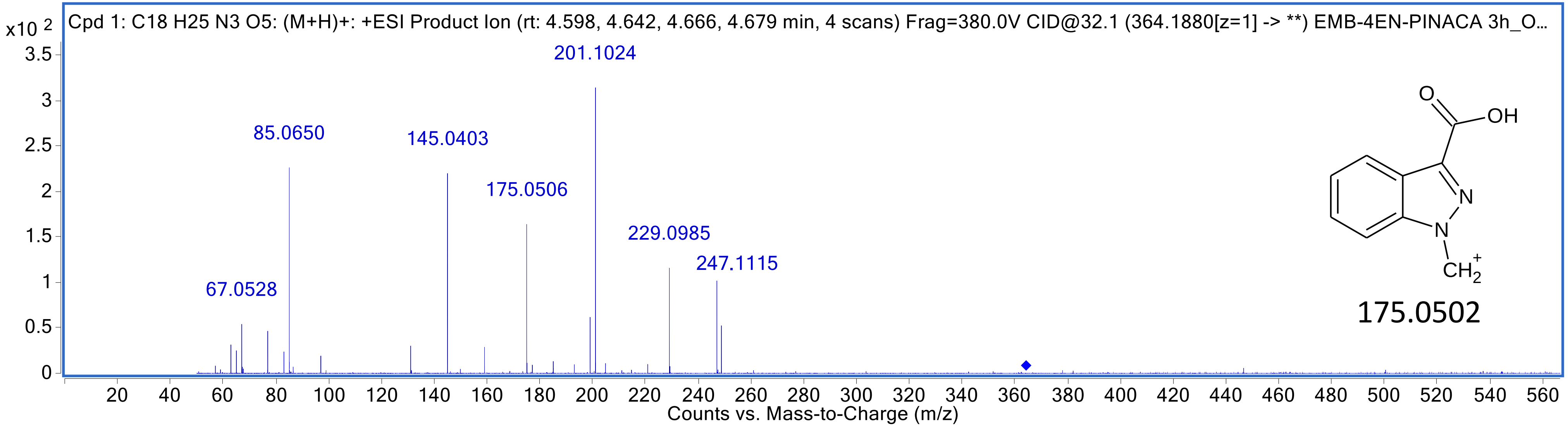

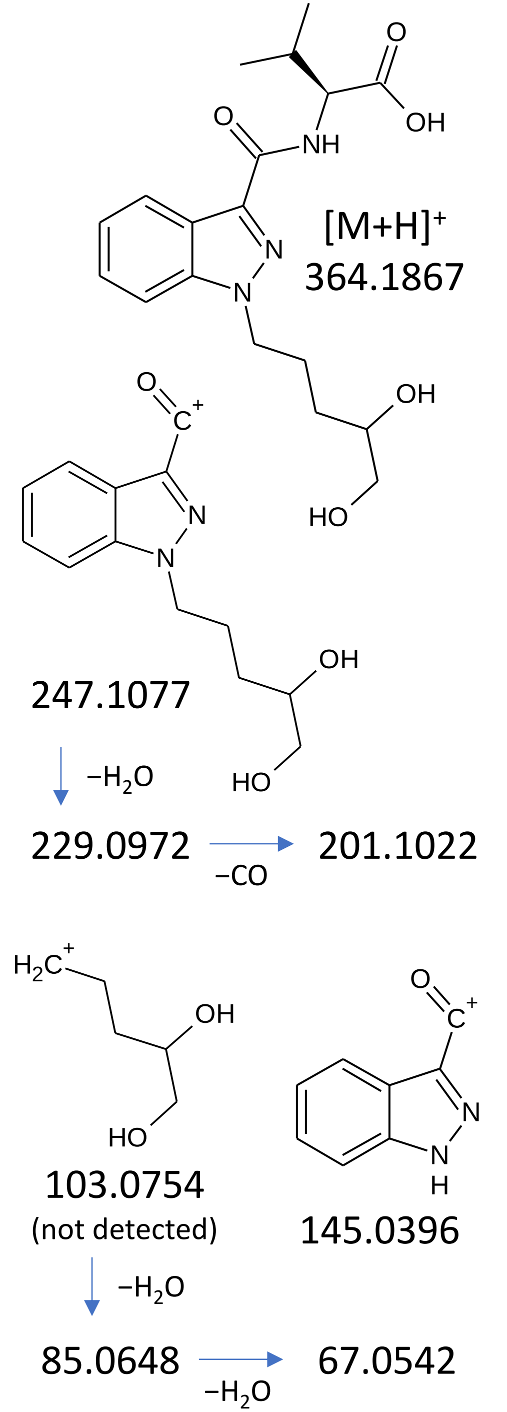

D3, Ester hydrolysis + glucuronidation, RT 7.14 min, *m/z* 506.2134

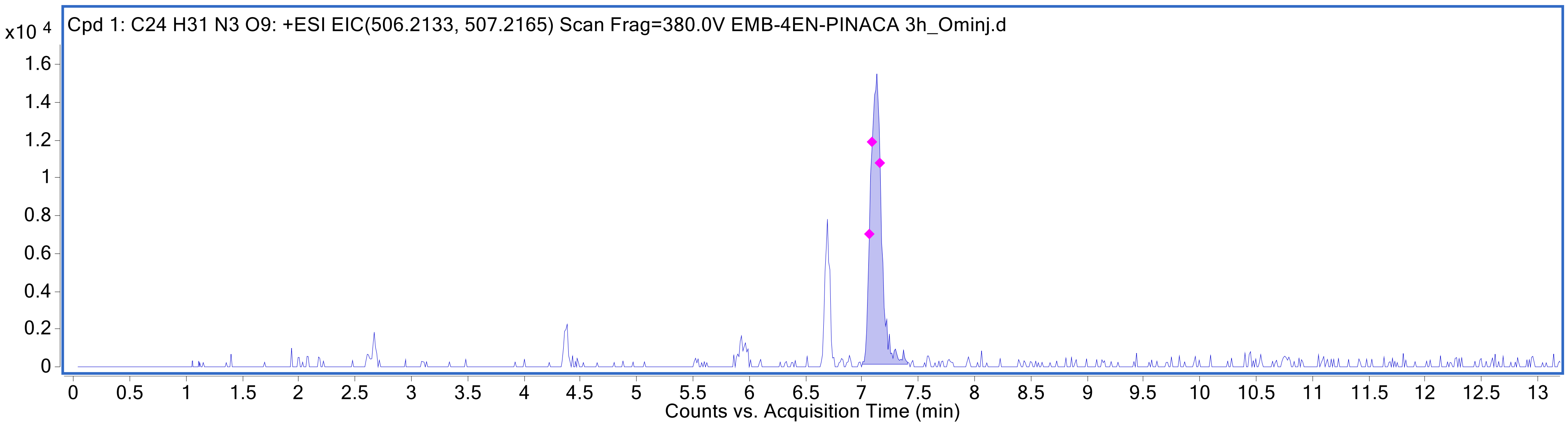

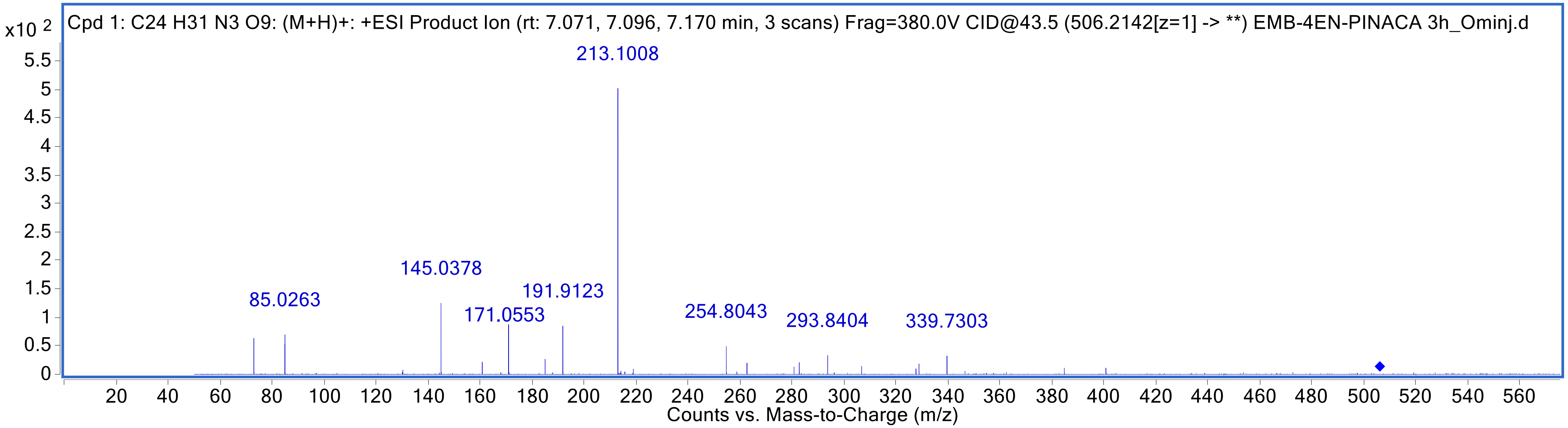

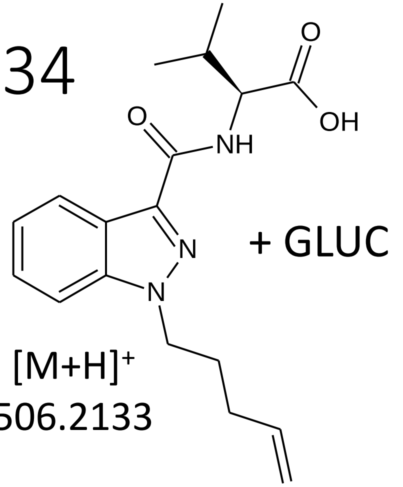

$[M+H]^+$
506.2133

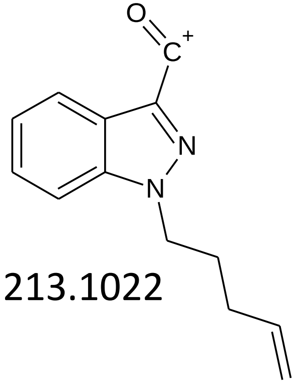

213.1022

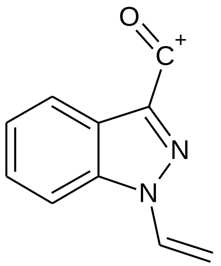

171.0553

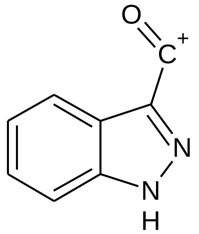

145.0396

D4, Ester hydrolysis + dihydrodiol formation + glucuronidation, RT 3.73 min, *m/z* 540.2190

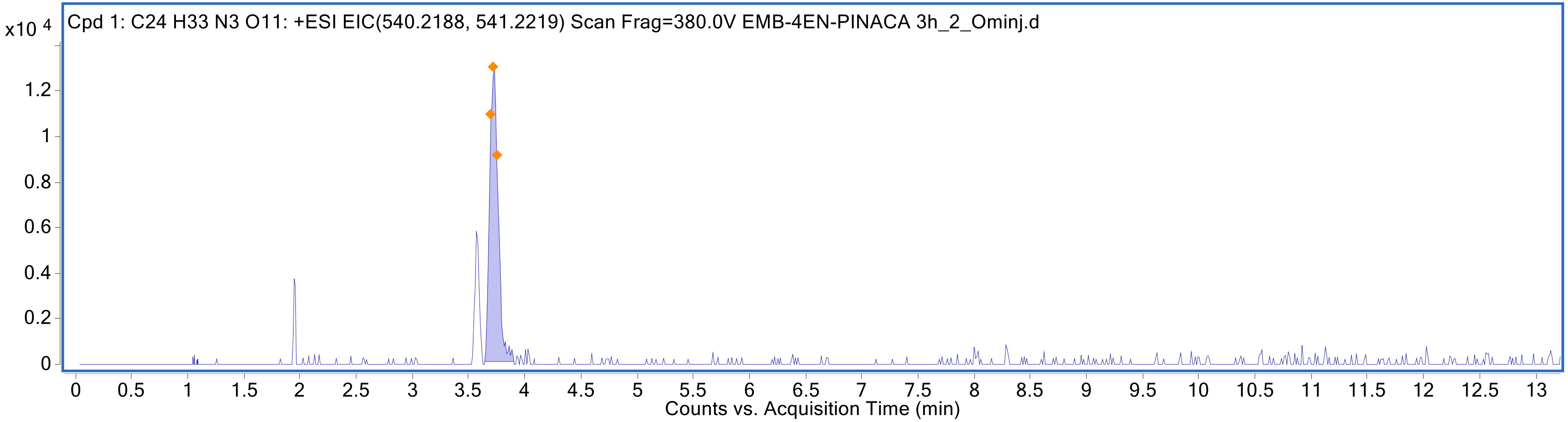

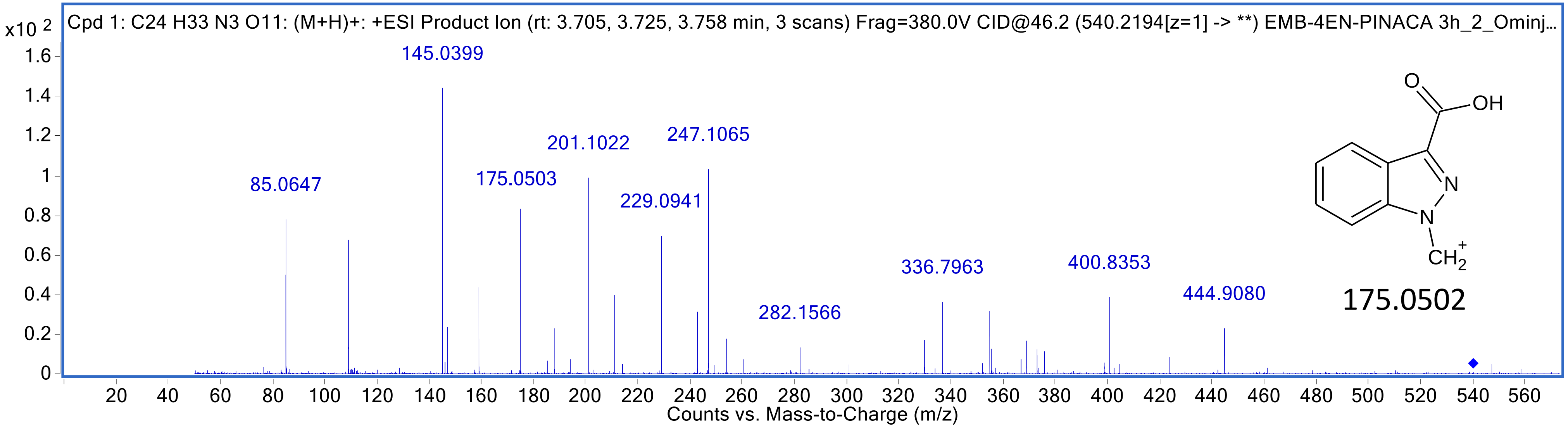

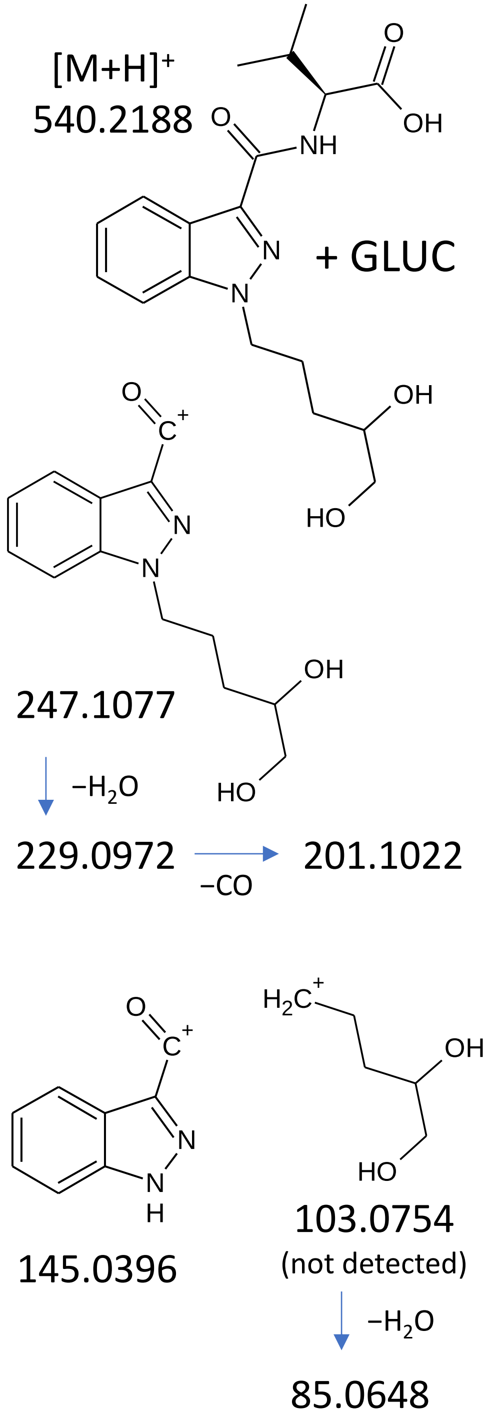

D5, Ester hydrolysis + di-hydroxylation (pentenyl tail), RT 5.12 min, *m/z* 362.1714

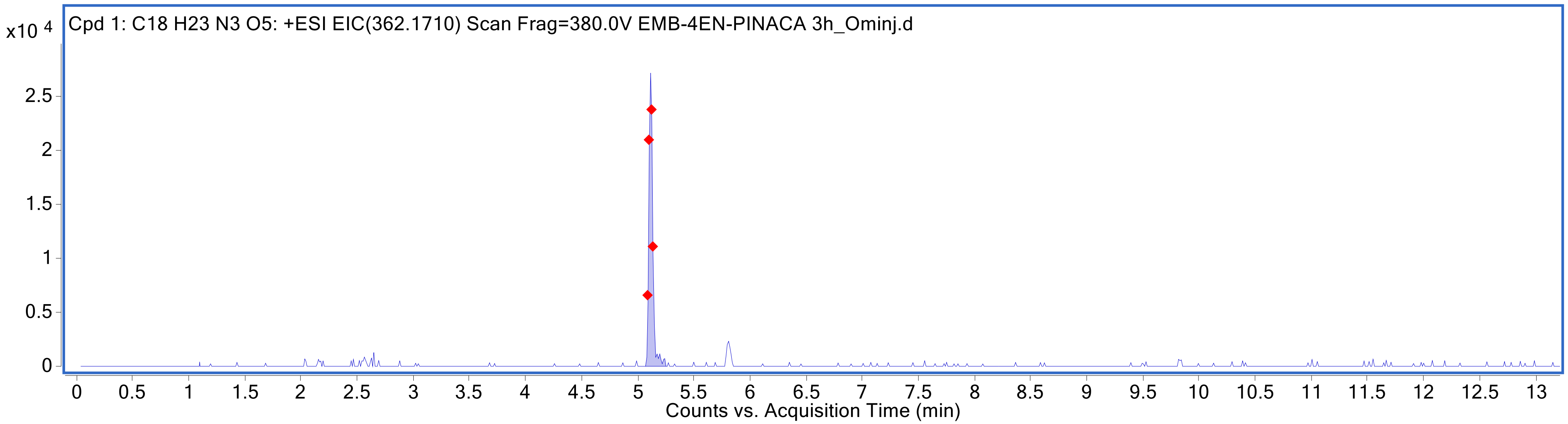

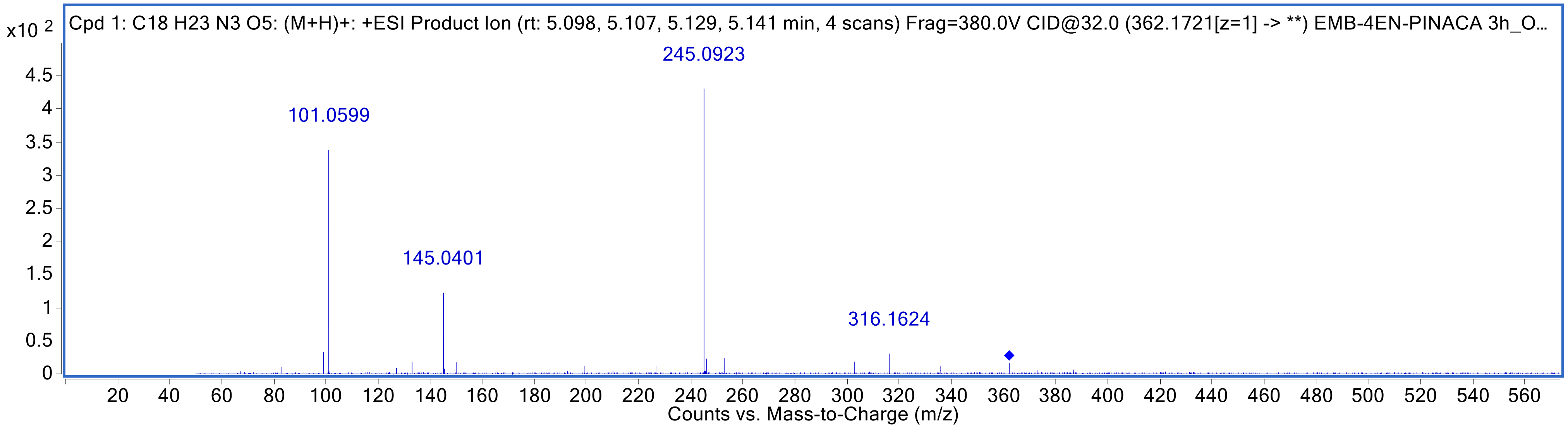

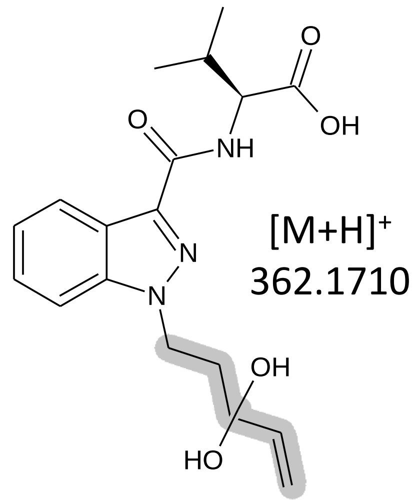

$[M+H]^+$
362.1710

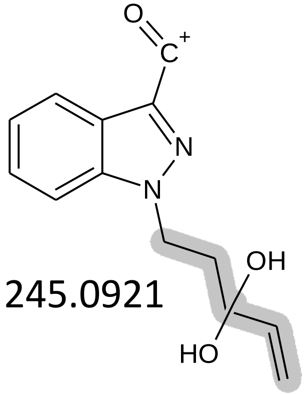

245.0921

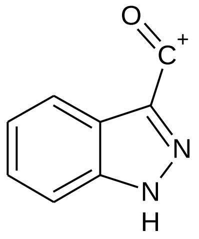

145.0396

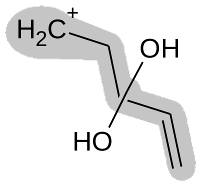

101.0597

D6, Ester hydrolysis + mono-hydroxylation (pentenyl tail), RT 6.11 min, *m/z* 346.1764

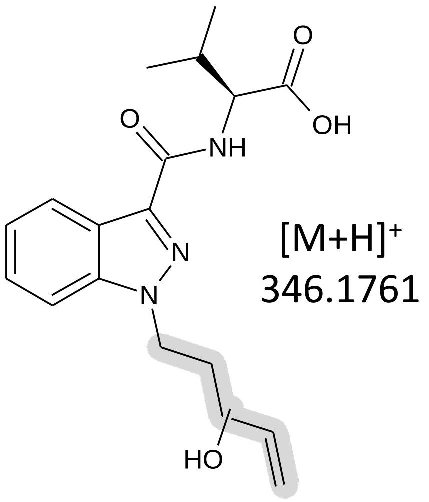

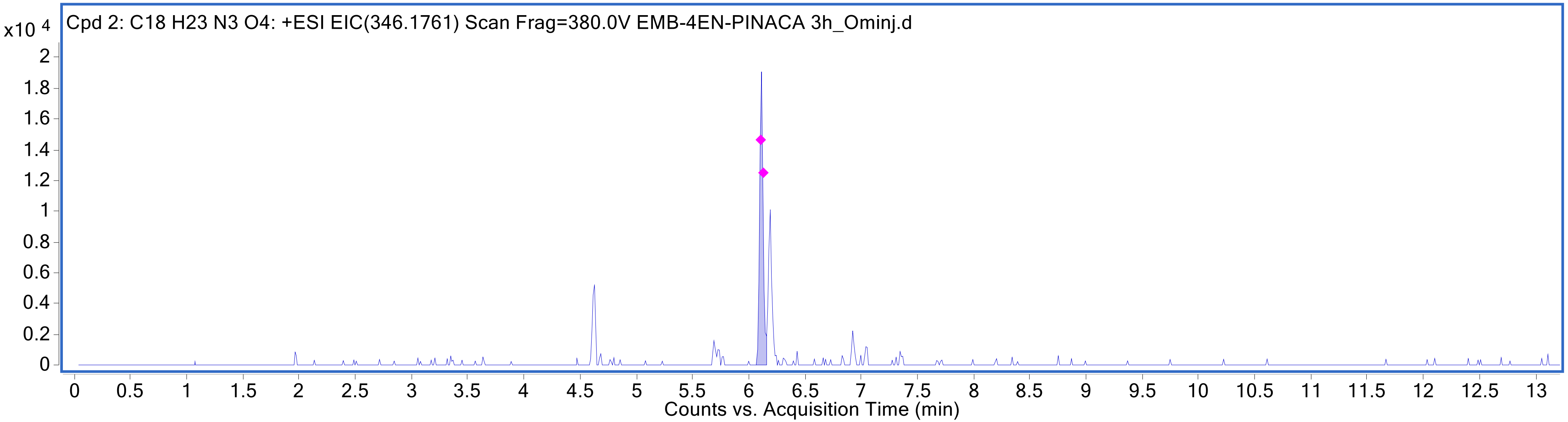

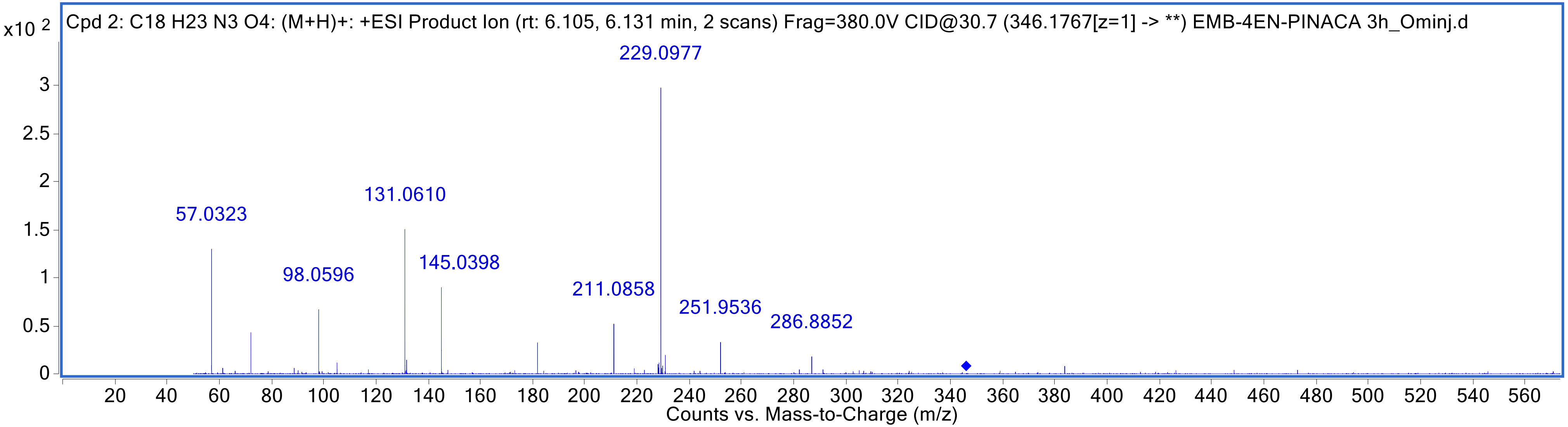

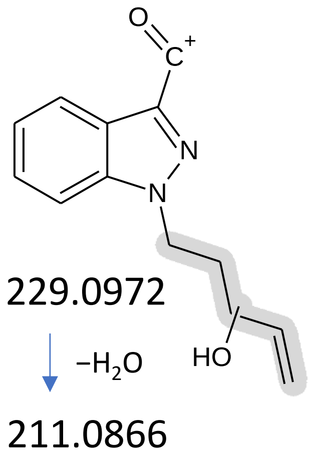

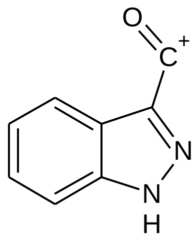

Dihydrodiol reference standard, RT 6.73 min, *m/z* 392.2215

(not detected in HHep incubations)

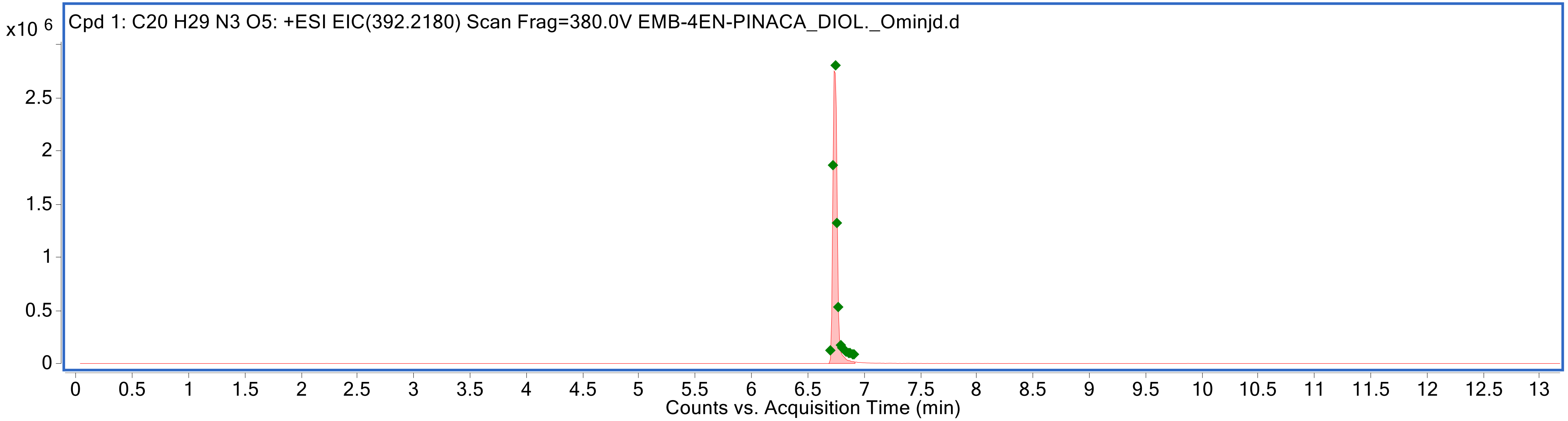

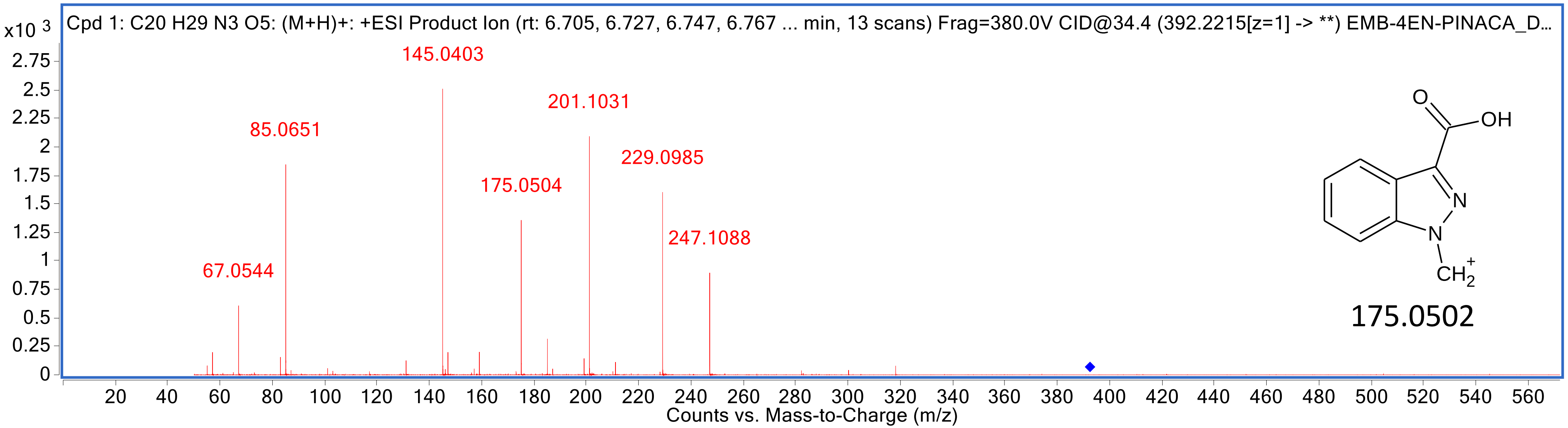

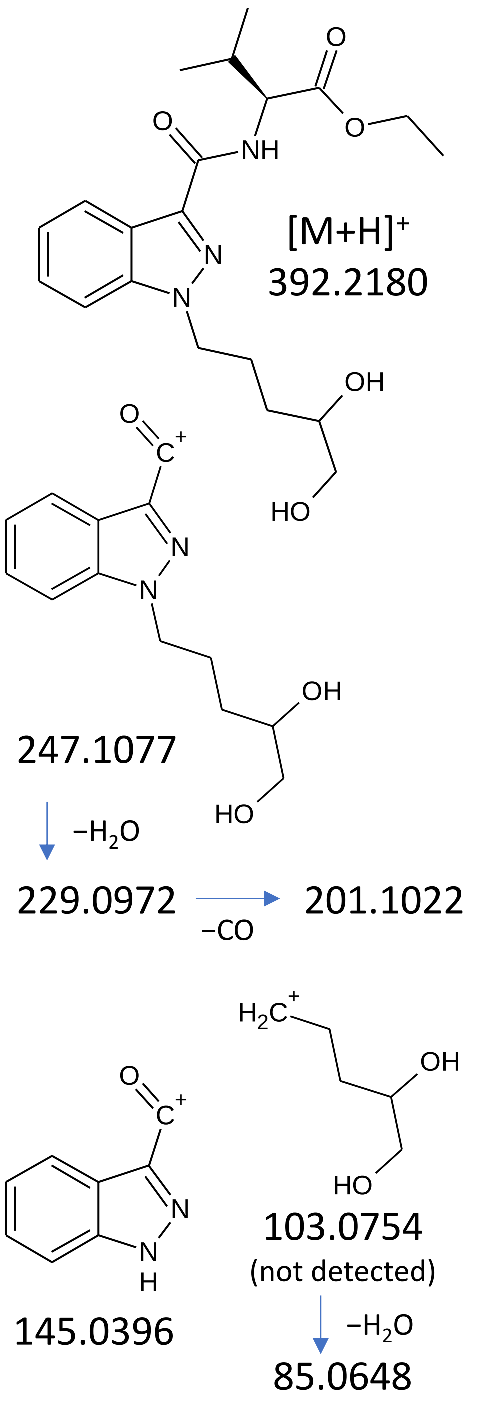

D7, Ester hydrolysis + mono-hydroxylation (pentenyl tail), RT 6.19 min, *m/z* 346.1770

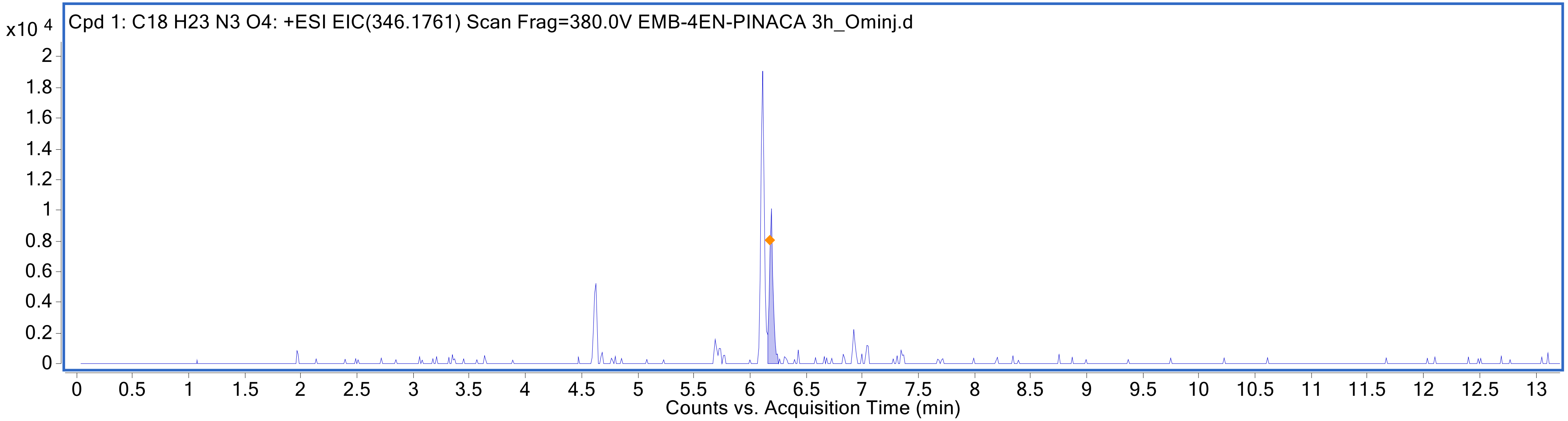

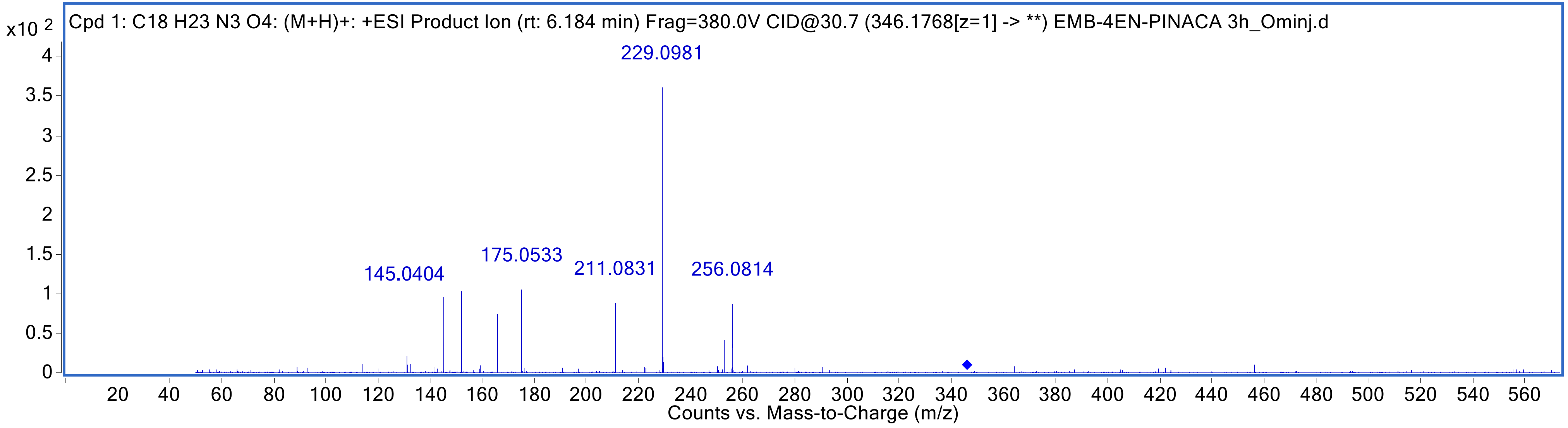

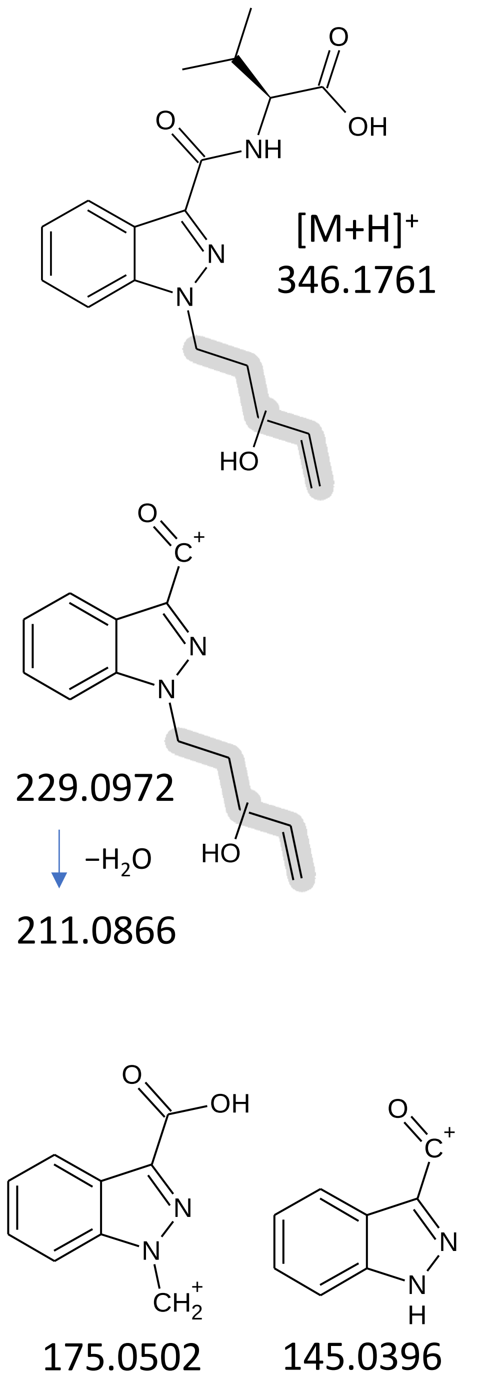

MDMB-4en-PICA

Metabolism

MDMB-4en-PICA, RT 12.04 min, *m/z* 357.2209

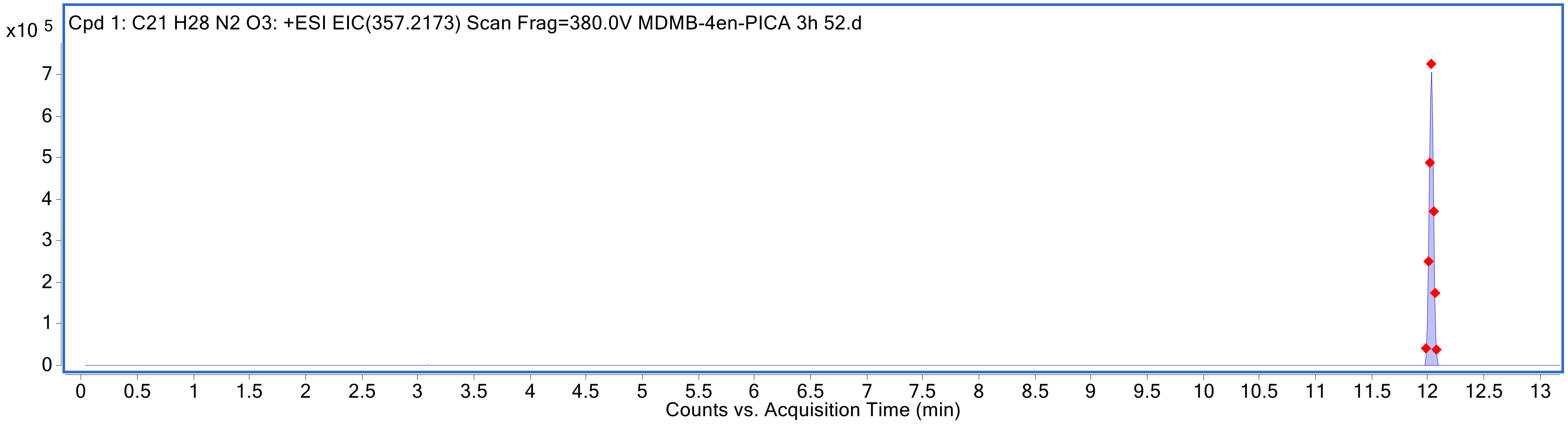

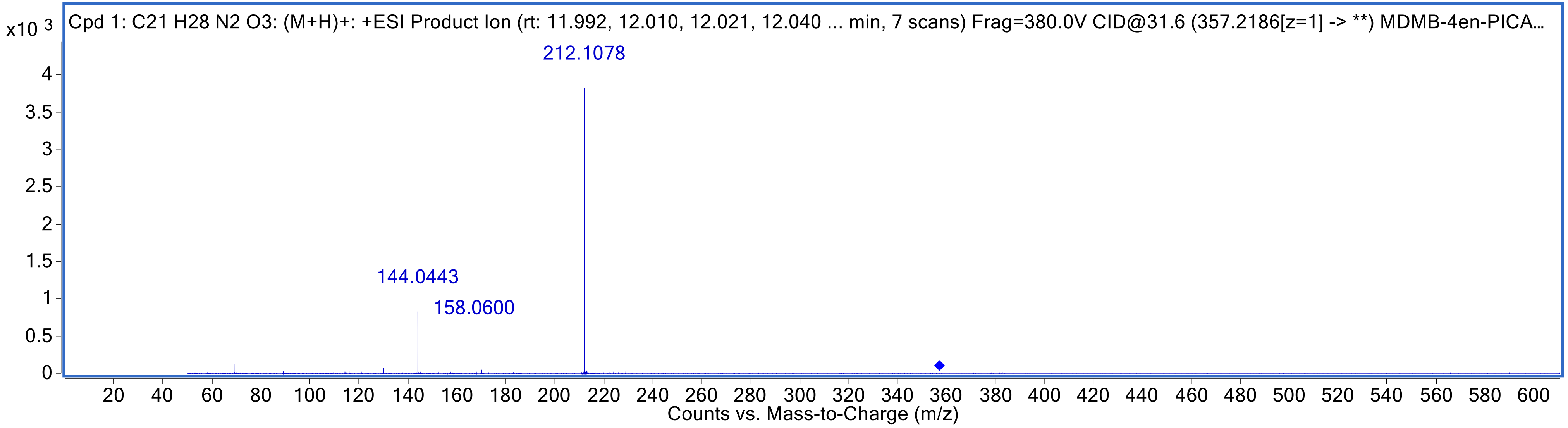

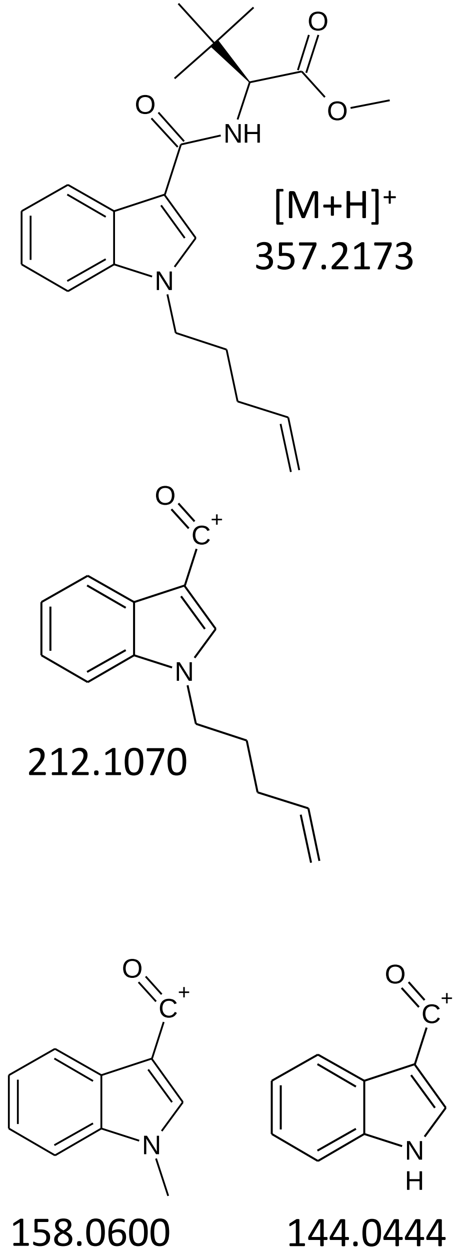

E1, Ester hydrolysis, RT 9.94 min, *m/z* 343.2026

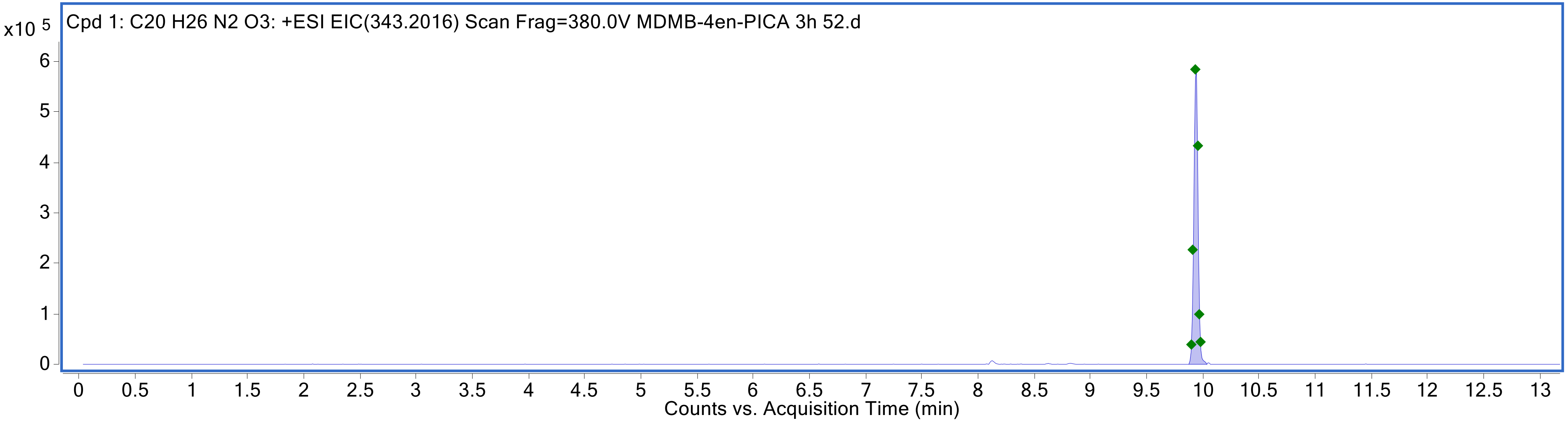

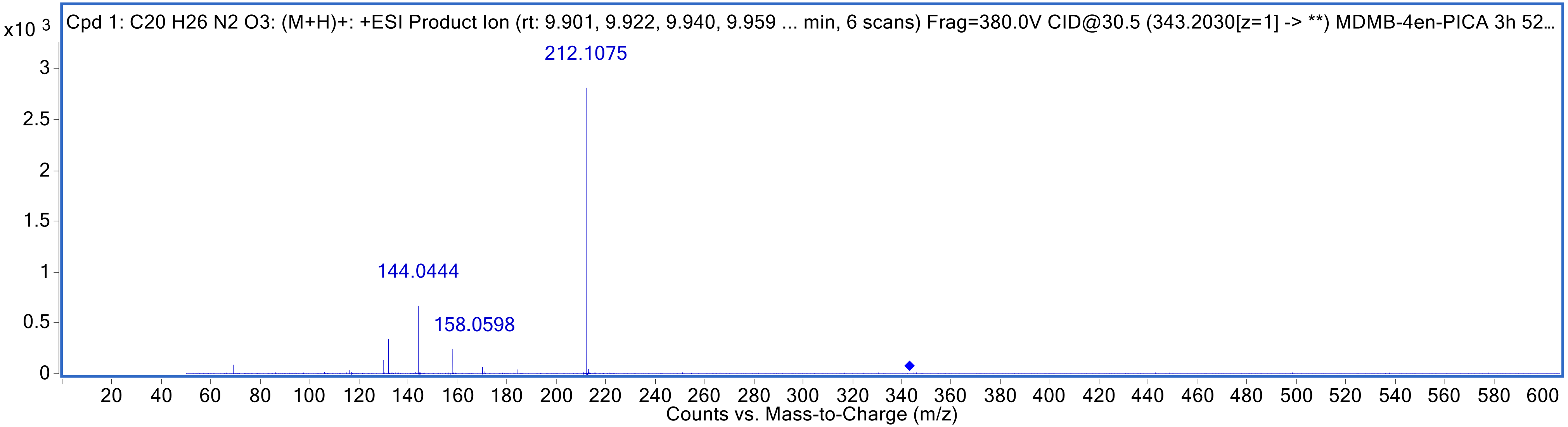

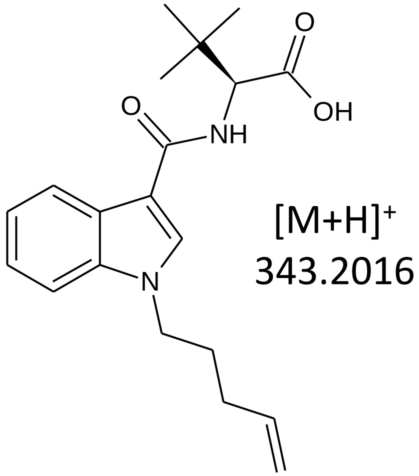

$[M+H]^+$
343.2016

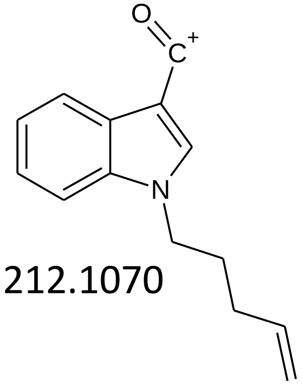

212.1070

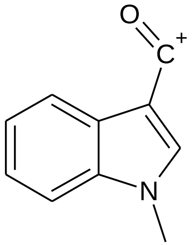

158.0600

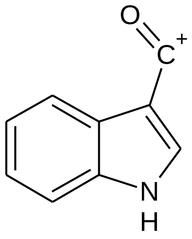

144.0444

E2, Dihydrodiol formation, RT 7.05 min, *m/z* 391.2238

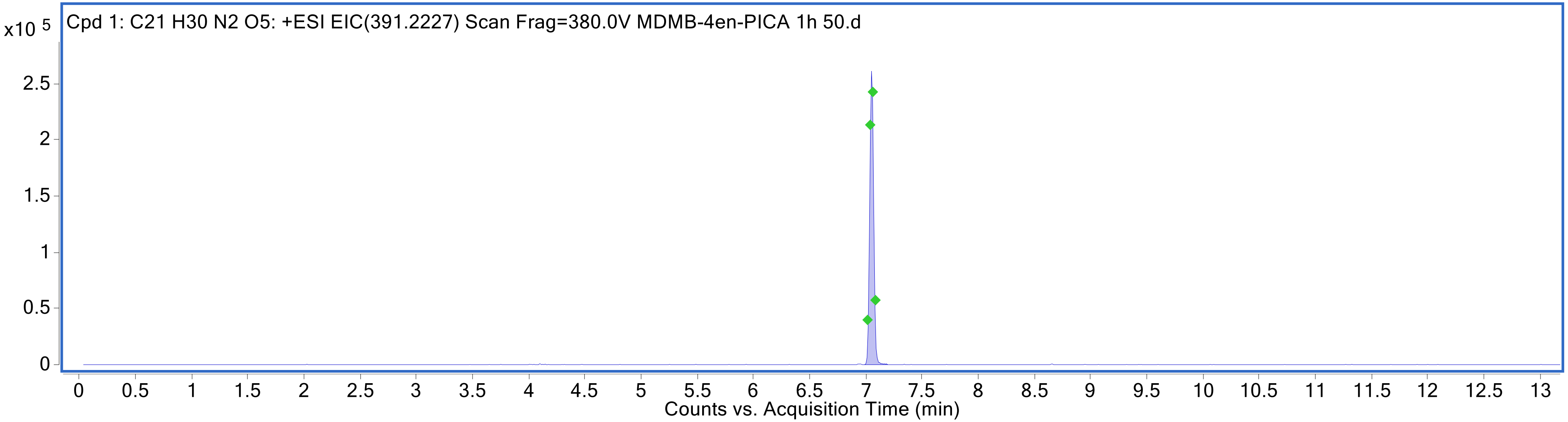

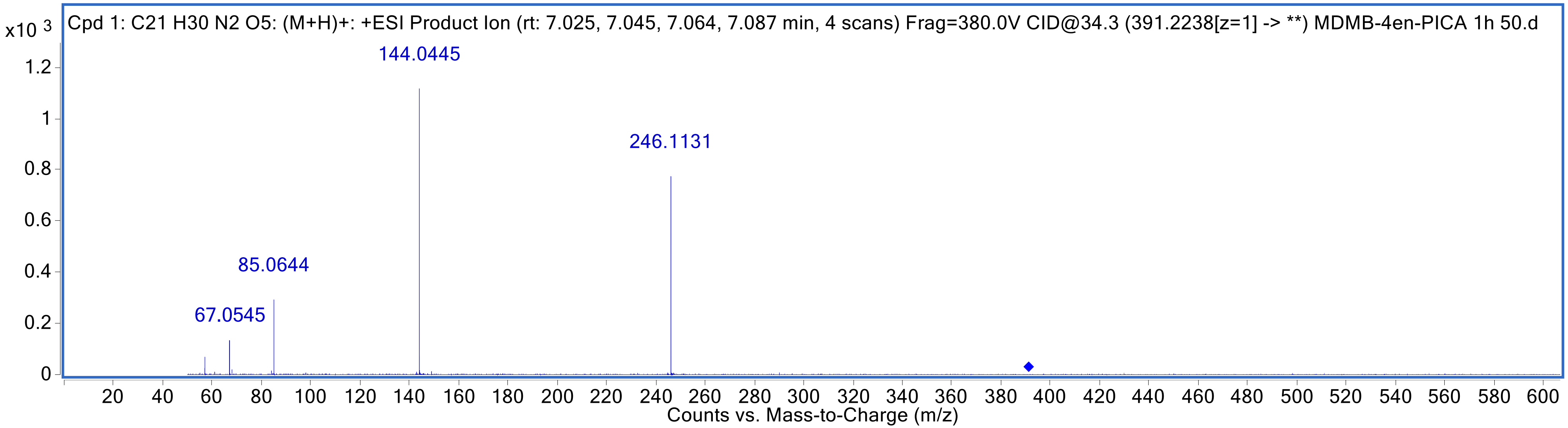

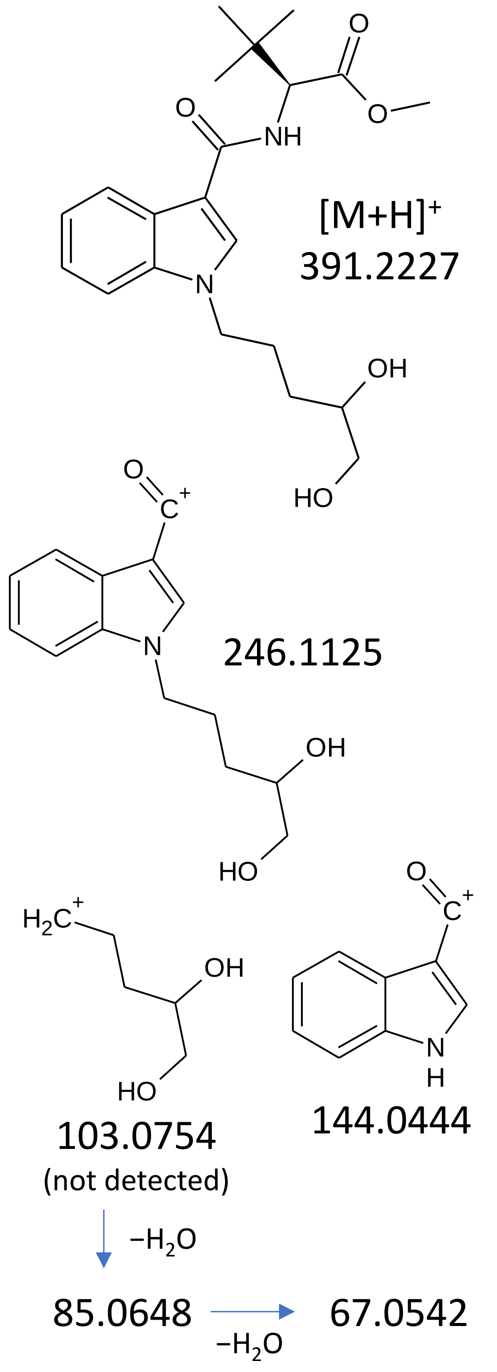

Dihydrodiol reference standard, RT 7.05 min, *m/z* 391.2236

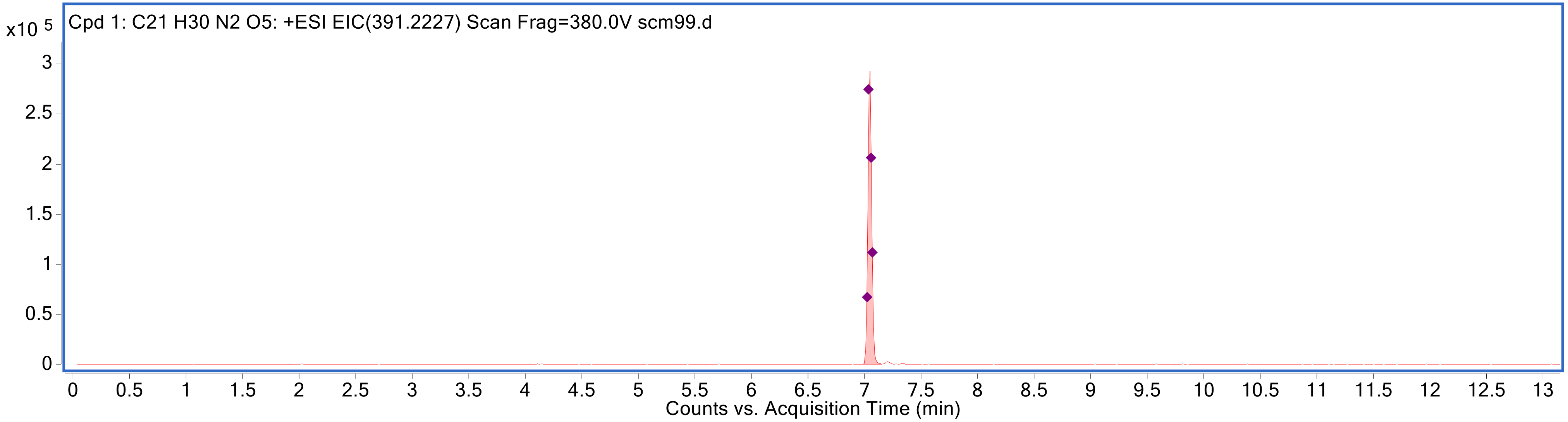

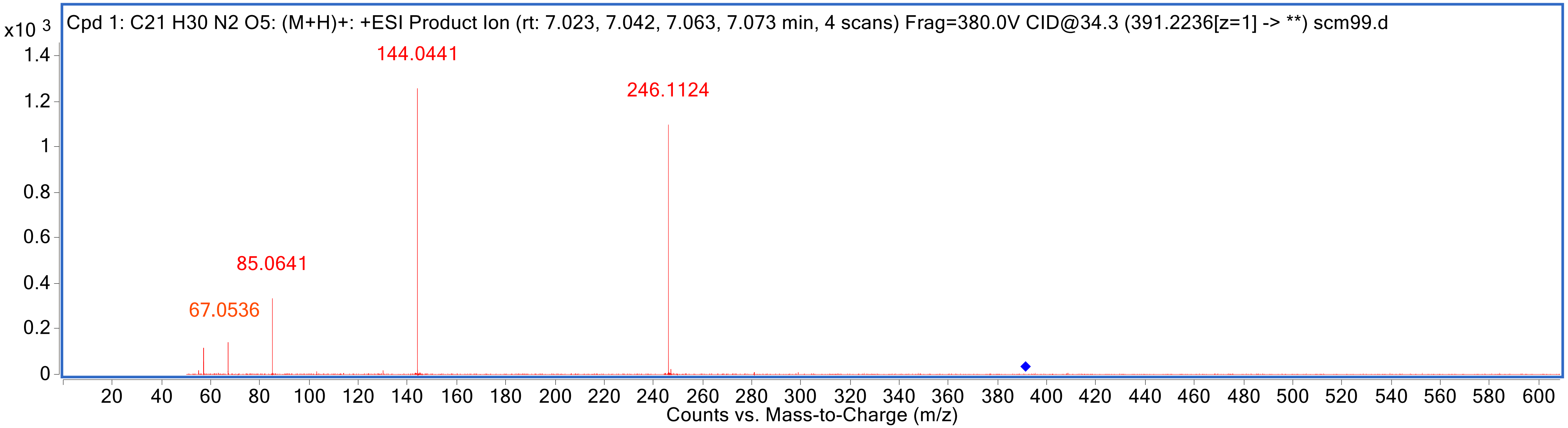

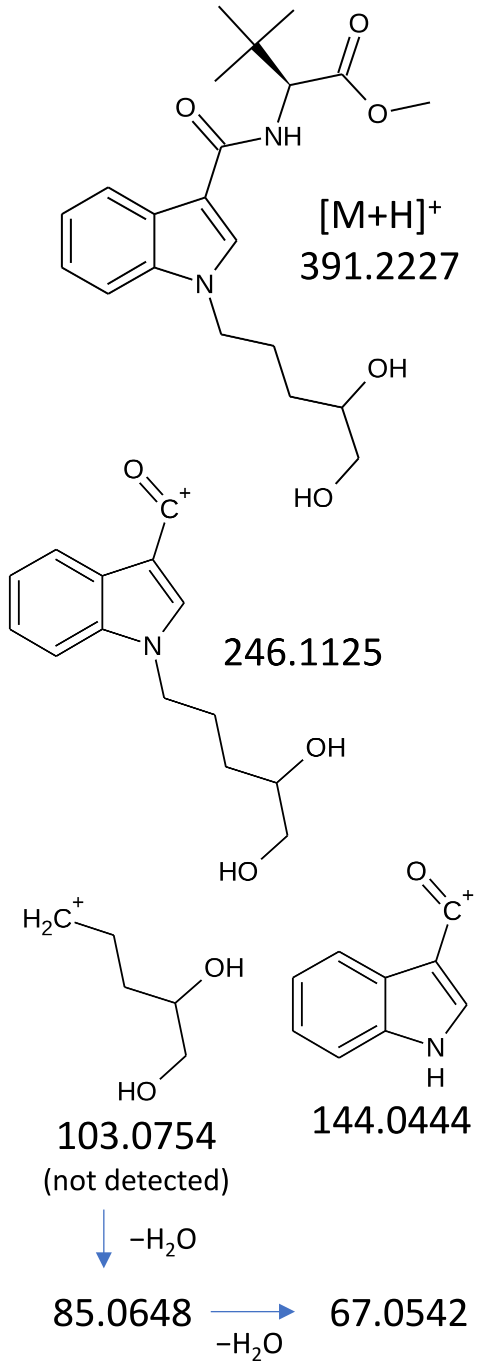

E3, Ester hydrolysis + dihydrodiol formation, RT 5.42 min, *m/z* 377.2072

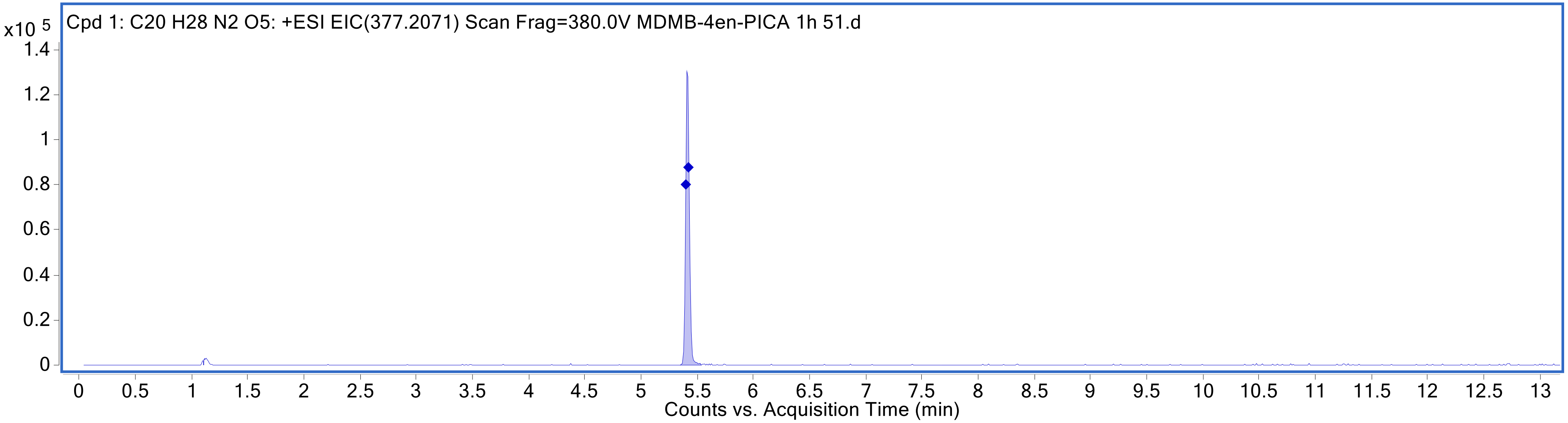

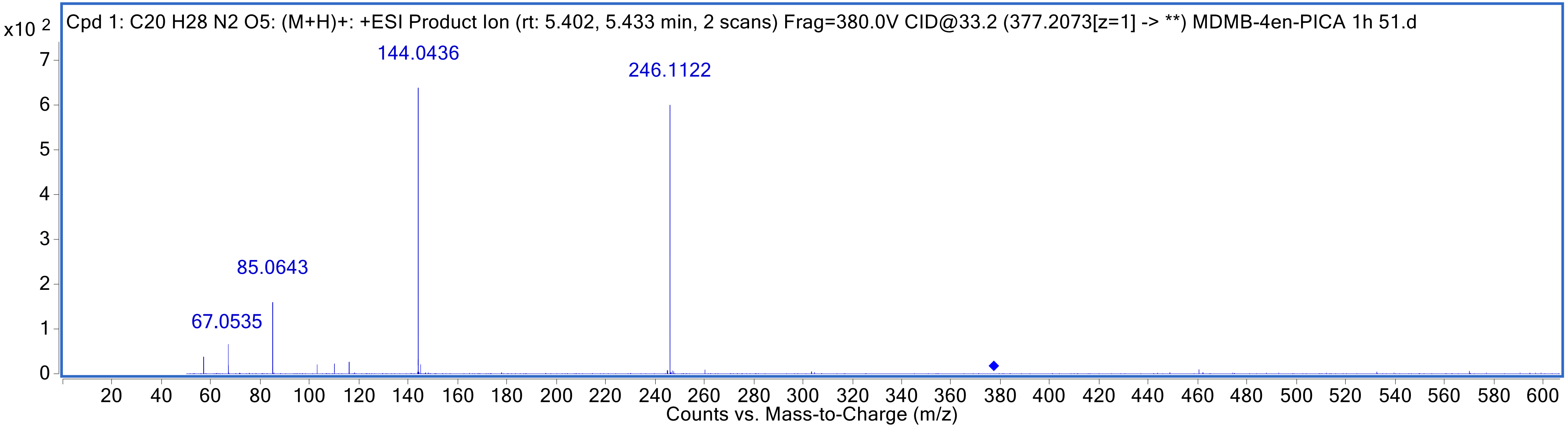

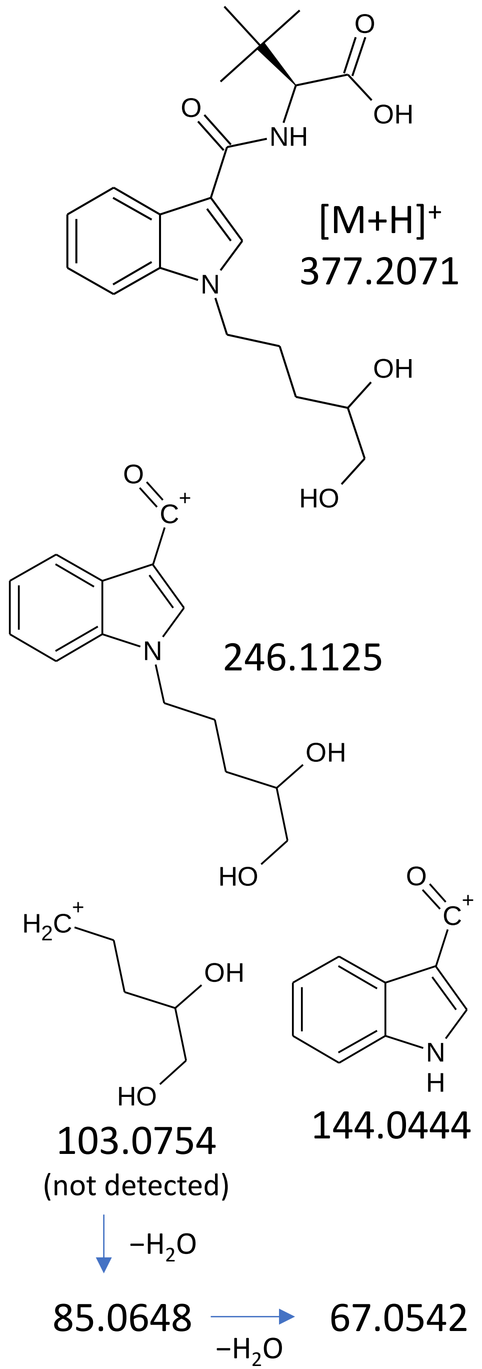

E4, Mono-hydroxylation (pentenyl tail), RT 9.08 min, *m/z* 373.2131

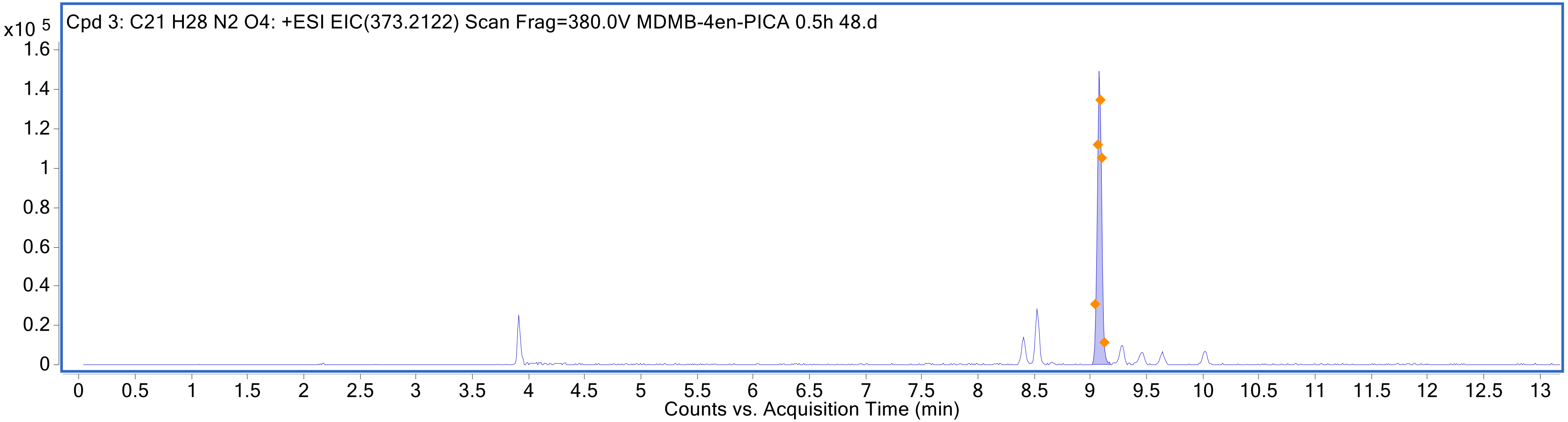

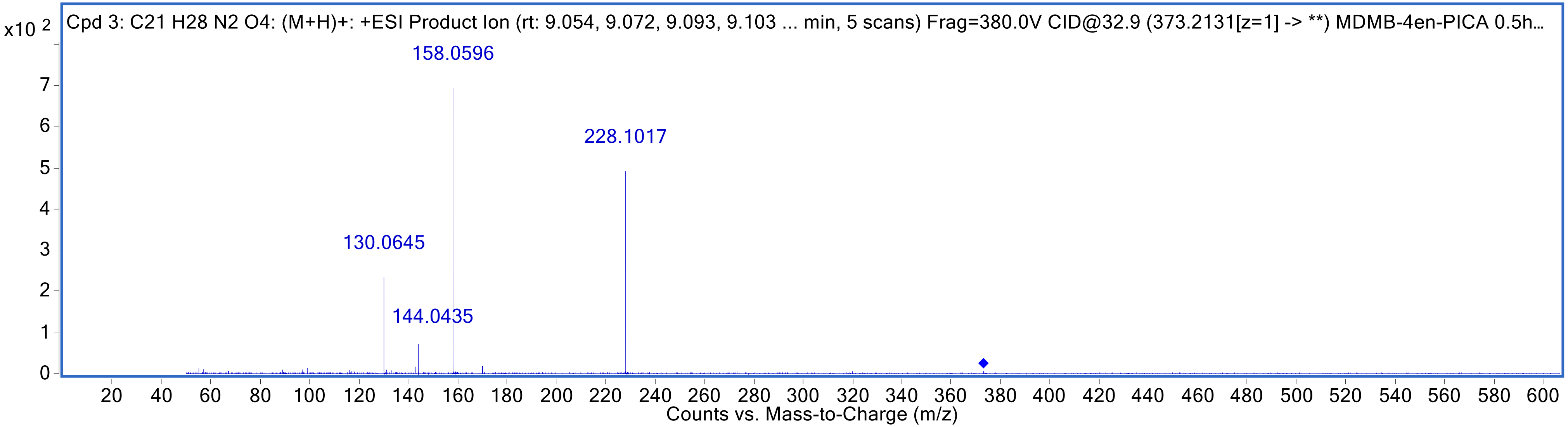

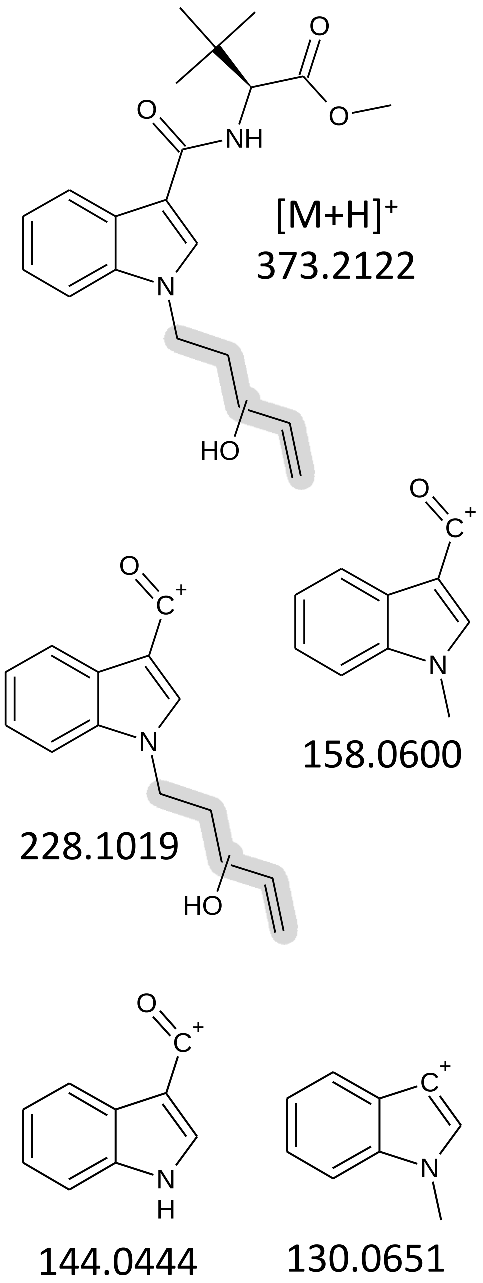

E5, Secondary amide hydrolysis + mono-hydroxylation (pentenyl tail), RT 7.05 min, *m/z* 246.1133

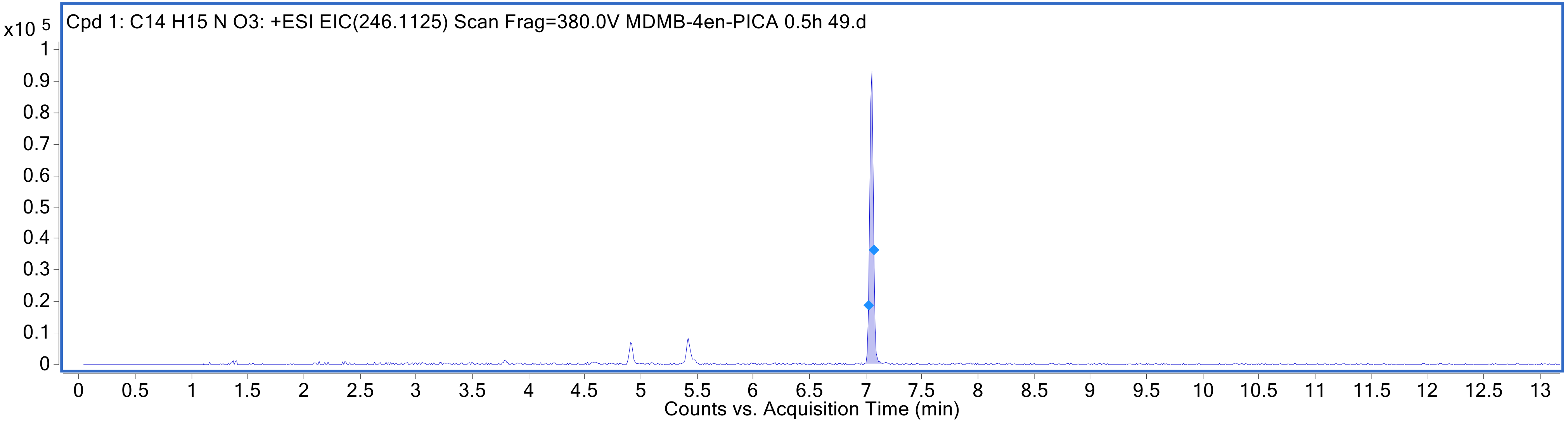

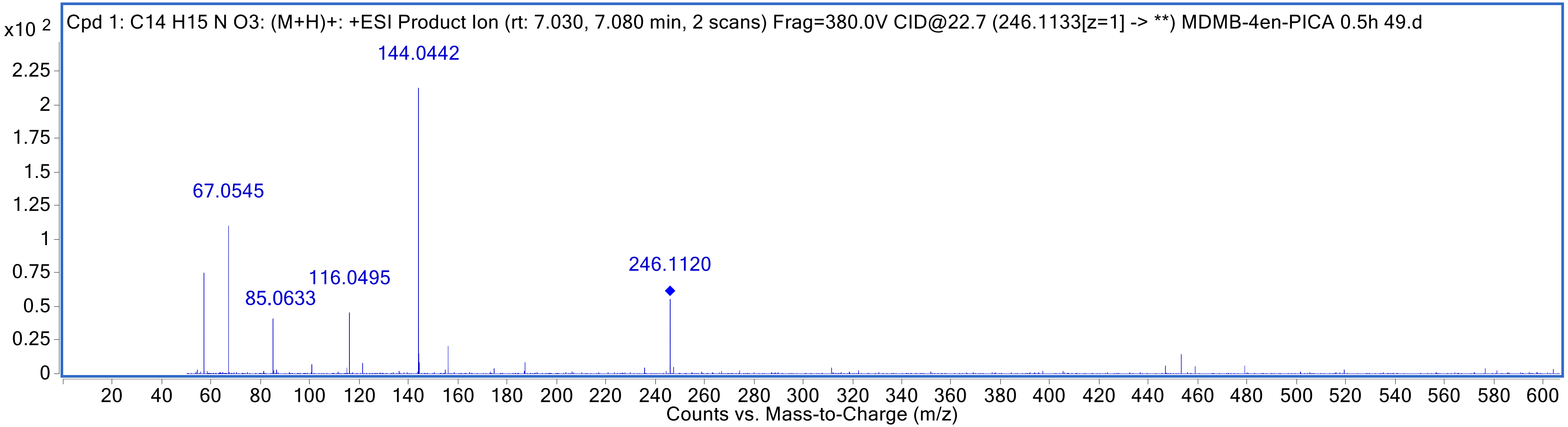

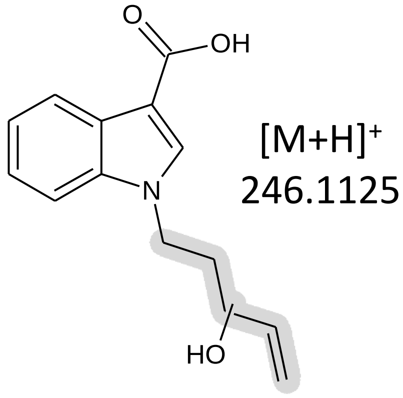

$[M+H]^+$
246.1125

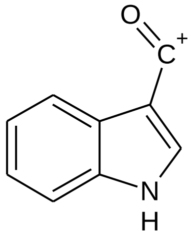

144.0444

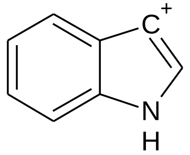

116.0495

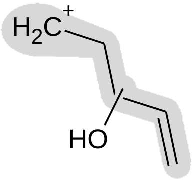

85.0648 → 67.0542
$-H_2O$

E6, Ester hydrolysis + *N*-dealkylation, RT 5.71 min, *m/z* 275.1396

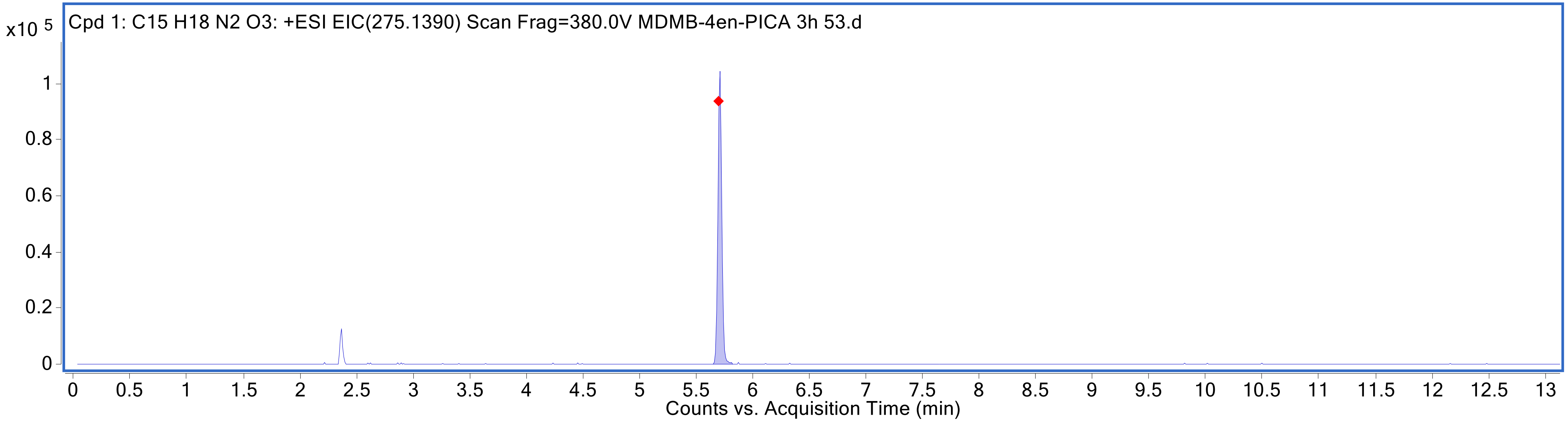

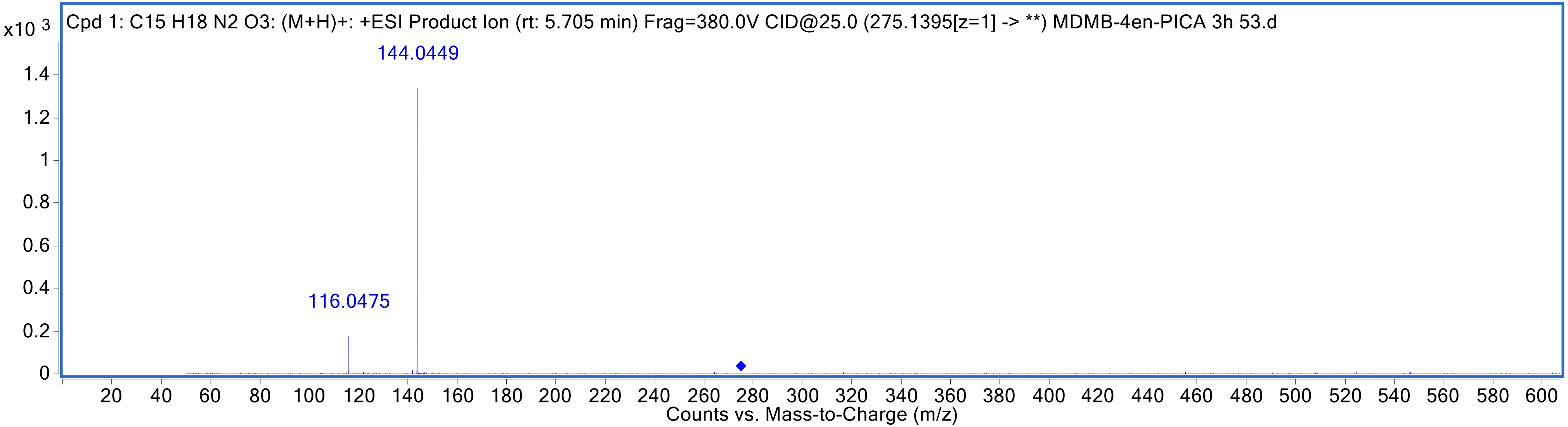

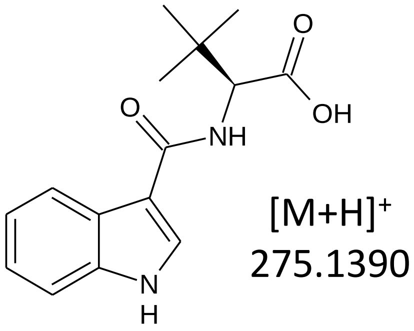

$[M+H]^+$
275.1390

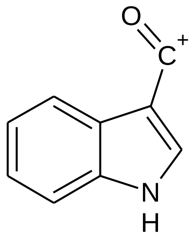

144.0444

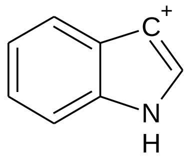

116.0495

E7, Ester hydrolysis + glucuronidation, RT 8.13 min, *m/z* 519.2341

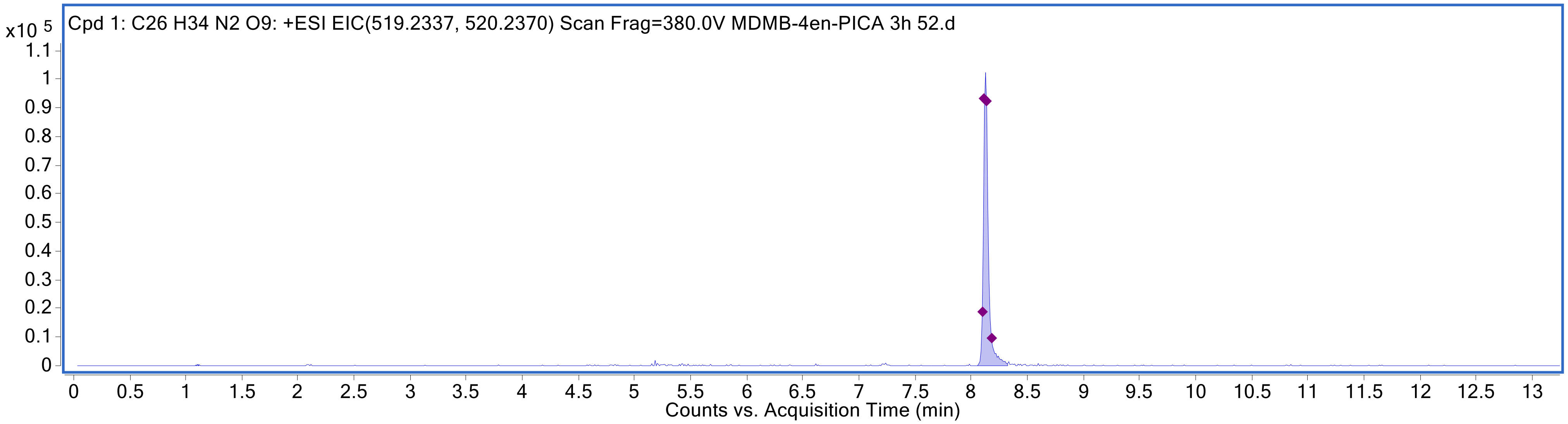

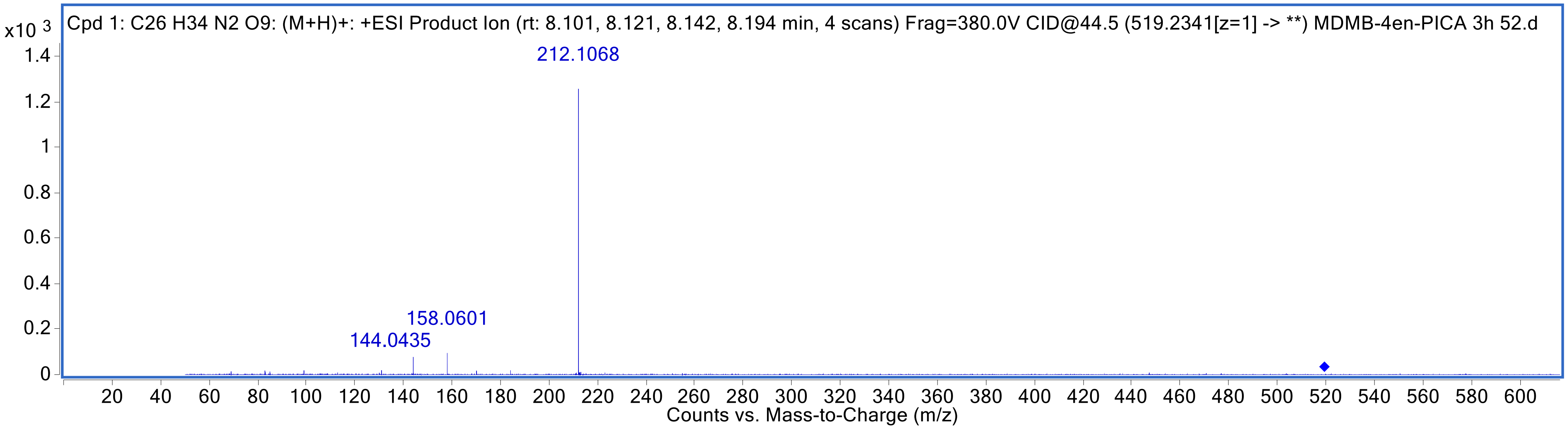

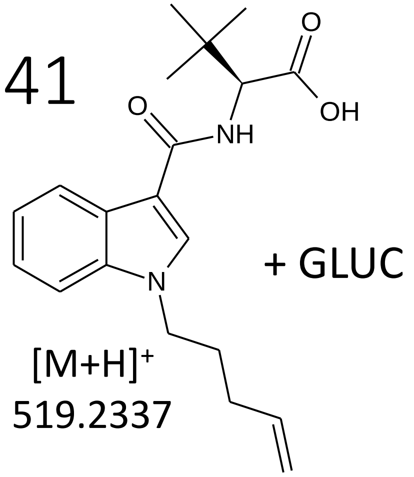

[M+H]+
519.2337

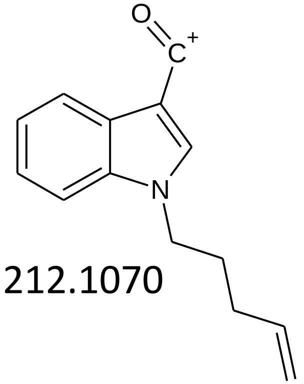

212.1070

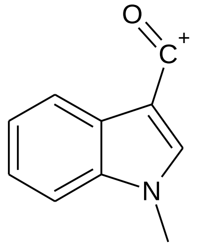

158.0600

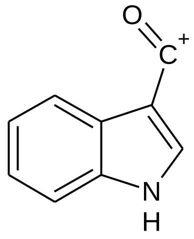

144.0444

E8, Ester hydrolysis + di-hydroxylation (pentenyl tail + *tert*-butyl), RT 7.95 min, *m/z* 375.1917

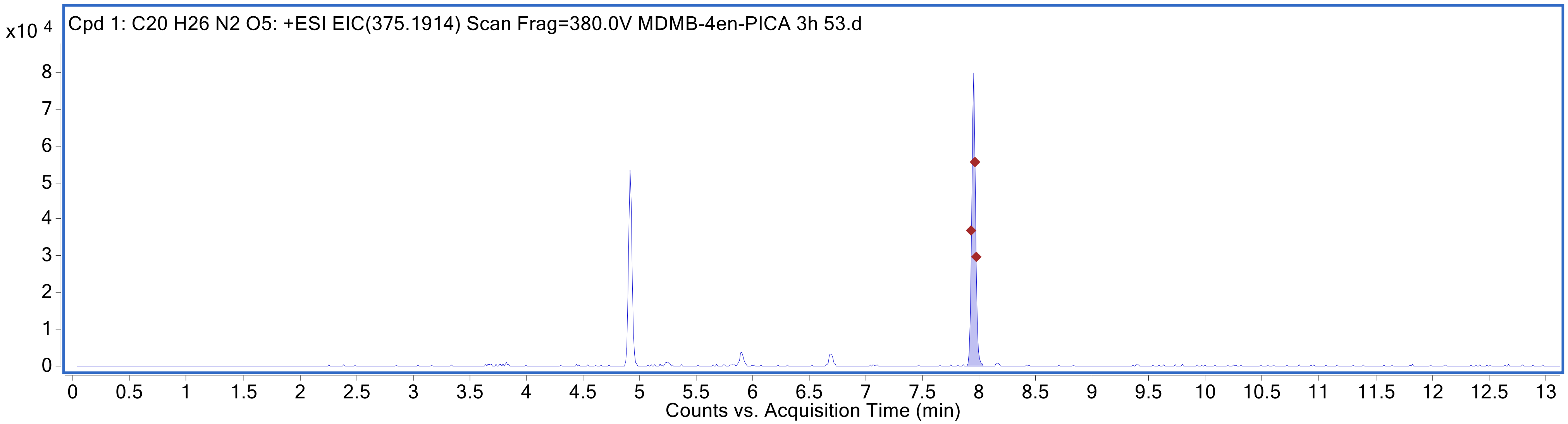

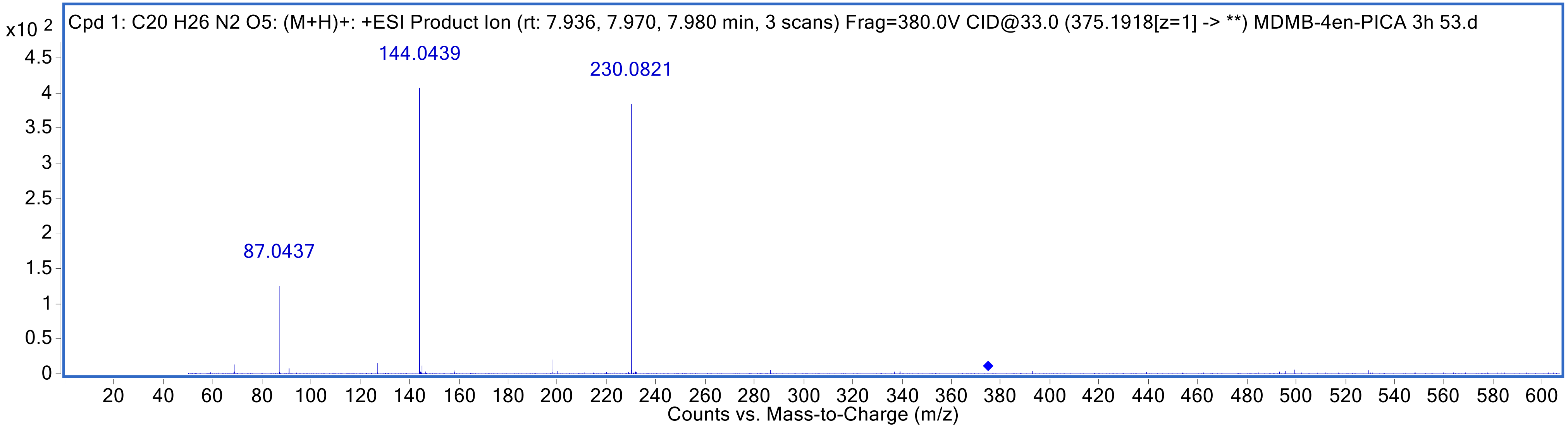

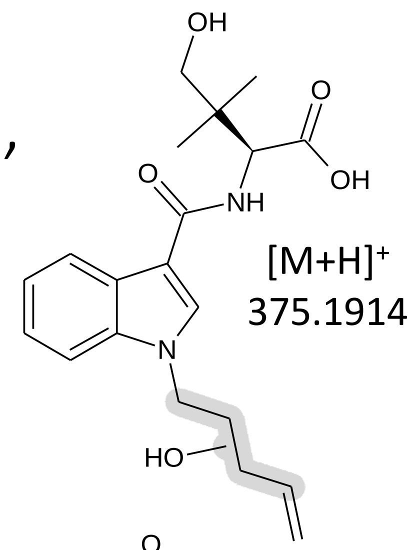

$[M+H]^+$
375.1914

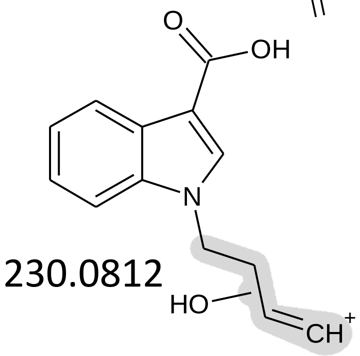

230.0812

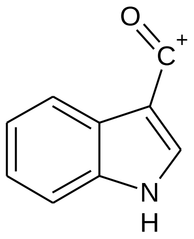

144.0444

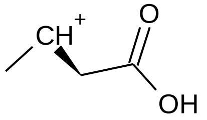

87.0441

E9, Mono-hydroxylation + glucuronidation (indole core), RT 6.74 min, *m/z* 549.2434

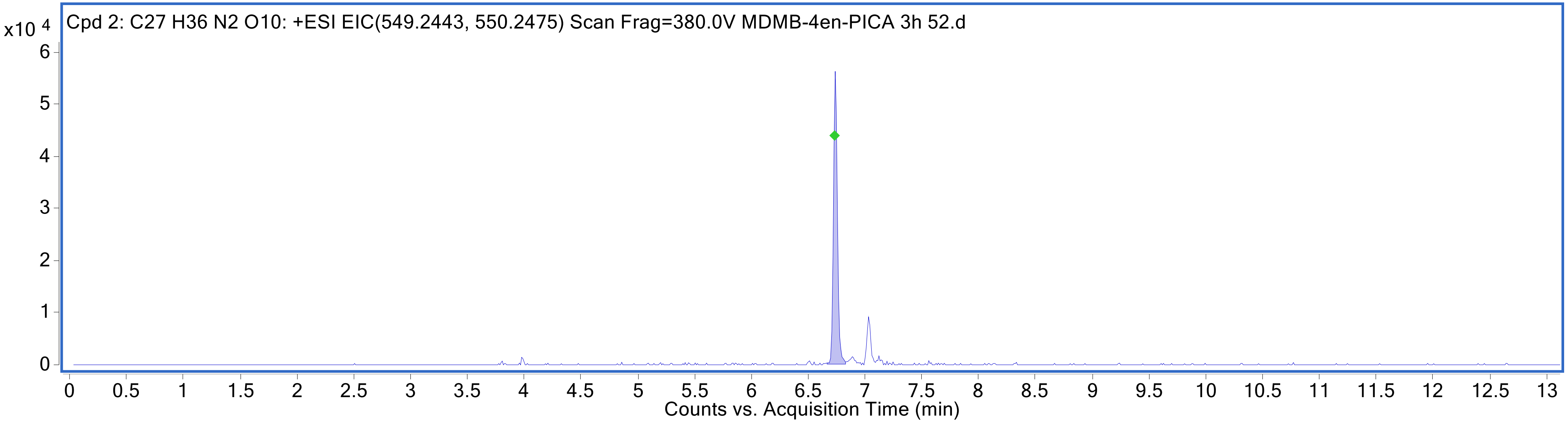

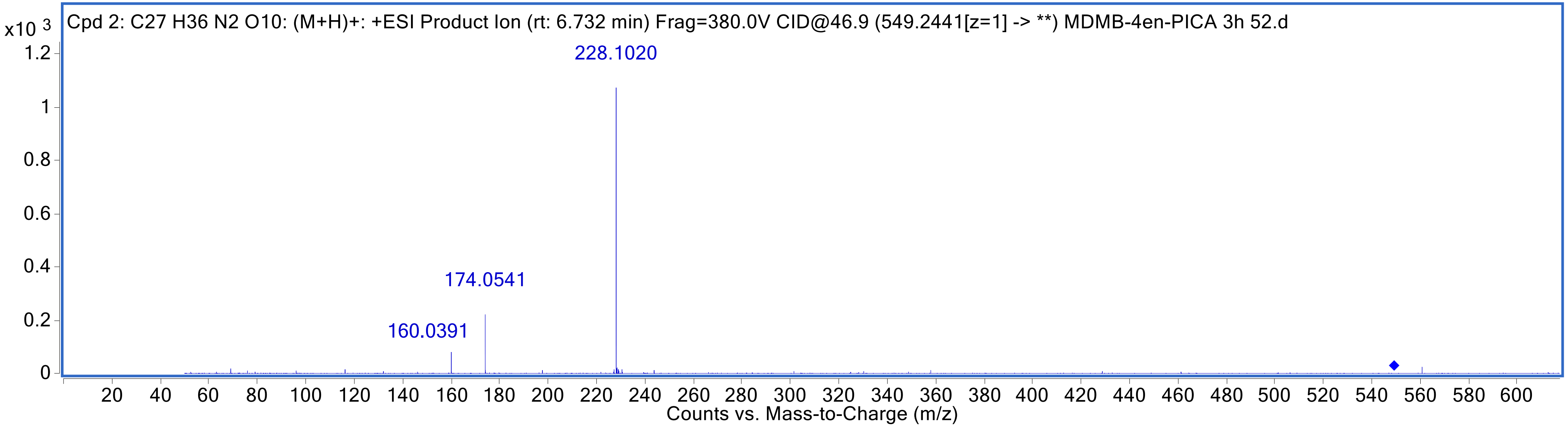

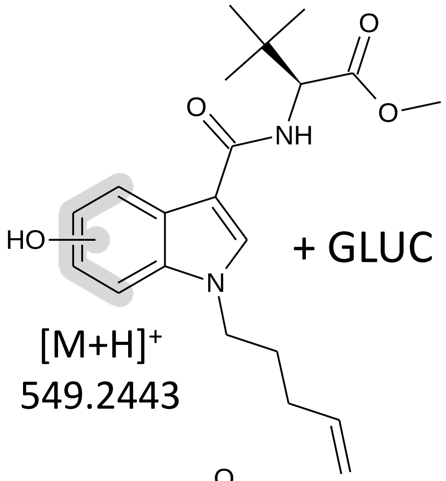

$[M+H]^+$
549.2443

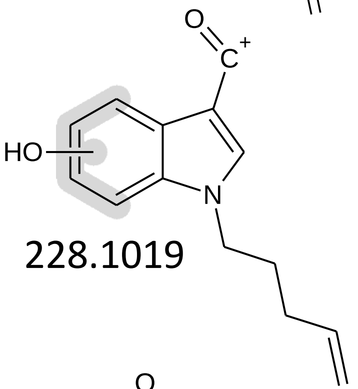

228.1019

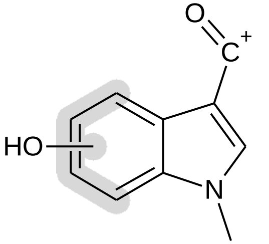

174.0550

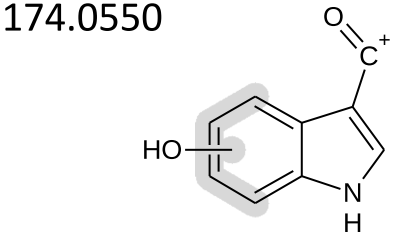

160.0393

E10, Ester hydrolysis + dihydrodiol formation + dehydrogenation, RT 4.91 min, *m/z* 375.1916

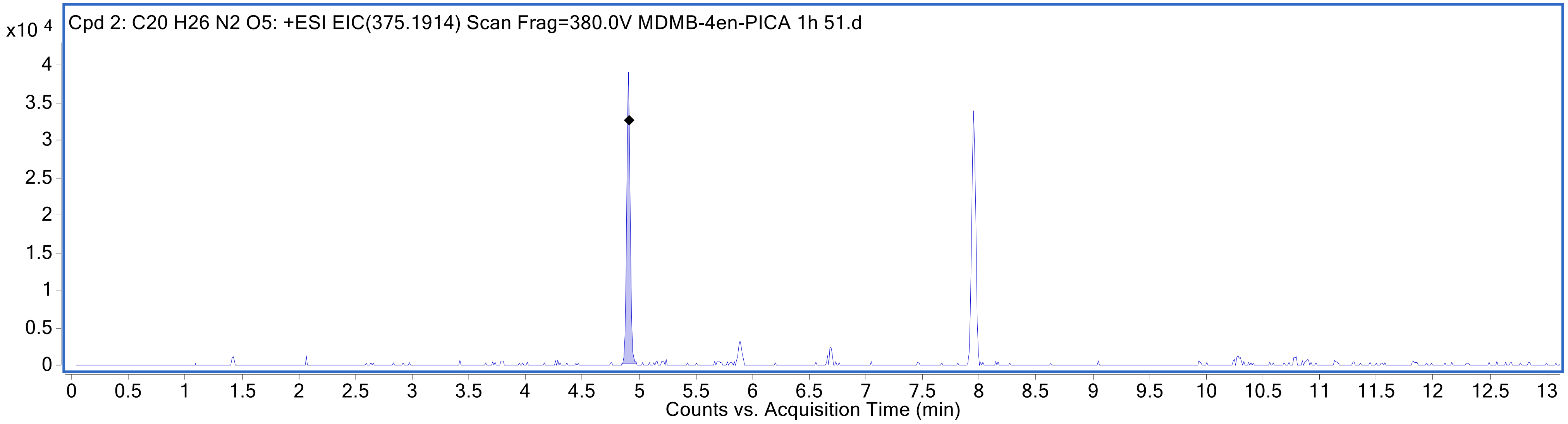

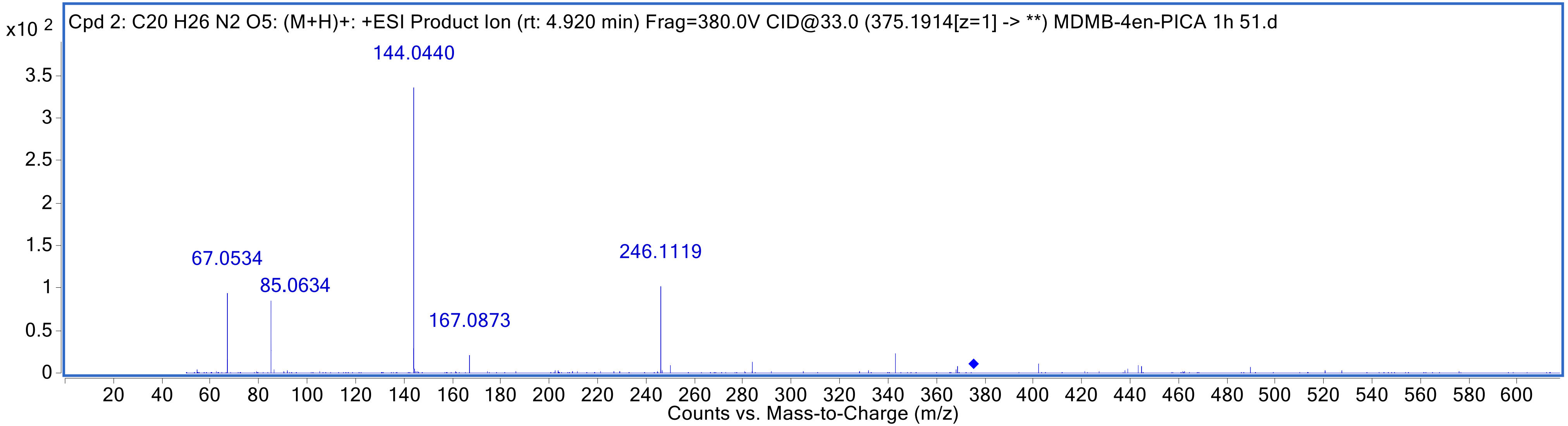

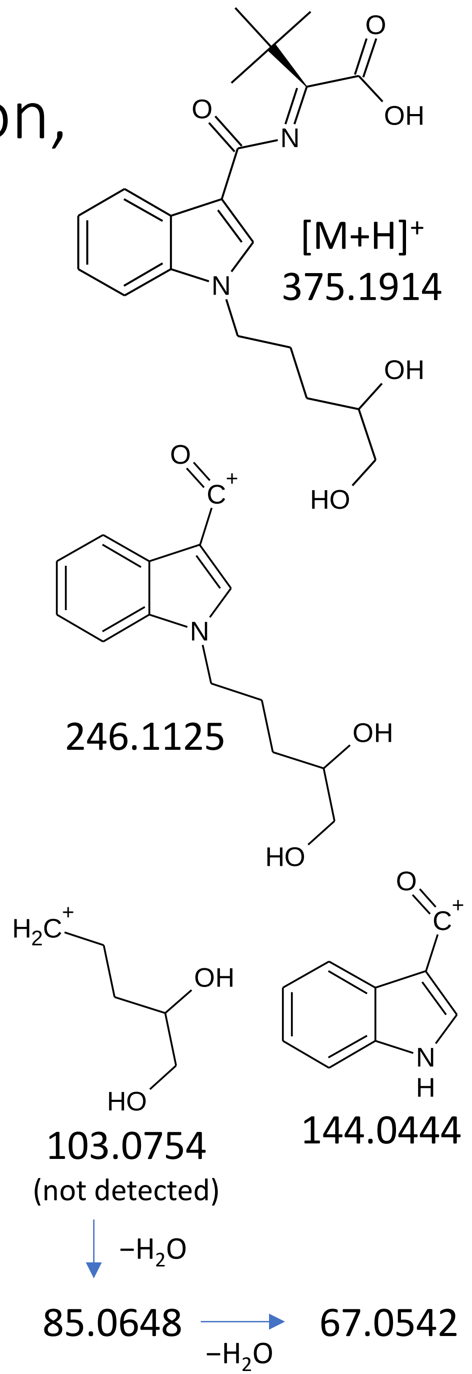

E11, Di-hydroxylation (pentenyl tail), RT 8.47 min, *m/z* 389.2077

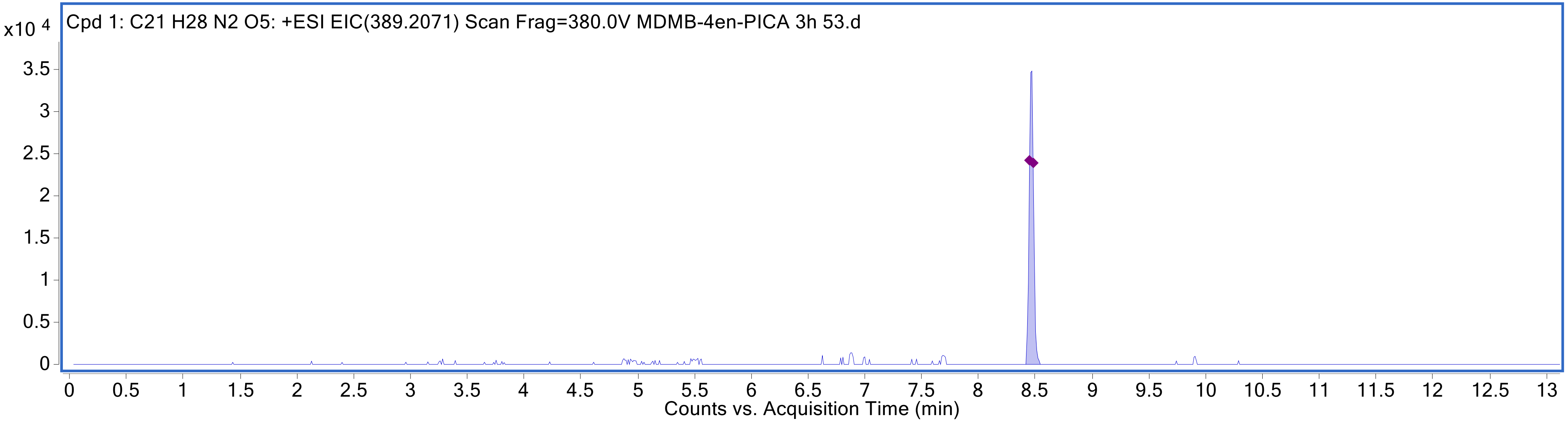

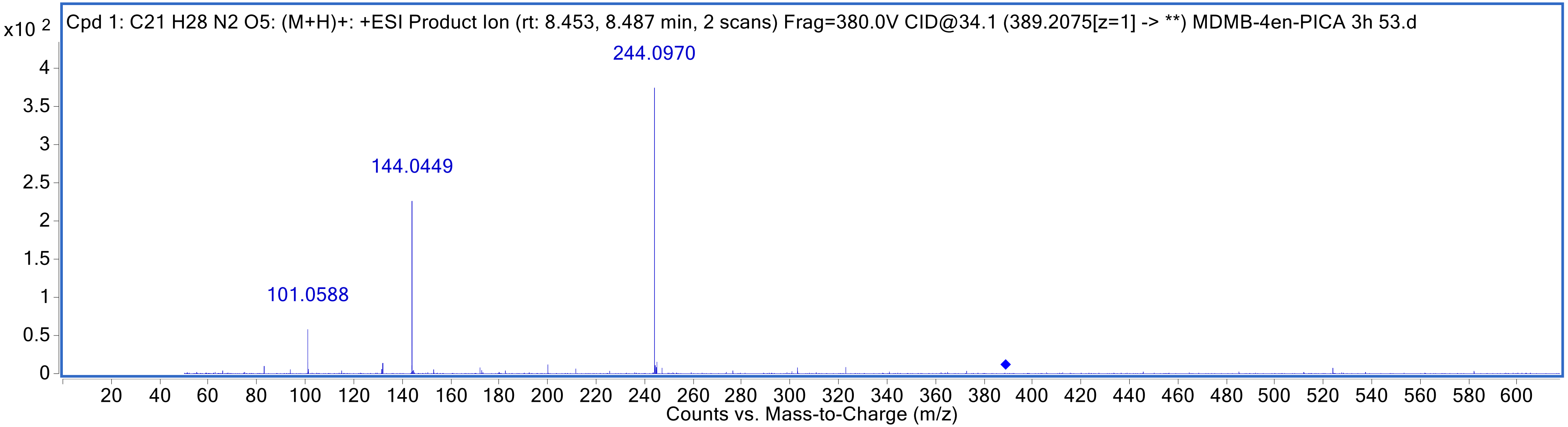

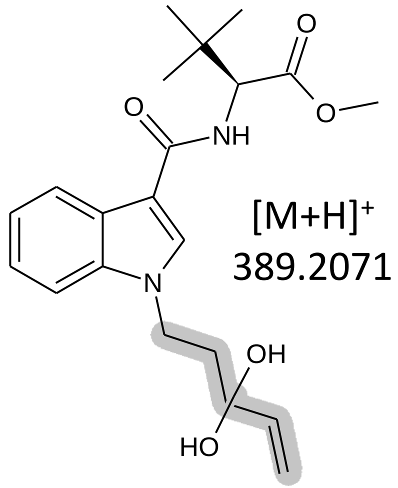

$[M+H]^+$
389.2071

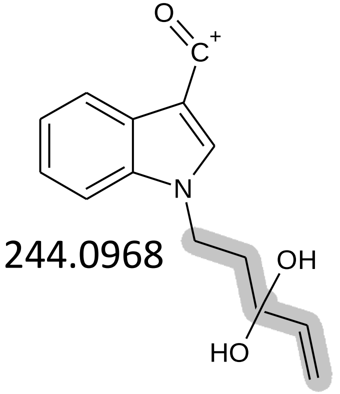

244.0968

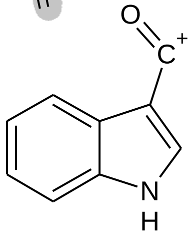

144.0444

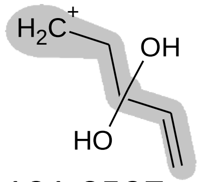

101.0597

E12, Ester hydrolysis + mono-hydroxylation (pentenyl tail), RT 7.14 min, *m/z* 359.1966

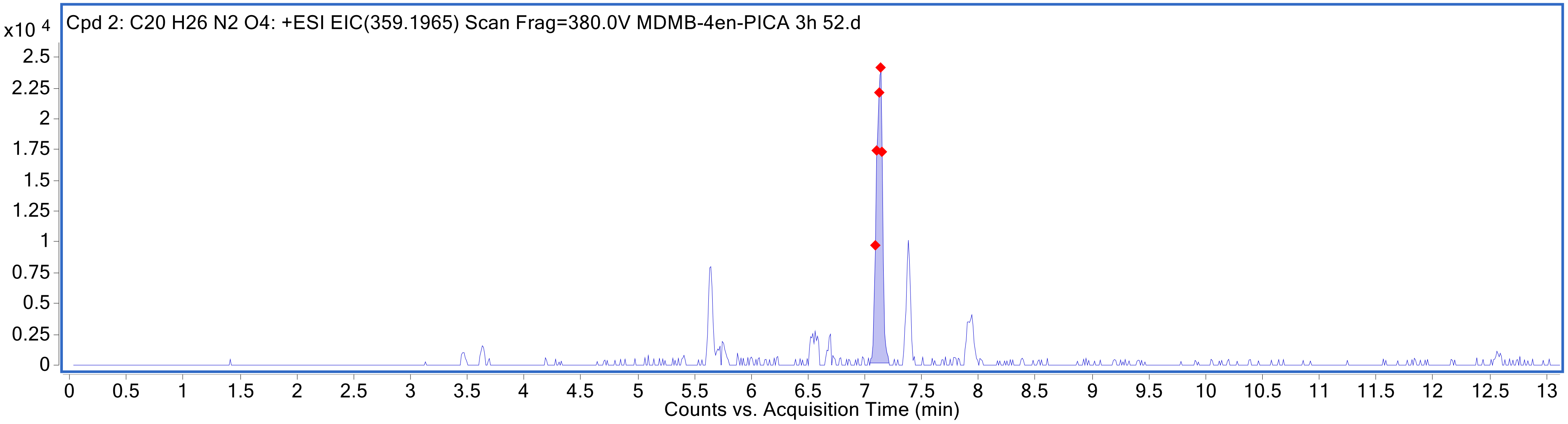

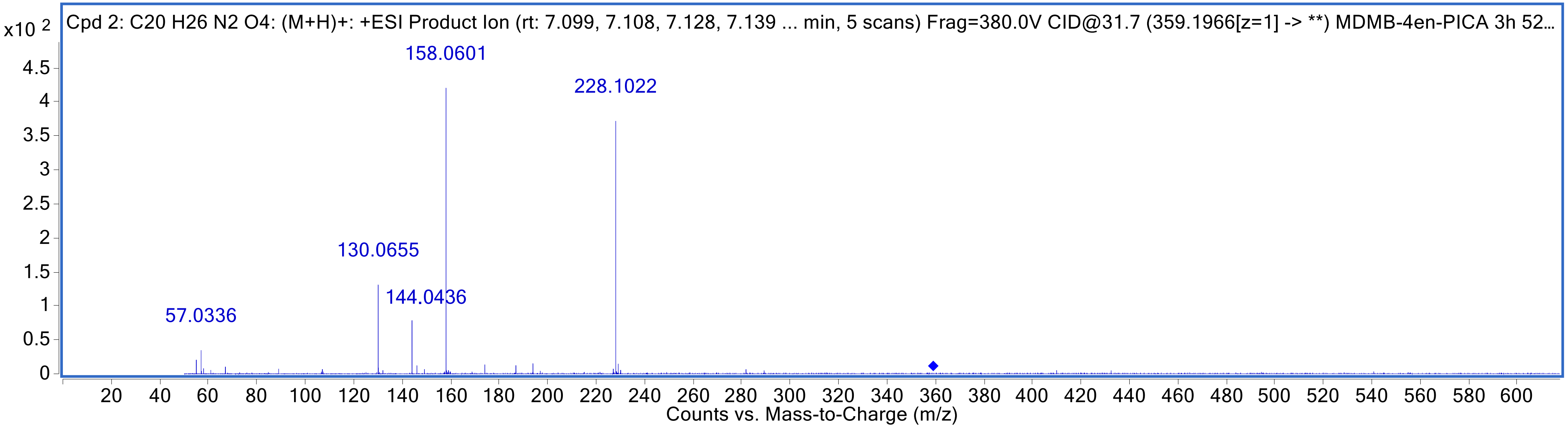

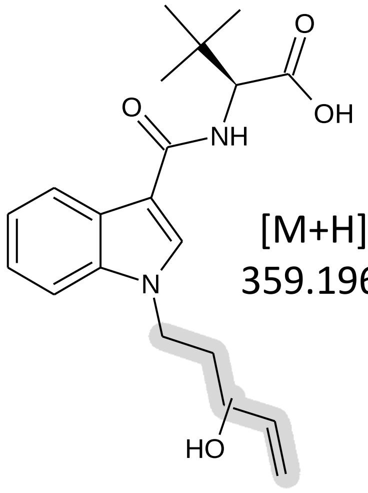

$[M+H]^+$
359.1965

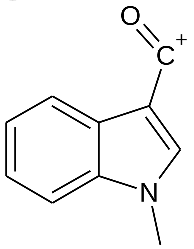

158.0600

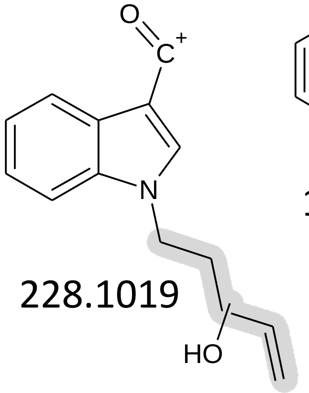

228.1019

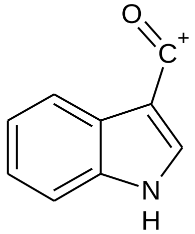

144.0444

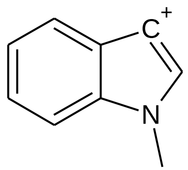

130.0651

E13, Ester hydrolysis + dehydrogenation, RT 9.81 min, *m/z* 341.1863

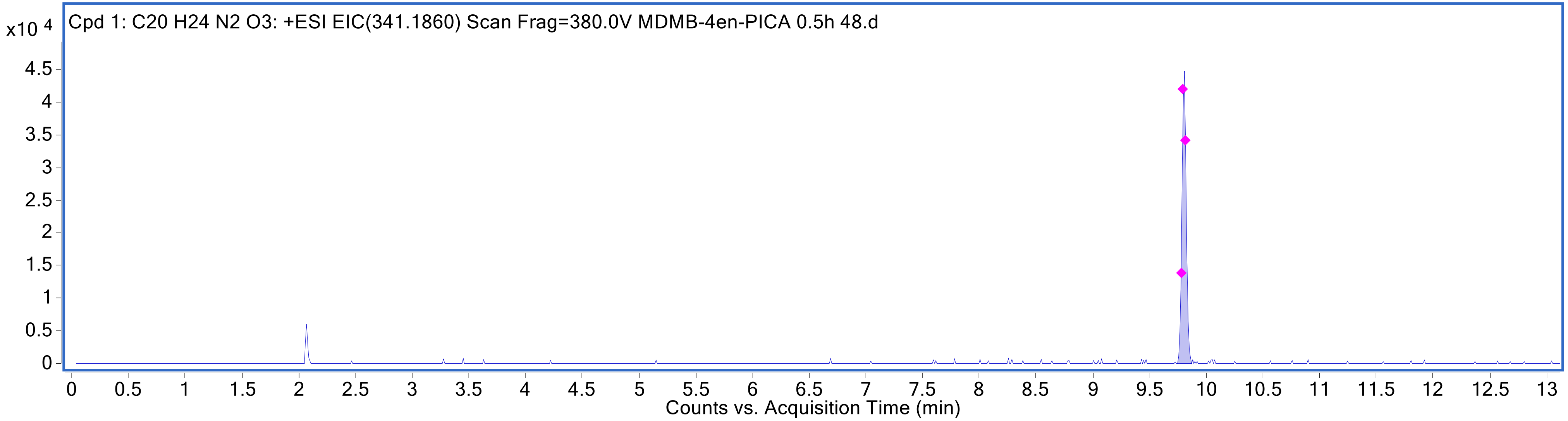

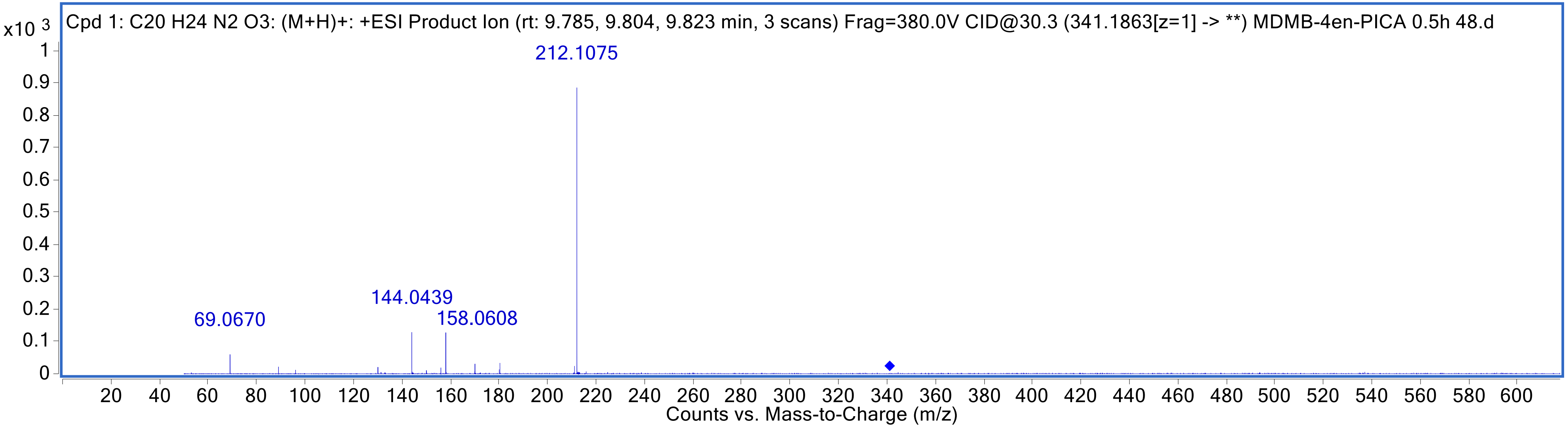

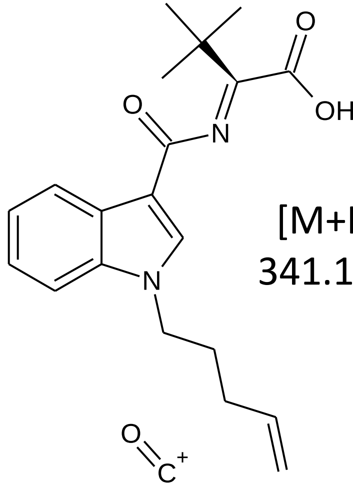

$[M+H]^+$
341.1860

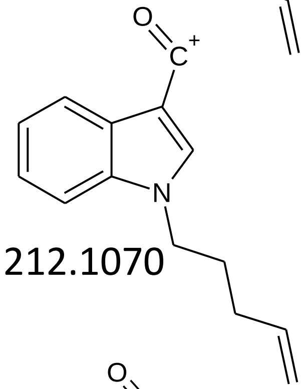

212.1070

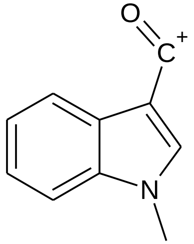

158.0600

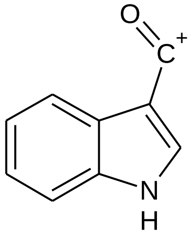

144.0444

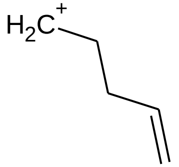

69.0699

E14, Dihydrodiol formation + mono-hydroxylation (indole core) + glucuronidation, RT 4.11 min, *m/z* 583.2490

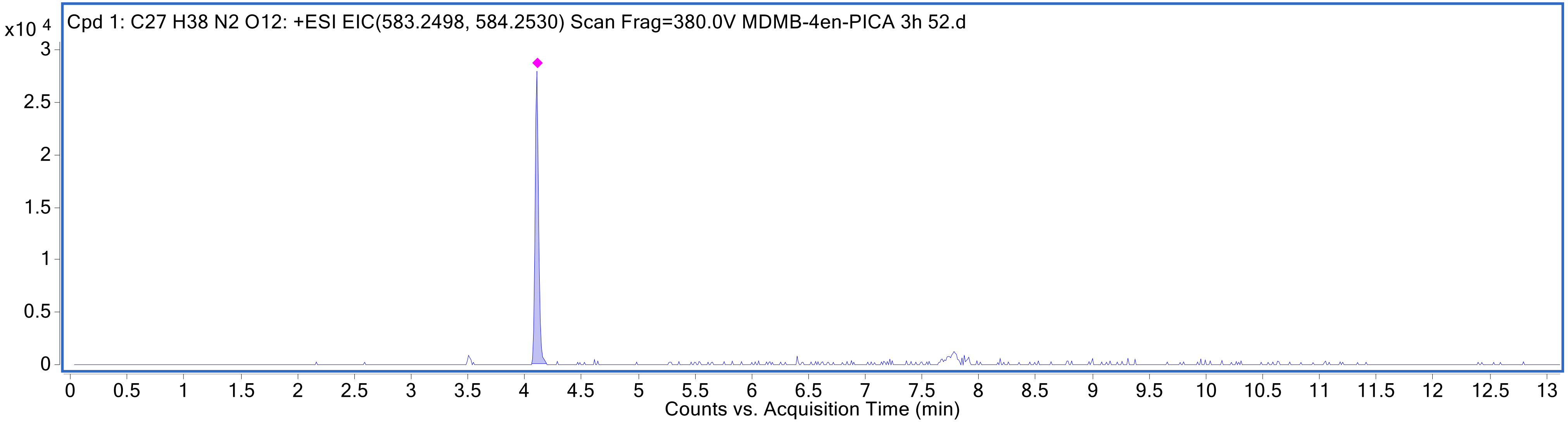

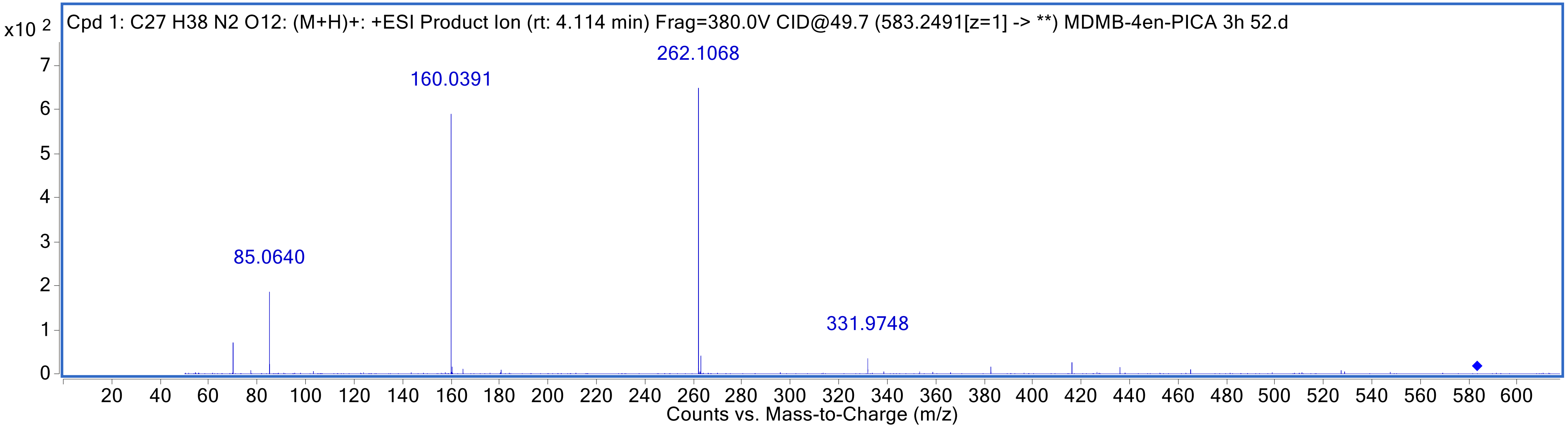

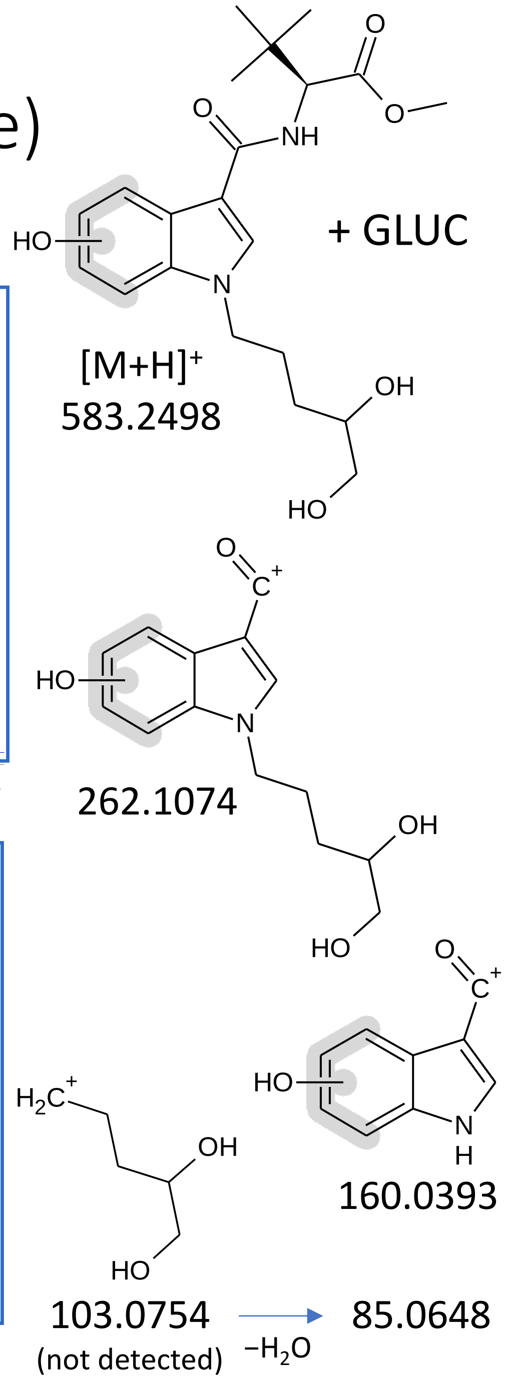

E15, Ketone formation + mono-hydroxylation (pentenyl tail), RT 8.16 min, *m/z* 387.1915

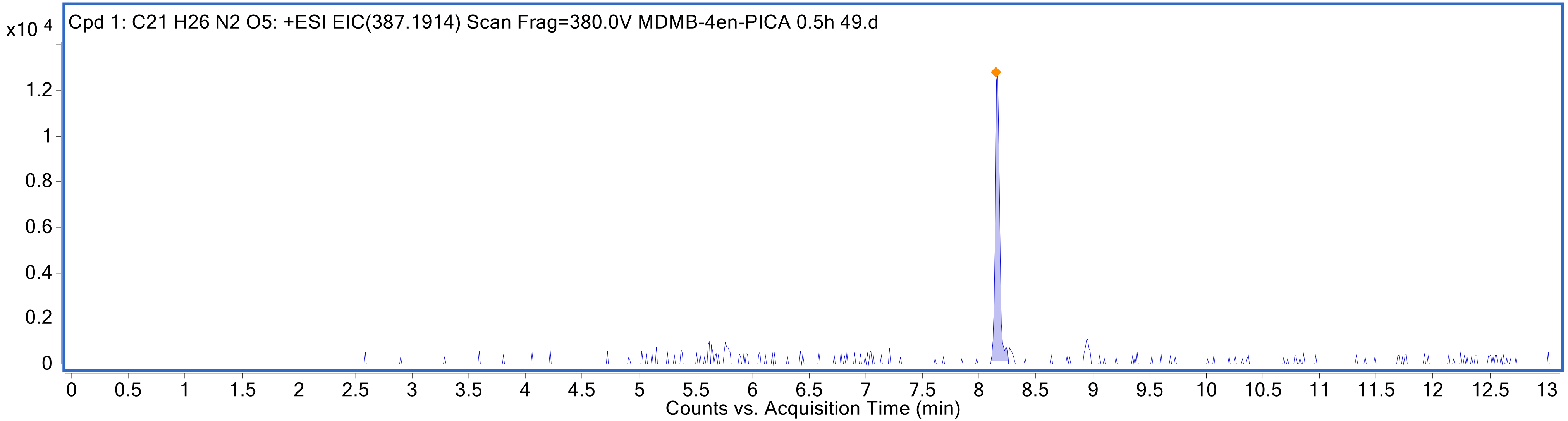

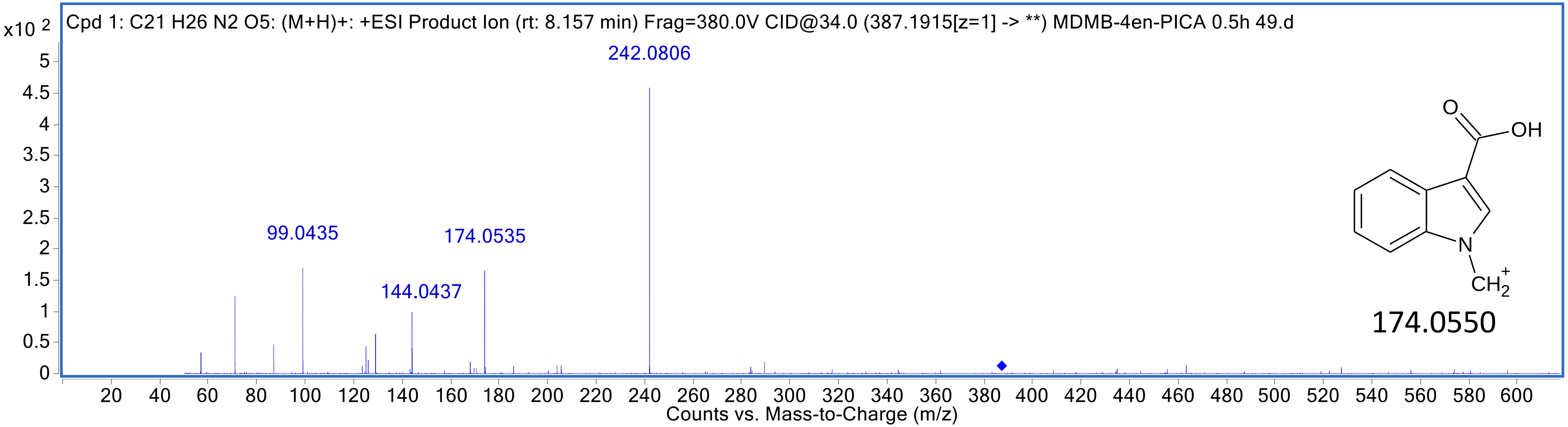

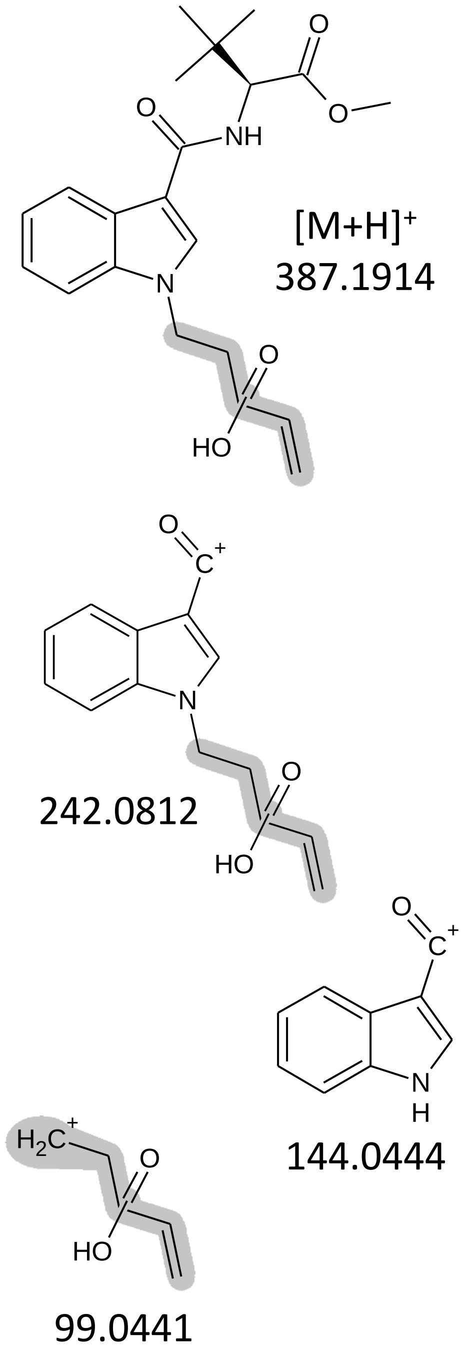

E16, Mono-hydroxylation (pentenyl tail), RT 8.53 min, *m/z* 373.2117

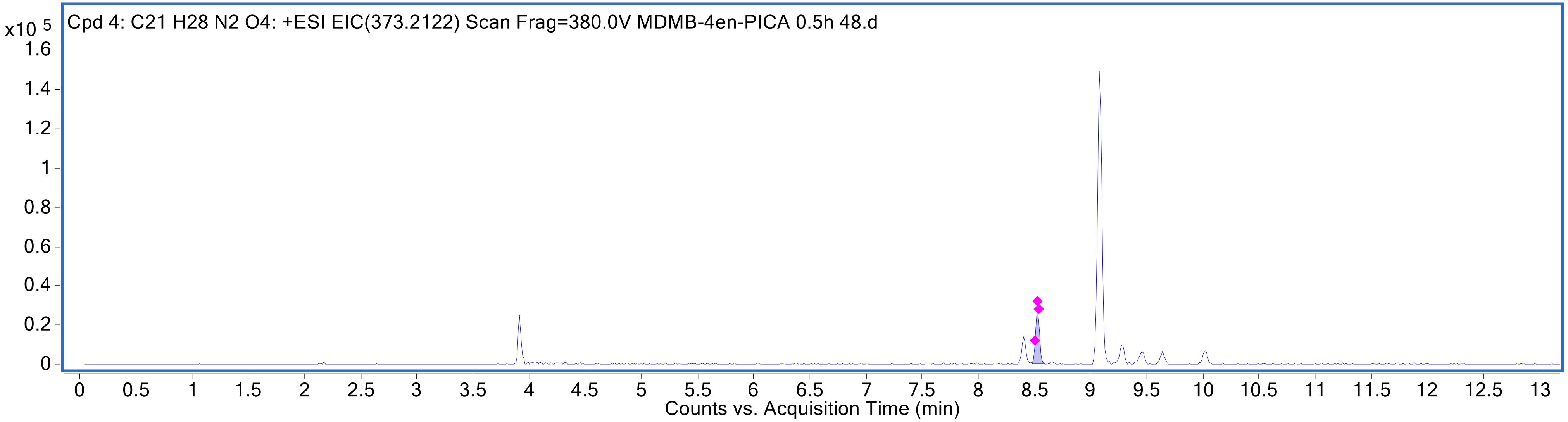

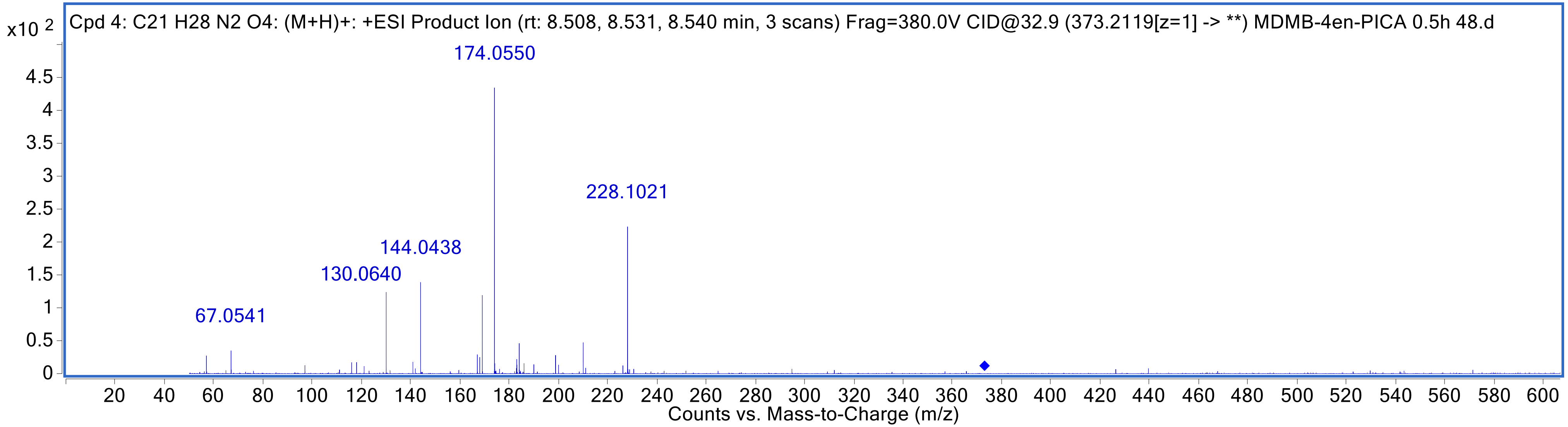

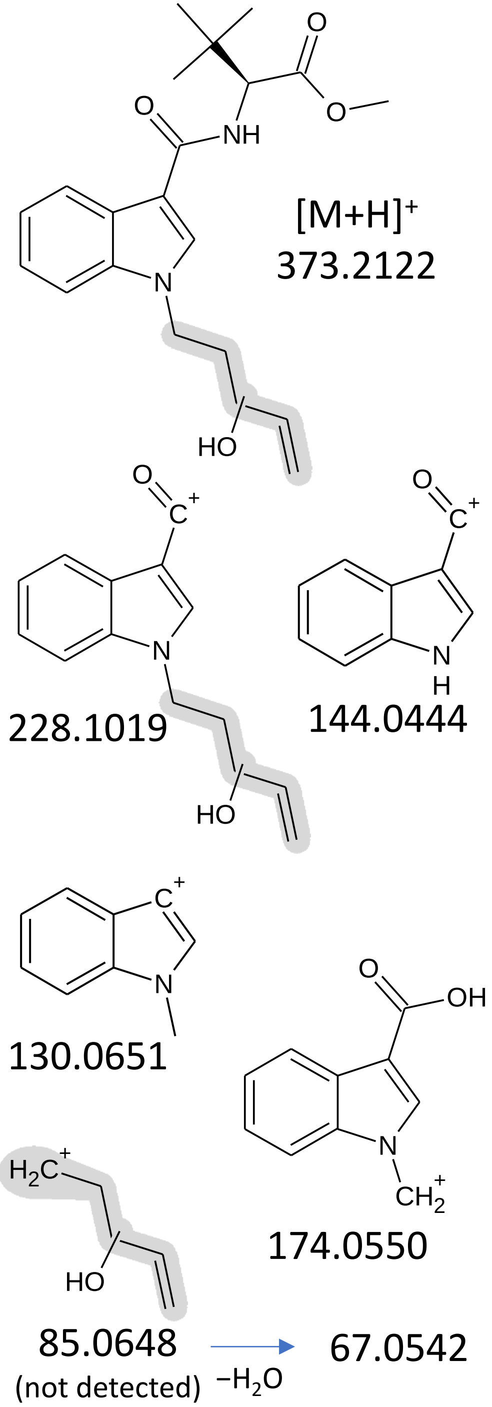

E17, Mono-hydroxylation (indole core), RT 9.29 min, *m/z* 373.2121

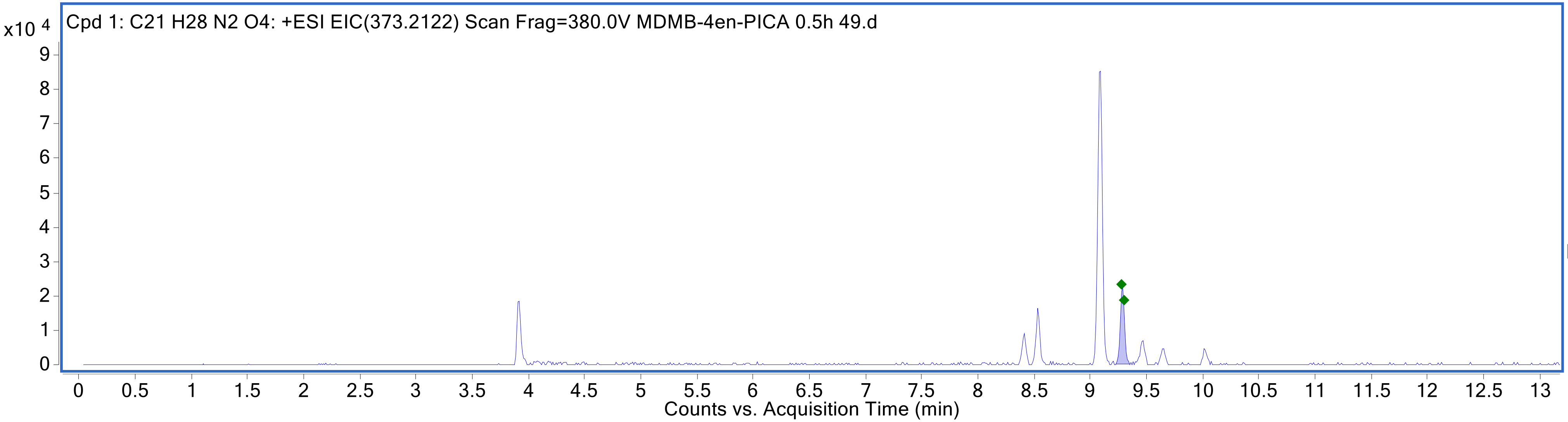

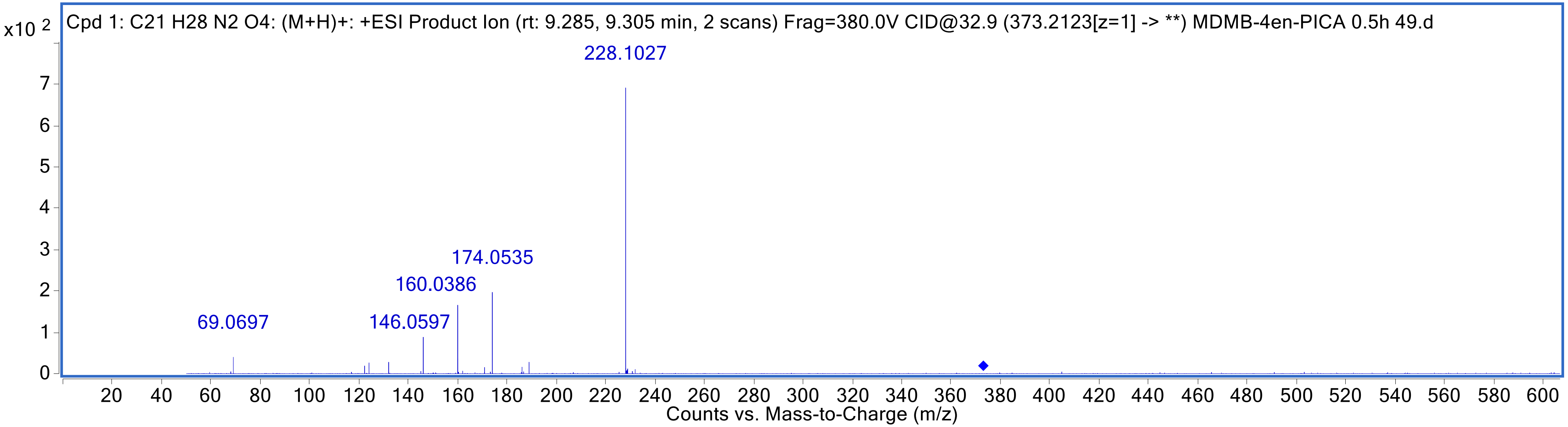

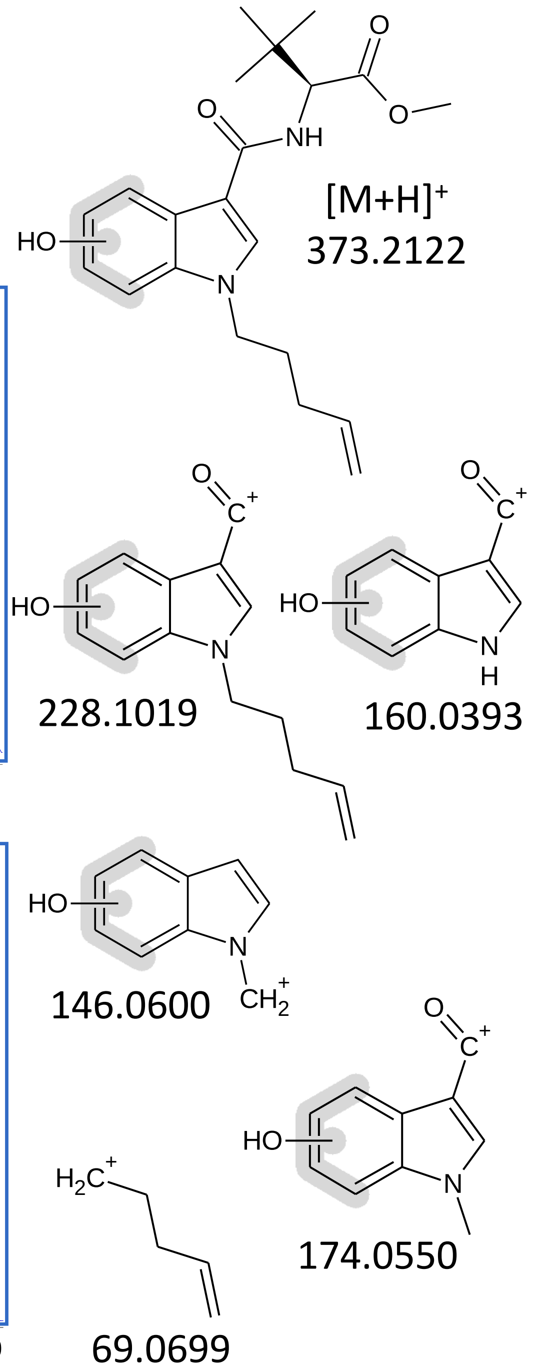

E18, Ester hydrolysis + mono-hydroxylation (indole core), RT 7.38 min, *m/z* 359.1963

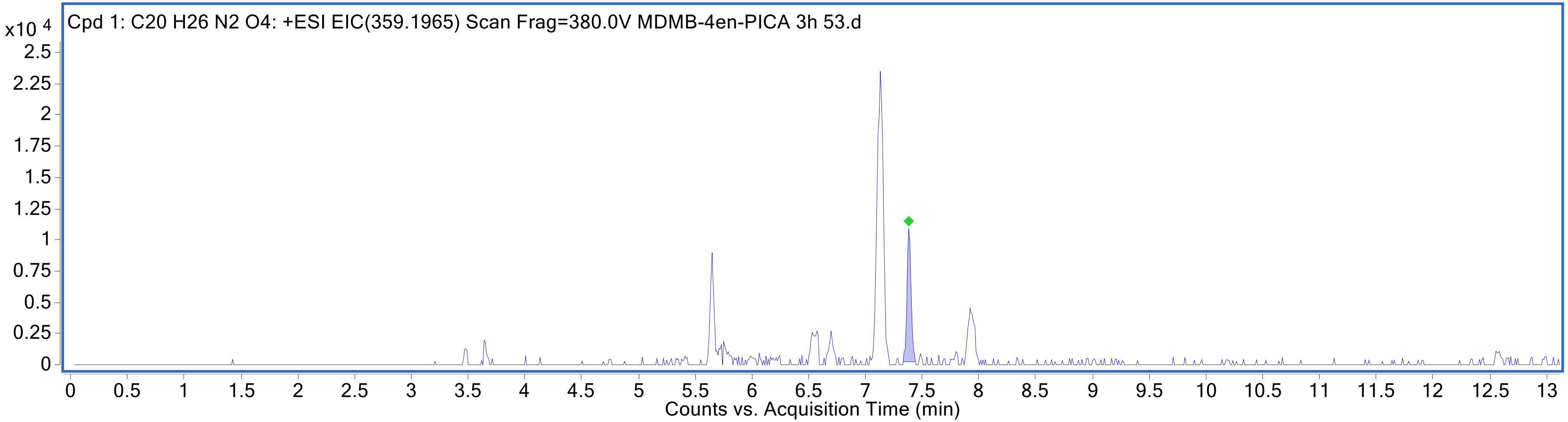

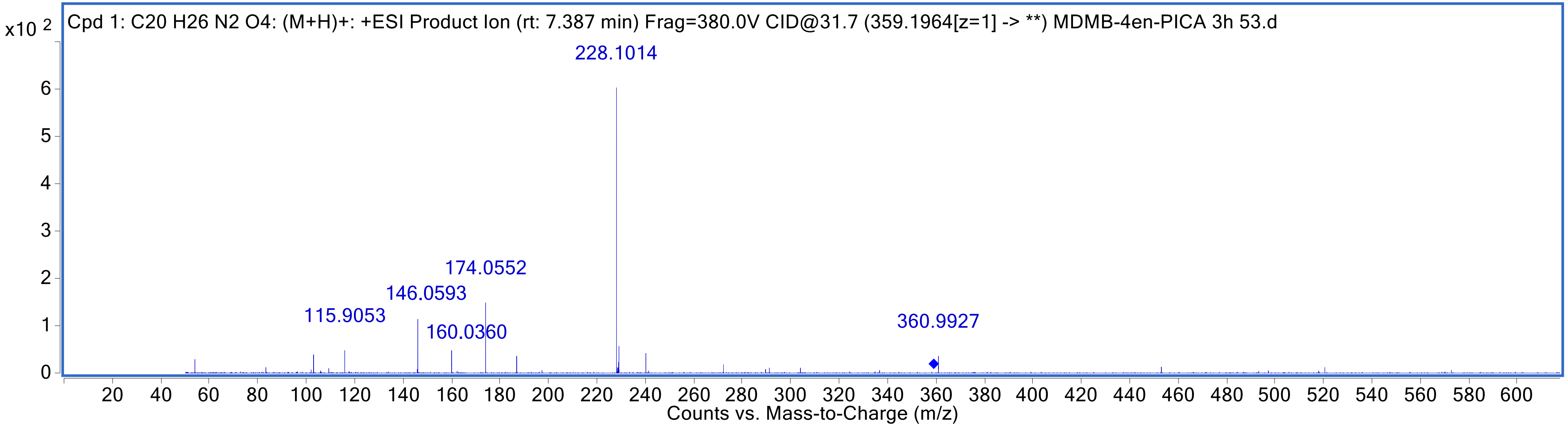

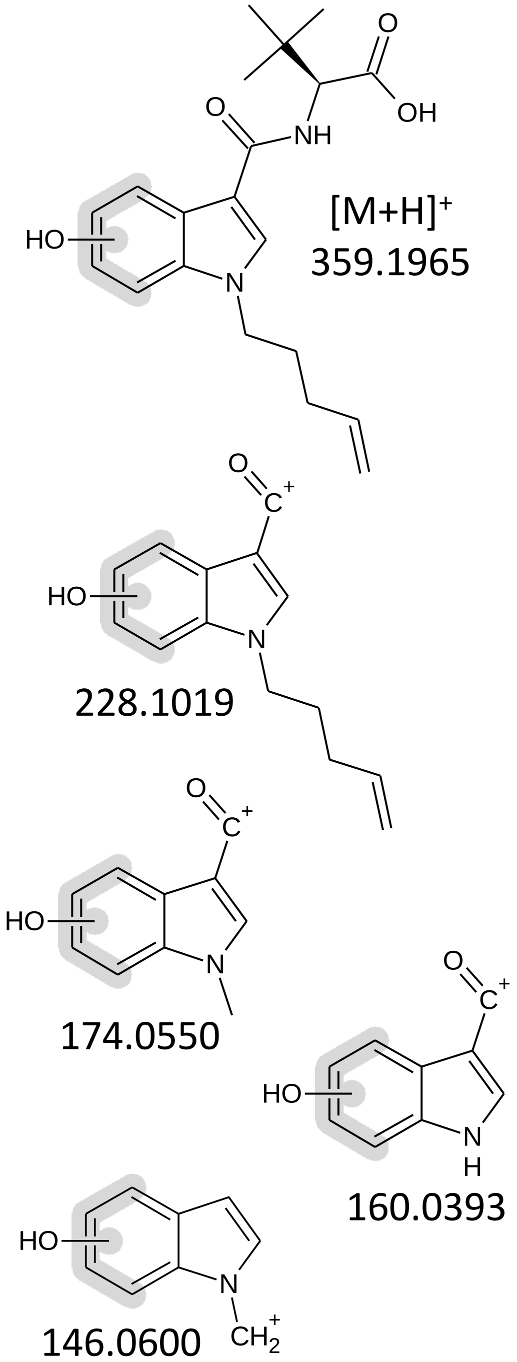

E19, Mono-hydroxylation (pentenyl tail), RT 8.41 min, *m/z* 373.2125

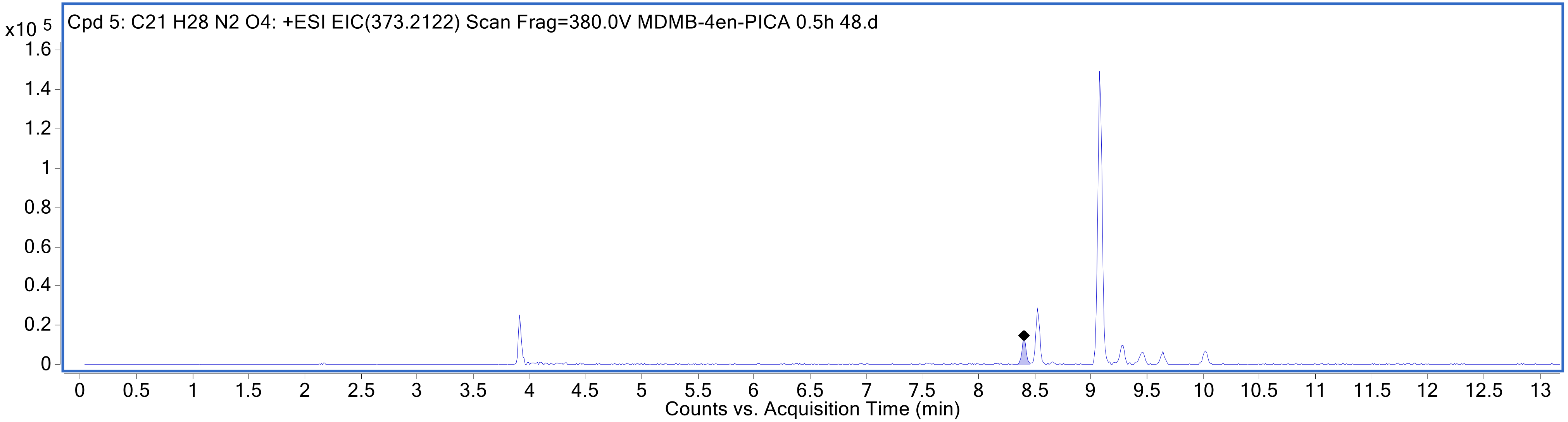

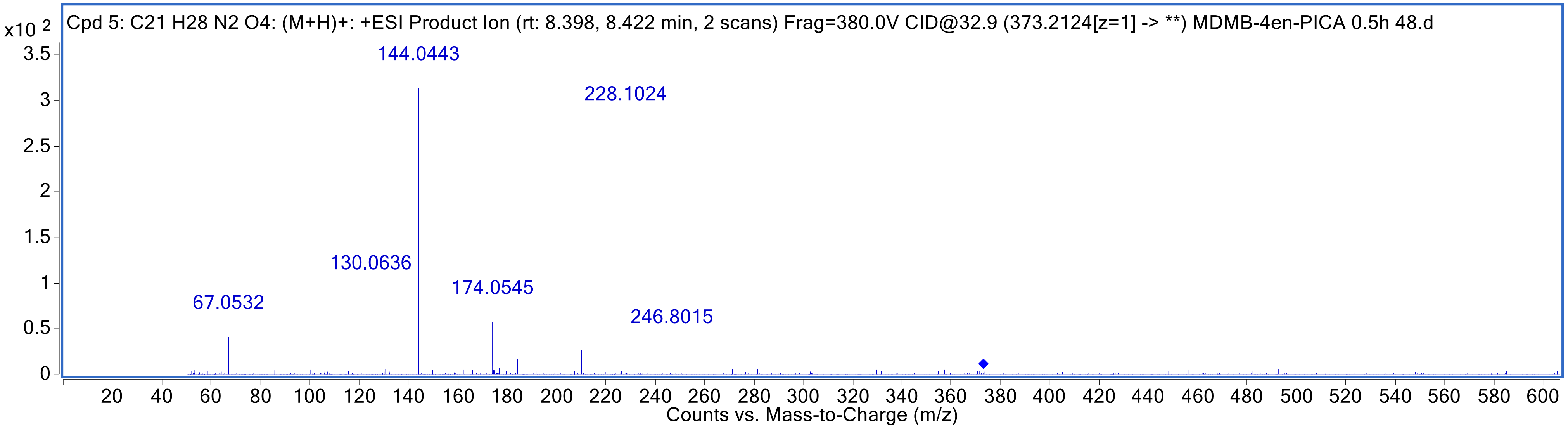

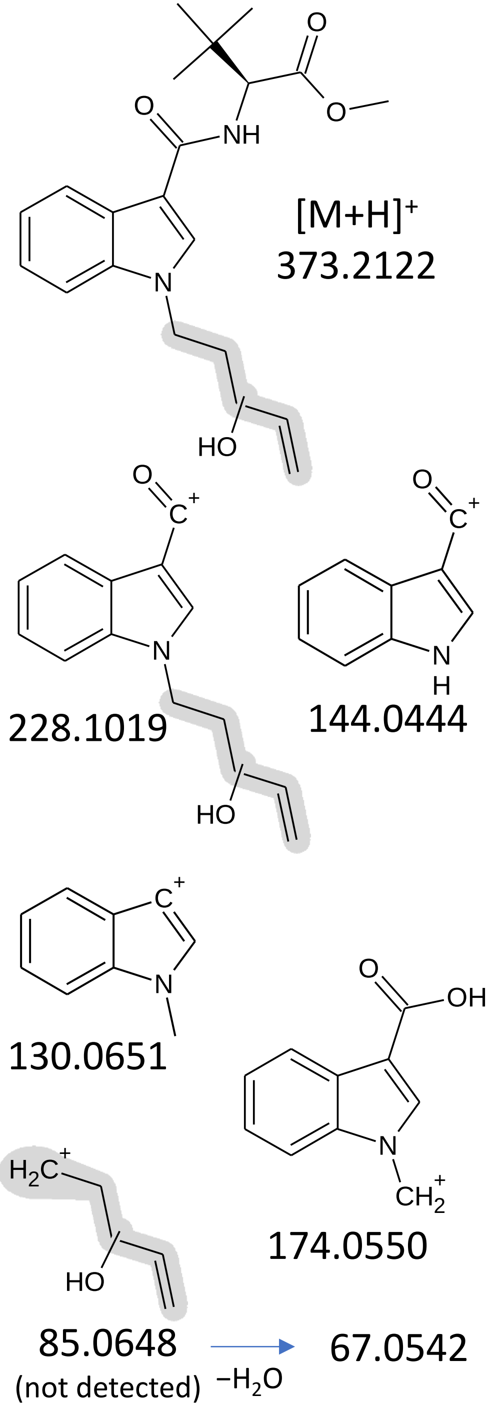

MDMB-4en-PINACA

Metabolism

MDMB-4en-PINACA, RT 13.02 min, *m/z* 358.2161

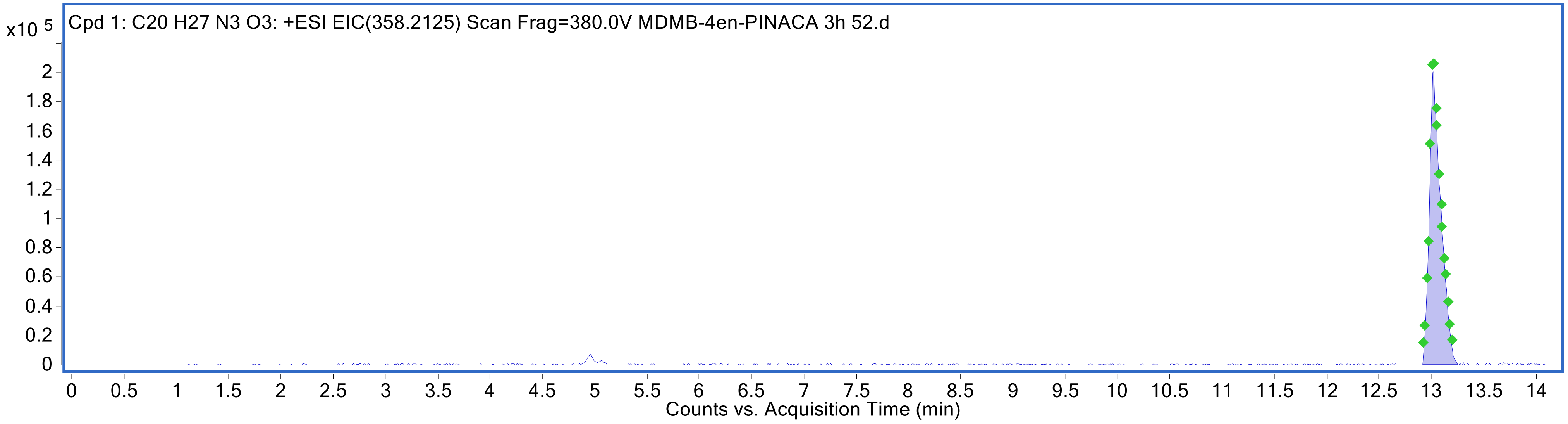

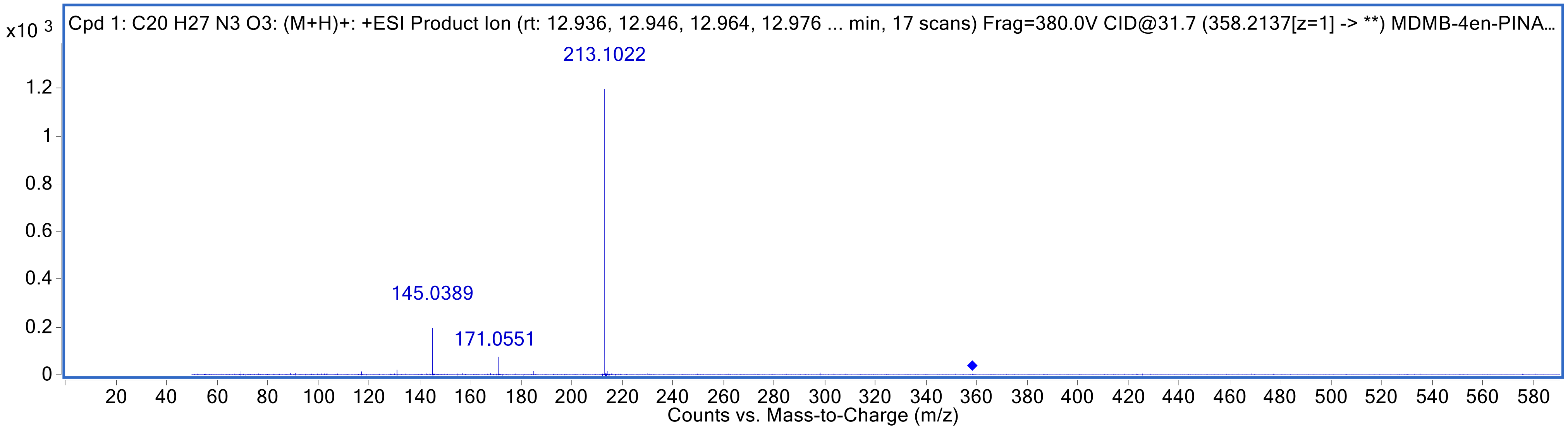

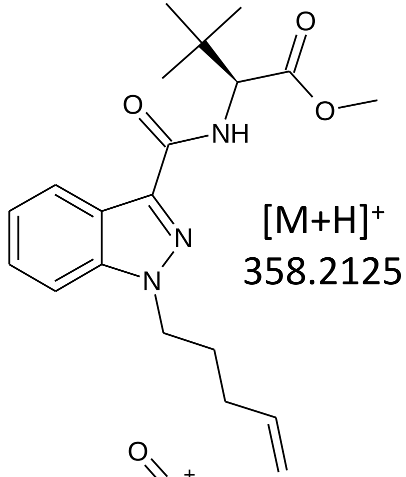

$[M+H]^+$
358.2125

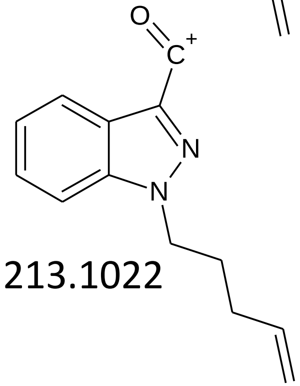

213.1022

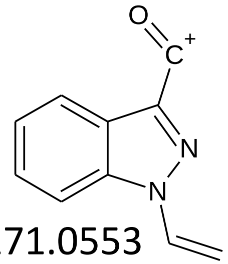

171.0553

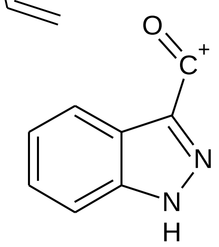

145.0396

F1, Ester hydrolysis, RT 10.59 min, *m/z* 344.1981

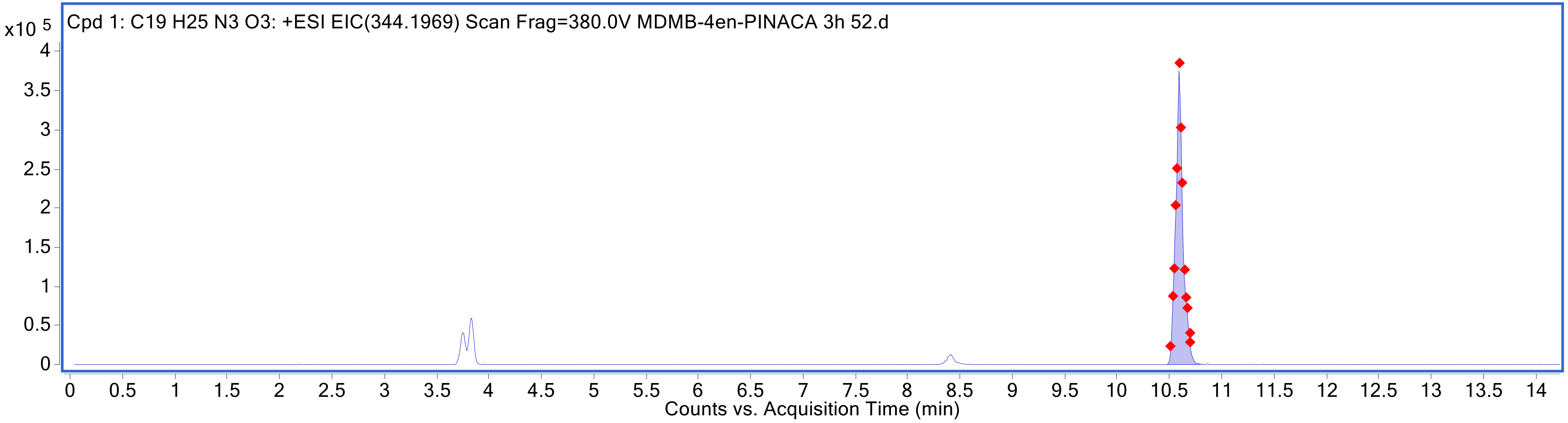

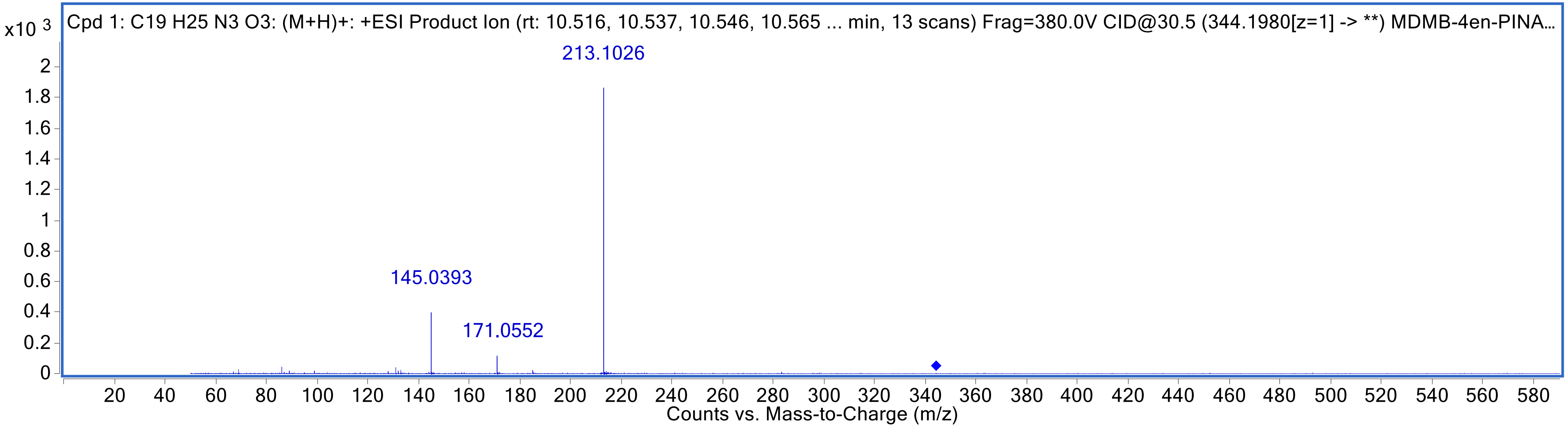

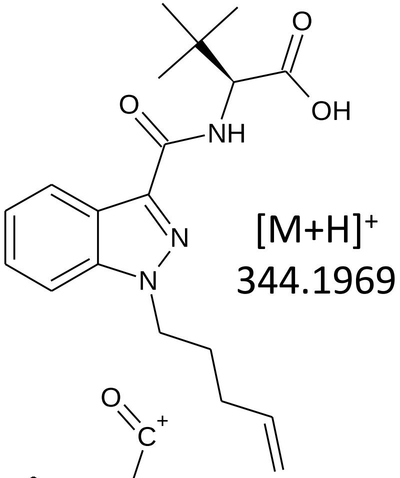

$[M+H]^+$
344.1969

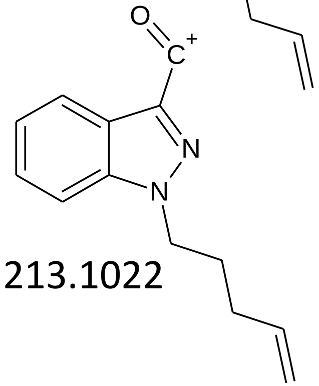

213.1022

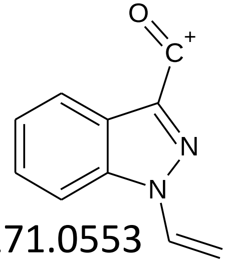

171.0553

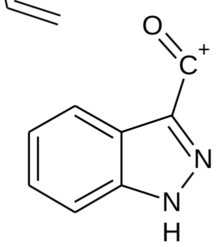

145.0396

F2, Dihydrodiol formation, RT 7.29 min, *m/z* 392.2188

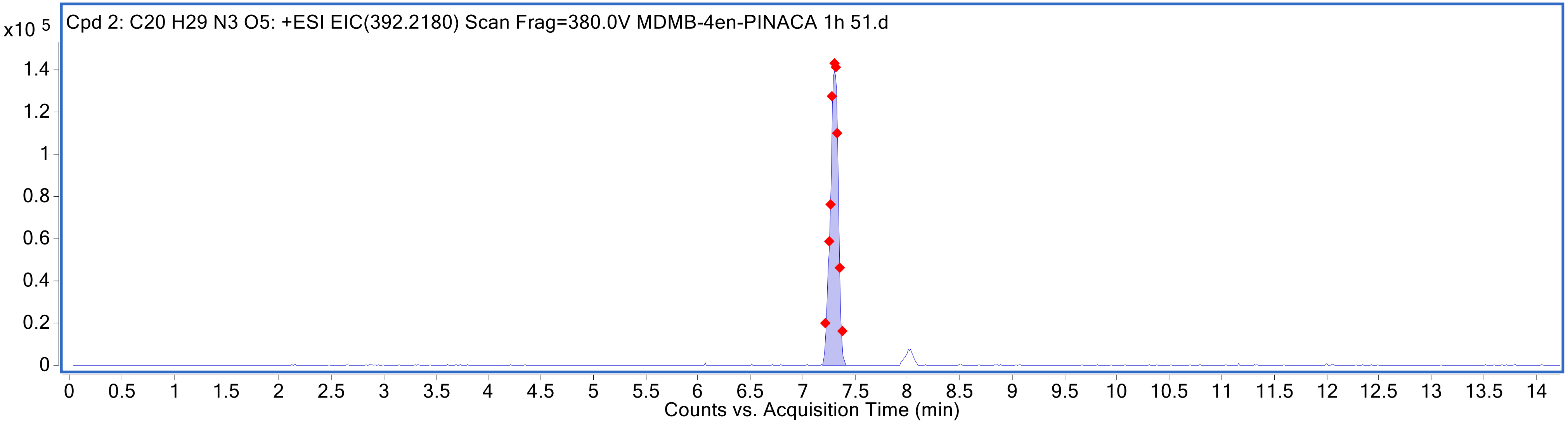

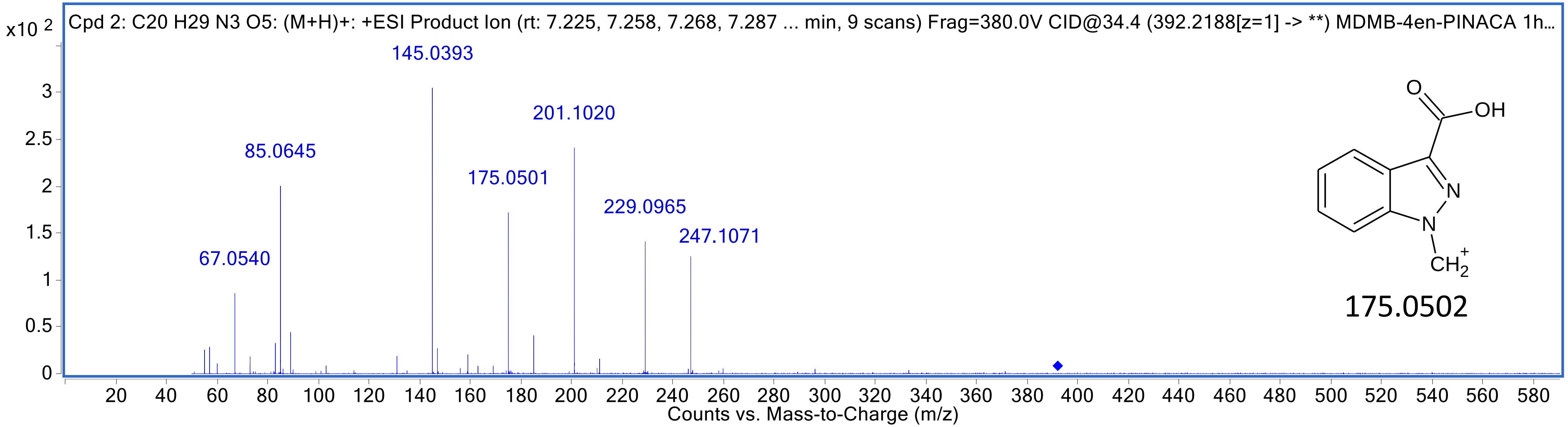

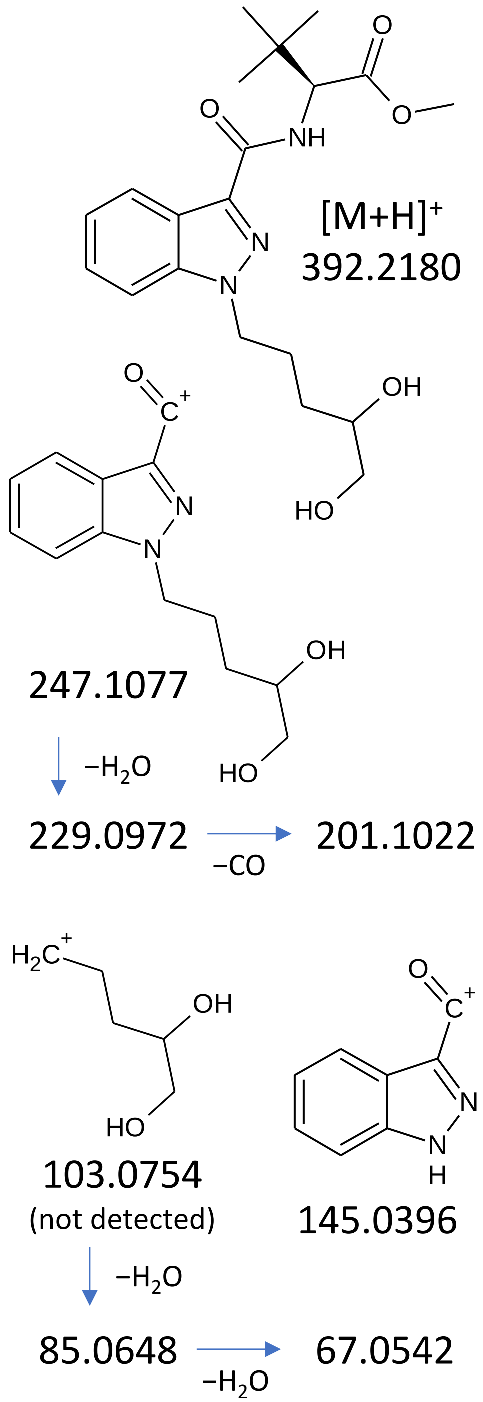

Dihydrodiol reference standard, RT 7.29 min, *m/z* 392.2190

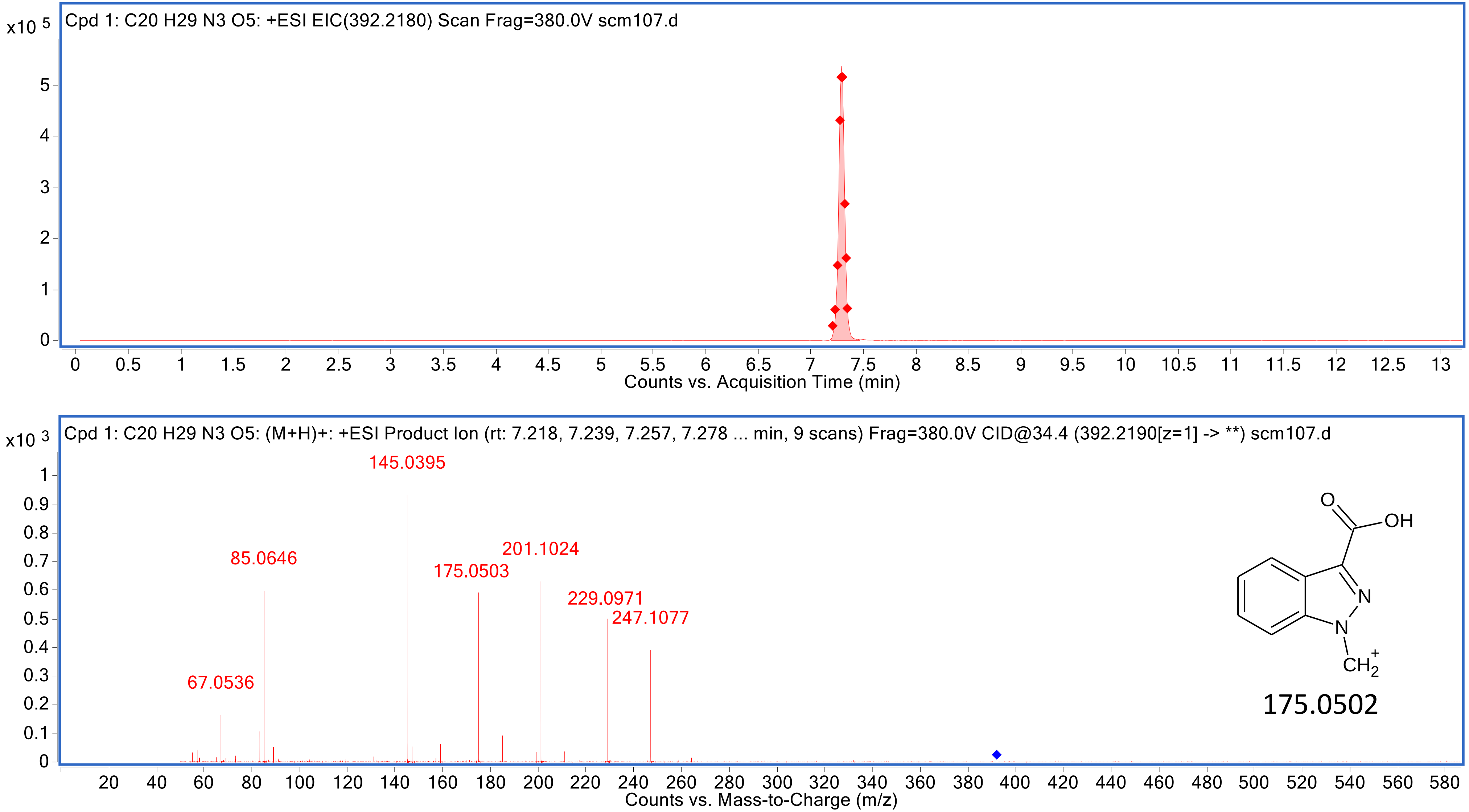

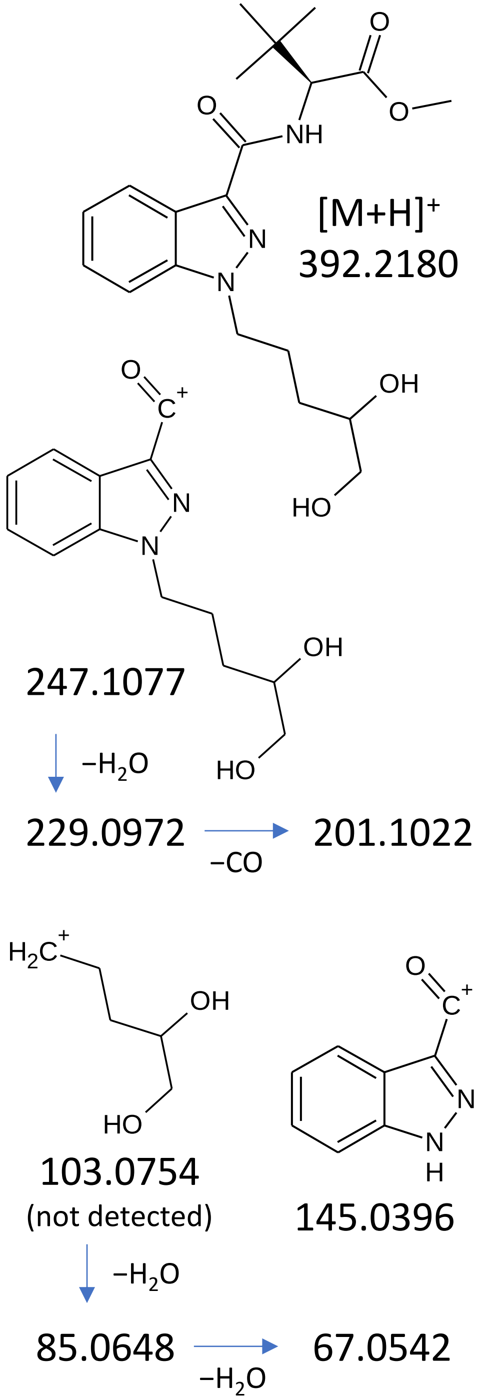

F3, Ester hydrolysis + dihydrodiol formation, RT 5.62 min, *m/z* 378.2026

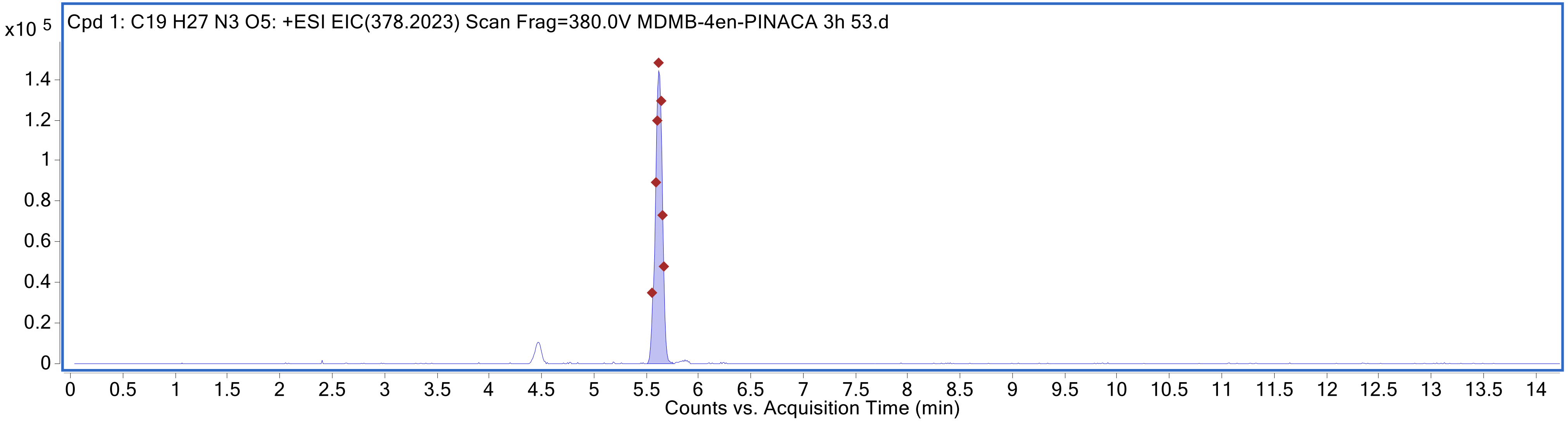

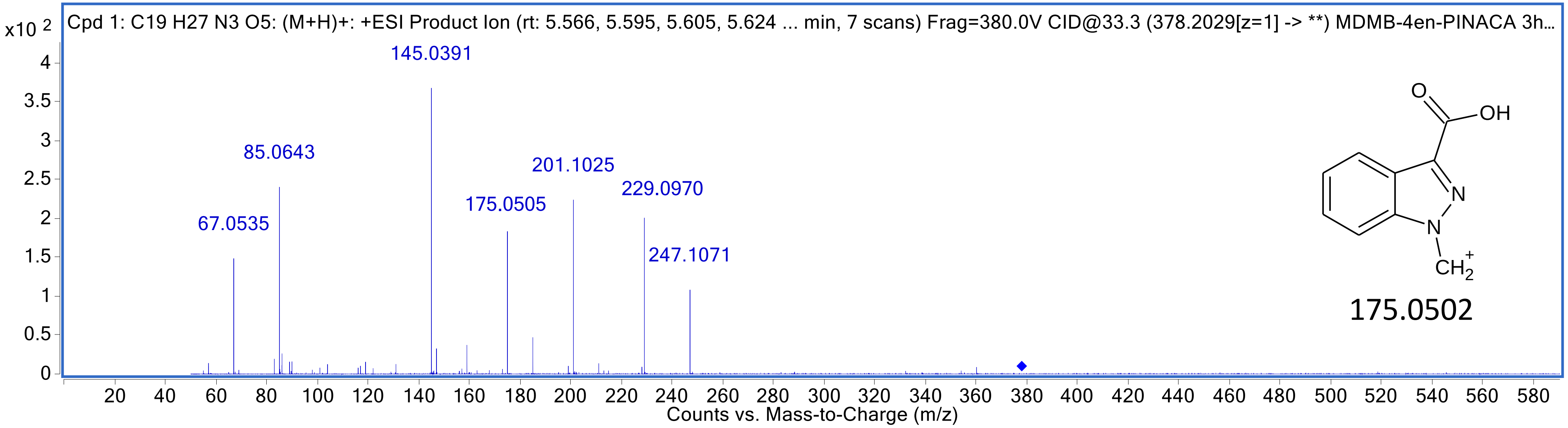

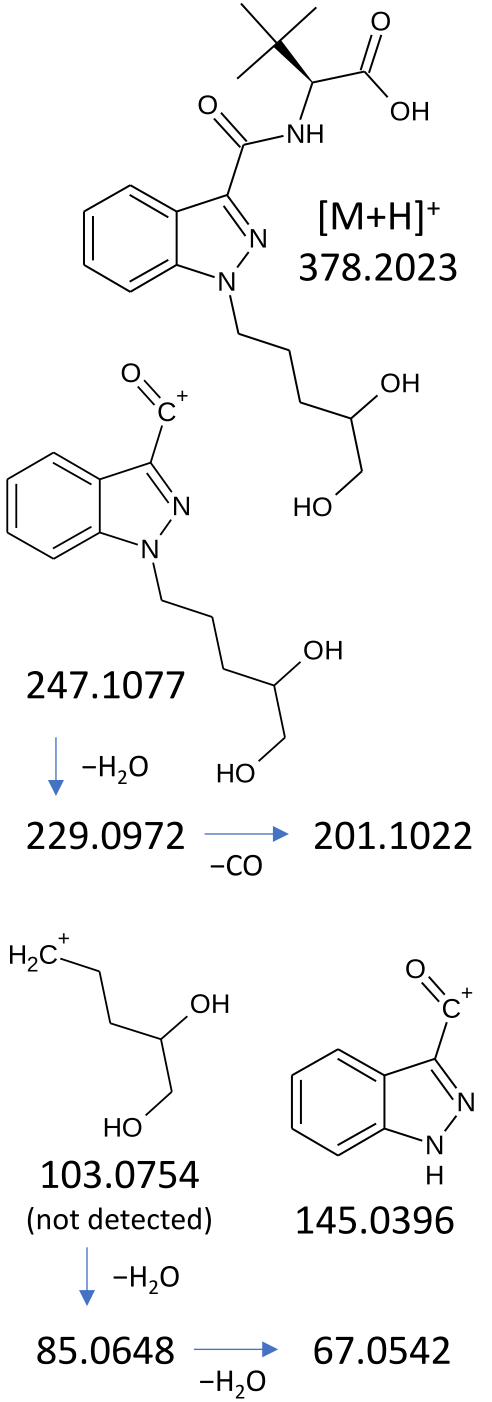

F4, Ester hydrolysis + dihydrodiol formation + dehydrogenation, RT 5.14 min, *m/z* 376.1870

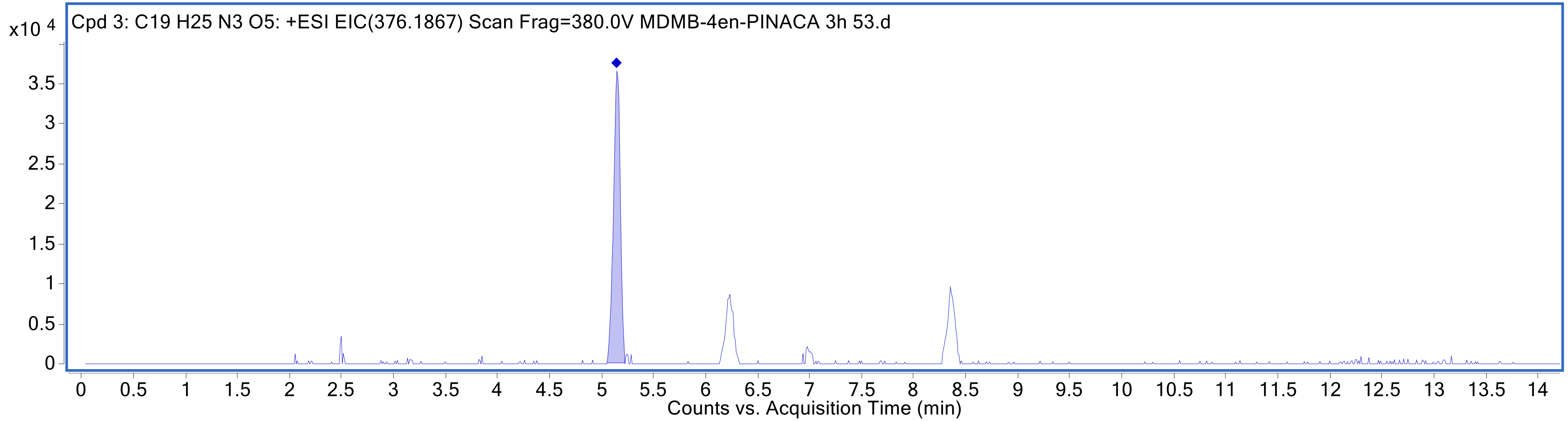

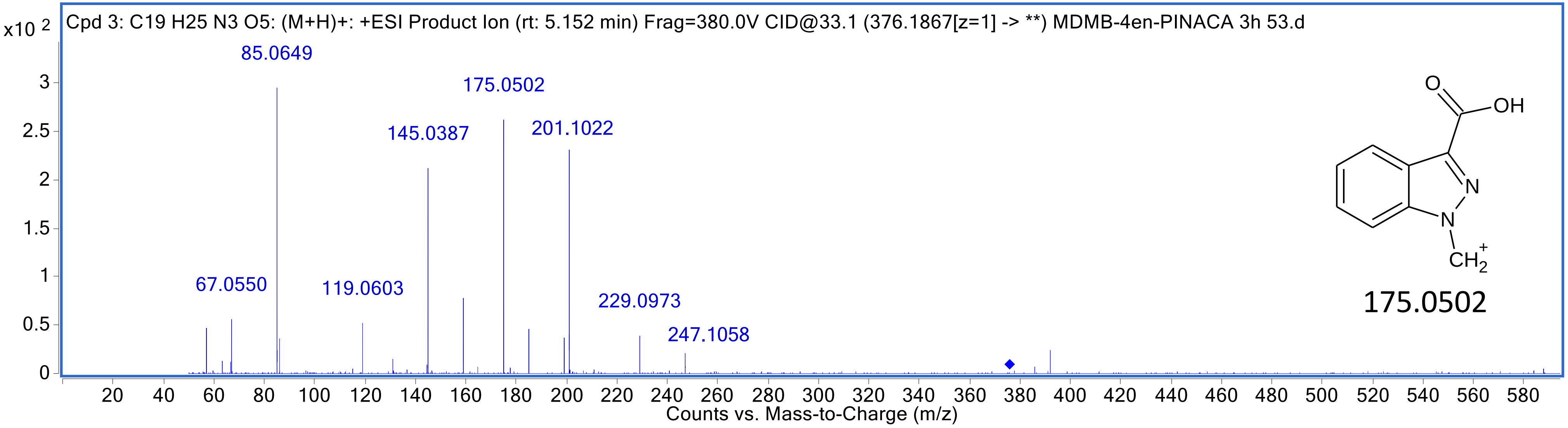

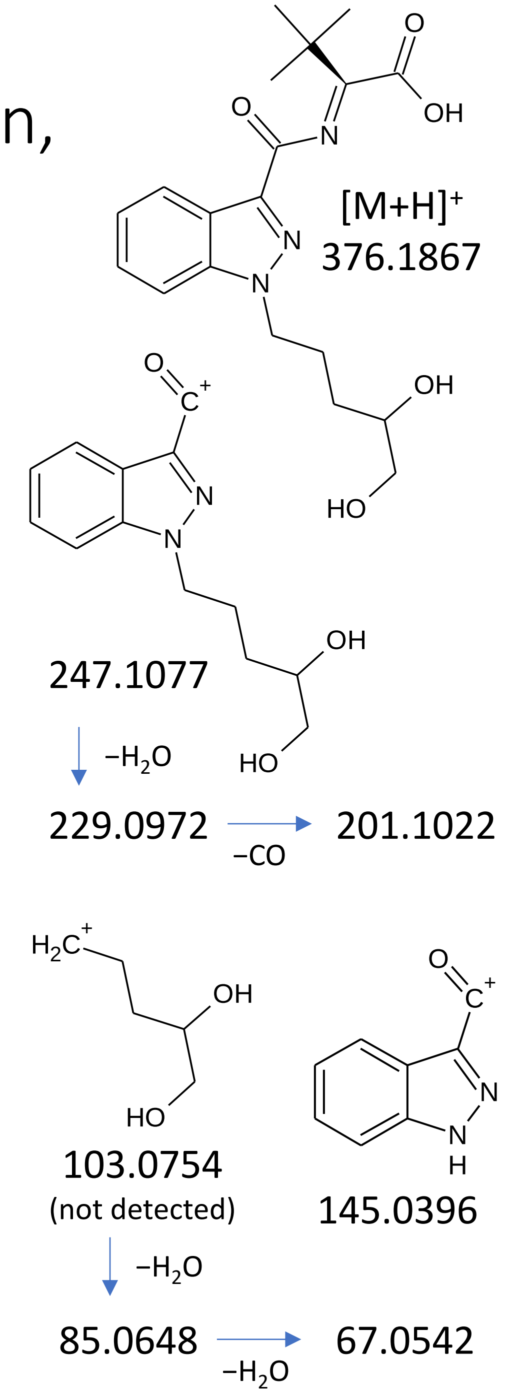

F5, Mono-hydroxylation (pentenyl tail), RT 9.59 min, *m/z* 374.2077

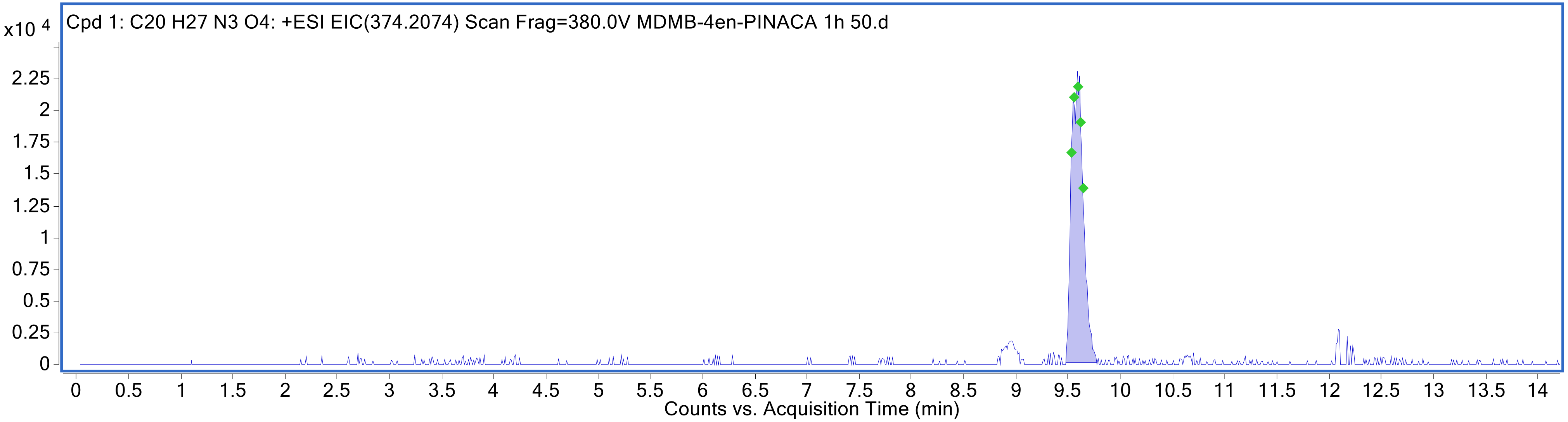

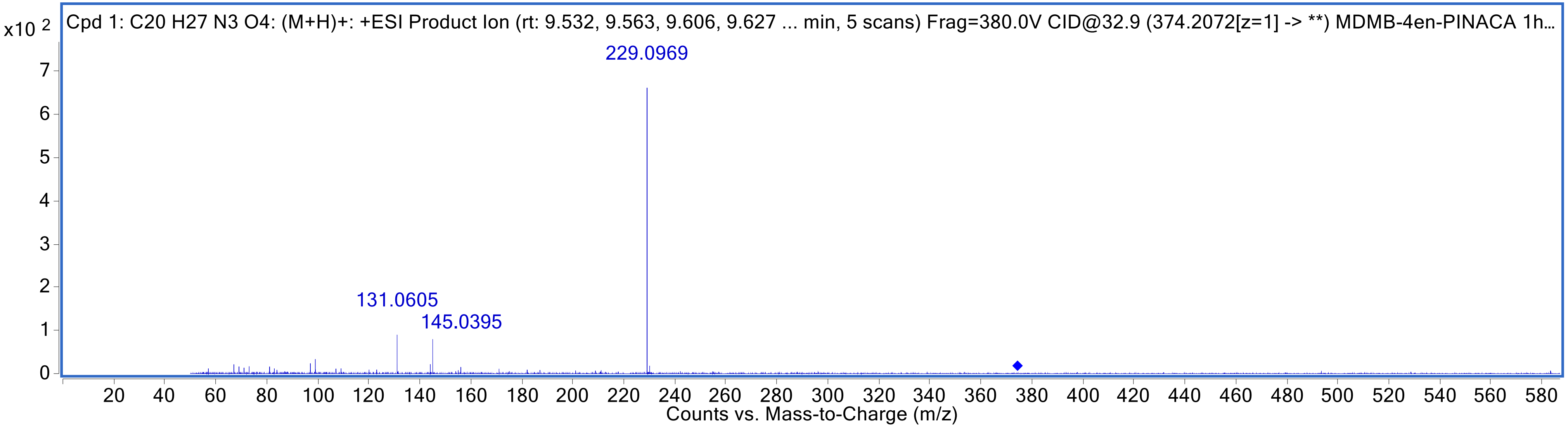

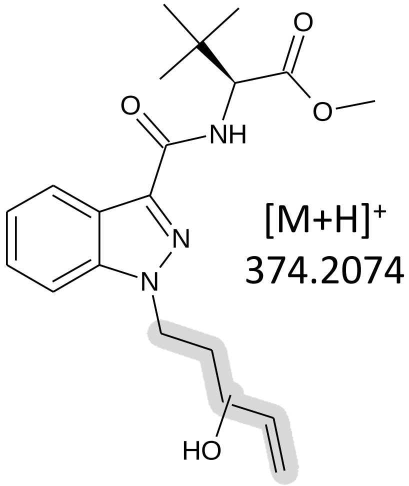

$[M+H]^+$
374.2074

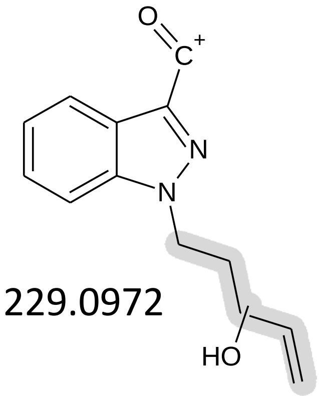

229.0972

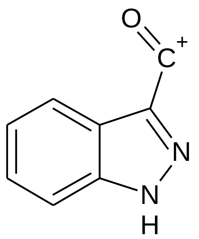

145.0396

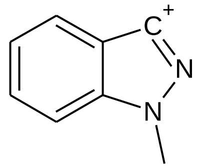

131.0604

F6, Ester hydrolysis + mono-hydroxylation (pentenyl tail), RT 7.39 min, *m/z* 360.1913

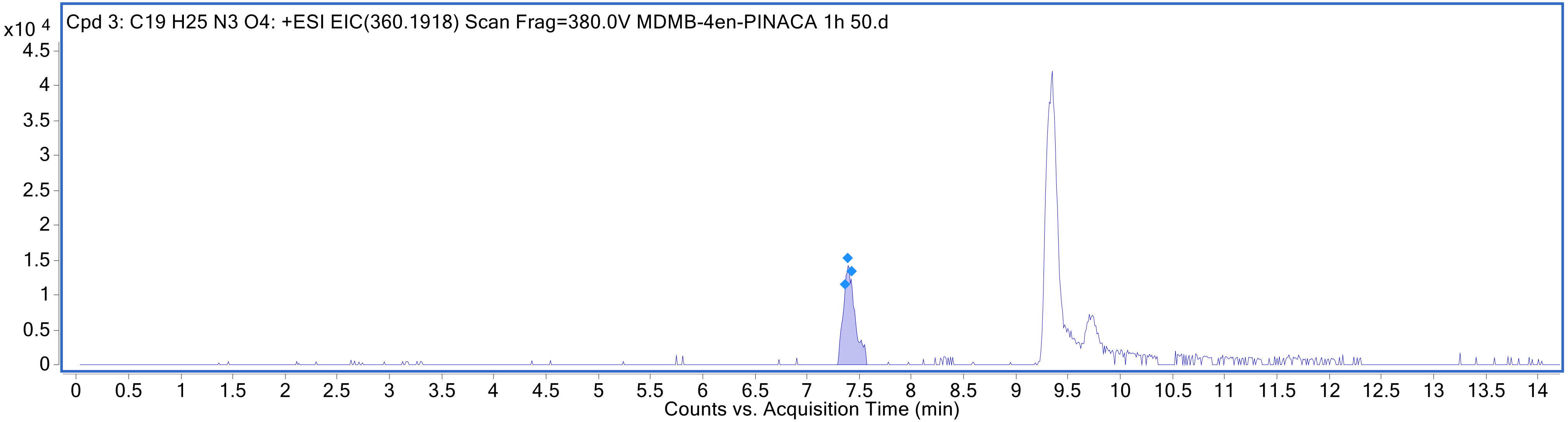

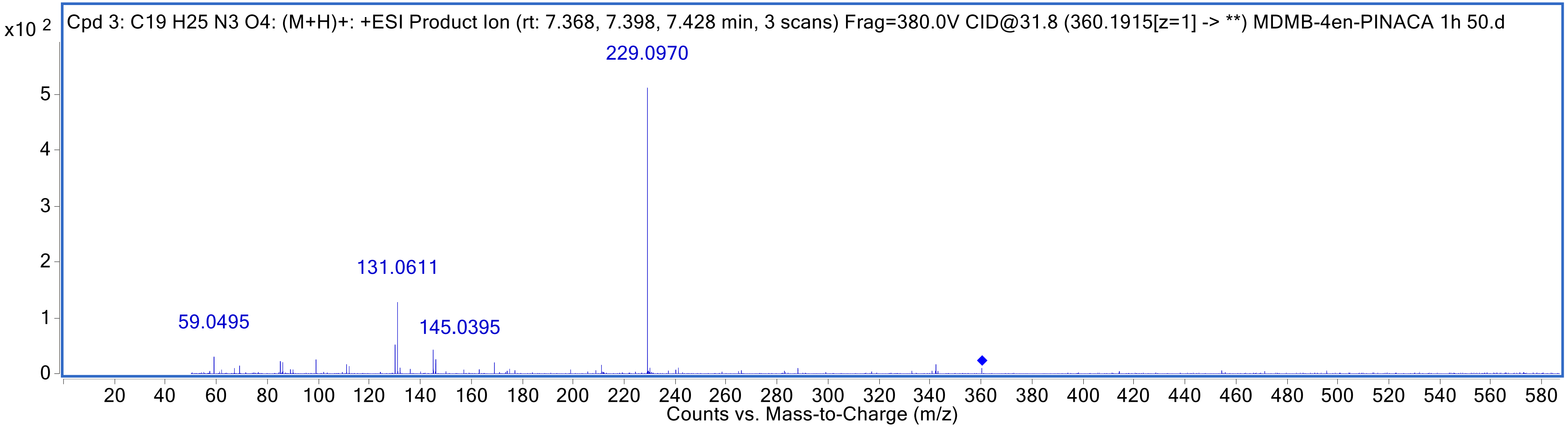

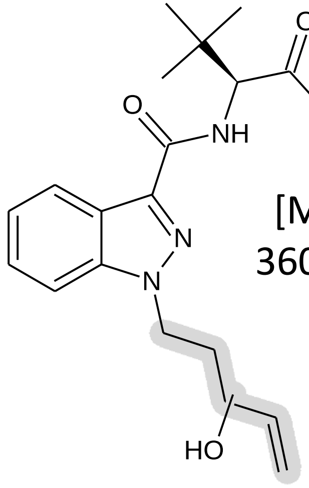

[M+H]+
360.1918

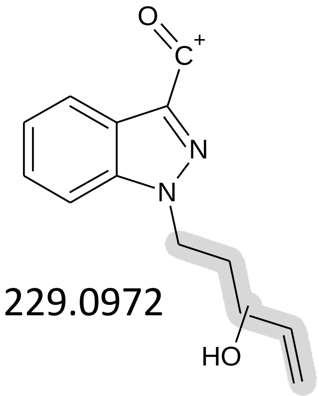

229.0972

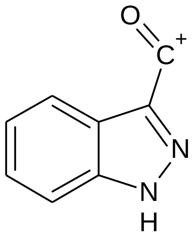

145.0396

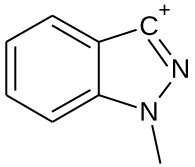

131.0604

F7, Ester hydrolysis + glucuronidation, RT 8.41 min, *m/z* 520.2287

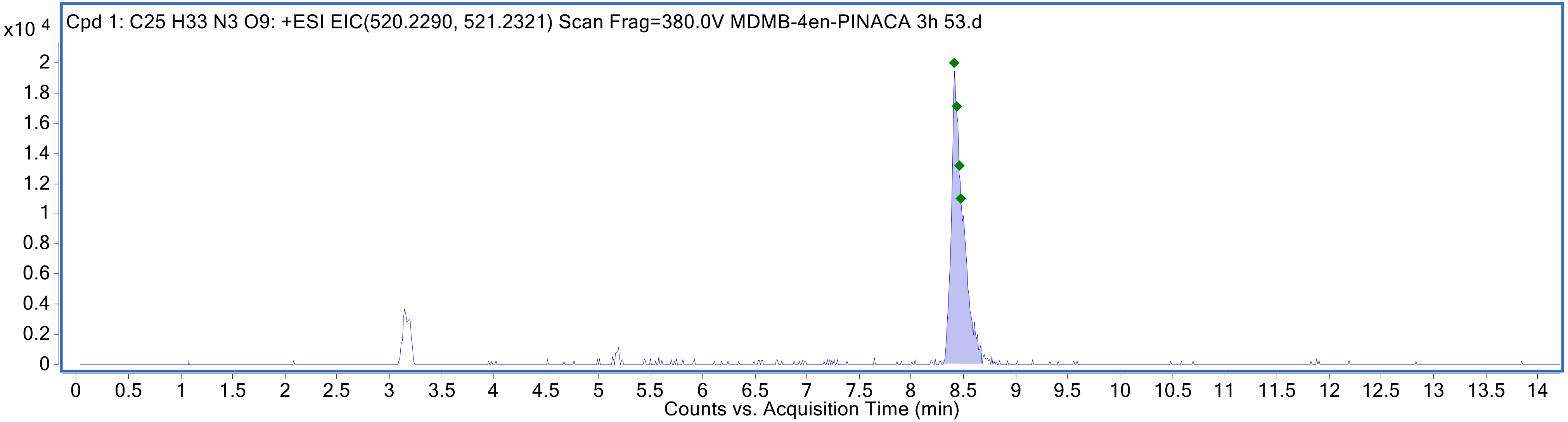

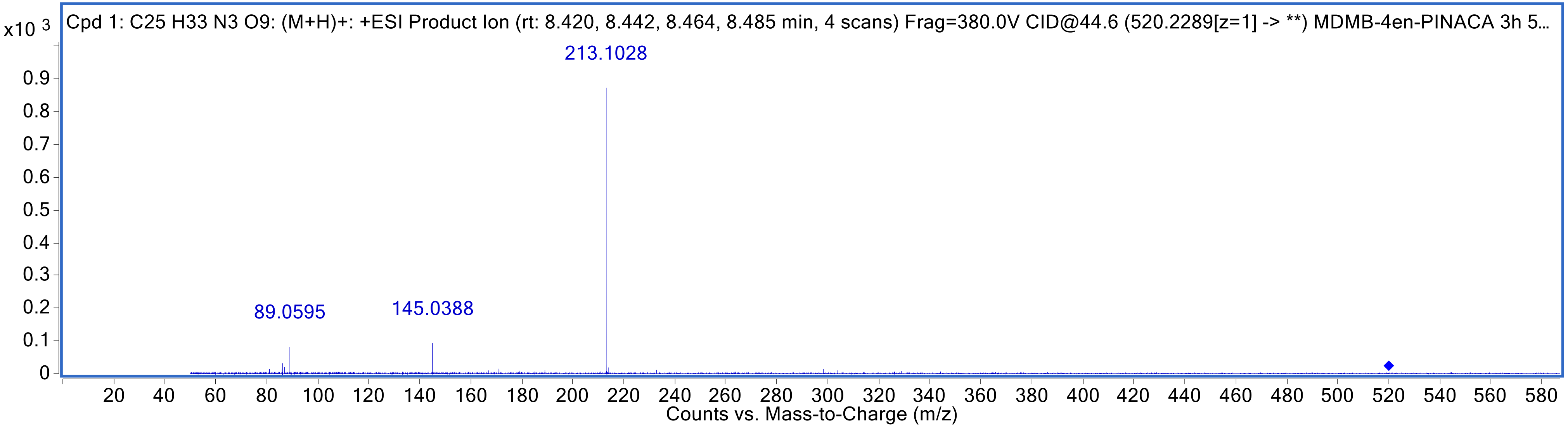

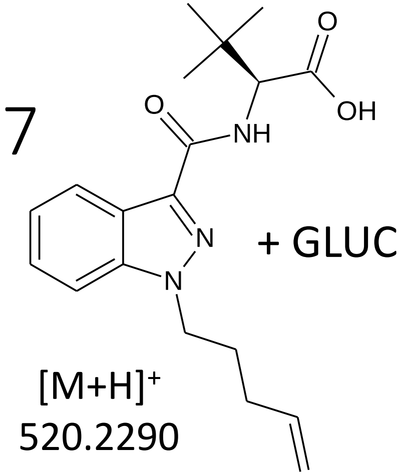

$[M+H]^+$
520.2290

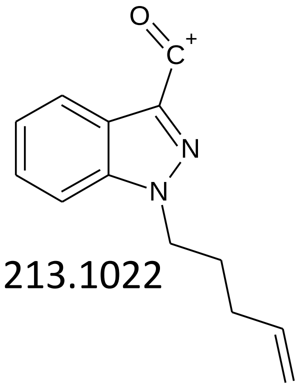

213.1022

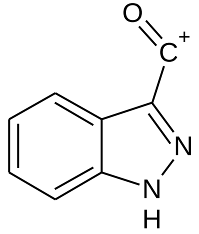

145.0396

F8, Dihydrodiol formation + glucuronidation, RT 6.02 min, *m/z* 568.2497

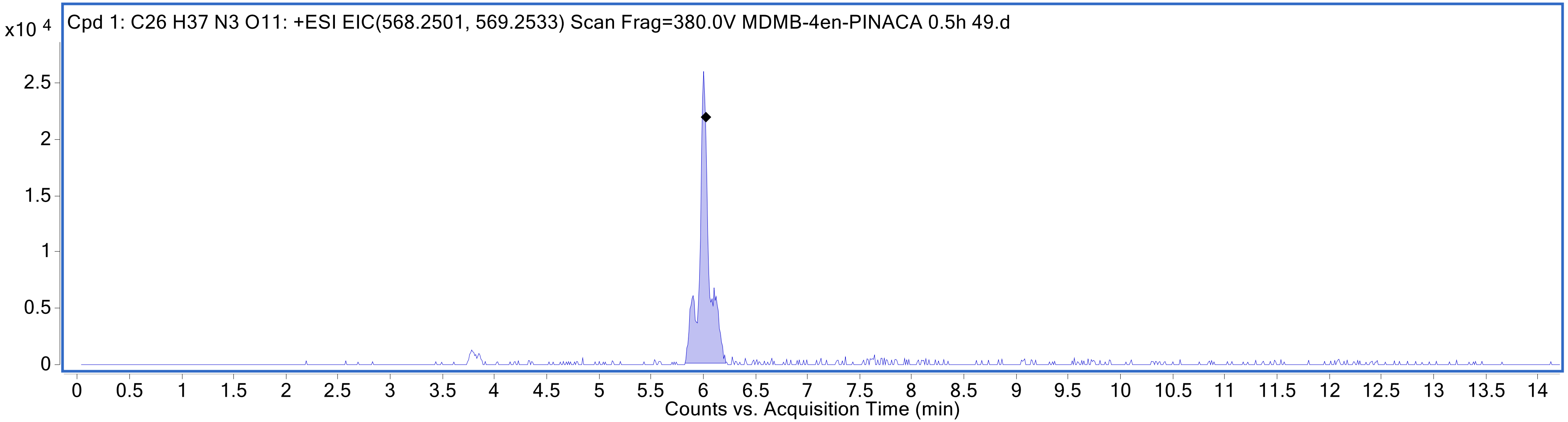

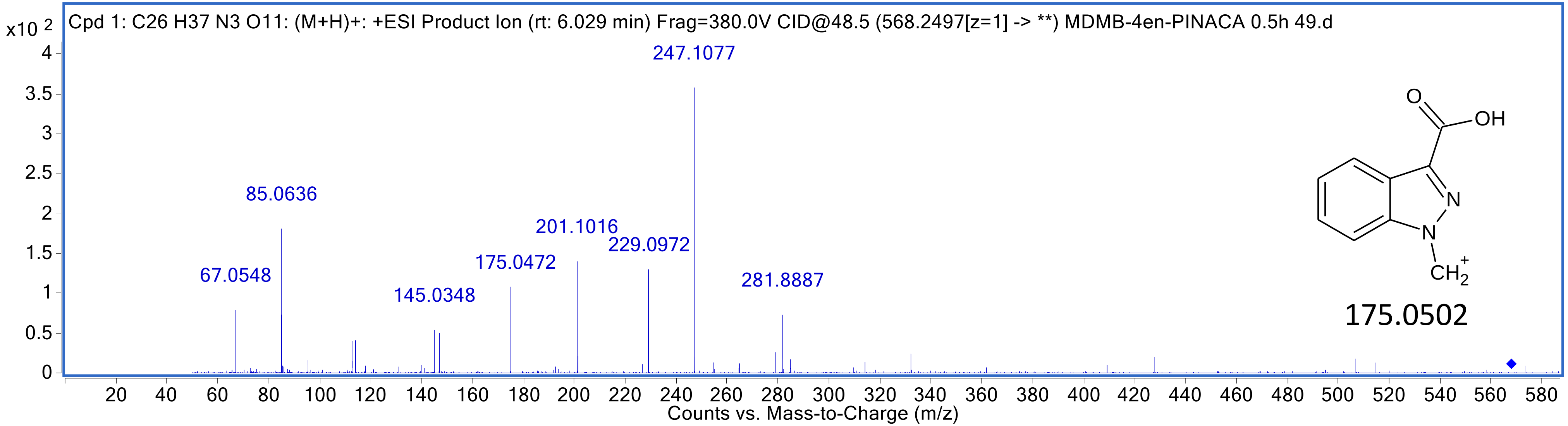

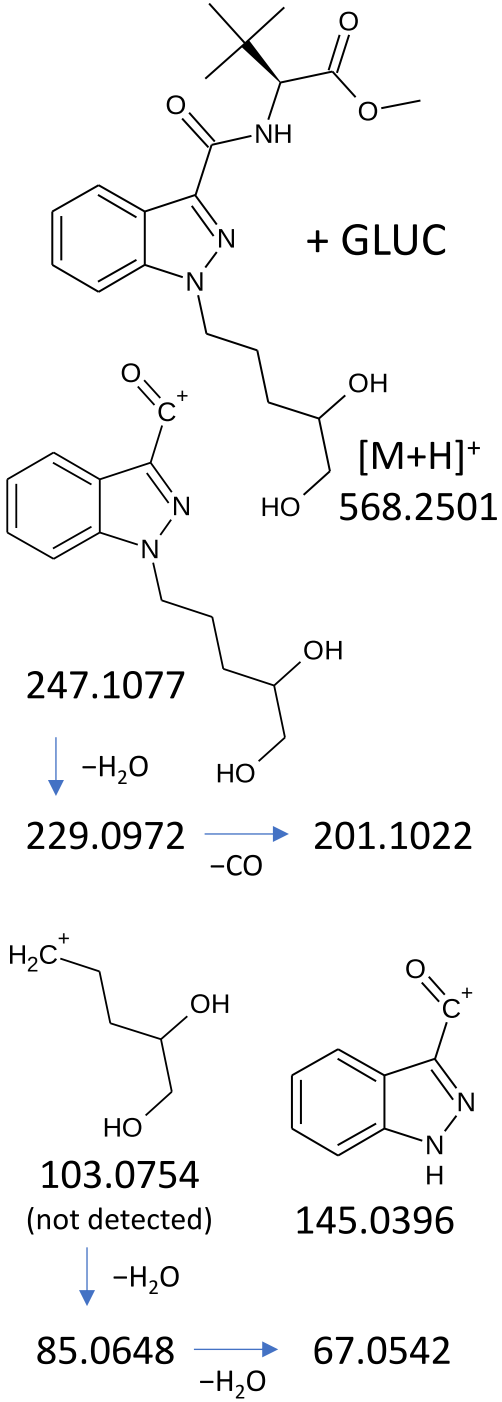

F9, Ester hydrolysis + dihydrodiol formation + glucuronidation, RT 4.47 min, *m/z* 554.2337

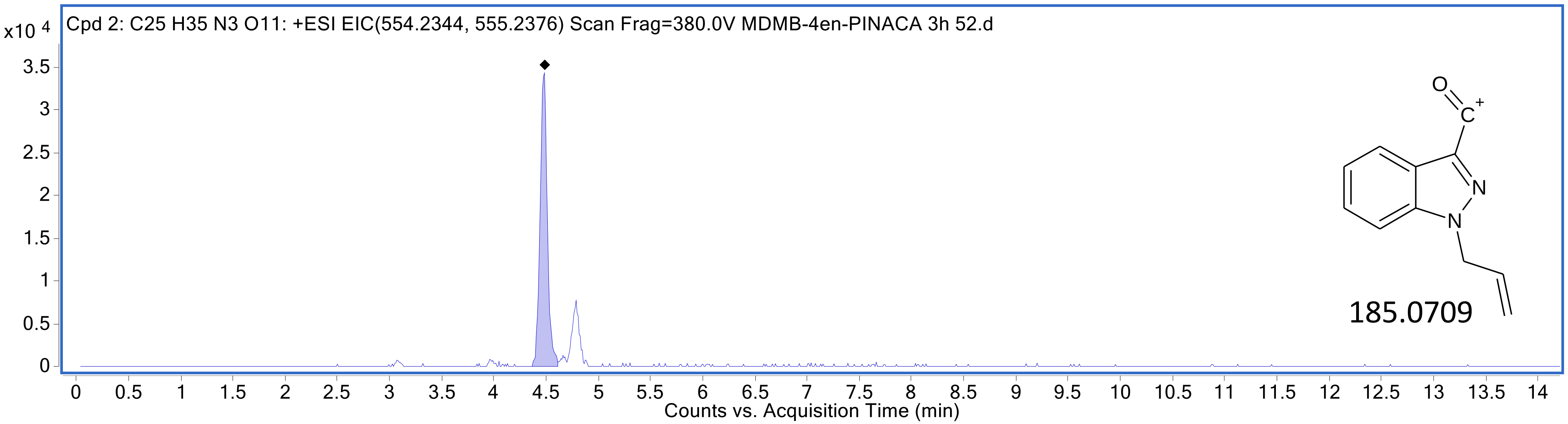

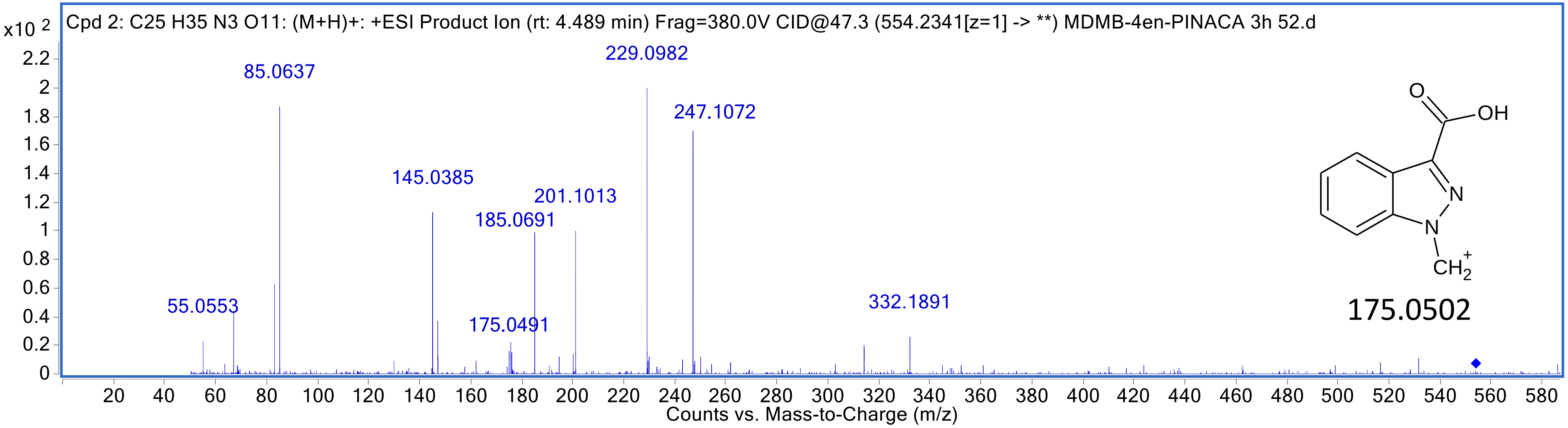

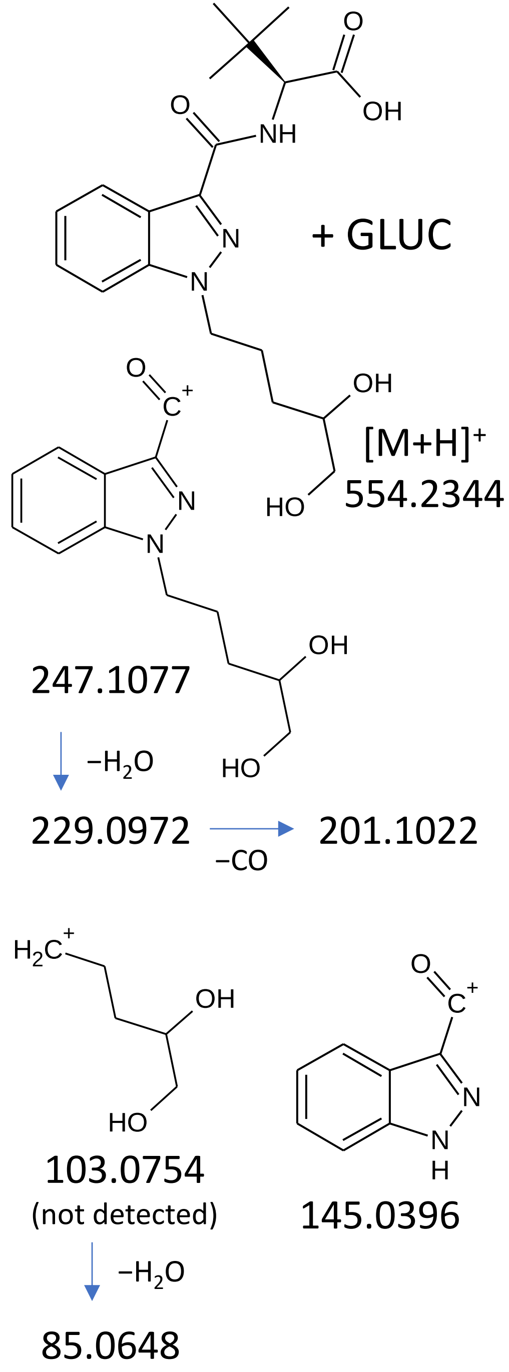

AB-4en-PICA

Metabolism

AB-4en-PICA, RT 8.14 min, *m/z* 328.2034

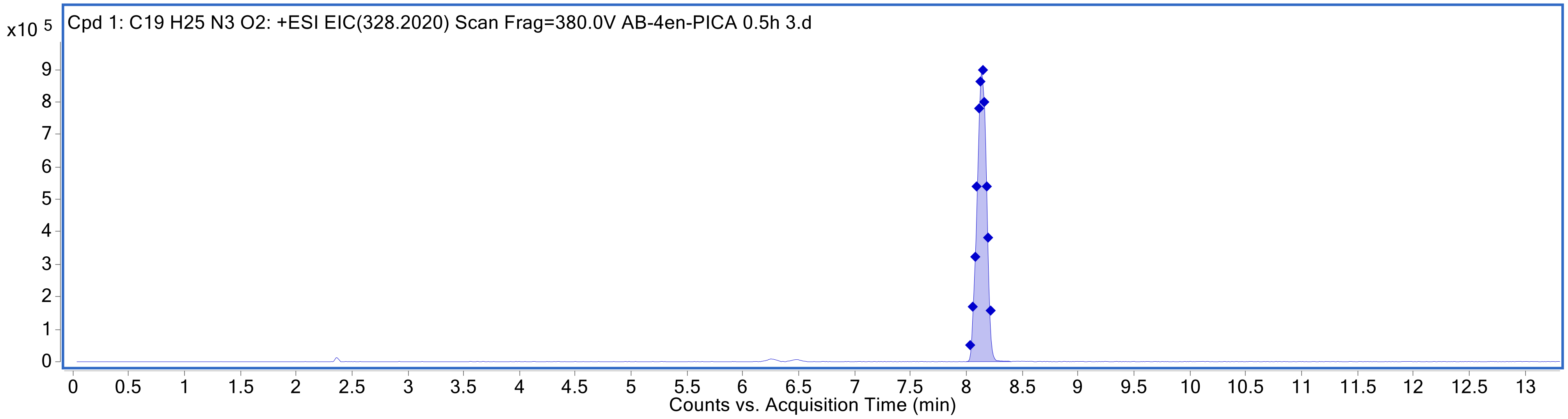

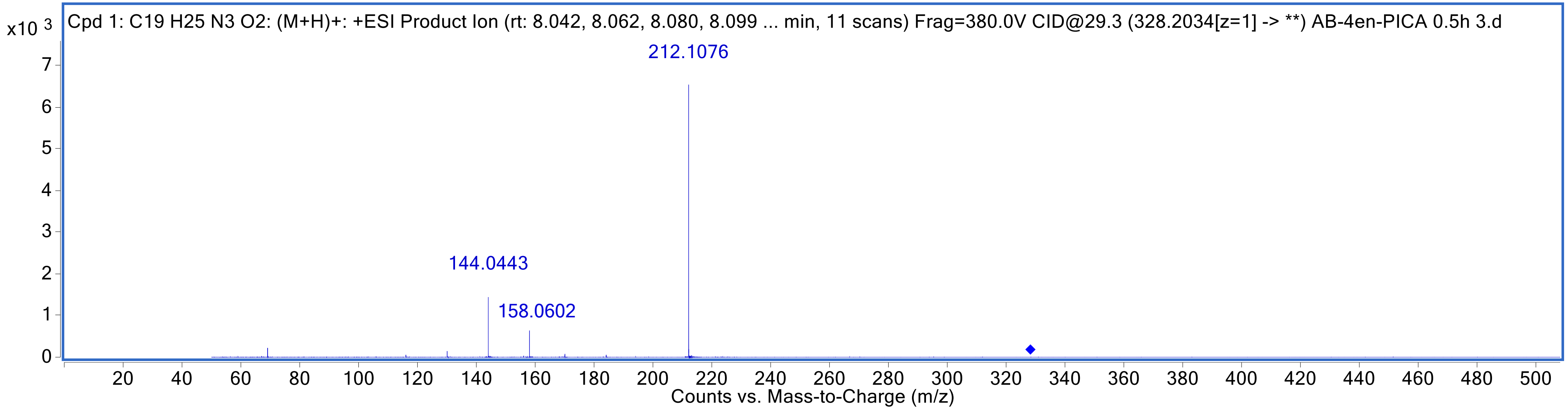

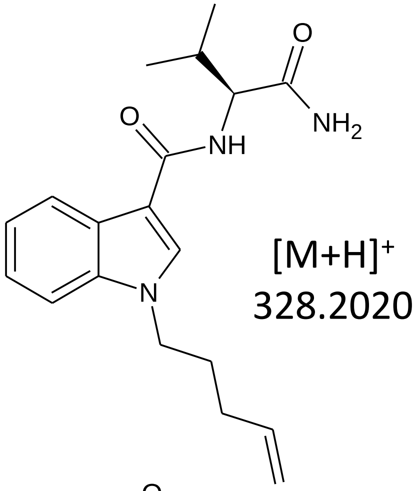

$[M+H]^+$
328.2020

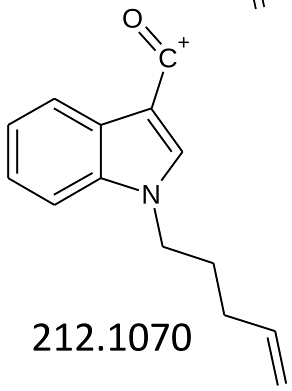

212.1070

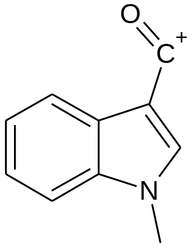

158.0600

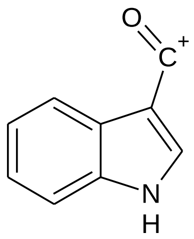

144.0444

G1, Mono-hydroxylation (pentenyl tail), RT 5.45 min, *m/z* 344.1973

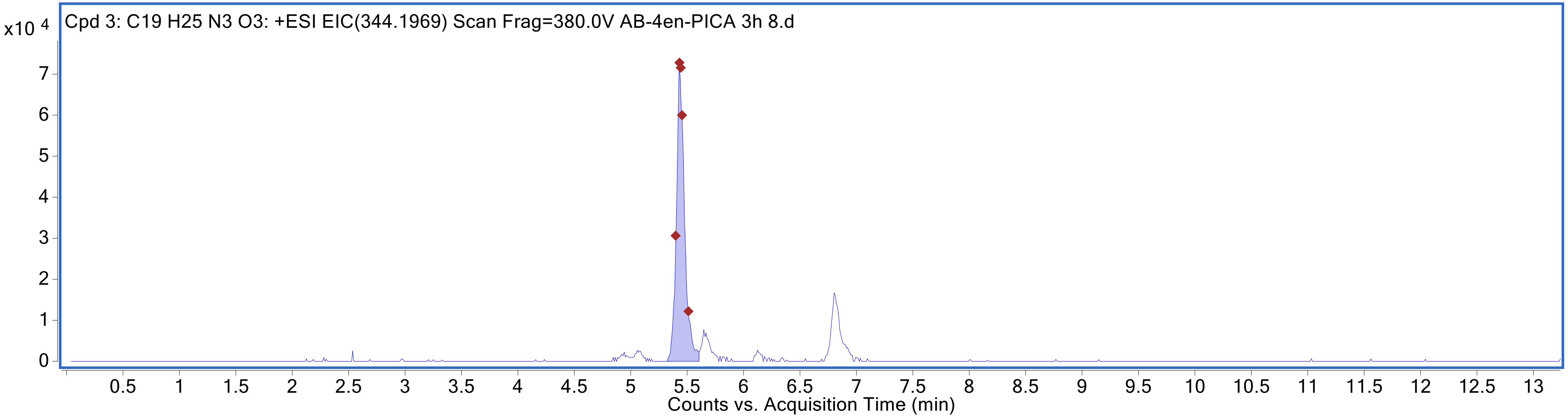

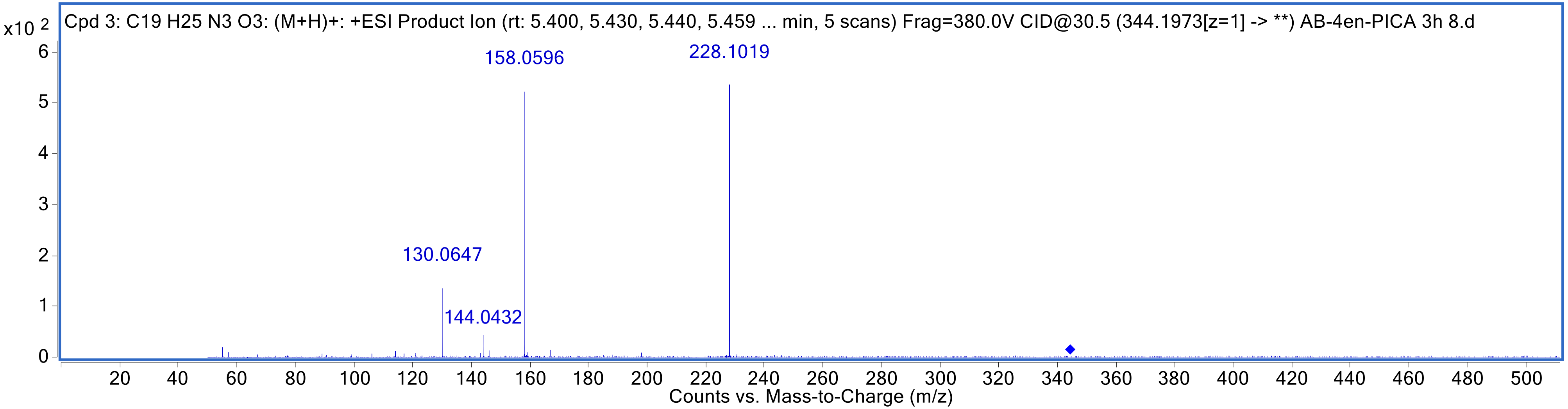

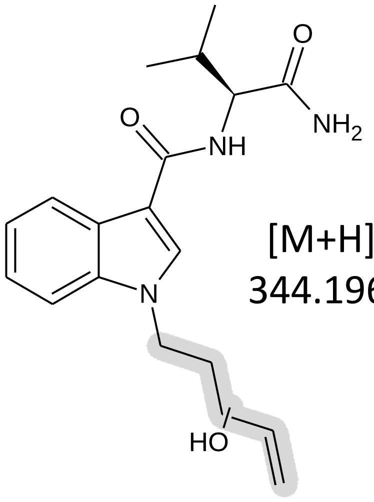

[M+H]+
344.1969

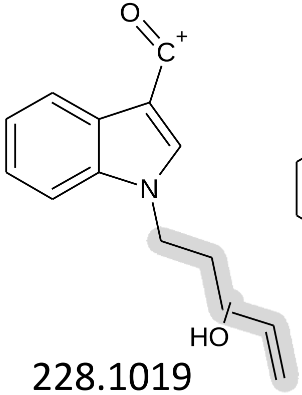

228.1019

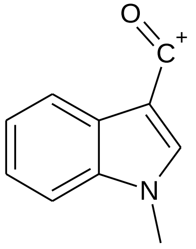

158.0600

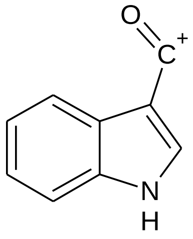

144.0444

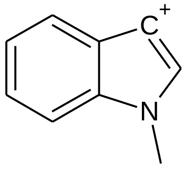

130.0651

G2, Terminal amide hydrolysis, RT 9.08 min, *m/z* 329.1862

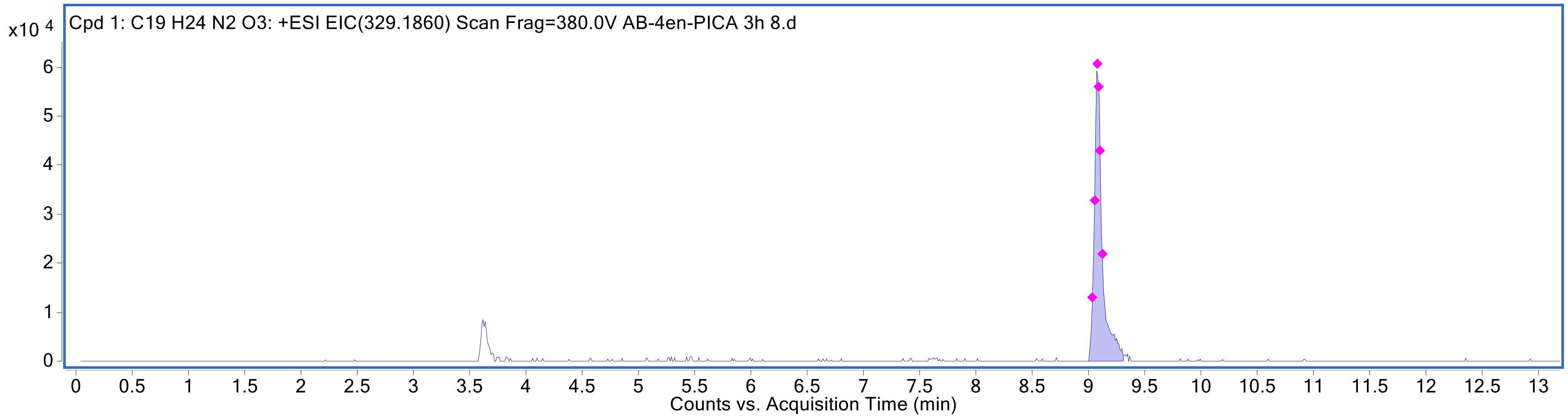

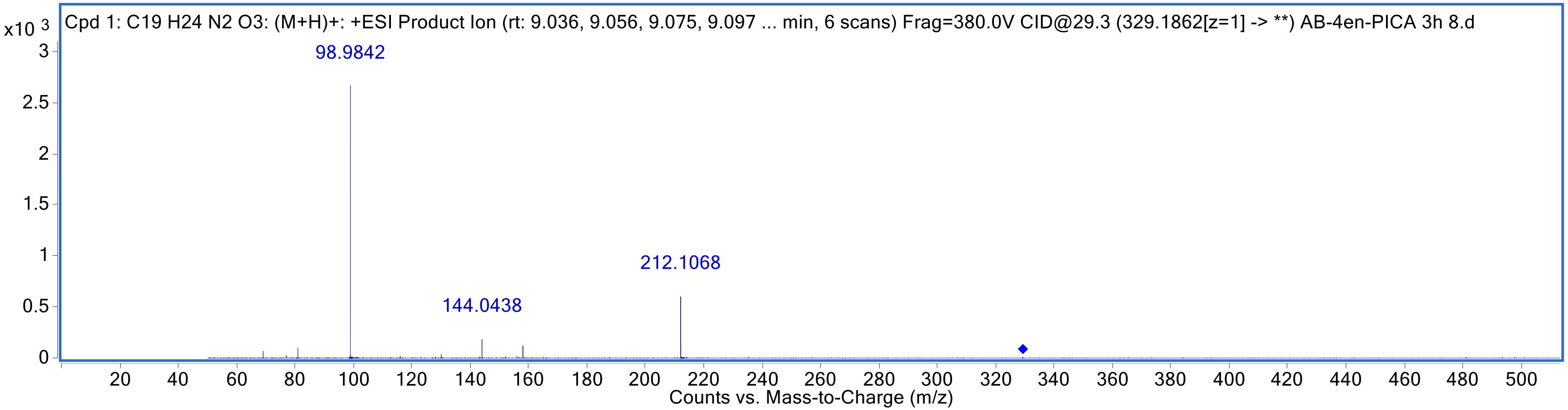

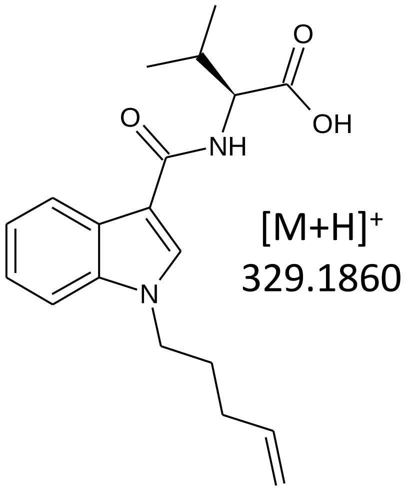

$[M+H]^+$
329.1860

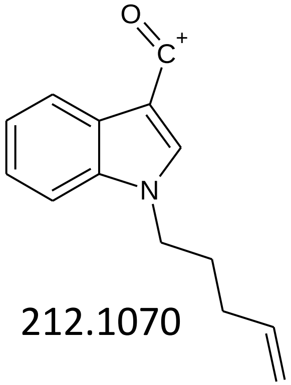

212.1070

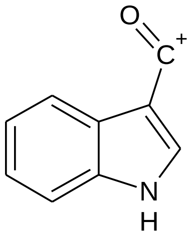

144.0444

G3, *N*-dealkylation, RT 3.95 min, *m/z* 260.1395

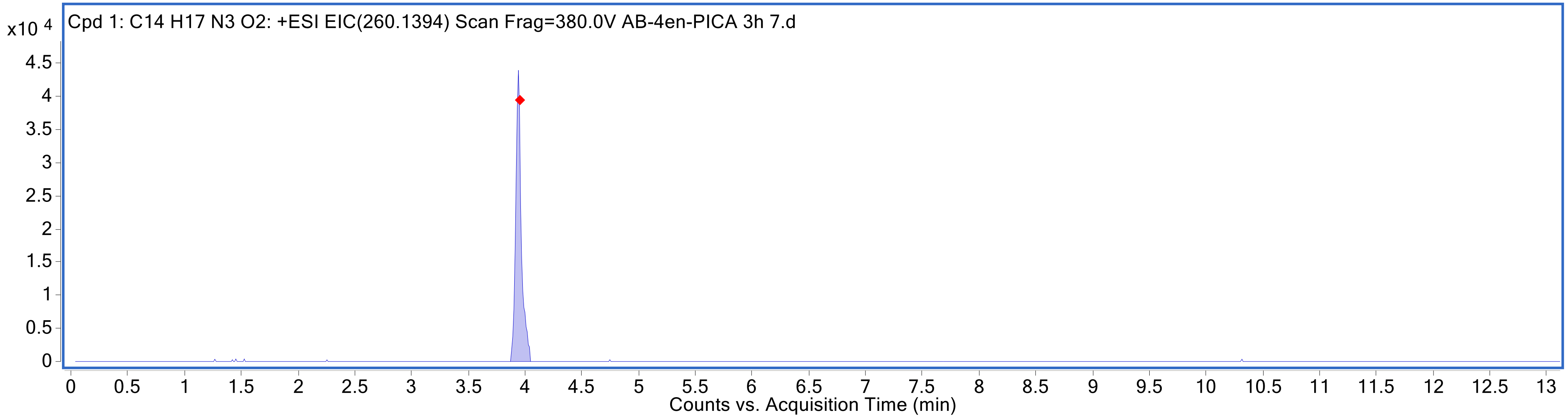

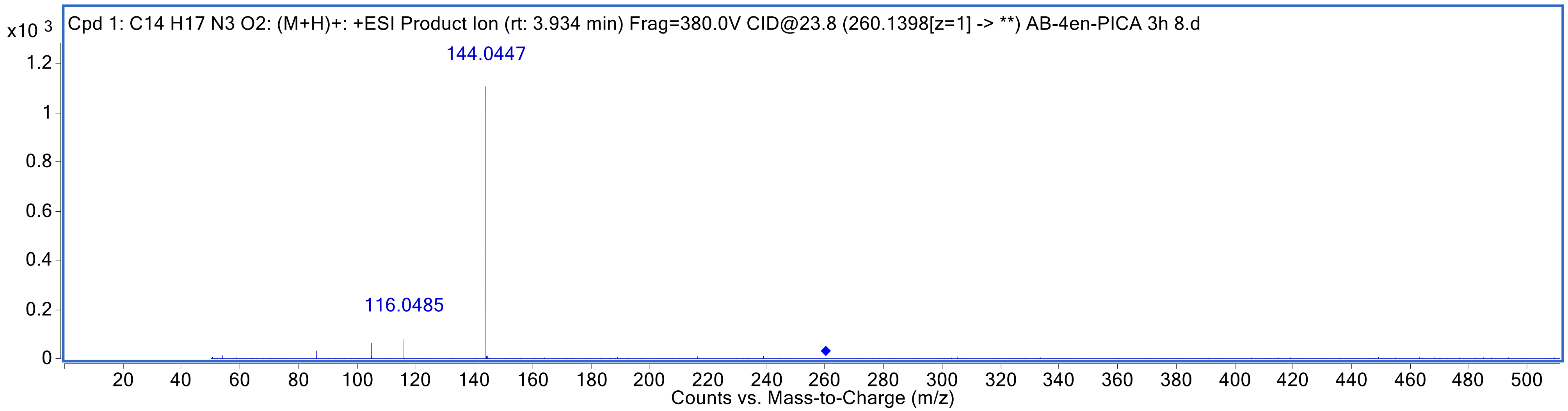

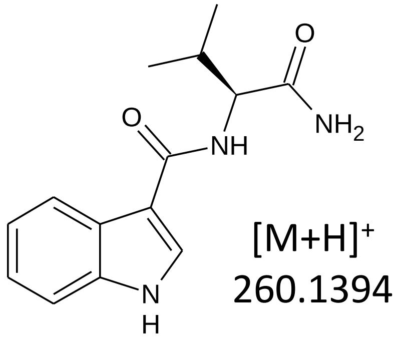

$[M+H]^+$
260.1394

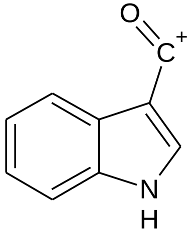

144.0444

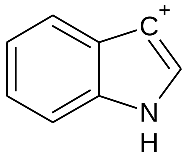

116.0495

G4, Dihydrodiol formation, RT 3.97 min, *m/z* 362.2076

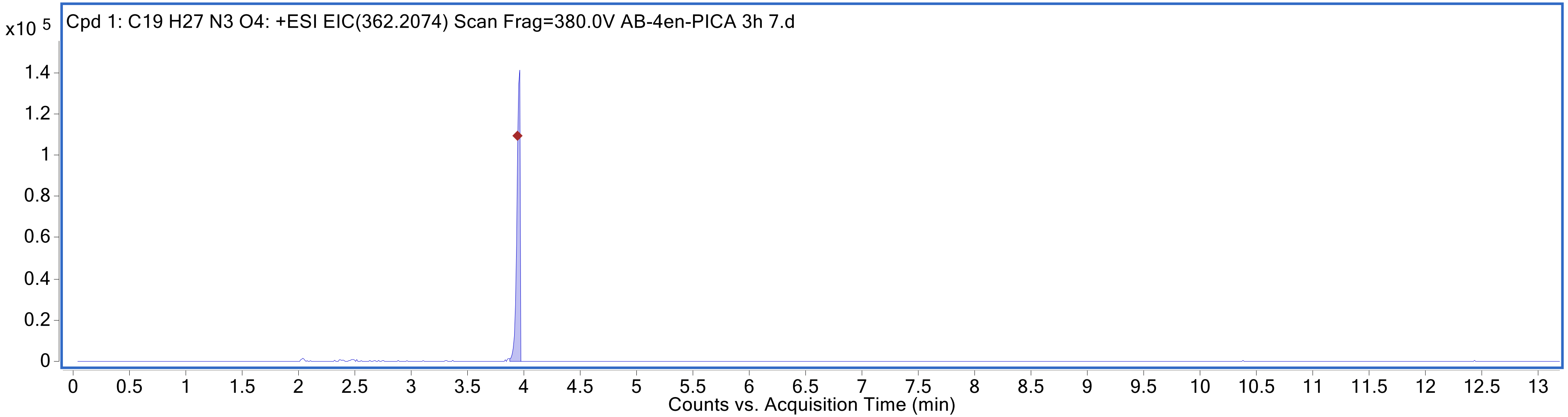

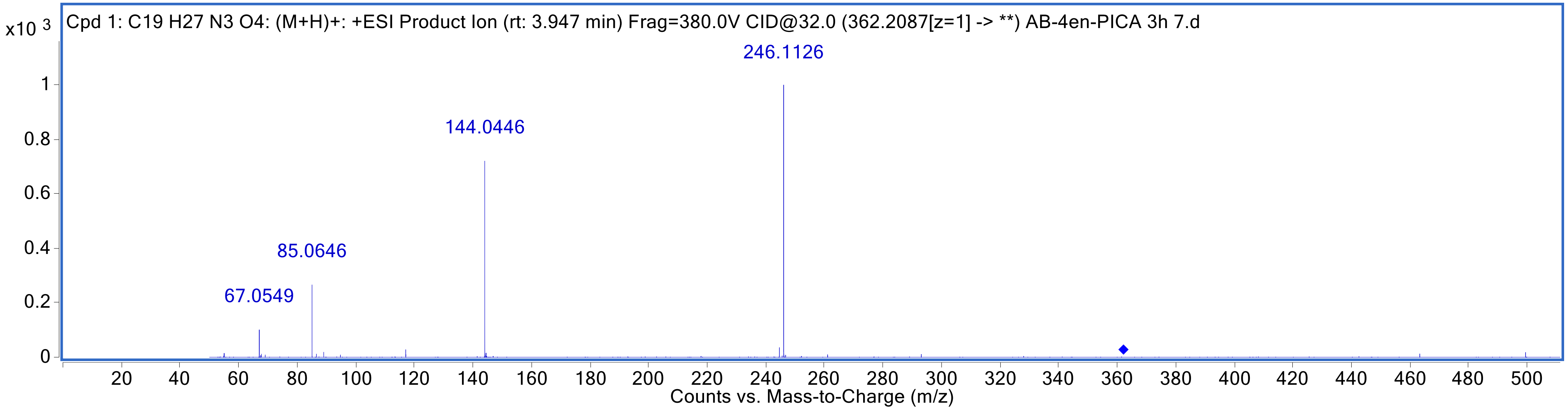

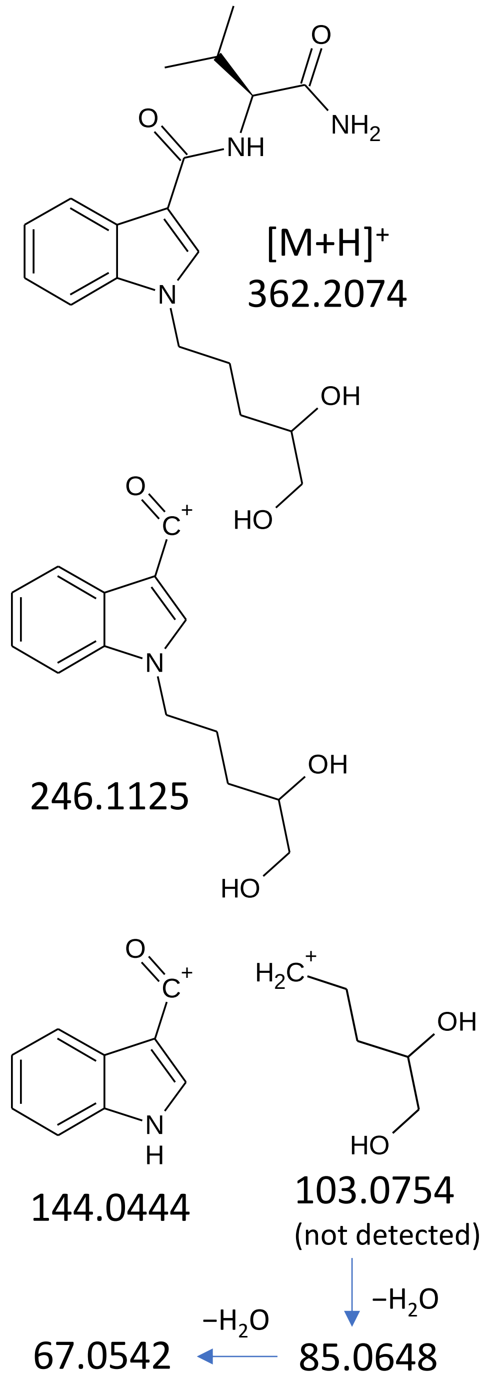

Dihydrodiol reference standard, RT 3.96 min, *m/z* 362.2069

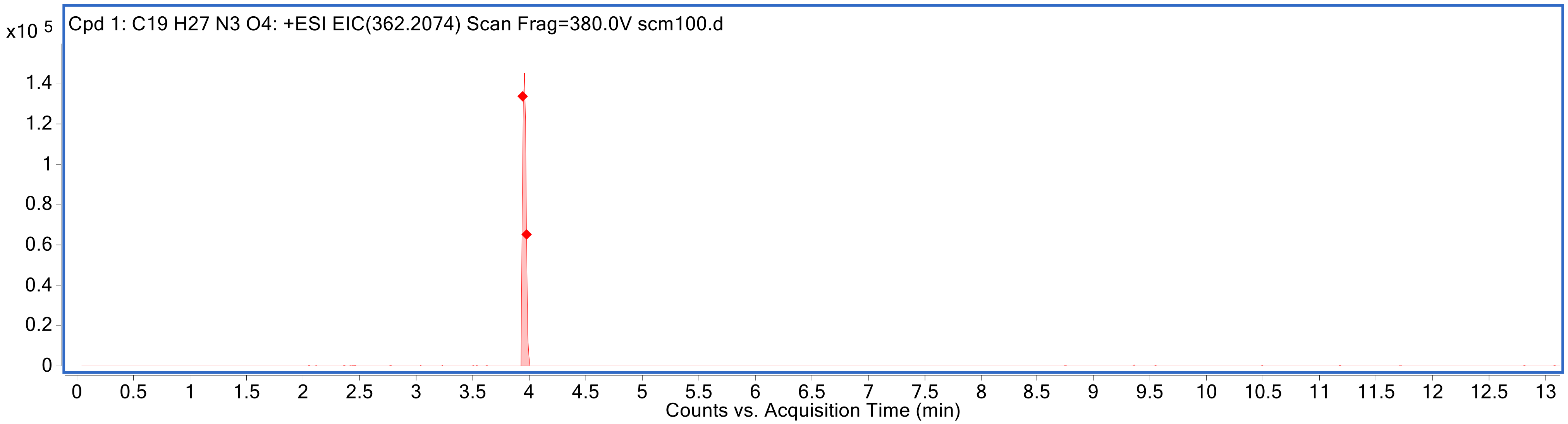

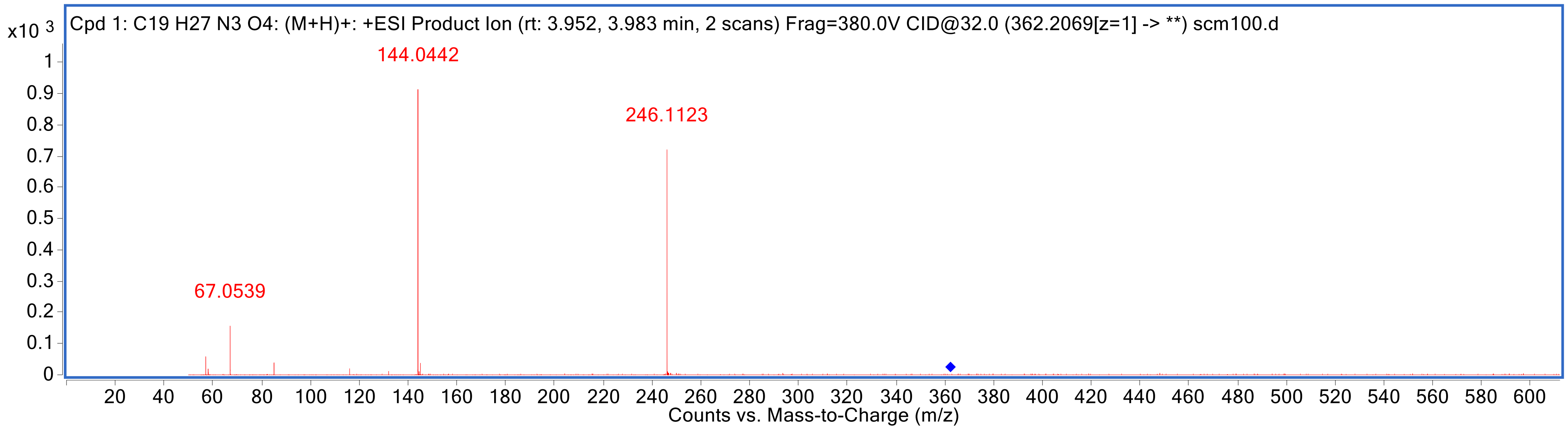

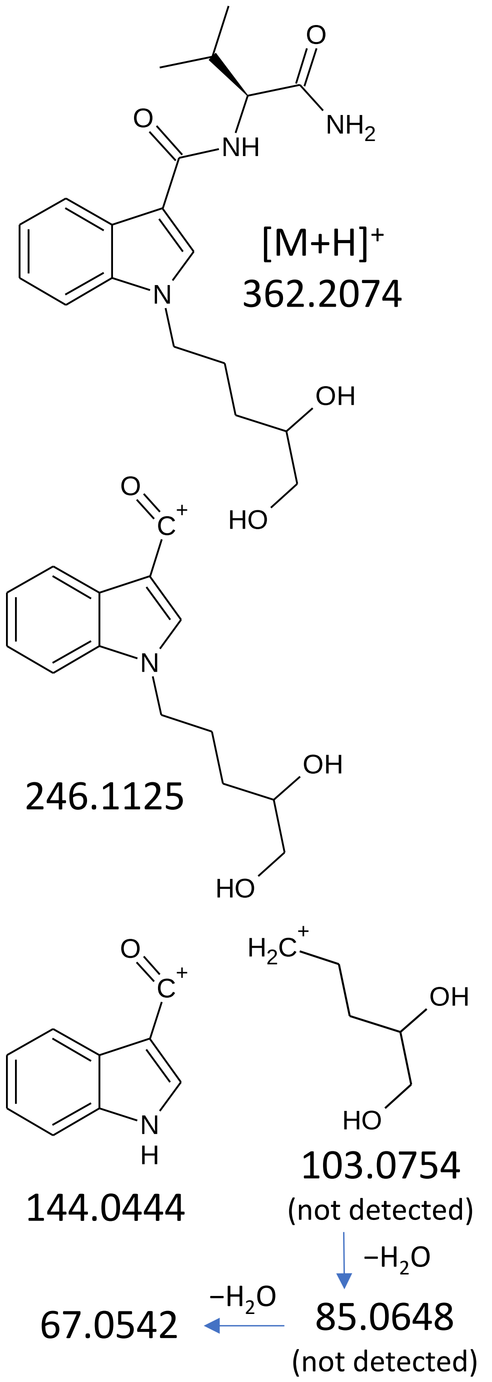

G5, Mono-hydroxylation (*iso*-propyl), RT 6.82 min, *m/z* 344.1970

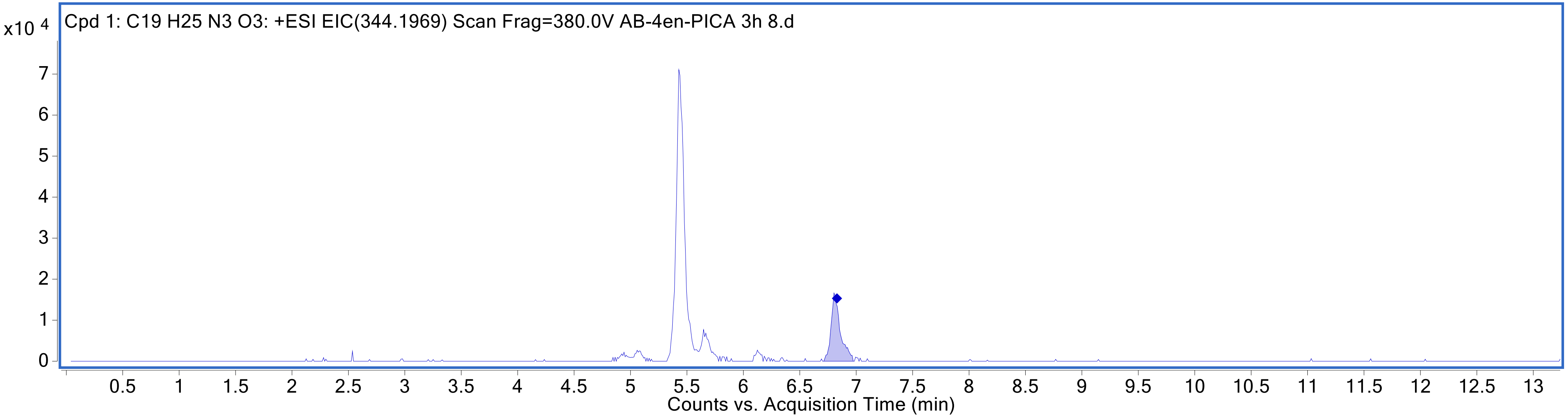

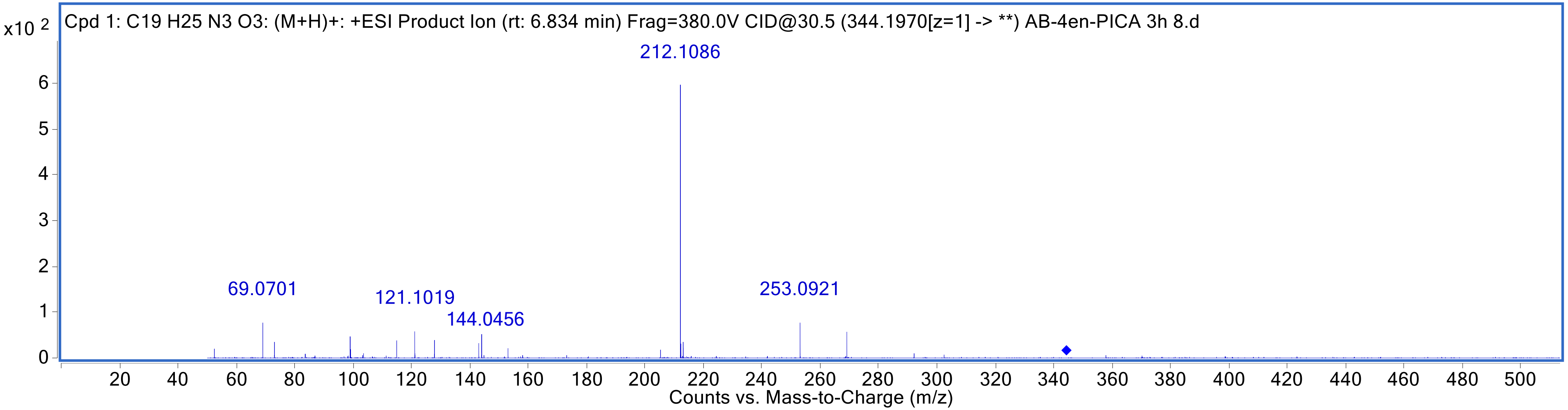

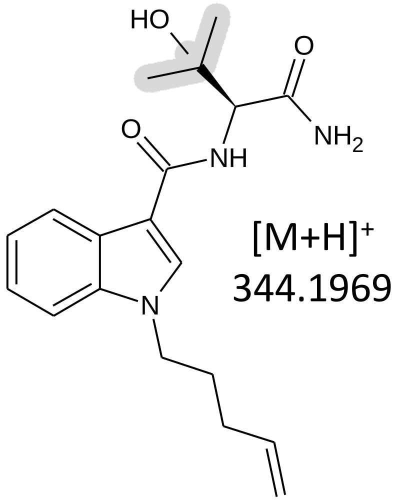

[M+H]+
344.1969

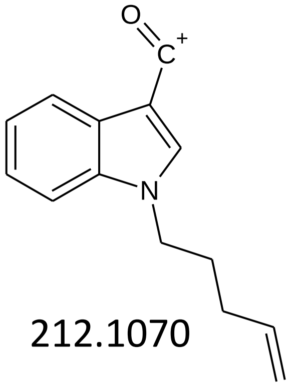

212.1070

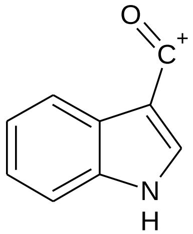

144.0444

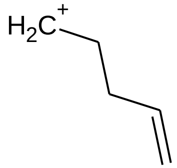

69.0699

AB-4en-PINACA

Metabolism

AB-4en-PINACA, RT 8.61 min, *m/z* 329.2005

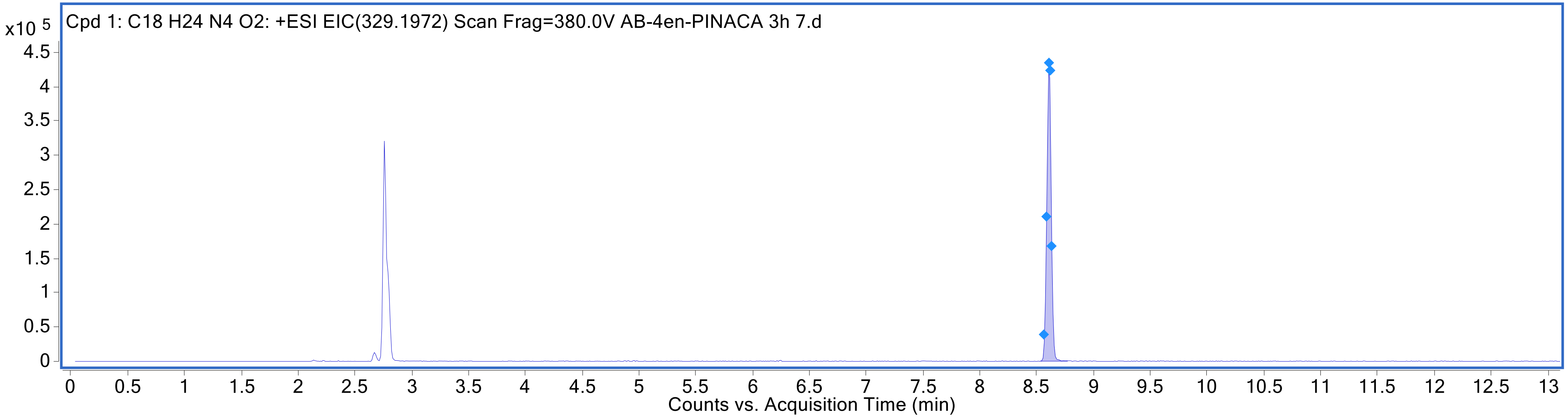

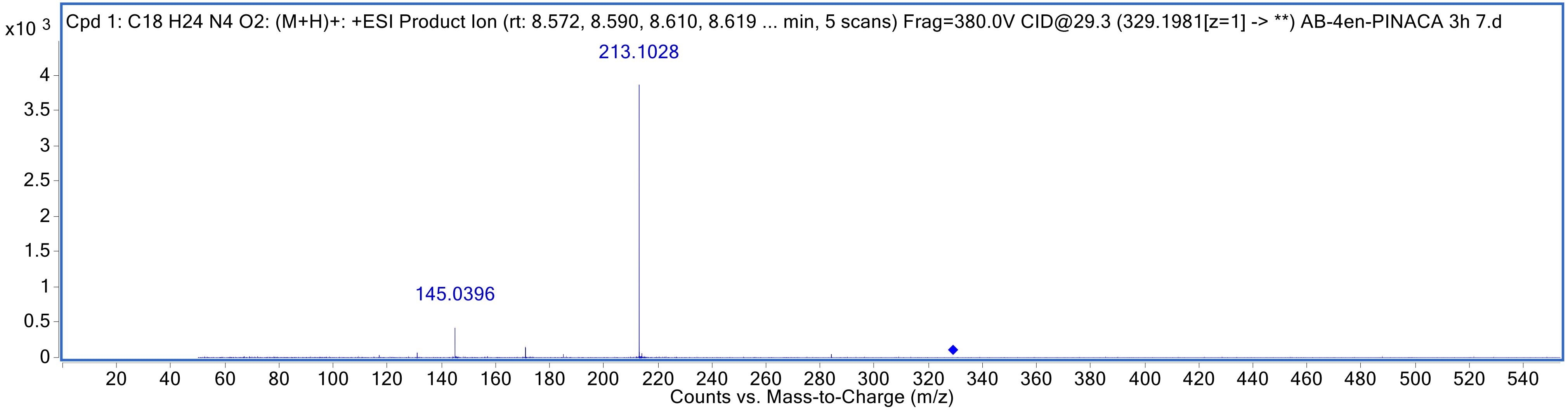

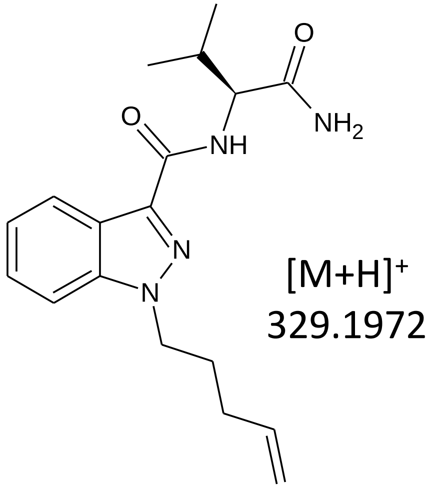

$[M+H]^+$
329.1972

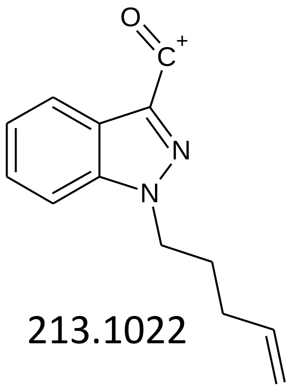

213.1022

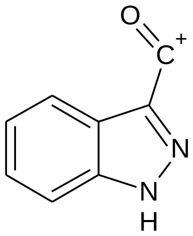

145.0396

H1, Terminal amide hydrolysis, RT 9.77 min, m/z 330.1840

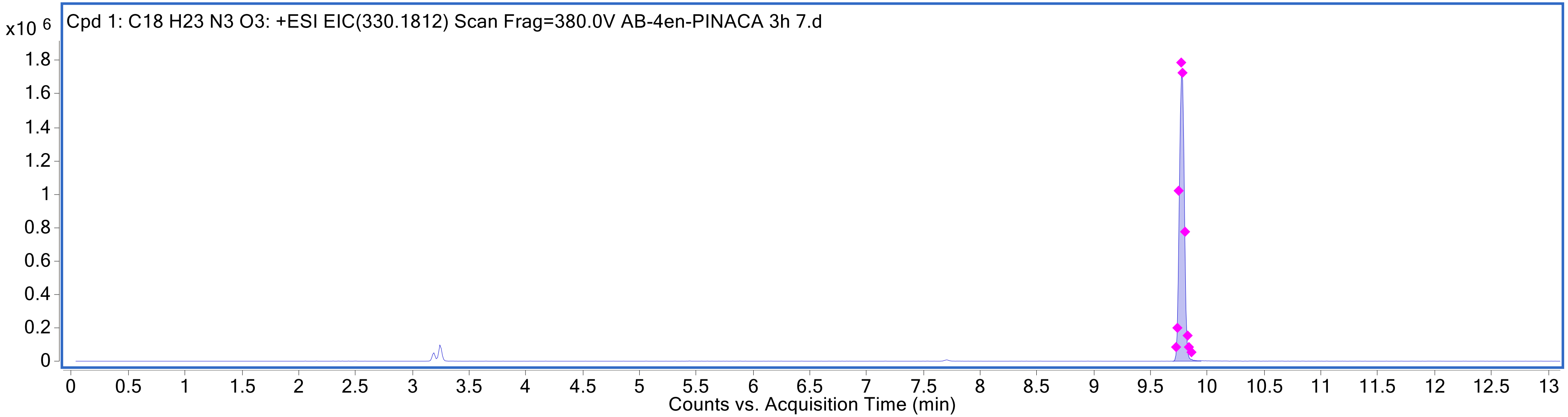

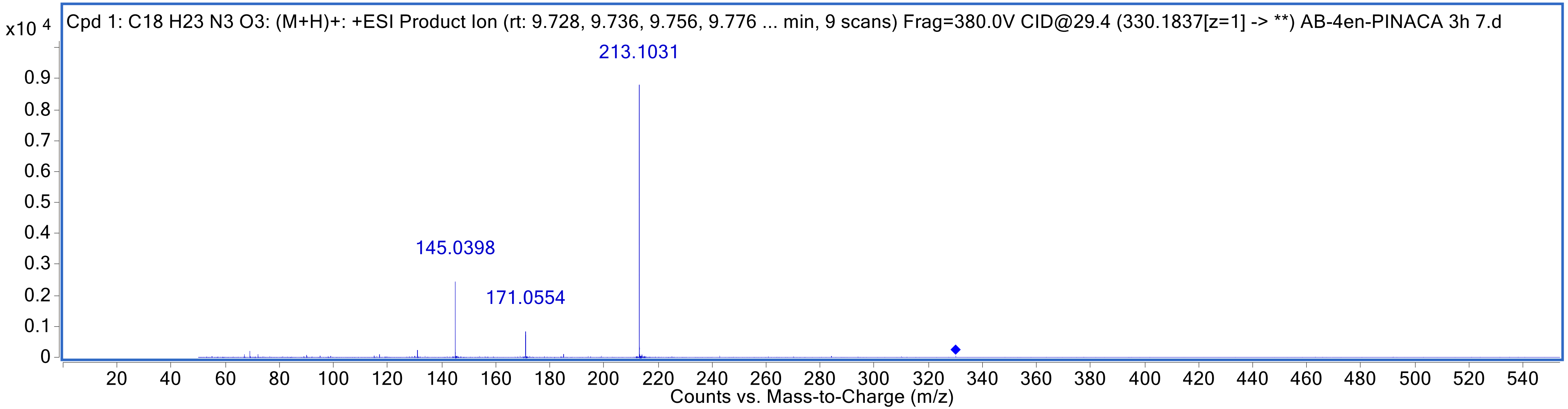

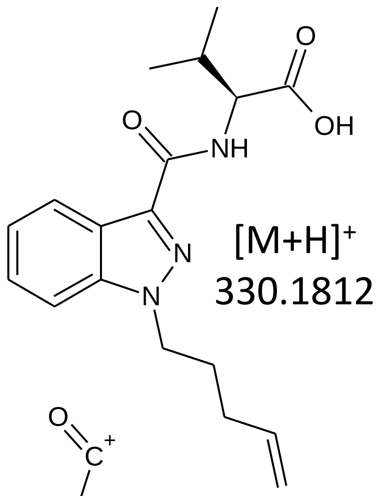

[M+H]+
330.1812

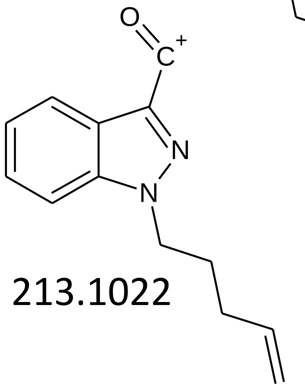

213.1022

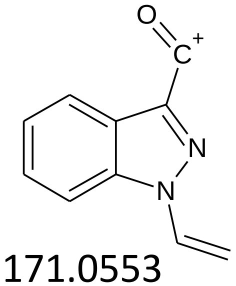

171.0553

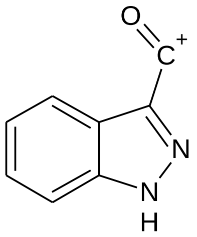

145.0396

H2, Terminal amide hydrolysis + dihydrodiol formation, RT 4.88 min, *m/z* 364.1877

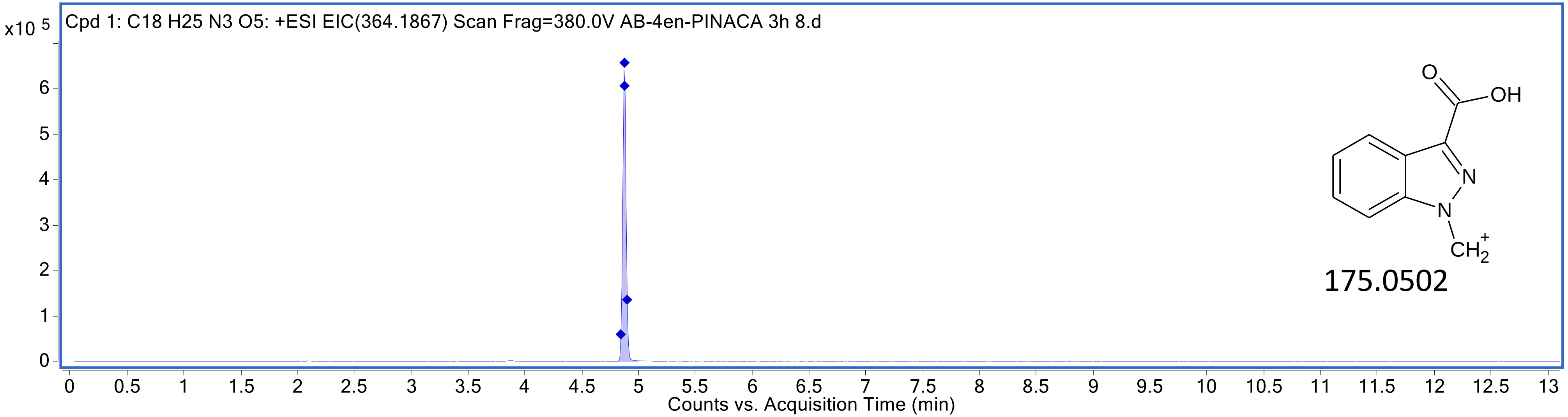

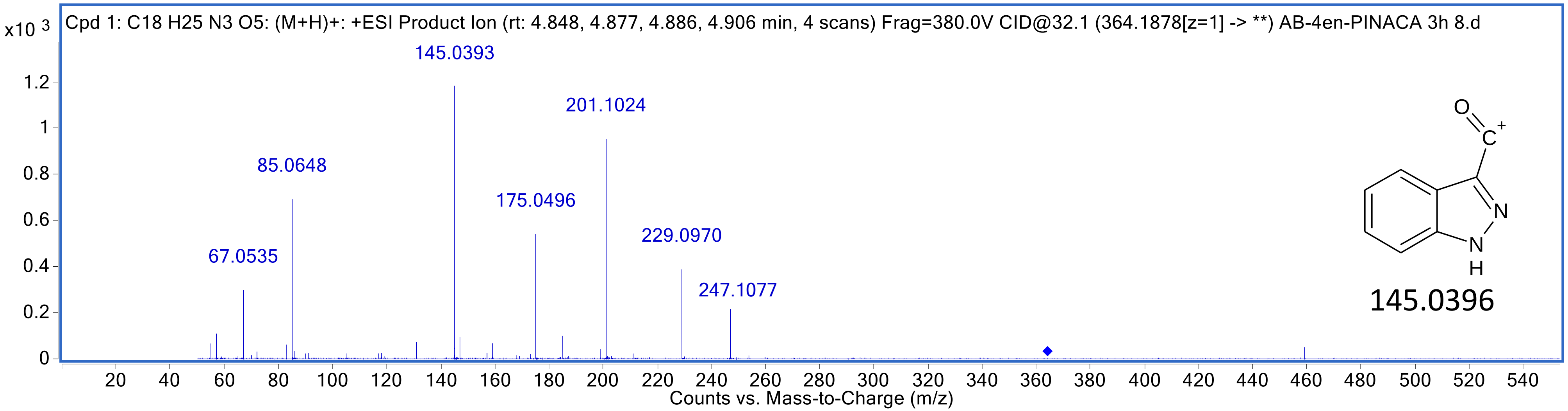

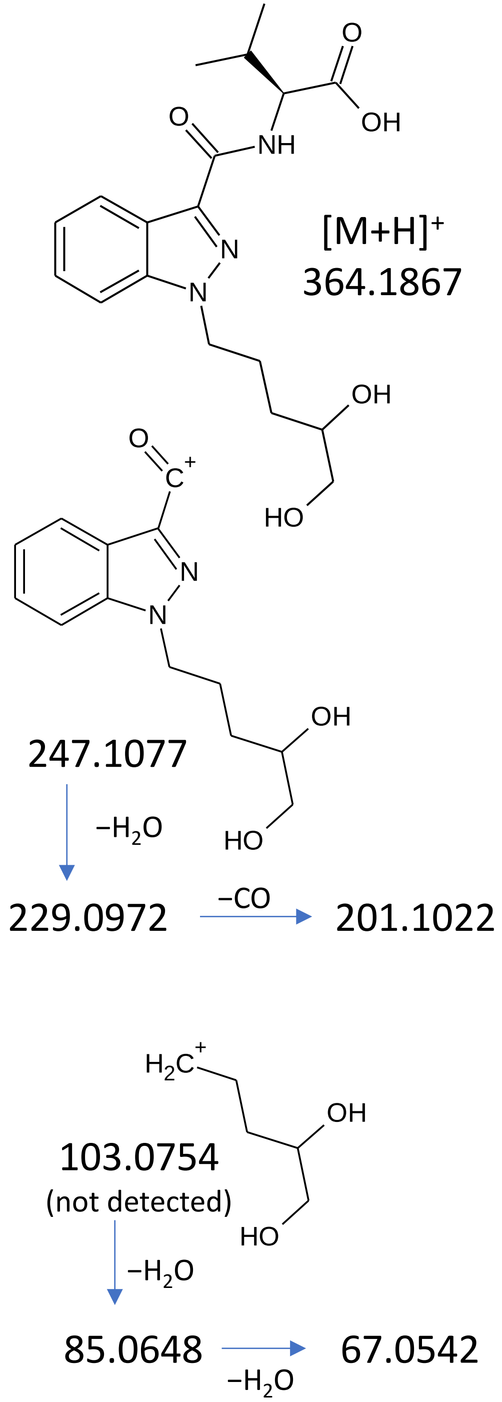

H3, Dihydrodiol formation, RT 4.04 min, *m/z* 363.2032

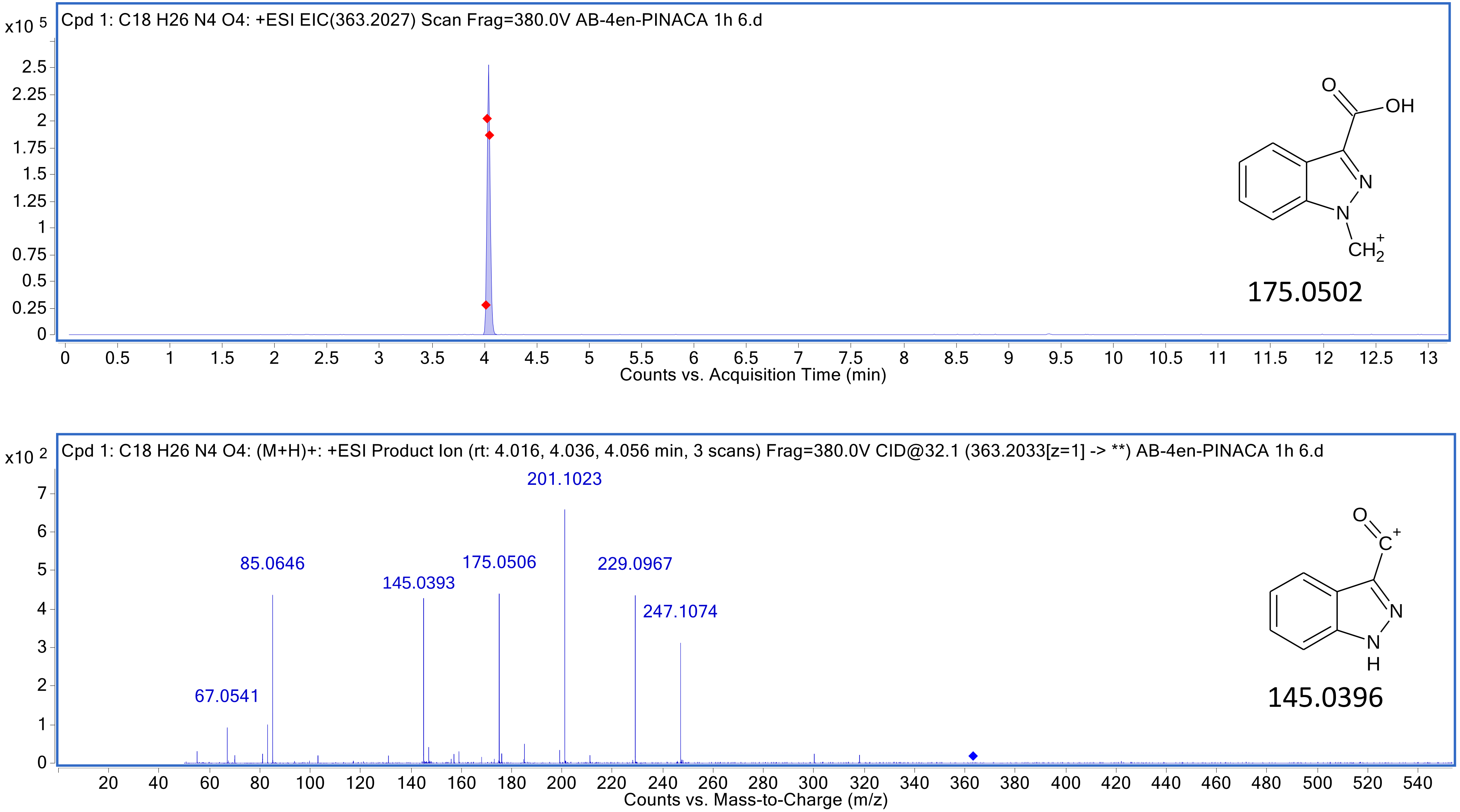

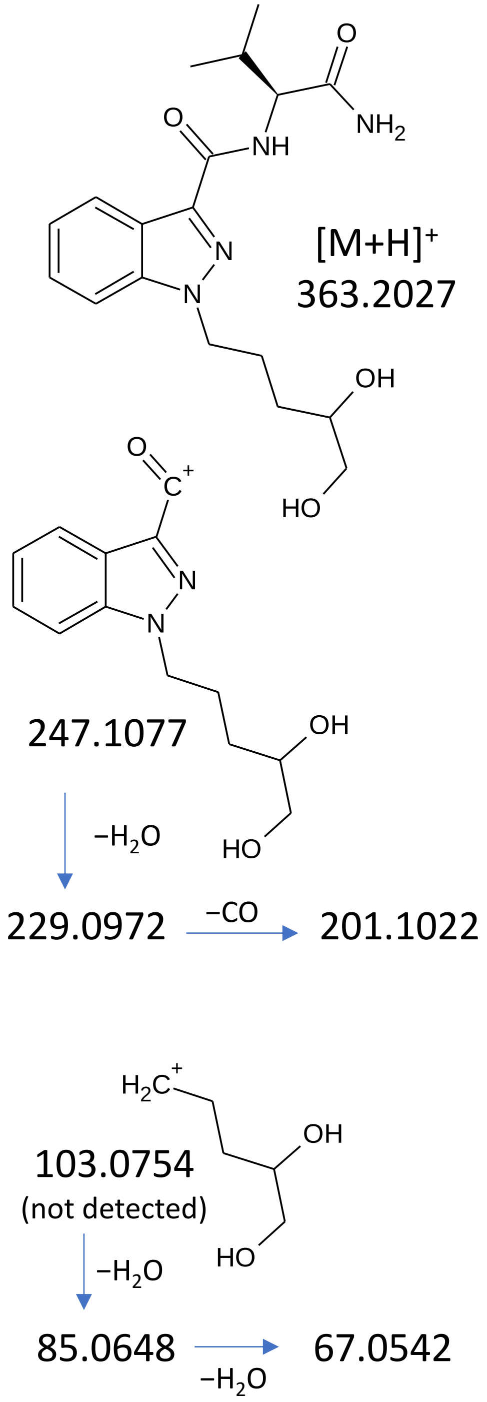

Dihydrodiol reference standard, RT 4.05 min, *m/z* 363.2029

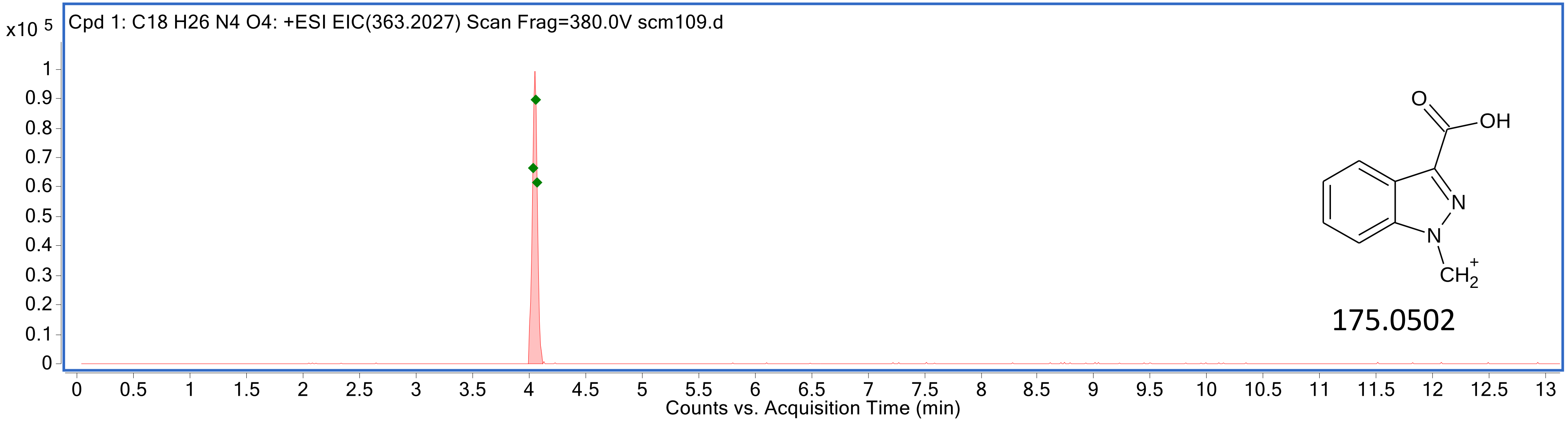

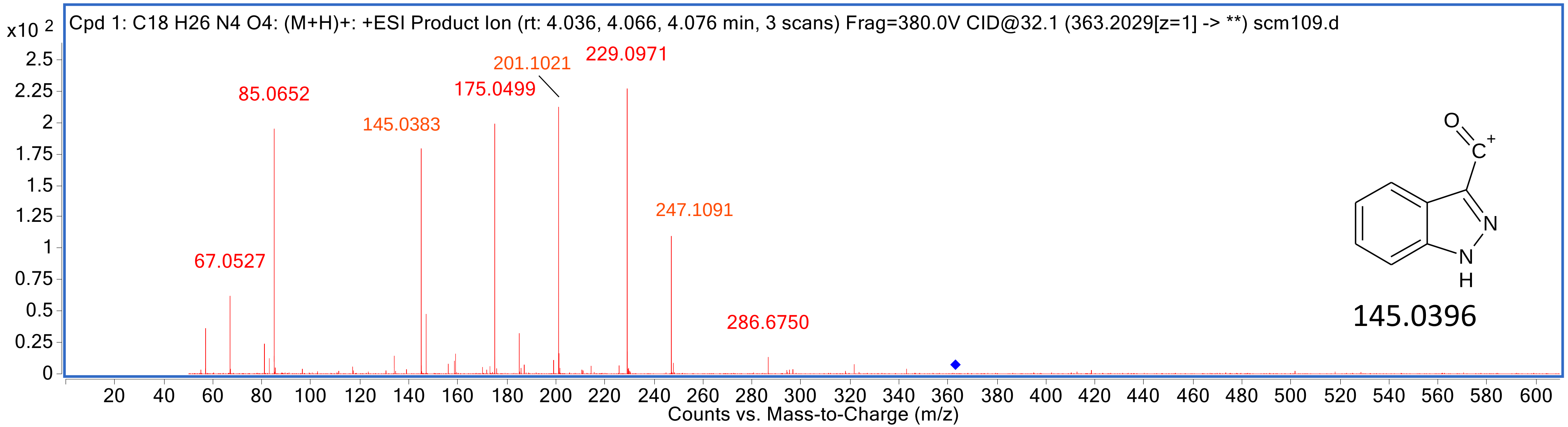

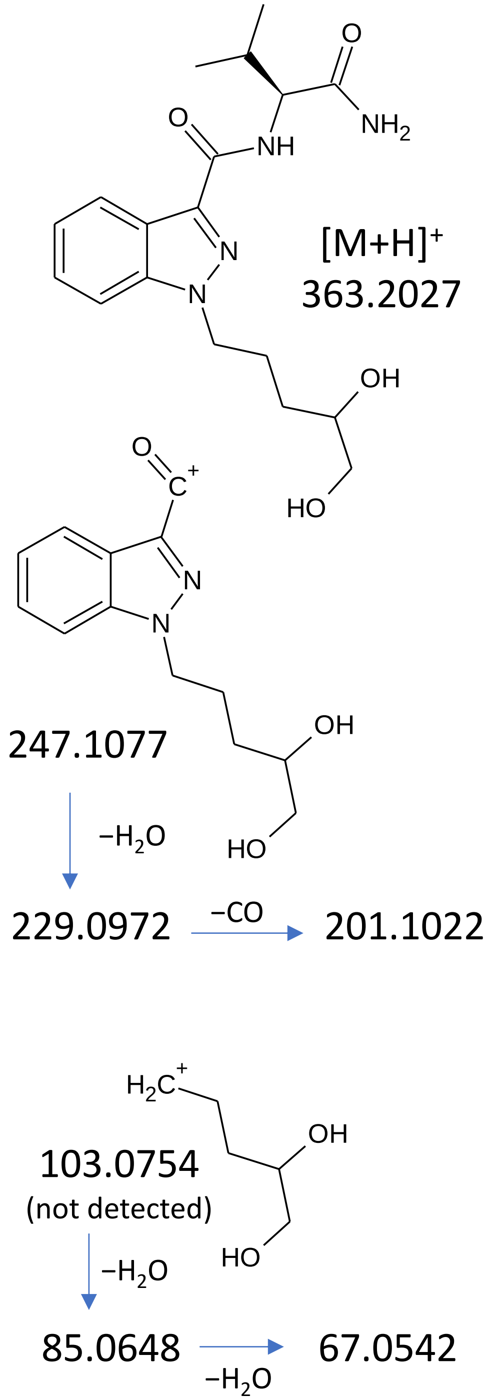

H4, Mono-hydroxylation (pentenyl tail), RT 5.55 min, *m/z* 345.1923

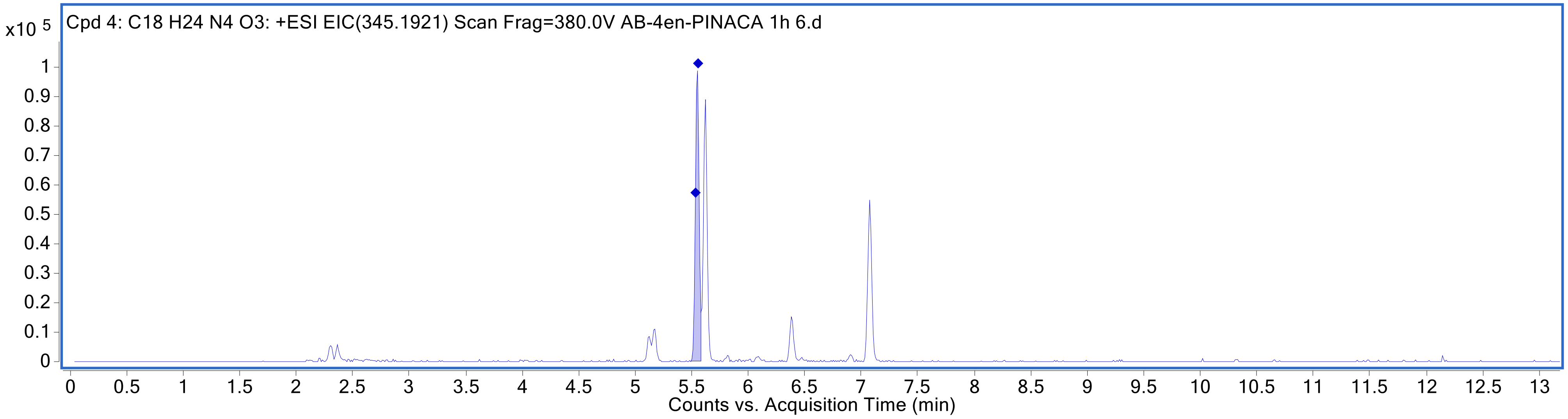

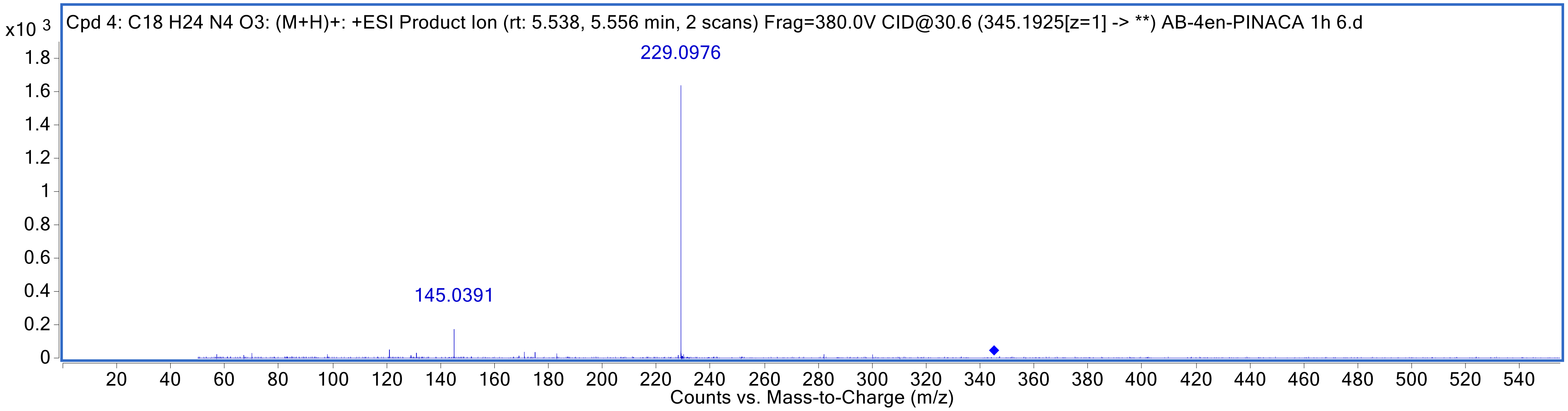

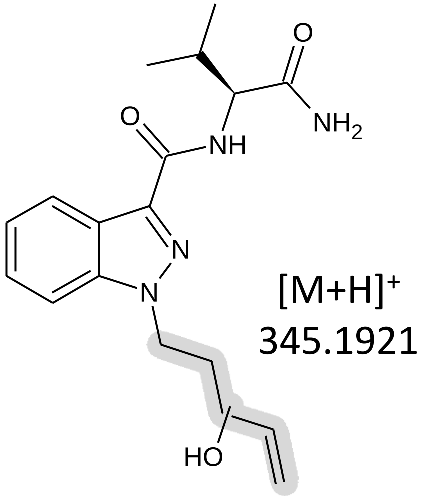

$[M+H]^+$
345.1921

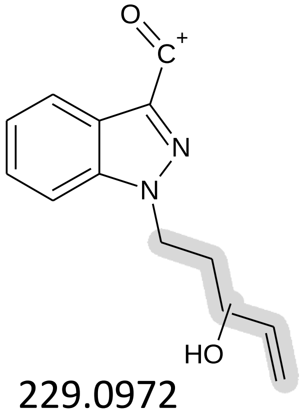

229.0972

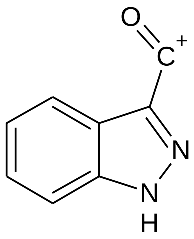

145.0396

H5, Mono-hydroxylation (pentenyl tail), RT 5.62 min, *m/z* 345.1920

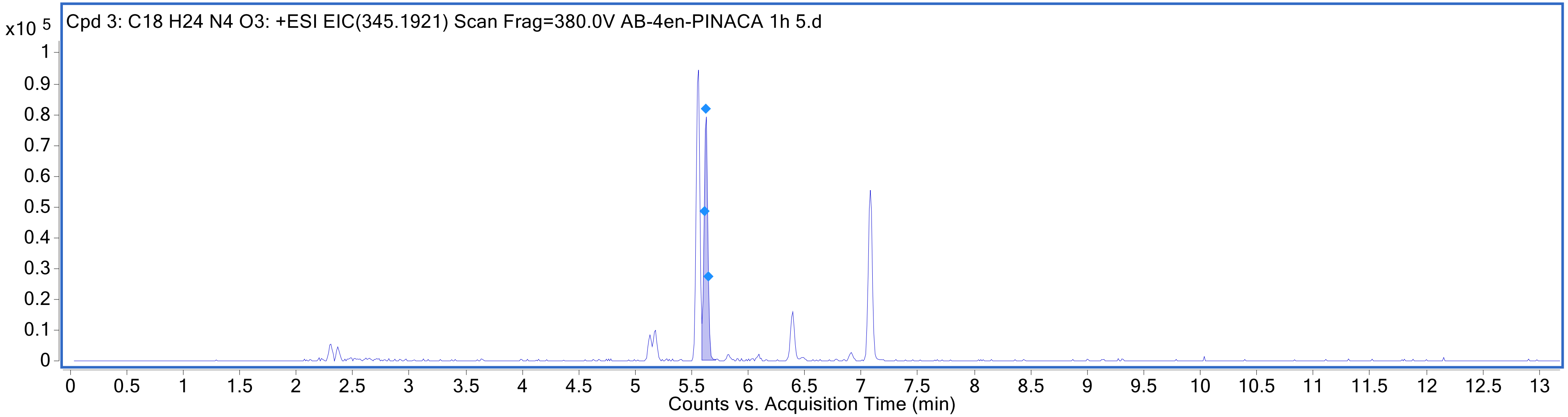

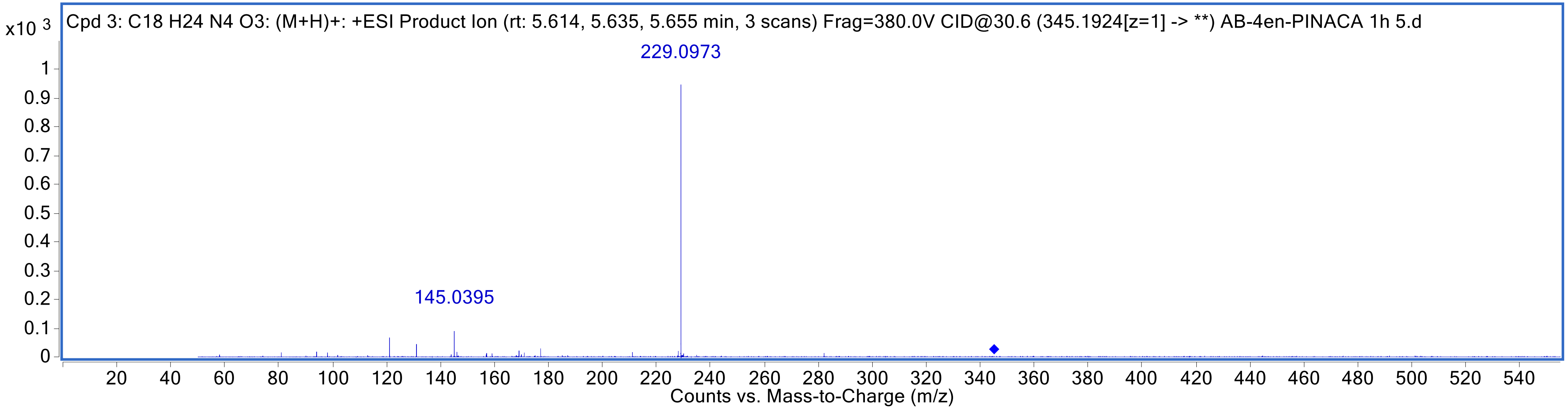

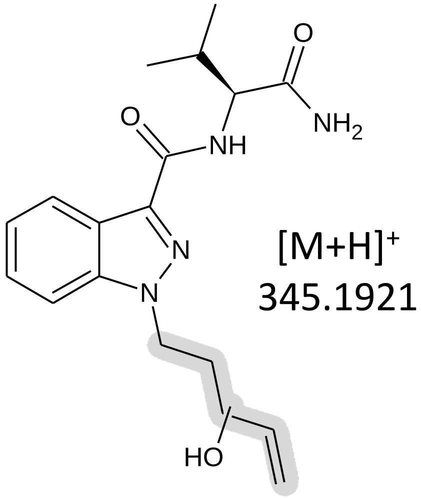

$[M+H]^+$
345.1921

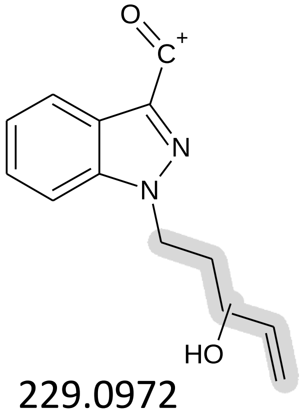

229.0972

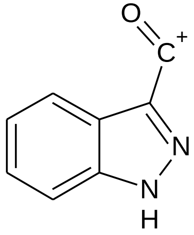

145.0396

H6, Mono-hydroxylation (*iso*-propyl), RT 7.08 min, *m/z* 345.1924

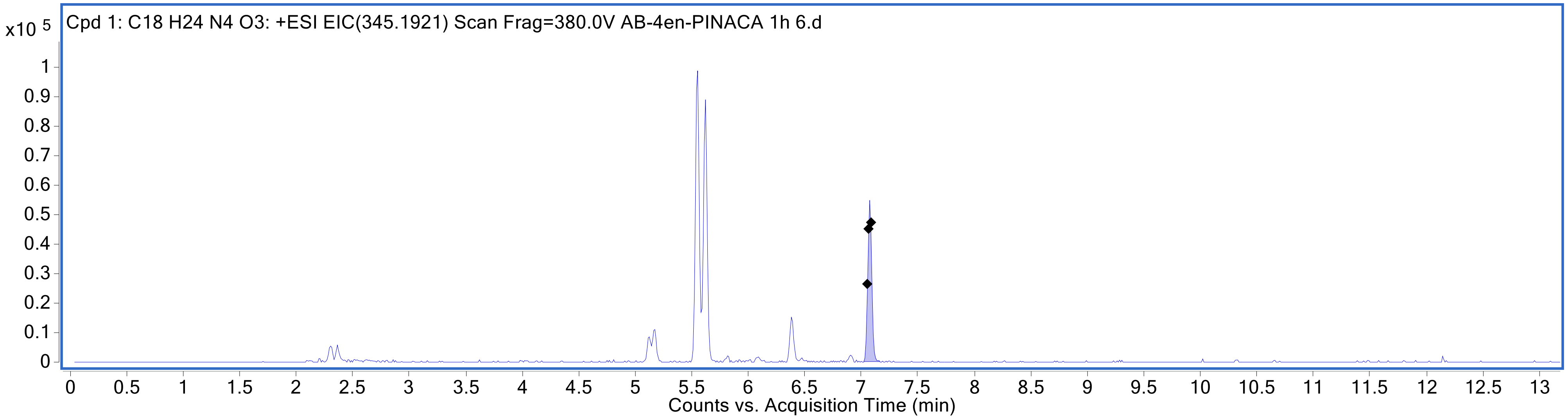

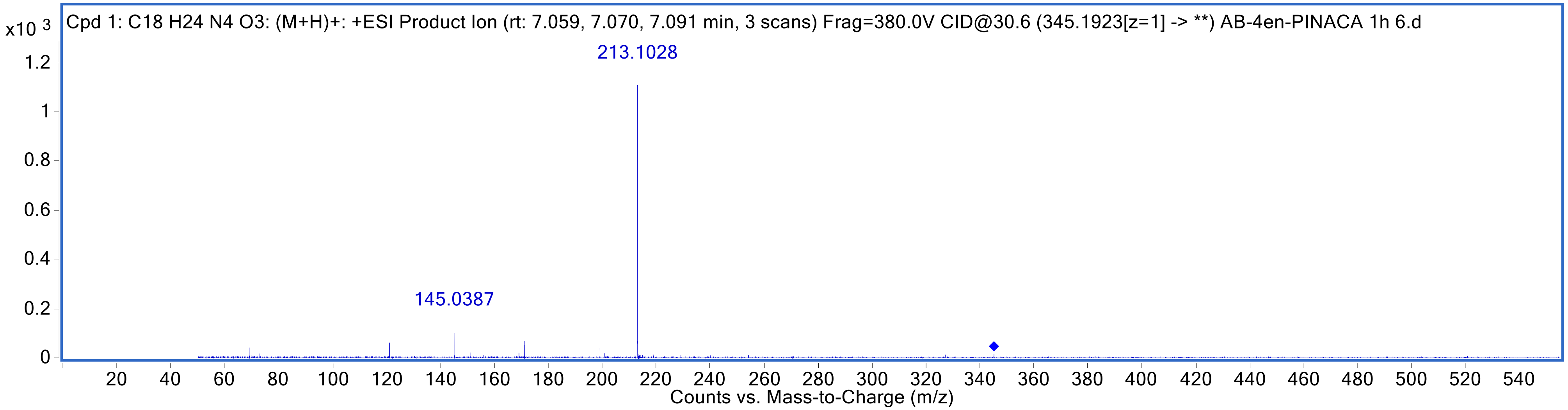

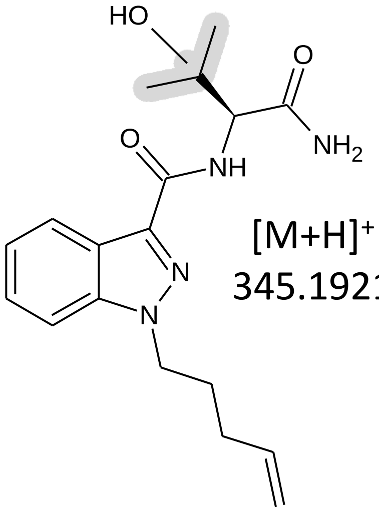

$[M+H]^+$
345.1921

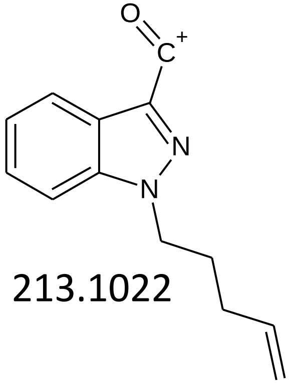

213.1022

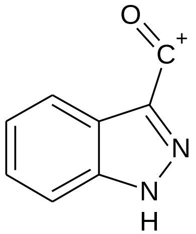

145.0396

H7, Terminal amide hydrolysis + glucuronidation, RT 7.70 min, *m/z* 506.2126

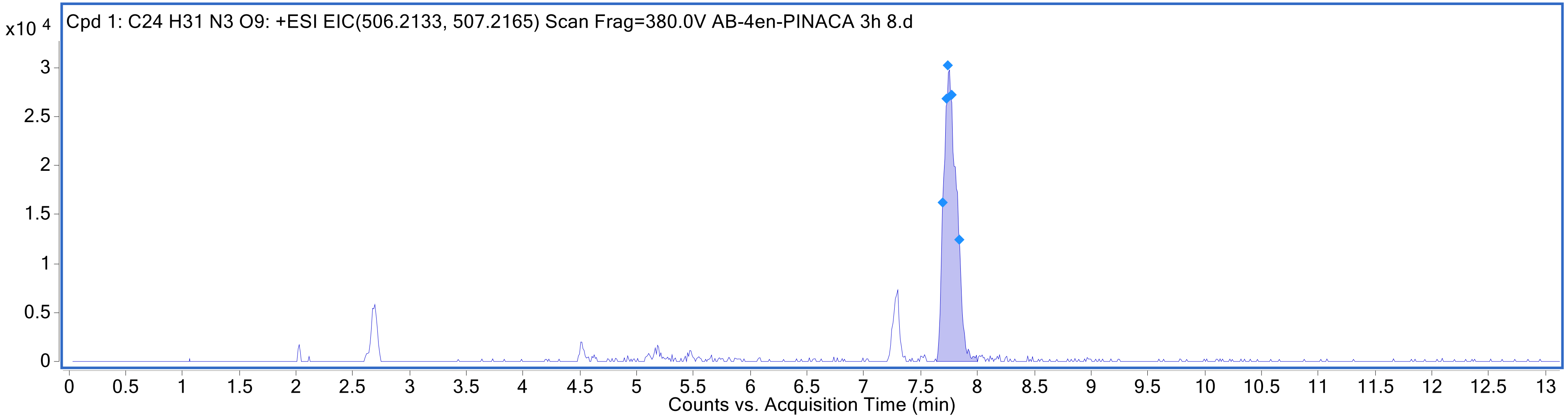

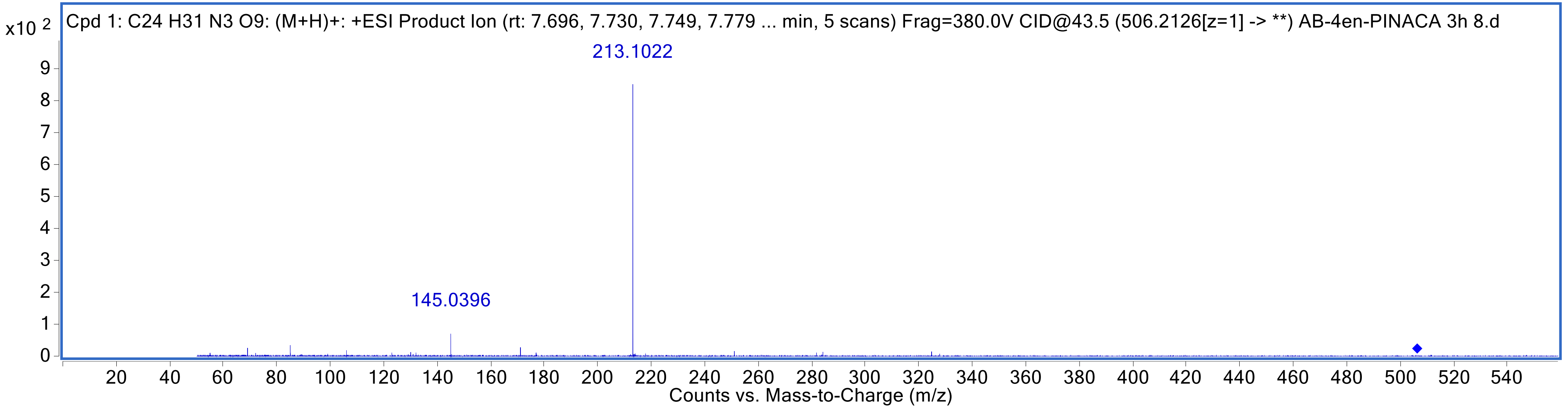

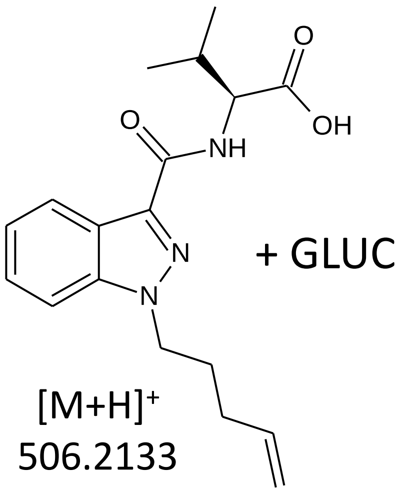

[M+H]+
506.2133

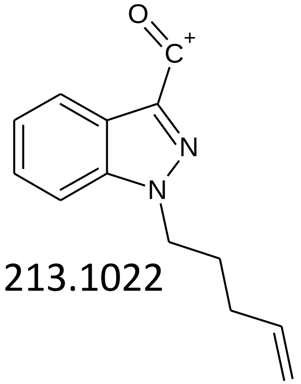

213.1022

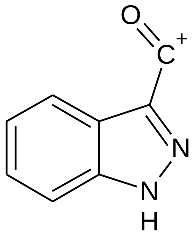

145.0396

H8, Terminal amide hydrolysis + mono-hydroxylation (pentenyl tail), RT 6.58 min, *m/z* 346.1762

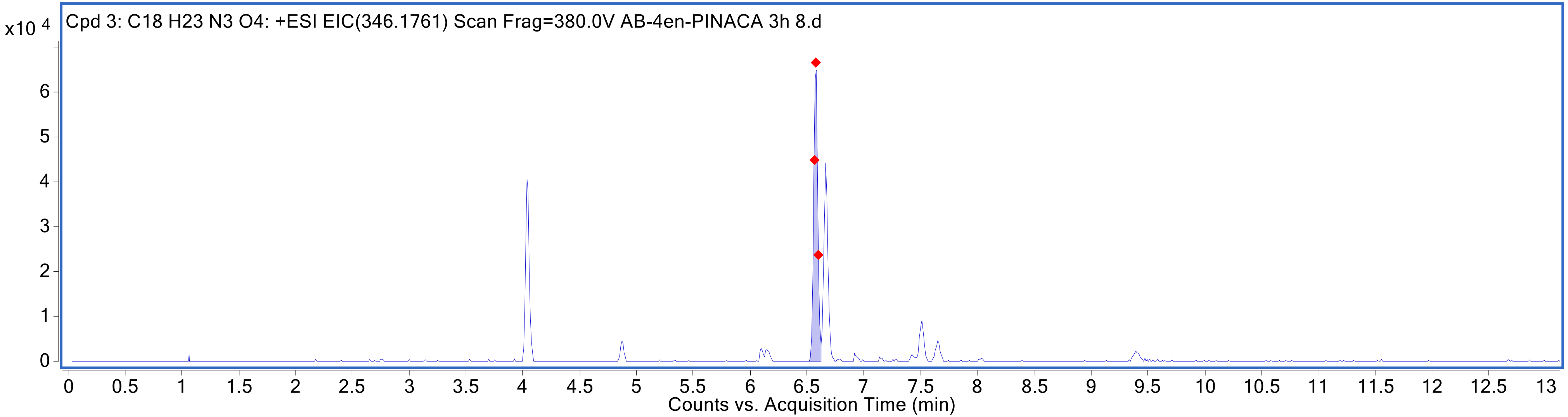

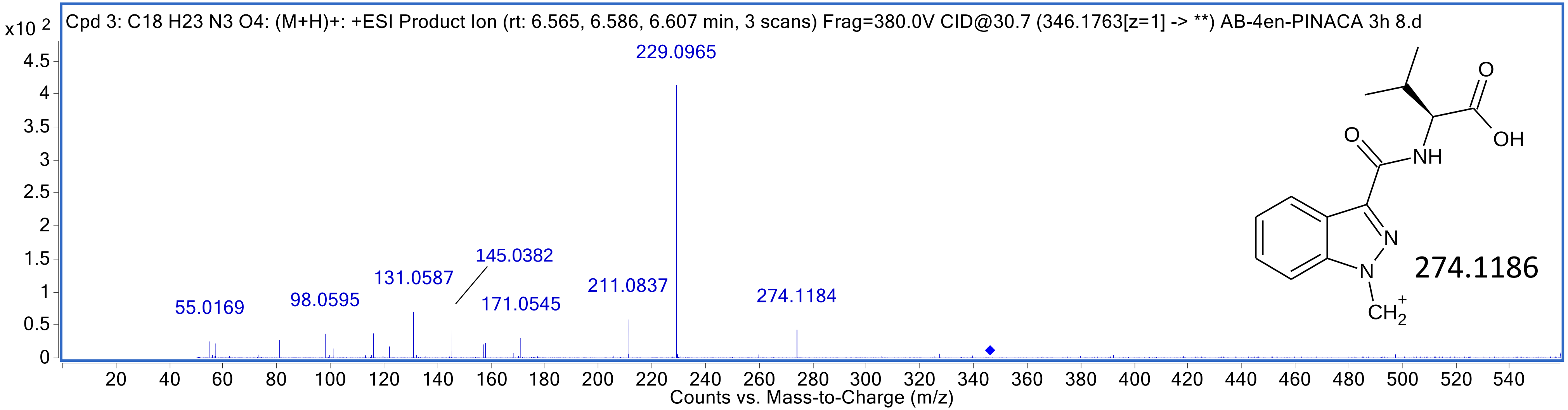

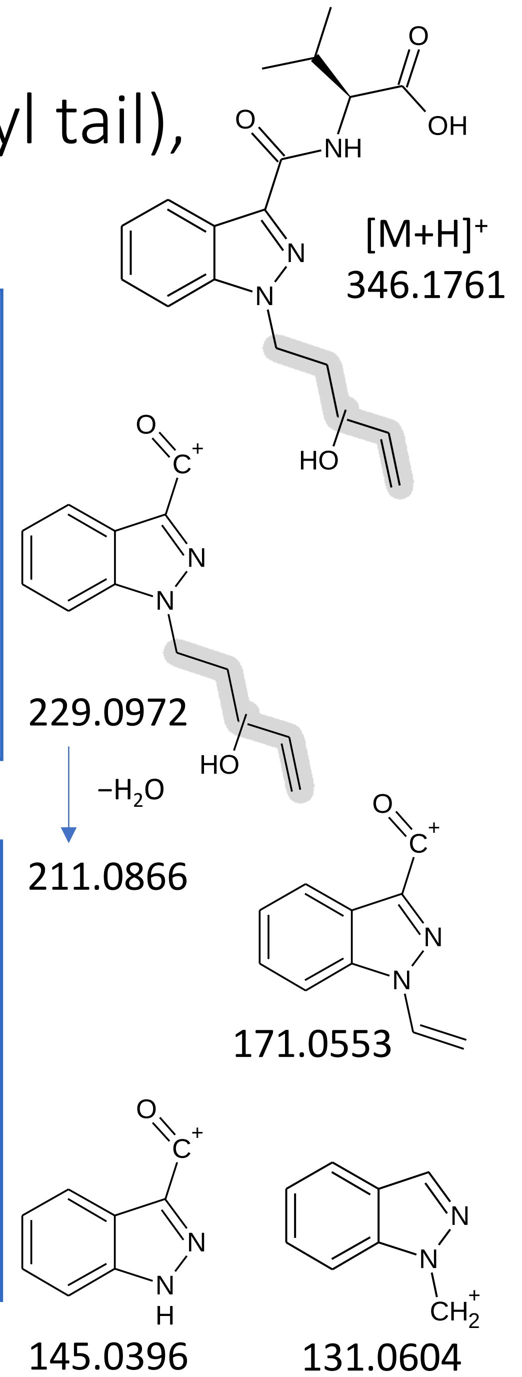

H9, Terminal amide hydrolysis + di-hydroxylation (pentenyl tail), RT 5.42 min, *m/z* 362.1704

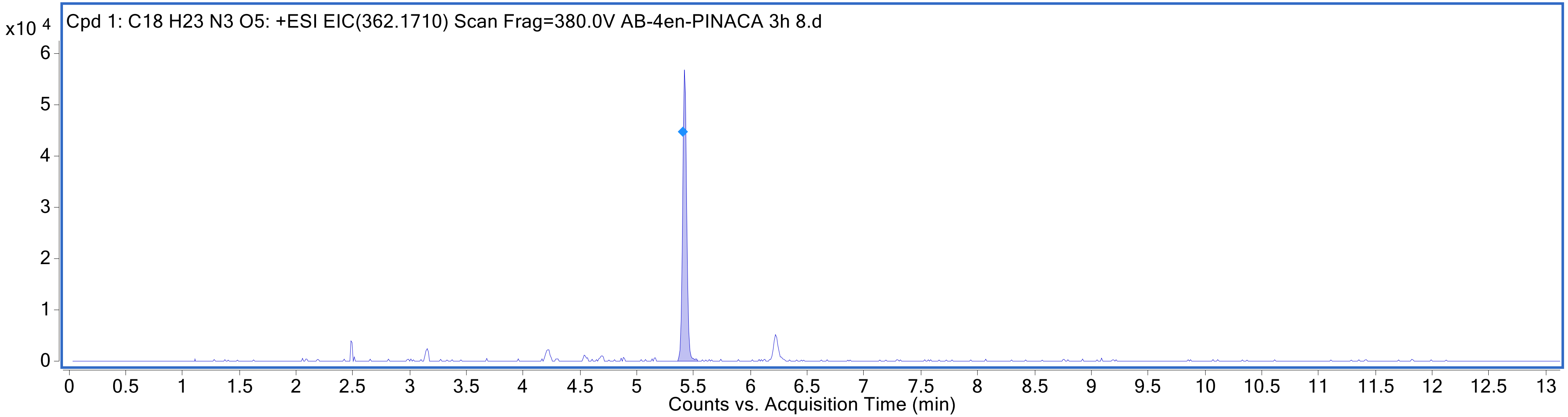

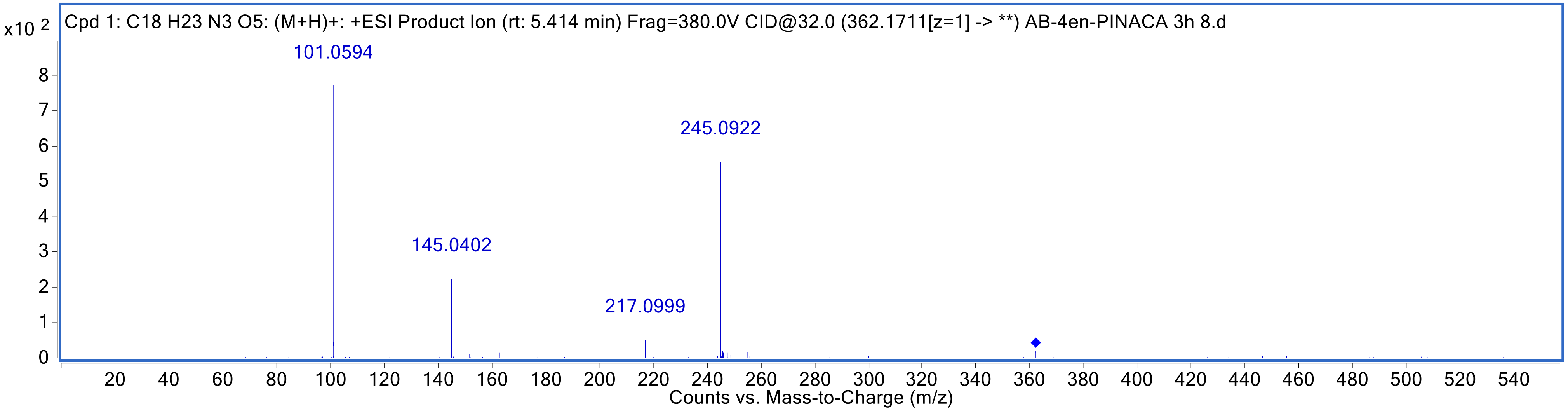

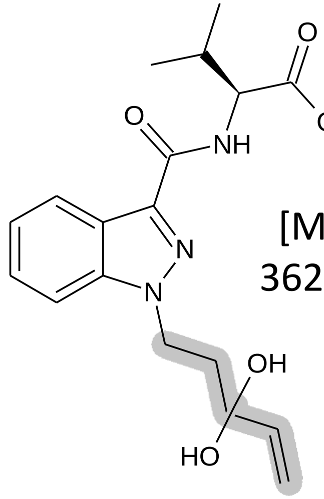

$[M+H]^+$
362.1710

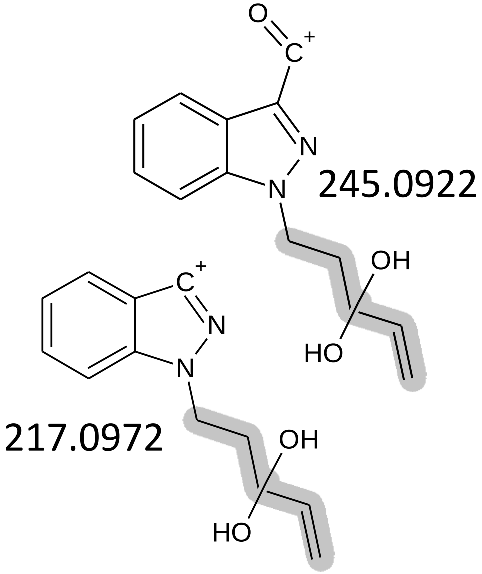

245.0922

217.0972

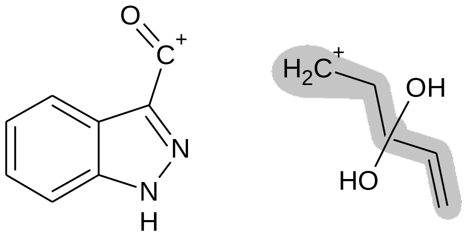

145.0396

101.0597

H10, Terminal amide hydrolysis + mono-hydroxylation (pentenyl tail), RT 6.67 min, *m/z* 346.1764

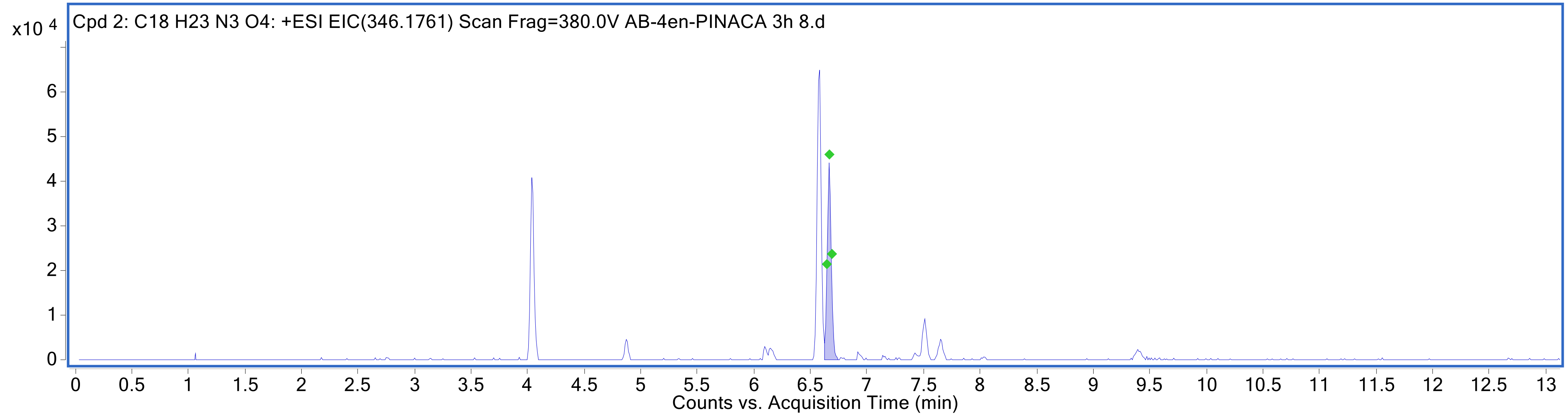

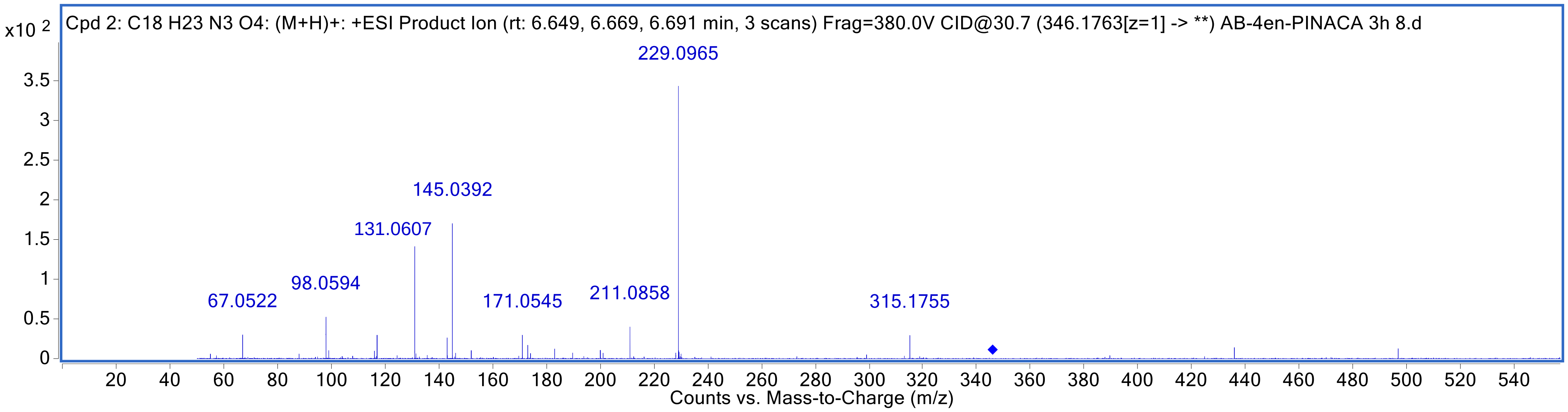

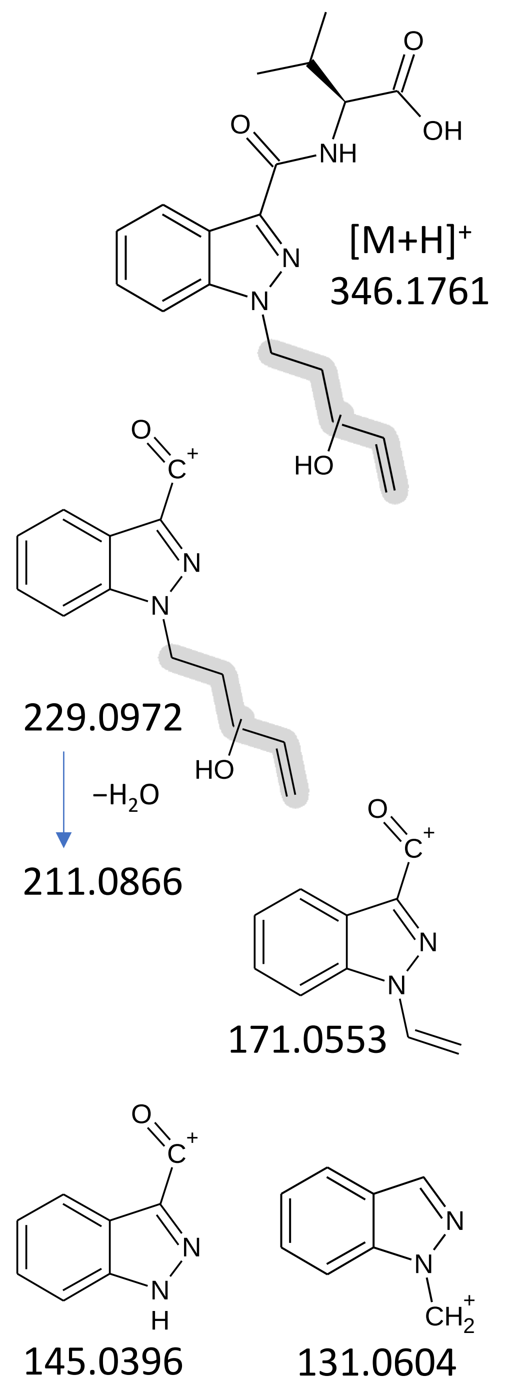

H11, Terminal amide hydrolysis + dihydrodiol formation + glucuronidation, RT 3.87 min, *m/z* 540.2180

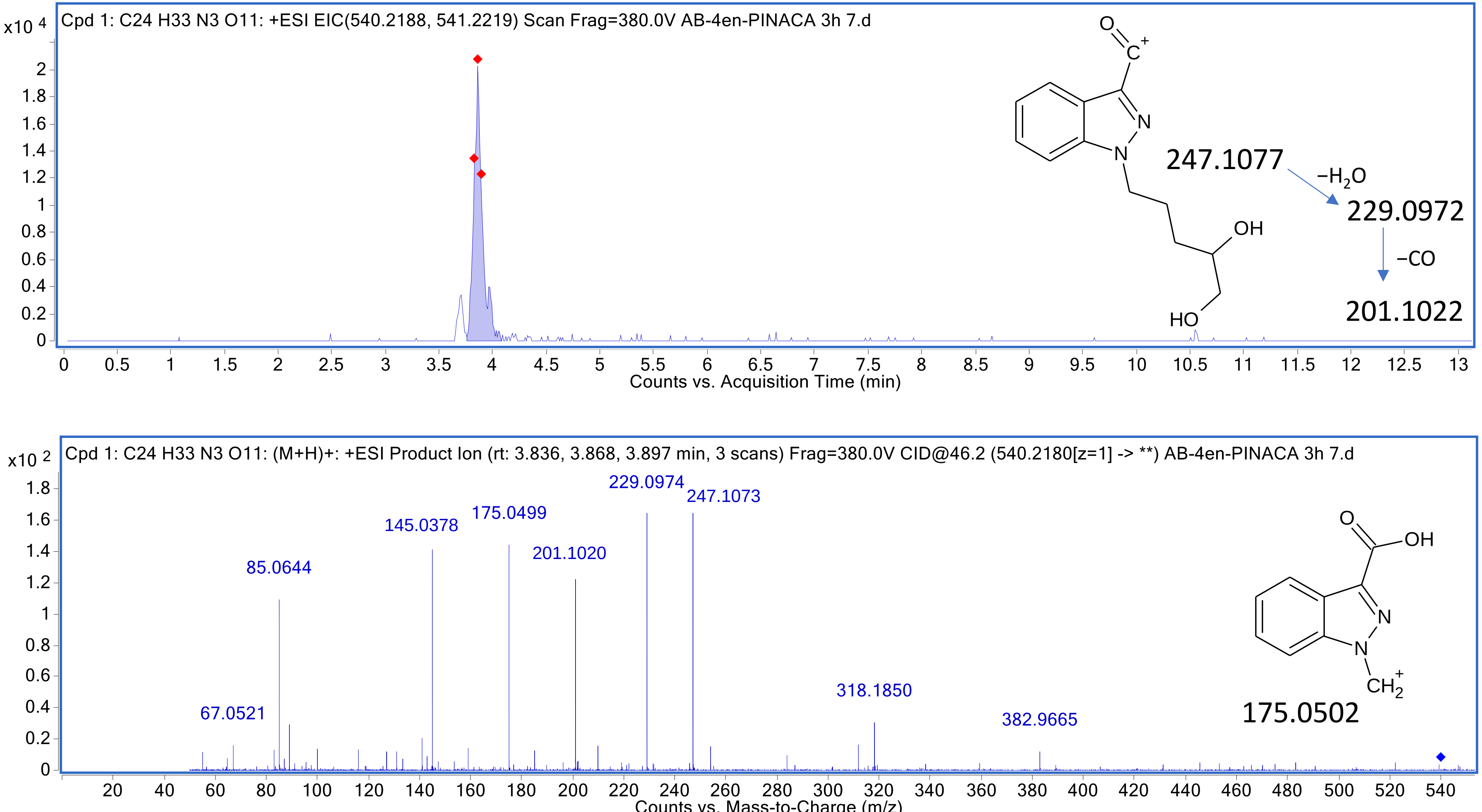

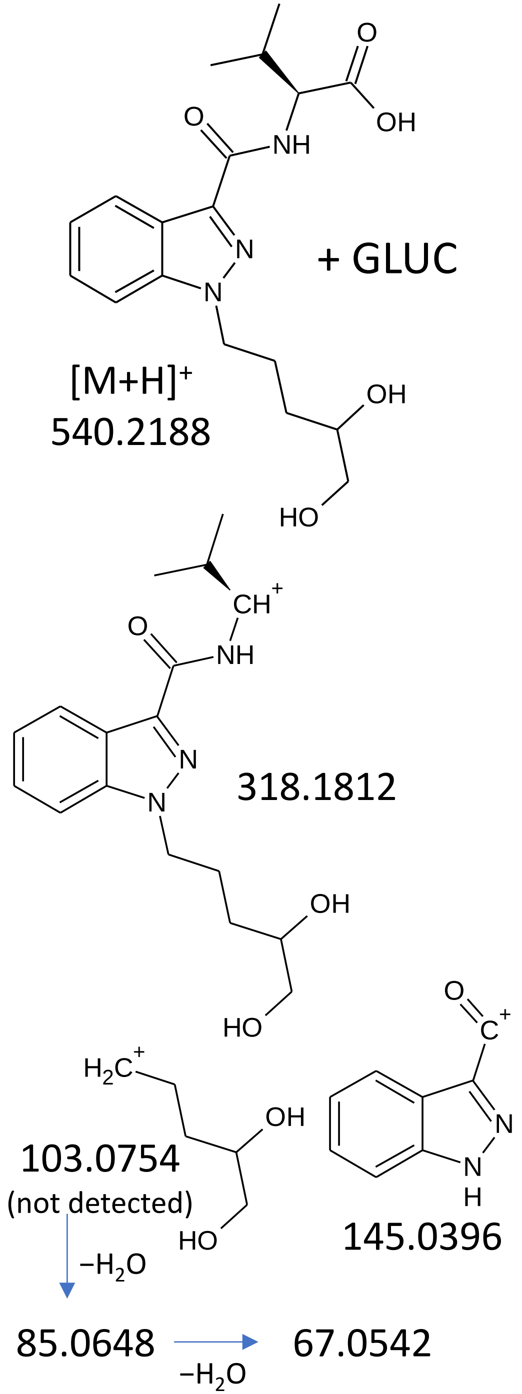

H12, Mono-hydroxylation (pentenyl tail), RT 6.39 min, *m/z* 345.1917

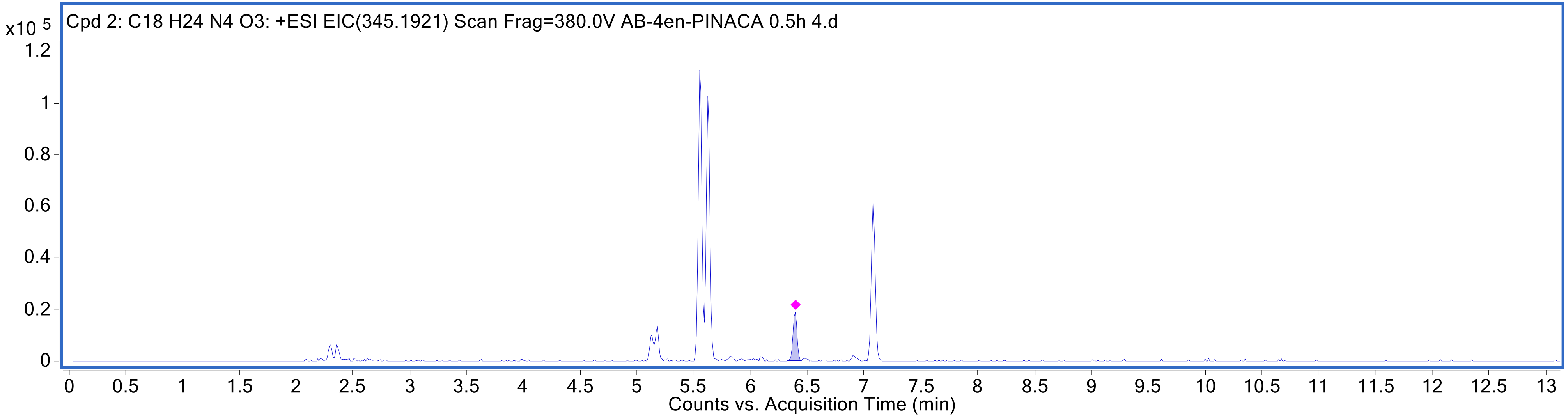

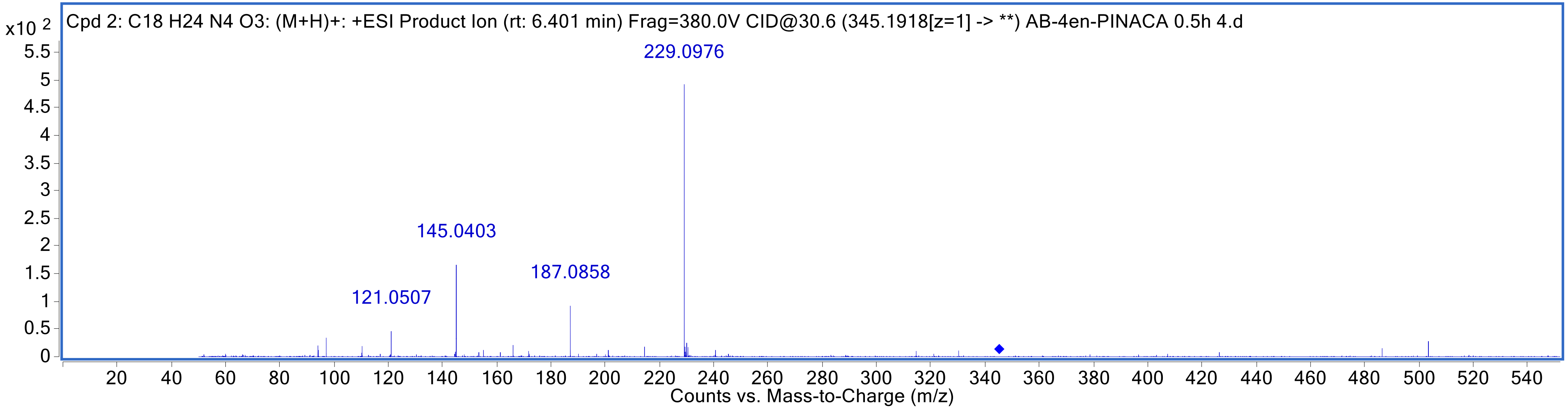

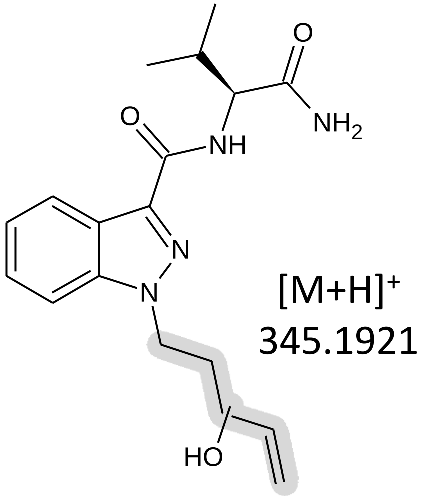

$[M+H]^+$

345.1921

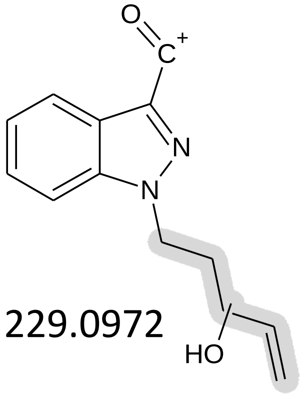

229.0972

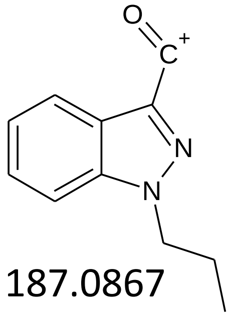

187.0867

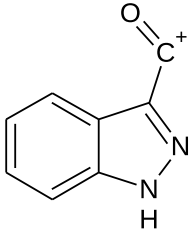

145.0396

H13, Dihydrodiol + mono-hydroxylation (*iso*-propyl), RT 3.16 min, *m/z* 379.1970

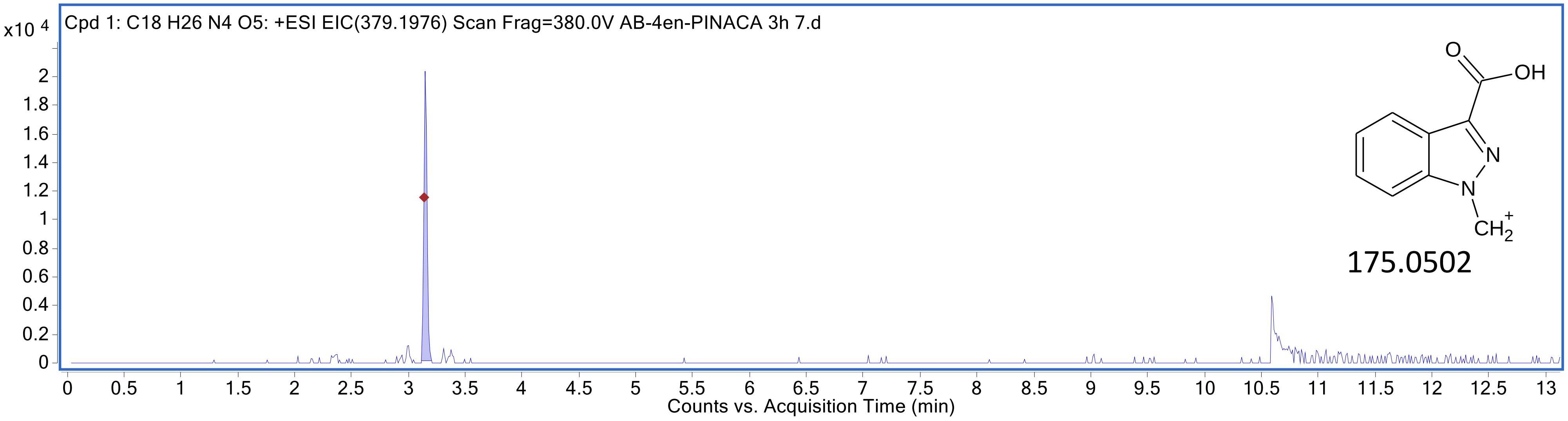

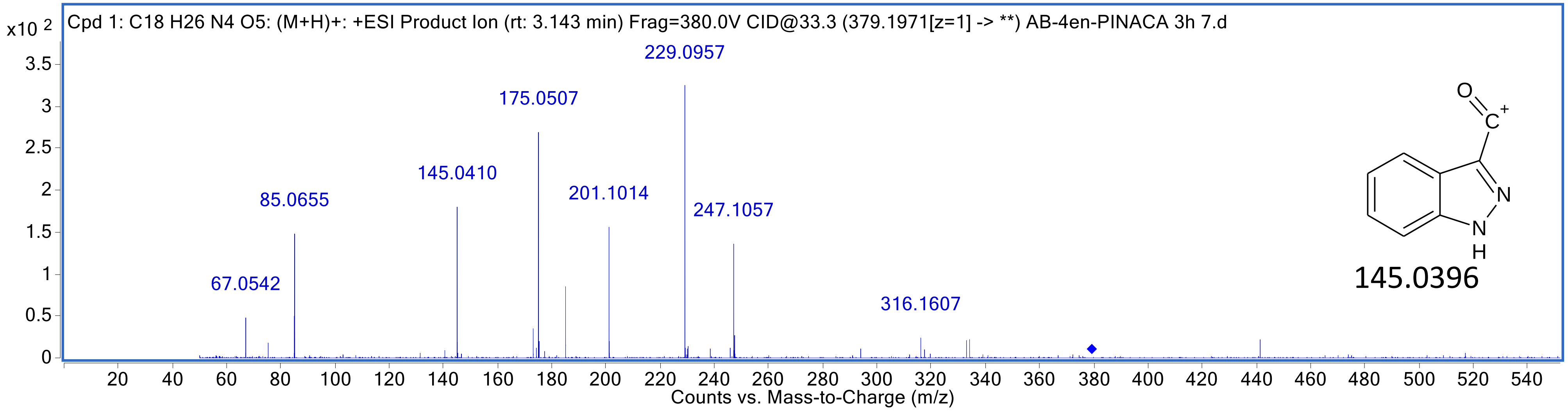

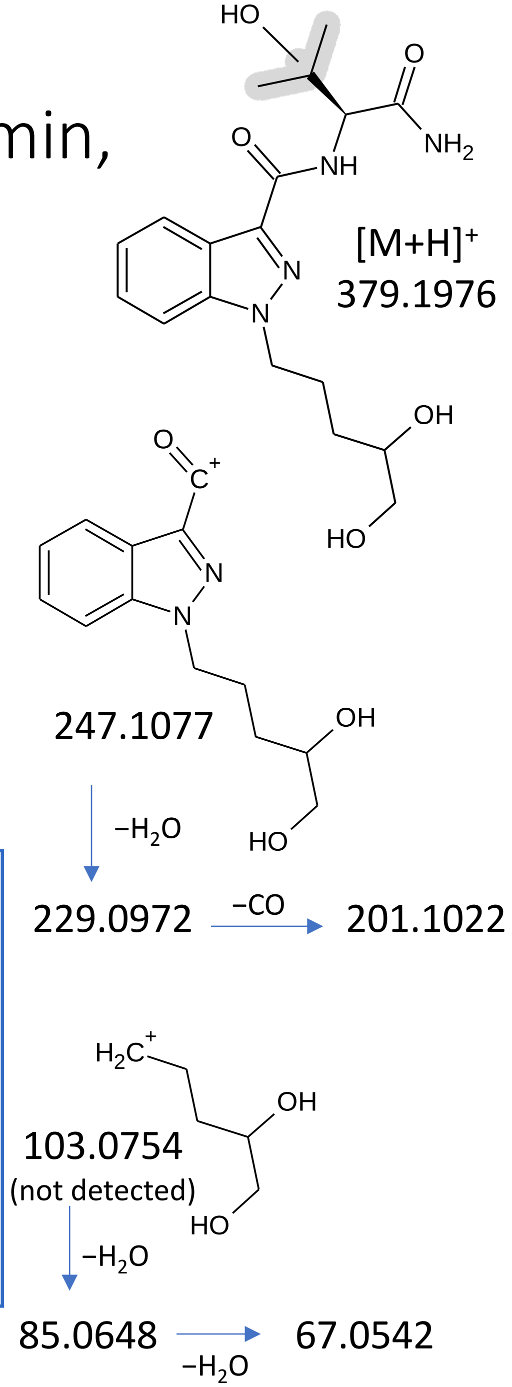

ADB-4en-PICA

Metabolism

ADB-4en-PICA, RT 9.06 min, *m/z* 342.2210

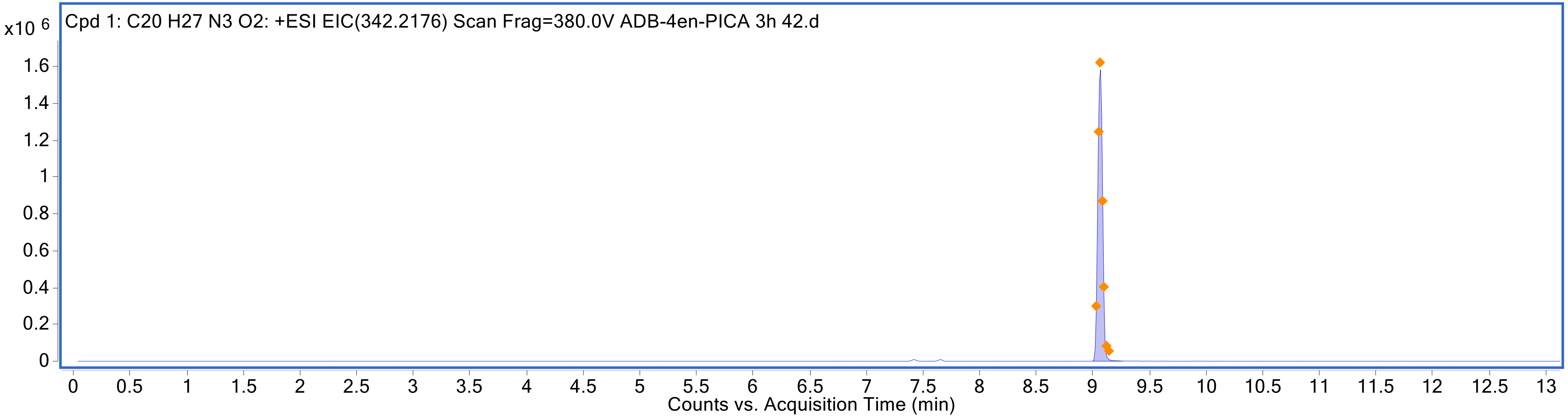

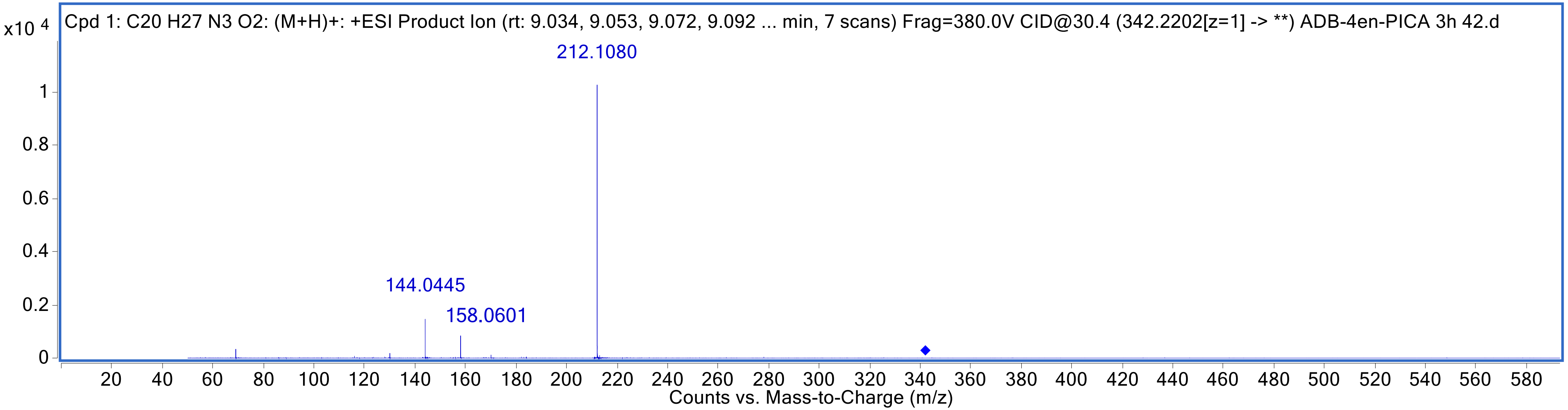

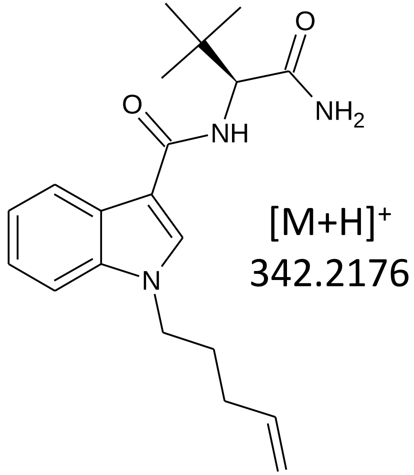

$[M+H]^+$
342.2176

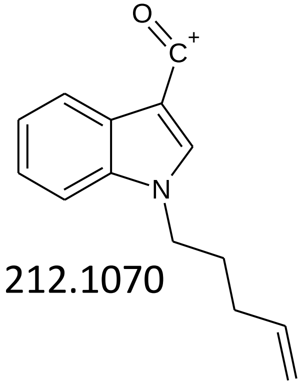

212.1070

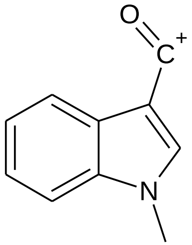

158.0600

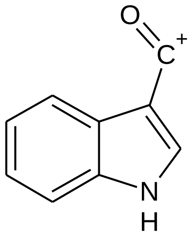

144.0444

I1, Mono-hydroxylation (pentenyl tail), RT 6.30 min, *m/z* 358.2133

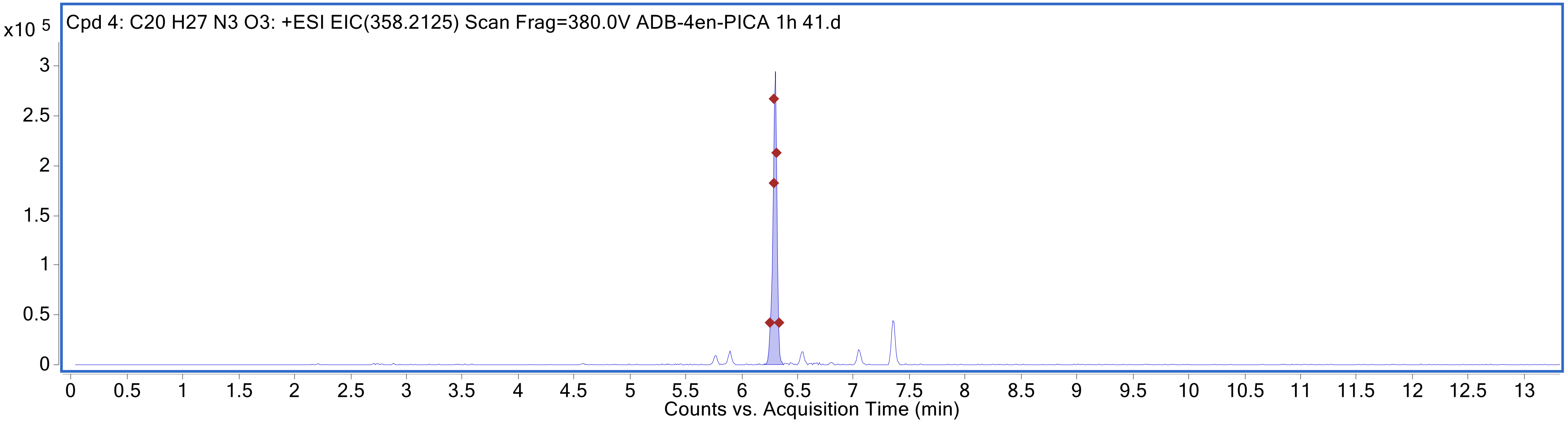

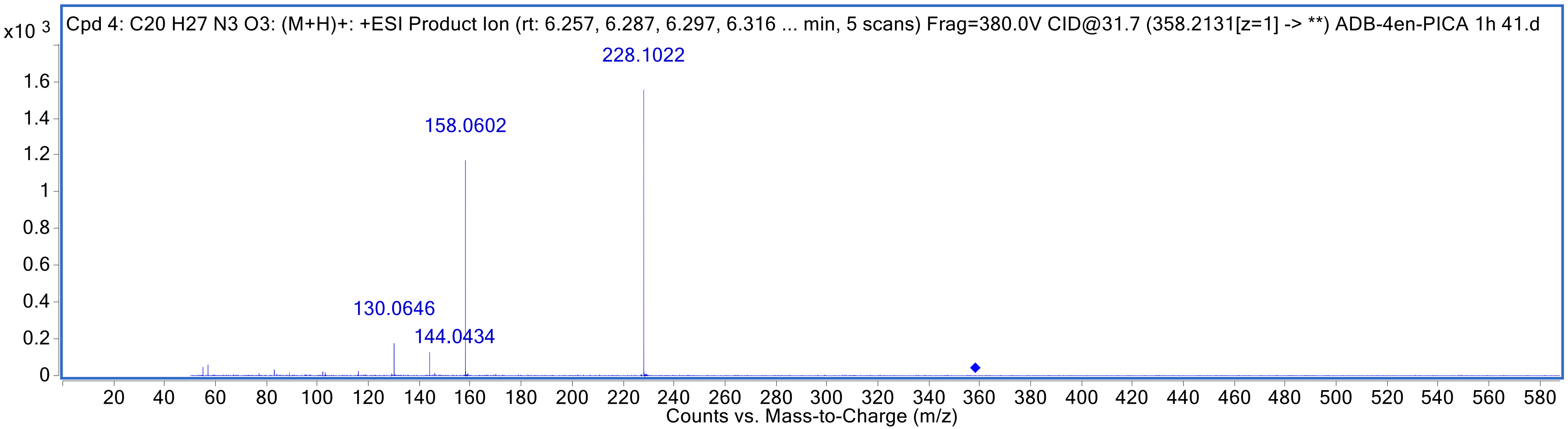

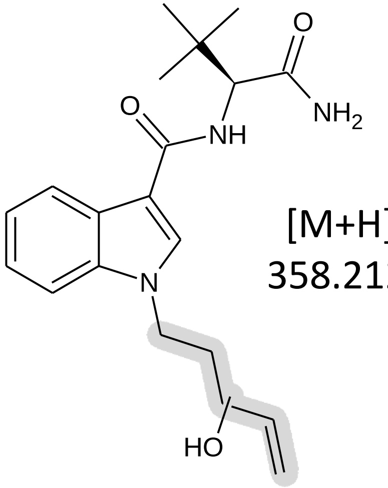

$[M+H]^+$
358.2125

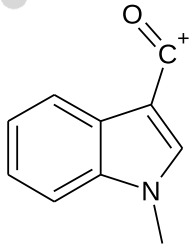

158.0600

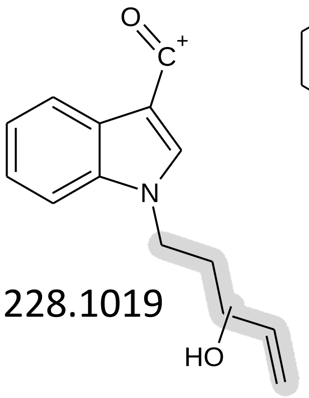

228.1019

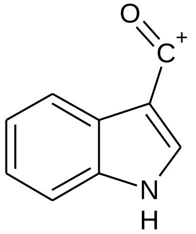

144.0444

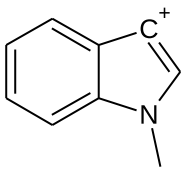

130.0651

I2, Dihydrodiol formation, RT 4.69 min, *m/z* 376.2238

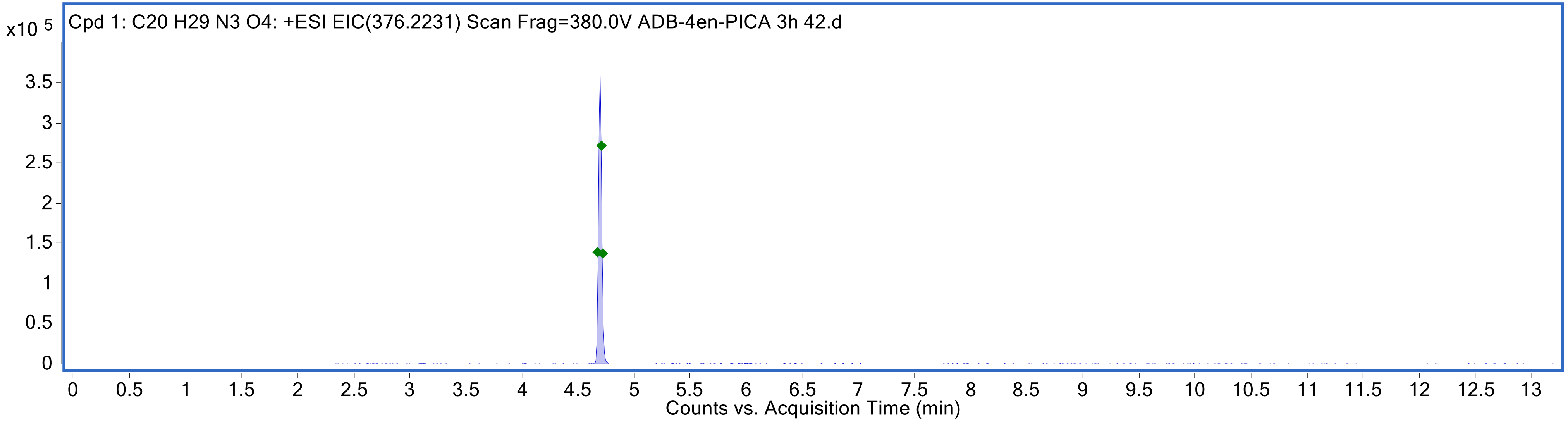

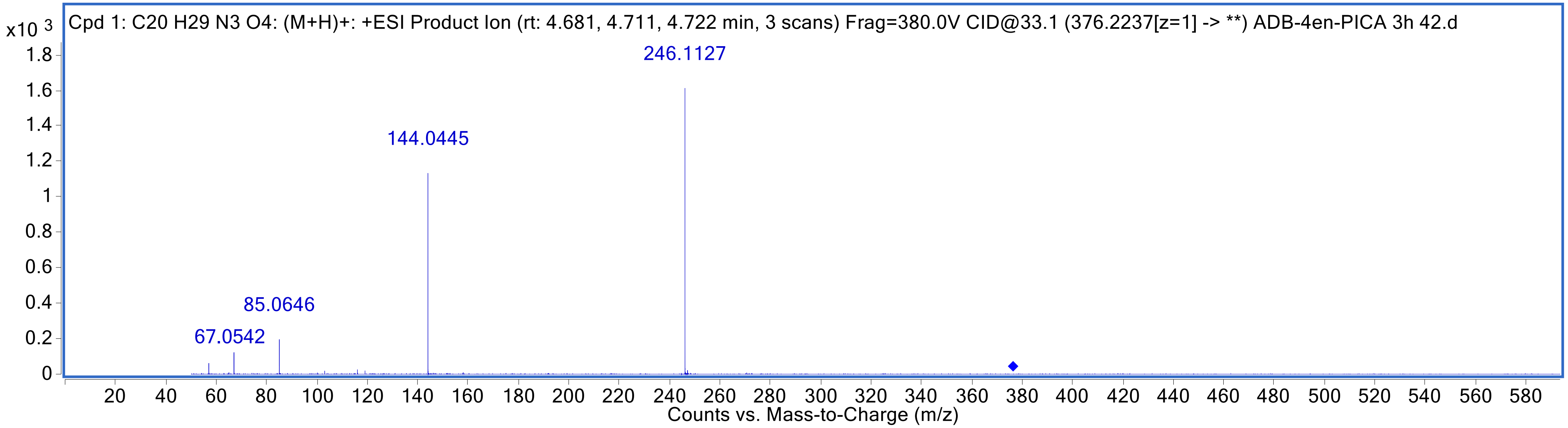

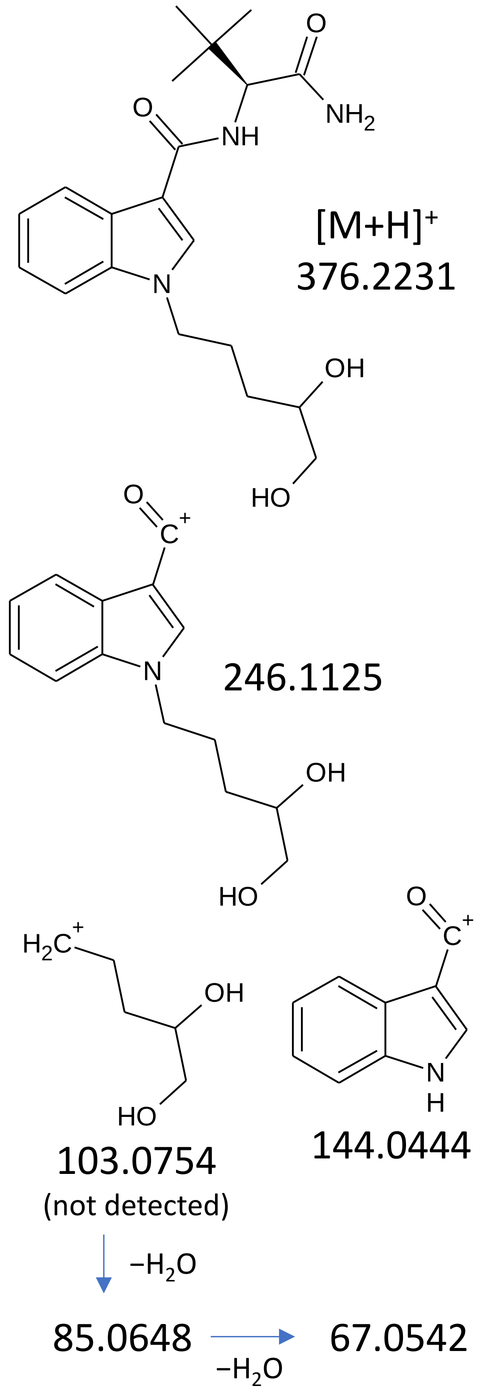

Dihydrodiol reference standard, RT 4.70 min, *m/z* 376.2228

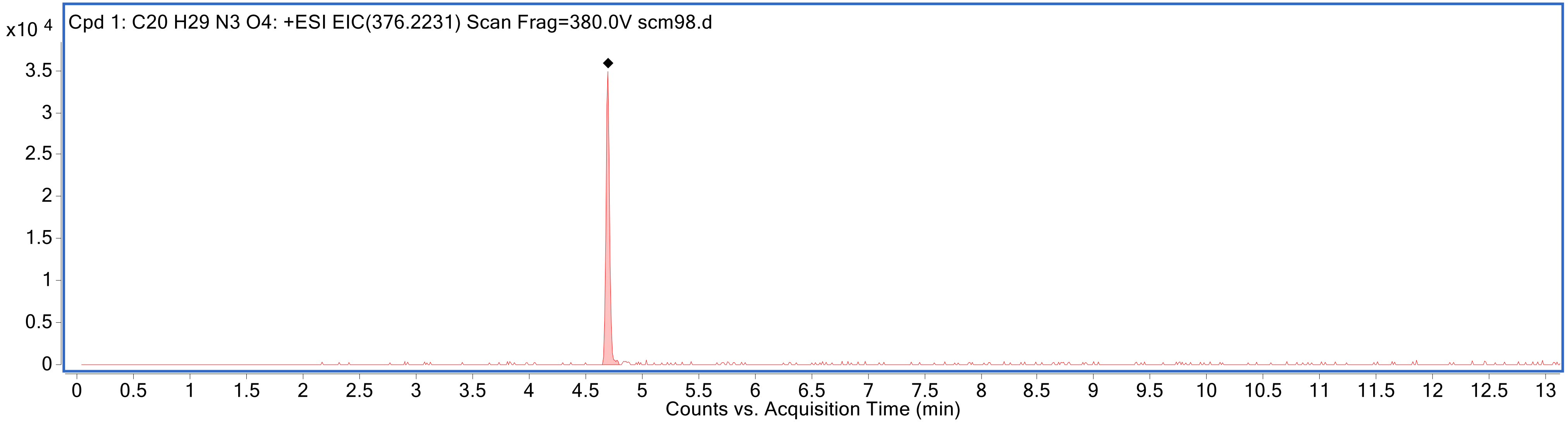

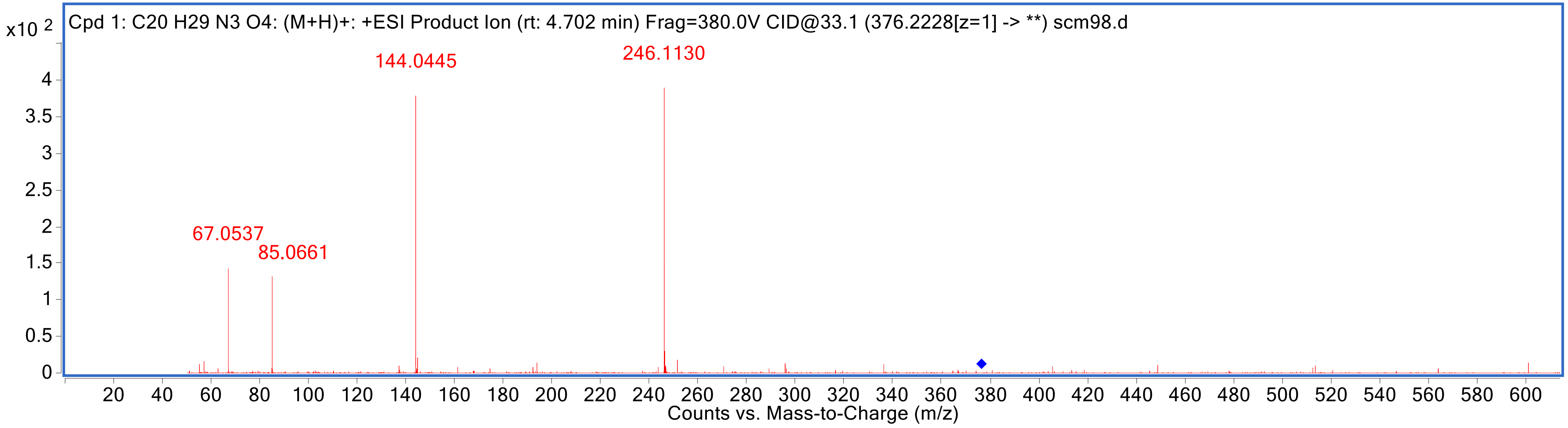

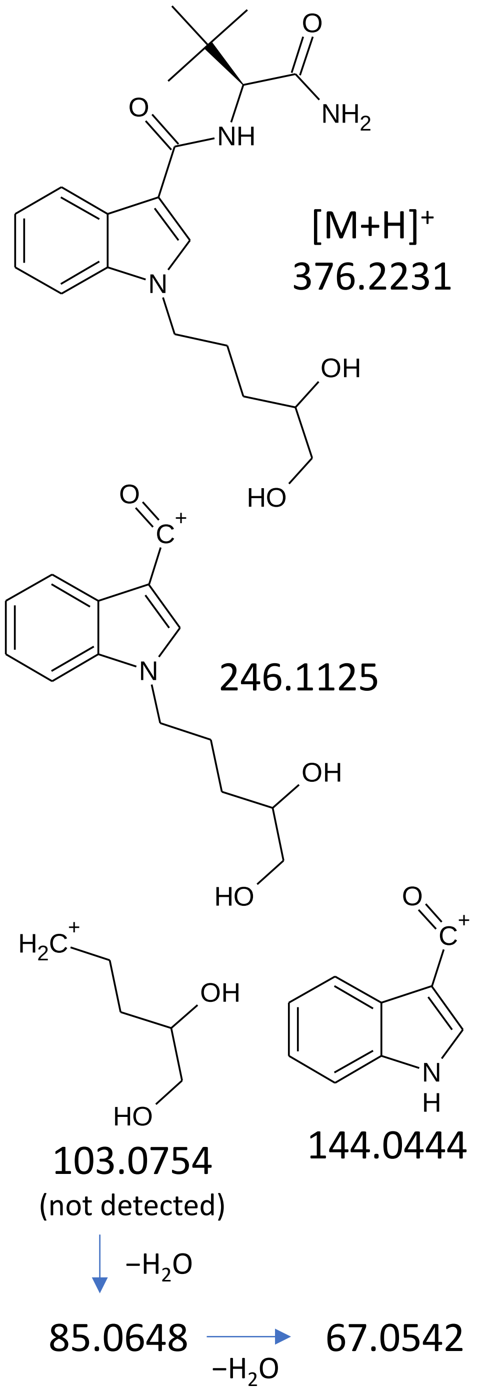

I3, *N*-dealkylation, RT 4.82 min, *m/z* 274.1555

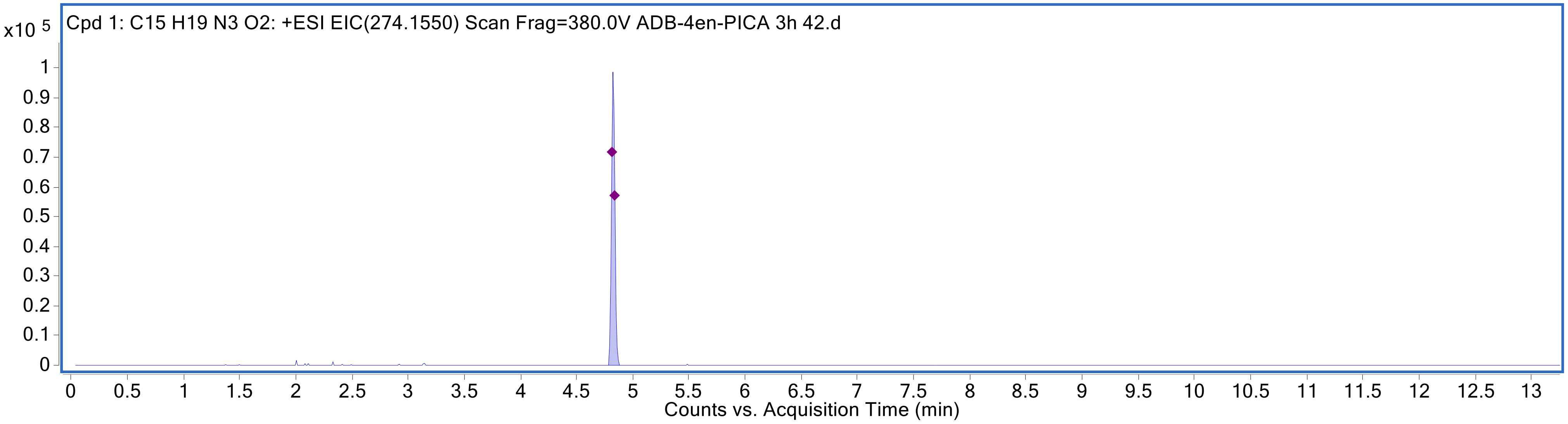

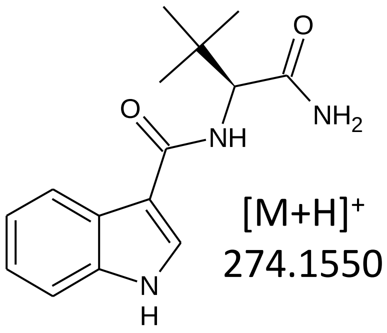

[M+H]+
274.1550

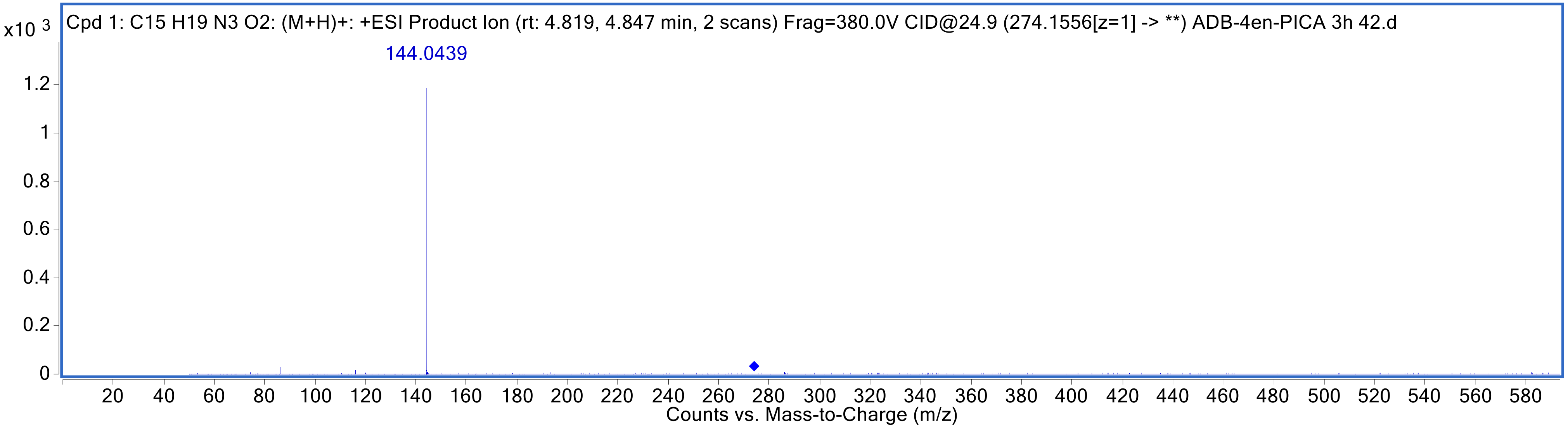

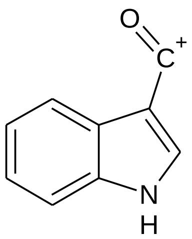

144.0444

I4, Mono-hydroxylation (*tert*-butyl), RT 7.36 min, *m/z* 358.2131

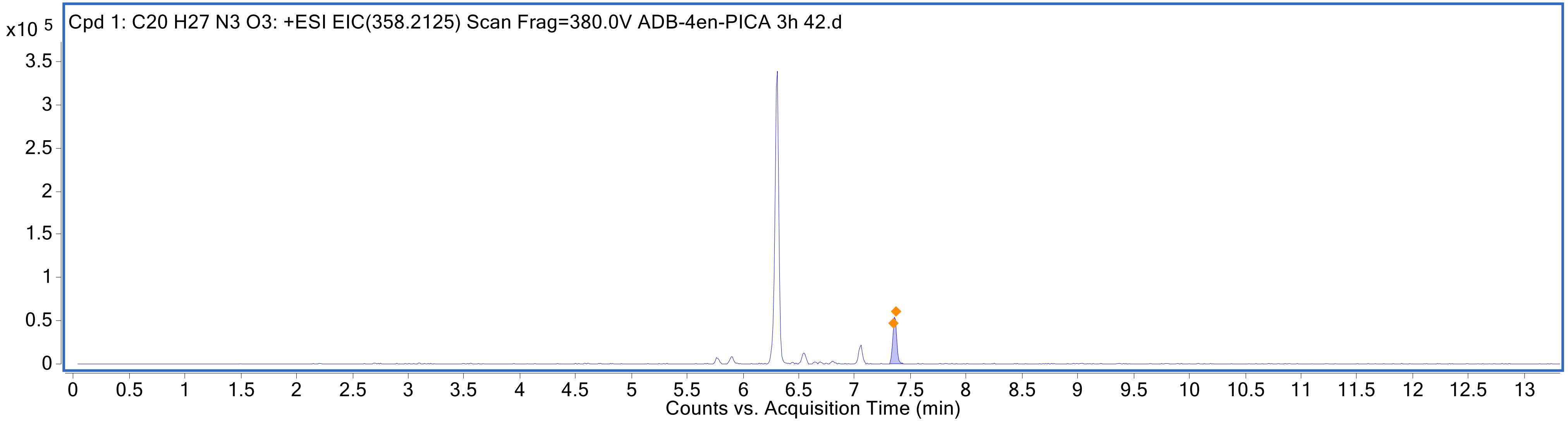

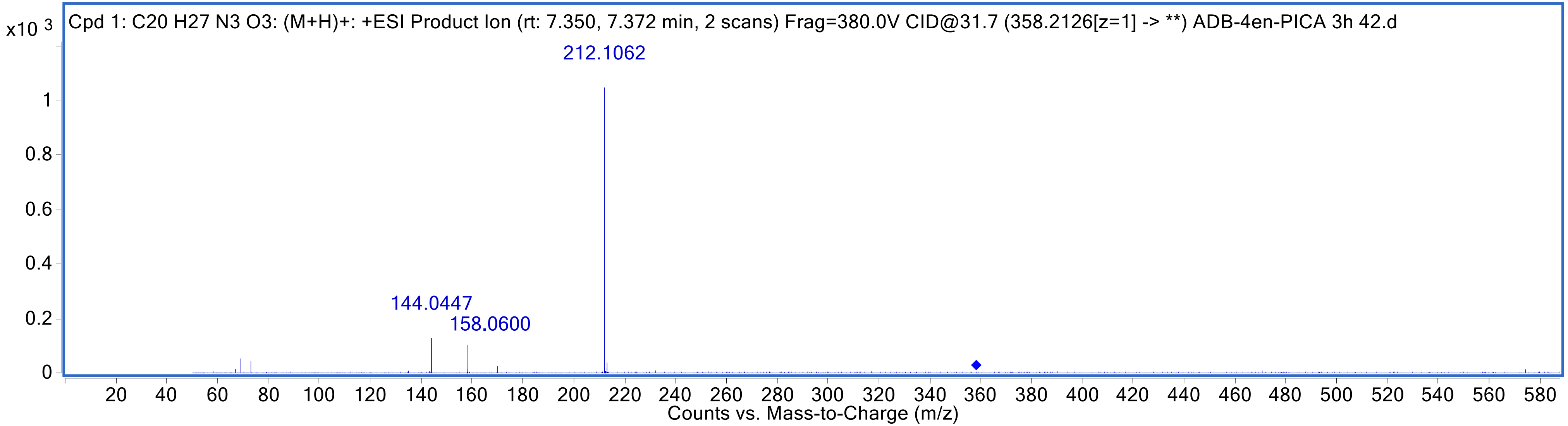

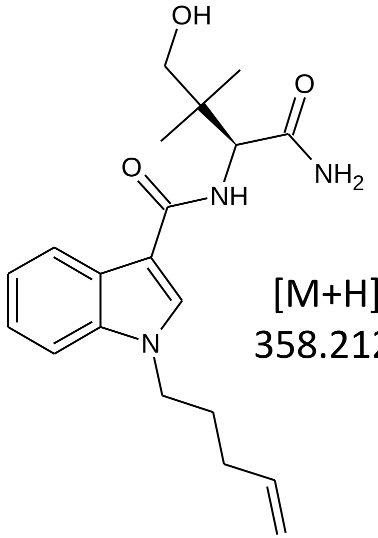

$[M+H]^+$

358.2125

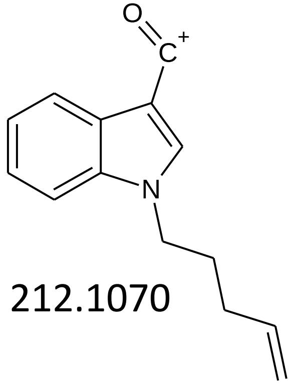

212.1070

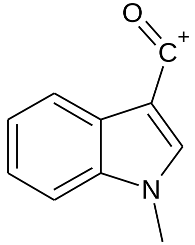

158.0600

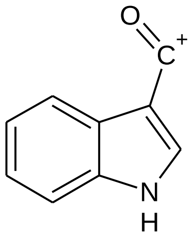

144.0444

I5, Mono-hydroxylation (indole core) + glucuronidation, RT 4.49 min, *m/z* 534.2440

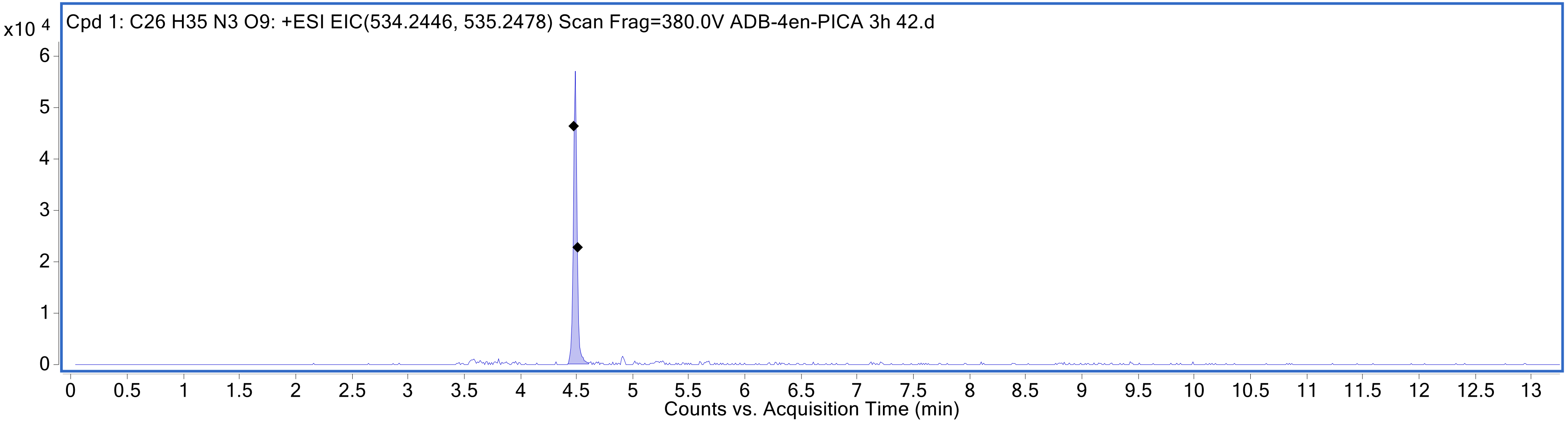

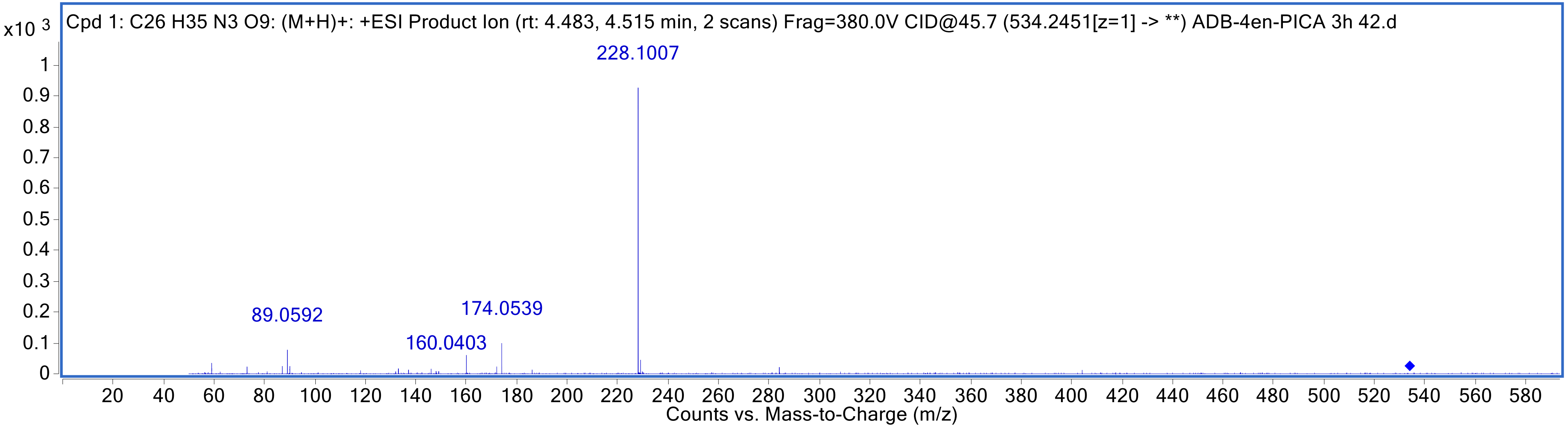

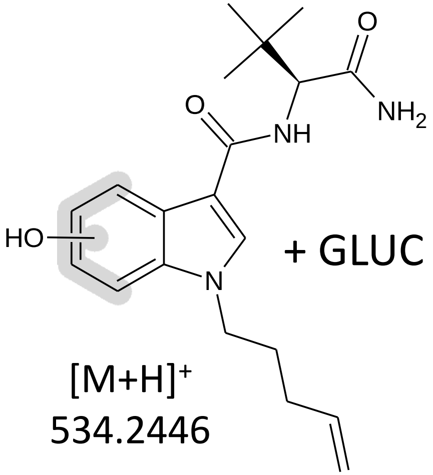

[M+H]+
534.2446

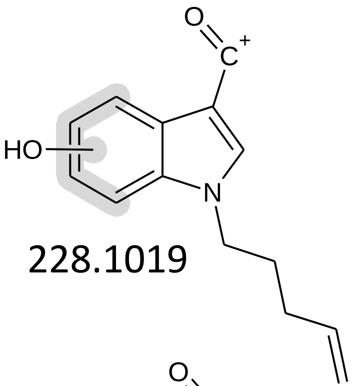

228.1019

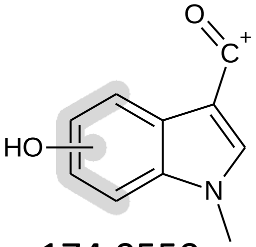

174.0550

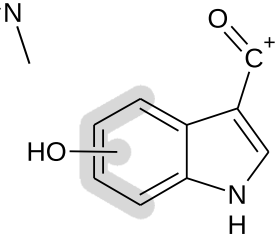

160.0393

I6, Mono-hydroxylation (pentenyl tail), RT 7.05 min, *m/z* 358.2122

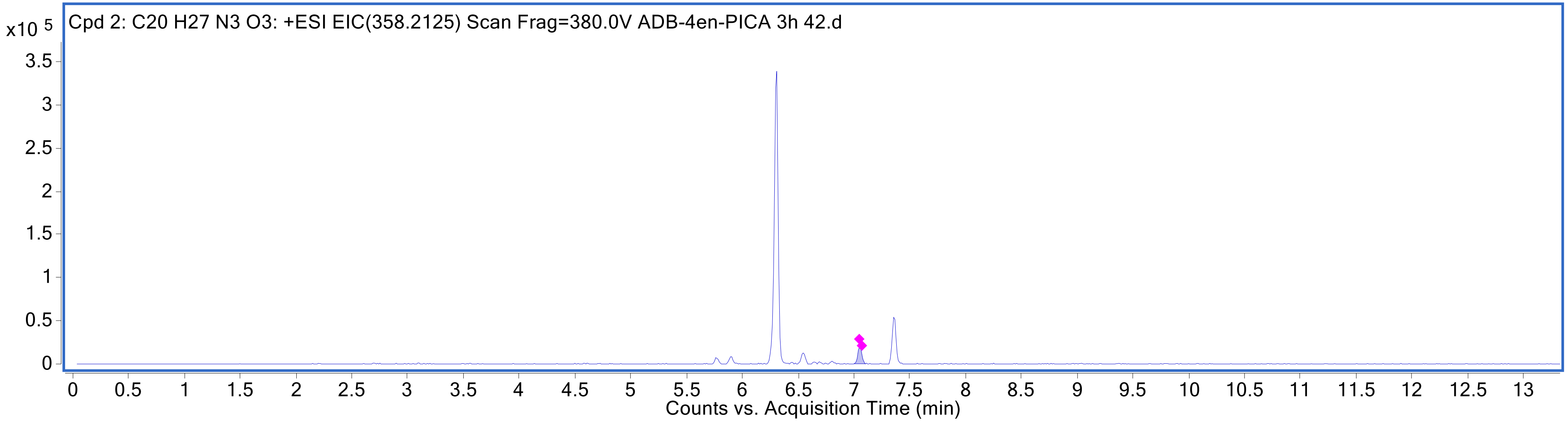

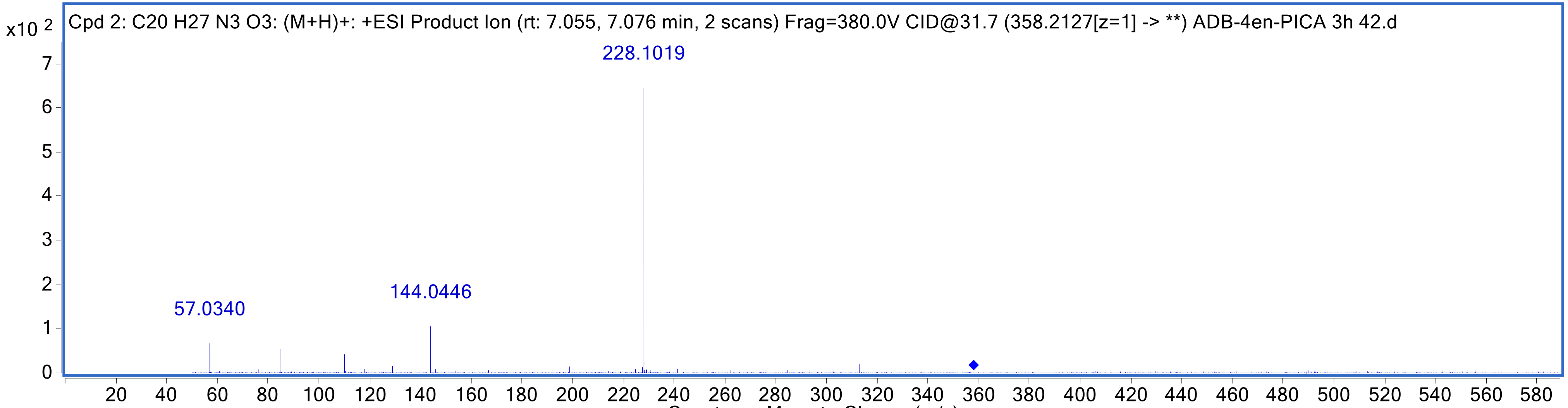

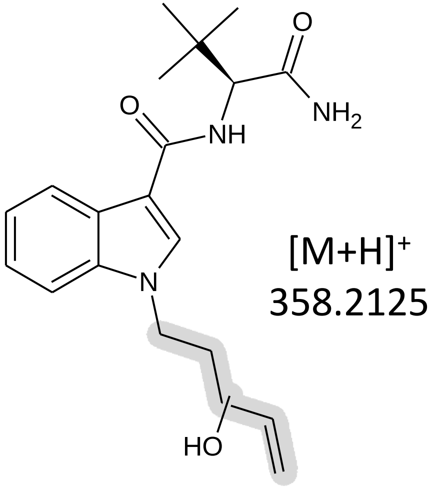

[M+H]+
358.2125

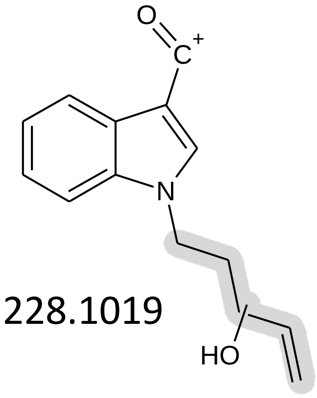

228.1019

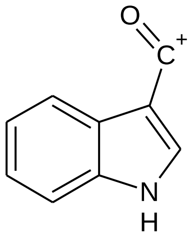

144.0444

I7, Dihydrodiol formation + mono-hydroxylation (indole core) + glucuronidation, RT 2.58 min, *m/z* 568.2495

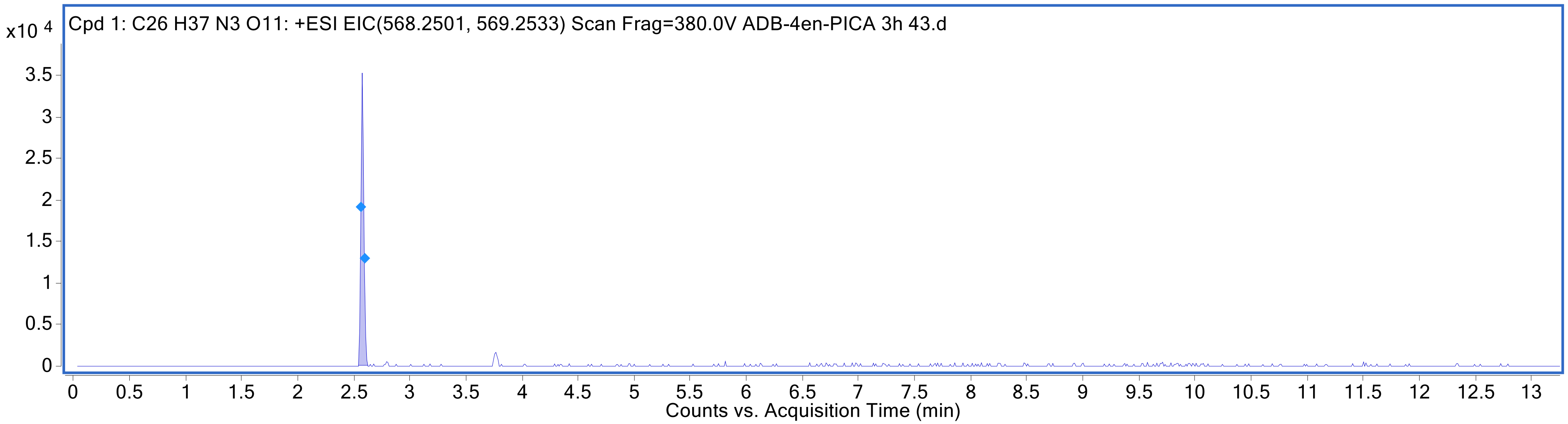

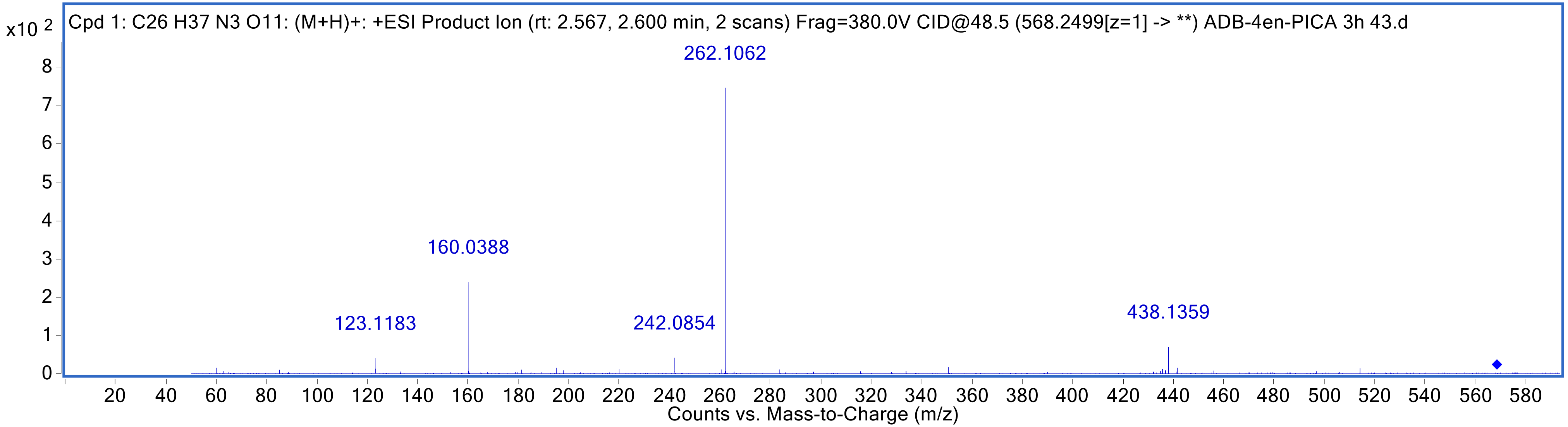

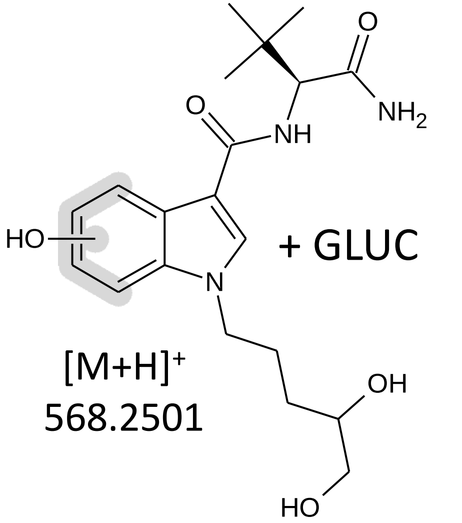

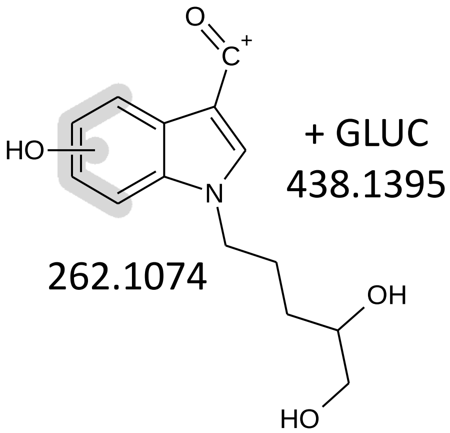

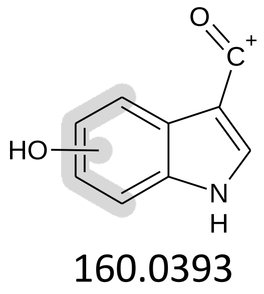

I8, Di-hydroxylation (pentenyl tail), RT 5.91 min, *m/z* 374.2073

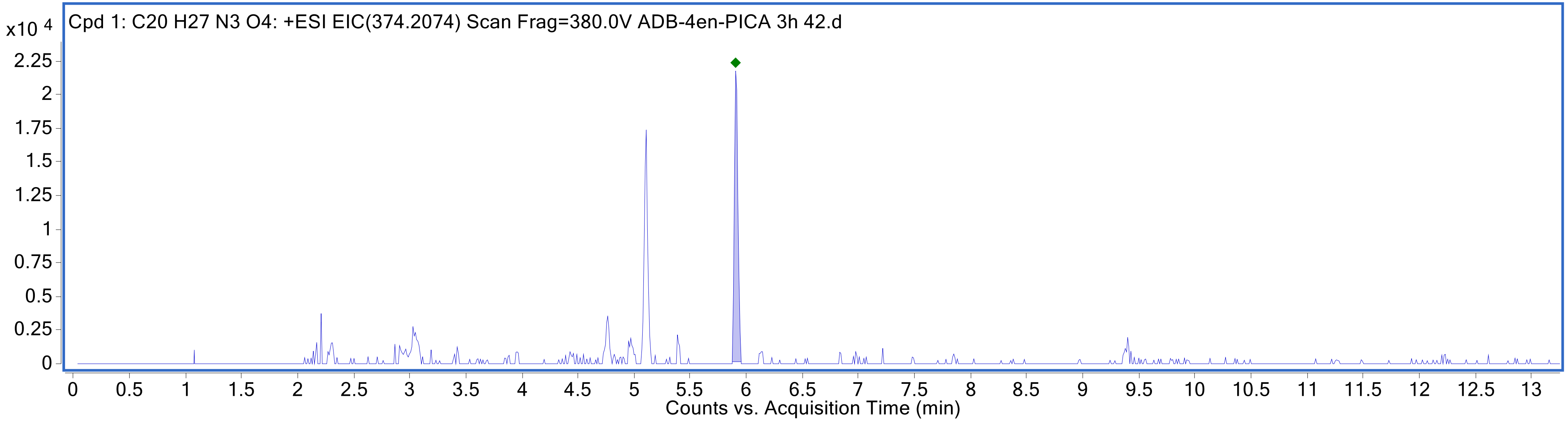

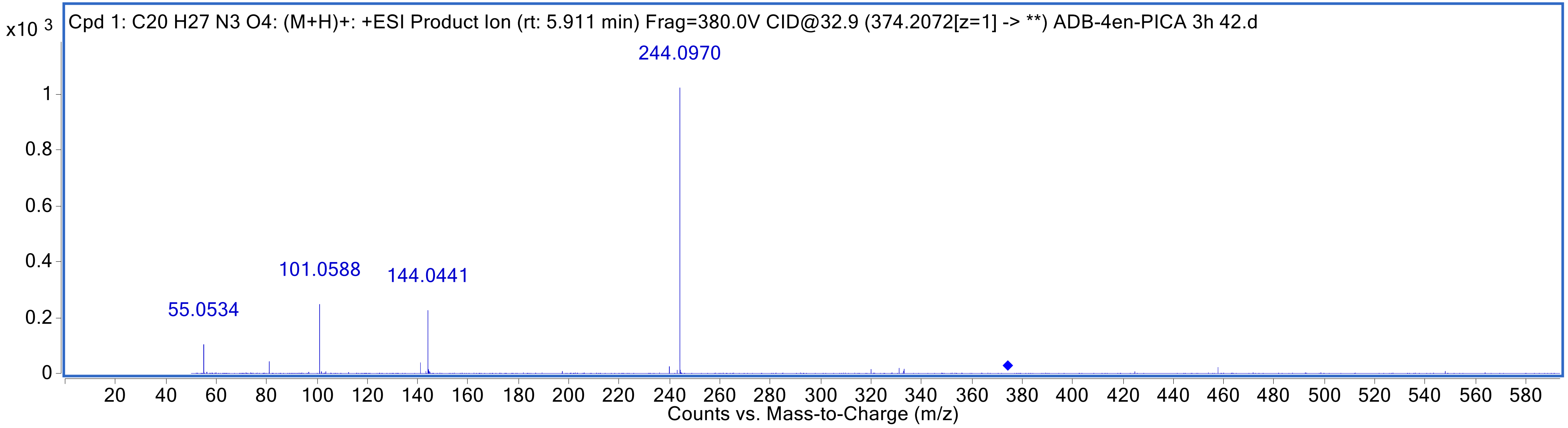

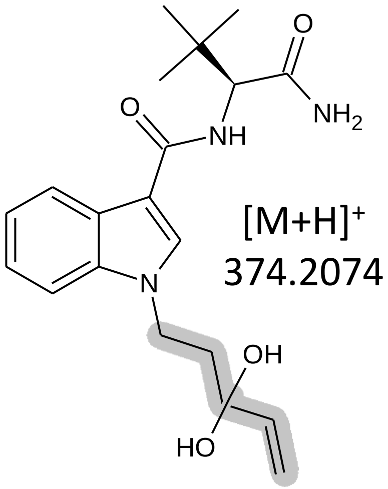

[M+H]+
374.2074

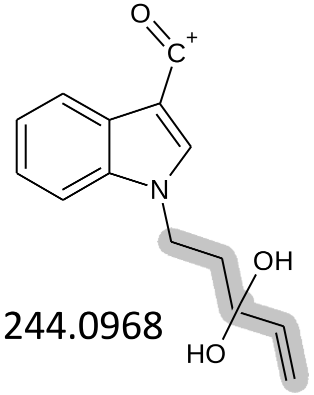

244.0968

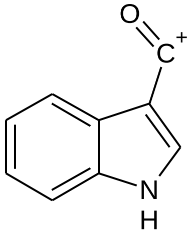

144.0444

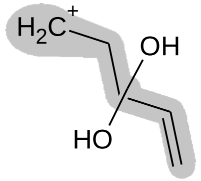

101.0597

I9, Mono-hydroxylation (indole core), RT 6.54 min, *m/z* 358.2118

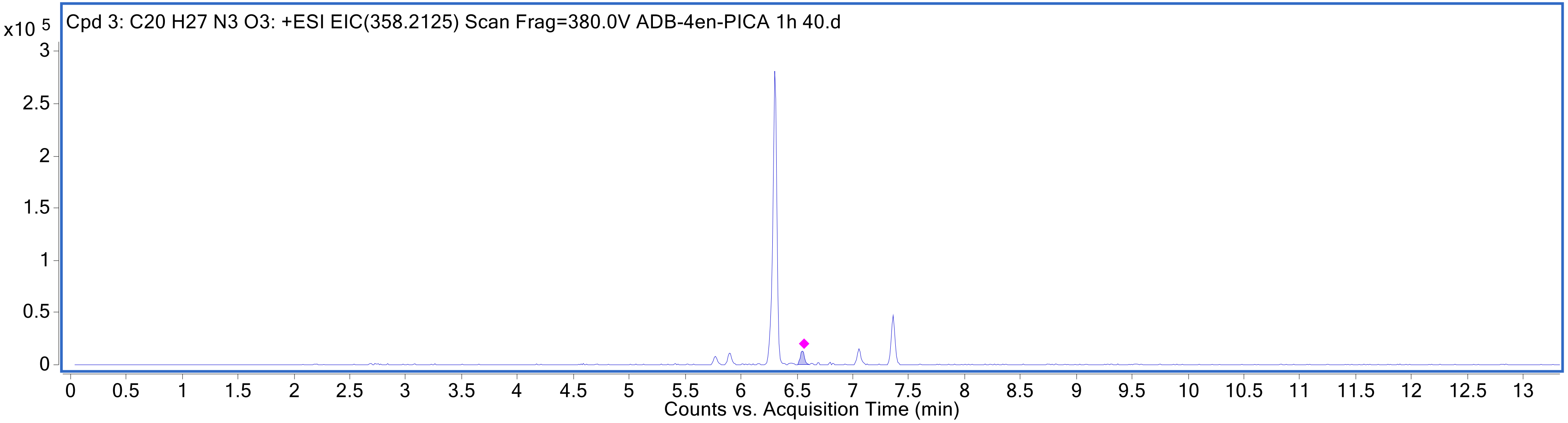

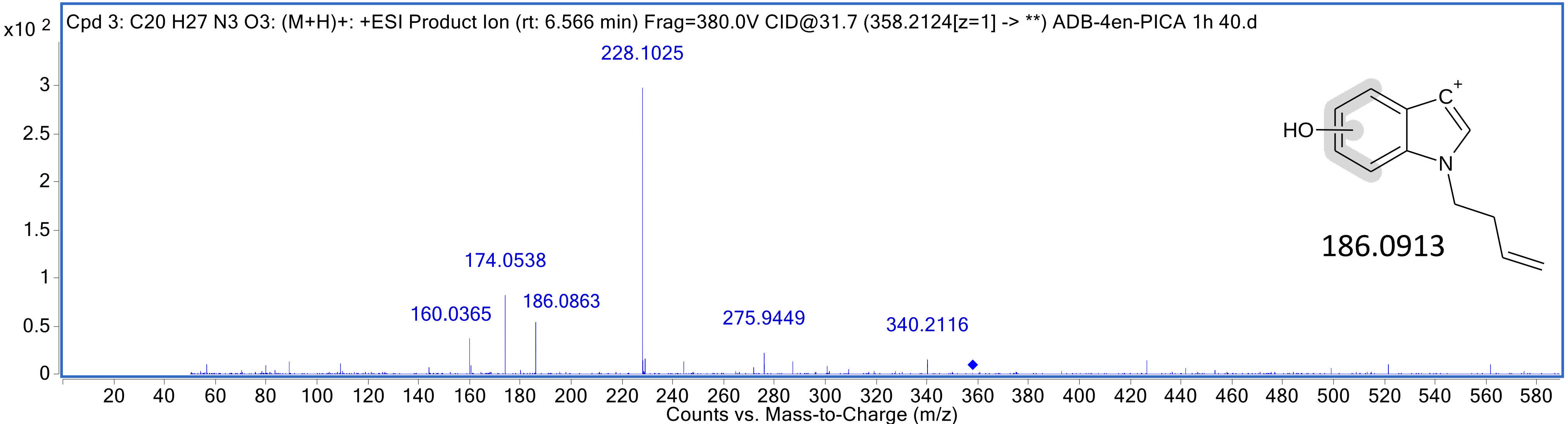

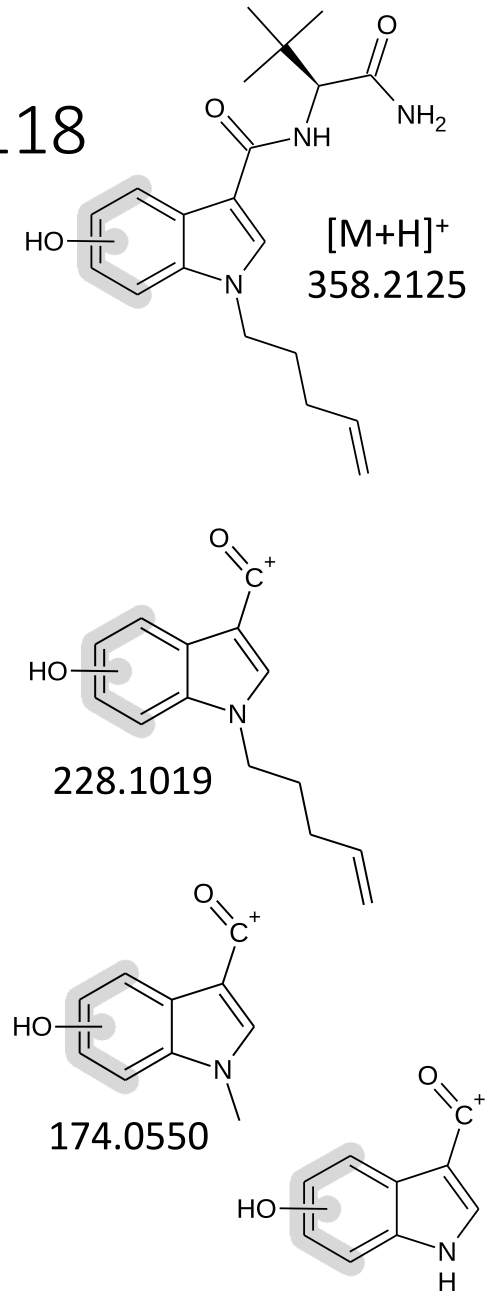

I10, Dihydrodiol formation + mono-hydroxylation (*tert*-butyl), RT 3.42 min, *m/z* 392.2178

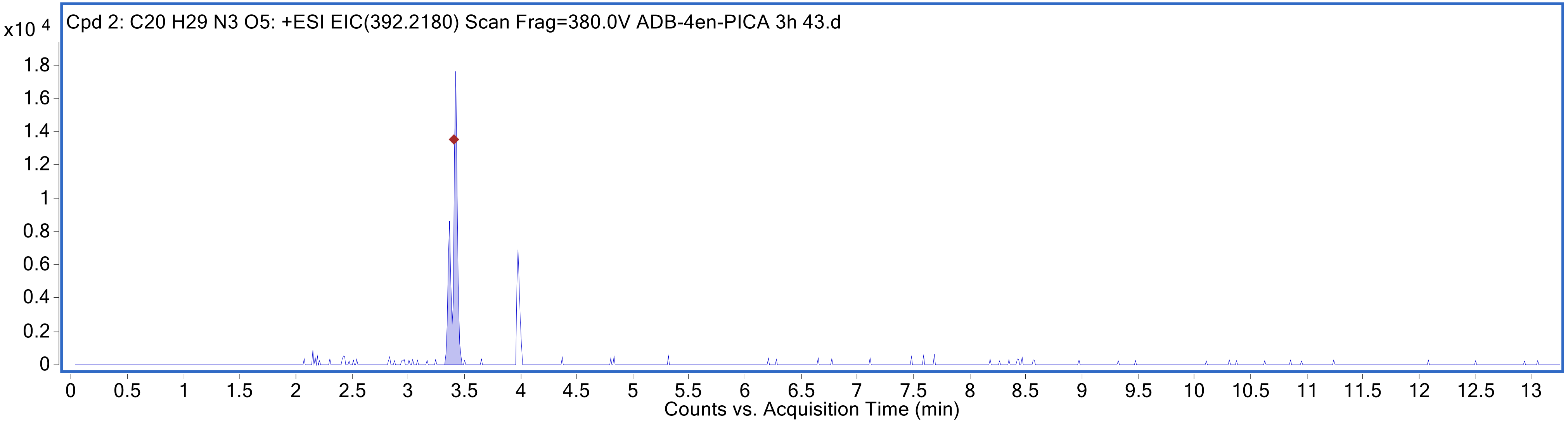

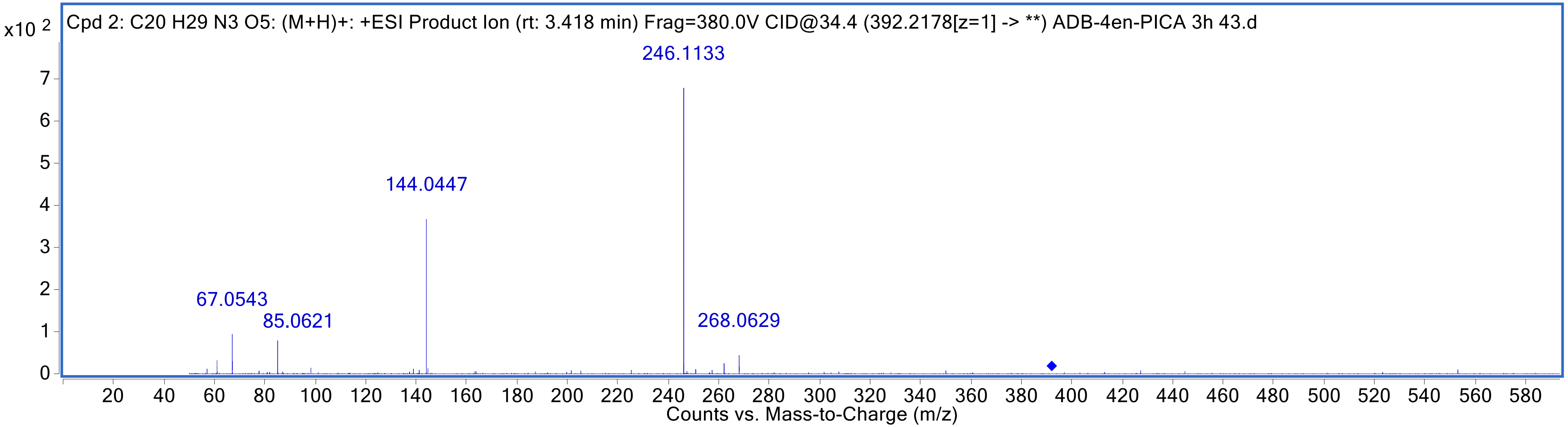

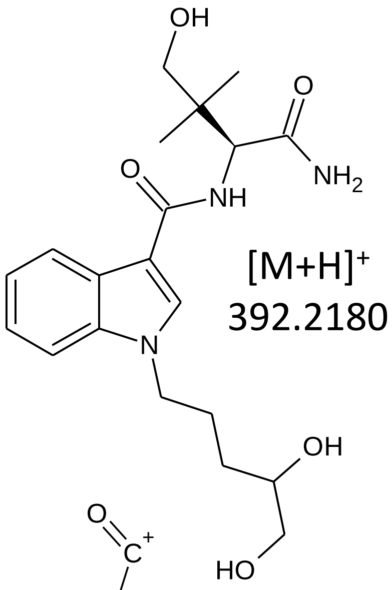

$[M+H]^+$
392.2180

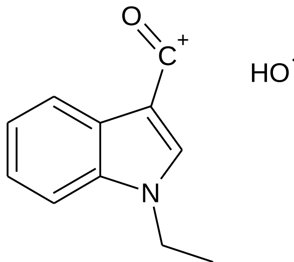

246.1125

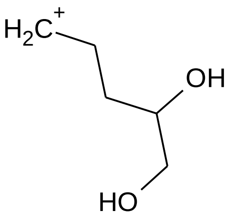

103.0754
(not detected)

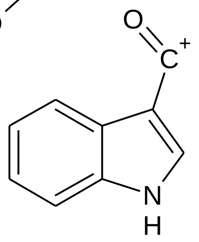

144.0444

$-H_2O$

85.0648 → 67.0542

$-H_2O$

I11, Di-hydroxylation (pentenyl tail), RT 5.10 min, *m/z* 374.2069

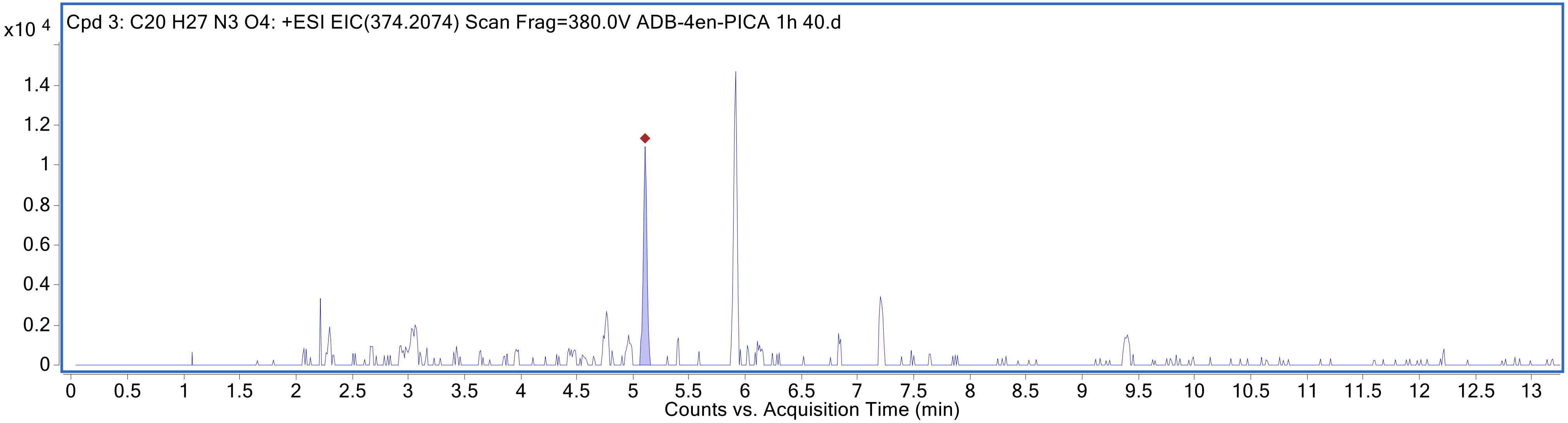

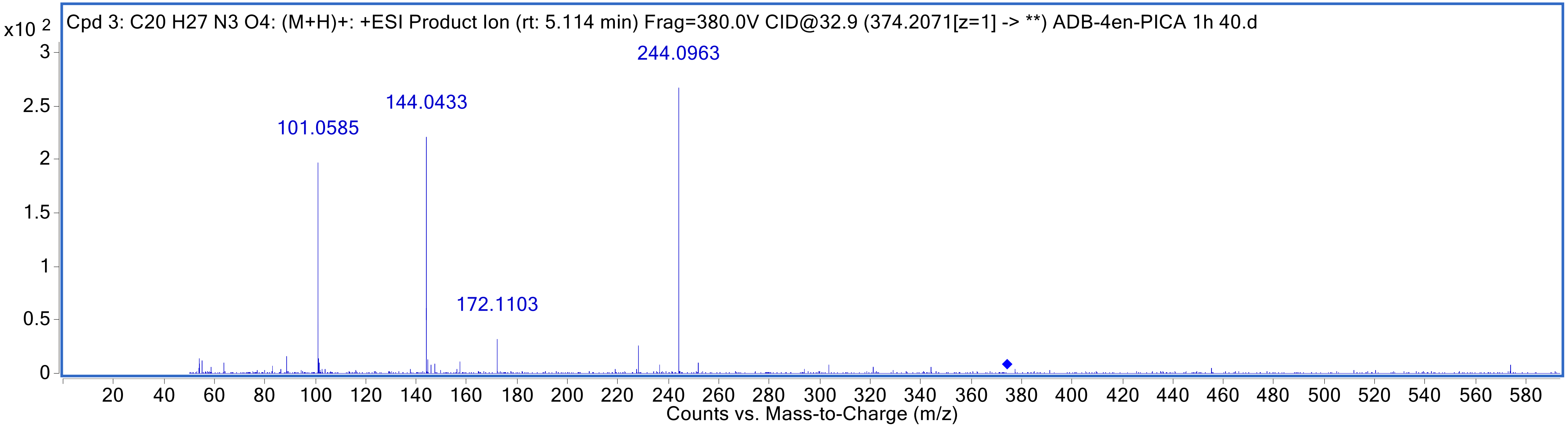

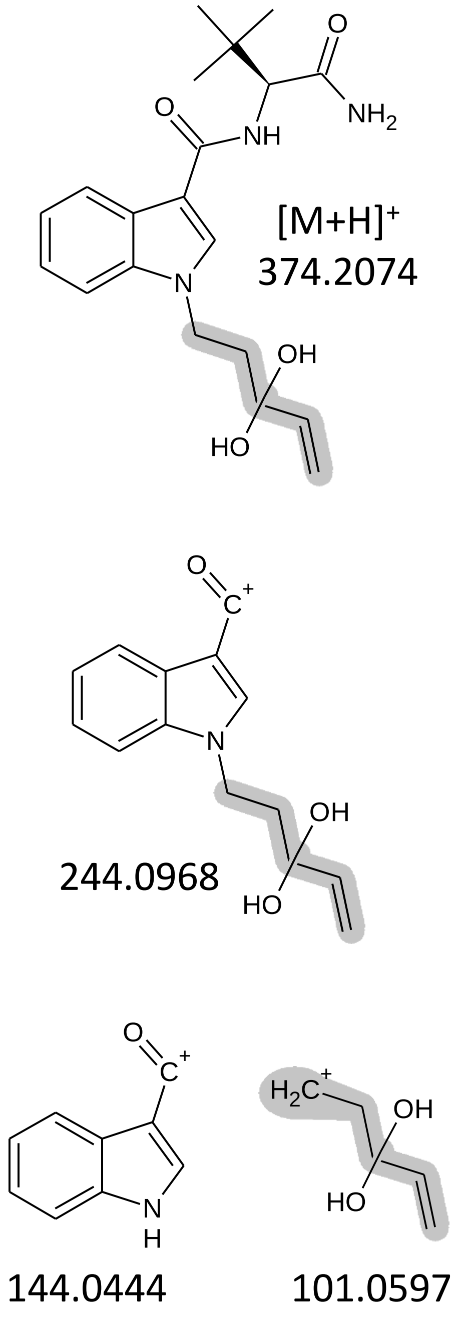

I12, Mono-hydroxylation (pentenyl tail), RT 5.76 min, *m/z* 358.2121

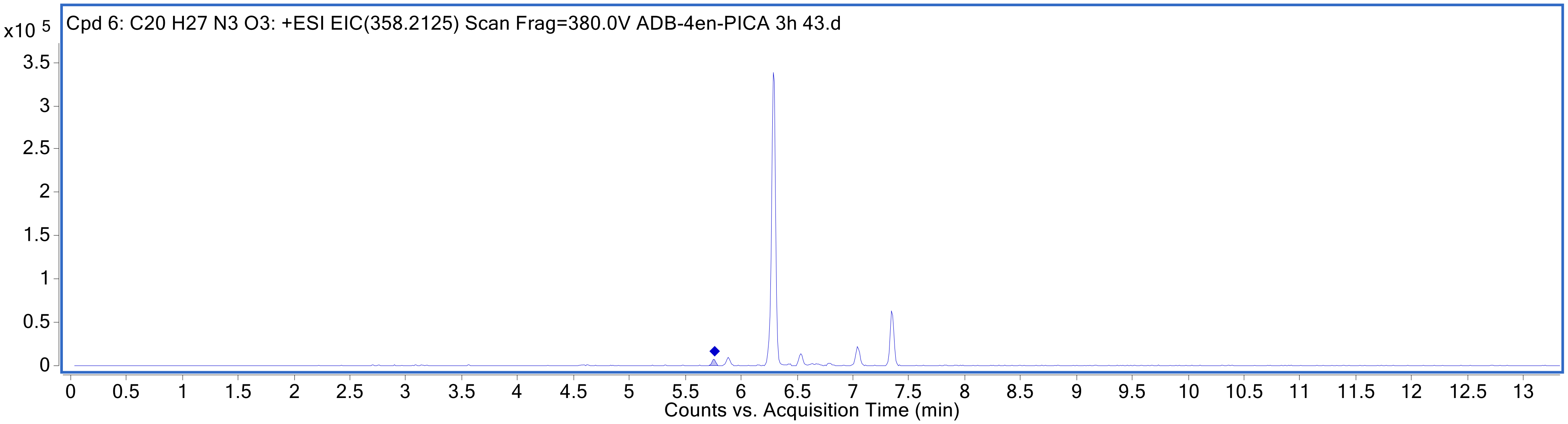

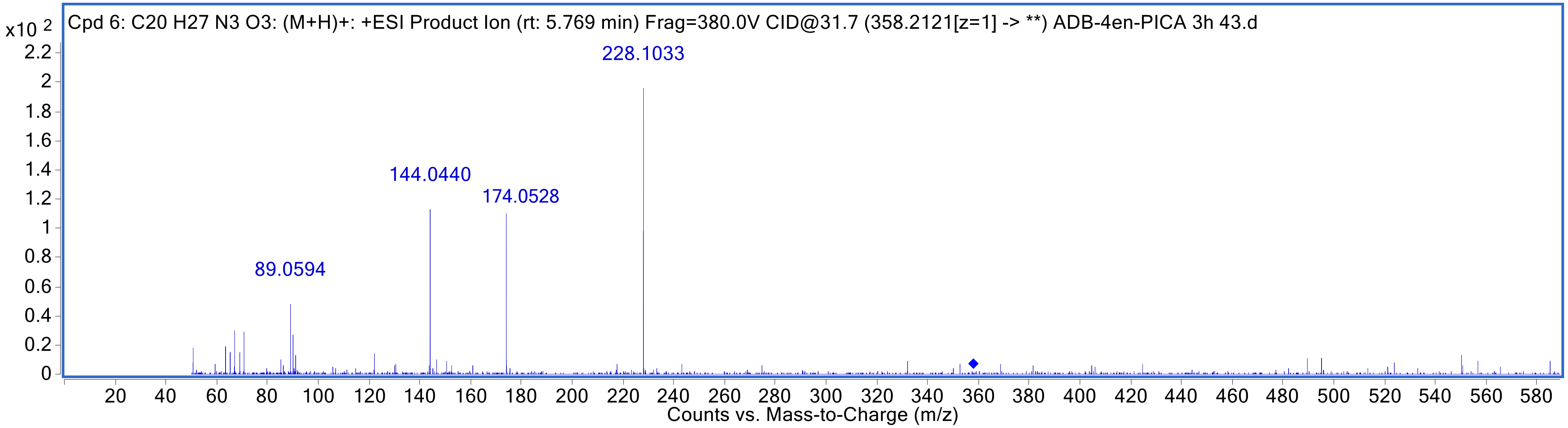

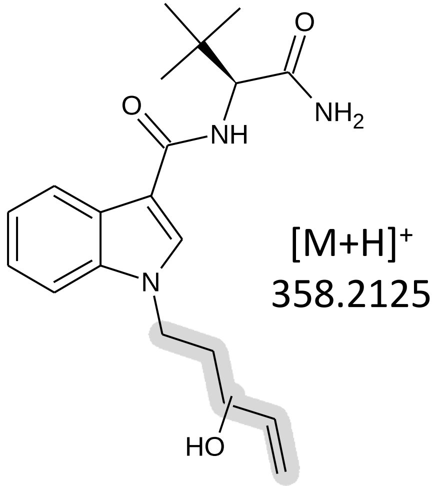

$[M+H]^+$
358.2125

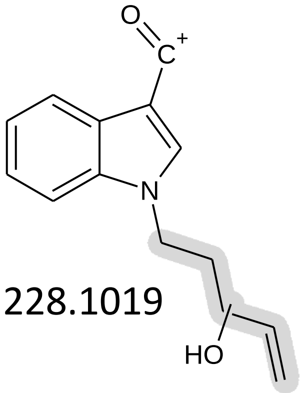

228.1019

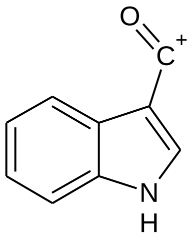

144.0444

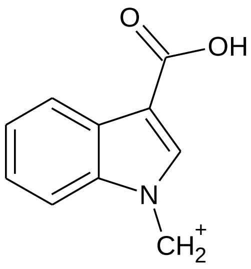

174.0550

ADB-4en-PINACA

Metabolism

ADB-4en-PINACA, RT 9.54 min, *m/z* 343.2150

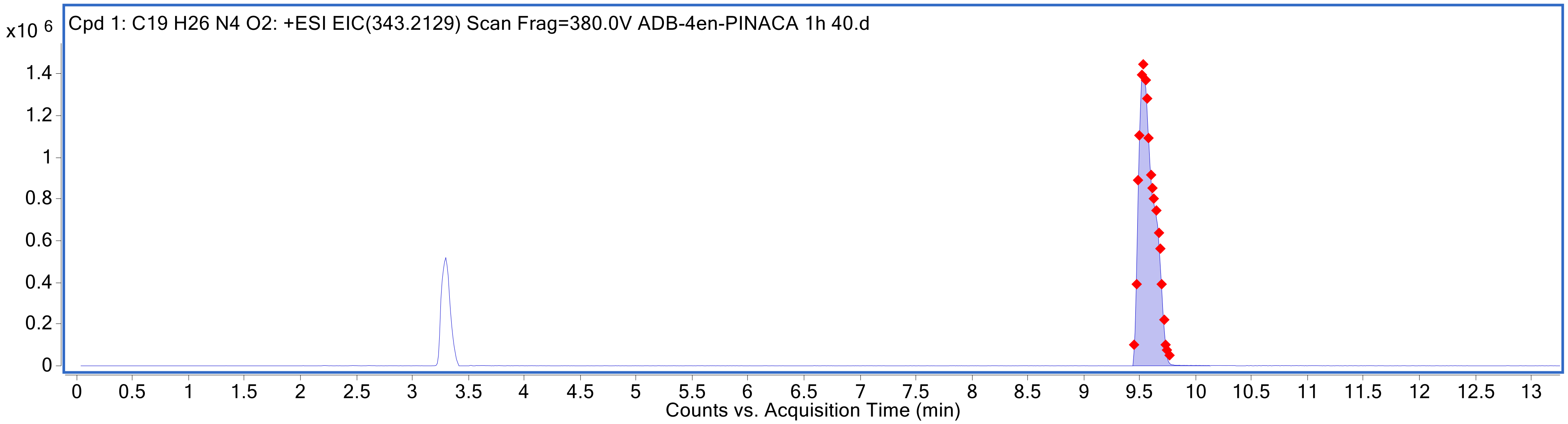

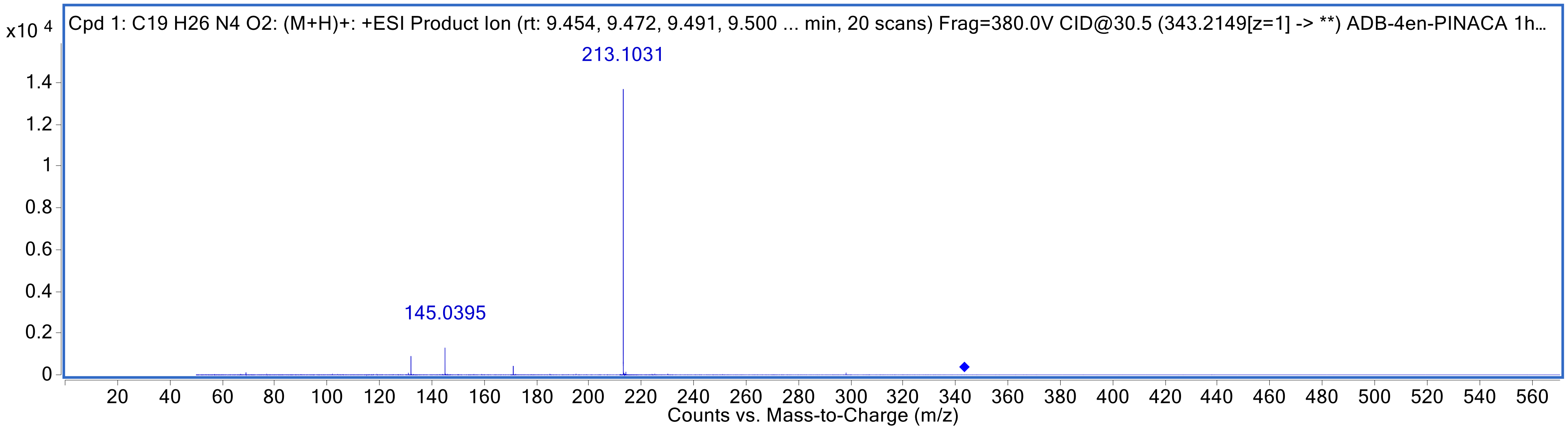

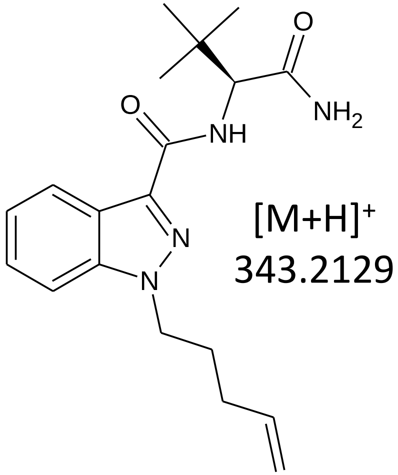

$[M+H]^+$
343.2129

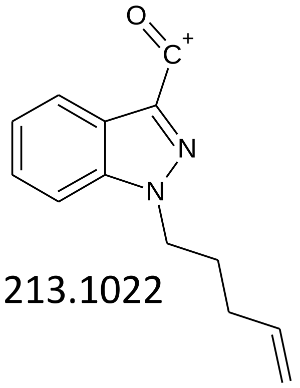

213.1022

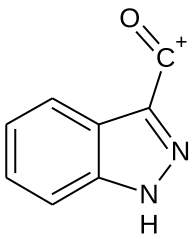

145.0396

J1, Mono-hydroxylation (pentenyl tail), RT 6.41 min, *m/z* 359.2081

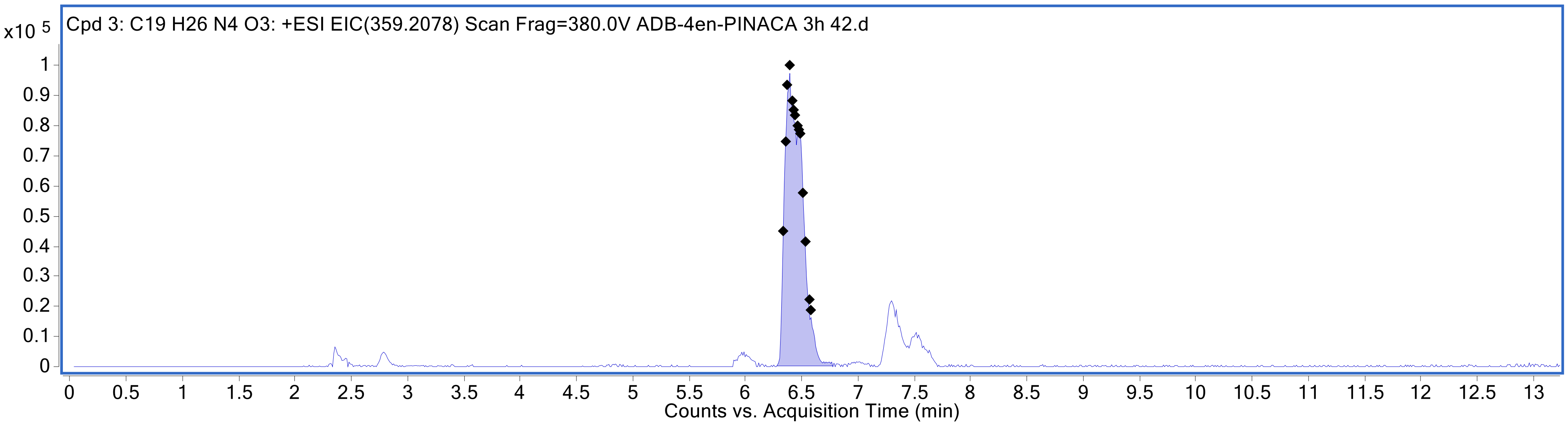

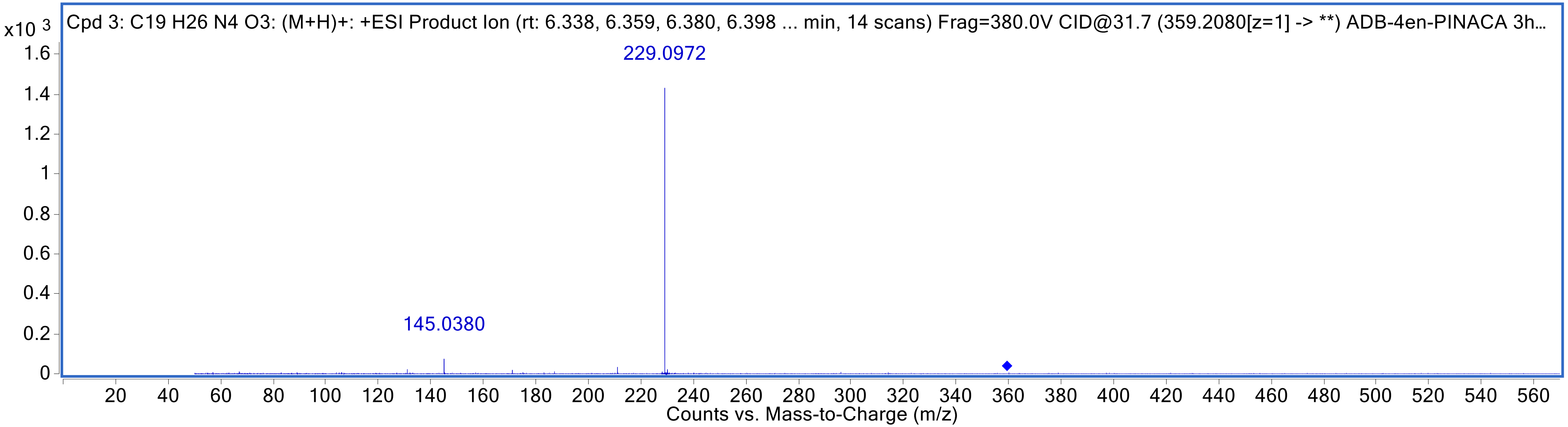

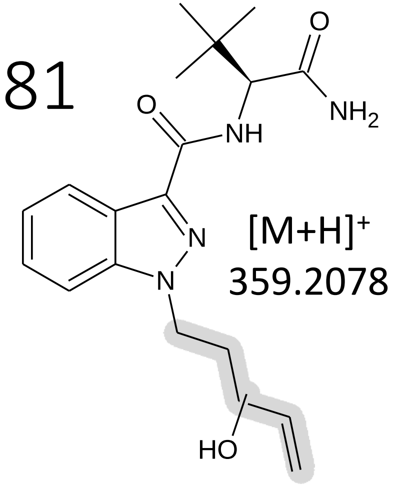

$[M+H]^+$
359.2078

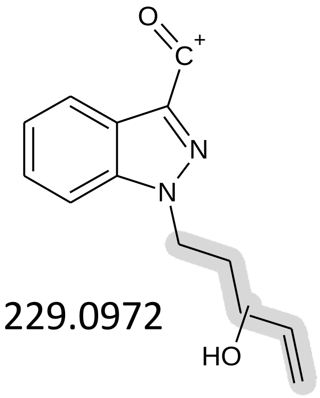

229.0972

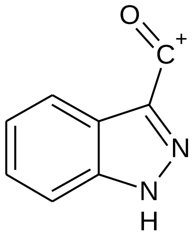

145.0396

J2, Dihydrodiol formation, RT 4.76 min, *m/z* 377.2184

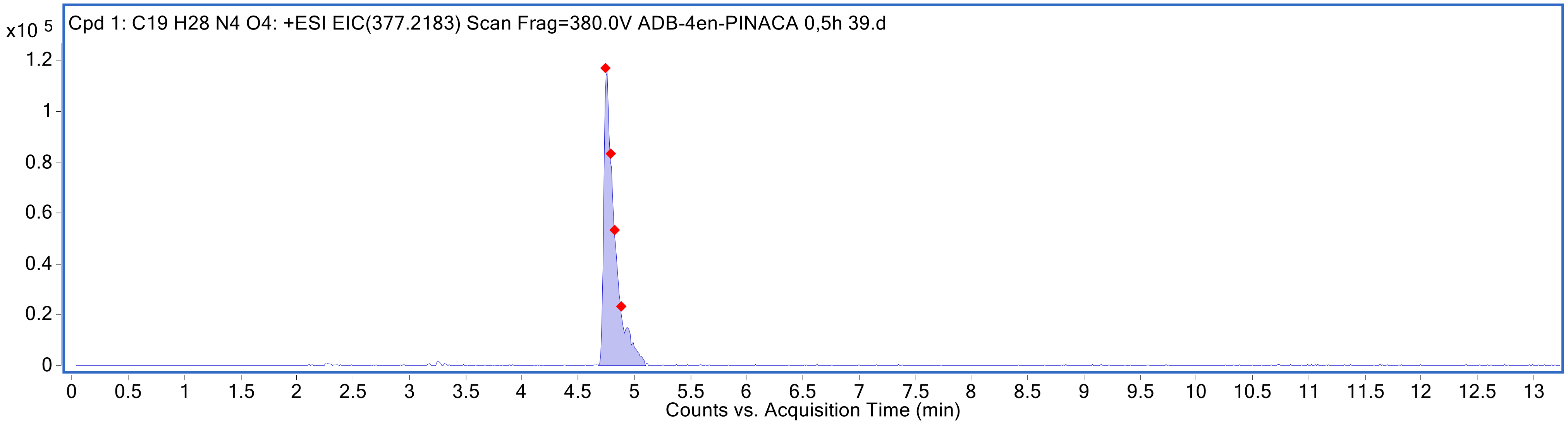

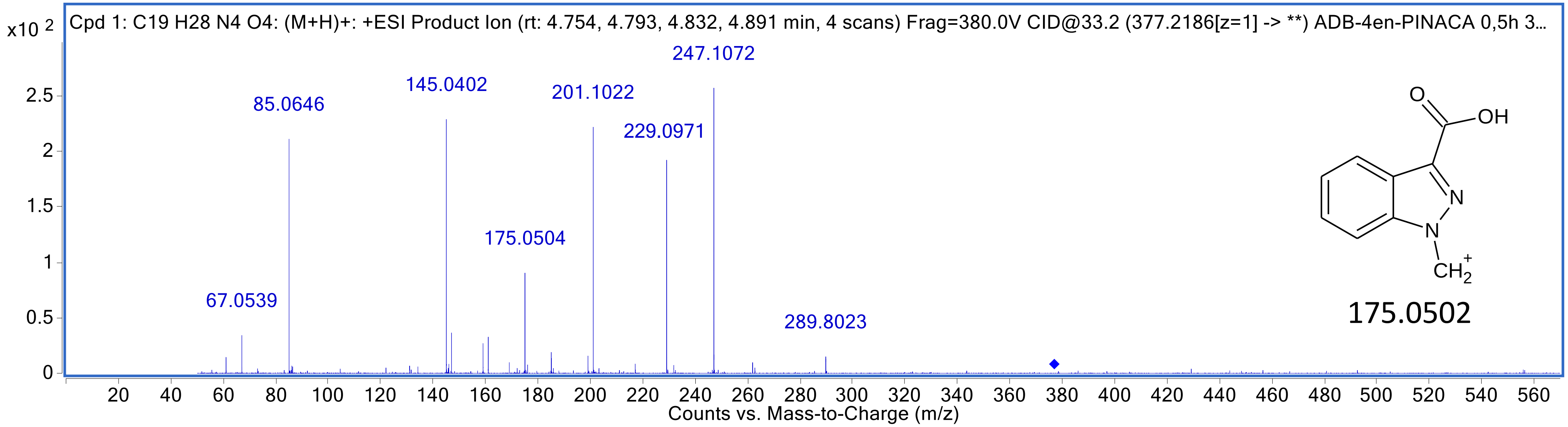

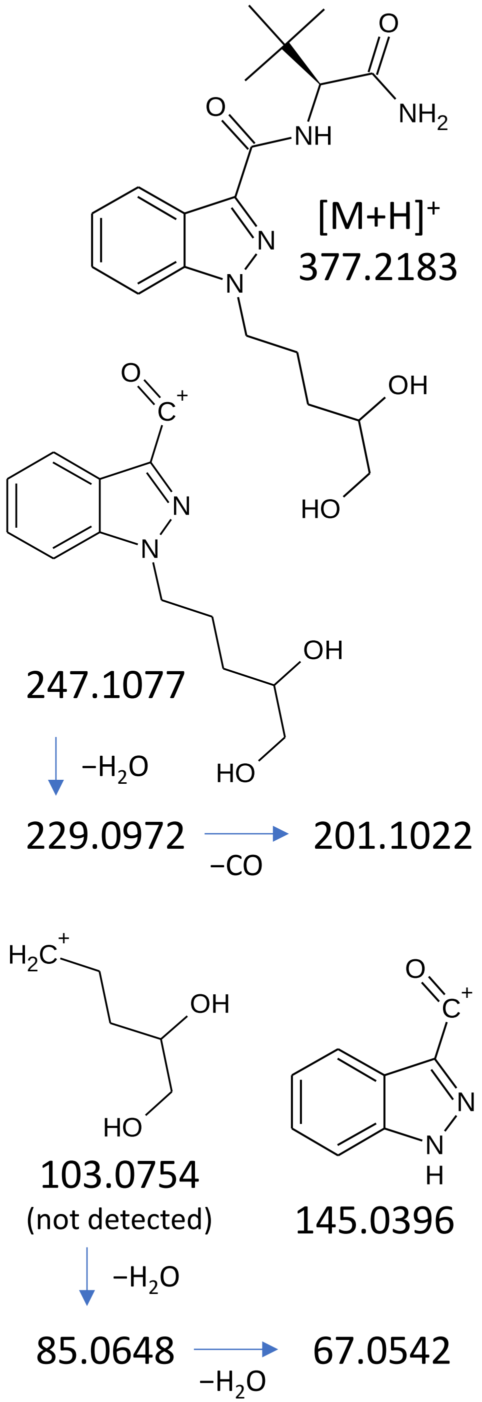

Dihydrodiol reference standard, RT 4.78 min, *m/z* 377.2185

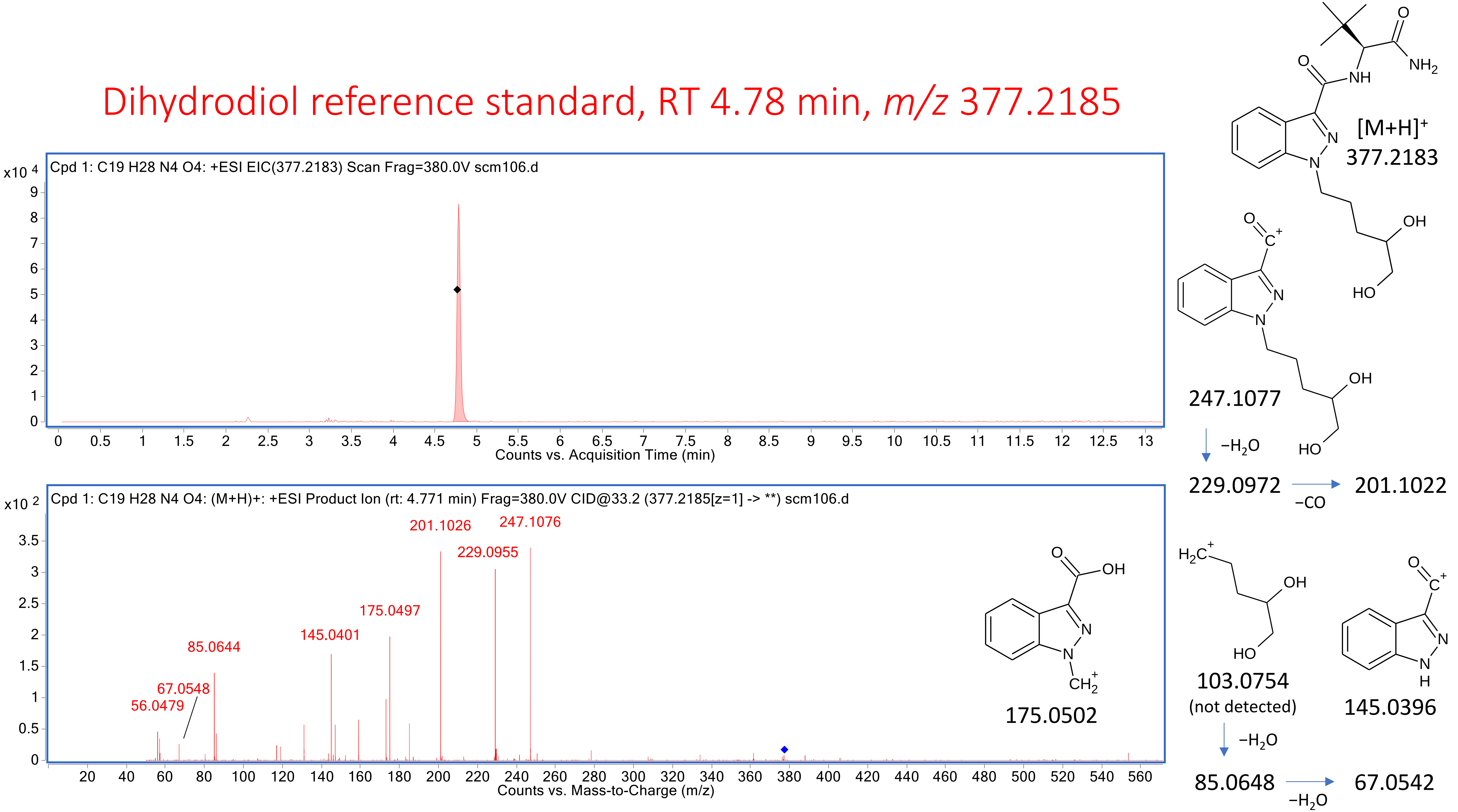

J3, Terminal amide hydrolysis, RT 10.58 min, *m/z* 344.1966

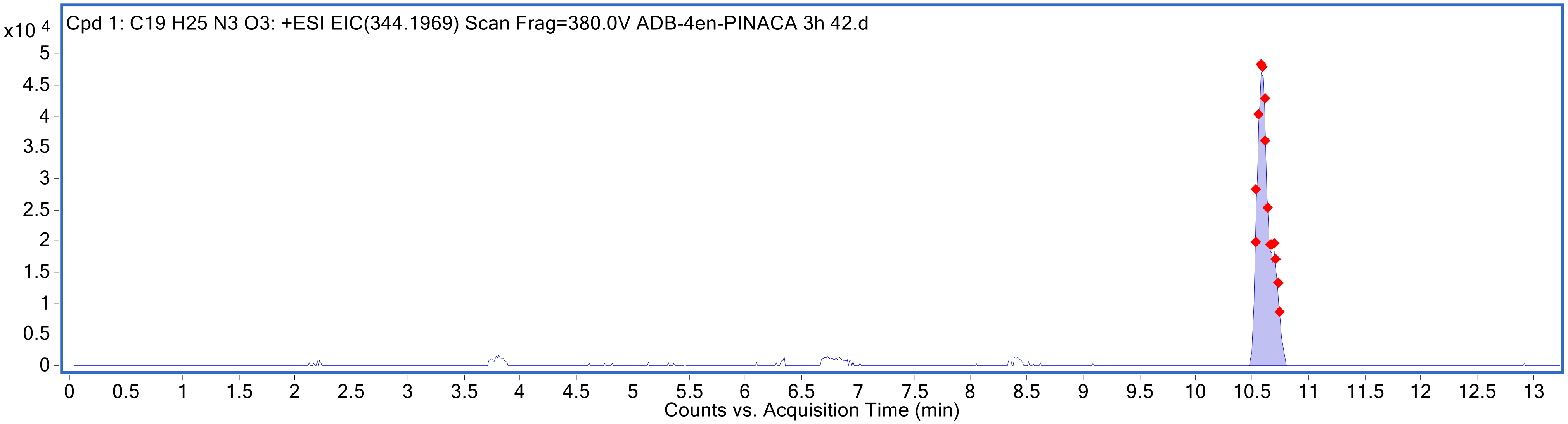

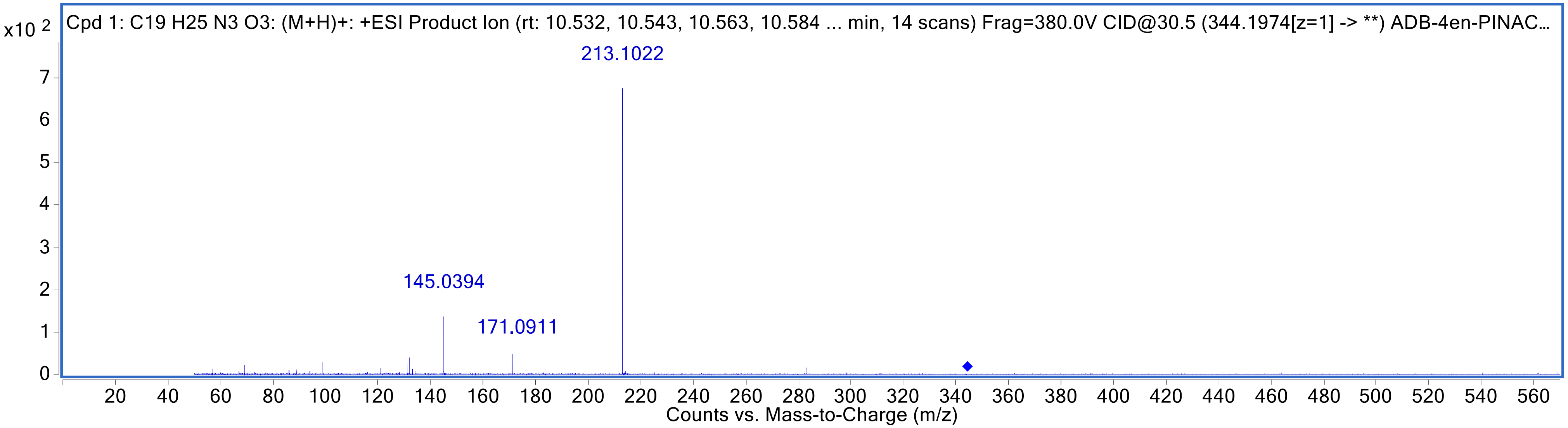

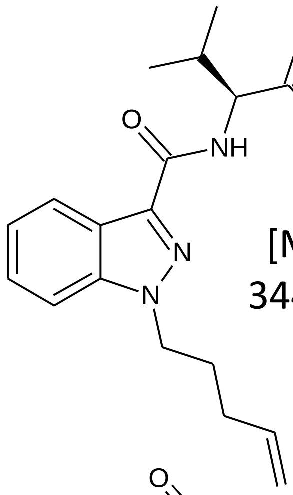

$[M+H]^+$
344.1969

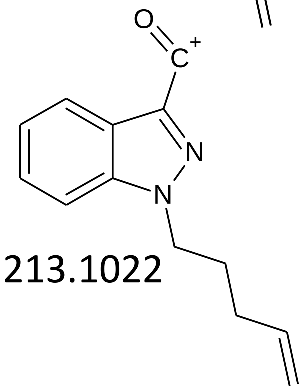

213.1022

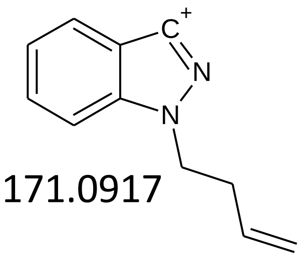

171.0917

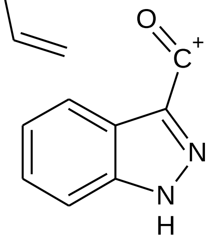

145.0396

J4, Mono-hydroxylation (indazole core), RT 7.30 min, *m/z* 359.2079

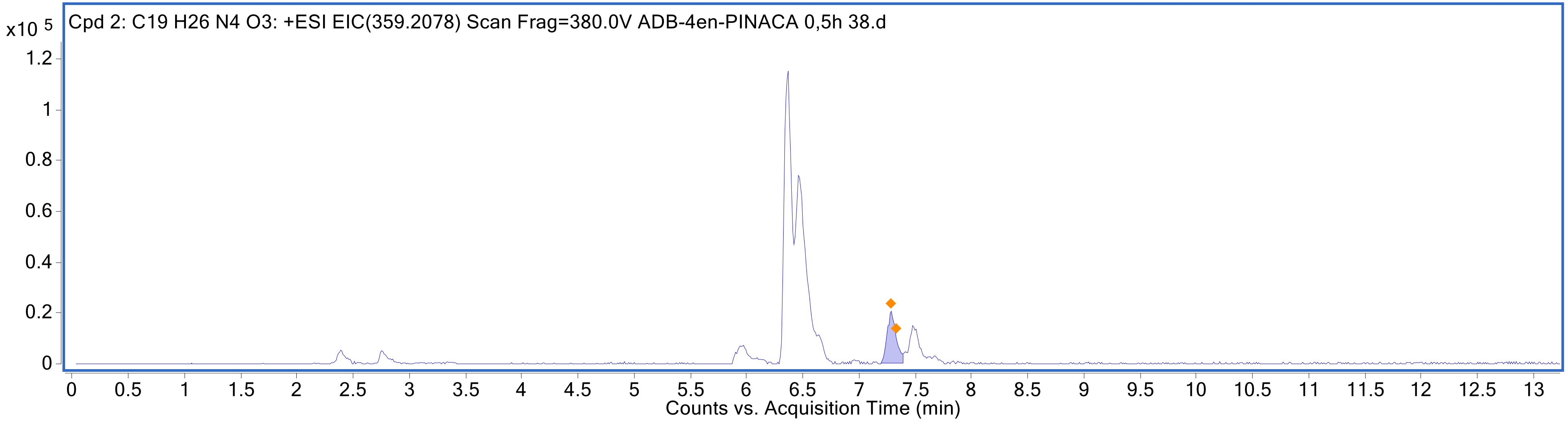

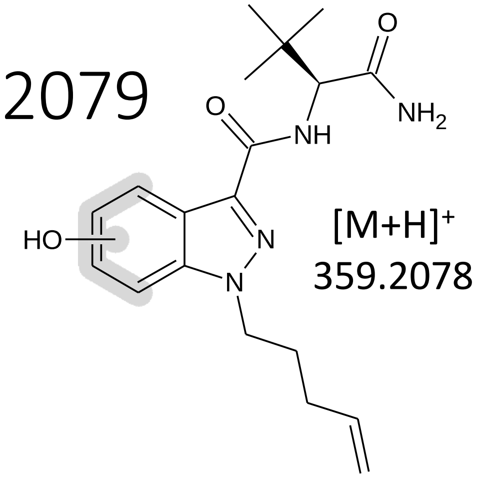

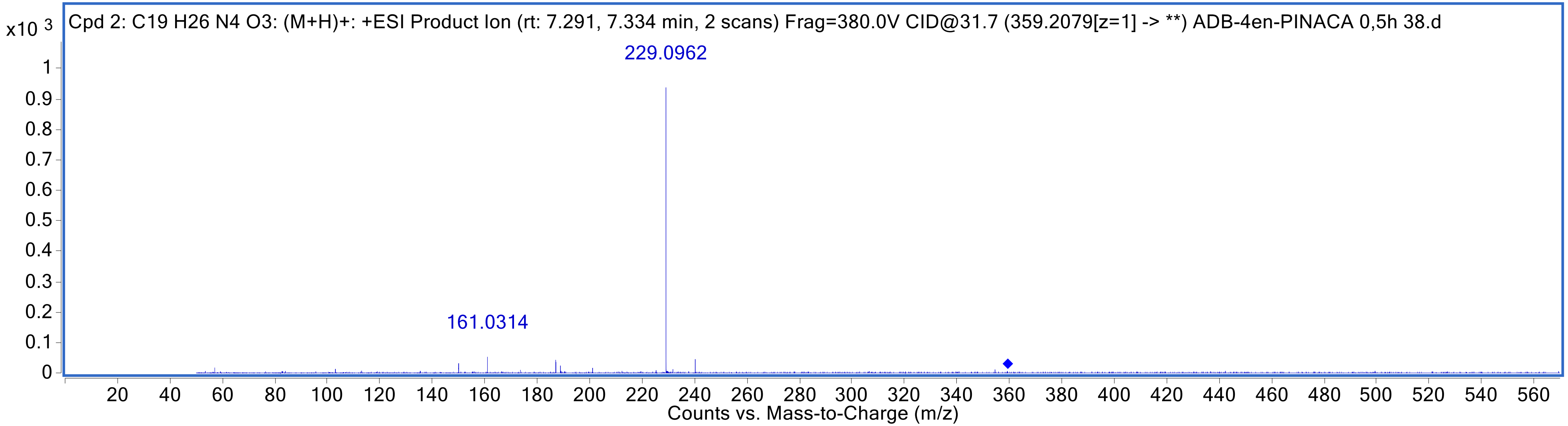

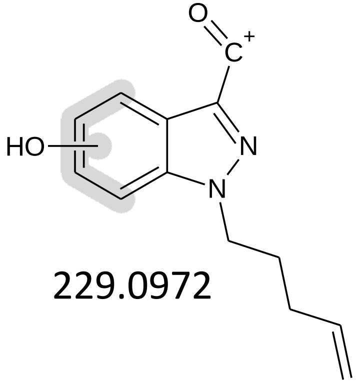

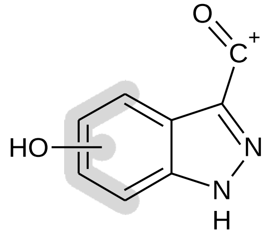

J5, Mono-hydroxylation (*tert*-butyl), RT 7.52 min, *m/z* 359.2077

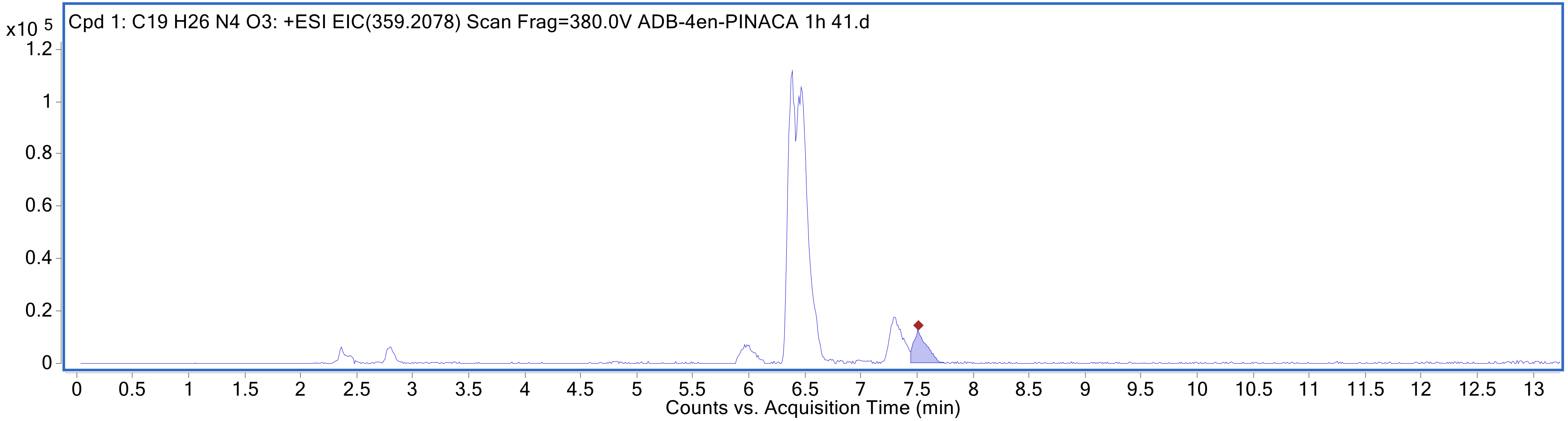

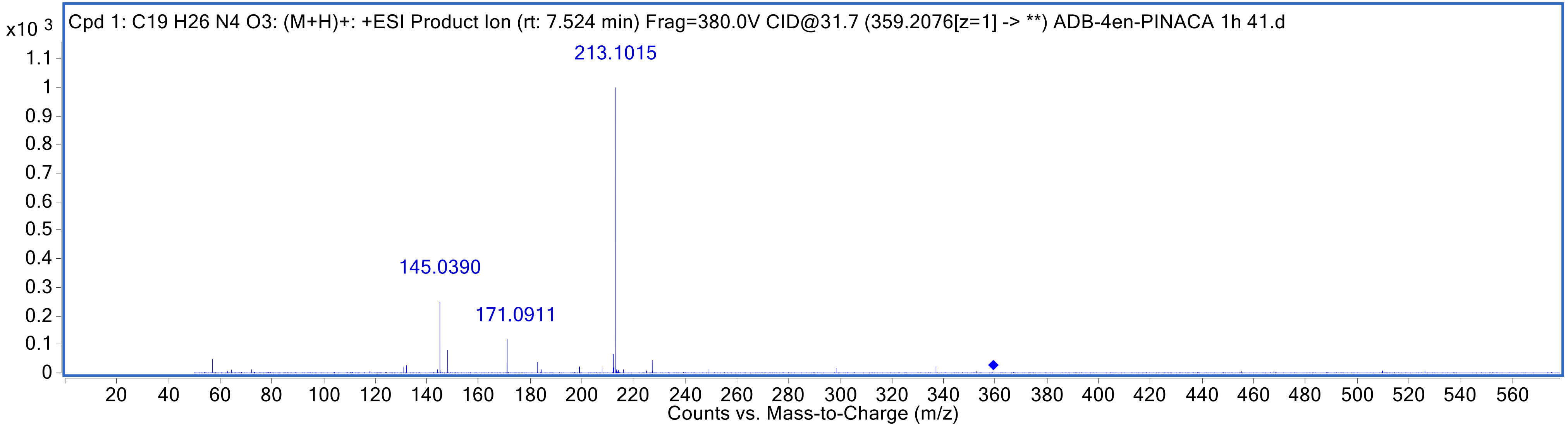

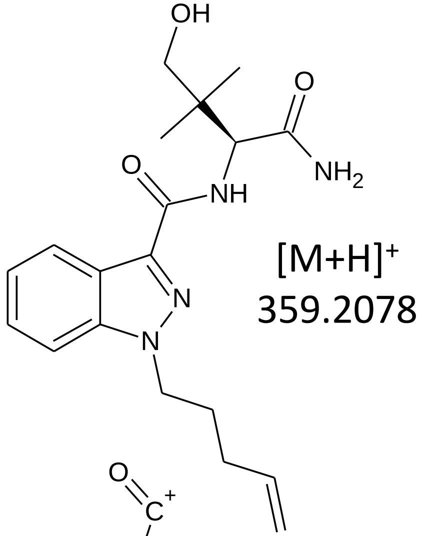

[M+H]+
359.2078

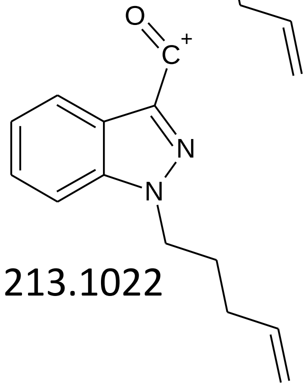

213.1022

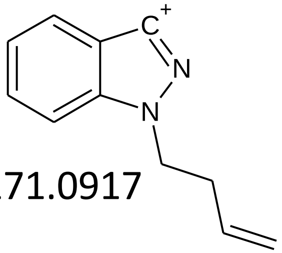

171.0917

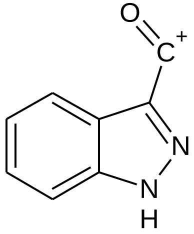

145.0396

J6, Dihydrodiol formation + glucuronidation, RT 4.05 min, *m/z* 553.2500

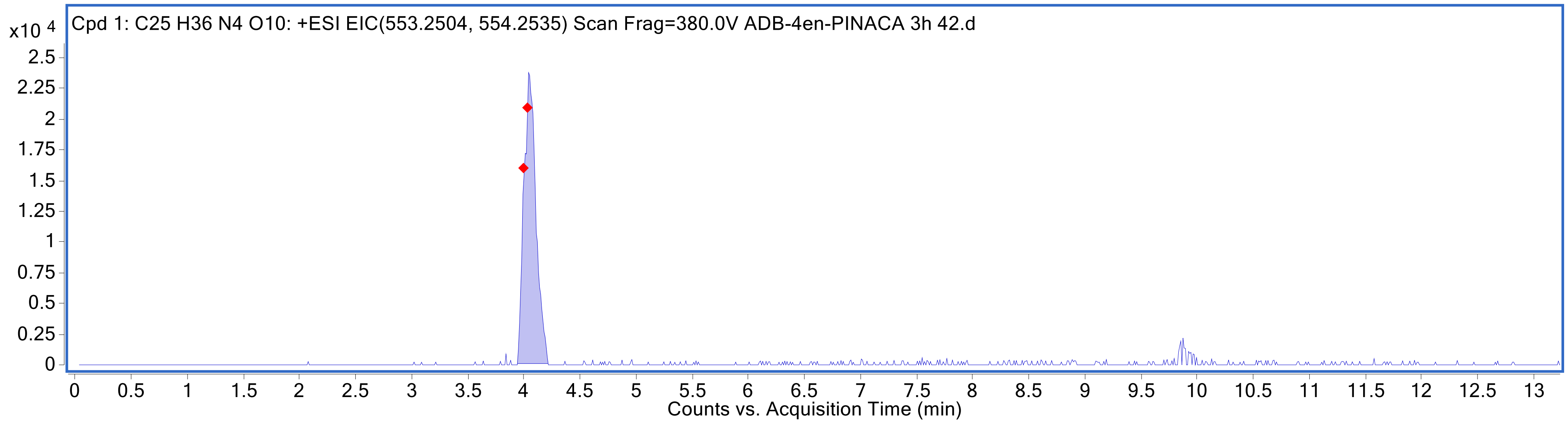

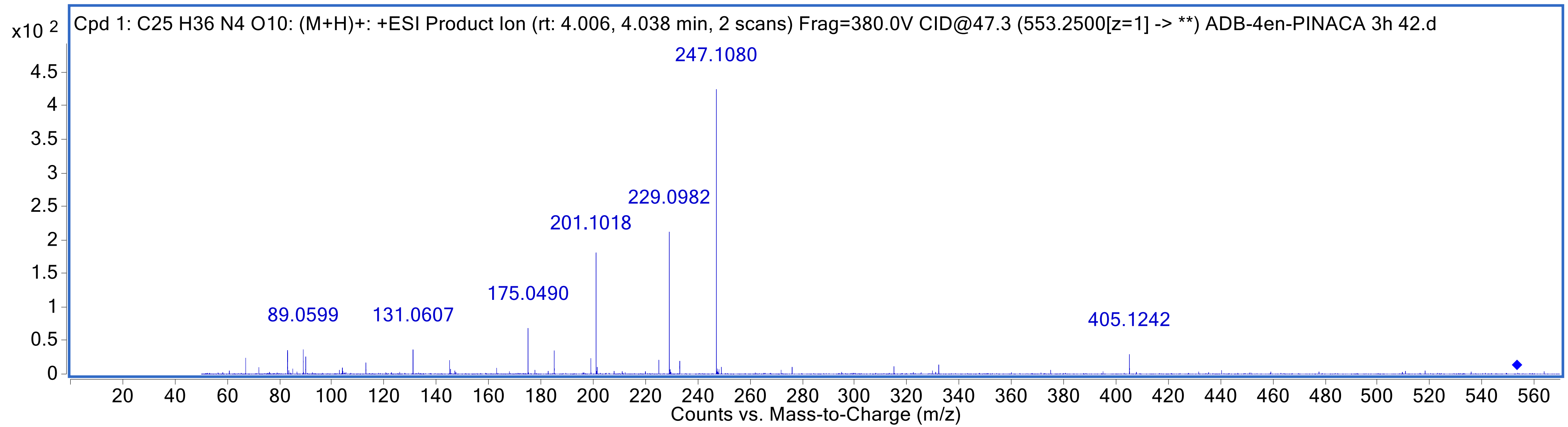

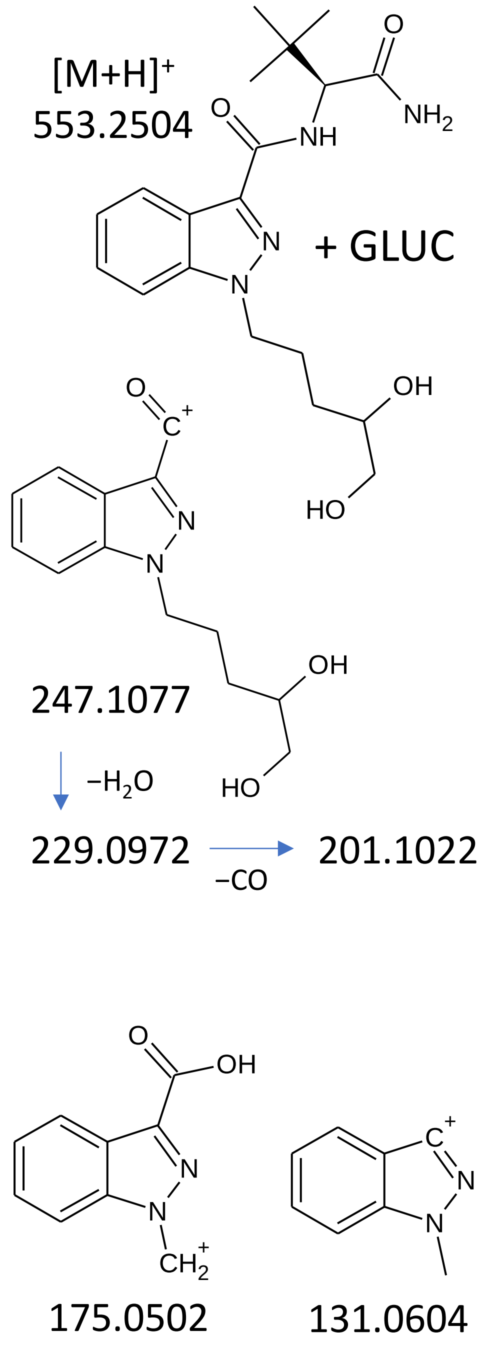

J7, Di-hydroxylation (pentenyl tail), RT 5.27 min, *m/z* 375.2021

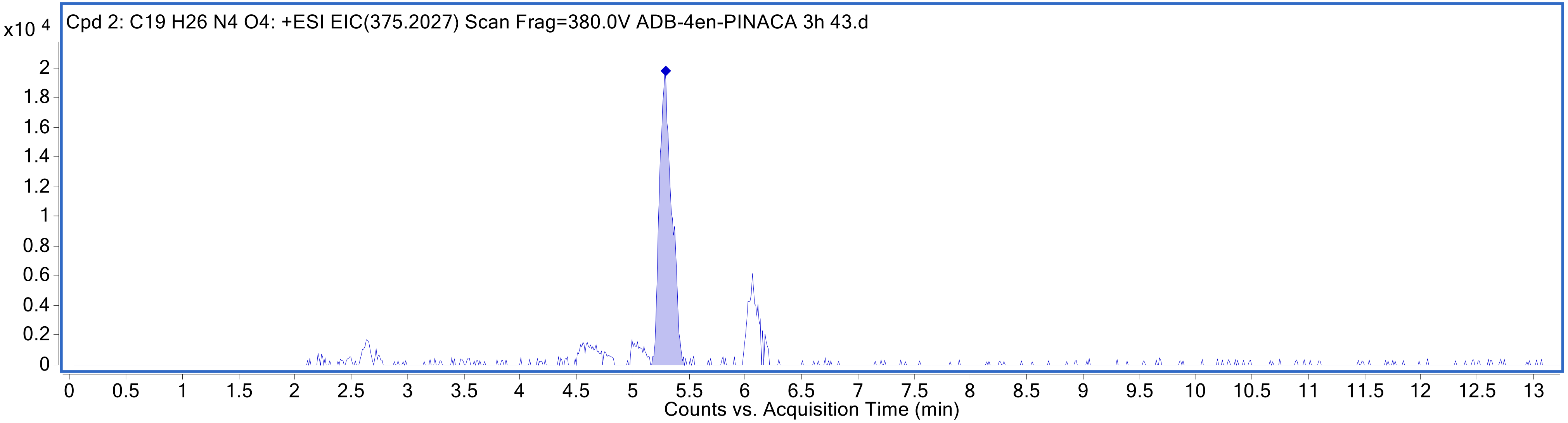

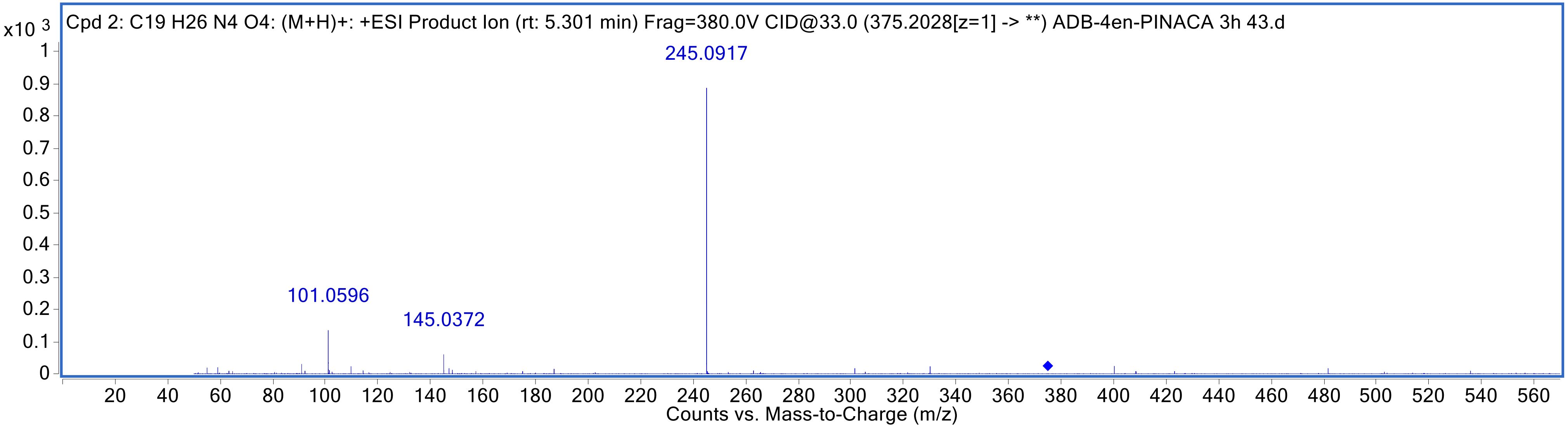

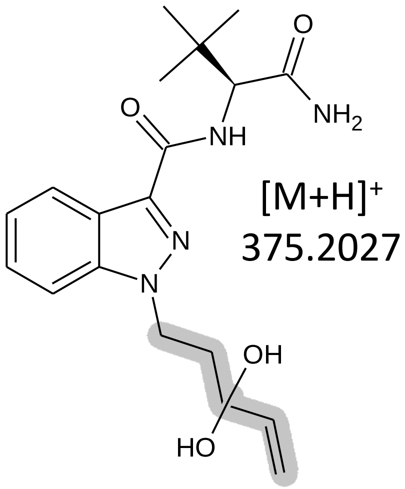

$[M+H]^+$ 375.2027

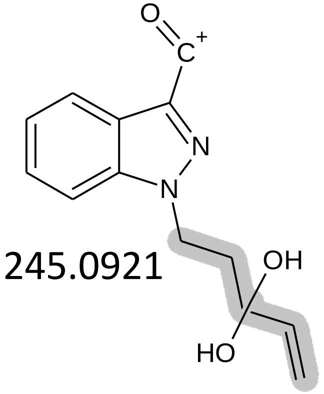

245.0921

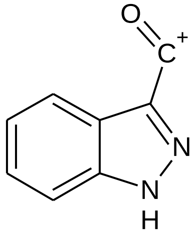

145.0396

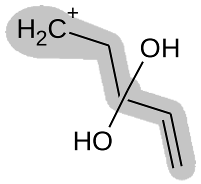

101.0597

J8, Ketone formation + mono-hydroxylation (pentenyl tail), RT 5.71 min, *m/z* 373.1863

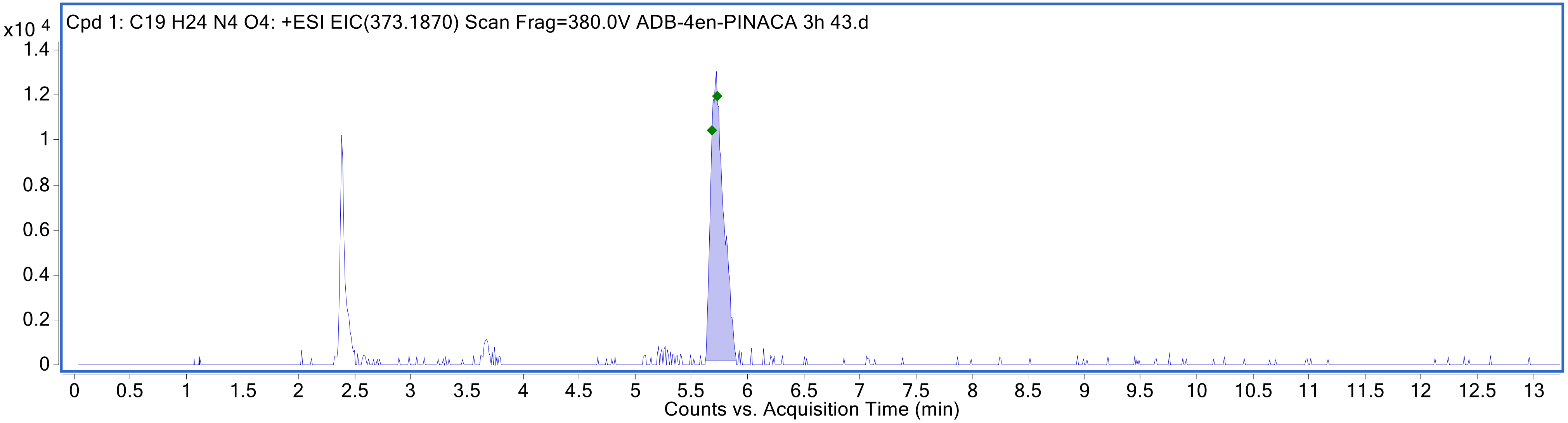

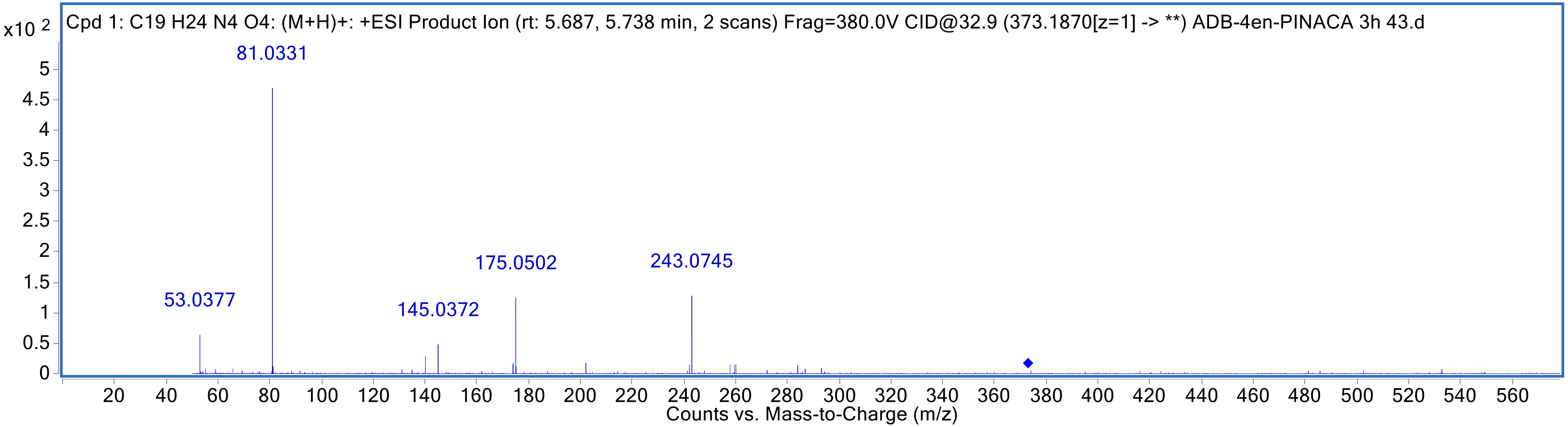

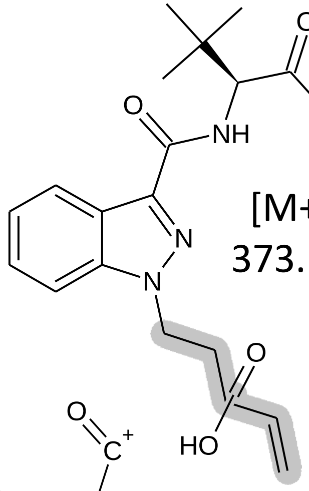

$[M+H]^+$
373.1870

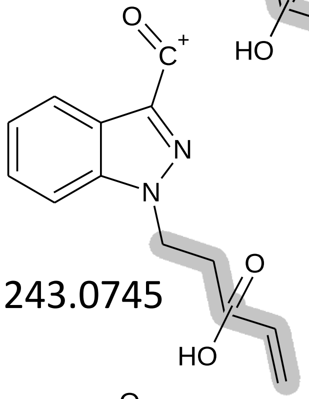

243.0745

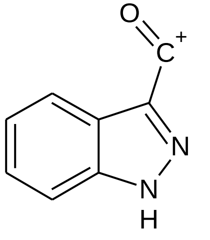

145.0396

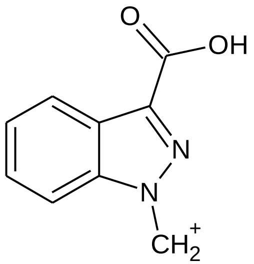

175.0502

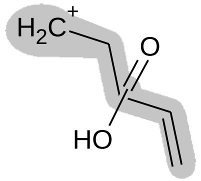

99.0441
(not detected)

→ $-H_2O$ 81.0335

J9, Dehydrogenation (pentenyl tail), RT 8.97 min, *m/z* 341.1973

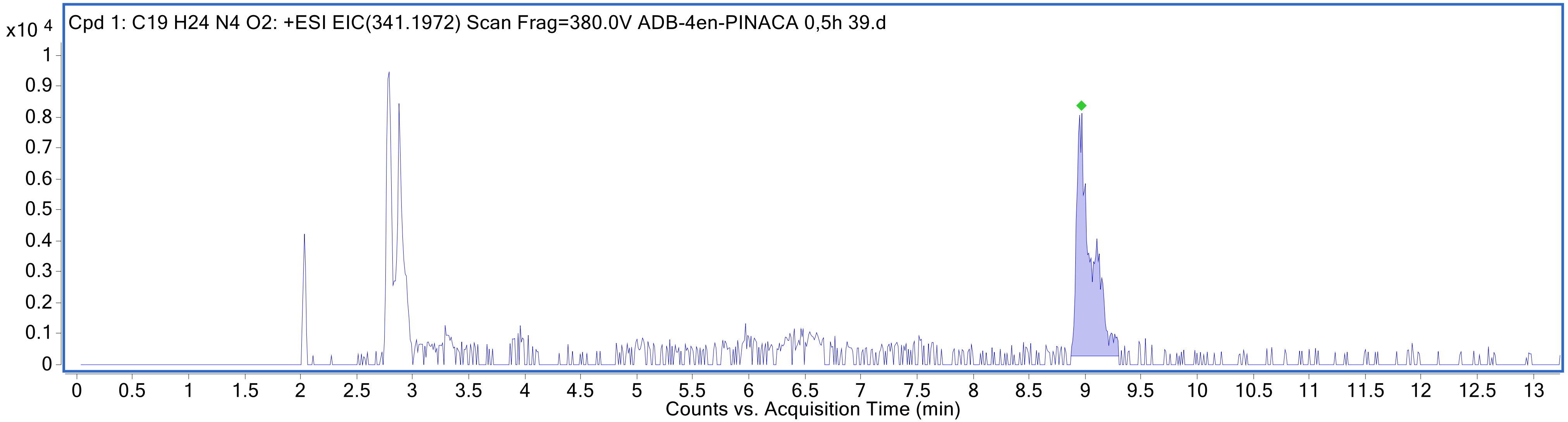

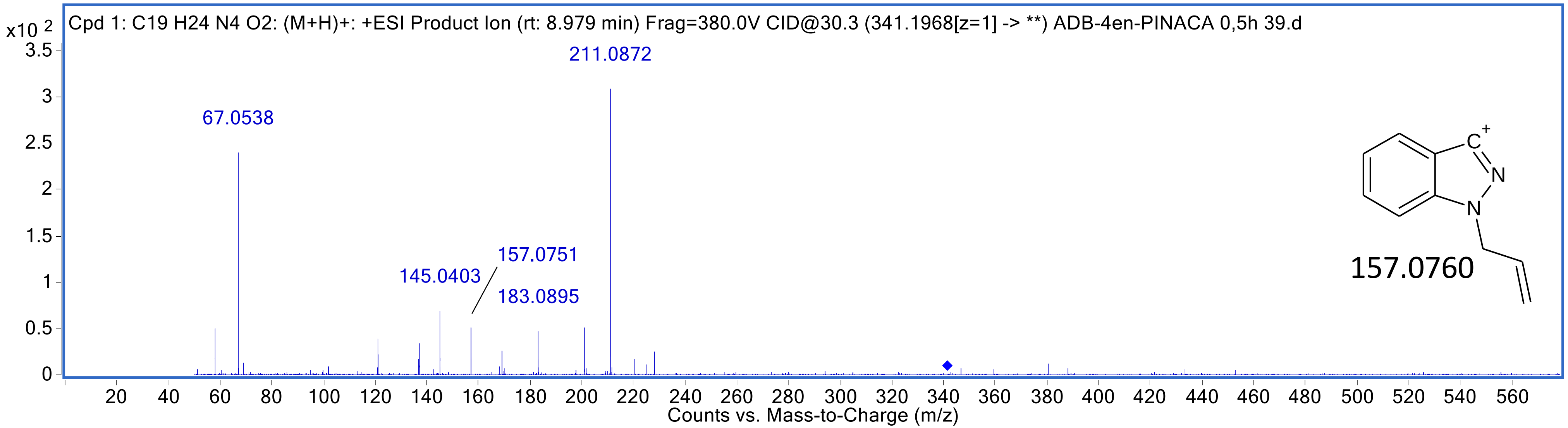

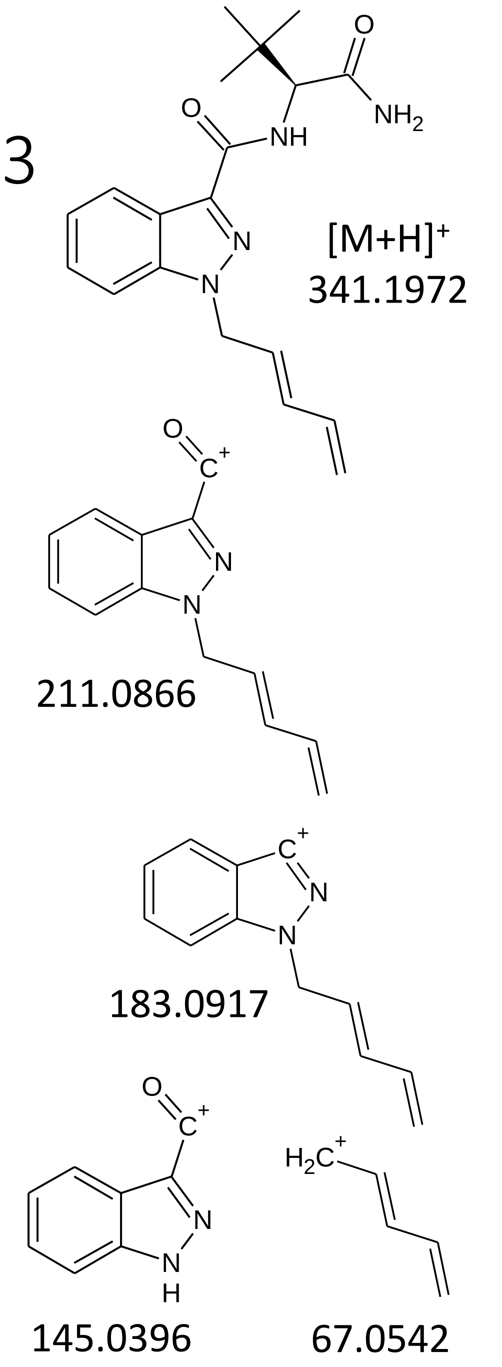

J10, Dihydrodiol formation + mono-hydroxylation (*tert*-butyl), RT 3.42 min, *m/z* 393.2126

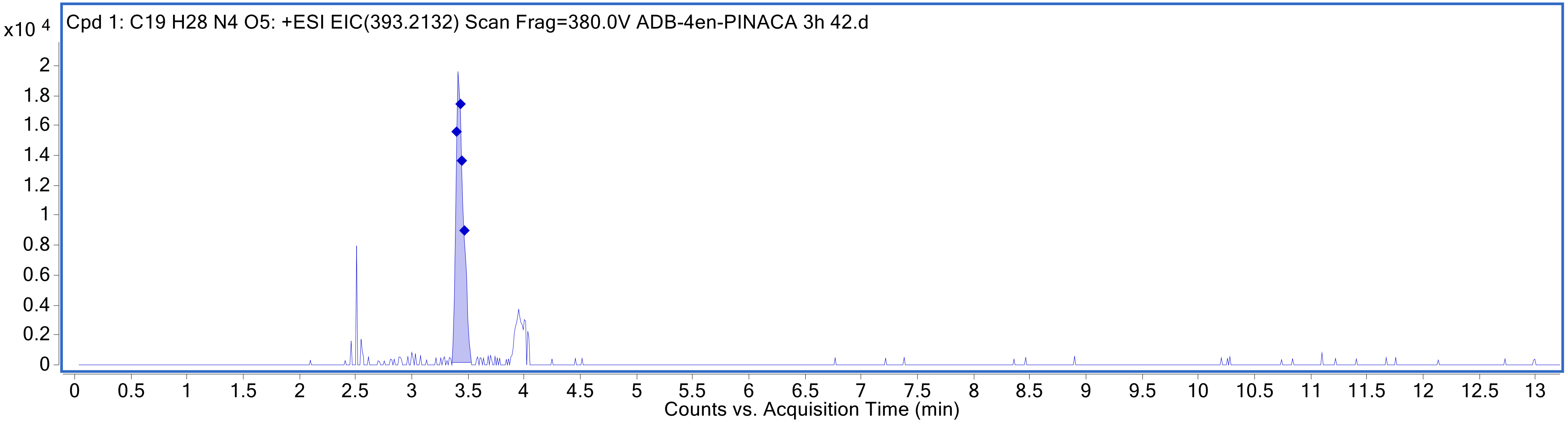

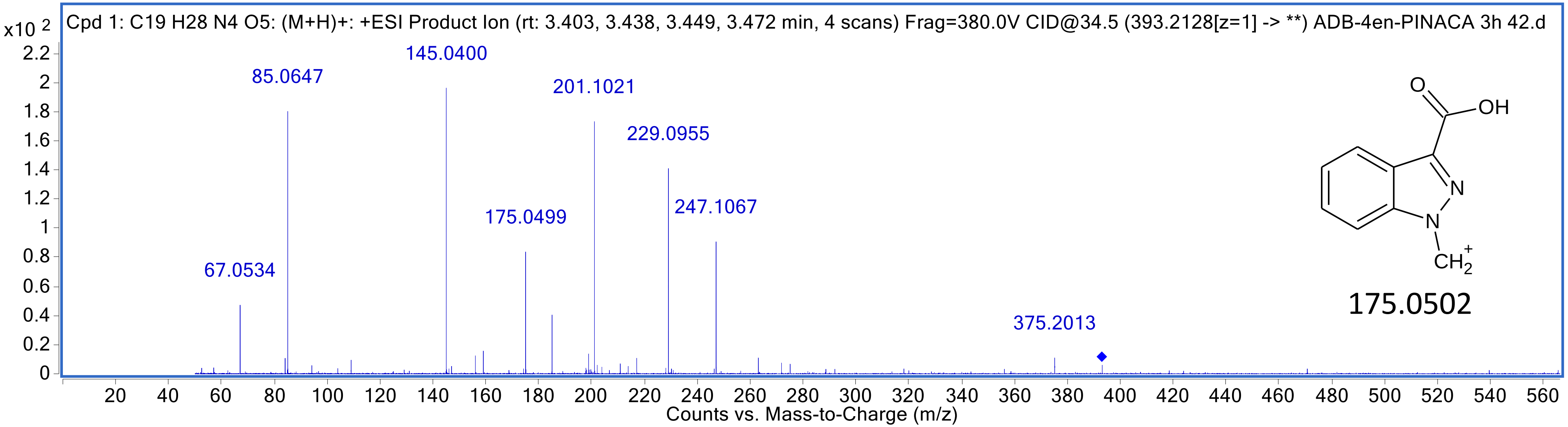

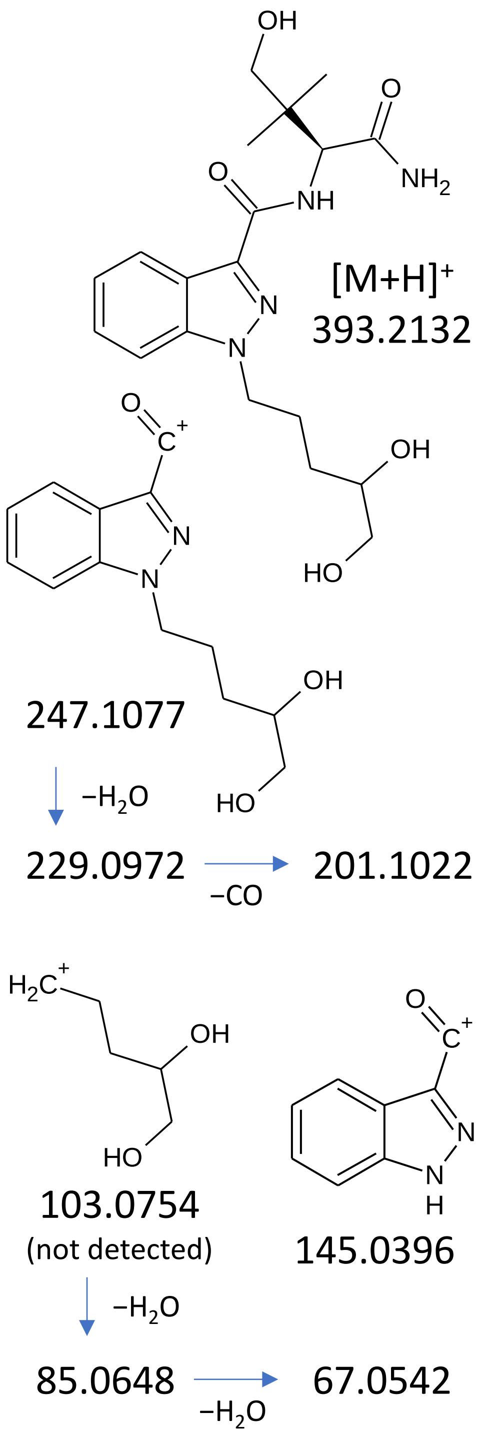

J11, Terminal amide hydrolysis + dihydrodiol formation, RT 5.60 min, *m/z* 378.2014

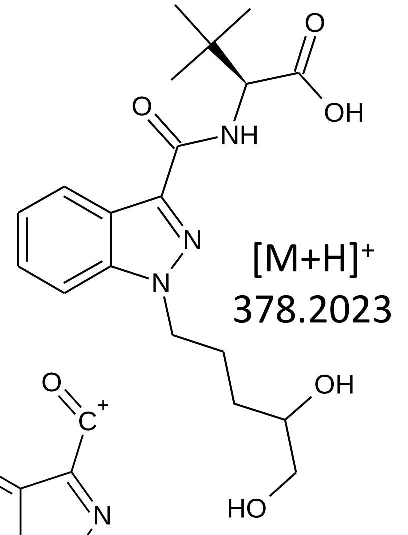

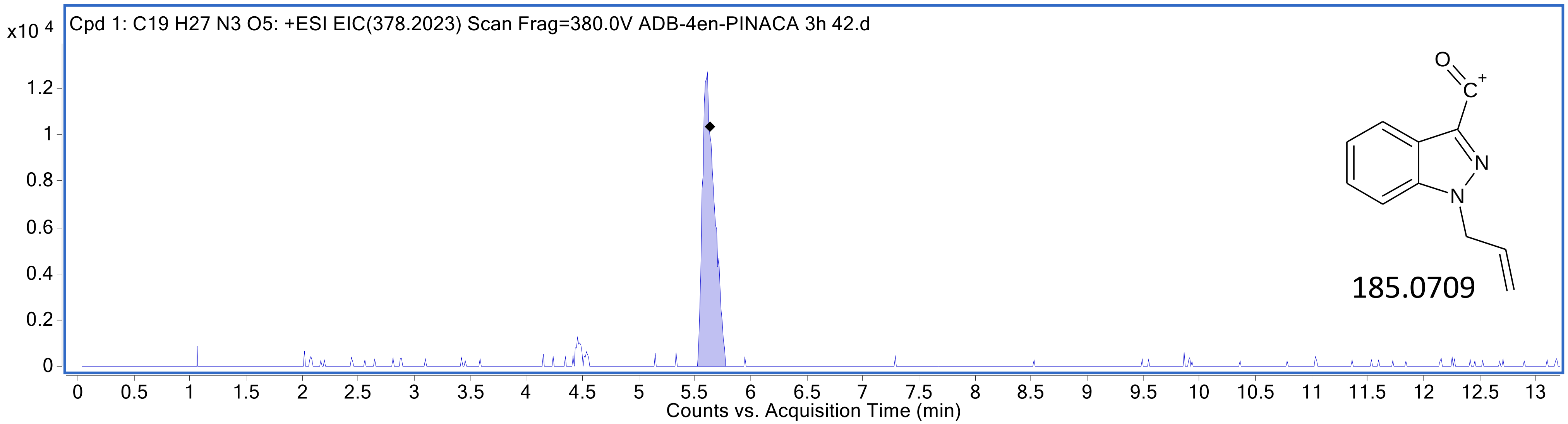

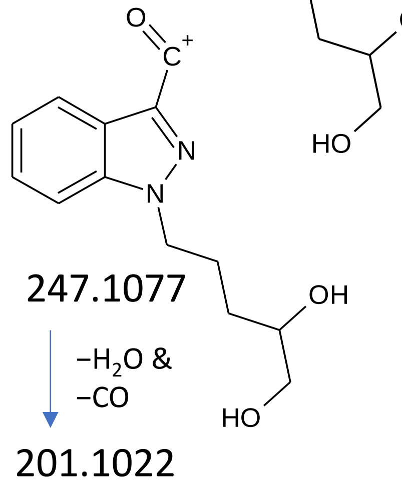

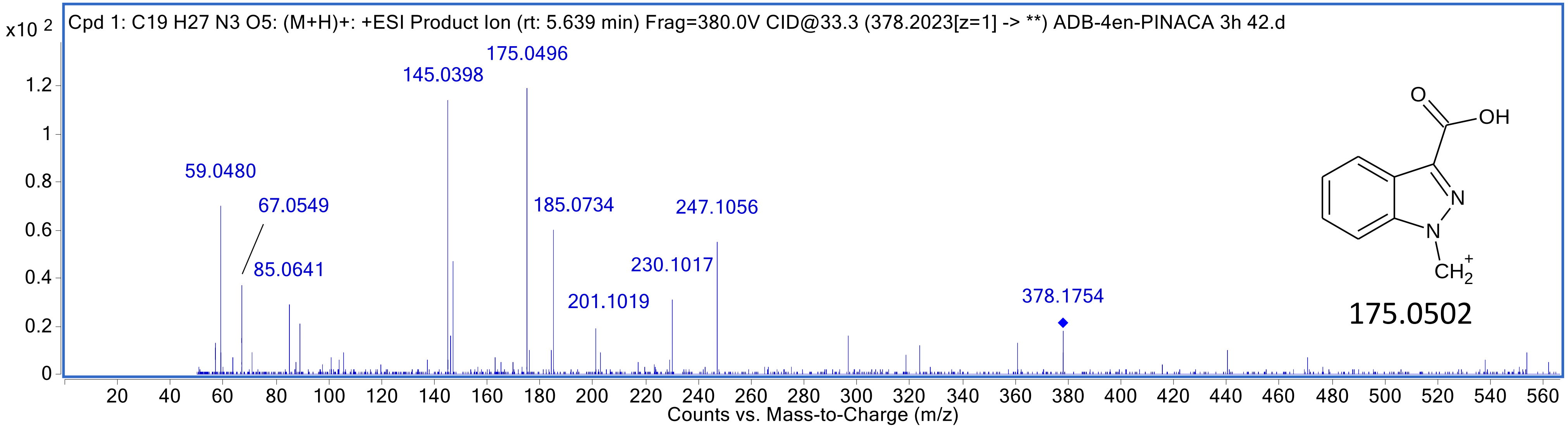

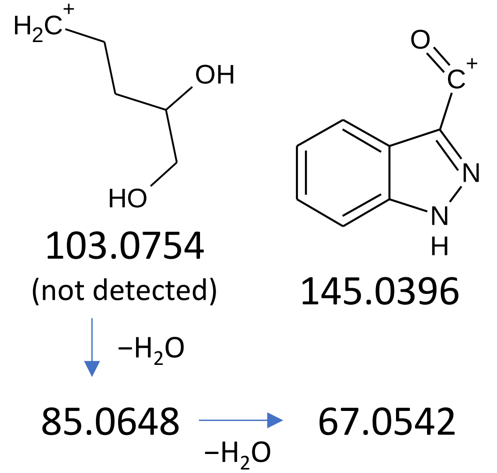

BZO-4en-POXIZID

Metabolism

BZO-4en-POXIZID, RT 10.69 min, *m/z* 334.1563

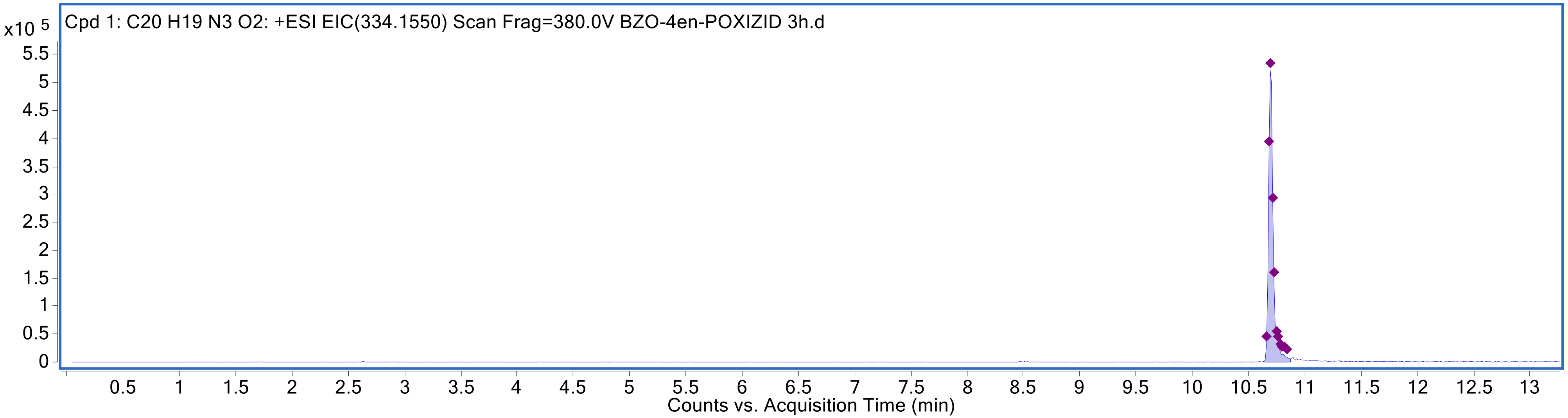

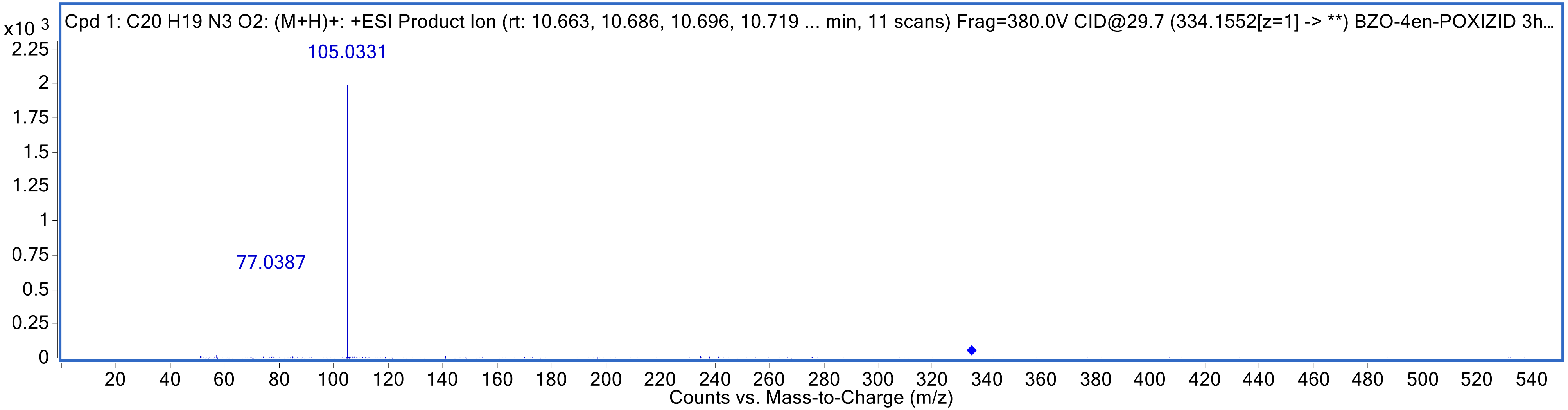

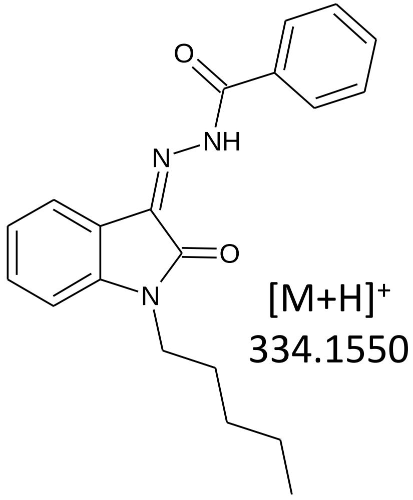

$[M+H]^+$
334.1550

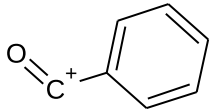

105.0335

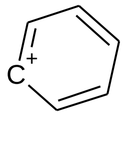

77.0386

K1, Dihydrodiol formation, RT 5.76 min, *m/z* 368.1606

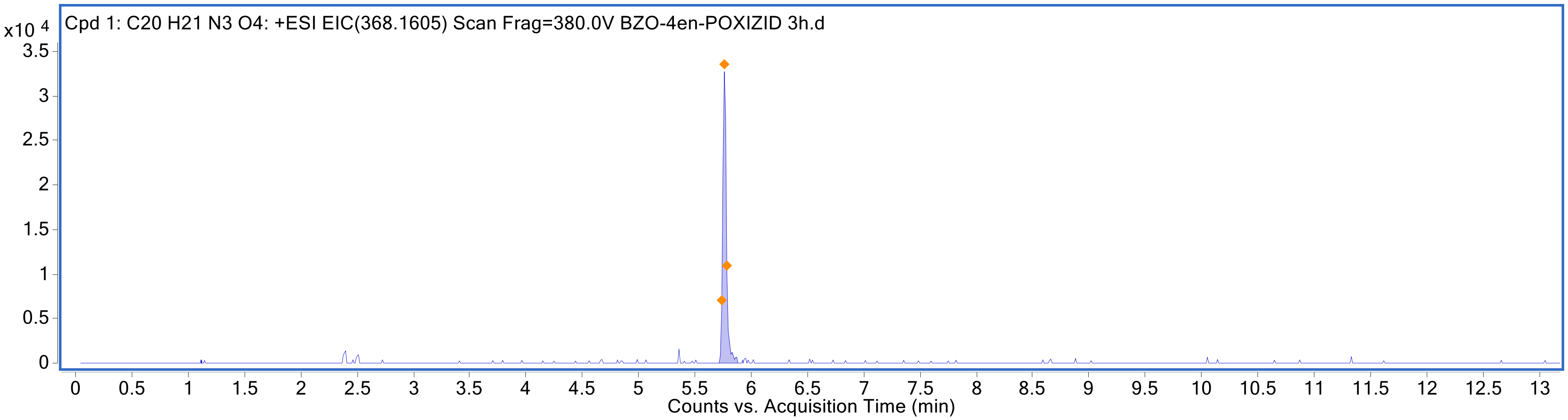

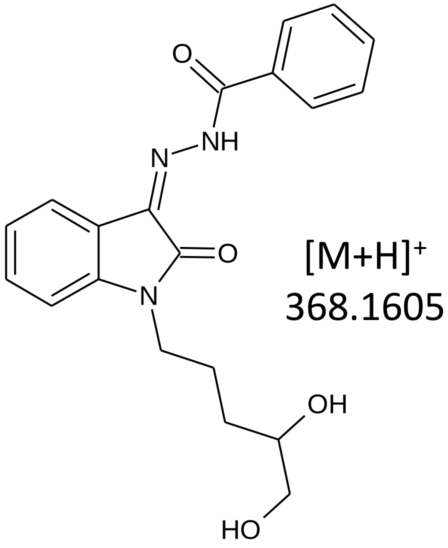

[M+H]+
368.1605

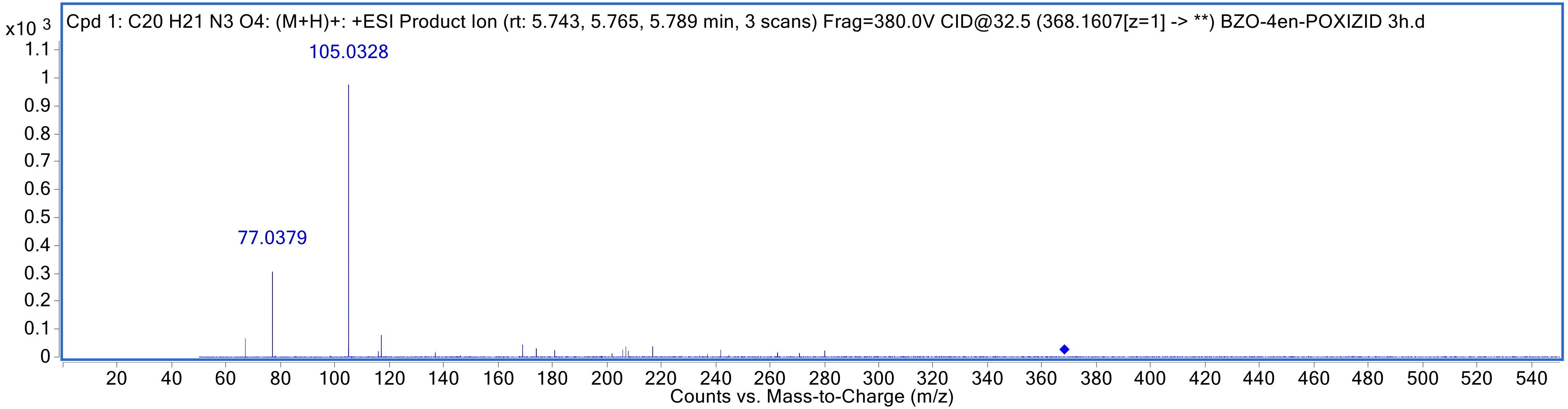

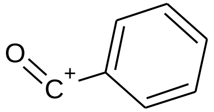

105.0335

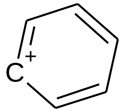

77.0386

K2, Mono-hydroxylation (pentenyl tail) + glucuronidation, RT 6.89 min, *m/z* 526.1818

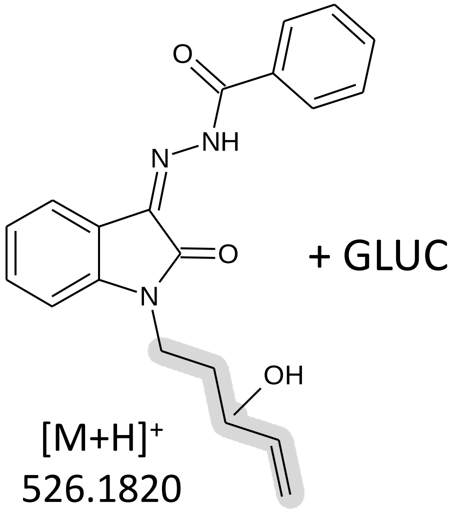

[M+H]+
526.1820

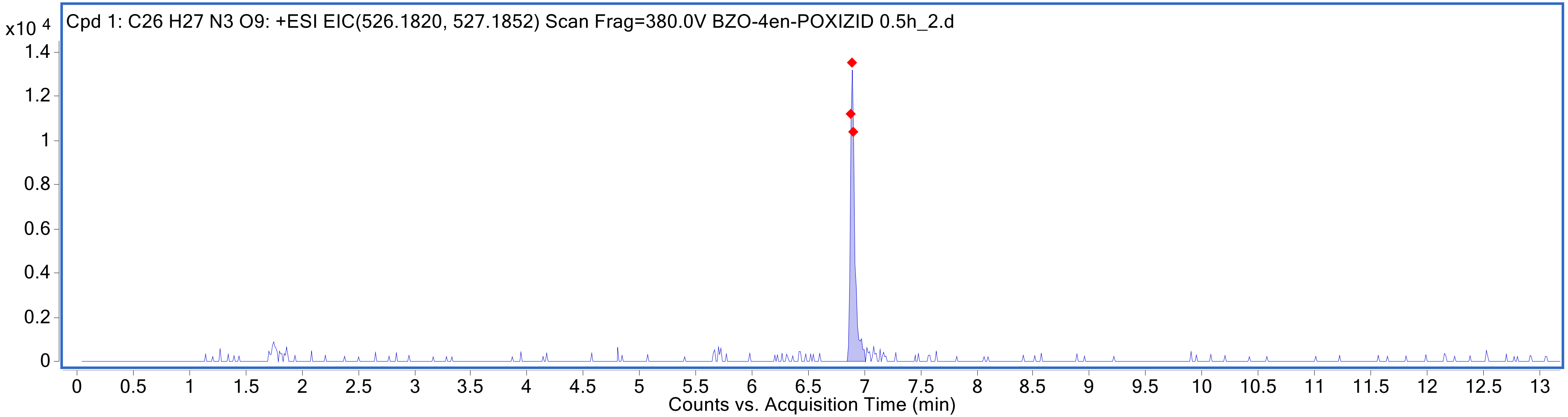

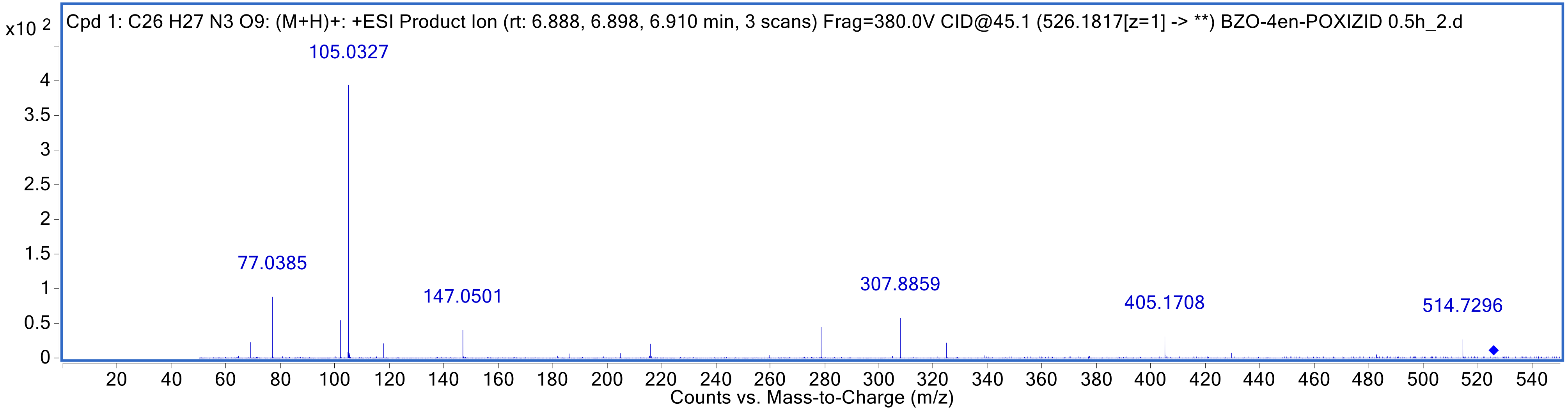

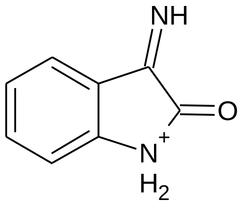

147.0553

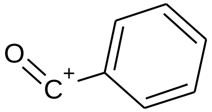

105.0335

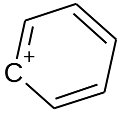

77.0386

K3, Mono-hydroxylation (pentenyl tail), RT 7.73 min, *m/z* 350.1498

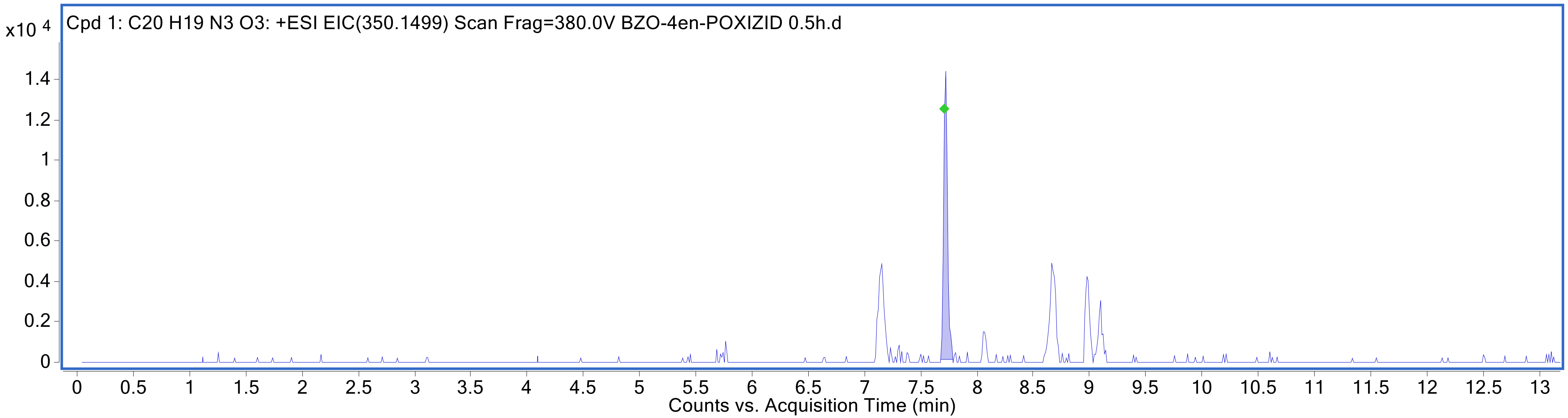

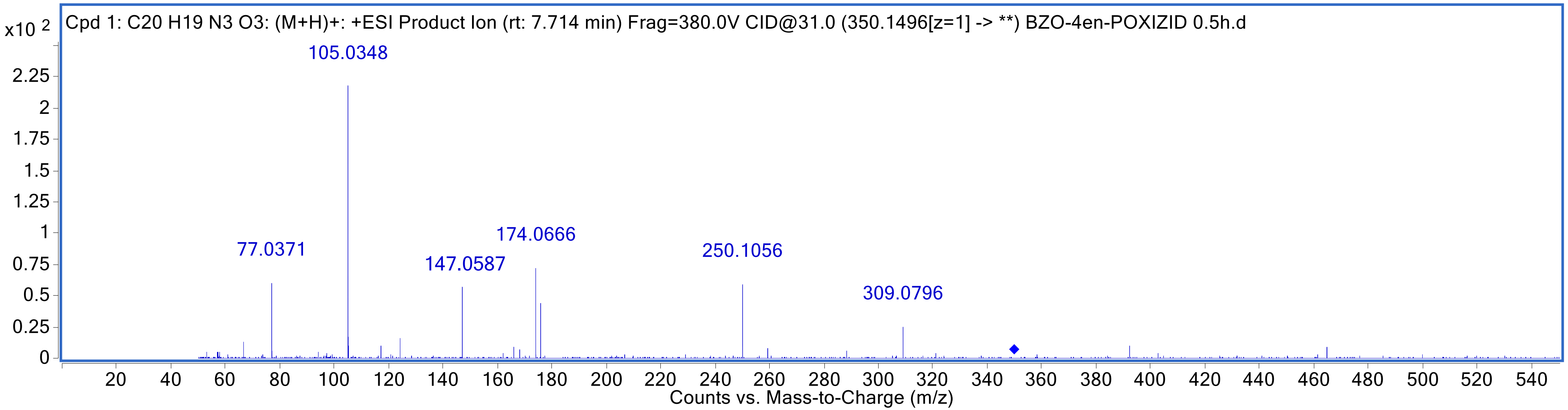

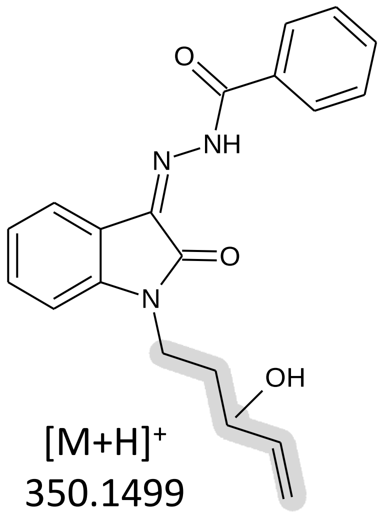

$[M+H]^+$
350.1499

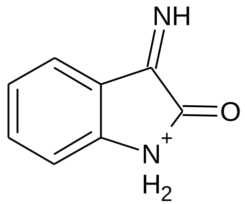

147.0553

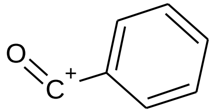

105.0335

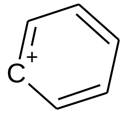

77.0386
