# Supplementary material for: Structure–metabolism relationships of 4-pentenyl synthetic cannabinoid receptor agonists using in vitro human hepatocyte incubations and high-resolution mass spectrometry
Source: Arch Toxicol. 2025 May 20;99(8):3331–41. doi: 10.1007/s00204-025-04080-6 (PMC12367891; doi:10.1007/s00204-025-04080-6)
Supplement: Supplementary file 2 — Supplementary file2 (PDF 1333 KB) [file 204_2025_4080_MOESM2_ESM.pdf]

Structure–metabolism relationships of 4-pentenyl synthetic cannabinoid receptor agonists using *in vitro* human hepatocyte incubations and high-resolution mass spectrometry

Supplementary Information for Archives of Toxicology – Metabolic Pathway Figures

Steven R Baginski[1,*], Karin Lindbom[2], Bryan Valencia Crespo[3], Ghidaa Bessa[3], Tobias Rautio[3], Xiongyu Wu[3], Johan Dahlén[3], Lorna A Nisbet[1], Craig McKenzie[1,4], Henrik Gréen[2,5,*]

[1] Leverhulme Research Centre for Forensic Science, School of Science and Engineering, University of Dundee, Dundee, UK

[2] Division of Clinical Chemistry and Pharmacology, Department of Biomedical and Clinical Sciences, Linköping University, Linköping, Sweden

[3] Department of Physics, Chemistry and Biology, Linköping University, Linköping, Sweden

[4] Chiron AS, Trondheim, Norway

[5] Department of Forensic Genetics and Forensic Toxicology, National Board of Forensic Medicine, Linköping, Sweden

*Corresponding authors:

Steven Baginski, sbaginski001@dundee.ac.uk and Henrik Gréen, henrik.green@liu.se

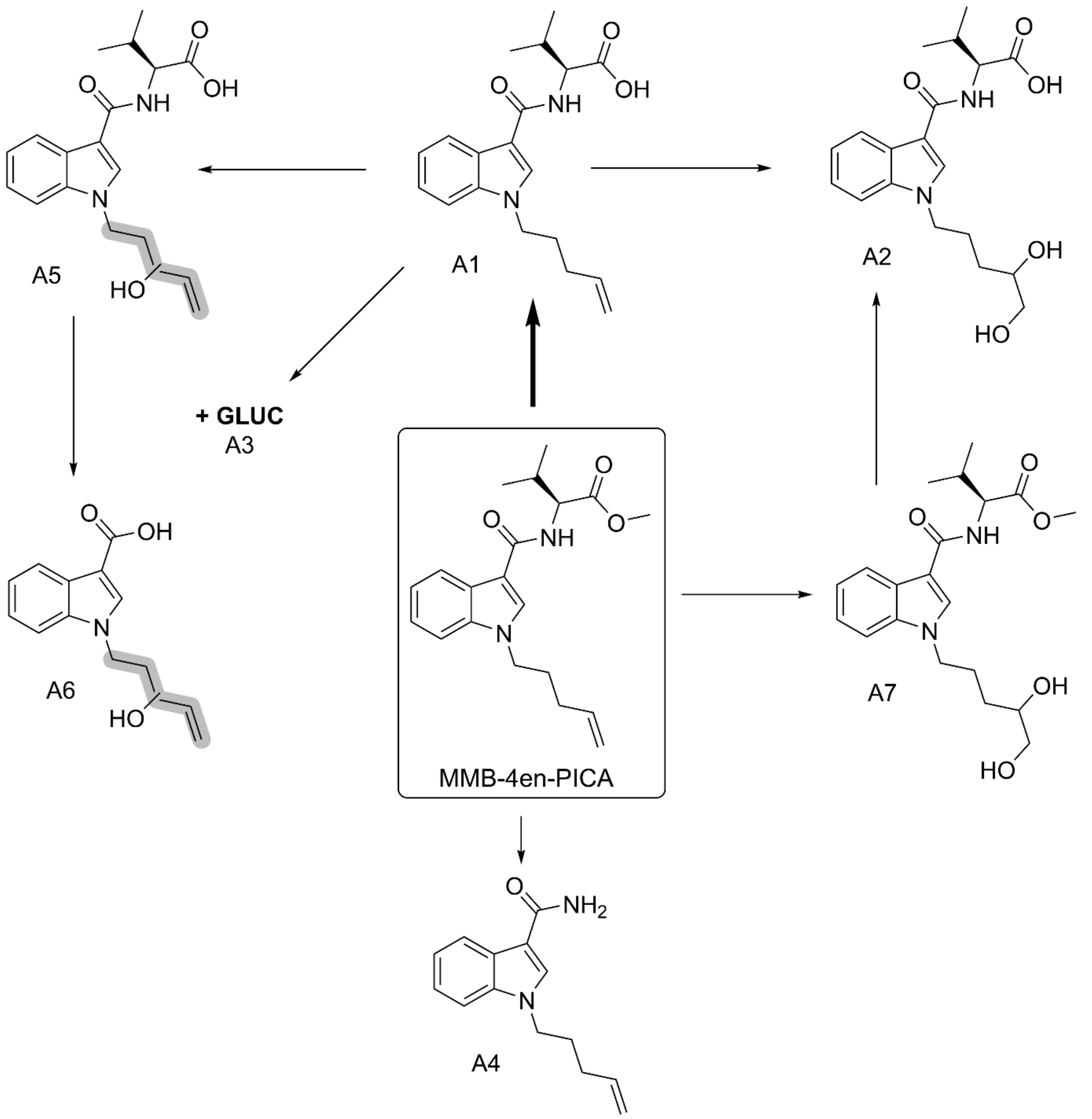

Fig. S1 Proposed metabolic pathways of MMB-4en-PICA following incubation with human hepatocytes

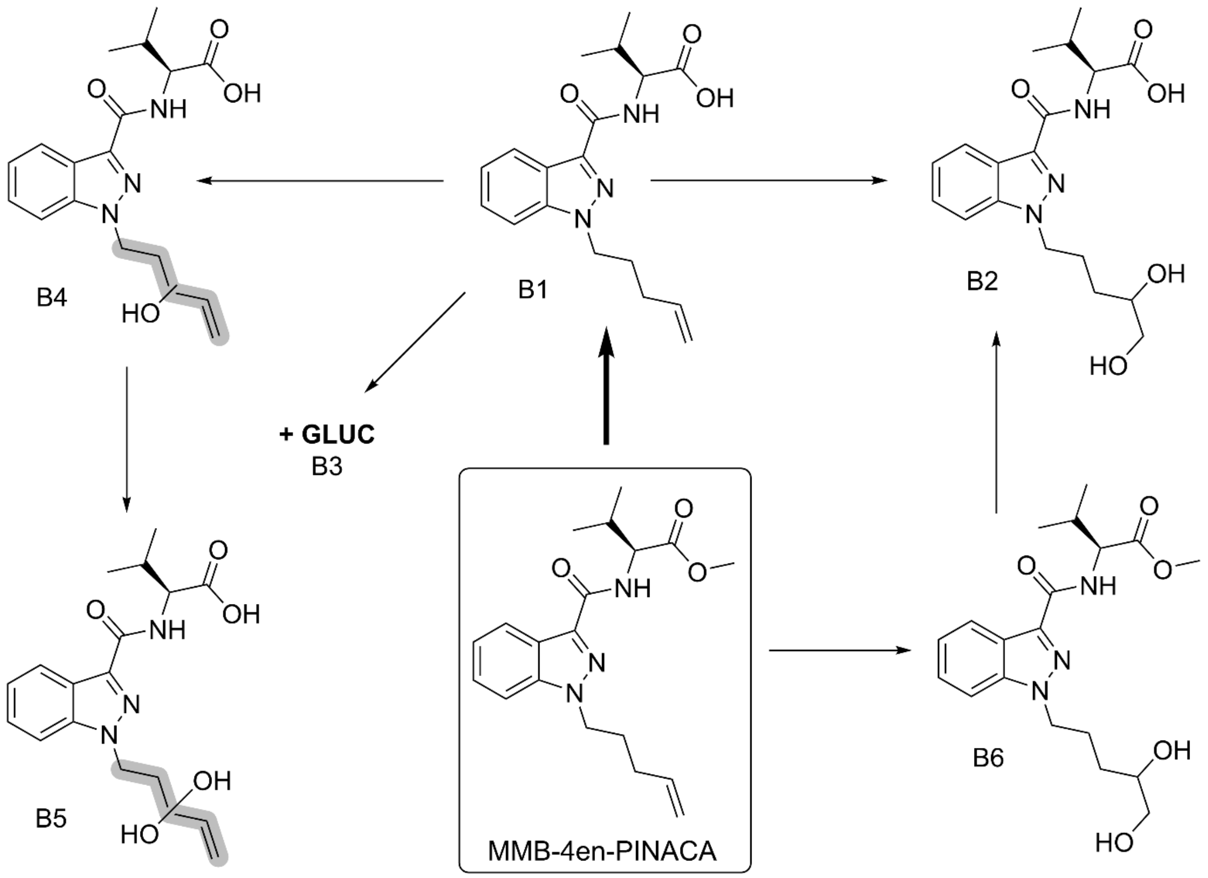

Fig. S2 Proposed metabolic pathways of MMB-4en-PINACA following incubation with human hepatocytes

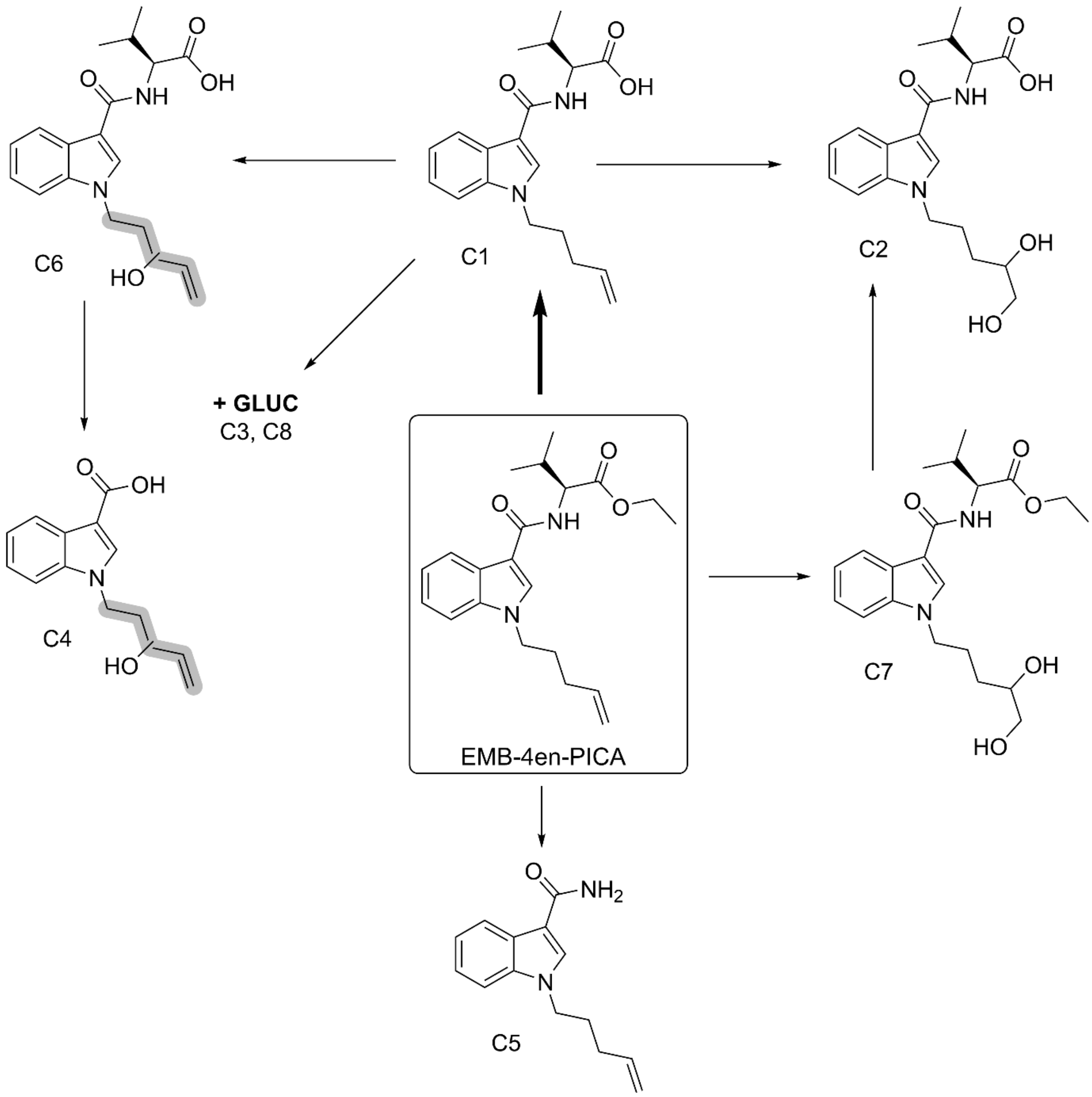

Fig. S3 Proposed metabolic pathways of EMB-4en-PICA following incubation with human hepatocytes

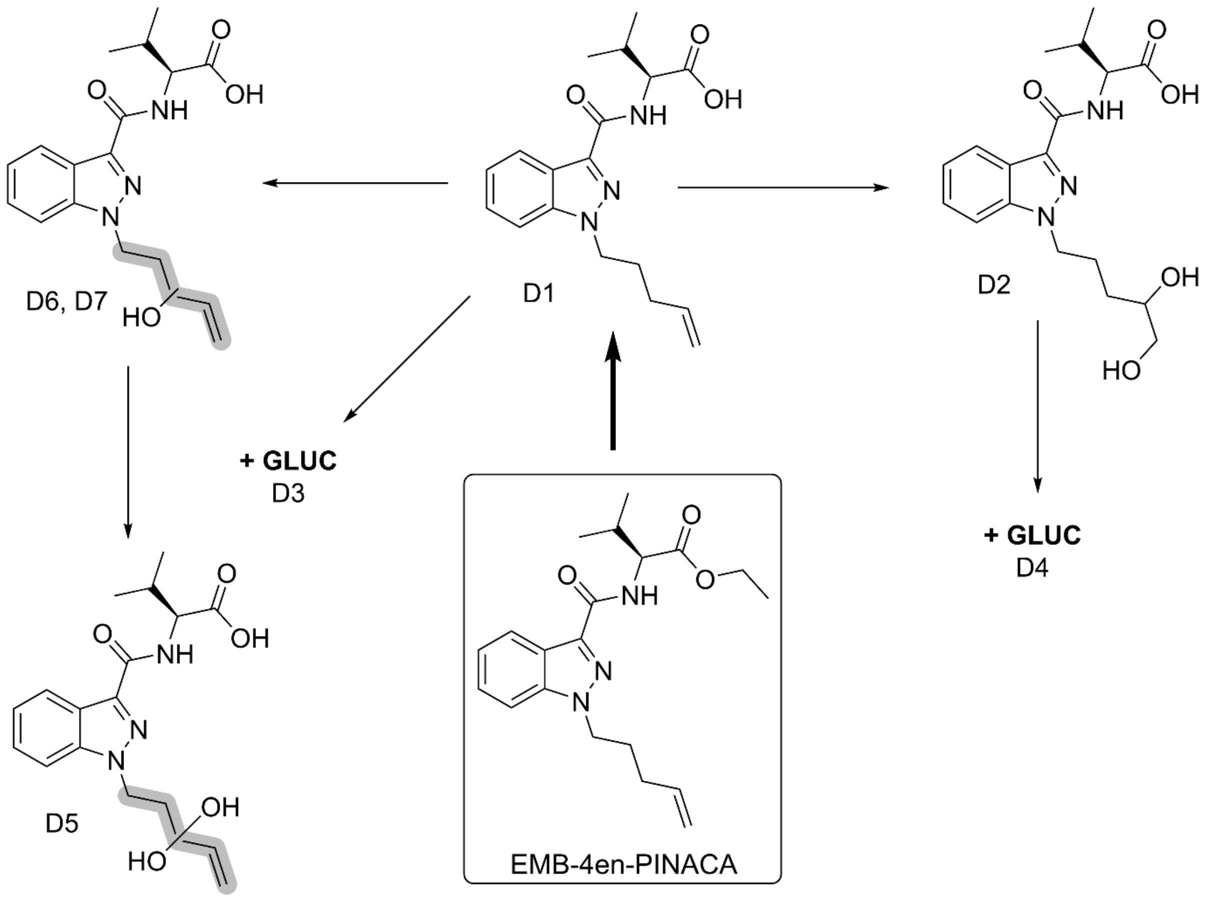

Fig. S4 Proposed metabolic pathways of EMB-4en-PINACA following incubation with human hepatocytes

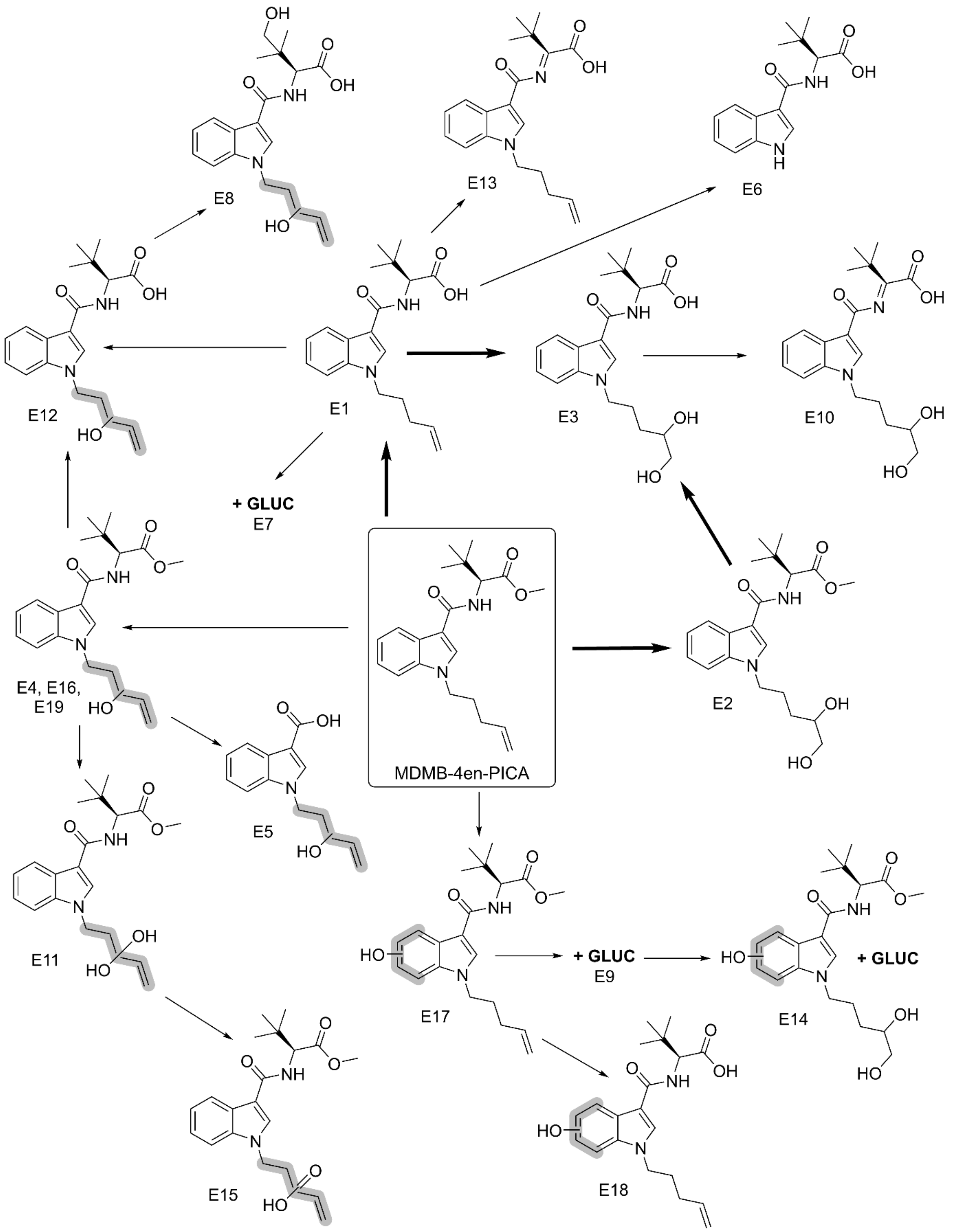

Fig. S5 Proposed metabolic pathways of MDMB-4en-PICA following incubation with human hepatocytes

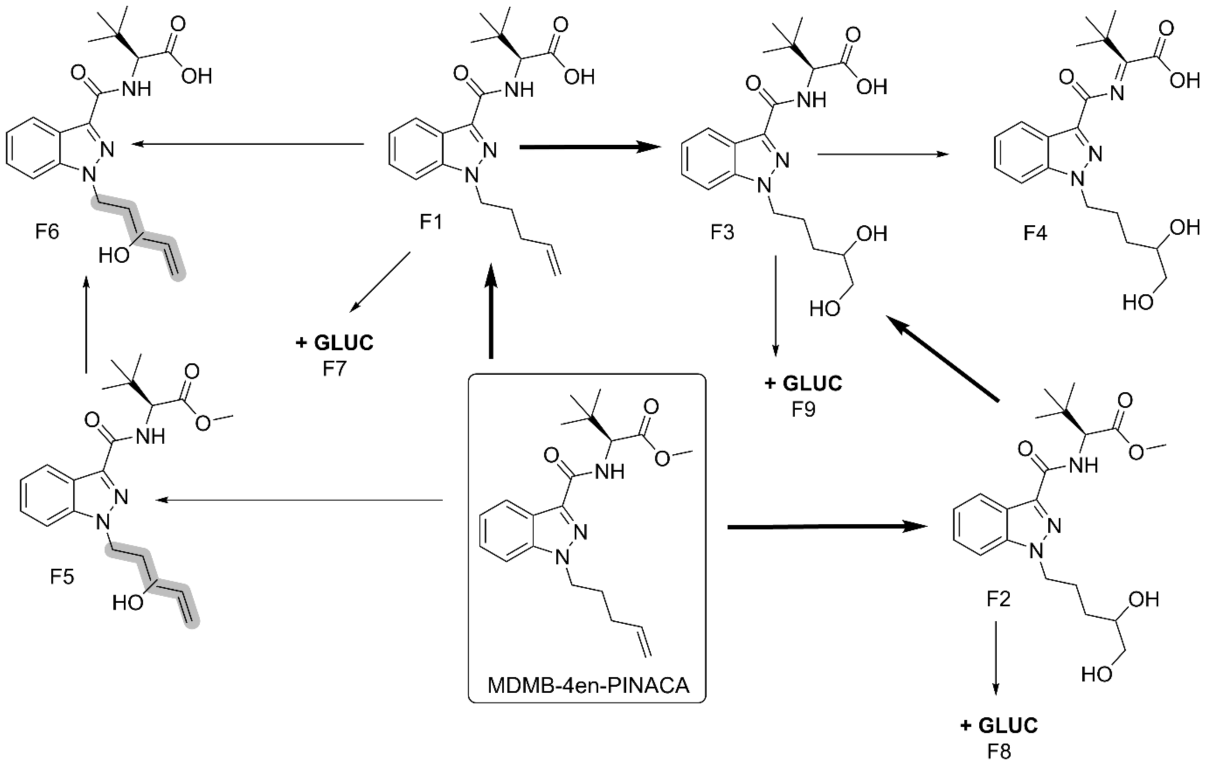

Fig. S6 Proposed metabolic pathways of MDMB-4en-PINACA following incubation with human hepatocytes

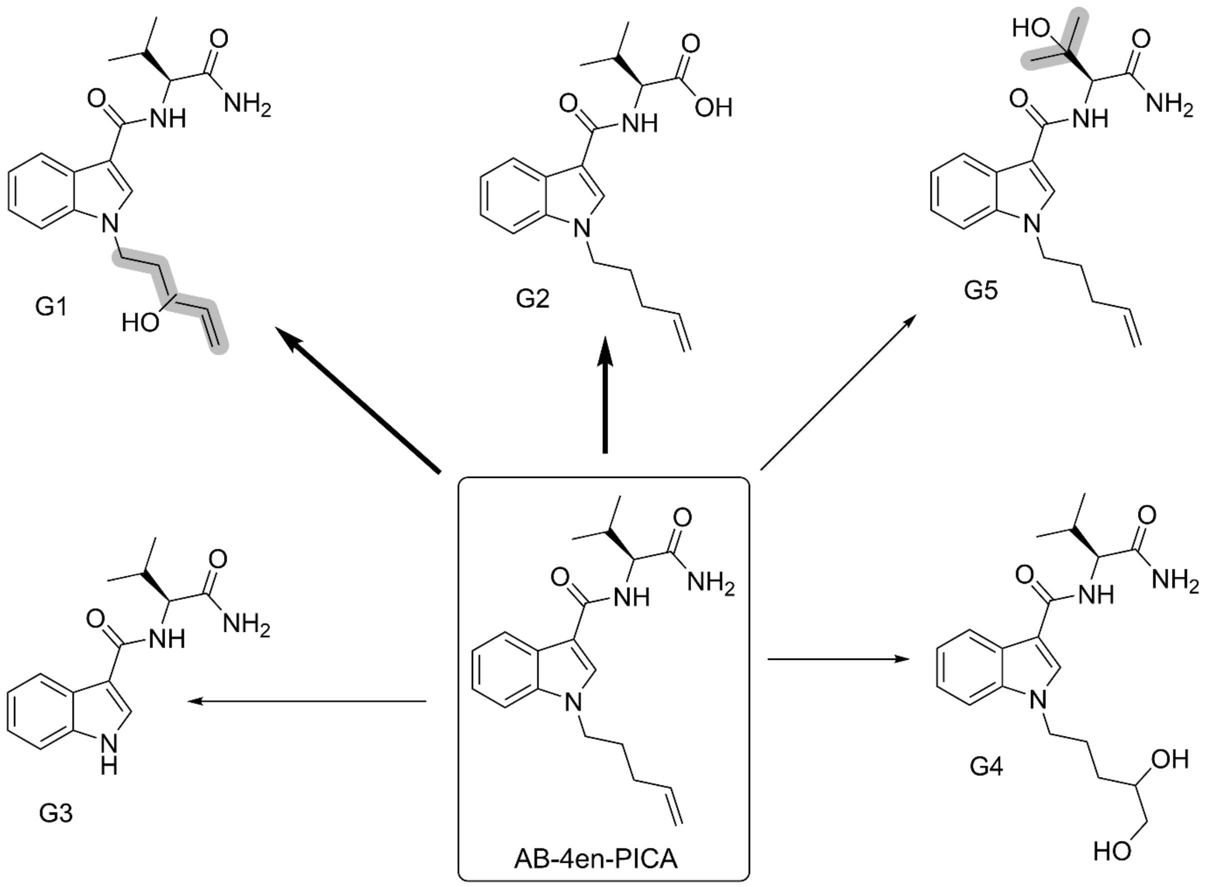

Fig. S7 Proposed metabolic pathways of AB-4en-PICA following incubation with human hepatocytes

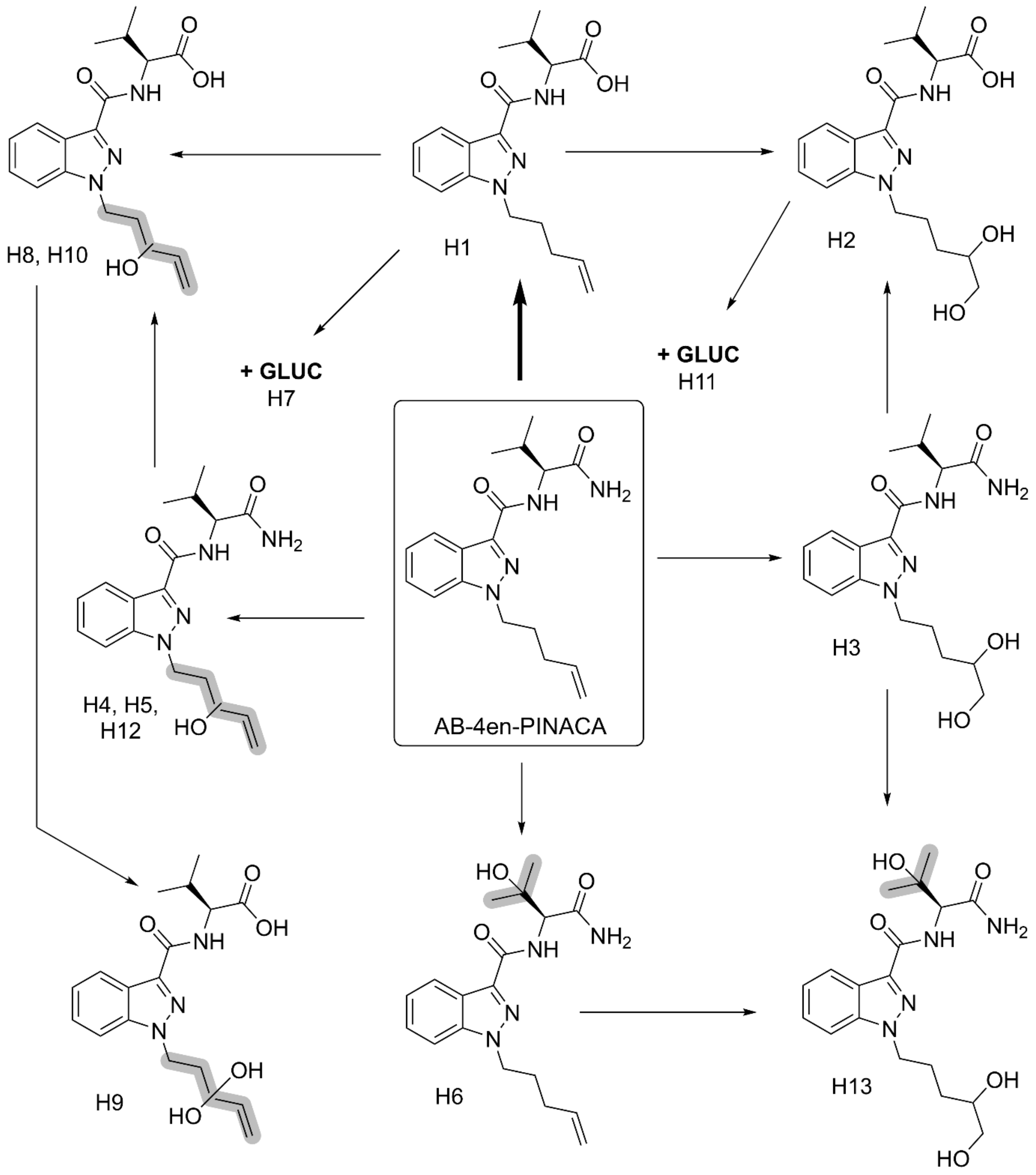

Fig. S8 Proposed metabolic pathways of AB-4en-PINACA following incubation with human hepatocytes

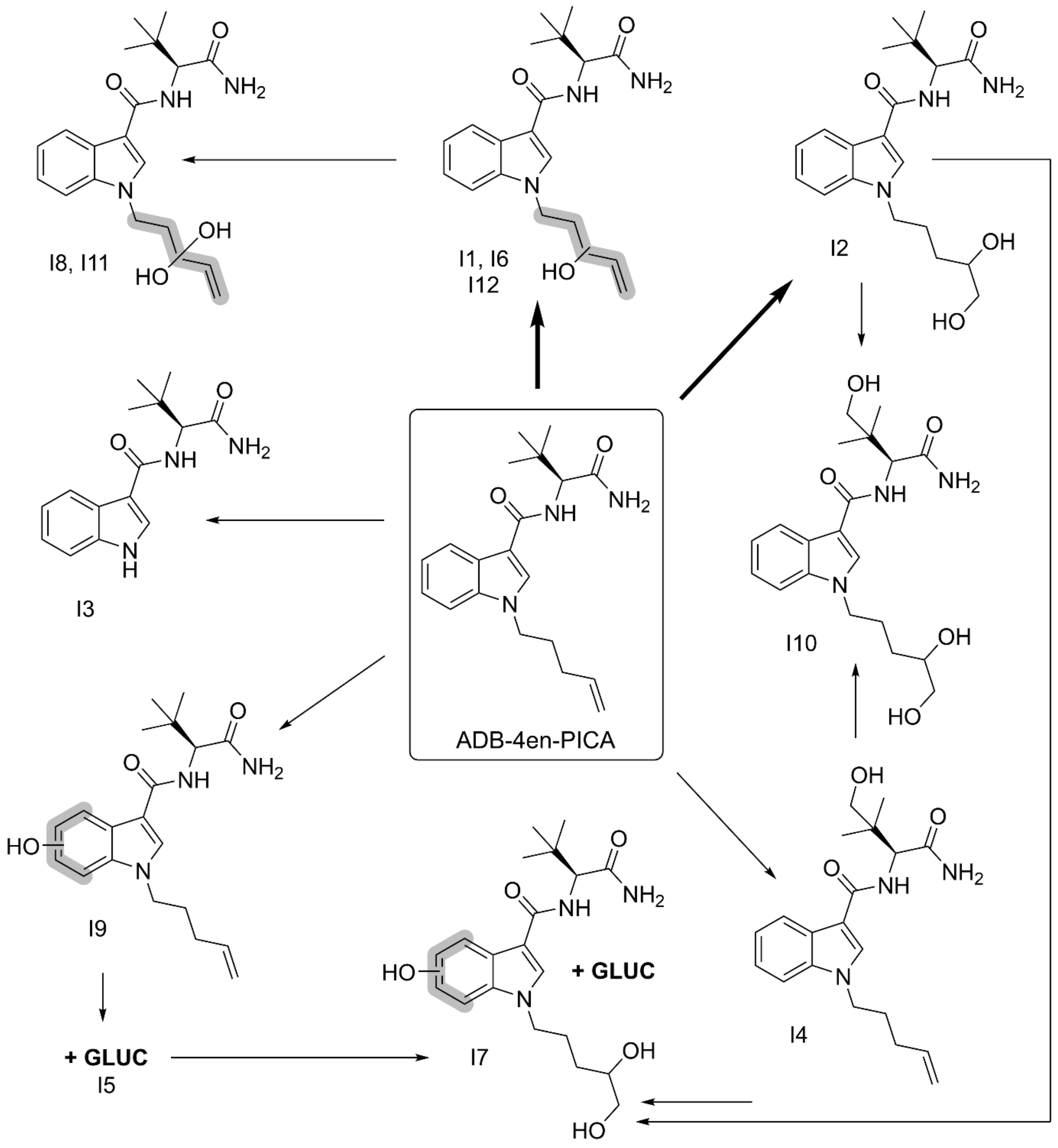

Fig. S9 Proposed metabolic pathways of ADB-4en-PICA following incubation with human hepatocytes

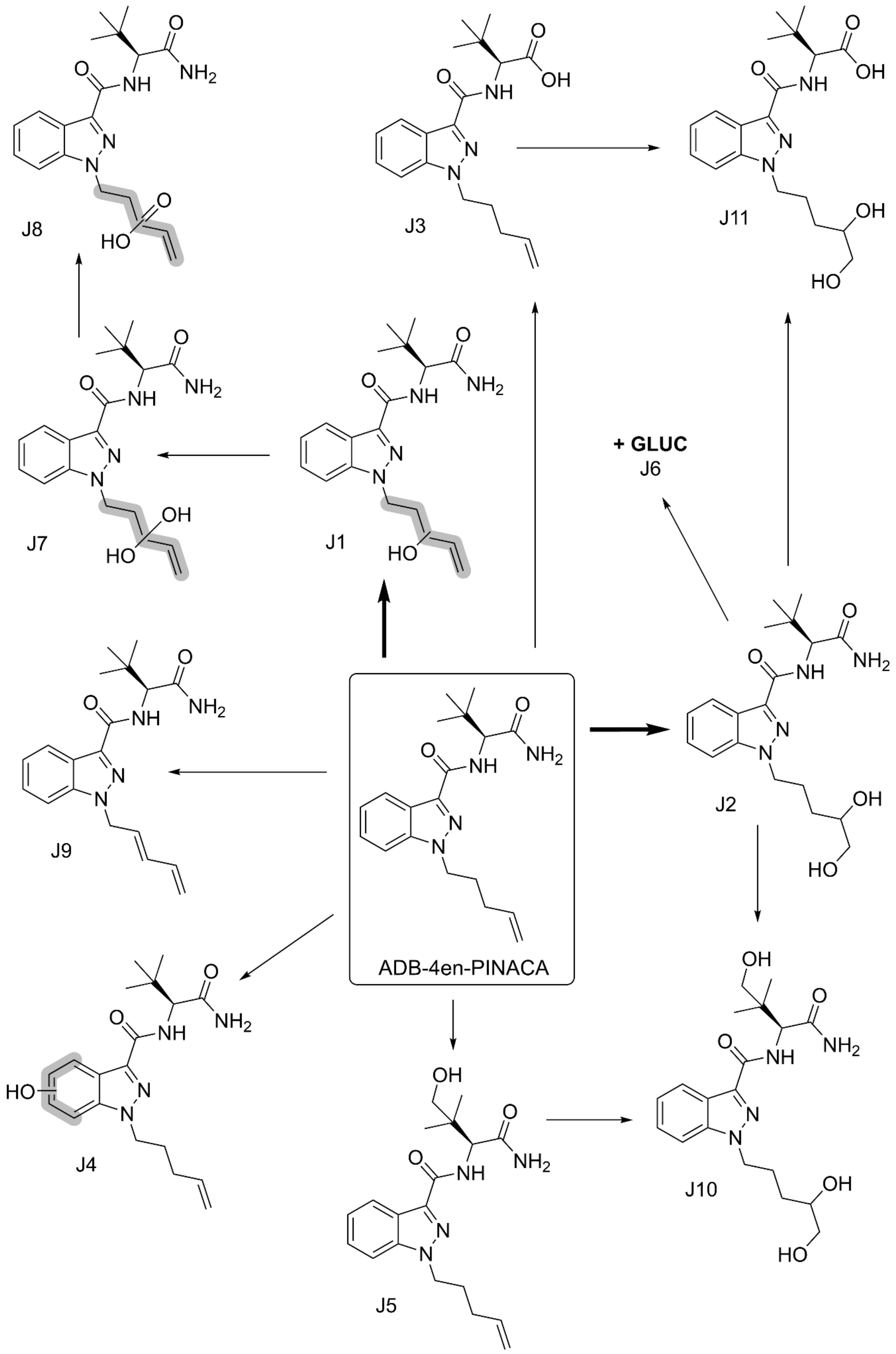

Fig. S10 Proposed metabolic pathways of ADB-4en-PINACA following incubation with human hepatocytes

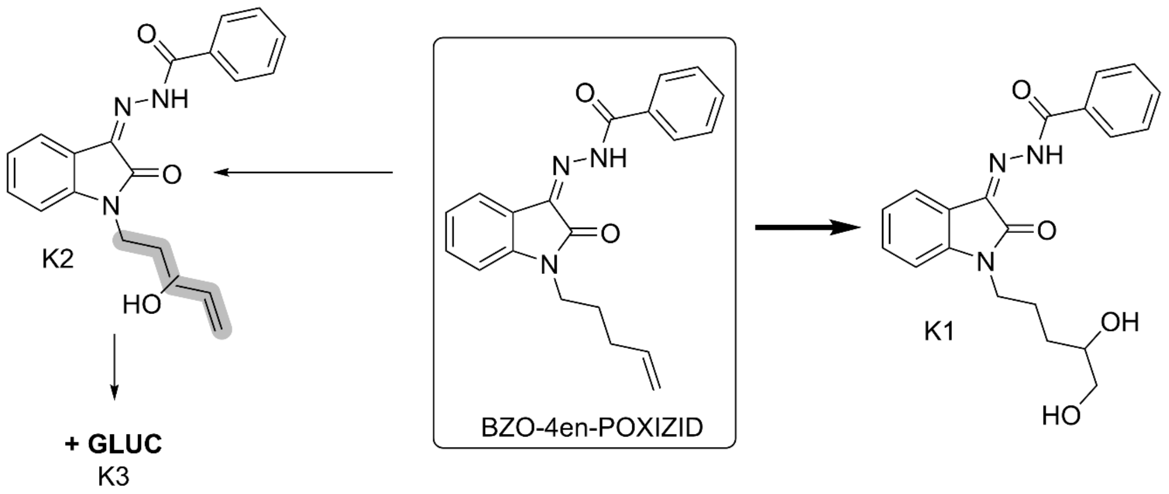

Fig. S11 Proposed metabolic pathways of BZO-4en-POXIZID following incubation with human hepatocytes
