# Supplementary material for: Structure–metabolism relationships of 4-pentenyl synthetic cannabinoid receptor agonists using in vitro human hepatocyte incubations and high-resolution mass spectrometry
Source: Arch Toxicol. 2025 May 20;99(8):3331–41. doi: 10.1007/s00204-025-04080-6 (PMC12367891; doi:10.1007/s00204-025-04080-6)
Supplement: Supplementary file 3 — Supplementary file3 (PDF 338 KB) [file 204_2025_4080_MOESM3_ESM.pdf]

Structure–metabolism relationships of 4-pentenyl synthetic cannabinoid receptor agonists using *in vitro* human hepatocyte incubations and high-resolution mass spectrometry

Supplementary Information for Archives of Toxicology – Metabolite Tables

Steven R Baginski[1,*], Karin Lindbom[2], Bryan Valencia Crespo[3], Ghidaa Bessa[3], Tobias Rautio[3], Xiongyu Wu[3], Johan Dahlén[3], Lorna A Nisbet[1], Craig McKenzie[1,4], Henrik Gréen[2,5,*]

[1] Leverhulme Research Centre for Forensic Science, School of Science and Engineering, University of Dundee, Dundee, UK

[2] Division of Clinical Chemistry and Pharmacology, Department of Biomedical and Clinical Sciences, Linköping University, Linköping, Sweden

[3] Department of Physics, Chemistry and Biology, Linköping University, Linköping, Sweden

[4] Chiron AS, Trondheim, Norway

[5] Department of Forensic Genetics and Forensic Toxicology, National Board of Forensic Medicine, Linköping, Sweden

*Corresponding authors: Steven Baginski, sbaginski001@dundee.ac.uk and Henrik Gréen, henrik.green@liu.se

Table S1 MMB-4en-PICA metabolites with biotransformation, chemical formula, accurate mass of the protonated molecule, mass error (maximum and minimum), retention time (RT), peak area and metabolite percentages

				**Mass error (ppm)**			**Peak area (× 1000)**							
**Met ID**	**Biotransformation**	**Chemical formula**	**$[M+H]^+$ (*m/z*)**	**Max**	**Min**	**Mean RT (min)**	**0.5 h - A**	**0.5 h - B**	**1 h - A**	**1 h - B**	**3 h - A**	**3 h - B**	**Total**	**% Met Total**
	MMB-4en-PICA	$C_{20}H_{26}N_2O_3$	343.2038	9.54	0.34	11.07	4,043	4,552	1,123	1,363	204	215	11,500	–
A1	Ester hydrolysis	$C_{19}H_{24}N_2O_3$	329.1879	6.18	0.32	9.15	3,817	4,310	4,240	4,410	3,421	3,351	23,549	85.8%
A2	Ester hydrolysis + dihydrodiol formation	$C_{19}H_{26}N_2O_5$	363.1919	2.03	0.70	4.66	163	102	242	284	325	347	1,464	5.3%
A3	Ester hydrolysis + glucuronidation	$C_{25}H_{32}N_2O_9$	505.2180	0.90	0.24	7.49	102	115	168	155	248	248	1,036	3.8%
A4	Head group–linker cleavage	$C_{14}H_{16}N_2O$	229.1340	2.44	1.34	6.91	99	78	129	138	76	73	594	2.2%
A5	Ester hydrolysis + mono-hydroxylation (pentenyl tail)	$C_{19}H_{24}N_2O_4$	345.1811	−1.76	0.28	6.31	33	30	47	58	58	57	284	1.0%
A6	Secondary amide hydrolysis + mono-hydroxylation (pentenyl tail)	$C_{14}H_{15}NO_3$	246.1129	1.82	−0.40	4.66	31	*20*	43	55	60	63	272	1.0%
A7	Dihydrodiol formation	$C_{20}H_{28}N_2O_5$	377.2069	2.00	0.09	6.09	49	54	49	55	*13*	*12*	233	0.8%
												**Metabolite Total**	27,432	100%

Italics: peak areas below or around 20,000 counts, which were manually assessed.

Table S2 MMB-4en-PINACA metabolites with biotransformation, chemical formula, accurate mass of the protonated molecule, mass error (maximum and minimum), retention time (RT), peak area and metabolite percentages

Met ID	Biotransformation	Chemical formula	$[M+H]^+$ (*m/z*)	Mass error (ppm)		Mean RT (min)	Peak area (× 1000)							% Met Total
				Max	Min		0.5 h - A	0.5 h - B	1 h - A	1 h - B	3 h - A	3 h - B	Total	
	MMB-4en-PINACA	$C_{19}H_{25}N_3O_3$	344.1989	5.25	0.69	12.05	938	860	524	549	234	167	3,274	−
B1	Ester hydrolysis	$C_{18}H_{23}N_3O_3$	330.1827	5.44	3.77	9.72	7,429	9,013	6,409	7,511	3,907	5,023	39,293	88.6%
B2	Ester hydrolysis + dihydrodiol formation	$C_{18}H_{25}N_3O_5$	364.1873	1.85	0.06	4.85	402	604	319	352	760	731	3,167	7.1%
B3	Ester hydrolysis + glucuronidation	$C_{24}H_{31}N_3O_9$	506.2122	−2.23	−0.62	7.68	70	94	63	71	154	186	638	1.4%
B4	Ester hydrolysis + mono-hydroxylation (pentenyl tail)	$C_{18}H_{23}N_3O_4$	346.1756	−1.23	−0.58	6.55	66	120	53	61	101	101	501	1.1%
B5	Ester hydrolysis + di-hydroxylation (pentenyl tail)	$C_{18}H_{23}N_3O_5$	362.1709	−1.10	−0.07	5.39	58	78	56	67	106	101	464	1.0%
B6	Dihydrodiol formation	$C_{19}H_{27}N_3O_5$	378.2026	−2.38	−0.41	6.36	65	93	40	45	*15*	*16*	274	0.6%
												**Metabolite Total**	44,337	100%

Italics: peak areas below or around 20,000 counts, which were manually assessed.

Table S3 EMB-4en-PICA metabolites with biotransformation, chemical formula, accurate mass of the protonated molecule, mass error (maximum and minimum), retention time (RT), peak area and metabolite percentages

				**Mass error (ppm)**			**Peak area (× 1000)**							
**Met ID**	**Biotransformation**	**Chemical formula**	**$[M+H]^+$ (*m/z*)**	**Max**	**Min**	**Mean RT (min)**	**0.5 h - A**	**0.5 h - B**	**1 h - A**	**1 h - B**	**3 h - A**	**3 h - B**	**Total**	**% Met Total**
	EMB-4en-PICA	$C_{21}H_{28}N_2O_3$	357.2188	6.69	0.20	10.62	8,040	6,180	2,790	2,626	572	925	21,133	−
C1	Ester hydrolysis	$C_{19}H_{24}N_2O_3$	329.1868	3.72	−0.70	8.28	5,142	5,428	3,906	3,856	4,544	4,985	27,863	89.8%
C2	Ester hydrolysis + dihydrodiol formation	$C_{19}H_{26}N_2O_5$	363.1913	−1.39	0.07	4.42	144	169	143	155	322	297	1,230	4.0%
C3	Ester hydrolysis + glucuronidation	$C_{25}H_{32}N_2O_9$	505.2179	−1.46	0.18	6.86	101	116	109	130	191	207	854	2.8%
C4	Secondary amide hydrolysis + mono-hydroxylation (pentenyl tail)	$C_{14}H_{15}NO_3$	246.1121	−2.86	0.03	4.42	65	78	67	69	149	130	558	1.8%
C5	Head group–linker cleavage	$C_{14}H_{16}N_2O$	229.1330	−4.25	0.14	6.41	37	45	20	21	29	29	181	0.6%
C6	Ester hydrolysis + mono-hydroxylation (pentenyl tail)	$C_{19}H_{24}N_2O_4$	345.1806	−2.94	−0.14	5.87	*16*	22	*18*	21	39	40	155	0.5%
C7	Dihydrodiol formation	$C_{21}H_{30}N_2O_5$	391.2225	−1.18	−0.93	6.44	34	35	*13*	*14*	ND	ND	96	0.3%
C8	Ester hydrolysis + glucuronidation	$C_{25}H_{32}N_2O_9$	505.2193	1.88	1.59	6.55	ND	ND	*6*	*7*	35	36	84	0.3%
												**Metabolite Total**	31,020	100%

Italics: peak areas below or around 20,000 counts, which were manually assessed. ND: not detected.

Table S4 EMB-4en-PINACA metabolites with biotransformation, chemical formula, accurate mass of the protonated molecule, mass error (maximum and minimum), retention time (RT), peak area and metabolite percentages

				**Mass error (ppm)**			**Peak area (× 1000)**							
**Met ID**	**Biotransformation**	**Chemical formula**	**$[M+H]^+$ (*m/z*)**	**Max**	**Min**	**Mean RT (min)**	**0.5 h - A**	**0.5 h - B**	**1 h - A**	**1 h - B**	**3 h - A**	**3 h - B**	**Total**	**% Met Total**
	EMB-4en-PINACA	$C_{20}H_{27}N_3O_3$	358.2146	6.76	3.43	11.61	11,065	10,619	7,869	7,786	3,048	3,037	43,424	−
D1	Ester hydrolysis	$C_{18}H_{23}N_3O_3$	330.1834	7.02	5.67	8.84	9,777	8,952	7,553	7,315	6,804	7,025	47,425	92.3%
D2	Ester hydrolysis + dihydrodiol formation	$C_{18}H_{25}N_3O_5$	364.1875	3.60	0.96	4.63	308	301	303	360	681	645	2,598	5.1%
D3	Ester hydrolysis + glucuronidation	$C_{24}H_{31}N_3O_9$	506.2134	1.78	0.33	7.14	90	104	94	91	96	100	574	1.1%
D4	Ester hydrolysis + dihydrodiol formation + glucuronidation	$C_{24}H_{33}N_3O_{11}$	540.2190	3.24	−0.40	3.73	40	42	43	45	67	66	304	0.6%
D5	Ester hydrolysis + di-hydroxylation (pentenyl tail)	$C_{18}H_{23}N_3O_5$	362.1714	2.48	−0.66	5.12	29	27	25	31	59	63	232	0.5%
D6	Ester hydrolysis + mono-hydroxylation (pentenyl tail)	$C_{20}H_{27}N_3O_3$	346.1764	1.59	1.44	6.11	21	21	*20*	21	41	40	164	0.3%
D7	Ester hydrolysis + mono-hydroxylation (pentenyl tail)	$C_{18}H_{23}N_3O_3$	346.1770	2.67	3.43	6.19	*11*	*11*	*11*	*14*	24	21	80	0.2%
												**Metabolite Total**	51,379	100%

Italics: peak areas below or around 20,000 counts, which were manually assessed.

Table S5 MDMB-4en-PICA metabolites with biotransformation, chemical formula, accurate mass of the protonated molecule, mass error (maximum and minimum), retention time (RT), peak area and metabolite percentages

				**Mass error (ppm)**			**Peak area (× 1000)**							
**Met ID**	**Biotransformation**	**Chemical formula**	**[M+H]$^+$ (*m/z*)**	**Max**	**Min**	**Mean RT (min)**	**0.5 h - A**	**0.5 h - B**	**1 h - A**	**1 h - B**	**3 h - A**	**3 h - B**	**Total**	**% Met Total**
	MDMB-4en-PICA	$C_{21}H_{28}N_2O_3$	357.2209	9.65	3.49	12.04	4,563	3,494	3,163	3,206	3,738	3,861	22,025	−
E1	Ester hydrolysis	$C_{20}H_{26}N_2O_3$	343.2026	4.00	2.71	9.94	1,321	1,096	1,181	1,167	1,536	1,575	7,877	35.7%
E2	Dihydrodiol formation	$C_{21}H_{30}N_2O_5$	391.2238	2.42	1.81	7.05	1,382	934	643	632	369	387	4,347	19.7%
E3	Ester hydrolysis + dihydrodiol formation	$C_{20}H_{28}N_2O_5$	377.2072	2.26	−0.06	5.42	76	121	222	304	372	373	1,468	6.7%
E4	Mono-hydroxylation (pentenyl tail)	$C_{21}H_{28}N_2O_4$	373.2131	1.92	0.15	9.08	407	247	147	132	52	53	1,038	4.7%
E5	Secondary amide hydrolysis + mono-hydroxylation (pentenyl tail)	$C_{14}H_{15}NO_3$	246.1133	3.81	1.59	7.05	332	222	150	147	83	82	1,018	4.6%
E6	Ester hydrolysis + *N*-dealkylation	$C_{15}H_{18}N_2O_3$	275.1396	3.81	1.62	5.71	130	110	137	155	228	246	1,007	4.6%
E7	Ester hydrolysis + glucuronidation	$C_{26}H_{34}N_2O_9$	519.2341	1.59	0.09	8.13	71	67	101	94	309	316	959	4.3%
E8	Ester hydrolysis + di-hydroxylation (pentenyl tail + *tert*-butyl)	$C_{20}H_{26}N_2O_5$	375.1917	1.25	0.04	7.95	121	105	81	85	166	188	745	3.4%
E9	Mono-hydroxylation + glucuronidation (indole core)	$C_{27}H_{36}N_2O_{10}$	549.2434	−1.49	−0.10	6.74	132	112	91	82	137	158	713	3.2%
E10	Ester hydrolysis + dihydrodiol formation + dehydrogenation	$C_{20}H_{26}N_2O_5$	375.1916	−0.77	0.01	4.91	94	72	78	83	113	116	556	2.5%
E11	Di-hydroxylation (pentenyl tail)	$C_{21}H_{28}N_2O_5$	389.2077	1.16	−0.27	8.47	101	81	70	72	86	93	503	2.3%
E12	Ester hydrolysis + mono-hydroxylation (pentenyl tail)	$C_{20}H_{26}N_2O_4$	359.1966	1.09	0.00	7.14	43	46	72	86	96	93	436	2.0%
E13	Ester hydrolysis + dehydrogenation	$C_{20}H_{24}N_2O_3$	341.1863	−1.14	−0.13	9.81	121	74	51	40	33	36	354	1.6%
E14	Dihydrodiol formation + mono-hydroxylation (indole core) + glucuronidation	$C_{27}H_{38}N_2O_{12}$	583.2490	−1.29	−0.34	4.11	38	36	33	33	59	67	266	1.2%
E15	Ketone formation + mono-hydroxylation (pentenyl tail)	$C_{21}H_{26}N_2O_5$	387.1915	−1.22	0.01	8.16	47	34	27	29	40	43	220	1.0%
E16	Mono-hydroxylation (pentenyl tail)	$C_{21}H_{28}N_2O_4$	373.2117	−1.65	0.03	8.53	70	36	23	21	*9*	*9*	168	0.8%
E17	Mono-hydroxylation (indole core)	$C_{21}H_{28}N_2O_4$	373.2121	0.84	0.05	9.29	29	57	31	23	*11*	*11*	164	0.7%

E18	Ester hydrolysis + mono-hydroxylation (indole core)	$C_{20}H_{26}N_2O_4$	359.1963	−1.26	0.22	7.38	13	24	24	21	32	33	147	0.7%
E19	Mono-hydroxylation (pentenyl tail)	$C_{21}H_{28}N_2O_4$	373.2125	−0.28	0.28	8.41	33	20	*10*	*10*	*5*	*6*	86	0.4%
												**Metabolite Total**	22,072	100%

Italics: peak areas below or around 20,000 counts, which were manually assessed.

Table S6 MDMB-4en-PINACA metabolites with biotransformation, chemical formula, accurate mass of the protonated molecule, mass error (maximum and minimum), retention time (RT), peak area and metabolite percentages

				**Mass error (ppm)**			**Peak area (× 1000)**							
**Met ID**	**Biotransformation**	**Chemical formula**	**[M+H]$^+$ (*m/z*)**	**Max**	**Min**	**Mean RT (min)**	**0.5 h - A**	**0.5 h - B**	**1 h - A**	**1 h - B**	**3 h - A**	**3 h - B**	**Total**	**% Met Total**
	MDMB-4en-PINACA	$C_{20}H_{27}N_3O_3$	358.2161	10.54	2.25	13.02	23,470	17,844	17,098	23,602	1,579	1,692	85,286	−
F1	Ester hydrolysis	$C_{19}H_{25}N_3O_3$	344.1981	3.23	2.09	10.59	2,735	2,612	2,360	3,028	1,827	1,783	14,344	49.7%
F2	Dihydrodiol formation	$C_{20}H_{29}N_3O_5$	392.2188	−2.13	−0.45	7.29	2,075	1,512	747	771	44	70	5,218	18.1%
F3	Ester hydrolysis + dihydrodiol formation	$C_{19}H_{27}N_3O_5$	378.2026	1.61	0.39	5.62	215	690	388	419	710	696	3,117	10.8%
F4	Ester hydrolysis + dihydrodiol formation + dehydrogenation	$C_{19}H_{25}N_3O_5$	376.1870	0.67	−0.09	5.14	572	590	322	333	129	179	2,125	7.4%
F5	Mono-hydroxylation (pentenyl tail)	$C_{20}H_{27}N_3O_4$	374.2077	1.25	0.27	9.59	617	365	190	226	ND	ND	1,397	4.8%
F6	Ester hydrolysis + mono-hydroxylation (pentenyl tail)	$C_{19}H_{25}N_3O_4$	360.1913	−1.51	0.22	7.39	149	136	116	127	116	140	784	2.7%
F7	Ester hydrolysis + glucuronidation	$C_{25}H_{33}N_3O_9$	520.2287	−1.45	−0.29	8.41	84	103	86	93	148	152	667	2.3%
F8	Dihydrodiol formation + glucuronidation	$C_{26}H_{37}N_3O_{11}$	568.2497	−2.67	−0.39	6.02	129	165	88	101	69	85	637	2.2%
F9	Ester hydrolysis + dihydrodiol + glucuronidation	$C_{25}H_{35}N_3O_{11}$	554.2337	−1.71	0.17	4.47	39	74	69	76	163	156	577	2.0%
												**Metabolite Total**	28,866	100%

ND: not detected.

Table S7 AB-4en-PICA metabolites with biotransformation, chemical formula, accurate mass of the protonated molecule, mass error (maximum and minimum), retention time (RT), peak area and metabolite percentages

Met ID	Biotransformation	Chemical formula	$[M+H]^+$ (*m/z*)	Mass error (ppm) Max	Mass error (ppm) Min	Mean RT (min)	Peak area (× 1000) 0.5 h - A	0.5 h - B	1 h - A	1 h - B	3 h - A	3 h - B	Total	% Met Total
	AB-4en-PICA	$C_{19}H_{25}N_3O_2$	328.2034	6.28	3.26	8.14	5,378	7,721	5,485	5,194	3,083	3,525	30,387	−
G1	Mono-hydroxylation (pentenyl tail)	$C_{19}H_{25}N_3O_3$	344.1973	0.80	0.04	5.45	217	319	262	243	322	357	1,719	39.3%
G2	Terminal amide hydrolysis	$C_{19}H_{24}N_2O_3$	329.1862	1.19	0.18	9.08	58	100	94	85	253	293	882	20.2%
G3	*N*-dealkylation	$C_{14}H_{17}N_3O_2$	260.1395	1.59	0.61	3.95	83	94	97	97	156	186	714	16.3%
G4	Dihydrodiol formation	$C_{19}H_{27}N_3O_4$	362.2076	4.72	0.40	3.97	ND	ND	24	83	284	291	680	15.6%
G5	Mono-hydroxylation (*iso*-propyl)	$C_{19}H_{25}N_3O_3$	344.1970	−0.88	0.24	6.82	42	74	44	49	79	89	376	8.6%
												**Metabolite Total**	4,372	100%

ND: not detected.

Table S8 AB-4en-PINACA metabolites with biotransformation, chemical formula, accurate mass of the protonated molecule, mass error (maximum and minimum), retention time (RT), peak area and metabolite percentages

Met ID	Biotransformation	Chemical formula	$[M+H]^+$ (*m/z*)	Mass error (ppm) Max	Mass error (ppm) Min	Mean RT (min)	Peak area (× 1000) 0.5 h - A	0.5 h - B	1 h - A	1 h - B	3 h - A	3 h - B	Total	% Met Total
	AB-4en-PINACA	$C_{18}H_{24}N_4O_2$	329.2005	11.14	2.59	8.61	7,682	8,651	9,093	9,107	1,185	1,189	36,908	−
H1	Terminal amide hydrolysis	$C_{18}H_{23}N_3O_3$	330.1840	7.13	4.58	9.77	3,255	3,643	4,409	5,641	5,395	6,058	28,402	68.6%
H2	Terminal amide hydrolysis + dihydrodiol formation	$C_{18}H_{25}N_3O_5$	364.1877	2.73	1.28	4.88	377	437	425	518	1,367	1,371	4,494	10.8%
H3	Dihydrodiol formation	$C_{18}H_{26}N_4O_4$	363.2032	1.70	0.89	4.04	412	491	454	527	460	499	2,842	6.9%
H4	Mono-hydroxylation (pentenyl tail)	$C_{18}H_{24}N_4O_3$	345.1923	−1.23	0.09	5.55	196	255	218	229	65	70	1,033	2.5%
H5	Mono-hydroxylation (pentenyl tail)	$C_{18}H_{24}N_4O_3$	345.1920	0.94	0.14	5.62	177	235	188	210	76	88	973	2.3%
H6	Mono-hydroxylation (*iso*-propyl)	$C_{18}H_{24}N_4O_3$	345.1924	0.94	−0.19	7.08	115	152	142	136	91	104	739	1.8%
H7	Terminal amide hydrolysis + glucuronidation	$C_{24}H_{31}N_3O_9$	506.2126	−1.18	0.02	7.70	52	55	63	76	194	237	678	1.6%
H8	Terminal amide hydrolysis + mono-hydroxylation (pentenyl tail)	$C_{18}H_{23}N_3O_4$	346.1762	−0.62	0.07	6.58	68	70	69	83	177	165	632	1.5%
H9	Terminal amide hydrolysis + di-hydroxylation (pentenyl tail)	$C_{18}H_{23}N_3O_5$	362.1704	−1.45	0.07	5.42	30	34	39	54	132	139	429	1.0%
H10	Terminal amide hydrolysis + mono-hydroxylation (pentenyl tail)	$C_{18}H_{23}N_3O_4$	346.1764	−1.17	0.37	6.67	40	47	39	49	123	111	410	1.0%
H11	Terminal amide hydrolysis + dihydrodiol formation + glucuronidation	$C_{24}H_{33}N_3O_{11}$	540.2180	−1.66	−0.62	3.87	33	37	39	44	108	108	369	0.9%
H12	Mono-hydroxylation (pentenyl tail)	$C_{18}H_{24}N_4O_3$	345.1917	−0.90	−0.17	6.39	39	52	44	41	27	25	227	0.5%
H13	Dihydrodiol formation + mono-hydroxylation (*iso*-propyl)	$C_{18}H_{26}N_4O_5$	379.1970	−1.73	−0.14	3.16	32	38	31	30	39	34	204	0.5%
												**Metabolite Total**	41,430	100%

Table S9 ADB-4en-PICA metabolites with biotransformation, chemical formula, accurate mass of the protonated molecule, mass error (maximum and minimum), retention time (RT), peak area and metabolite percentages

				**Mass error (ppm)**			**Peak area (× 1000)**							
**Met ID**	**Biotransformation**	**Chemical formula**	**$[M+H]^+$ (*m/z*)**	**Max**	**Min**	**Mean RT (min)**	**0.5 h - A**	**0.5 h - B**	**1 h - A**	**1 h - B**	**3 h - A**	**3 h - B**	**Total**	**% Met Total**
	ADB-4en-PICA	$C_{20}H2_{7}N_3O_2$	342.2210	8.56	5.94	9.06	8,518	8,611	7,753	8,073	4,745	4,616	42,316	−
I1	Mono-hydroxylation (pentenyl tail)	$C_{20}H_{27}N_3$ O_3	358.2133	2.82	1.34	6.30	813	488	731	761	832	871	4,495	39.0%
I2	Dihydrodiol formation	$C_{20}H_{29}N_3O_4$	376.2238	4.70	4.69	4.69	407	267	487	508	740	818	3,226	28.0%
I3	*N*-dealkylation	$C_{15}H_{19}N_3O_2$	274.1555	1.93	0.43	4.82	169	104	155	151	216	222	1,017	8.8%
I4	Mono-hydroxylation (*tert*-butyl)	$C_{20}H_{27}N_3O_3$	358.2131	1.55	−0.01	7.36	139	74	117	118	138	160	745	6.5%
I5	Mono-hydroxylation (indole core) + glucuronidation	$C_{26}H_{35}N_3O_9$	534.2440	−1.11	−0.16	4.49	55	41	85	82	129	151	544	4.7%
I6	Mono-hydroxylation (pentenyl tail)	$C_{20}H_{27}N_3O_3$	358.2122	0.67	0.00	7.05	32	24	43	39	59	65	262	2.3%
I7	Dihydrodiol formation + mono-hydroxylation (indole core) + glucuronidation	$C_{26}H_{37}N_3O_{11}$	568.2495	−2.19	−0.55	2.58	29	25	43	40	57	65	258	2.2%
I8	Di-hydroxylation (pentenyl tail)	$C_{20}H_{27}N_3O_4$	374.2073	1.21	−0.07	5.91	27	22	34	38	56	61	238	2.1%
I9	Mono-hydroxylation (indole core)	$C_{20}H_{27}N_3O_3$	358.2118	−3.00	−0.78	6.54	38	*22*	41	40	48	38	227	2.0%
I10	Dihydrodiol formation + mono-hydroxylation (*tert*-butyl)	$C_{20}H_{29}N_3O_5$	392.2178	−1.53	−0.43	3.42	25	*17*	27	40	48	54	210	1.8%
I11	Di-hydroxylation (pentenyl tail)	$C_{20}H_{27}N_3O_4$	374.2069	−2.04	−0.22	5.10	24	*18*	24	30	41	40	177	1.5%
I12	Mono-hydroxylation (pentenyl tail)	$C_{20}H_{27}N_3O_3$	358.2121	−2.31	−0.14	5.76	23	21	22	24	*20*	21	129	1.1%
												**Metabolite Total**	11,528	100%

Italics: peak areas below or around 20,000 counts, which were manually assessed.

Table S10 ADB-4en-PINACA metabolites with biotransformation, chemical formula, accurate mass of the protonated molecule, mass error (maximum and minimum), retention time (RT), peak area and metabolite percentages

Met ID	Biotransformation	Chemical formula	$[M+H]^+$ (*m/z*)	Mass error (ppm) Max	Min	Mean RT (min)	Peak area (× 1000) 0.5 h - A	0.5 h - B	1 h - A	1 h - B	3 h - A	3 h - B	Total	% Met Total
	ADB-4en-PINACA	$C_{19}H_{26}N_4O_2$	343.2150	9.19	3.17	9.54	11,598	19,124	13,658	14,698	5,186	8,462	72,727	−
J1	Mono-hydroxylation (pentenyl tail)	$C_{19}H_{26}N_4O_3$	359.2081	1.31	0.47	6.41	1,072	1,412	1,195	1,262	1,073	994	7,007	37.4%
J2	Dihydrodiol formation	$C_{19}H_{28}N_4O_4$	377.2184	1.83	0.05	4.76	749	793	836	874	1,445	1,277	5,973	31.9%
J3	Terminal amide hydrolysis	$C_{19}H_{25}N_3O_3$	344.1966	1.42	0.46	10.58	77	92	130	141	382	522	1,344	7.2%
J4	Mono-hydroxylation (indazole core)	$C_{19}H_{26}N_4O_3$	359.2079	−2.32	0.00	7.30	119	155	246	147	190	139	996	5.3%
J5	Mono-hydroxylation (*tert*-butyl)	$C_{19}H_{26}N_4O_3$	359.2077	−4.42	−0.23	7.52	120	130	92	109	100	107	658	3.5%
J6	Dihydrodiol formation + glucuronidation	$C_{25}H_{36}N_4O_{10}$	553.2500	−2.02	−0.41	4.05	63	68	79	81	182	139	612	3.3%
J7	Di-hydroxylation (pentenyl tail)	$C_{19}H_{26}N_4O_4$	375.2021	−2.62	−0.59	5.27	65	91	102	113	84	147	602	3.2%
J8	Ketone formation + mono-hydroxylation (pentenyl tail)	$C_{19}H_{24}N_4O_4$	373.1863	−1.84	−0.19	5.71	73	60	69	72	127	106	506	2.7%
J9	Dehydrogenation (pentenyl tail)	$C_{19}H_{24}N_4O_2$	341.1973	−2.63	−0.45	8.97	52	79	72	73	78	57	411	2.2%
J10	Dihydrodiol formation + mono-hydroxylation (*tert*-butyl)	$C_{19}H_{28}N_4O_5$	393.2126	−2.40	−0.86	3.42	71	54	50	48	88	43	354	1.9%
J11	Terminal amide hydrolysis + dihydrodiol formation	$C_{19}H_{27}N_3O_5$	378.2014	−3.88	−0.14	5.60	26	*18*	31	27	95	67	264	1.4%
												**Metabolite Total**	18,727	100%

Italics: peak areas below or around 20,000 counts, which were manually assessed.

Table S11 BZO-4en-POXIZID metabolites with biotransformation, chemical formula, accurate mass of the protonated molecule, mass error (maximum and minimum), retention time (RT), peak area and metabolite percentages

				**Mass error (ppm)**			**Peak area (× 1000)**							
**Met ID**	**Biotransformation**	**Chemical formula**	**[M+H]$^+$ (*m/z*)**	**Max**	**Min**	**Mean RT (min)**	**0.5 h - A**	**0.5 h - B**	**1 h - A**	**1 h - B**	**3 h - A**	**3 h - B**	**Total**	**% Met Total**
	BZO-4en-POXIZID	$C_{20}H_{19}N_3O_2$	334.1563	4.96	0.68	10.69	9,877	9,492	4,816	5,442	1,505	1,648	32,780	−
K1	Dihydrodiol formation	$C_{20}H_{21}N_3O_4$	368.1606	0.41	0.04	5.76	151	134	83	91	74	83	617	61.5%
K2	Mono-hydroxylation (pentenyl tail) + glucuronidation	$C_{26}H_{27}N_3O_9$	526.1818	−0.73	0.09	6.89	36	36	33	38	63	70	277	27.6%
K3	Mono-hydroxylation (pentenyl tail)	$C_{20}H_{19}N_3O_3$	350.1498	−0.68	0.16	7.73	35	30	*15*	*15*	*7*	*7*	109	10.9%
												**Metabolite Total**	1,002	100%

Italics: peak areas below or around 20,000 counts, which were manually assessed.
