# Supplementary material for: Structure–metabolism relationships of 4-pentenyl synthetic cannabinoid receptor agonists using in vitro human hepatocyte incubations and high-resolution mass spectrometry
Source: Arch Toxicol. 2025 May 20;99(8):3331–41. doi: 10.1007/s00204-025-04080-6 (PMC12367891; doi:10.1007/s00204-025-04080-6)

Structure–metabolism relationships of 4-pentenyl synthetic cannabinoid receptor agonists using *in vitro* human hepatocyte incubations and high-resolution mass spectrometry

Supplementary Information for Archives of Toxicology – NMR Spectra

Steven R Baginski[1,*], Karin Lindbom[2], Bryan Valencia Crespo[3], Ghidaa Bessa[3], Tobias Rautio[3], Xiongyu Wu[3], Johan Dahlén[3], Lorna A Nisbet[1], Craig McKenzie[1,4], Henrik Gréen[1,5,*]

[1] Leverhulme Research Centre for Forensic Science, School of Science and Engineering, University of Dundee, Dundee, UK

[2] Division of Clinical Chemistry and Pharmacology, Department of Biomedical and Clinical Sciences, Linköping University, Linköping, Sweden

[3] Department of Physics, Chemistry and Biology, Linköping University, Linköping, Sweden

[4] Chiron AS, Trondheim, Norway

[5] Department of Forensic Genetics and Forensic Toxicology, National Board of Forensic Medicine, Linköping, Sweden

*Corresponding authors: Steven Baginski, sbaginski001@dundee.ac.uk and Henrik Gréen, henrik.green@liu.se

MMB-4en-PICA

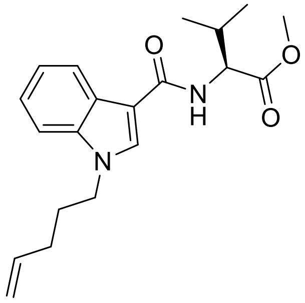

^{1}H-NMR Spectrum

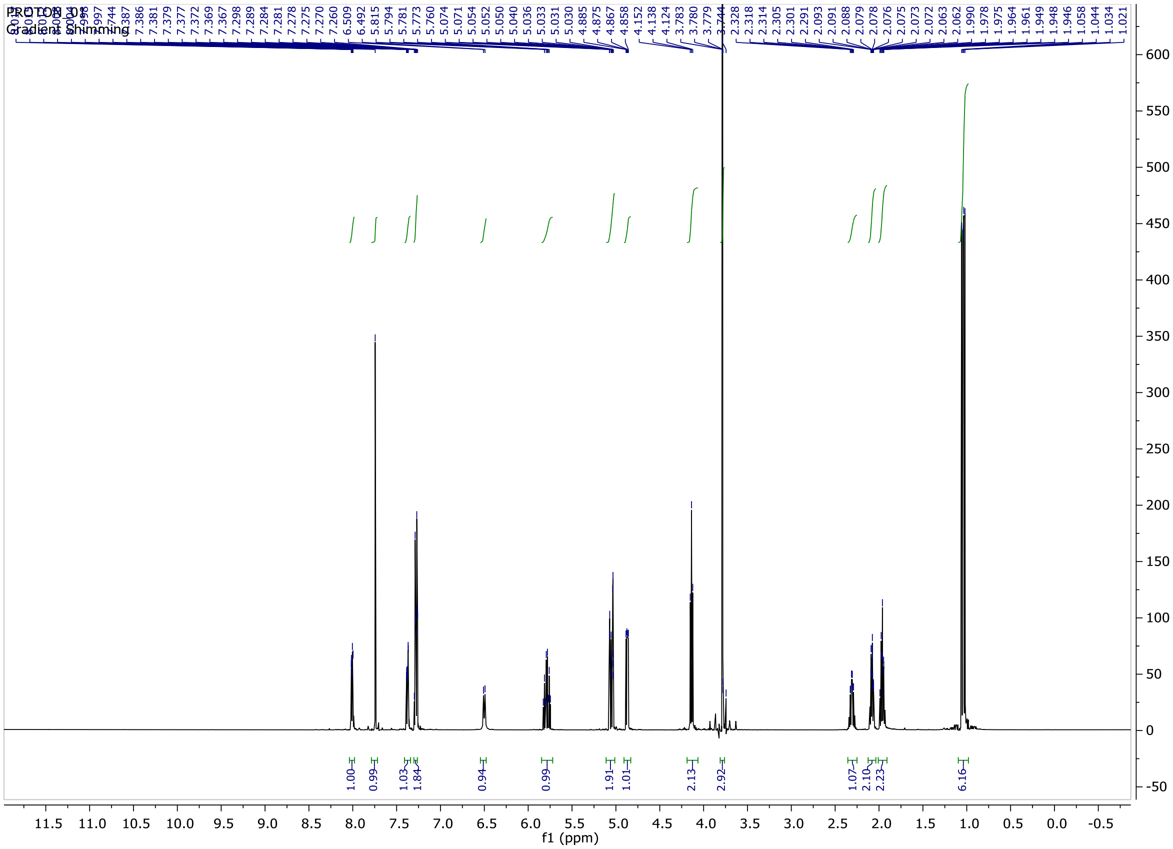

^{13}C-NMR Spectrum

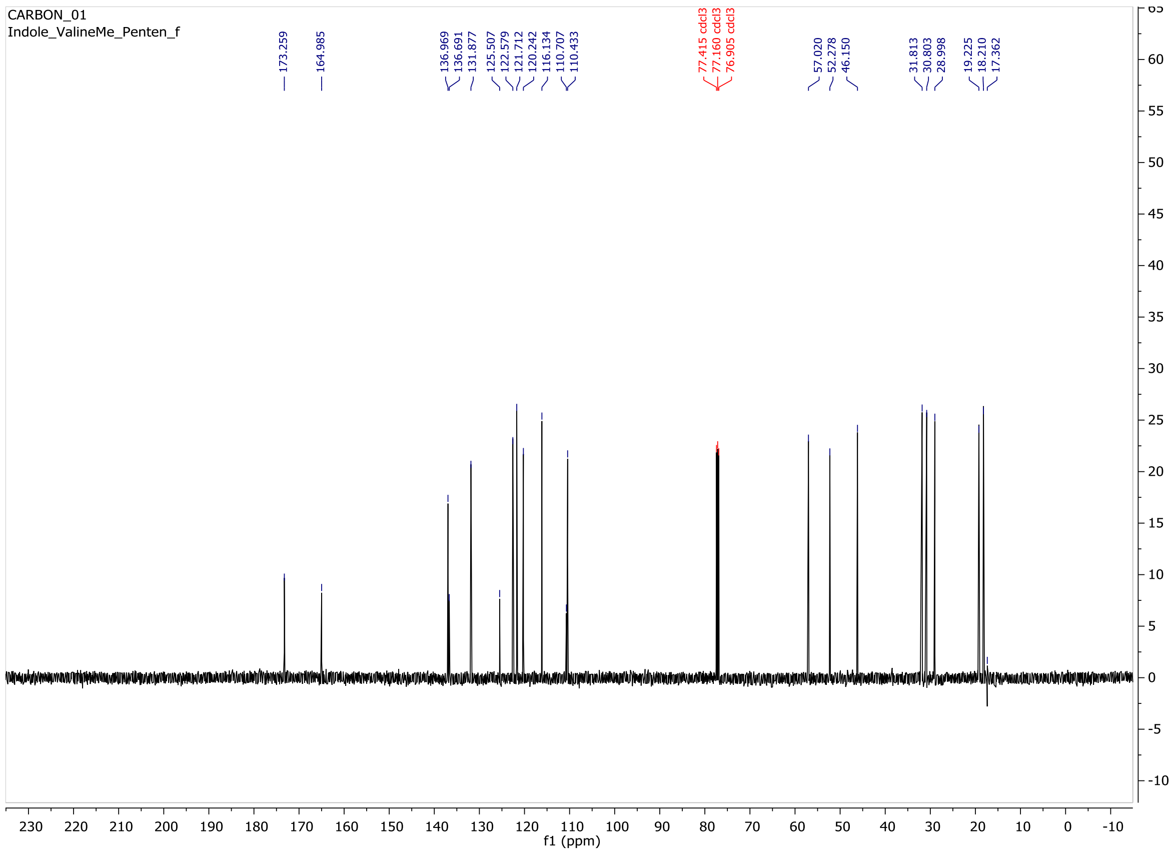

MMB-4en-PICA dihydrodiol (A7)

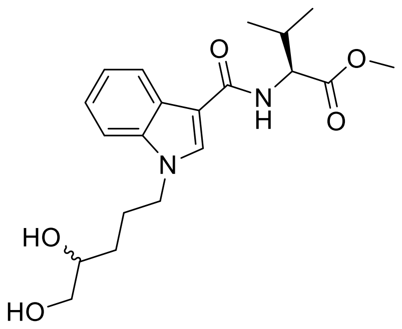

^{1}H-NMR Spectrum

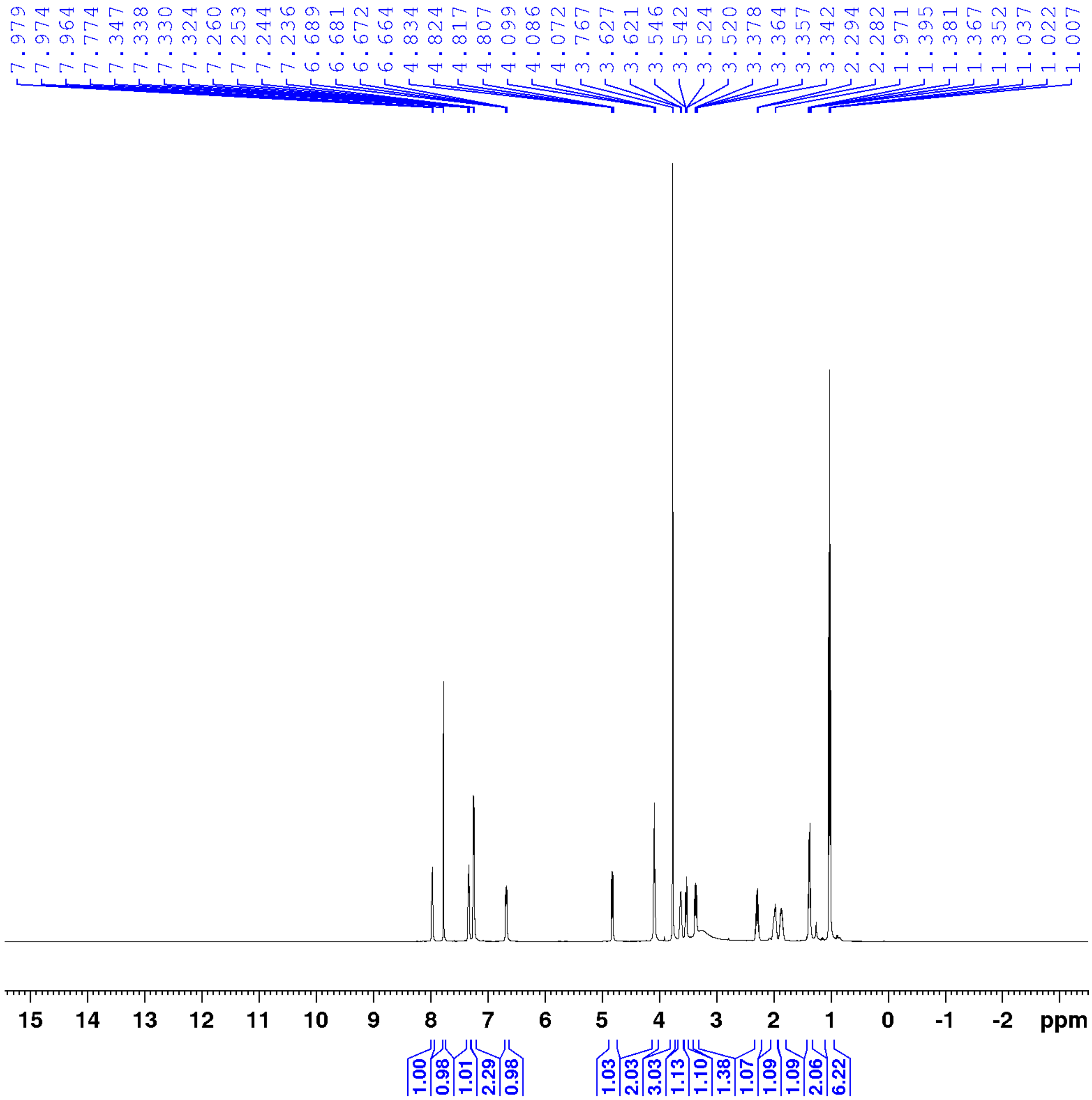

^{13}C-NMR Spectrum

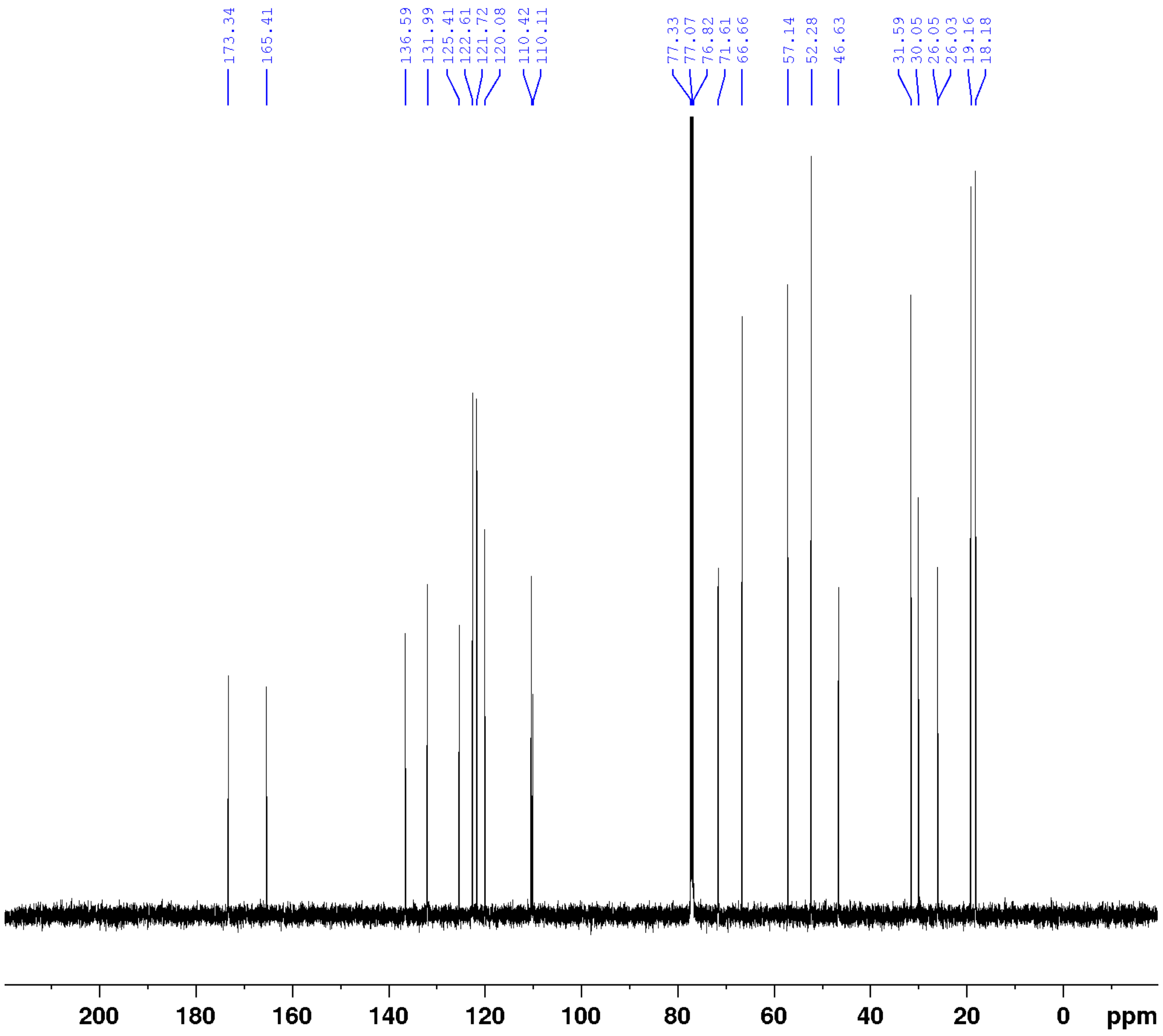

MMB-4en-PINACA

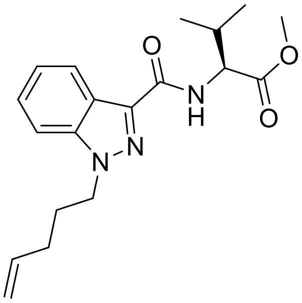

^{1}H-NMR Spectrum

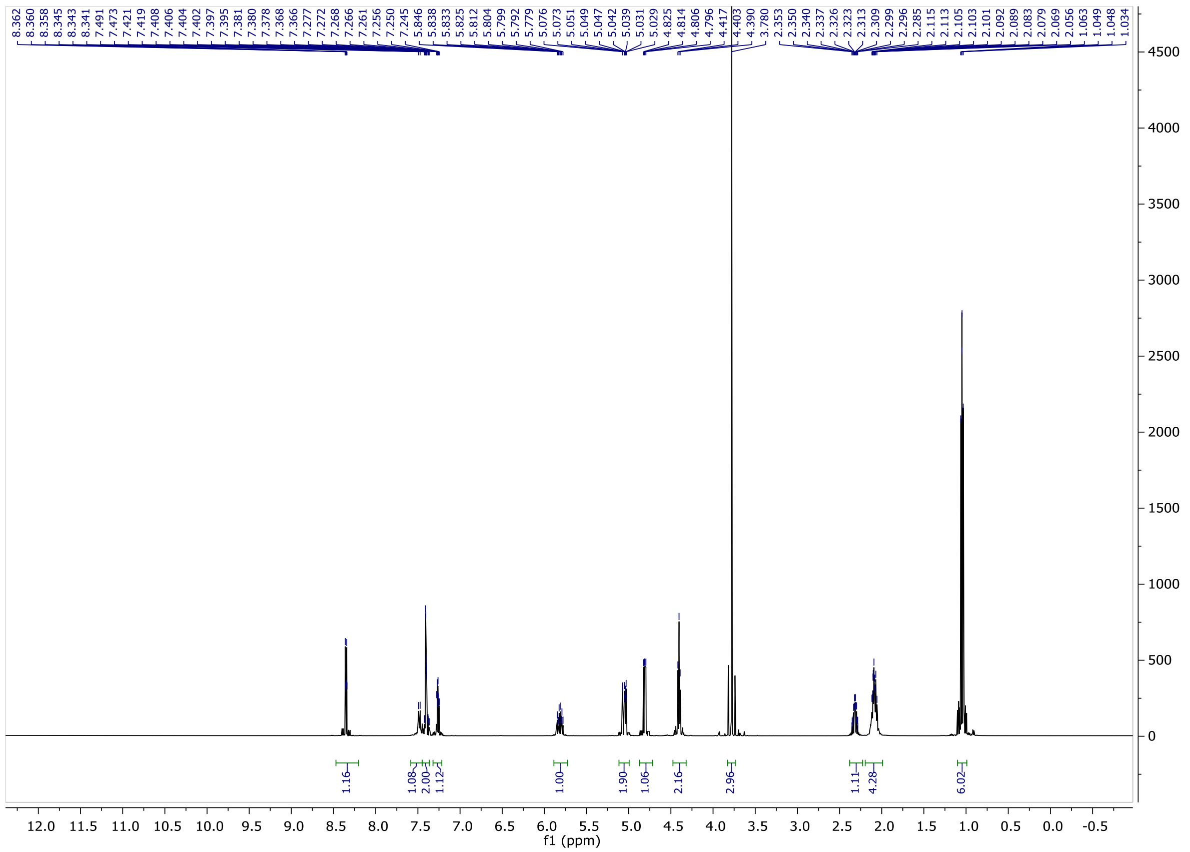

^{13}C-NMR Spectrum

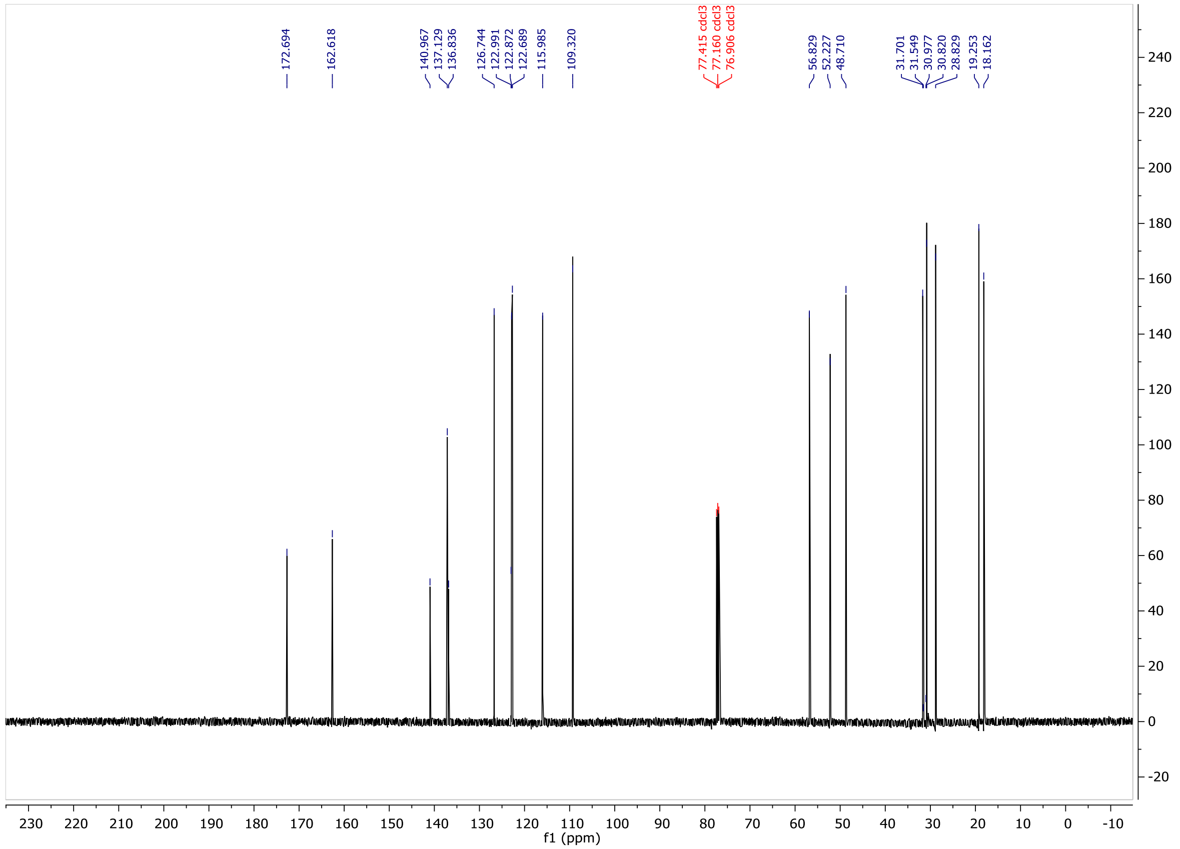

MMB-4en-PINACA dihydrodiol (B6)

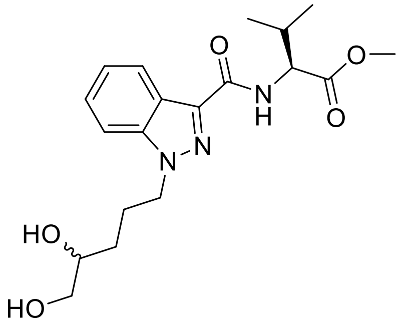

^{1}H-NMR Spectrum

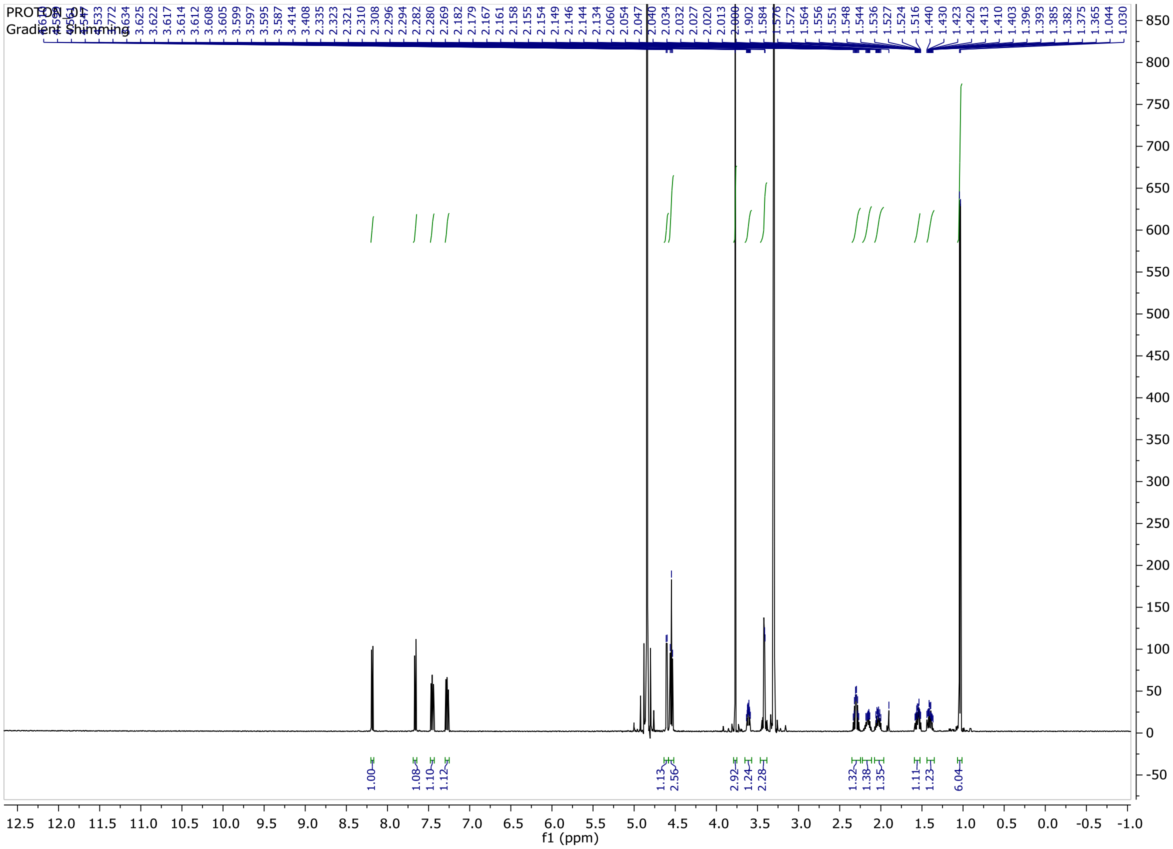

^{13}C-NMR Spectrum

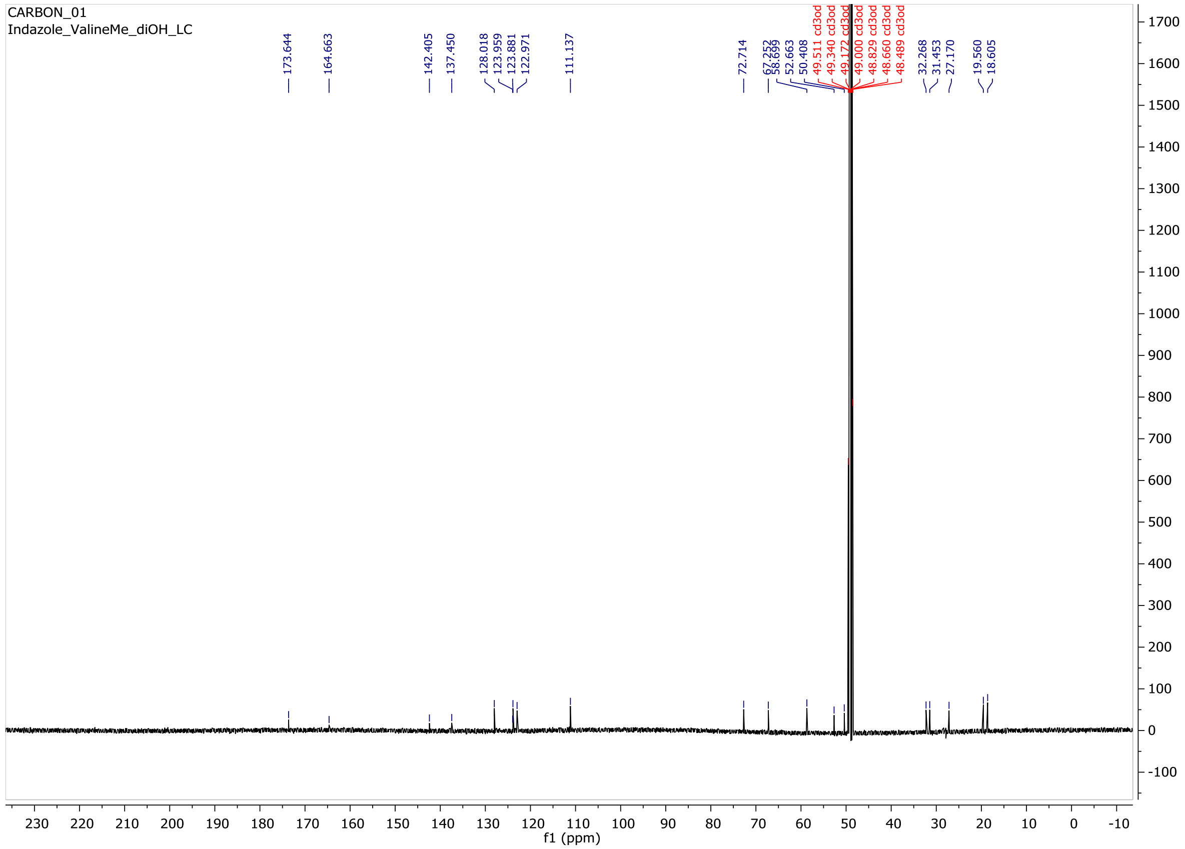

EMB-4en-PICA

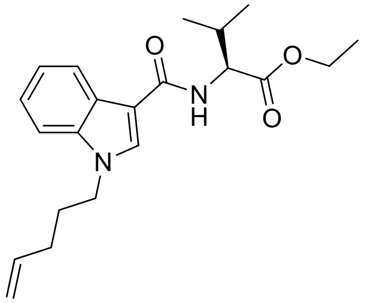

^{1}H-NMR Spectrum

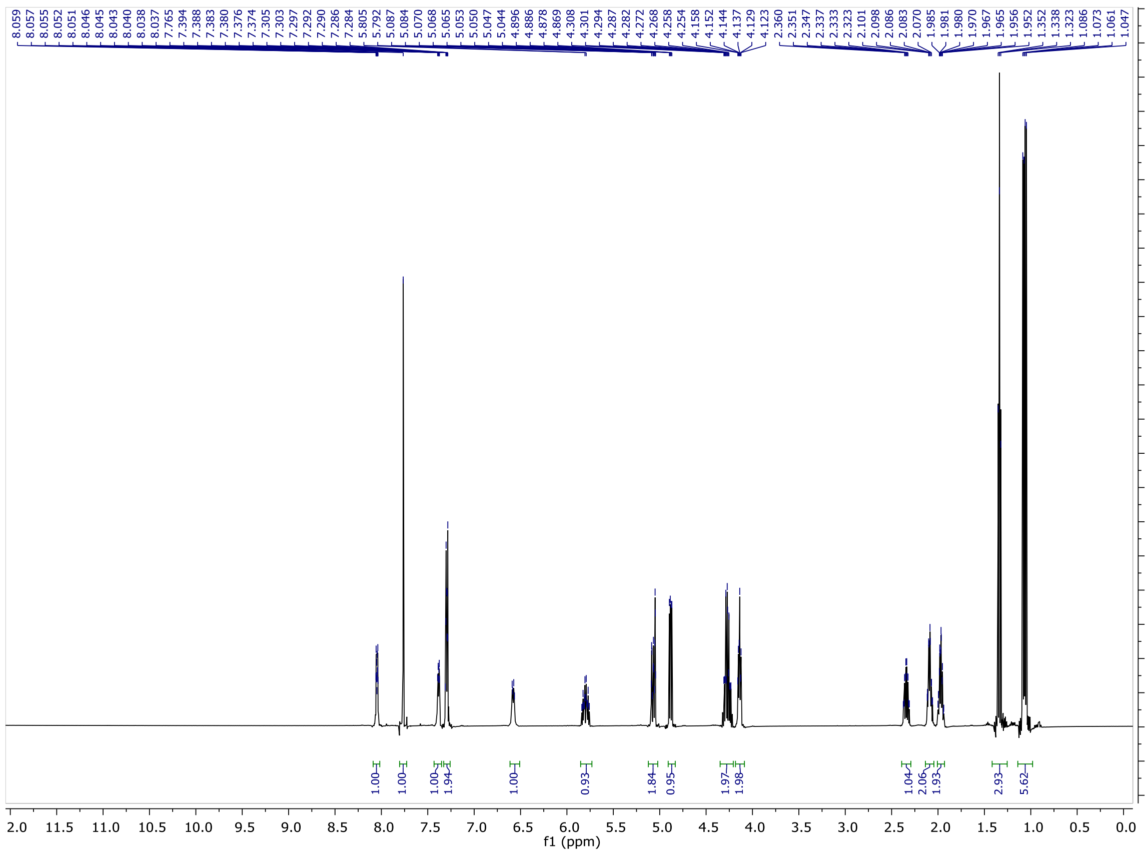

^{13}C-NMR Spectrum

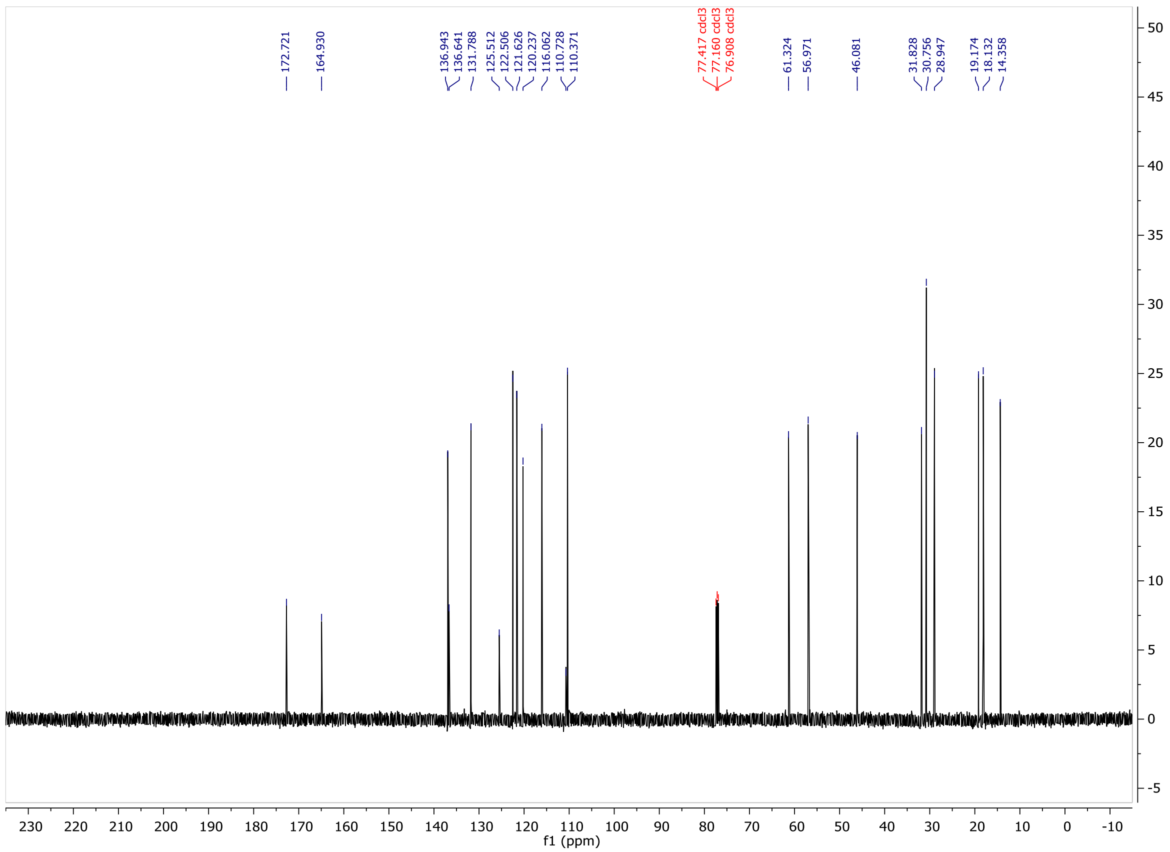

EMB-4en-PICA dihydrodiol (C7)

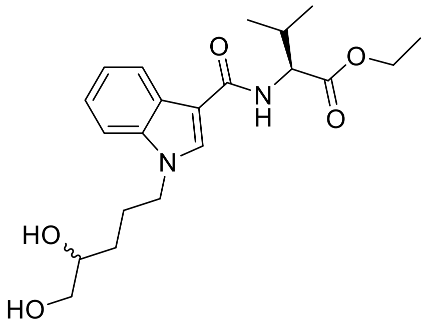

^{1}H-NMR Spectrum

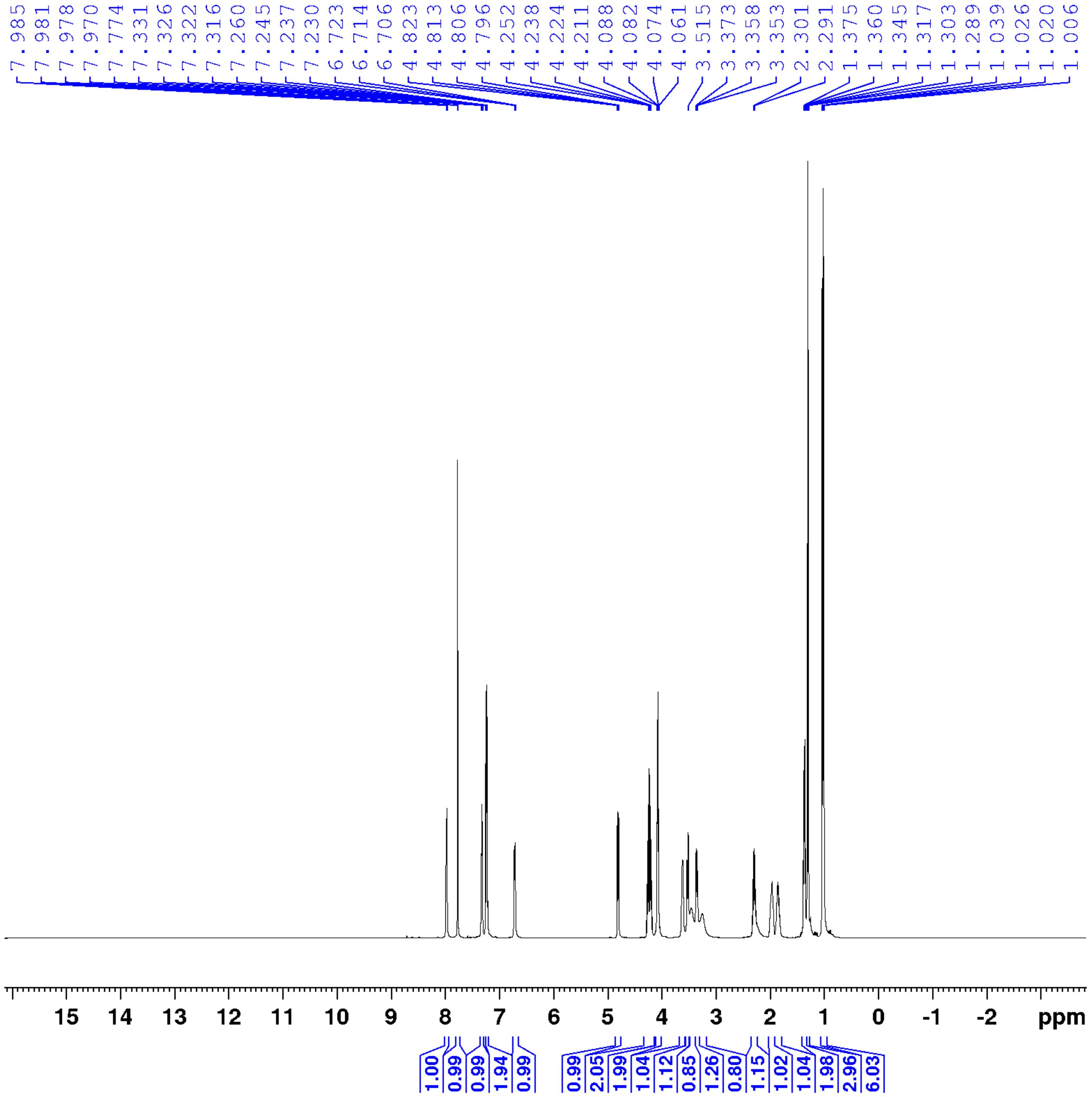

^{13}C-NMR Spectrum

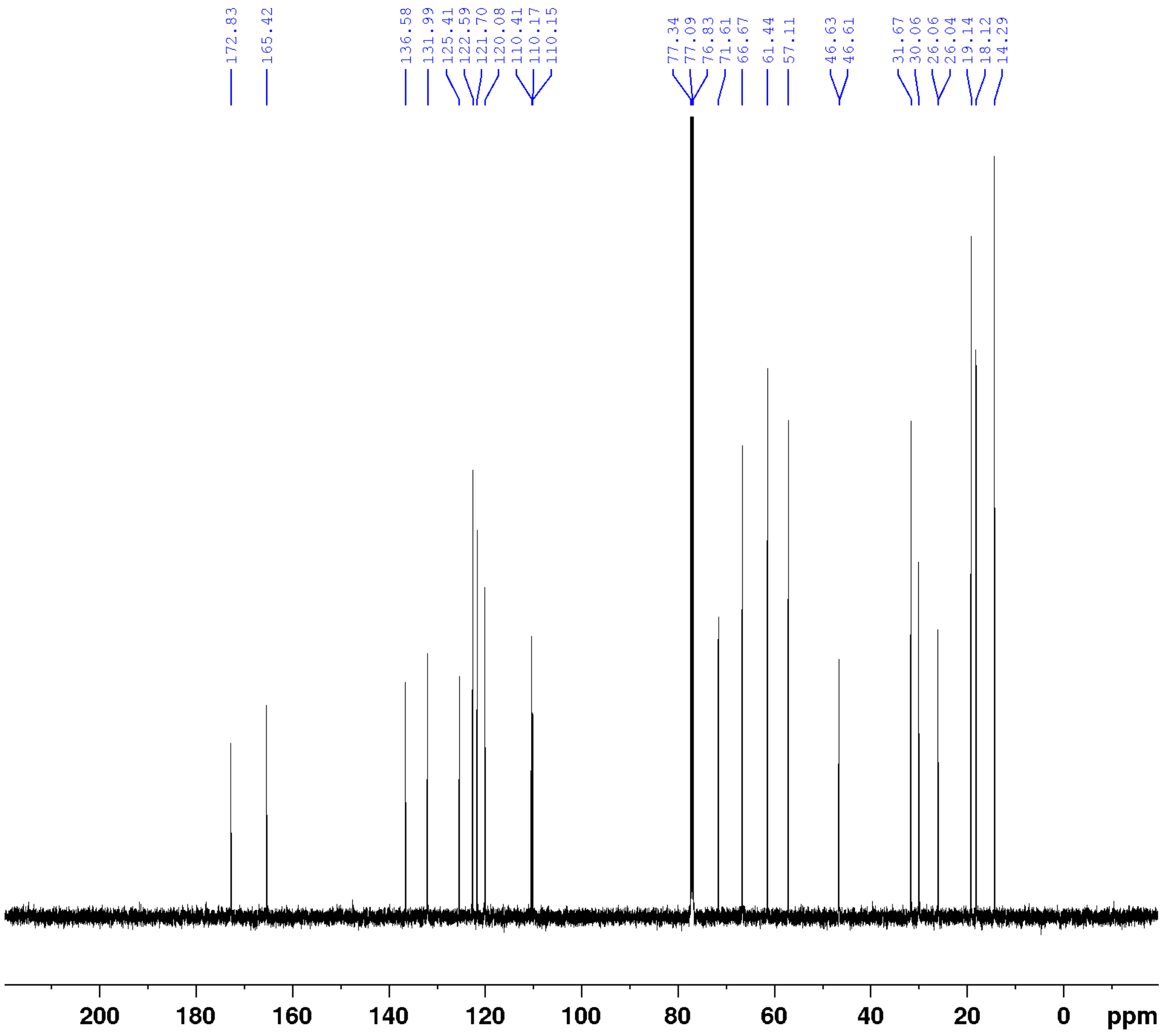

EMB-4en-PINACA

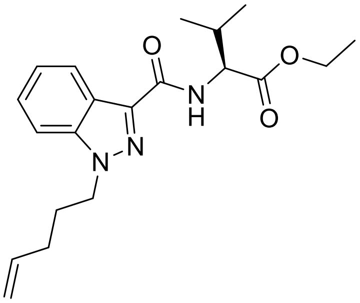

^{1}H-NMR Spectrum

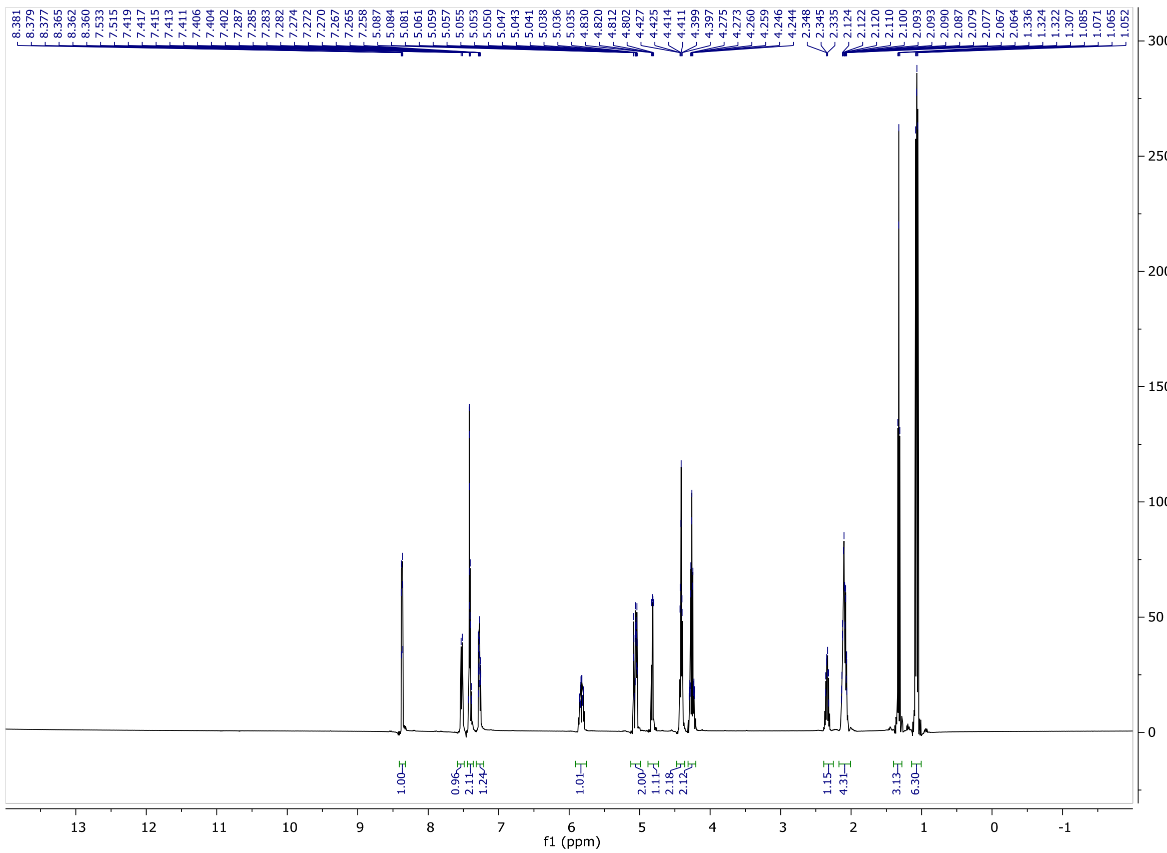

^{13}C-NMR Spectrum

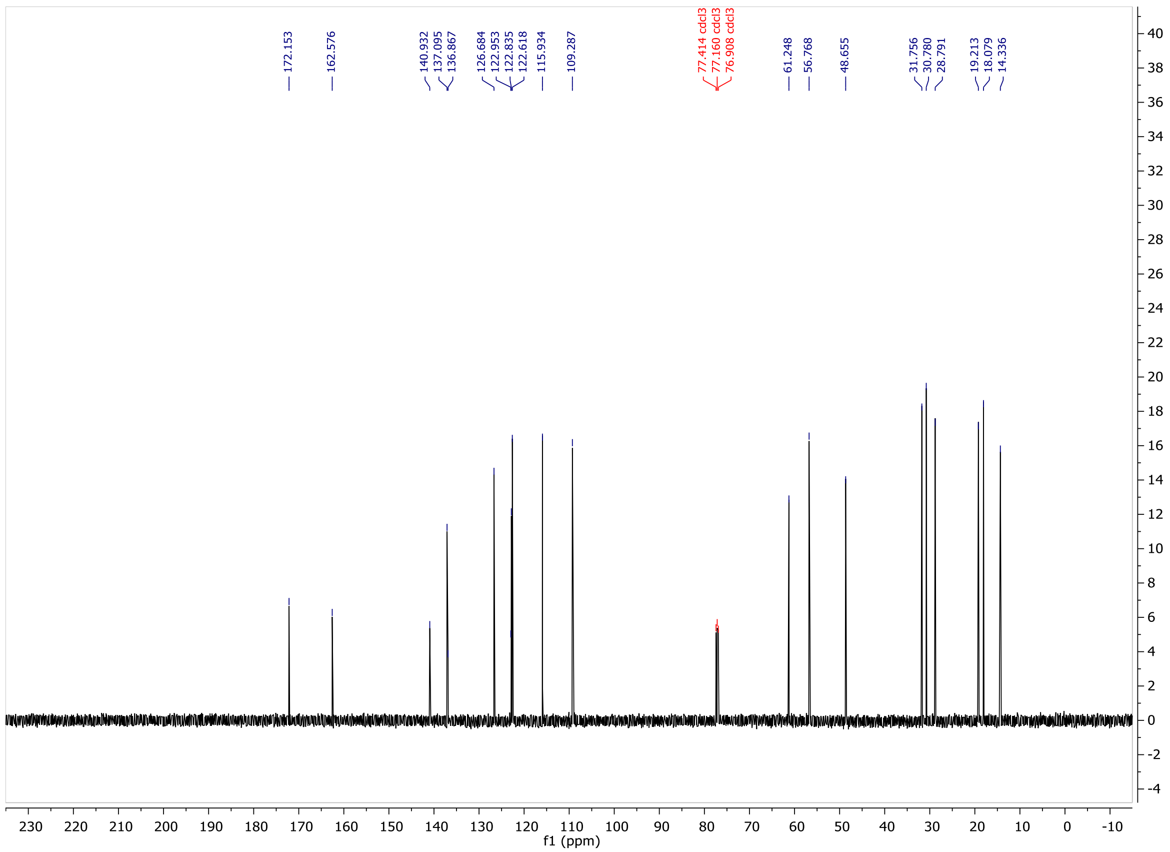

EMB-4en-PINACA dihydrodiol

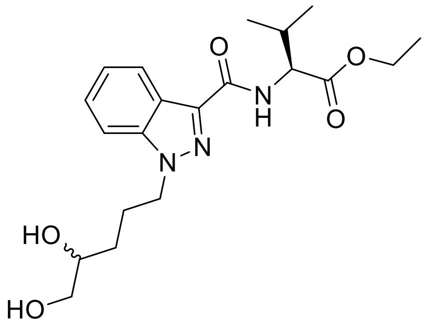

^{1}H-NMR Spectrum

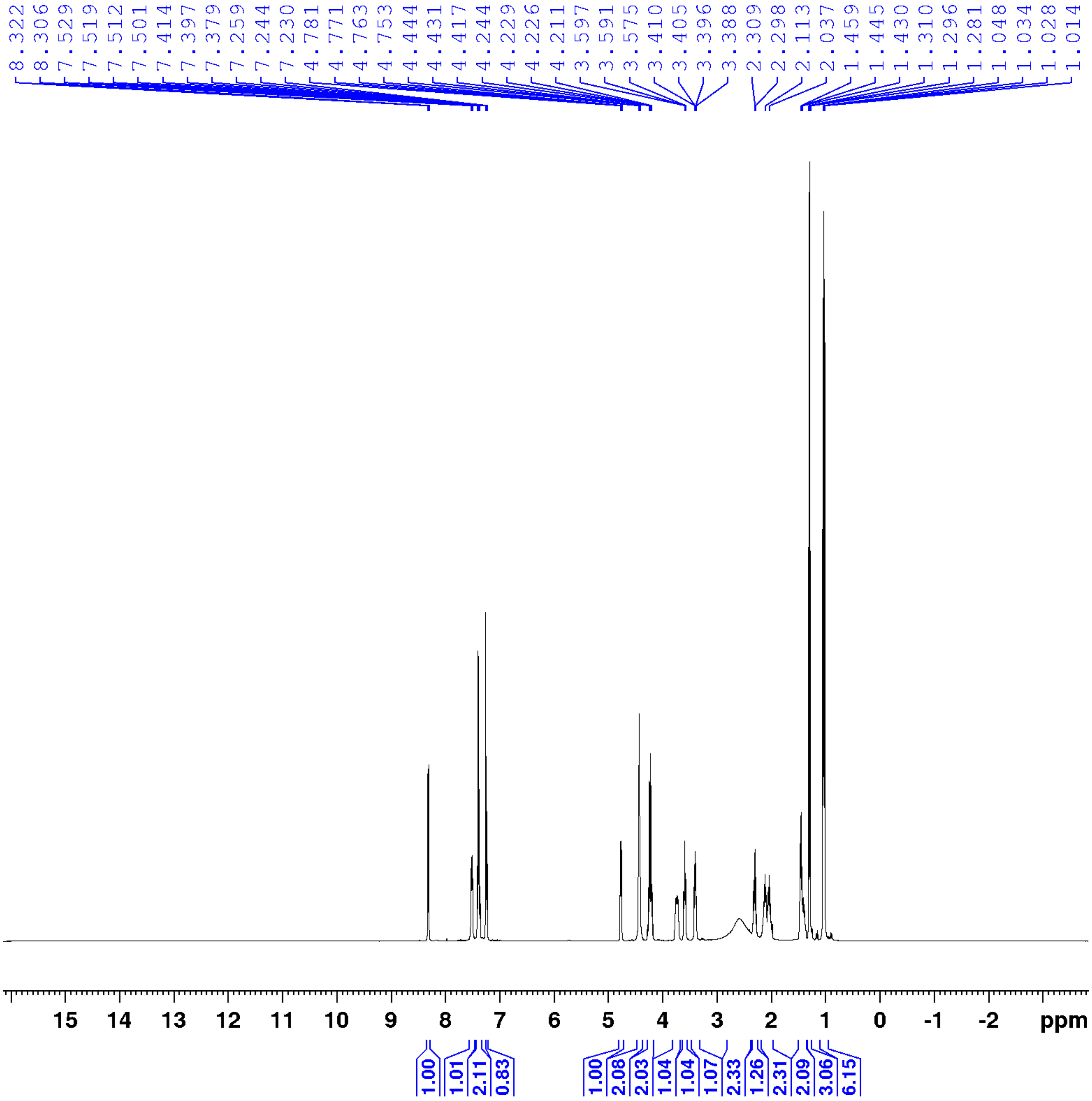

13C-NMR Spectrum

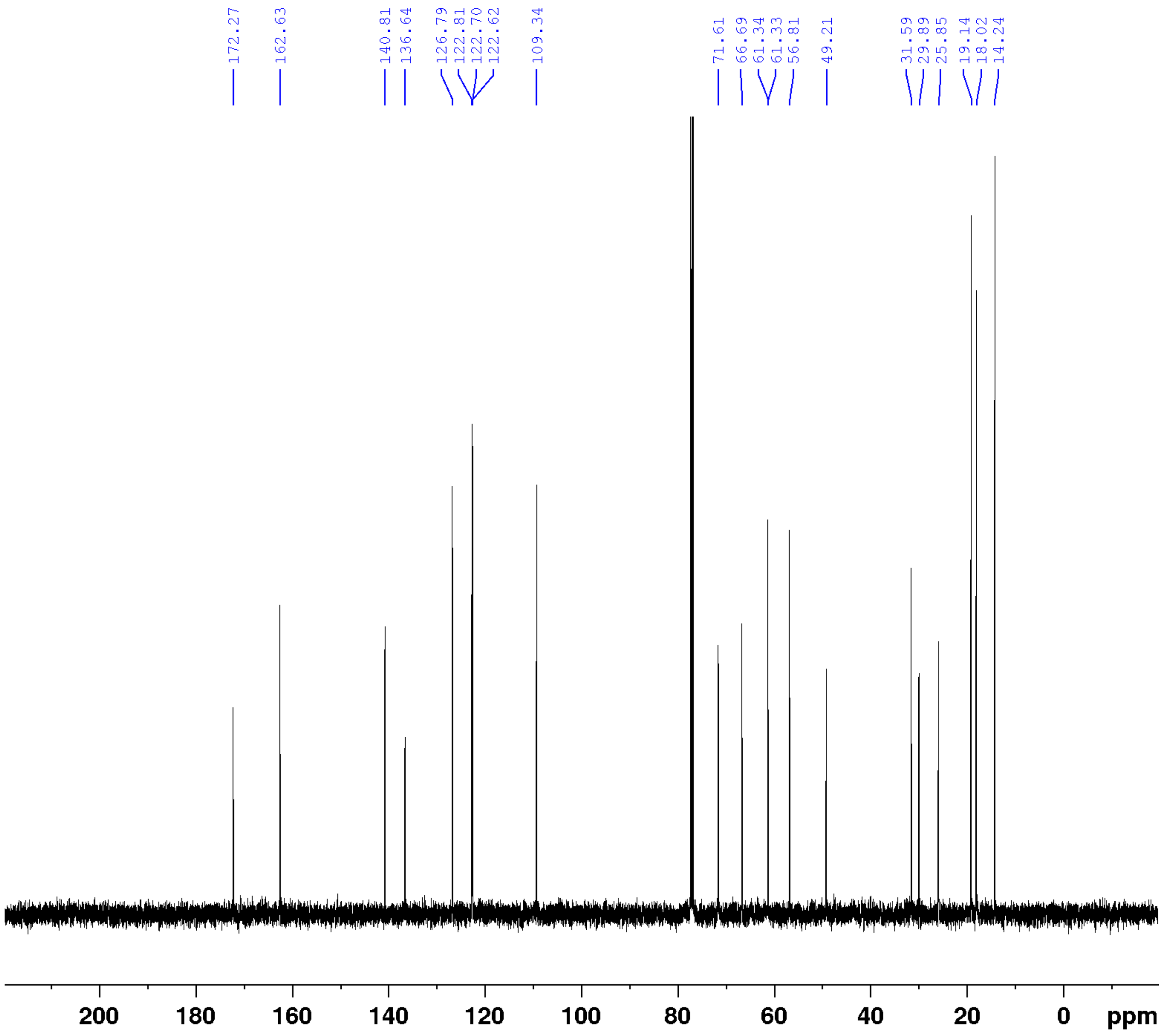

MDMB-4en-PICA

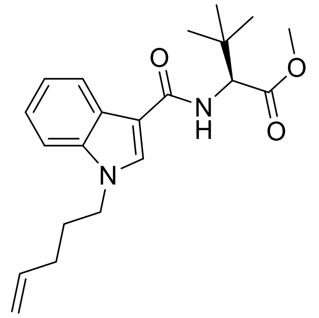

^{1}H-NMR Spectrum

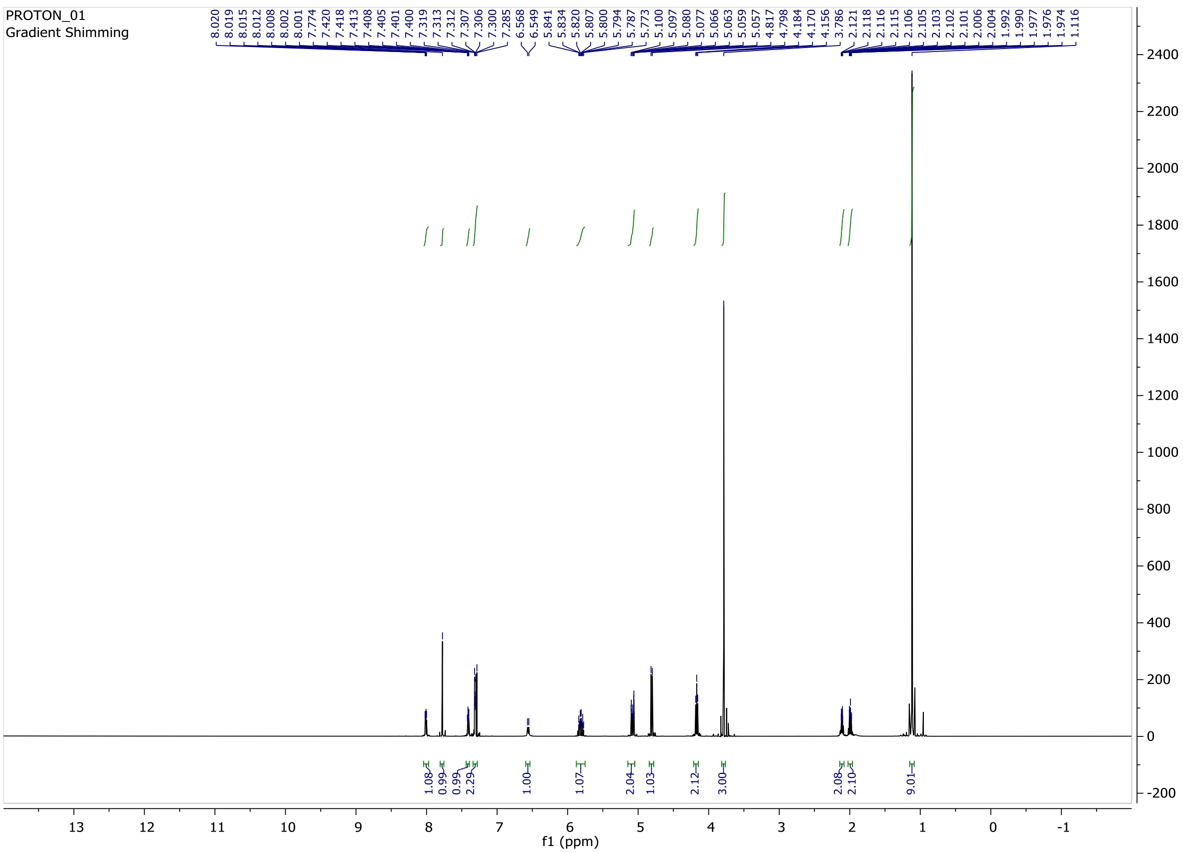

^{13}C-NMR Spectrum

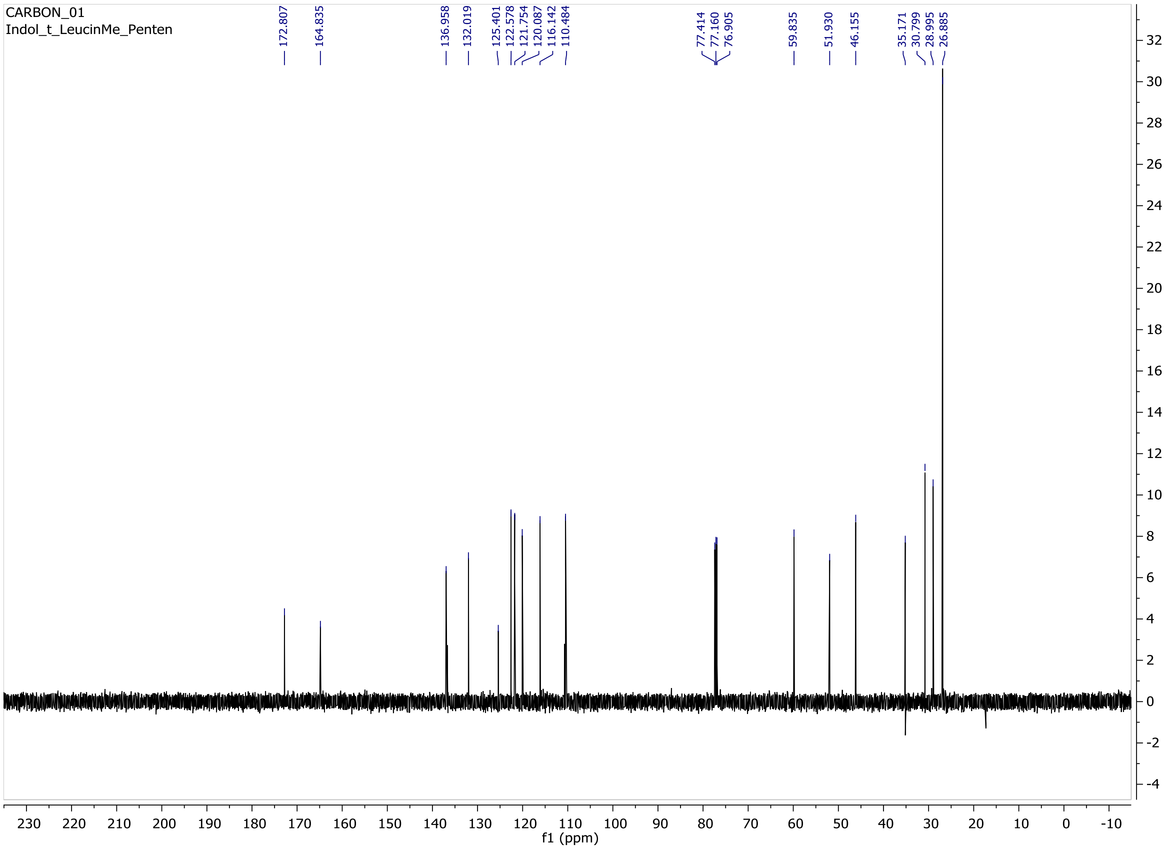

MDMB-4en-PICA dihydrodiol (E2)

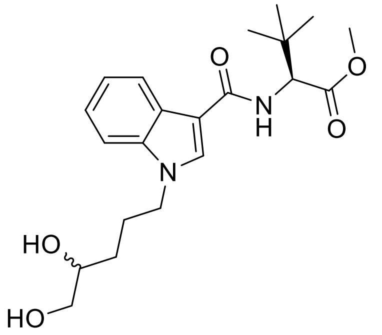

^{1}H-NMR Spectrum

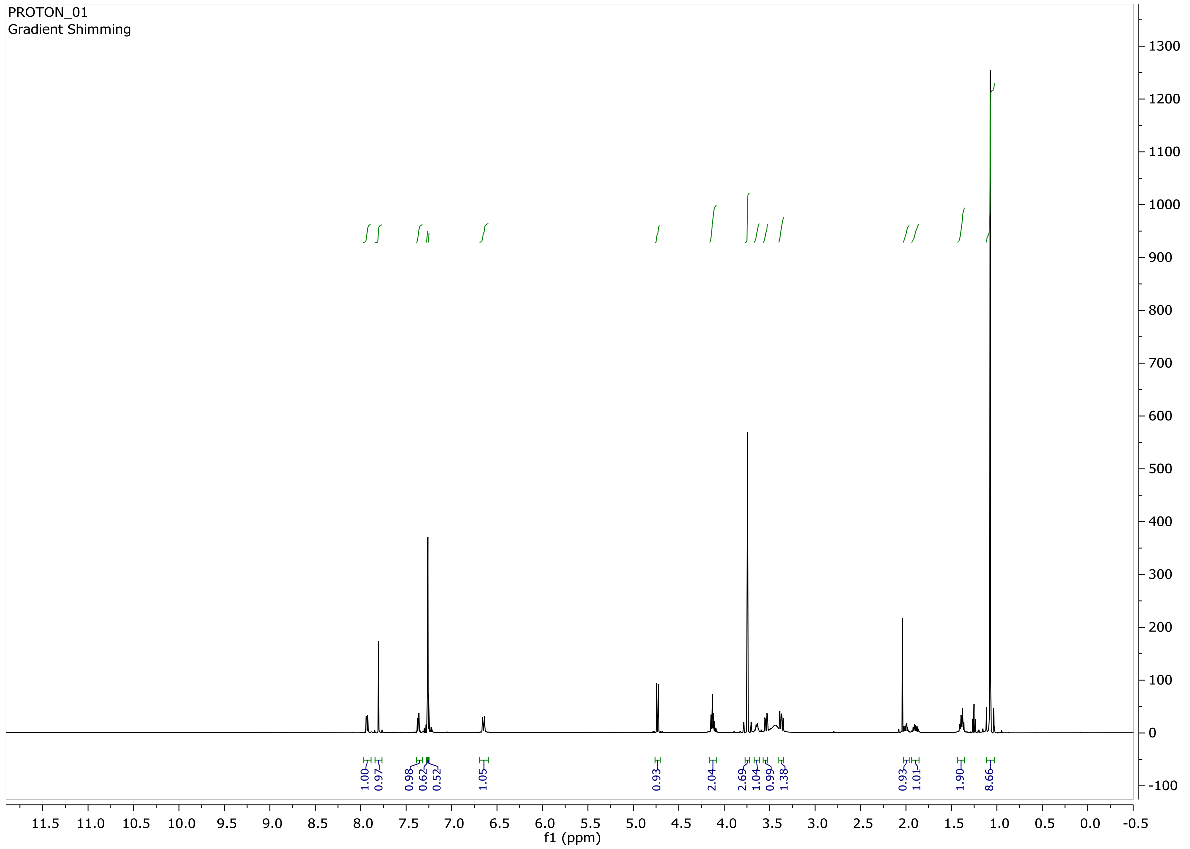

^{13}C-NMR Spectrum

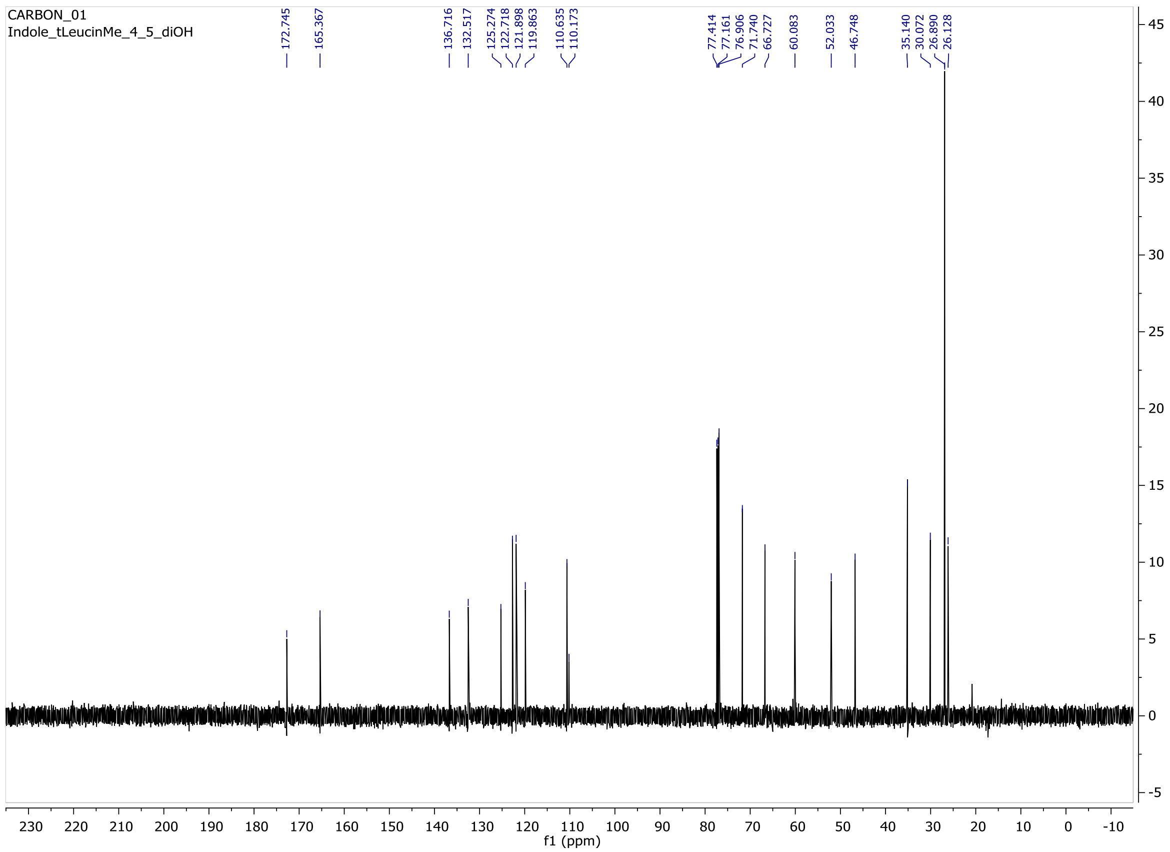

MDMB-4en-PINACA

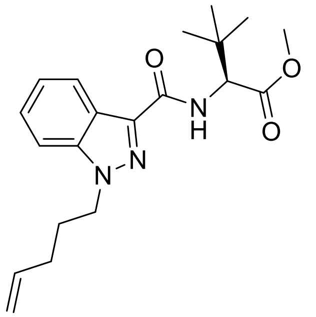

^{1}H-NMR Spectrum

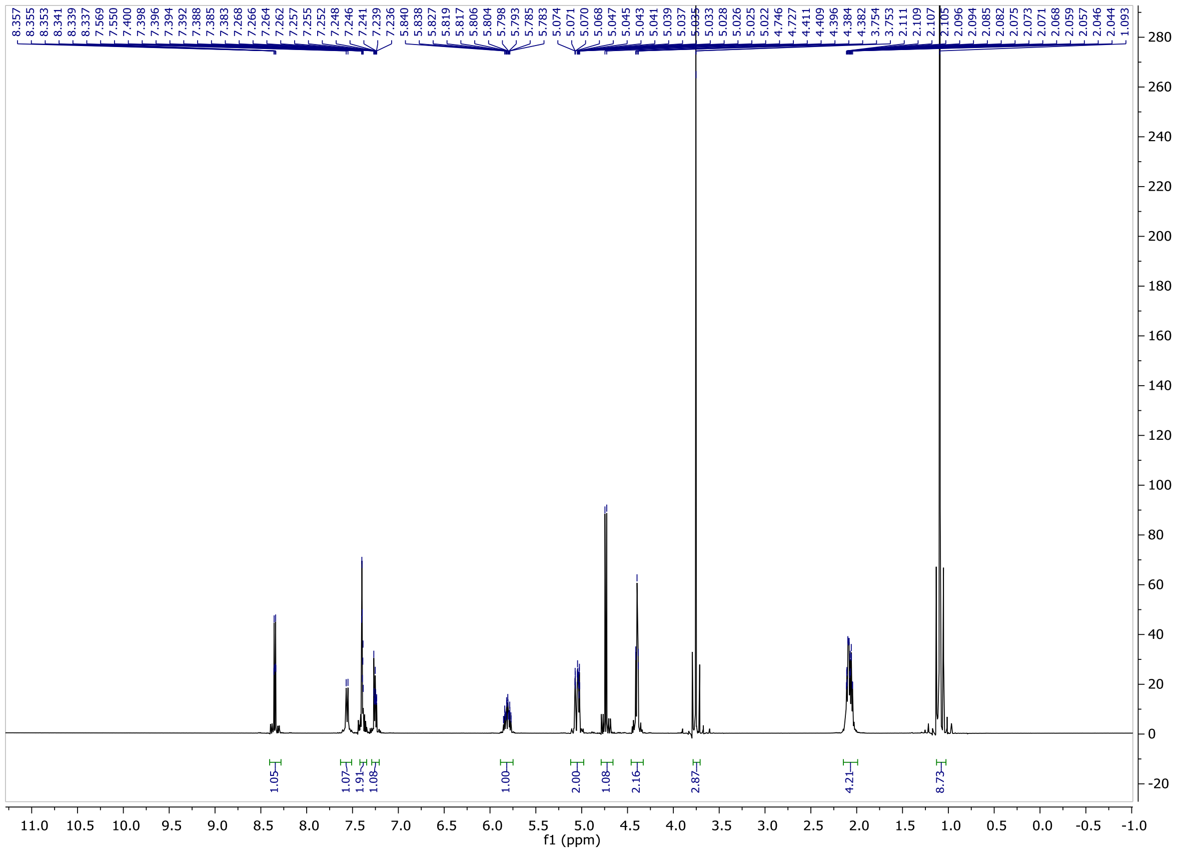

^{13}C-NMR Spectrum

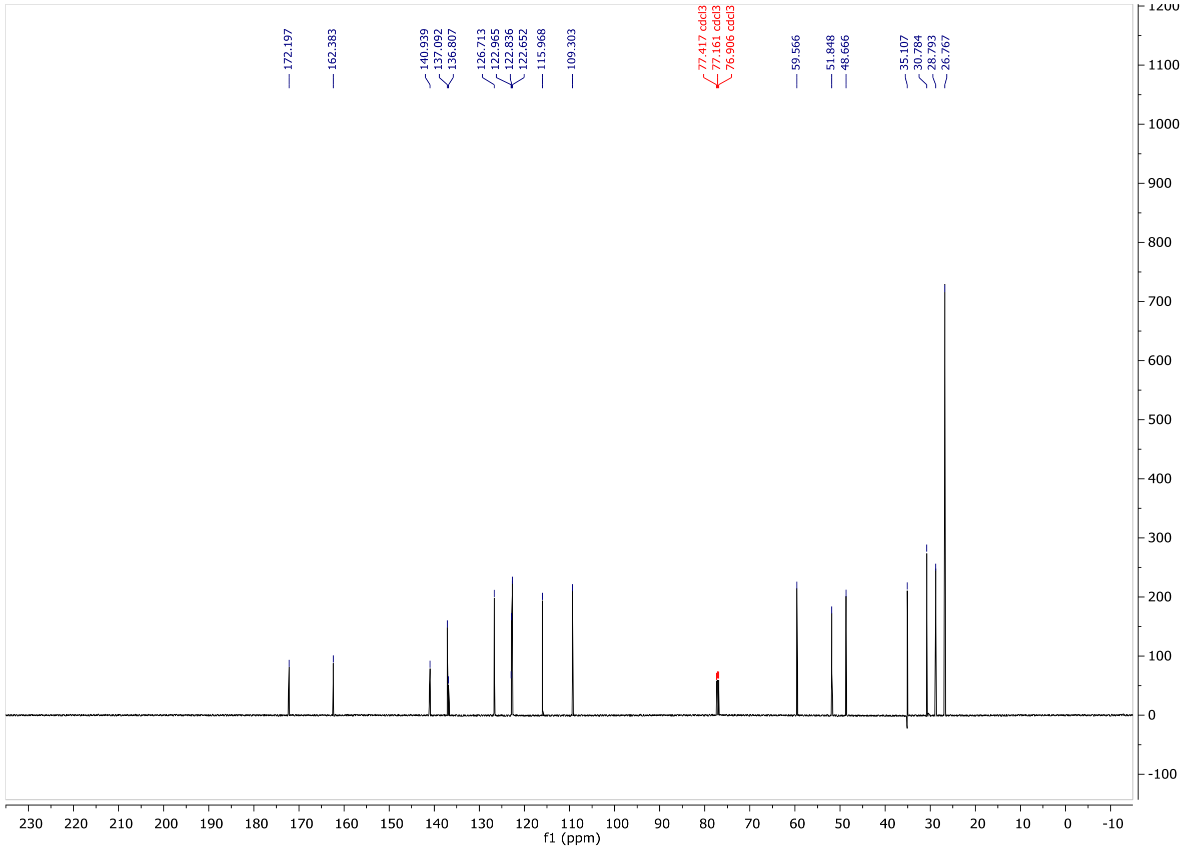

MDMB-4en-PINACA dihydrodiol (F2)

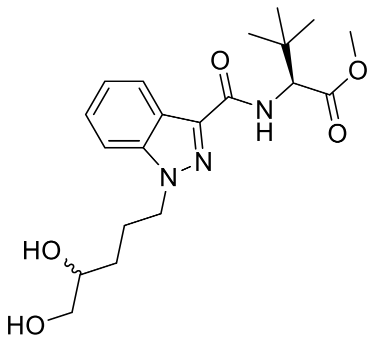

^{1}H-NMR Spectrum

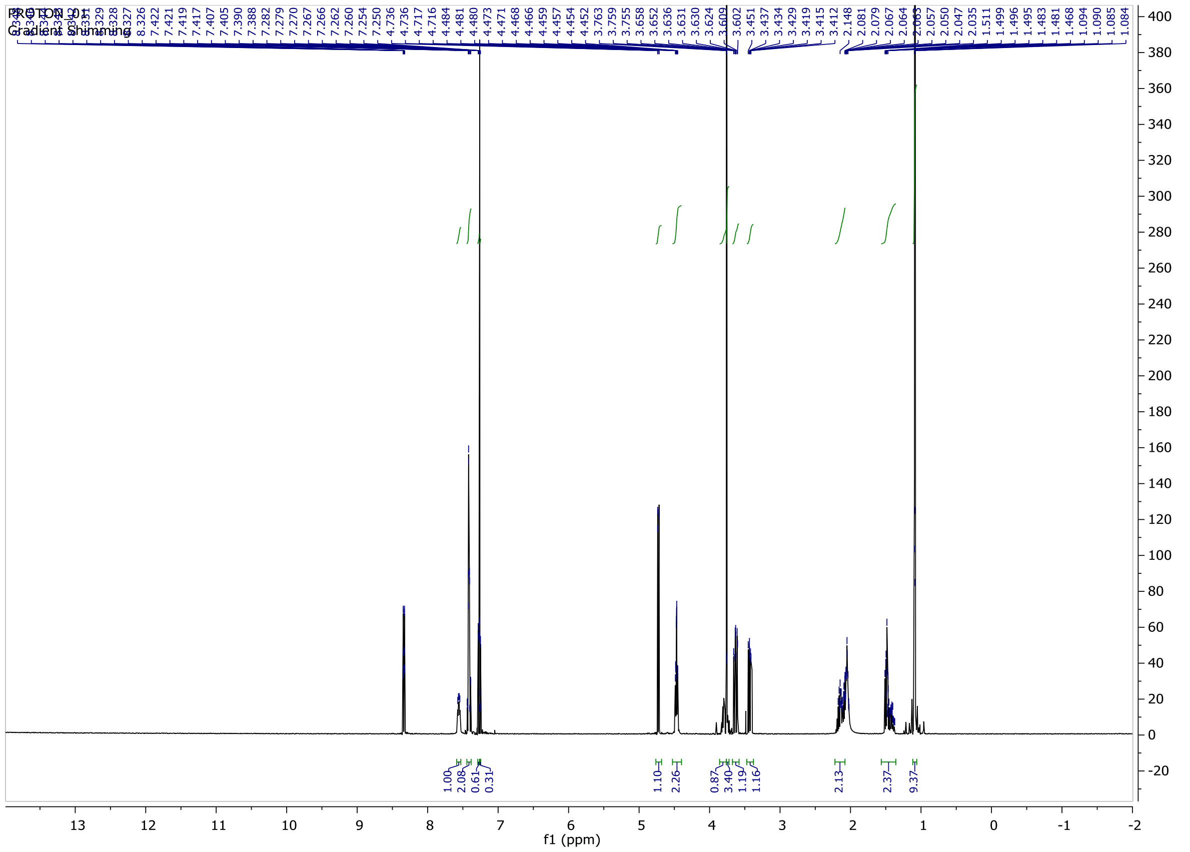

^{13}C-NMR Spectrum

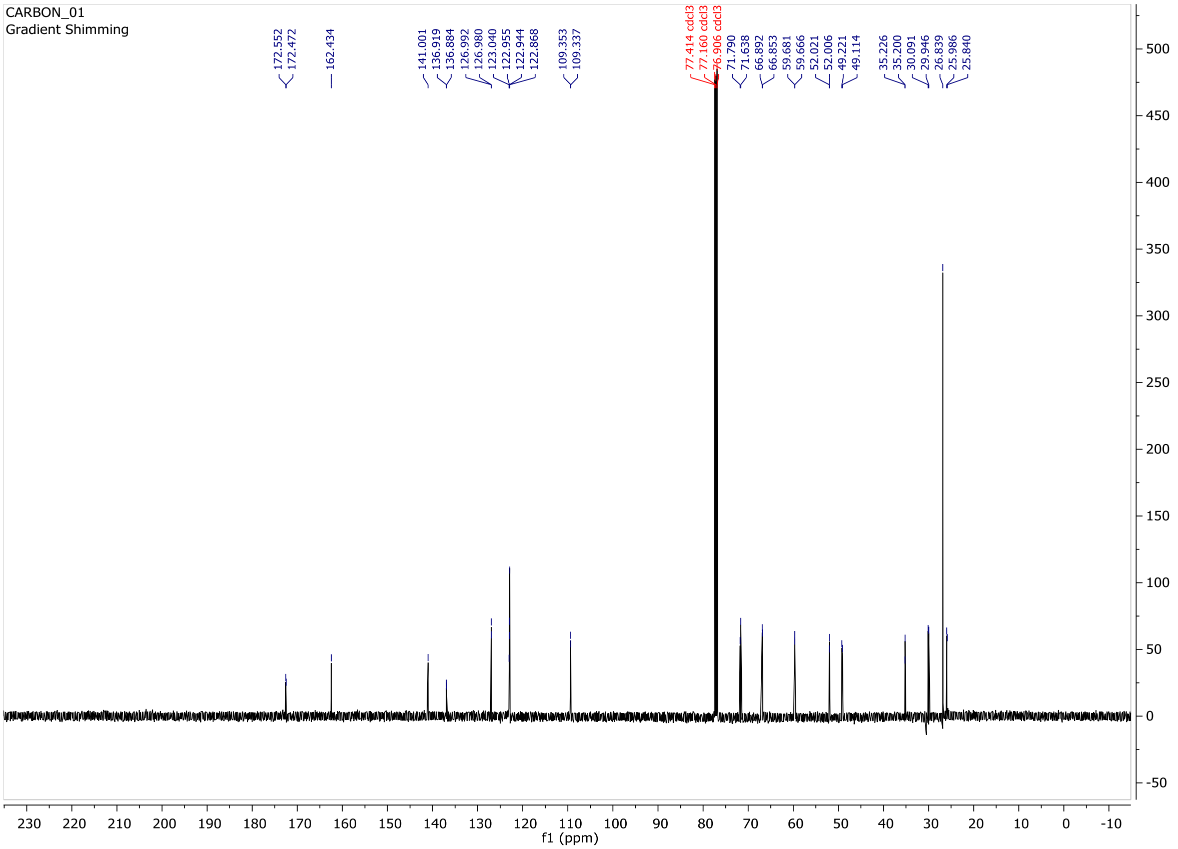

AB-4en-PICA

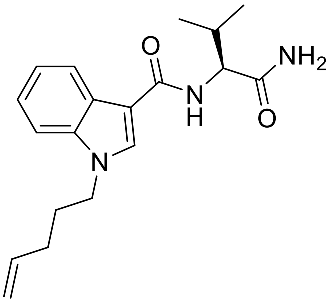

^{1}H-NMR Spectrum

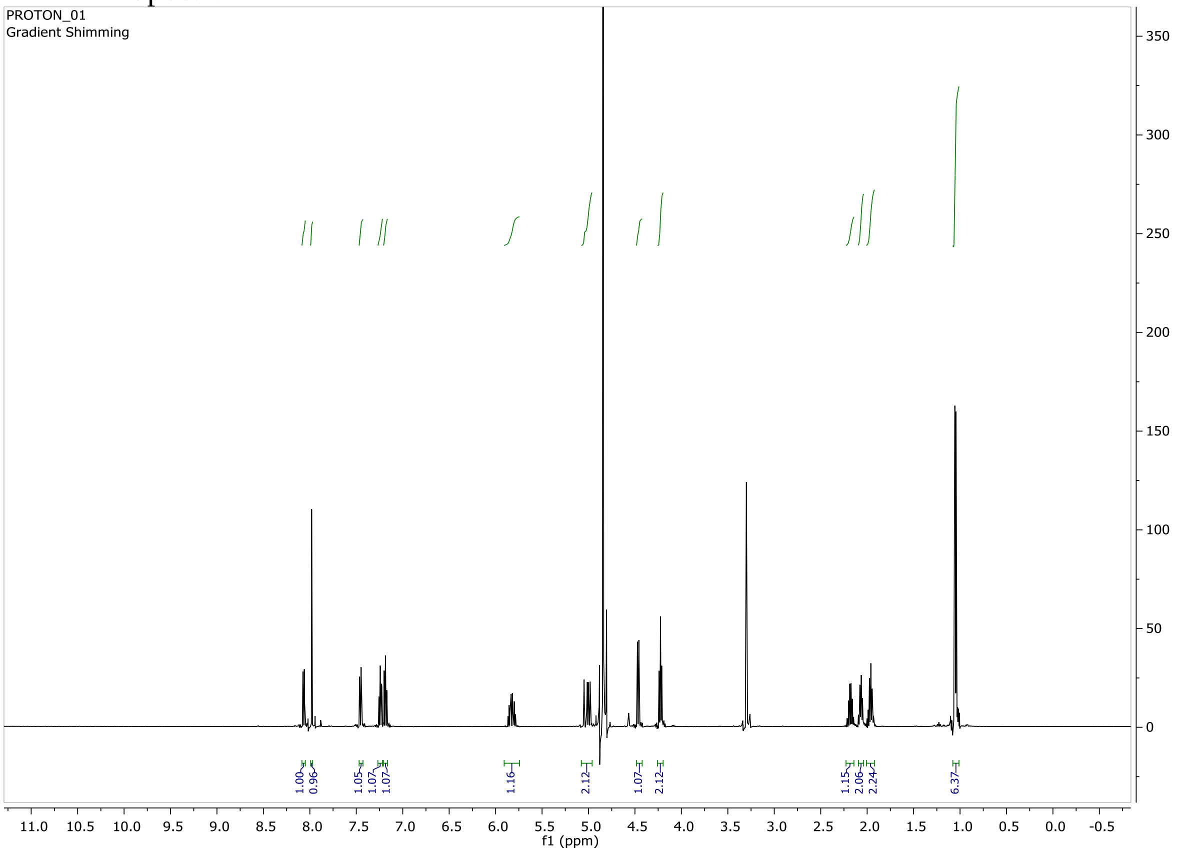

^{13}C-NMR Spectrum

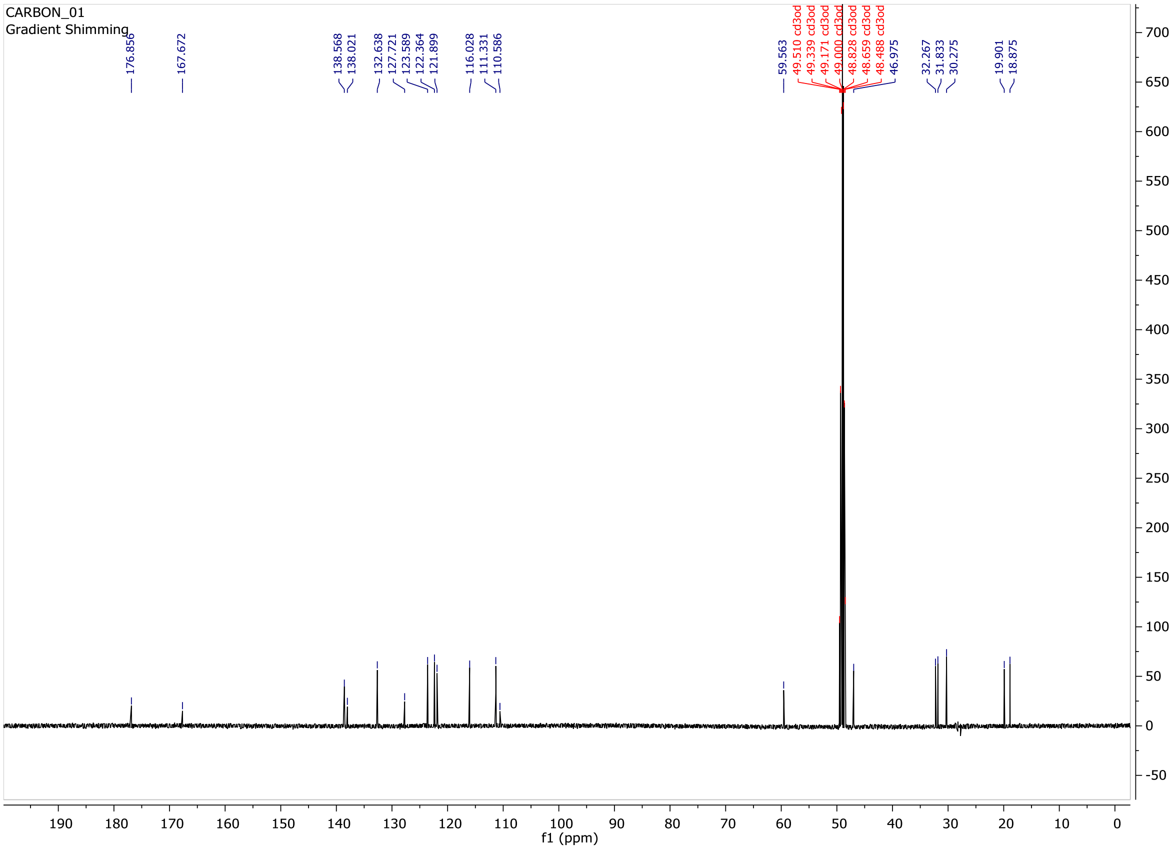

AB-4en-PICA dihydrodiol (G4)

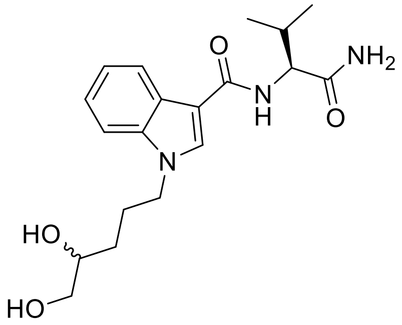

^{1}H-NMR Spectrum

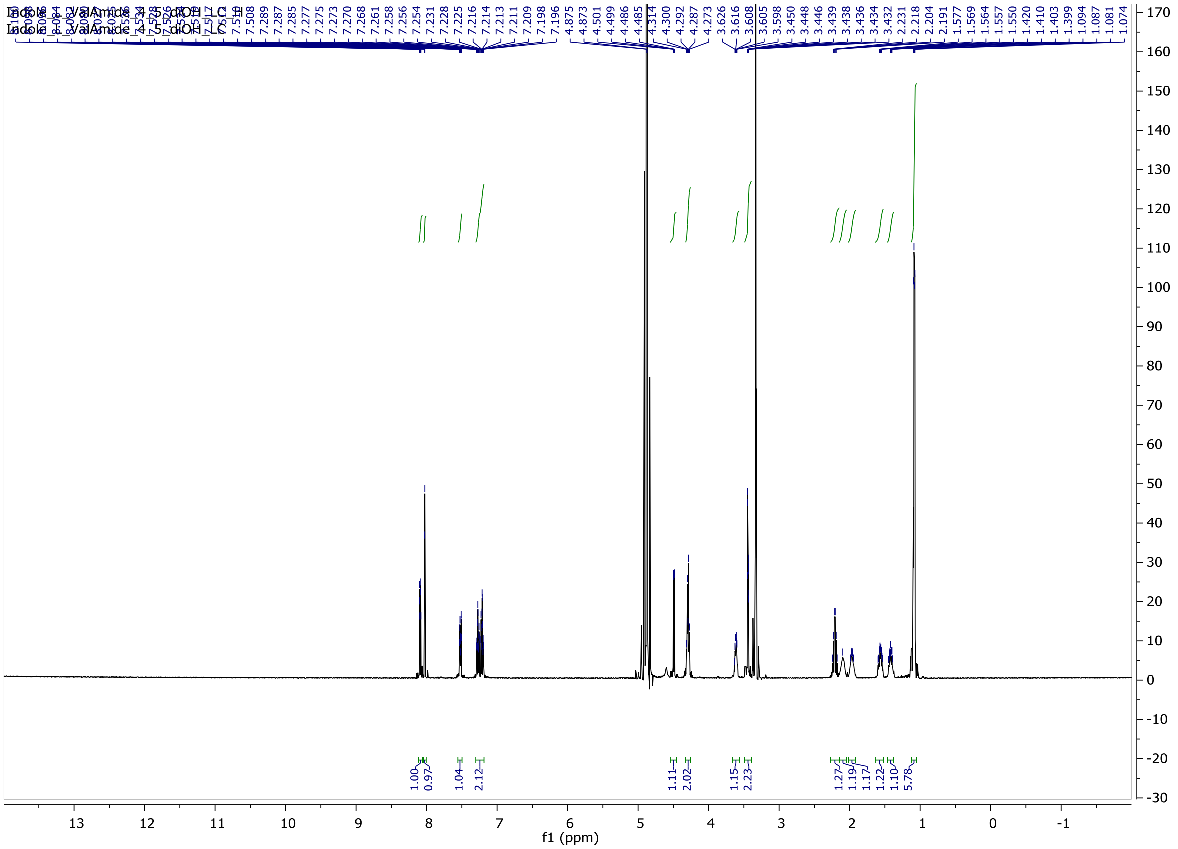

^{13}C-NMR Spectrum

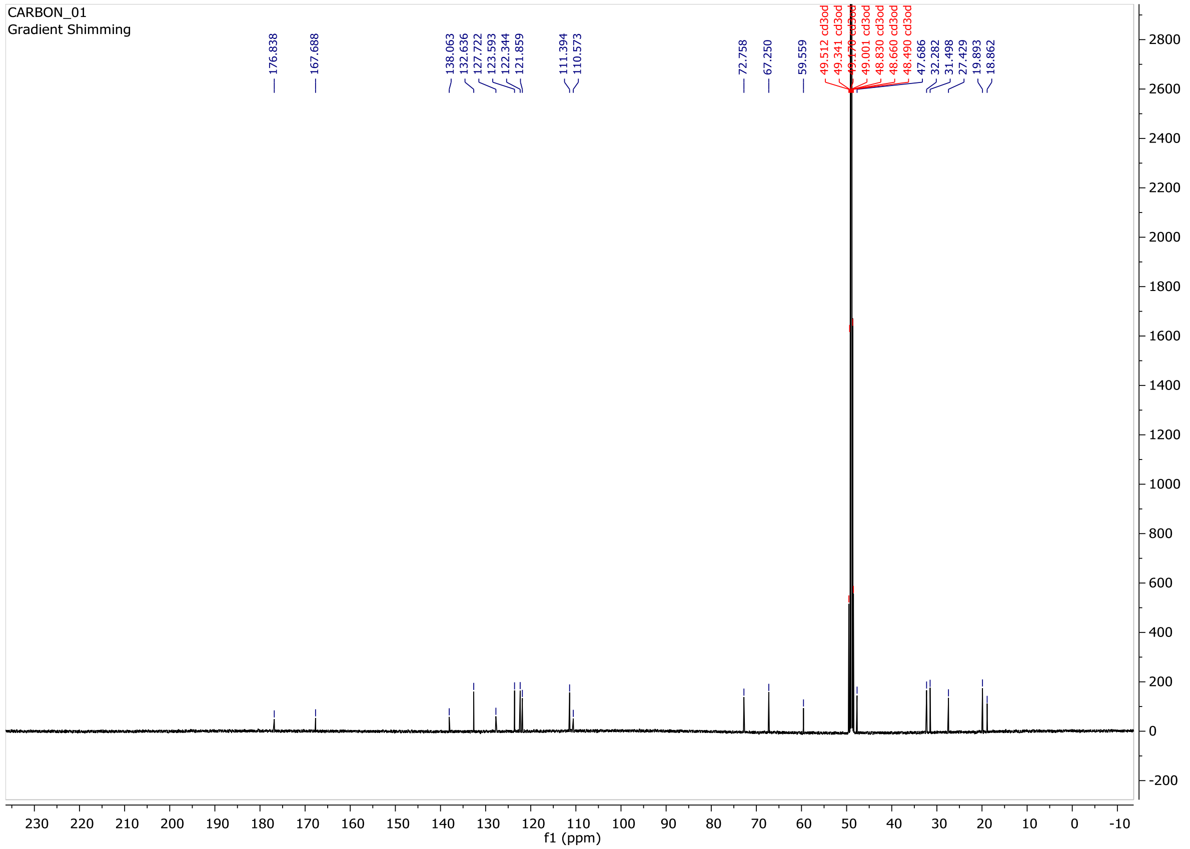

AB-4en-PINACA

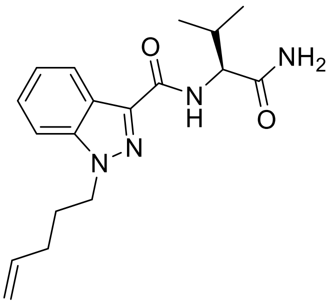

^{1}H-NMR Spectrum

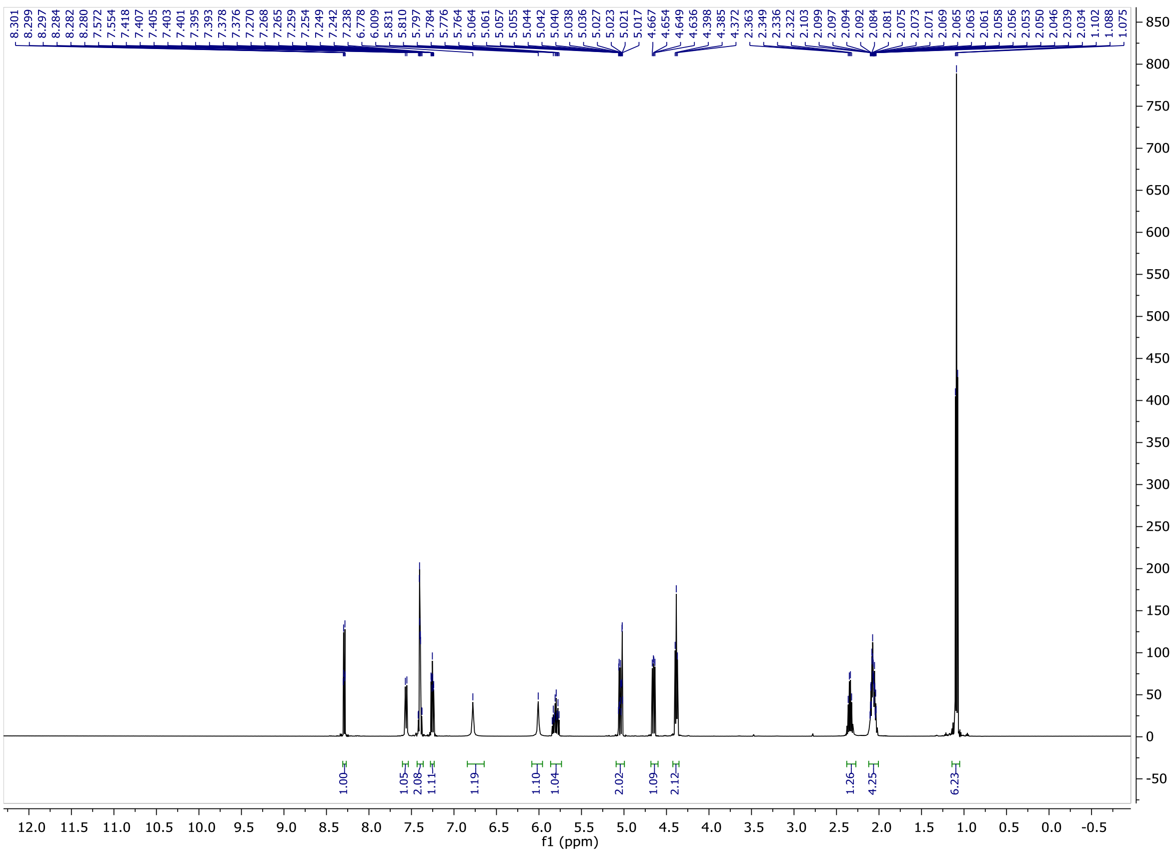

^{13}C-NMR Spectrum

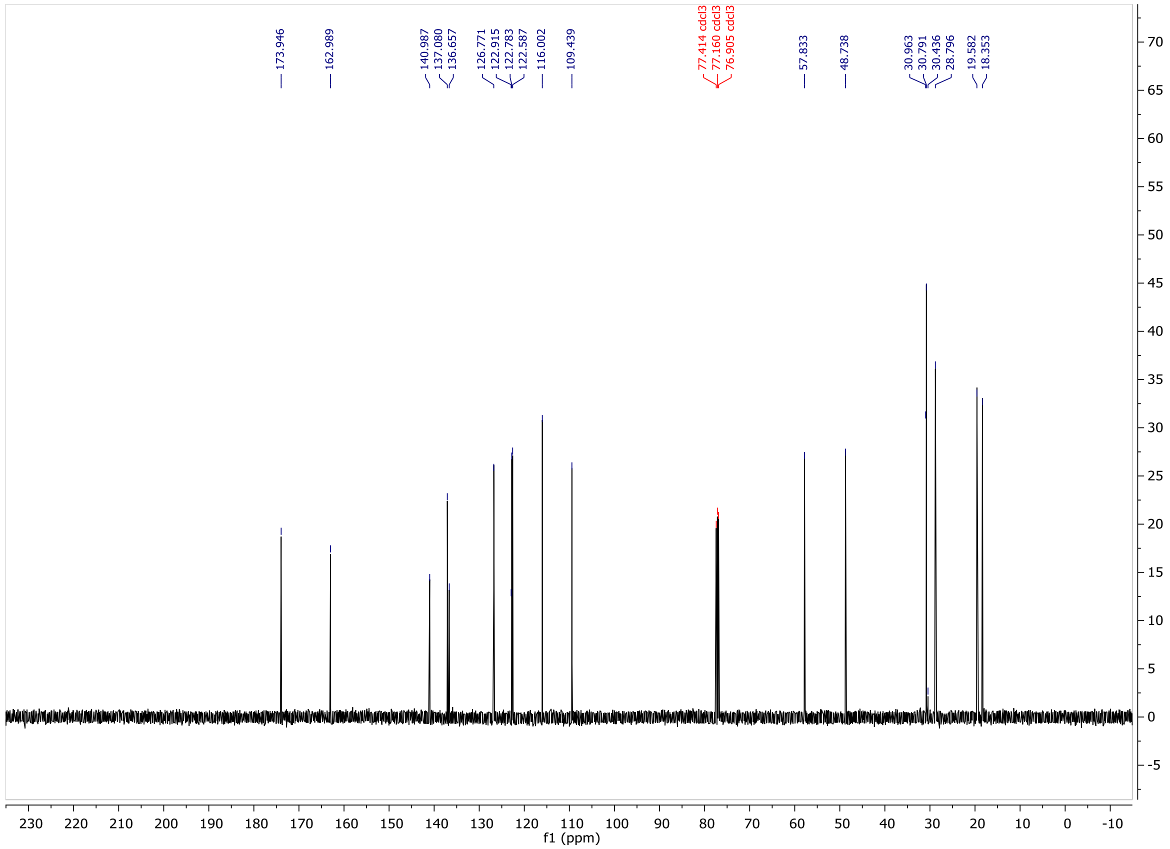

AB-4en-PINACA dihydrodiol (H3)

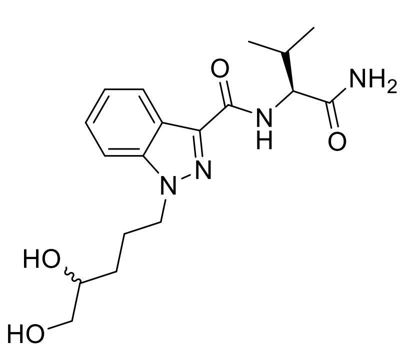

^{1}H-NMR Spectrum

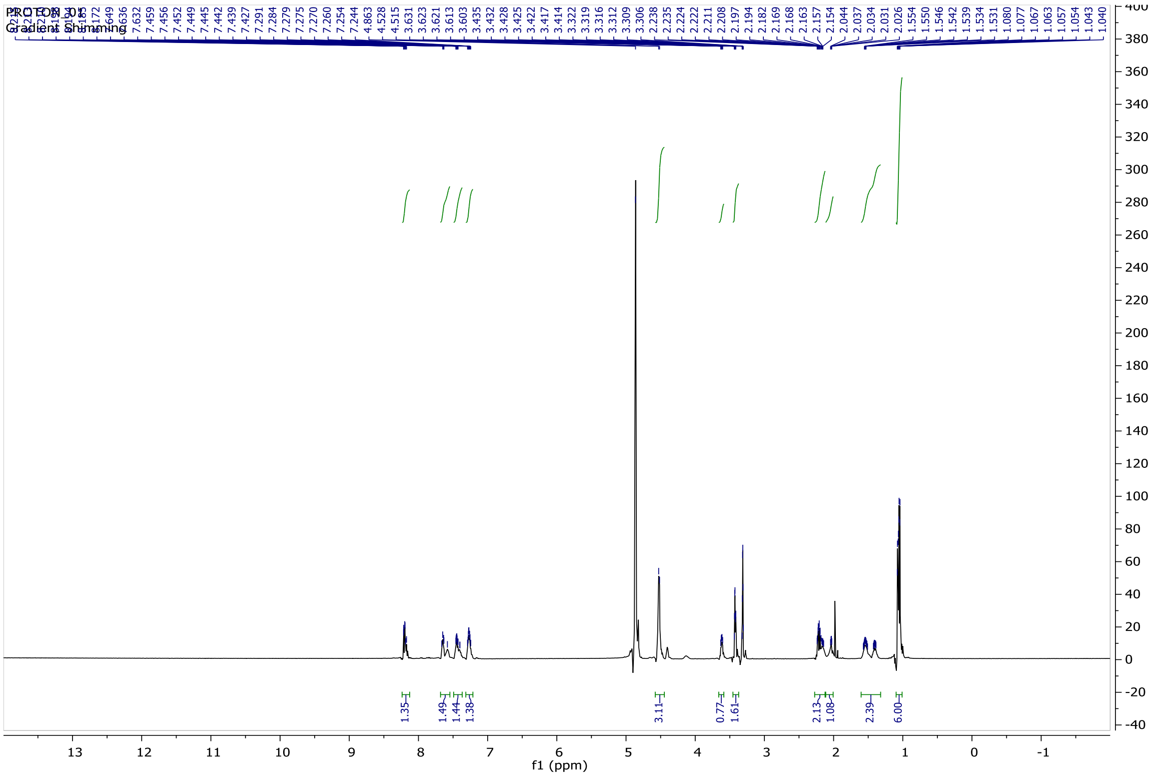

^{13}C-NMR Spectrum

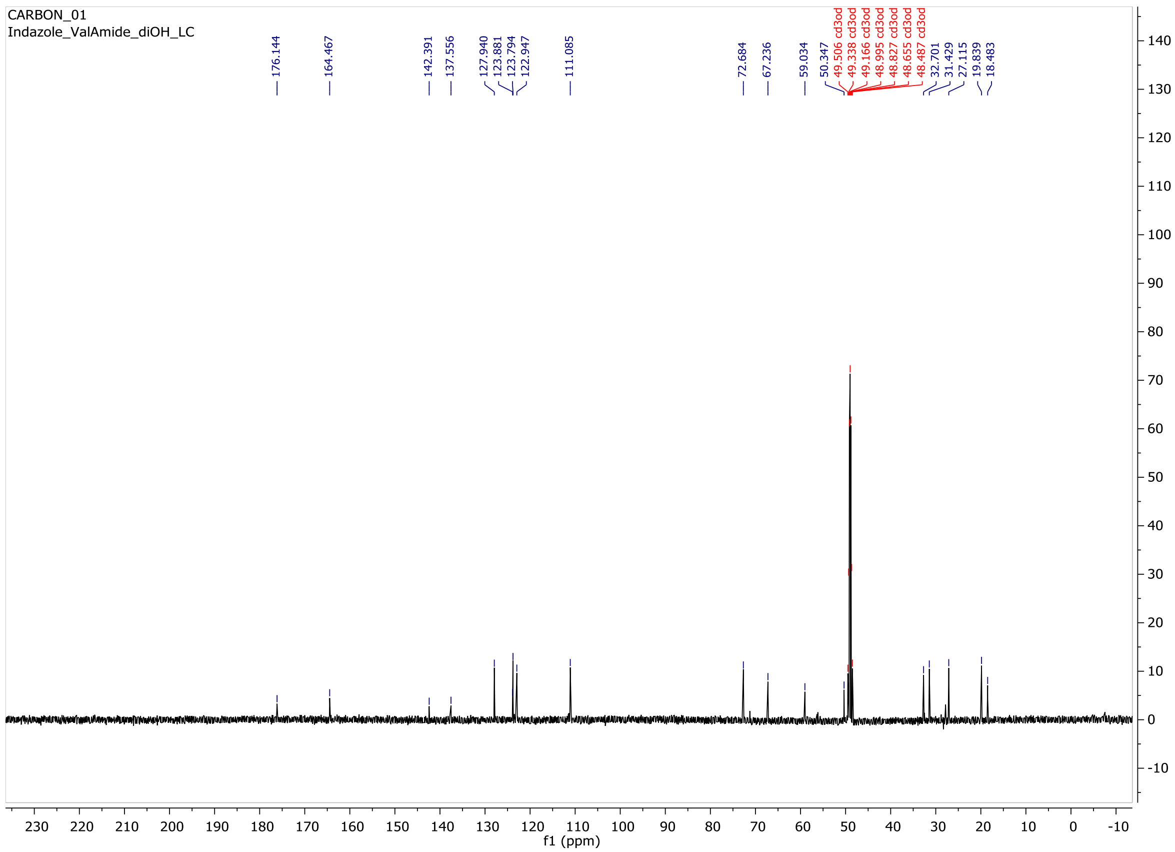

ADB-4en-PICA

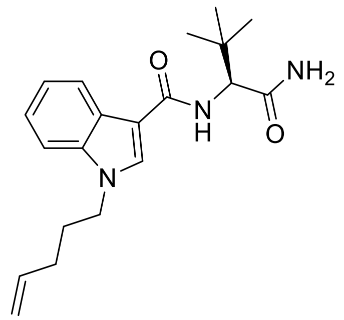

^{1}H-NMR Spectrum

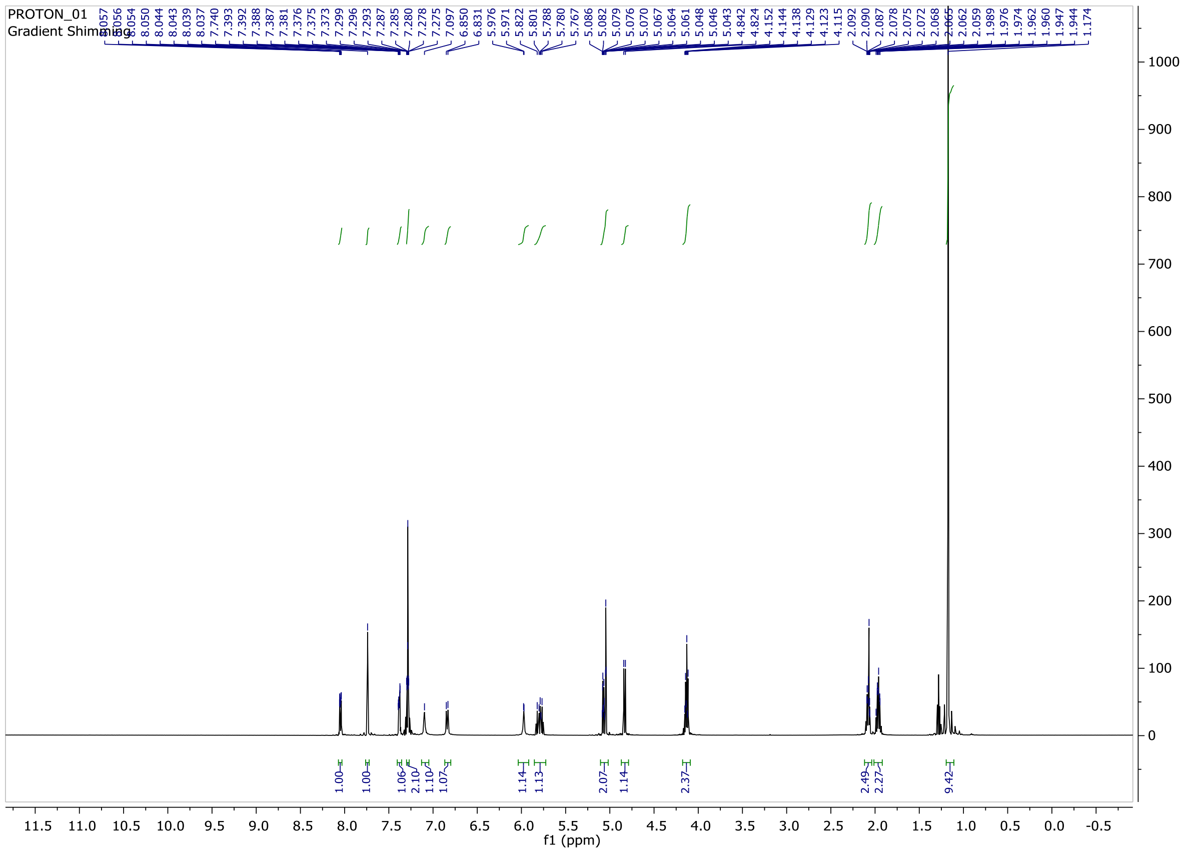

^{13}C-NMR Spectrum

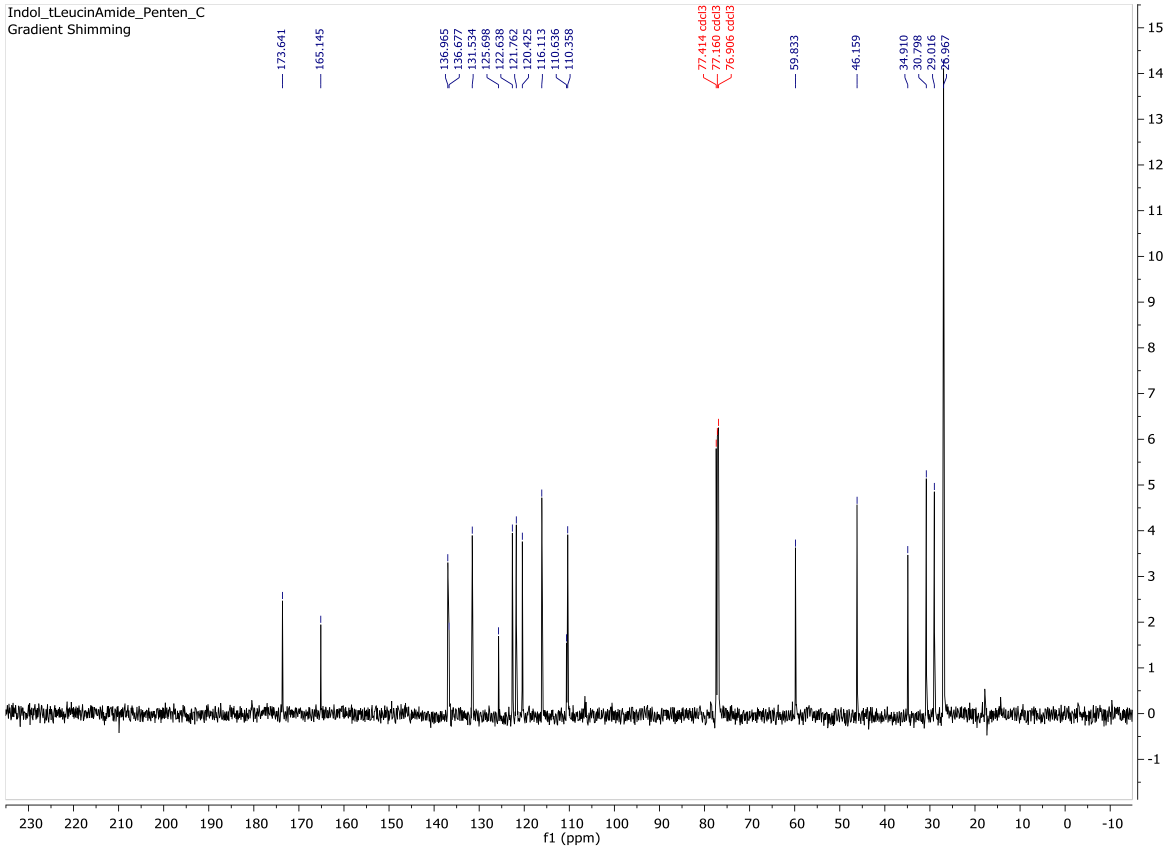

ADB-4en-PICA dihydrodiol (I2)

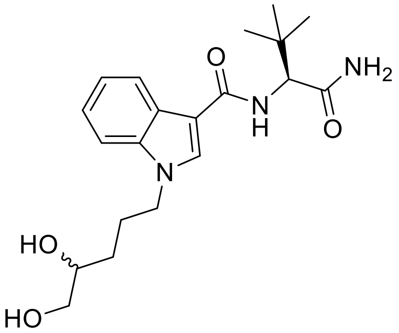

^{1}H-NMR Spectrum

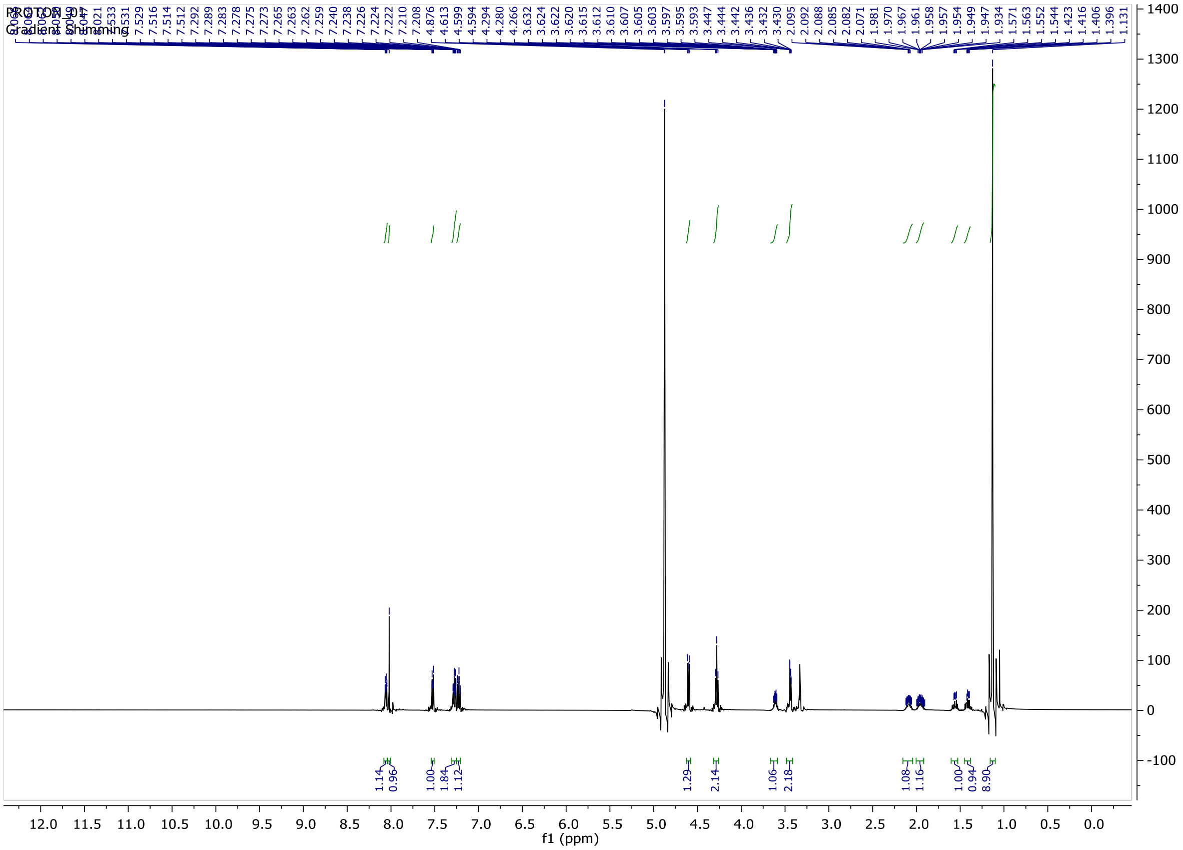

^{13}C-NMR Spectrum

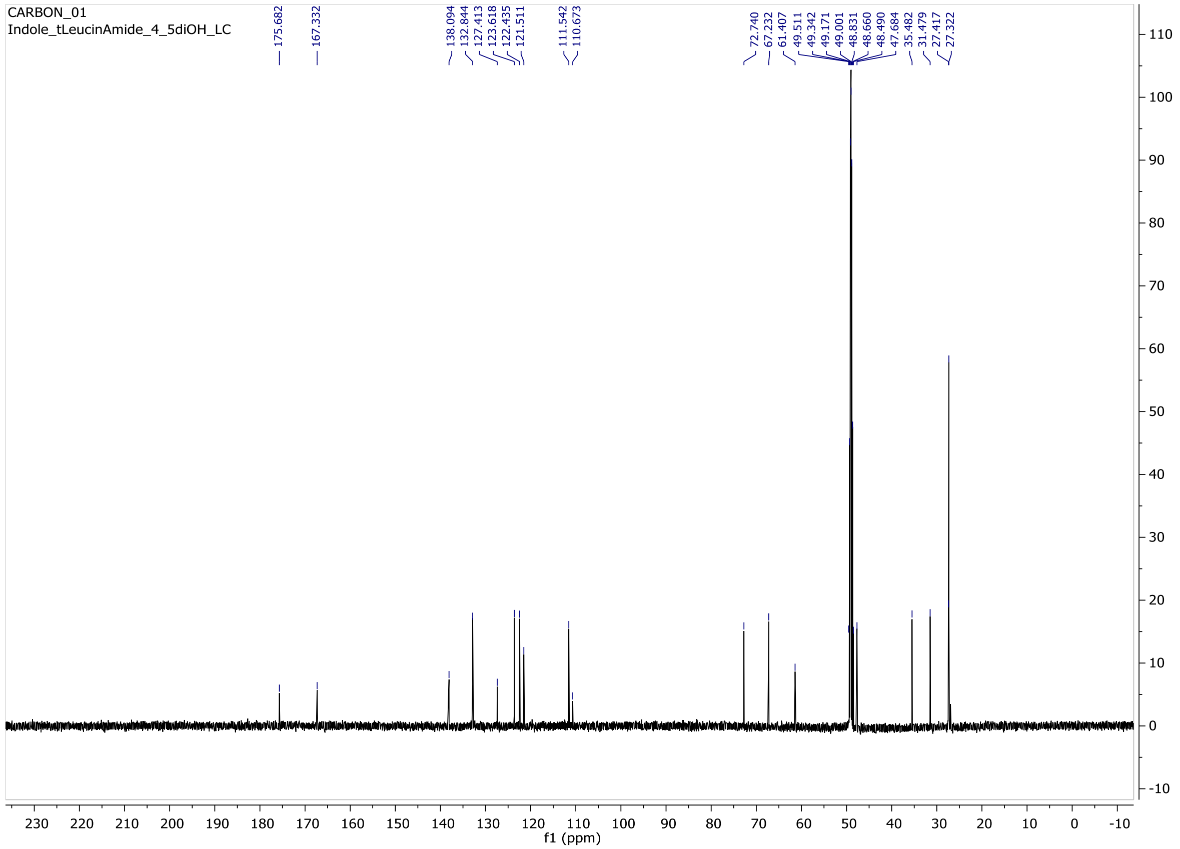

ADB-4en-PINACA

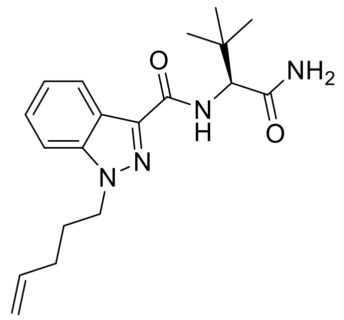

^{1}H-NMR Spectrum

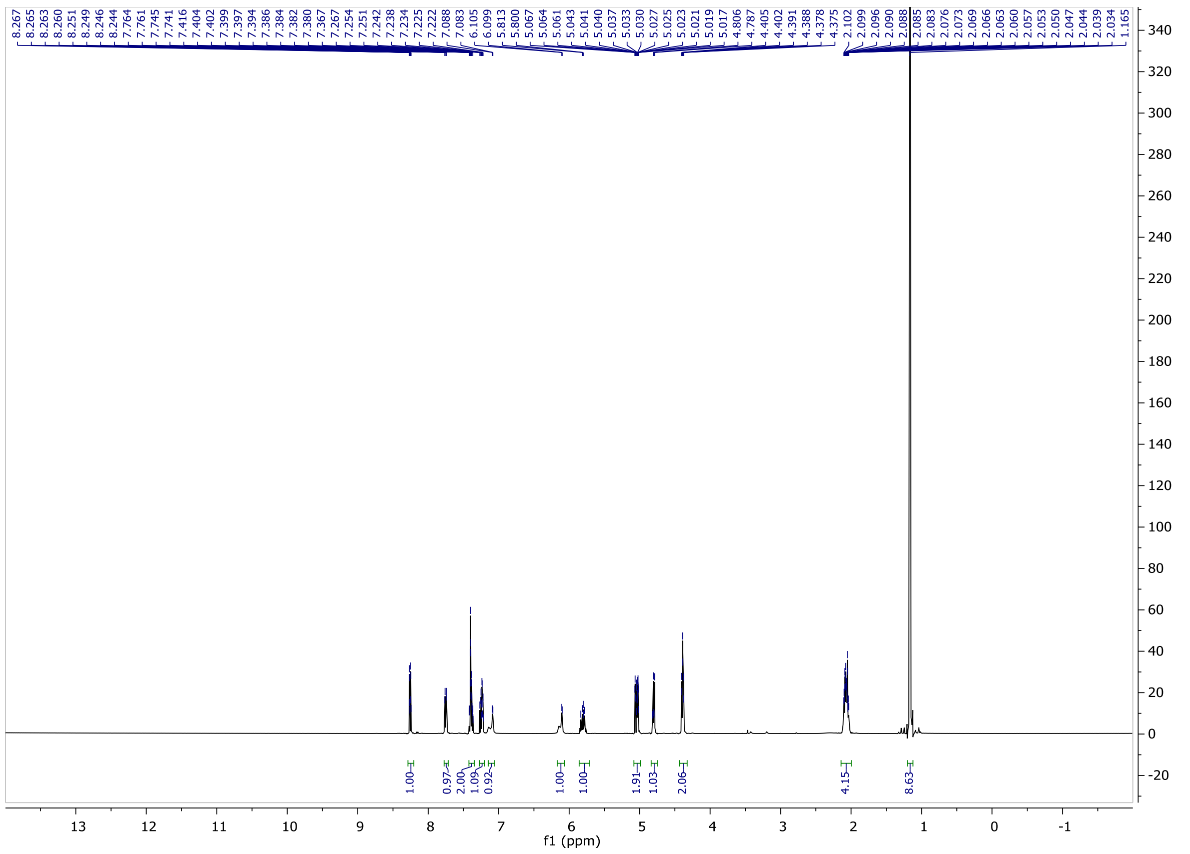

^{13}C-NMR Spectrum

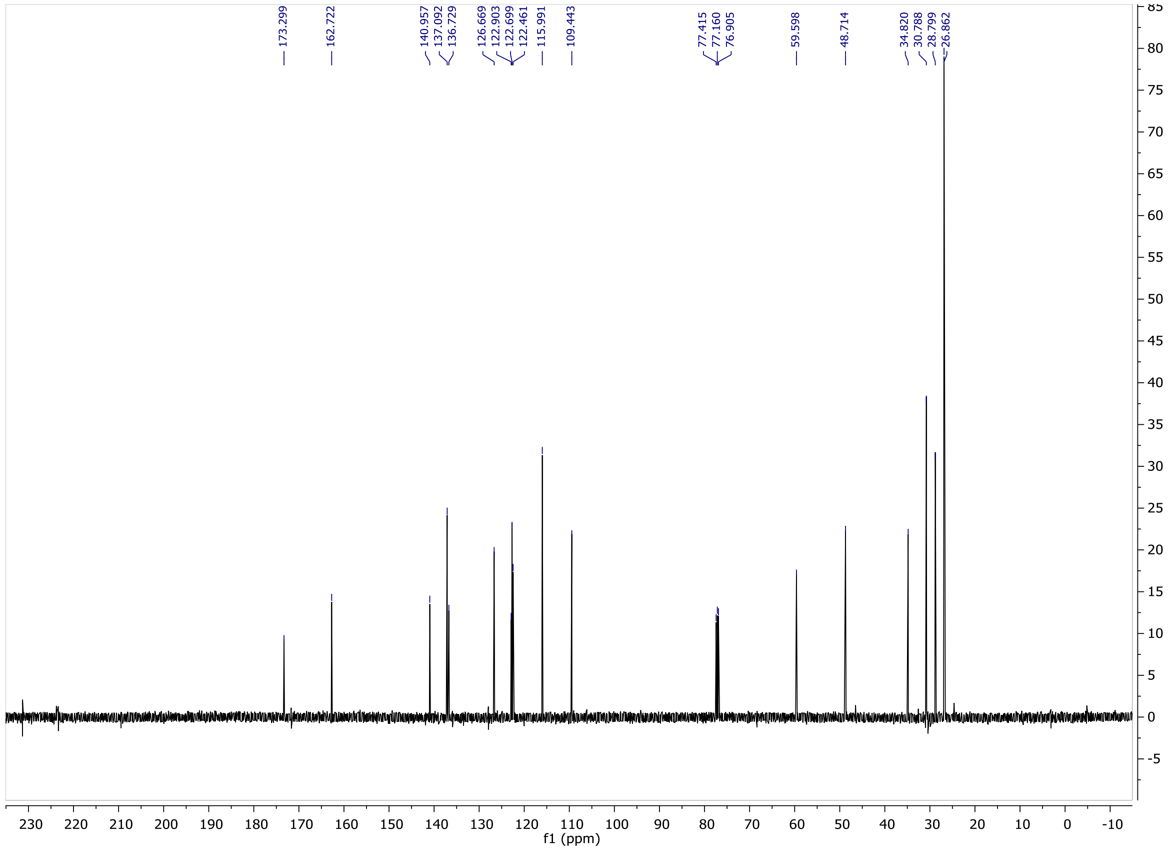

ADB-4en-PINACA dihydrodiol (J2)

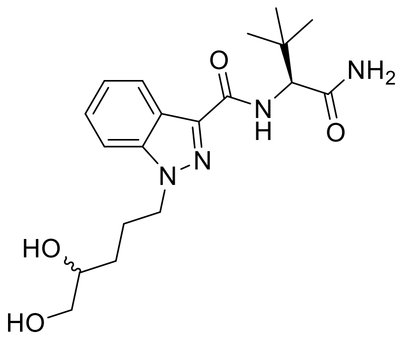

^{1}H-NMR Spectrum

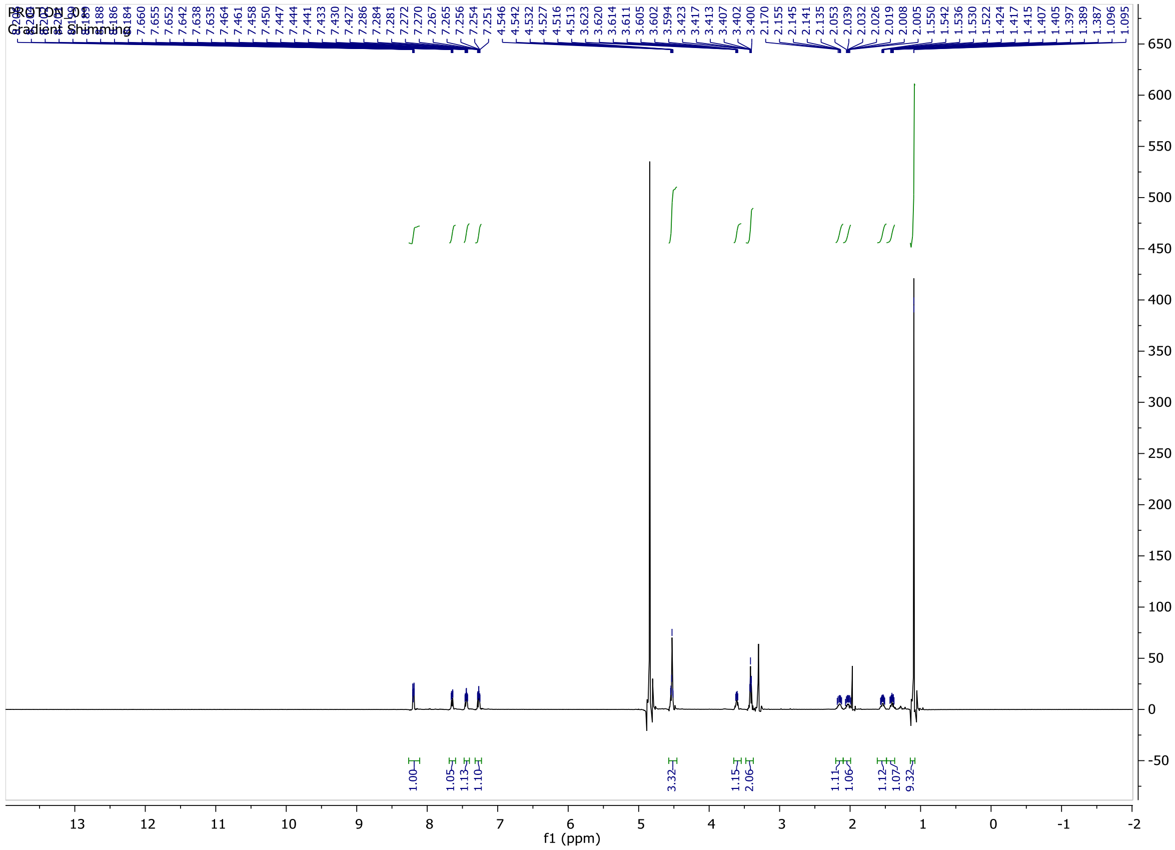

^{13}C-NMR Spectrum

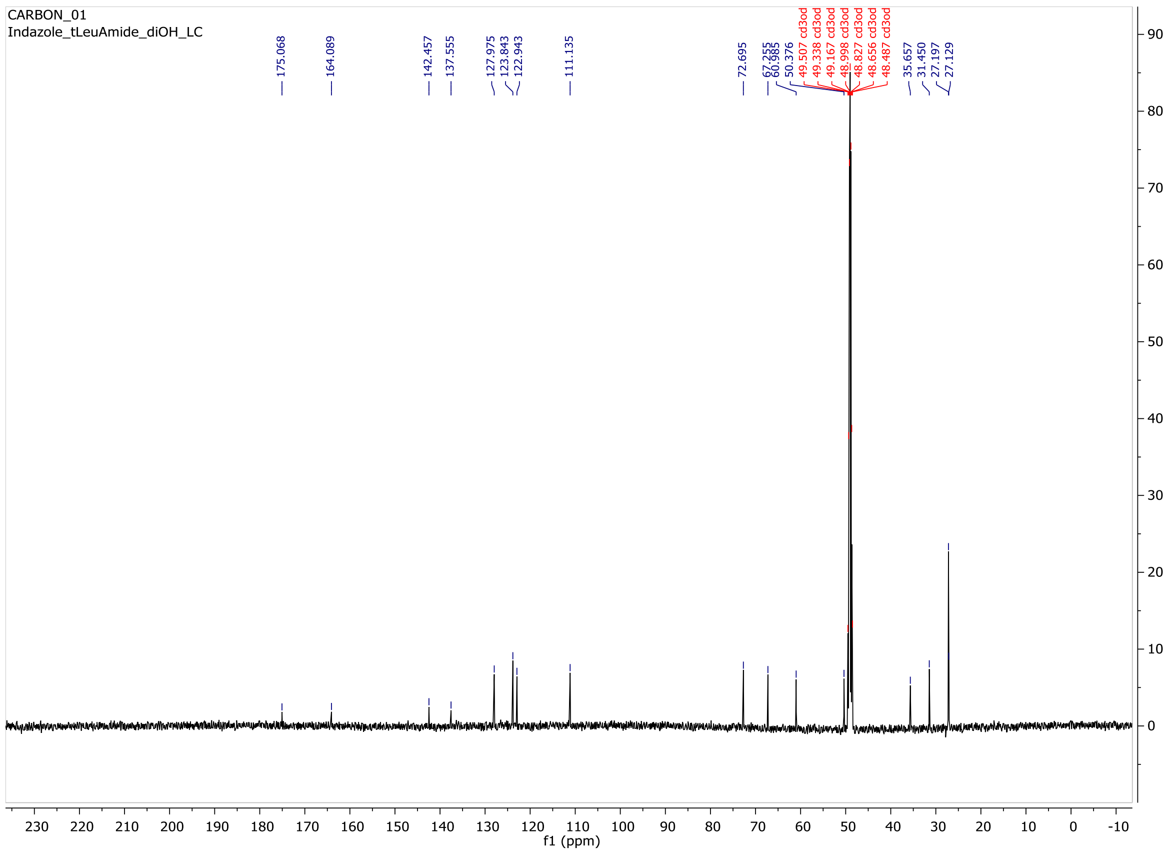

Supplement: Supplementary file 4 — Supplementary file4 (PDF 4726 KB) [file 204_2025_4080_MOESM4_ESM.pdf]
